# Compression of Large Sets of Sequence Data Reveals Fine Diversification of Functional Profiles in Multigene Families of Proteins: A Study for Peptidyl-Prolyl *cis/trans* Isomerases (PPIase)

**DOI:** 10.3390/biom9020059

**Published:** 2019-02-11

**Authors:** Andrzej Galat

**Affiliations:** Retired from: Service d’Ingénierie Moléculaire des Protéines (SIMOPRO), CEA-Université Paris-Saclay, F-91191 Gif/Yvette, France; andrzej.galat@sfr.fr; Tel.: +33-016-446-5072

**Keywords:** FKBP, cyclophilin, PPIase, heat-map, immunophilin

## Abstract

In this technical note, we describe analyses of more than 15,000 sequences of FK506-binding proteins (FKBP) and cyclophilins, also known as peptidyl-prolyl *cis/trans* isomerases (PPIases). We have developed a novel way of displaying relative changes of amino acid (AA)-residues at a given sequence position by using heat-maps. This type of representation allows simultaneous estimation of conservation level in a given sequence position in the entire group of functionally-related paralogues (multigene family of proteins). We have also proposed that at least two FKBPs, namely FKBP36, encoded by the *Fkbp6* gene and FKBP51, encoded by the *Fkbp5* gene, can form dimers bound via a disulfide bridge in the nucleus. This type of dimer may have some crucial function in the regulation of some nuclear complexes at different stages of the cell cycle.

## 1. Introduction

About 30 years ago, an exciting adventure began in finding some correlations between pharmacological activities of macrocyclic hydrophobic drugs, namely the cyclic peptide cyclosporine A (CsA), and two macrolides, namely FK506 and rapamycin, which have profound and clinically useful immunosuppressive effects, especially in organ transplantations and in combating some immune disorders. Curiously, it has been found that these molecules bind to abundant cytosolic proteins, which have significant potential to accelerate *cis/trans* isomerization of X-Pro bonds in synthetic peptides and proteins and which are called since then peptidylprolyl *cis/trans* isomerases (PPIases) [1,2,3]. At that time, only some fragmented information existed on the cytosolic CsA-binding protein (Cyclophilin-A, CyP-A) and a 12 kDa FK506-binding protein (FKBP12). Research on the immunosuppressive activities of these three molecules has, however, been rapidly advancing and has led to an ingenious proposition that there must be additional intracellular factors whose blocking of their enzymatic activity is the principal cause of immunosuppression. This reasoning was based on the fact that the intracellular concentration of cyclophilin-A and FKBP-12a are too high for being totally saturated by the immunosuppressive molecules under physiological conditions. For around 25 years, it has been believed that the immunosuppressive activity of the CyPA/CsA and FKBP12a/FK506 complexes are due to blocking the access to the activity site of calcineurin-A/calcineurin-B/calmodulin complex [4], which in turn causes retention of the phosphorylated form of the transcription factor (nuclear factor of activated T cells, NF-AT) that is a key factor of transcription of some cytokines. In contrast, the FKBP12a/rapamycin complex binds to a large kinase mTOR (mammalian target of rapamycin) and also hinders the access to its kinase activity site [5]. Both calcineurin and TOR are expressed in cells starting from yeasts and ending in mammalian organisms [3]. 

In this communication, we present some glimpses of analyses of a very large number of sequences of the FK506-binding proteins (FKBPs) and cyclophilins. Analyses of several thousands of sequences that were compressed and displayed as heat-maps revealed how their functionally-crucial amino acind (AA)-residues had evolved in disparate organisms. Consideration of these massive data in light of some rather limited knowledge of physiological functions of PPIases and their structural attributes allowed derivation of several novel hypothesis, which could eventually be interesting for further investigation.

## 2. Materials and Methods 

### 2.1. BLAST Searches

The non-redundant protein sequence database (NrPSD) and several genomic databases assembled in the National Center for Biotechnology Information, NCBI (http://ncbi.nlm.nih.gov) were used [6]. Database searches were made with the BLAST program, accessible via the NCBI server [7]. We used the following inputs: 1° the entire sequences of diverse small cyclophilins and FKBPs; and 2° the PPIase domains of large cyclophilins and FKBPs. Coherent groups of orthologs of 17 human cyclophilins and 12 human FKBPs were obtained if the upper cutoff limit was set up to 1000 sequences. We used a combination of in-house-made software and a manual approach for the selection of a set containing 8054 sequences of cyclophilins out of 17,000 hits and 7793 of the FKBPs out of the 12,000 BLAST hits (Figure S1A,B, supplementary materials). Some sequences were removed, such as artificially-produced sequence variants, synthetic sequences, sequences coming from X-ray structures, sequence fragments and some duplicated entries. This step was extremely time consuming and accounted for about 95% of the work described in this note. Those efforts however allowed to create preliminary sets of coherent sequences of PPIases that were processed by a series of software as it has been described in my previous papers [8,9]. For more sophisticated applications of the described methodology, normally we need several additional ‘purification’ cycles, which in each case requires many dozens of Seq_gen and ClustalW executions followed by manual selection and corrections. All MSAs have the same order of sequences as it has been supplied by BLAST.

### 2.2. Multiple Sequence Alignment

The sorted-out sets of sequences from BLAST [7] searches were aligned with the multiple sequence alignment (MSA) program Clustal-1.83W [10]. Also, the MUSCLE program (EMBL-EBI) was used in some cases [11,12].

### 2.3. Calculation of Some Sequence Attributes and Their Statistics

Computing procedures for hydrophobicity profiles, the overall hydrophobicity indexes (HIs), the theoretical isoelectric points (pIs) and some statistical measures such as standard deviation (σ), skewness and kurtosis of these sequence attributes were recently described [13].

### 2.4. A Strategy for Analyses of Large-Scale Multiple Sequence Alignments

We used the following strategy for derivation of AA changes in the MSAs of 21 FK506-like binding domains (FKBDs) and 17 cyclophilin like domains (CLDs) that are orthologues of their human counterparts. 1° Shannon’s information entropy *I*_ej_(A) at position j of the MSA was calculated from the following formula:

$$I_{\mathrm{ej}}\left(x\right)=\sum_{x=1}^{20}p_{\mathrm{xj}}Log\left(p_{\mathrm{xj}}\right)$$

where *p*_aj_ is the probability of the appearance of amino acid residue of given type a, for example, Ala, at position j. The fully conserved sequence position in a given MSA has *I*_ei_(*x*) = 0.0 (uncertainty of information entropy *I*_e_ = 0.0) whereas the maximal value of *I*_ei_(*x*) may exceed 3 if scaled down to the natural logarithm and remains ≤1 if scaled down to the logarithm of base 20. Shannon’s entropy values equal or close to zero imply that given sequence position is well conserved whereas larger values indicate for some amino acid (AA) variability in the position. 2° We used Simpson population diversity index (PDI) as a measure of diversity of AAs in each column of the MSA. We divided AA-residues for the following eight physical-chemical types: (1) hydrophobic: Ala, Ile, Leu, Val; (2) aromatic: Phe, Tyr, Trp, (3) small size: Gly; (4) special properties: Cys; (5) hydrophilic: Asn, Gln, Ser, Thr; negatively-charged Asp, Glu; (8) positively charged: R and K. Simpson PDIs equal or close to zero indicate that the position is occupied by AA residues with similar physical-chemical profiles whereas values higher than average implies that the position contain AA residues of different physical-chemical profiles. The following formula was used to calculate the Simpson PDIs, where k is the number of types of AAs, *n* is the number of amino acids (AAs) residues having the given physical-chemical type and *N* is the total number of AAs in the column:

$$\mathrm{Simpson}\;\mathrm{DPI}=1-\left\{\sum_{i=1}^{k}\left(n\left(n-1\right)/\left(N\left(N-1\right)\right)\right)\right\}$$

**Figure 1:**
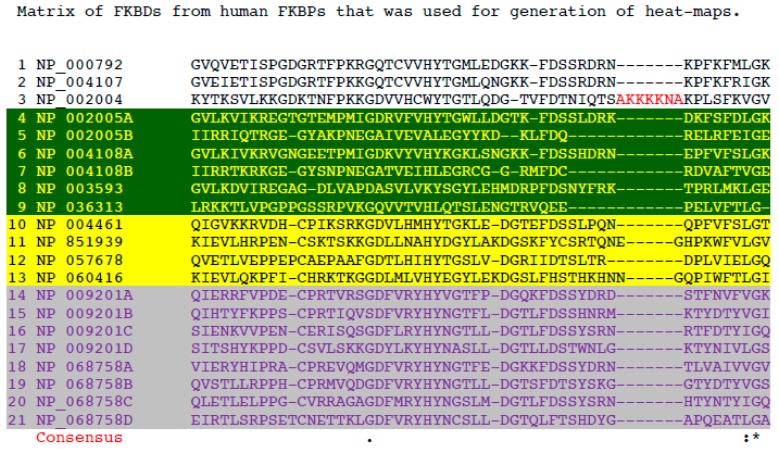

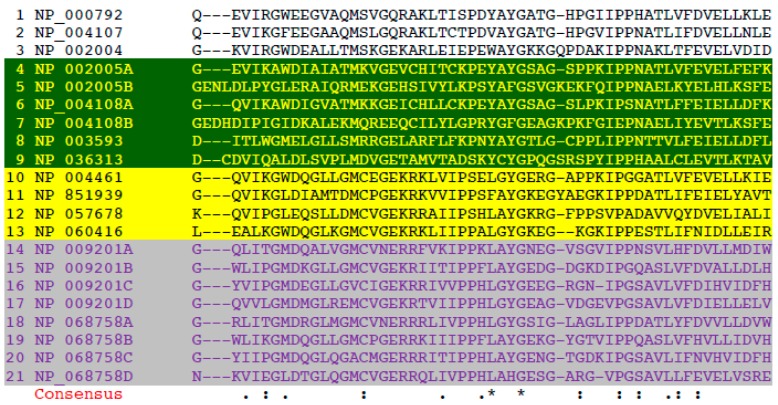
Matrix of human FK506-like binding domains (FKBDs) used for the construction of the heat map shown in Figure 3. 1— FKBP12a. 2—FKBP12b; 3—FKBP25; 4 and 5—first and second FKBDs in hFKBP52; 6 and 7 first and second FKBDs in hFKBP51; 8—FKBP36; 9—hFKBP38; 10—FKBP13; 11—FKBP encoded by the *Fkbp7* gene; 12—FKBP encoded by *Fkbp11* gene; 13—FKBP encoded by the *Fkbp14* gene; 14 to 18, the FKBDs of hFKBP9; 19—22, the FKBDs in hFKBP10.

**Figure 2:**
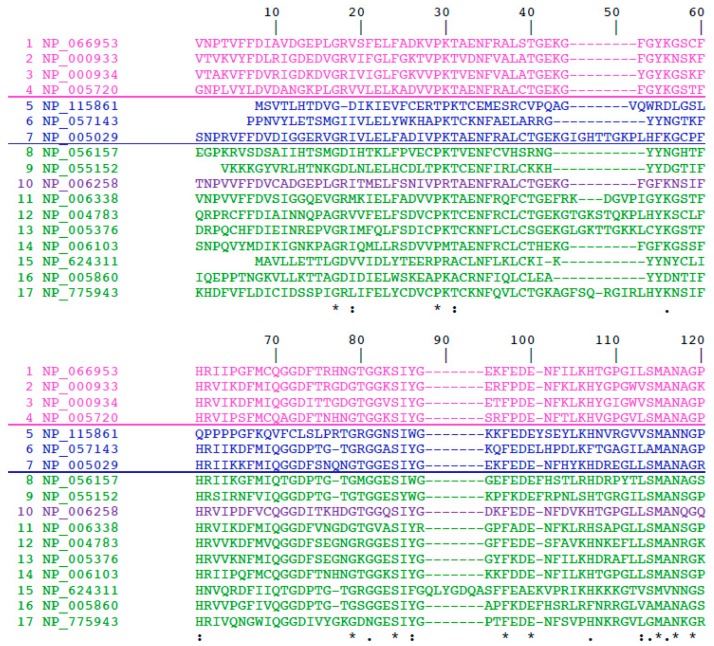

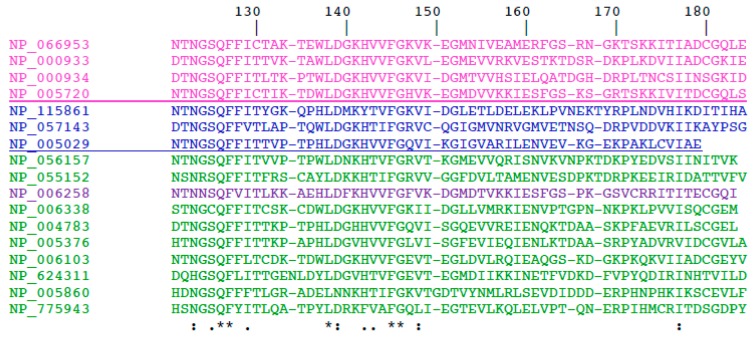
Matrix of human cyclophilin used for construction of heat-map. 1—Cyclophilin-As (CyP-A) (cytosolic); 2—CyPB (endoplasmic reticulum (ER)-retained cyclophilin), 3—CyPC; 4—CyPF (mitochondral cyclophilin); 5 and 6 and PPIL3A and PPIL1 small spliceosome-associated cyclophilins encoded by the *Ppil3a* and *Pil1* genes; 7—CyPD (40 kDa heat-shock cyclophilin, cytosolic); 8—cyclophilin containing WD-domains; 9—CyP58 and its orthologues; 10—325 kDa cyclophilin associated to nuclear membrane; 11—CyP19—spliceosome associated small cyclophilin; 12—large splicesome-associated cyclophilin; 13—large spliceosome-associated spliceosome; 14—CyP30, spliceosome-associated; 14 to 17—spliceosome-associated cyclophilin.

The 3° ensemble of calculated Shannon’s information entropy and Simpson PDIs for the columns in the MSAs of each group of orthologues FKBPs, and cyclophilins, were displayed as heat-maps, which were enriched with some pertinent information derived from analyses of the X-ray structures of some immunophilins. Heat-maps were constructed from the templates shown in Figure 1 and Figure 2, which were made from the aligned sequences of PPIase domains of human FKBPs and cyclophilins, respectively. Information entropy values and Simpson population diversity indexes calculated from the MSAs of a given set of the cyclophilins, and for a given set of the FKBPs, were overlaid in the 2D matrix (heat-map) whose colors were chosen with the Word package. The following coloring system was used: for values between 0.0 and 0.25 (white); for ≥0.25 green; and those approaching 1 in red. Heat-maps were created with Excel version16.16.6 (190114), which was purchased from the Microsoft Corporation (Redmond, WA, USA). The left-side column corresponds the numeration of AA residues in the reference matrix/template MSA, namely Figure 1 and Figure 2.

### 2.5. Analyses of Crystallographic Structures

X-ray structures were drawn with the PyMol program, which was purchased from the Schrodinger Company [14]. Some X-ray structures of the cyclophilins and FKBPs were downloaded from the Protein Data Bank (PDB) database [15].

### 2.6. In Home-Made Software

In Appendix A is given an overall presentation of sequential utilization of these three programs. In Figure S2A (supplementary materials) we give the source code in Fortran 77 for a short program named Data_gen that can transform flat GenBank entries downloaded from BLAST searches into database of sequences. It generates several other files, such as data.rem (summary of entries), data.fas (sequences in FASTA format), data.cod (all codes of sequences arranged for further processing). In Figure S2B we show the source code of the Seq_gen program (supplementary materials). Seq_gen program, which takes the data.cod file with chosen sequences as the input file that contains sequences (entire or trimmed), which are ready to the ClustalW1.83 multiple sequence alignment program (EMBL-EBI located at the Wellcome Genome Campus in Hinxton, Cambridge, UK). It also calculates some sequence attributes and statistics of the chosen sequences. In Figure S3 we give the source code of the Map_display program (supplementary materials). Electronic versions of source codes and their compiled versions on High Sierra Macintosh (10.13.6, Apple Inc., Silicon Valley, Cupertino, California, USA.) with several examples of input modules are available from the author for persons working in non-profit organizations. We used the Absoft18.0 Fortran compiler that was purchased from the Absoft Company (Keego Harbor, MI, USA). The programs were executed on a MacOS High Sierra operating computer. Also, the GNU version of Fortran 77 [16] compiler was used and its newer version Fortran 90/95 [17], thus those who cannot afford for Absoft18.0 could still compile my programs with the publicly available software. 

## 3. Results 

### 3.1. FK506-Binding Proteins 

**Figure 3:**
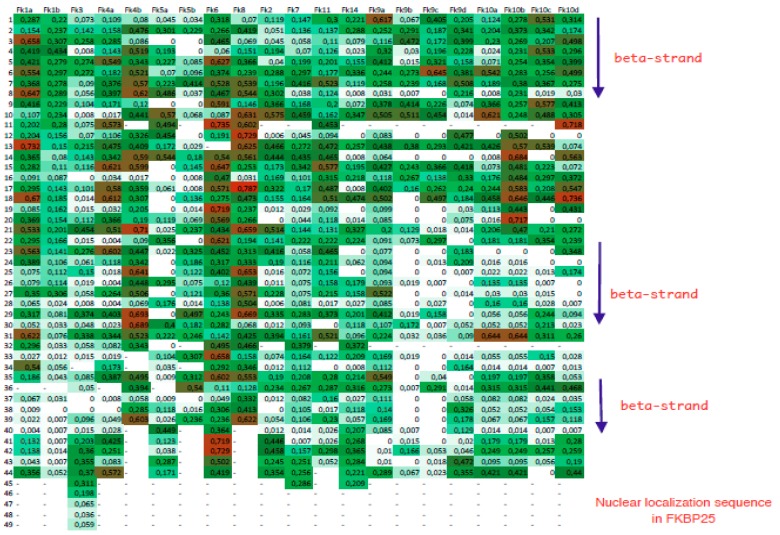

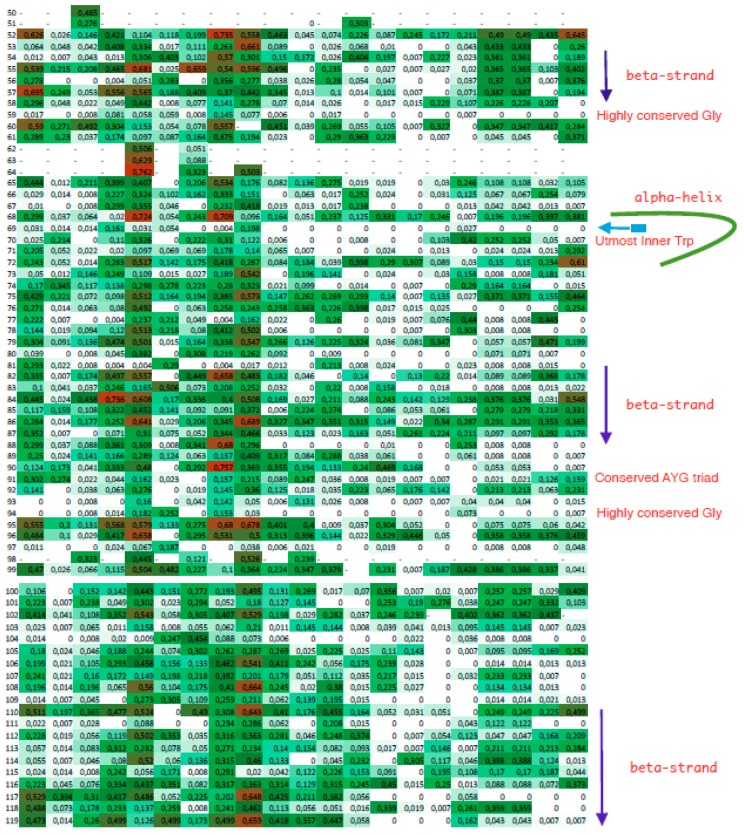
Heat-map of the FKBPs encoded in various genomes. Fk1a and Fk1b—FKBP12a and FKBP12b, respectively; Fk3—FKBP25; Fk4a and Fk4b, N-terminal and C-terminal FKBDs in FKBP52; Fk5a and Fk5b, as above for FKBP51; Fk2, Fk7, Fk11, Fk14 correspond to endoplasmic reticulum specific FKBPs, namely FKBP13, FKBP23, FKBP19, and FKBP22, whereas Fk9a, Fk9b, Fk9c and Fk9d as well as Fk10a, Fk10b, Fk10c and Fk10d correspond to the four FKBDs starting from the N-terminus of FKBP63 and FKBP65.

Archetypal FKBD has from 10 to 12 kDa. Large FKBPs are fusion proteins containing up to four FKBDs and several different domains, such as WD40 repeats (β-transducine repeats), tetratricopeptide repeats (TPRs), RNA-recognition domain (RRM), and different others [18]. The FKBPs have wide phylogenetic distribution, although their prokaryotic forms have unique domains that are not present in the FKBPs from higher organisms [19]. A heat-map summarizing Shannon’s information entropy values derived from 7793 sequences of the FKBDs is shown in Figure 3, whereas the heat-map comprising Simpson PDIs is shown in Figure S4 (supplementary materials). The heat-maps do not include the FKBDs from 135 kDa FKBP, namely FKBP15 and aryl-receptor interacting protein (AIP; FKBP37), which have low sequence similarity with the majority of the other FKBPs. 

There are several particular features of the sequence, such as red/ox disulfide bonds in all five FKBP embedded in the endoplasmic reticulum ER-FKBPs. These ER-retained FKBPs seem to have a better level of sequence conservation than the other FKBDs as illustrated by many white fields in the heat-map. It could be due to their specialized activity as folding chaperones of specific sets of proteins. The other FKBDs have a lesser level of sequence conservation, FKBP1a especially seems to have rather divergent sequences. 

**Figure 4:**
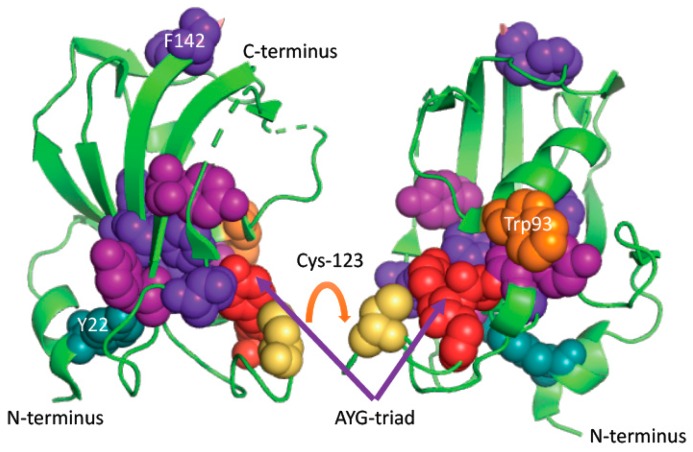
X-ray structure of the FKBD domain of hFKBP36. Cys123 (yellow) is surface-exposed and could form intramolecular disulfide bond and give rise to a dimer as shown by an arrow. Phe residues are in violet, Tyr are in forest color whereas highly conserved triad in the FKBPs (AYG and its variants) is a part of the long loop linking the C-terminusb b-strand with the rest of the molecule. It is in red as shown by an arrow.

Examination of the X-ray structures of human FKBP36 (3B7X.pdb) that is encoded by the *Fkbp6* gene [20] revealed that it has one free surface-exposed cysteine residue, which could form a disulfide bond with each other by linking two PPIase domains; its hypothetical structure is illustrated on Figure 4. It is noticeable that this Cys-residue is well conserved in the FKBP36 from different *phyla* (see Figure S5A,B, supplementary materials). Overall sequence similarity of the analyzed sequences of Fkbp6s is about 53% although its distant homologue expressed in *Drosophila* has about 33% similar to its human counterpart. Both proteins are indeed very hydrophobic.

**Figure 5:**
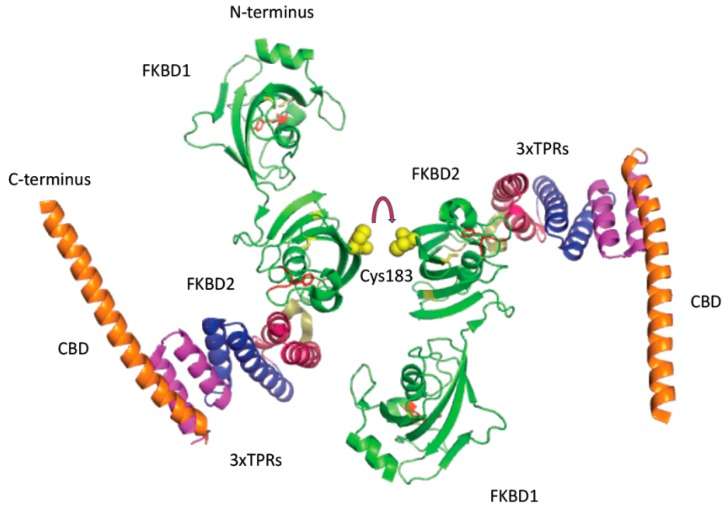
X-ray structure of the FKBP51 with its two FKBDs and the surface exposed Cys153 in FKBD2. FKBD1 and FKBD2 are in green with the AYG triads in red. Three consecutive tetratricopeptide repeats (TPRs) (α-helical structures) are in deep red, blue and magenta, respectively. C-terminus forming a long α-helix is in orange. This helix may have some affinity to calmodulin and was named here calmodulin-binding domain (CBD).

Human FKBP51 (5OMP.pdb) that is encoded by the *Fkbp5* gene [21], has three Cys-residues in FKBD-2, but one of them, namely Cys183, is surface exposed in Figure 5. Cys183 in the second FKBD of hFKBP51 was largely well-conserved in the FKBP51s encoded in genomes of different phyla (Figure S6 in supplementary materials). Moreover, Cys103 and Cys107 (FKBD1 domain) and Cys176 with Cys215 (FKBD2 domain) are well hidden in the interion of the protein.

It is considered that PPIases are, in principle, monomeric proteins that can associate with different targets in the cell. Our analyses suggest that in some cases dimeric forms are possible, which are linked by a disulfide bond. Such forms may function in the ER or nuclear space. It is crucial to note that Cys123 is very well conserved from mammals to *Drosophila melanogaster*. Hypothetic dimeric forms of these two FKBPs, namely FKBP36 and FKBP51, could, however, create powerful regulatory molecules of some nuclear events where disulfide bonds are stable. In the cytosol, FKBP51 probably exists as a monomeric form. Surface-exposed Cys123 in hFKBP36 could form a disulfide bond and become a crucial factor in the nuclear events related to later stages of oogensis of the germline stem cells [22]. In fact, it has been shown that a distant orthologue of hFKBP36 is a crucial factor in germline cells during development of *Drosophila melanogaster*, which was called shutdown [22]. Such a dimeric construct of FKBP36 in the nucleus could gain a novel function by binding with both PPIases units of two different epitopes of an entity, thereby making such entities crucial factors, which are ready for binding to some transcriptional complexes and promoters. That would make FKBP36, beside FKBP25 [23,24], a second DNA-binding FKBP via its association with oxysterol-binding protein (OSBP). Although both FKBD in hFKBP36 and FKBD-2 in FKBP51 do not have PPIase activity, their hypothetical in-situ formed dimes in the nucleus may acquire non-standard PPIase activity and participate in some crucial nuclear events.

### 3.2. Cyclophilins

**Figure 6:**
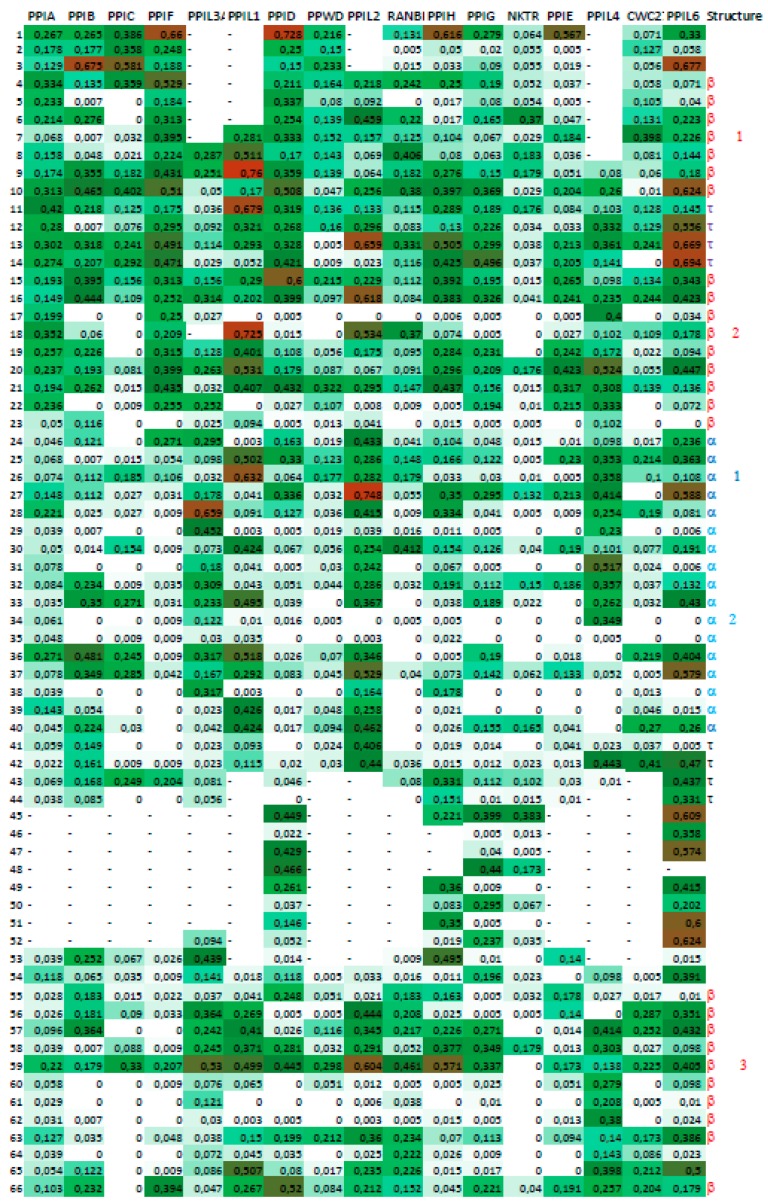

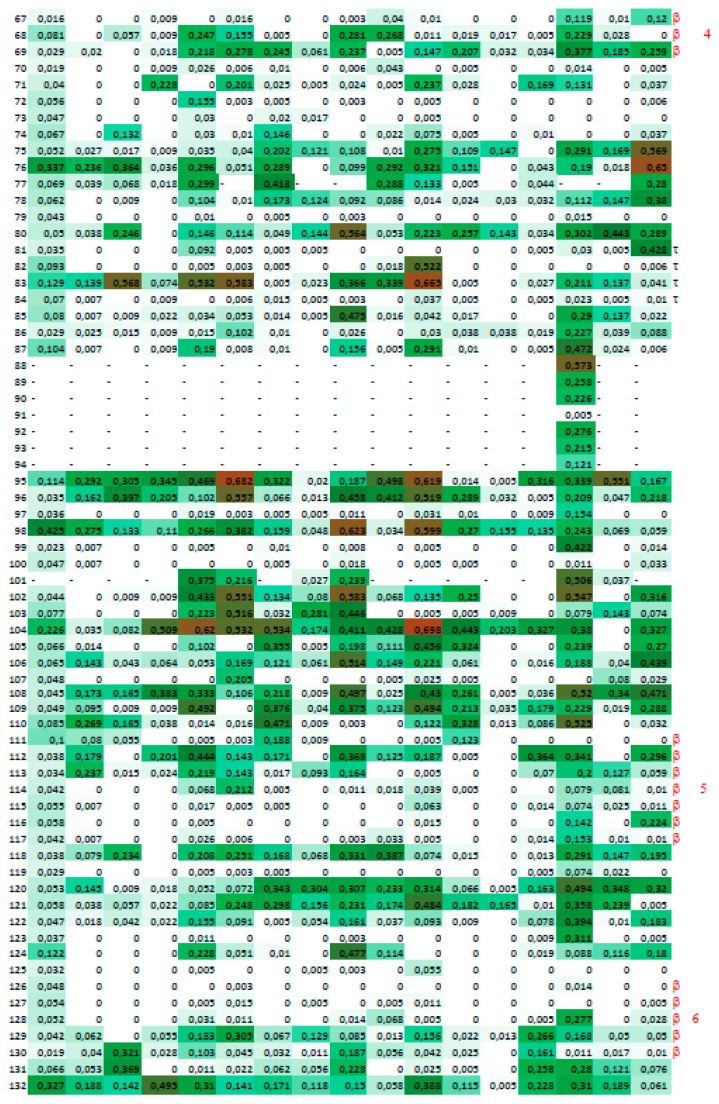

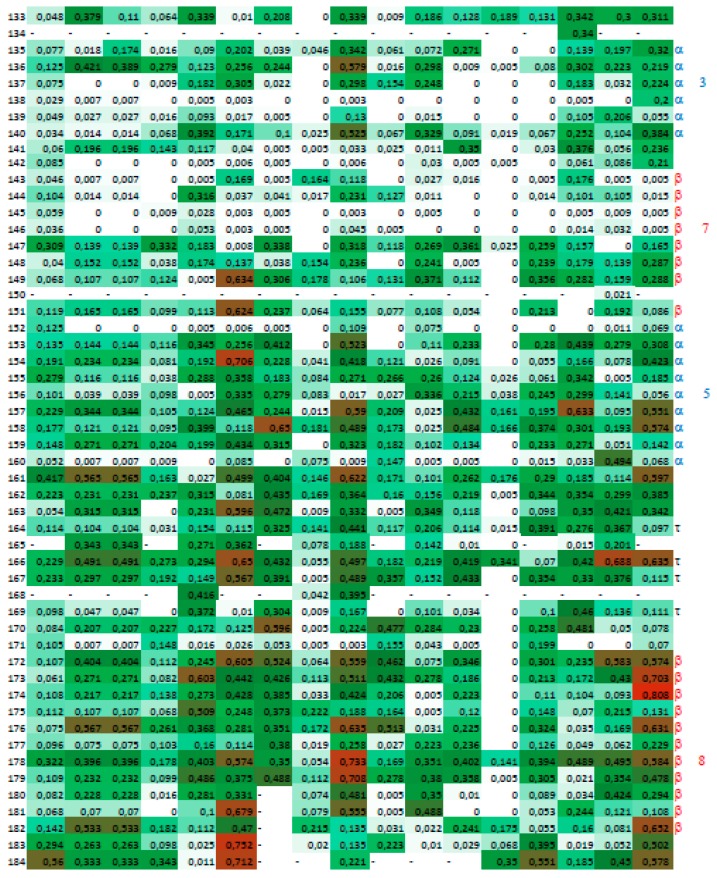
Heat-map of the cyclophilins encoded in various genomes. Vertical sequence alignments (Figure S7A and vertical MSA of the cyclophilins, Figure S7B, supplementary materials) could be useful for finding some correlations between the first column that has the numbering taken from Figure 1 and Figure 2, respectively. The order of vertical lines is as described in Figure 2. We used the names of genes coding each of the analyzed paralogue of human cyclophilins and its orthologues encoded in the genomes of disparate species.

The cyclophilins have a wide phylogenetic distribution starting from prokaryotes and ending in mammalian organisms. Monodomain cyclophilins have molecular mass varying from 16 to 18 kDa, whereas several multidomain proteins encoded in diverse genomes have always only one CLD, and their masses vary from 25 to 330 kDa. An overview of the X-ray structures of the cyclophilins has been published [25]. It shows that despite overall similar 3D structures, the cyclophilins have many unique geometrical features that can distinguish each from the others. The physical-chemical attributes of human cyclophilins have been recently reviewed [8] and here we describe only conservation of some sequence and functional features derived from the heat-maps created from the matrix of the aligned human cyclophilins (Figure 2). A total of 8054 sequences of cyclophilins from various organisms were analyzed and displayed on a compressed heat-map. In Figure 6 Shannon’s information entropy values for the CLDs of the orthologues of 17 human cyclophilins are shown, whereas Simpson PDIs are shown in Figure S7 in supplementary materials. 

Analyses of Figure 6 shows that the N-terminus and C-terminus segments in most of the cyclophilins have rather low sequence conservation, but their central segments have high positional conservation levels. hCyP157 and its orthologues (CyP157s) encoded in different genomes have the highest sequence conservation, which can be seen as white vertical spaces in the heat-map (Figure S8A,B in supplementary materials). Likewise, hCyP88 and its orthologues, as well as CyPBs and CyPCs, have a good sequence conservation. Even if some sequence positions viewed in a horizontal way have relatively low or equal to Shannon’s information entropy, several positions in this line may have somewhat higher values. Those multiple positions of rising values (seeing in horizontal sense) probably represent discrete conformational spaces, which permit replacement of amino acid side chain (AA-mutation). The integrated form of such discrete conformation spaces in a given protein is the driving force for the acquirement of functionally-diversified properties via AA-substitutions. 

## 4. Discussion

In this technical note we have presented several issues on how to deal with a large number of sequences of the multigene families of proteins, which have the archetypal sequence and various numbers of paralogues. Shannon’s information entropy values and Simpson PDIs displayed on the heat-maps show quantified and compressed forms of a set of MSAs, which certainly allow a better insight into conservation of sequence positions. Due to the different mutation rates in DNA coding sequences, which allow the refinement of various cellular and systemic adaptation processes and their integration in disparate organisms, the archetypal sequence has lesser sequence similarity to some of its paralogues than to the others. This was the main reason why BLAST searches supplied variable numbers of ‘true’ orthologues for each paralogue of human FKBPs or cyclophilins. For example, hFKBP38 has a low sequence similarity to the other paralogues of hFKBP12a, thus the search supplied a long list of FKBP38s encoded in different genomes (Figure S9A,B in supplementary materials). Moreover, FKBP38 is a mitochondrial protein that is encoded in the organisms ranging from mammals to those of a lower level of development. In contrast, searching the protein database with the sequence of hFKBP12a supplied both FKBP12a’s and FKBP12b’s, because their sequences are very similar. Some of the other FKBPs, such as those expressed in the ER, supplied comparable numbers of ‘true’ orthologues (between 300 and 400) encoded in different genomes. This would imply that essentially all available data coming from genome sequencing projects have been found by BLAST. 

The heat-maps summarize the level of conservation of sequence positions (Shannon’s information entropy values) and physical-chemical character of amino acids (Simpson PDIs). However, such massive amounts of data illustrate only overall conservation of positions in sequences coming from disparate organisms that may have little to do with each other, especially in the evolutionary manner. For a better insight into conservation of sequence positions in a given set of orthologues, we recommend analyses for coherent small evolutionary-related groups of organisms; it could be more interesting to analyze variations of amino acid positions for mammalian sequences or sequences coming from disparate arthropods, or other closely related sets of organisms. 

Research on the immunophilins started from seminal discoveries linking immunosuppression caused by CsA, FK506 and rapamycin with the capacity of these molecules to form ternary complexes immunosuppressor/immunophilin/(target kinase (TOR) or calcineurin (phosphatase) [1,2,3,4,5]. There are very few examples how cis/trans isomerization of X-Pro bonds influence diverse cellular processes. Cis/trans isomerization of X-Pro epitopes is easy to be made under in vitro conditions using different PPIases and tagged short peptides having X-Pro epitope. Under in vivo conditions we could only assume that PPIases are catalysts for certain X-Pro bonds and by virtue of that they may control some crucial cellular processes. For example, it has been shown that mixed lineage leukemia 1 (MLL1) protein PHD3-bromo cassette connects H3K4me (lysine modification of histone 3) readout to isomerization of Pro residues by CyP33 (PPIE) and histone deacetylase (HDAC)-mediated repression [26,27]. 

The presence of some immunophilins in various cellular compartments [8,28] implies that they are involved in various discrete interaction networks that control many systemic processes. Hypothetical dimerized forms of some immunophilins such as human FKBP36 could have gain analogous function to that of signal transducing adaptor proteins (STAPs) and may be crucial for transcription of certain genes. This distant orthologue of shutdown is encoded on chromosome 6 and is a part of long DNA segment including 11 genes, which is deleted in Williams syndrome [29]. It is involved in chromosomal pairing during meiosis [30] and also interacts with oxysterol-binding protein (OSBP) [31]. FKBP51 could be considered as part of vesicular transport adaptor that shuttle proteins from one cellular localization to another such as from mitochondria to nucleus [28].

Here we briefly discussed several novel issues concerning highly conserved sequence features, some of which may have a crucial impact on the diverse multifaceted activities of PPIases. Our discussion remains as a set of hypotheses, but probably some of them will be verified in the near future. Certainly, for such a massive number of sequences there is an amount of information that remains hidden. Those hidden mysteries would be unraveled if there could be some real progress in research on PPIases and their multiple functions in various organisms.

## Figures and Tables

**Figure A1:**
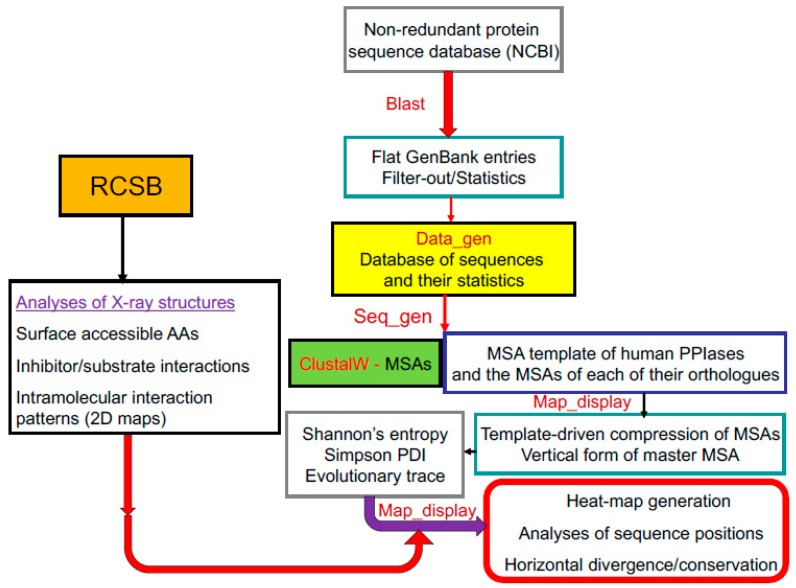
Overall schema of programs used in this note.

## Supplementary Materials

The following are available online at https://www.mdpi.com/2218-273X/9/2/59/s1, see Figure S1A,B have the numbers of the FKBPs and cyclophilins analyzed in this note. Figure S2A displays the source code of Data_gen. Figure S2B has the source code of Seq_gen. Figure S3 has the source code of Map_display. Figure S4 has the heat-map for the FKBPs (Simpson PDIs). Figure S5A contains the MSA of the orthologues of human FKBP36 whereas Figure S5B keeps the names of the aligned sequences. Figure S6 has MSA of FKBD2 orthologous to human FKBP51 (Fkbp5). Figure S7 has the heat-map of the cyclophilins (Simpson PDIs). Figure S8A has the MSA of sequences orthologous to the CLD of human CyP157 whereas Figure S8B keeps the names of the aligned sequences. Figure S9A has the MSA of sequences orthologous to human FKBP38 (Fkbp8) and Figure S9B keeps the names of the aligned sequences. Figure S10–MSA of more refined sequences orthologous to human FKBP36 (Fkbp6). There are also two data-files that contain vertical MSA of the FKBPs and the cyclophilins, which could be useful to confront with the heat-maps.
