# Supplementary material for: Compression of Large Sets of Sequence Data Reveals Fine Diversification of Functional Profiles in Multigene Families of Proteins: A Study for Peptidyl-Prolyl cis/trans Isomerases (PPIase)
Source: Biomolecules. 2019 Feb 11;9(2):59. doi: 10.3390/biom9020059 (PMC6406343; doi:10.3390/biom9020059)
Supplement: Supplementary file 1 [file biomolecules-09-00059-s001.zip › Figure S10 - MSA of FKBP36-second step.pdf]

Checking of entries from the NCBI database & Blast output. Below is the current list of entries of Fkbp6 and their IDs versus human Fkbp6.

NP_003593	2	100	XP_003815624	inactive peptidyl-prolyl cis-trans isomerase FKBP6 isoform X1 [Pan paniscus].
XP_003777068	4	100	XP_003276647	PREDICTED: inactive peptidyl-prolyl cis-trans isomerase FKBP6 [Nomascus leuco
XP_003276647	5	100	XP_003895916	inactive peptidyl-prolyl cis-trans isomerase FKBP6 isoform X1 [Papio anubis].
XP_003895916	6	100	EHH17242	Peptidyl-prolyl cis-trans isomerase FKBP6 [Macaca mulatta].
EHH17242	7	100	XP_001110932	PREDICTED: inactive peptidyl-prolyl cis-trans isomerase FKBP6 isoform X1 [Mac
XP_001110932	8	100	XP_011785750	PREDICTED: inactive peptidyl-prolyl cis-trans isomerase FKBP6 isoform X1 [Col
XP_011785750	9	100	NP_001128683	inactive peptidyl-prolyl cis-trans isomerase FKBP6 isoform b [Homo sapiens].
XP_008979905	15	100	XP_016868230	inactive peptidyl-prolyl cis-trans isomerase FKBP6 isoform X1 [Homo sapiens].
XP_012597698	18	100	XP_011931834	PREDICTED: inactive peptidyl-prolyl cis-trans isomerase FKBP6 isoform X2 [Cer
XP_011931834	19	100	XP_003895917	inactive peptidyl-prolyl cis-trans isomerase FKBP6 isoform X2 [Papio anubis].
XP_020752493	42	100	XP_014988800	PREDICTED: inactive peptidyl-prolyl cis-trans isomerase FKBP6 isoform X2 [Mac
XP_014988800	43	100	XP_011785755	PREDICTED: inactive peptidyl-prolyl cis-trans isomerase FKBP6 isoform X2 [Col
XP_007100027	76	100	XP_008966786	inactive peptidyl-prolyl cis-trans isomerase FKBP6 isoform X2 [Pan paniscus].
XP_006859898	103	100	XP_005549416	PREDICTED: inactive peptidyl-prolyl cis-trans isomerase FKBP6 isoform X5 [Mac
XP_007468849	107	100	XP_014988802	PREDICTED: inactive peptidyl-prolyl cis-trans isomerase FKBP6 isoform X3 [Mac
XP_005344682	120	100	NP_001349718	inactive peptidyl-prolyl cis-trans isomerase FKBP6 isoform d [Homo sapiens].
XP_006971357	125	100	XP_008966787	inactive peptidyl-prolyl cis-trans isomerase FKBP6 isoform X3 [Pan paniscus].
XP_015302699	140	100	XP_011765611	inactive peptidyl-prolyl cis-trans isomerase FKBP6 isoform X7 [Macaca nemestr
XP_012597702	142	100	XP_014988803	PREDICTED: inactive peptidyl-prolyl cis-trans isomerase FKBP6 isoform X4 [Mac
XP_014403986	148	100	XP_016800790	inactive peptidyl-prolyl cis-trans isomerase FKBP6 isoform X3 [Pan troglodyte
XP_007518064	154	100	PNI92595	FKBP6 isoform 5, partial [Pan troglodytes].
XP_021271278	199	100	XP_023074397	inactive peptidyl-prolyl cis-trans isomerase FKBP6 [Piliocolobus tephrosceles
POI24993	203	100	PNI92596	FKBP6 isoform 6 [Pan troglodytes].
XP_003815624	3	99	XP_003777068	inactive peptidyl-prolyl cis-trans isomerase FKBP6 isoform X1 [Pongo abelii].
NP_001128683	10	99	XP_017713171	PREDICTED: inactive peptidyl-prolyl cis-trans isomerase FKBP6 isoform X1 [Rhi
XP_017713171	11	99	XP_010385752	PREDICTED: inactive peptidyl-prolyl cis-trans isomerase FKBP6 isoform X1 [Rhi
XP_010385752	12	99	XP_002817917	inactive peptidyl-prolyl cis-trans isomerase FKBP6 isoform X2 [Pongo abelii].
XP_002817917	13	99	XP_004045599	PREDICTED: inactive peptidyl-prolyl cis-trans isomerase FKBP6 isoform X1 [Gor
XP_003895917	20	99	XP_008016547	PREDICTED: LOW QUALITY PROTEIN: inactive peptidyl-prolyl cis-trans isomerase
XP_022357643	25	99	XP_010385754	PREDICTED: inactive peptidyl-prolyl cis-trans isomerase FKBP6 isoform X3 [Rhi
XP_021563404	129	99	XP_018887307	PREDICTED: inactive peptidyl-prolyl cis-trans isomerase FKBP6 isoform X2 [Gor
XP_004761713	162	99	PNJ85878	FKBP6 isoform 5, partial [Pongo abelii].
XP_010283169	206	99	PNJ85879	FKBP6 isoform 6 [Pongo abelii].
XP_019715044	361	98	XP_018875413	PREDICTED: uncharacterized protein LOC101136274 isoform X1 [Gorilla gorilla g
XP_016868230	16	96	XP_003794239	inactive peptidyl-prolyl cis-trans isomerase FKBP6 isoform X1 [Otolemur garne
XP_003794239	17	95	XP_012597698	inactive peptidyl-prolyl cis-trans isomerase FKBP6 isoform X1 [Microcebus mur
XP_010385754	26	95	XP_017895560	PREDICTED: inactive peptidyl-prolyl cis-trans isomerase FKBP6 isoform X2 [Cap
XP_017895560	27	95	XP_005892900	PREDICTED: inactive peptidyl-prolyl cis-trans isomerase FKBP6 isoform X2 [Bos
XP_005892900	28	95	XP_010852603	PREDICTED: inactive peptidyl-prolyl cis-trans isomerase FKBP6 isoform X2 [Bis
XP_010852603	29	95	XP_546928	inactive peptidyl-prolyl cis-trans isomerase FKBP6 [Canis lupus familiaris].
XP_546928	30	95	XP_005225237	inactive peptidyl-prolyl cis-trans isomerase FKBP6 isoform X1 [Bos taurus].
XP_005225237	31	95	XP_021556246	inactive peptidyl-prolyl cis-trans isomerase FKBP6 isoform X1 [Neomonachus sc
XP_021556246	32	95	XP_011978492	PREDICTED: inactive peptidyl-prolyl cis-trans isomerase FKBP6 isoform X1 [Ovi
XP_011978492	33	95	NP_001296390	inactive peptidyl-prolyl cis-trans isomerase FKBP6 [Equus caballus].
NP_001296390	34	95	XP_011959859	PREDICTED: inactive peptidyl-prolyl cis-trans isomerase FKBP6 isoform X1 [Ovi
XP_011959859	35	95	XP_006942097	inactive peptidyl-prolyl cis-trans isomerase FKBP6 [Felis catus].
XP_019277206	39	95	XP_014705926	PREDICTED: inactive peptidyl-prolyl cis-trans isomerase FKBP6 [Equus asinus].
XP_014705926	40	95	XP_008540619	PREDICTED: inactive peptidyl-prolyl cis-trans isomerase FKBP6 [Equus przewals
XP_008540619	41	95	XP_020752493	inactive peptidyl-prolyl cis-trans isomerase FKBP6 isoform X1 [Odocoileus vir
XP_014652016	52	95	XP_012597701	inactive peptidyl-prolyl cis-trans isomerase FKBP6 isoform X2 [Microcebus mur
XP_004317177	55	95	AMY15662	FK506 binding protein 6 [Bos grunniens].
AMY15662	56	95	XP_005892901	PREDICTED: inactive peptidyl-prolyl cis-trans isomerase FKBP6 isoform X1 [Bos
XP_005892901	57	95	XP_017895559	PREDICTED: inactive peptidyl-prolyl cis-trans isomerase FKBP6 isoform X1 [Cap
XP_005662030	59	95	XP_010852604	PREDICTED: inactive peptidyl-prolyl cis-trans isomerase FKBP6 isoform X3 [Bis
XP_010852604	60	95	XP_010852602	PREDICTED: inactive peptidyl-prolyl cis-trans isomerase FKBP6 isoform X1 [Bis
EPY83956	62	95	NP_001095679	inactive peptidyl-prolyl cis-trans isomerase FKBP6 [Bos taurus].
XP_010970644	65	95	XP_004021020	PREDICTED: inactive peptidyl-prolyl cis-trans isomerase FKBP6 isoform X2 [Ovi
XP_006057167	70	95	XP_019843968	PREDICTED: inactive peptidyl-prolyl cis-trans isomerase FKBP6 [Bos indicus].
XP_006088351	79	95	XP_020752496	inactive peptidyl-prolyl cis-trans isomerase FKBP6 isoform X2 [Odocoileus vir

XP_014411577	93	95	XP_007073943	PREDICTED: inactive peptidyl-prolyl cis-trans isomerase FKBP6 [Panthera tigri
XP_006155025	95	95	XP_005328656	inactive peptidyl-prolyl cis-trans isomerase FKBP6 [Ictidomys tridecemlineatu
XP_011765611	141	95	XP_012597702	inactive peptidyl-prolyl cis-trans isomerase FKBP6 isoform X3 [Microcebus mur
XP_003934167	22	94	XP_012495716	PREDICTED: inactive peptidyl-prolyl cis-trans isomerase FKBP6 isoform X1 [Pro
XP_007100028	37	94	XP_005972253	PREDICTED: peptidyl-prolyl cis-trans isomerase FKBP6 isoform X2 [Pantholops h
XP_005972253	38	94	XP_019277206	PREDICTED: inactive peptidyl-prolyl cis-trans isomerase FKBP6 [Panthera pardu
XP_008979887	46	94	XP_004399150	PREDICTED: inactive peptidyl-prolyl cis-trans isomerase FKBP6 isoform X1 [Odo
XP_007186614	51	94	XP_014652016	PREDICTED: inactive peptidyl-prolyl cis-trans isomerase FKBP6 [Ceratotherium
XP_012597701	53	94	XP_004761711	PREDICTED: inactive peptidyl-prolyl cis-trans isomerase FKBP6 isoform X1 [Mus
XP_017895559	58	94	XP_005662030	inactive peptidyl-prolyl cis-trans isomerase FKBP6 isoform X1 [Sus scrofa].
XP_010342933	69	94	XP_006057167	PREDICTED: peptidyl-prolyl cis-trans isomerase FKBP6 [Bubalus bubalis].
XP_006201442	72	94	XP_005972252	PREDICTED: peptidyl-prolyl cis-trans isomerase FKBP6 isoform X1 [Pantholops h
XP_005344683	101	94	XP_020941978	inactive peptidyl-prolyl cis-trans isomerase FKBP6 isoform X2 [Sus scrofa].
XP_014425414	161	94	XP_004761713	PREDICTED: inactive peptidyl-prolyl cis-trans isomerase FKBP6 isoform X2 [Mus
XP_020488061	346	94	XP_015332442	PREDICTED: inactive peptidyl-prolyl cis-trans isomerase FKBP6, partial [Marmo
XP_004045599	14	93	XP_008979905	PREDICTED: inactive peptidyl-prolyl cis-trans isomerase FKBP6 isoform X2 [Cal
XP_008016547	21	93	XP_003934167	PREDICTED: inactive peptidyl-prolyl cis-trans isomerase FKBP6 isoform X1 [Sai
XP_012495716	23	93	XP_012304459	inactive peptidyl-prolyl cis-trans isomerase FKBP6 isoform X1 [Aotus nancymaa
XP_012304459	24	93	XP_022357643	inactive peptidyl-prolyl cis-trans isomerase FKBP6 isoform X1 [Enhydra lutris
XP_022444356	45	93	XP_008979887	PREDICTED: inactive peptidyl-prolyl cis-trans isomerase FKBP6 isoform X1 [Cal
XP_004268847	48	93	XP_019659608	PREDICTED: inactive peptidyl-prolyl cis-trans isomerase FKBP6 [Ailuropoda mel
XP_024602986	50	93	XP_007186614	PREDICTED: peptidyl-prolyl cis-trans isomerase FKBP6 isoform X1 [Balaenoptera
XP_004021020	66	93	XP_015450070	inactive peptidyl-prolyl cis-trans isomerase FKBP6 [Pteropus alecto].
XP_015450070	67	93	XP_017516100	PREDICTED: inactive peptidyl-prolyl cis-trans isomerase FKBP6 [Manis javanica
XP_017516100	68	93	XP_010342933	PREDICTED: inactive peptidyl-prolyl cis-trans isomerase FKBP6 isoform X2 [Sai
XP_005972252	73	93	XP_004380900	inactive peptidyl-prolyl cis-trans isomerase FKBP6 [Trichechus manatus latiro
XP_004380900	74	93	XP_007468850	PREDICTED: peptidyl-prolyl cis-trans isomerase FKBP6 isoform X2 [Lipotes vexi
XP_008966786	77	93	XP_020017458	inactive peptidyl-prolyl cis-trans isomerase FKBP6 [Castor canadensis].
XP_006773483	82	93	XP_012304461	inactive peptidyl-prolyl cis-trans isomerase FKBP6 isoform X3 [Aotus nancymaa
XP_016054558	85	93	XP_011369980	inactive peptidyl-prolyl cis-trans isomerase FKBP6 [Pteropus vampyrus].
XP_007073943	94	93	XP_006155025	PREDICTED: inactive peptidyl-prolyl cis-trans isomerase FKBP6 isoform X1 [Tup
XP_005549416	104	93	XP_024427494	inactive peptidyl-prolyl cis-trans isomerase FKBP6 [Desmodus rotundus].
XP_016001971	106	93	XP_007468849	PREDICTED: peptidyl-prolyl cis-trans isomerase FKBP6 isoform X1 [Lipotes vexi
XP_004620930	111	93	XP_008696858	PREDICTED: inactive peptidyl-prolyl cis-trans isomerase FKBP6 [Ursus maritimu
NP_001099392	122	93	XP_005396843	PREDICTED: inactive peptidyl-prolyl cis-trans isomerase FKBP6 [Chinchilla lan
XP_023420891	137	93	XP_006155027	PREDICTED: inactive peptidyl-prolyl cis-trans isomerase FKBP6 isoform X2 [Tup
XP_004630630	146	93	XP_004840130	inactive peptidyl-prolyl cis-trans isomerase FKBP6 isoform X1 [Heterocephalus
XP_016800790	149	93	ELK06863	FK506-binding protein 6 [Pteropus alecto].
ELK06863	150	93	EHB08283	FK506-binding protein 6 [Heterocephalus glaber].
EHB08283	151	93	XP_004706046	PREDICTED: inactive peptidyl-prolyl cis-trans isomerase FKBP6 [Echinops telfa
XP_004706046	152	93	XP_021110082	inactive peptidyl-prolyl cis-trans isomerase FKBP6 isoform X2 [Heterocephalus
XP_010306733	231	93	XP_008979919	PREDICTED: inactive peptidyl-prolyl cis-trans isomerase FKBP6 isoform X3 [Cal
XP_019619874	398	93	XP_016801711	inactive peptidyl-prolyl cis-trans isomerase FKBP6-like isoform X3 [Pan trogl
XP_011785755	44	92	XP_022444356	inactive peptidyl-prolyl cis-trans isomerase FKBP6 isoform X2 [Delphinapterus
XP_010852602	61	92	EPY83956	peptidyl-prolyl cis-trans isomerase FKBP6 [Camelus ferus].
XP_008139950	64	92	XP_010970644	PREDICTED: inactive peptidyl-prolyl cis-trans isomerase FKBP6 [Camelus bactri
XP_019843968	71	92	XP_006201442	PREDICTED: inactive peptidyl-prolyl cis-trans isomerase FKBP6 [Vicugna pacos]
XP_020752496	80	92	XP_022444355	inactive peptidyl-prolyl cis-trans isomerase FKBP6 isoform X1 [Delphinapterus
XP_024602987	88	92	XP_019610492	PREDICTED: inactive peptidyl-prolyl cis-trans isomerase FKBP6 [Rhinolophus si
XP_006889852	91	92	XP_007940604	PREDICTED: inactive peptidyl-prolyl cis-trans isomerase FKBP6 [Orycteropus af
XP_007940604	92	92	XP_014411577	PREDICTED: inactive peptidyl-prolyl cis-trans isomerase FKBP6 [Camelus ferus]
XP_010979058	98	92	XP_006249193	PREDICTED: inactive peptidyl-prolyl cis-trans isomerase FKBP6 isoform X2 [Rat
XP_002722023	100	92	XP_005344683	PREDICTED: inactive peptidyl-prolyl cis-trans isomerase FKBP6 isoform X2 [Mic
XP_012584338	109	92	XP_006971358	PREDICTED: inactive peptidyl-prolyl cis-trans isomerase FKBP6 isoform X2 [Per
XP_008696858	112	92	EGW01828	FK506-binding protein 6 [Cricetulus griseus].
XP_021018812	114	92	XP_012782940	PREDICTED: inactive peptidyl-prolyl cis-trans isomerase FKBP6 [Ochotona princ
XP_012782940	115	92	XP_019502388	PREDICTED: inactive peptidyl-prolyl cis-trans isomerase FKBP6 isoform X1 [Hip
XP_019502388	116	92	XP_021042681	inactive peptidyl-prolyl cis-trans isomerase FKBP6 isoform X2 [Mus pahari].
XP_021519217	119	92	XP_005344682	PREDICTED: inactive peptidyl-prolyl cis-trans isomerase FKBP6 isoform X1 [Mic
NP_001349718	121	92	NP_001099392	inactive peptidyl-prolyl cis-trans isomerase FKBP6 [Rattus norvegicus].
XP_005396843	123	92	XP_019502390	PREDICTED: inactive peptidyl-prolyl cis-trans isomerase FKBP6 isoform X2 [Hip

XP_019502390	124	92	XP_006971357	PREDICTED: inactive peptidyl-prolyl cis-trans isomerase FKBP6 isoform X1 [Per
XP_008966787	126	92	XP_003500991	PREDICTED: inactive peptidyl-prolyl cis-trans isomerase FKBP6 [Cricetulus gri
NP_291049	133	92	XP_021042679	inactive peptidyl-prolyl cis-trans isomerase FKBP6 isoform X1 [Mus pahari].
XP_021042679	134	92	XP_010617304	PREDICTED: inactive peptidyl-prolyl cis-trans isomerase FKBP6 isoform X1 [Fuk
XP_014988803	143	92	XP_010617307	PREDICTED: inactive peptidyl-prolyl cis-trans isomerase FKBP6 isoform X2 [Fuk
XP_004666244	145	92	XP_004630630	inactive peptidyl-prolyl cis-trans isomerase FKBP6 [Octodon degus].
XP_006258869	174	92	XP_005344684	PREDICTED: inactive peptidyl-prolyl cis-trans isomerase FKBP6 isoform X3 [Mic
NP_001264822	185	92	XP_021042683	inactive peptidyl-prolyl cis-trans isomerase FKBP6 isoform X4 [Mus pahari].
XP_010211497	209	92	XP_017388864	PREDICTED: inactive peptidyl-prolyl cis-trans isomerase FKBP6 [Cebus capucinu
XP_009901738	224	92	XP_012887159	PREDICTED: inactive peptidyl-prolyl cis-trans isomerase FKBP6 [Dipodomys ordi
XP_006942097	36	91	XP_007100028	inactive peptidyl-prolyl cis-trans isomerase FKBP6 isoform X2 [Physeter catod
XP_004399150	47	91	XP_004268847	PREDICTED: inactive peptidyl-prolyl cis-trans isomerase FKBP6 isoform X1 [Orc
XP_019659608	49	91	XP_024602986	inactive peptidyl-prolyl cis-trans isomerase FKBP6 isoform X1 [Neophocaena as
XP_004761711	54	91	XP_004317177	PREDICTED: inactive peptidyl-prolyl cis-trans isomerase FKBP6 isoform X1 [Tur
XP_007468850	75	91	XP_007100027	inactive peptidyl-prolyl cis-trans isomerase FKBP6 isoform X1 [Physeter catod
XP_020017458	78	91	XP_006088351	inactive peptidyl-prolyl cis-trans isomerase FKBP6 [Myotis lucifugus].
XP_022444355	81	91	XP_006773483	PREDICTED: inactive peptidyl-prolyl cis-trans isomerase FKBP6 [Myotis davidii
XP_012304461	83	91	XP_008591583	PREDICTED: inactive peptidyl-prolyl cis-trans isomerase FKBP6 [Galeopterus va
XP_008591583	84	91	XP_016054558	PREDICTED: inactive peptidyl-prolyl cis-trans isomerase FKBP6 [Miniopterus na
XP_011369980	86	91	XP_004268849	PREDICTED: inactive peptidyl-prolyl cis-trans isomerase FKBP6 isoform X2 [Orc
XP_004268849	87	91	XP_024602987	inactive peptidyl-prolyl cis-trans isomerase FKBP6 isoform X2 [Neophocaena as
XP_019610492	89	91	XP_004317179	PREDICTED: inactive peptidyl-prolyl cis-trans isomerase FKBP6 isoform X2 [Tur
XP_011765609	97	91	XP_010979058	PREDICTED: inactive peptidyl-prolyl cis-trans isomerase FKBP6 [Camelus dromed
XP_006249193	99	91	XP_002722023	PREDICTED: inactive peptidyl-prolyl cis-trans isomerase FKBP6 [Oryctolagus cu
XP_020941978	102	91	XP_006859898	PREDICTED: peptidyl-prolyl cis-trans isomerase FKBP6 [Chrysochloris asiatica]
EGW01828	113	91	XP_021018812	inactive peptidyl-prolyl cis-trans isomerase FKBP6 isoform X2 [Mus caroli].
NP_001264821	118	91	XP_021519217	inactive peptidyl-prolyl cis-trans isomerase FKBP6 isoform X2 [Meriones ungui
XP_018887307	130	91	XP_021018806	inactive peptidyl-prolyl cis-trans isomerase FKBP6 isoform X1 [Mus caroli].
XP_010617304	135	91	XP_021519216	inactive peptidyl-prolyl cis-trans isomerase FKBP6 isoform X1 [Meriones ungui
KYO33272	182	91	XP_021018814	inactive peptidyl-prolyl cis-trans isomerase FKBP6 isoform X3 [Mus caroli].
XP_021042683	186	91	XP_021519218	inactive peptidyl-prolyl cis-trans isomerase FKBP6 isoform X3 [Meriones ungui
NP_001095679	63	90	XP_008139950	PREDICTED: inactive peptidyl-prolyl cis-trans isomerase FKBP6 [Eptesicus fusc
XP_004317179	90	90	XP_006889852	PREDICTED: peptidyl-prolyl cis-trans isomerase FKBP6 [Elephantulus edwardii].
XP_024427494	105	90	XP_016001971	PREDICTED: inactive peptidyl-prolyl cis-trans isomerase FKBP6 [Rousettus aegy
XP_014988802	108	90	XP_012584338	PREDICTED: inactive peptidyl-prolyl cis-trans isomerase FKBP6 [Condylura cris
XP_006971358	110	90	XP_004620930	PREDICTED: inactive peptidyl-prolyl cis-trans isomerase FKBP6 [Sorex araneus]
XP_021042681	117	90	NP_001264821	inactive peptidyl-prolyl cis-trans isomerase FKBP6 isoform 2 [Mus musculus].
XP_003500991	127	90	XP_008846596	PREDICTED: inactive peptidyl-prolyl cis-trans isomerase FKBP6 [Nannospalax ga
XP_021018806	131	90	XP_017176678	PREDICTED: inactive peptidyl-prolyl cis-trans isomerase FKBP6 isoform X2 [Mus
XP_017176678	132	90	NP_291049	inactive peptidyl-prolyl cis-trans isomerase FKBP6 isoform 1 [Mus musculus].
XP_010617307	144	90	XP_004666244	PREDICTED: inactive peptidyl-prolyl cis-trans isomerase FKBP6 [Jaculus jaculu
XP_021018814	183	90	OBS57796	hypothetical protein A6R68_11069 [Neotoma lepida].
OBS57796	184	90	NP_001264822	inactive peptidyl-prolyl cis-trans isomerase FKBP6 isoform 3 [Mus musculus].
XP_021110082	153	89	XP_007518064	PREDICTED: inactive peptidyl-prolyl cis-trans isomerase FKBP6 [Erinaceus euro
XP_008846596	128	87	XP_021563404	inactive peptidyl-prolyl cis-trans isomerase FKBP6 [Carlito syrichta].
PNI92595	155	87	XP_020861399	inactive peptidyl-prolyl cis-trans isomerase FKBP6 isoform X1 [Phascolarctos
XP_001519210	159	87	XP_020861404	inactive peptidyl-prolyl cis-trans isomerase FKBP6 isoform X2 [Phascolarctos
XP_021519216	136	86	XP_023420891	LOW QUALITY PROTEIN: inactive peptidyl-prolyl cis-trans isomerase FKBP6 [Cavi
XP_020861399	156	86	XP_016284889	PREDICTED: inactive peptidyl-prolyl cis-trans isomerase FKBP6 isoform X2 [Mon
XP_016284889	157	86	XP_016284888	PREDICTED: inactive peptidyl-prolyl cis-trans isomerase FKBP6 isoform X1 [Mon
XP_006155027	138	84	XP_011765605	inactive peptidyl-prolyl cis-trans isomerase FKBP6 isoform X1 [Macaca nemestr
XP_011765605	139	84	XP_015302699	PREDICTED: inactive peptidyl-prolyl cis-trans isomerase FKBP6 isoform X1 [Mac
XP_021519218	187	84	XP_024653358	inactive peptidyl-prolyl cis-trans isomerase FKBP6 isoform X3 [Macaca nemestr
XP_009678488	189	84	XP_015302701	PREDICTED: inactive peptidyl-prolyl cis-trans isomerase FKBP6 isoform X2 [Mac
PNJ85878	163	82	XP_007441600	inactive peptidyl-prolyl cis-trans isomerase FKBP6 [Python bivittatus].
XP_005328656	96	78	XP_011765609	inactive peptidyl-prolyl cis-trans isomerase FKBP6 isoform X5 [Macaca nemestr
XP_016284888	158	78	XP_001519210	PREDICTED: inactive peptidyl-prolyl cis-trans isomerase FKBP6 isoform X2 [Orn
XP_007441600	164	78	XP_016080873	PREDICTED: inactive peptidyl-prolyl cis-trans isomerase FKBP6 isoform X1 [Orn
XP_005344684	175	78	XP_015670323	PREDICTED: inactive peptidyl-prolyl cis-trans isomerase FKBP6 [Protobothrops
XP_019401968	168	77	XP_013926437	PREDICTED: inactive peptidyl-prolyl cis-trans isomerase FKBP6 isoform X1 [Tha
XP_019401969	170	77	EMP30541	Peptidyl-prolyl cis-trans isomerase FKBP6, partial [Chelonia mydas].

XP_020662988	177	77	XP_007065519	PREDICTED: peptidyl-prolyl cis-trans isomerase FKBP6 [Chelonia mydas].
XP_003770271	191	77	ETE73718	Peptidyl-prolyl cis-trans isomerase FKBP6, partial [Ophiophagus hannah].
XP_020861404	160	76	XP_014425414	inactive peptidyl-prolyl cis-trans isomerase FKBP6, partial [Pelodiscus sinen
XP_019365238	167	76	XP_019401968	PREDICTED: inactive peptidyl-prolyl cis-trans isomerase FKBP6 isoform X1 [Cro
XP_013926437	169	76	XP_019401969	PREDICTED: inactive peptidyl-prolyl cis-trans isomerase FKBP6 isoform X2 [Cro
EMP30541	171	76	XP_015269029	PREDICTED: inactive peptidyl-prolyl cis-trans isomerase FKBP6 [Gekko japonicu
XP_015670323	176	76	XP_020662988	inactive peptidyl-prolyl cis-trans isomerase FKBP6 isoform X1 [Pogona vittice
XP_007065519	178	76	XP_020662989	inactive peptidyl-prolyl cis-trans isomerase FKBP6 isoform X2 [Pogona vittice
XP_020662989	179	76	XP_020662990	inactive peptidyl-prolyl cis-trans isomerase FKBP6 isoform X3 [Pogona vittice
XP_009486380	229	76	KGL76252	Peptidyl-prolyl cis-trans isomerase FKBP6, partial [Tinamus guttatus].
XP_004840130	147	75	XP_014403986	PREDICTED: inactive peptidyl-prolyl cis-trans isomerase FKBP6 [Myotis brandti
XP_015269029	172	75	XP_014464913	PREDICTED: inactive peptidyl-prolyl cis-trans isomerase FKBP6 isoform X2 [All
XP_014464913	173	75	XP_006258869	PREDICTED: inactive peptidyl-prolyl cis-trans isomerase FKBP6 isoform X1 [All
XP_025051231	181	75	KYO33272	inactive peptidyl-prolyl cis-trans isomerase FKBP6 [Alligator mississippiensi
3B7X_A	369	75	XP_014403985	PREDICTED: inactive peptidyl-prolyl cis-trans isomerase FKBP6-like [Myotis br
XP_016080873	165	74	XP_024066689	inactive peptidyl-prolyl cis-trans isomerase FKBP6 isoform X1 [Terrapene mexi
XP_024066689	166	74	XP_019365238	PREDICTED: inactive peptidyl-prolyl cis-trans isomerase FKBP6, partial [Gavia
XP_020662990	180	74	XP_025051231	inactive peptidyl-prolyl cis-trans isomerase FKBP6, partial [Alligator sinens
KFQ39479	242	72	EOB04024	FK506-binding protein 6, partial [Anas platyrhynchos].
XP_024653358	188	71	XP_009678488	PREDICTED: inactive peptidyl-prolyl cis-trans isomerase FKBP6 [Struthio camel
ETE73718	192	71	NP_989052	inactive peptidyl-prolyl cis-trans isomerase FKBP6 [Xenopus tropicalis].
XP_010584273	196	71	XP_017946629	PREDICTED: inactive peptidyl-prolyl cis-trans isomerase FKBP6 isoform X1 [Xen
XP_017946629	197	71	XP_008109385	PREDICTED: inactive peptidyl-prolyl cis-trans isomerase FKBP6 isoform X1 [Ano
XP_017388864	210	71	XP_008109386	PREDICTED: inactive peptidyl-prolyl cis-trans isomerase FKBP6 isoform X2 [Ano
XP_014342809	212	71	KFV84016	Peptidyl-prolyl cis-trans isomerase FKBP6, partial [Struthio camelus australi
XP_015302701	190	70	XP_003770271	inactive peptidyl-prolyl cis-trans isomerase FKBP6 [Sarcophilus harrisii].
NP_989052	193	70	NP_001085204	FK506 binding protein 6 L homeolog [Xenopus laevis].
XP_015151391	201	70	XP_018100492	PREDICTED: FK506 binding protein 6 L homeolog isoform X1 [Xenopus laevis].
XP_008944700	246	70	KFP93140	Peptidyl-prolyl cis-trans isomerase FKBP6, partial [Haliaeetus albicilla].
KFW03341	265	70	KFV99355	Peptidyl-prolyl cis-trans isomerase FKBP6, partial [Fulmarus glacialis].
XP_009643249	215	69	XP_009928379	PREDICTED: inactive peptidyl-prolyl cis-trans isomerase FKBP6, partial [Halia
XP_009512874	220	69	XP_009586283	PREDICTED: inactive peptidyl-prolyl cis-trans isomerase FKBP6, partial [Fulma
KFZ64979	258	69	KFW73903	Peptidyl-prolyl cis-trans isomerase FKBP6, partial [Phalacrocorax carbo].
XP_019903677	269	69	KFU91562	Peptidyl-prolyl cis-trans isomerase FKBP6, partial [Chaetura pelagica].
XP_021479427	293	69	KFR16943	Peptidyl-prolyl cis-trans isomerase FKBP6, partial [Opisthocomus hoazin].
XP_005449738	336	69	KFV20040	Peptidyl-prolyl cis-trans isomerase FKBP6, partial [Tauraco erythrolophus].
XP_009807780	219	68	XP_009512874	PREDICTED: inactive peptidyl-prolyl cis-trans isomerase FKBP6, partial [Phala
XP_009557002	245	68	XP_008944700	PREDICTED: inactive peptidyl-prolyl cis-trans isomerase FKBP6, partial [Merop
KFP21064	257	68	KFZ64979	Peptidyl-prolyl cis-trans isomerase FKBP6, partial [Podiceps cristatus].
XP_018958370	288	68	KGL96380	Peptidyl-prolyl cis-trans isomerase FKBP6, partial [Charadrius vociferus].
XP_014327714	363	68	XP_009081377	PREDICTED: inactive peptidyl-prolyl cis-trans isomerase FKBP6, partial [Acant
XP_020570386	385	68	KFP89108	Peptidyl-prolyl cis-trans isomerase FKBP6, partial [Acanthisitta chloris].
PNJ85879	207	67	XP_018412624	PREDICTED: inactive peptidyl-prolyl cis-trans isomerase FKBP6, partial [Nanor
XP_008109386	211	67	XP_014342809	PREDICTED: inactive peptidyl-prolyl cis-trans isomerase FKBP6 [Latimeria chal
XP_009321903	218	67	XP_009807780	PREDICTED: inactive peptidyl-prolyl cis-trans isomerase FKBP6, partial [Gavia
KFQ72829	222	67	XP_009698973	PREDICTED: inactive peptidyl-prolyl cis-trans isomerase FKBP6, partial [Caria
XP_009887190	228	67	XP_009486380	PREDICTED: inactive peptidyl-prolyl cis-trans isomerase FKBP6, partial [Pelec
XP_010194269	238	67	XP_009962683	PREDICTED: inactive peptidyl-prolyl cis-trans isomerase FKBP6, partial [Tyto
XP_010143455	251	67	KFQ87275	Peptidyl-prolyl cis-trans isomerase FKBP6, partial [Phoenicopterus ruber rube
XP_009863922	254	67	KFW66808	Peptidyl-prolyl cis-trans isomerase FKBP6, partial [Pygoscelis adeliae].
XP_018591809	256	67	KFP21064	Peptidyl-prolyl cis-trans isomerase FKBP6, partial [Egretta garzetta].
KFW73903	259	67	KFM05579	Peptidyl-prolyl cis-trans isomerase FKBP6, partial [Aptenodytes forsteri].
XP_016346087	263	67	KFV51798	Peptidyl-prolyl cis-trans isomerase FKBP6, partial [Gavia stellata].
AAH54673	286	67	KFP60963	Peptidyl-prolyl cis-trans isomerase FKBP6, partial [Cariama cristata].
XP_016350445	304	67	KFV47875	Peptidyl-prolyl cis-trans isomerase FKBP6, partial [Tyto alba].
XP_021479426	307	67	KFQ96759	Peptidyl-prolyl cis-trans isomerase FKBP6, partial [Nipponia nippon].
XP_005449737	349	67	KFO61461	Peptidyl-prolyl cis-trans isomerase FKBP6, partial [Corvus brachyrhynchos].
NP_001085204	194	66	XP_011592867	PREDICTED: inactive peptidyl-prolyl cis-trans isomerase FKBP6 [Aquila chrysae
XP_011592867	195	66	XP_010584273	PREDICTED: inactive peptidyl-prolyl cis-trans isomerase FKBP6 [Haliaeetus leu
XP_008109385	198	66	XP_021271278	inactive peptidyl-prolyl cis-trans isomerase FKBP6 [Numida meleagris].
XP_009698973	223	66	XP_009901738	PREDICTED: inactive peptidyl-prolyl cis-trans isomerase FKBP6, partial [Picoi

XP_009940812	226	66	XP_002187983	PREDICTED: inactive peptidyl-prolyl cis-trans isomerase FKBP6 [Taeniopygia gu
KGL76252	230	66	XP_010306733	PREDICTED: inactive peptidyl-prolyl cis-trans isomerase FKBP6, partial [Balea
KFP29338	240	66	XP_010147897	PREDICTED: inactive peptidyl-prolyl cis-trans isomerase FKBP6 [Eurypyga helia
XP_021396070	250	66	XP_010143455	PREDICTED: inactive peptidyl-prolyl cis-trans isomerase FKBP6, partial [Bucer
KFM05579	260	66	KFV69563	Peptidyl-prolyl cis-trans isomerase FKBP6, partial [Picoides pubescens].
KFV16869	296	66	KFO13846	Peptidyl-prolyl cis-trans isomerase FKBP6, partial [Balearica regulorum gibbe
XP_005808385	340	66	KFO85715	Peptidyl-prolyl cis-trans isomerase FKBP6, partial [Buceros rhinoceros silves
XP_007561093	353	66	PKU35662	peptidyl-prolyl cis-trans isomerase hypothetical protein [Limosa lapponica ba
XP_010022120	236	65	XP_010083598	PREDICTED: inactive peptidyl-prolyl cis-trans isomerase FKBP6, partial [Ptero
XP_023074397	200	64	XP_015151391	inactive peptidyl-prolyl cis-trans isomerase FKBP6 isoform X1 [Gallus gallus]
XP_018100492	202	64	POI24993	hypothetical protein CIB84_011258 [Bambusicola thoracicus].
PNI92596	204	64	XP_015736491	PREDICTED: inactive peptidyl-prolyl cis-trans isomerase FKBP6 [Coturnix japon
XP_008979919	232	64	XP_009998510	PREDICTED: inactive peptidyl-prolyl cis-trans isomerase FKBP6 [Chaetura pelag
XP_010083598	237	64	XP_010194269	PREDICTED: inactive peptidyl-prolyl cis-trans isomerase FKBP6, partial [Coliu
XP_009962683	239	64	KFP29338	Peptidyl-prolyl cis-trans isomerase FKBP6, partial [Colius striatus].
KFV99355	266	64	OXB56102	hypothetical protein ASZ78_007956 [Callipepla squamata].
XP_009988051	295	64	KFV16869	Peptidyl-prolyl cis-trans isomerase FKBP6, partial [Pterocles gutturalis].
XP_012685286	309	64	KFQ41466	Peptidyl-prolyl cis-trans isomerase FKBP6, partial [Nestor notabilis].
KFQ41466	310	64	XP_024997748	inactive peptidyl-prolyl cis-trans isomerase FKBP6 isoform X2 [Gallus gallus]
XP_010408101	318	64	KFO74526	Peptidyl-prolyl cis-trans isomerase FKBP6, partial [Cuculus canorus].
XP_019947232	328	64	KFP85199	Peptidyl-prolyl cis-trans isomerase FKBP6, partial [Apaloderma vittatum].
XP_009928379	216	63	XP_010124464	PREDICTED: inactive peptidyl-prolyl cis-trans isomerase FKBP6, partial [Chlam
XP_009463122	234	63	XP_007894626	PREDICTED: inactive peptidyl-prolyl cis-trans isomerase FKBP6 [Callorhinchus
XP_007894626	235	63	XP_010022120	PREDICTED: inactive peptidyl-prolyl cis-trans isomerase FKBP6, partial [Nesto
XP_010147897	241	63	KFQ39479	Peptidyl-prolyl cis-trans isomerase FKBP6, partial [Mesitornis unicolor].
KFP93140	247	63	KFP44723	Peptidyl-prolyl cis-trans isomerase FKBP6, partial [Chlamydotis macqueenii].
KFP44723	248	63	XP_017553040	PREDICTED: inactive peptidyl-prolyl cis-trans isomerase FKBP6 isoform X1 [Pyg
XP_010891852	253	63	XP_009863922	PREDICTED: inactive peptidyl-prolyl cis-trans isomerase FKBP6, partial [Apalo
KFV51798	264	63	KFW03341	Peptidyl-prolyl cis-trans isomerase FKBP6, partial [Eurypyga helias].
XP_016393789	272	63	XP_009093115	PREDICTED: inactive peptidyl-prolyl cis-trans isomerase FKBP6 isoform X1 [Ser
XP_020341239	300	63	KQK73326	Peptidyl-prolyl cis-trans isomerase FKBP6 [Amazona aestiva].
XP_008298046	351	63	KFP07119	Peptidyl-prolyl cis-trans isomerase FKBP6, partial [Calypte anna].
AWP19851	356	63	KFW82601	Peptidyl-prolyl cis-trans isomerase FKBP6, partial [Manacus vitellinus].
XP_014403985	370	63	XP_017553083	PREDICTED: inactive peptidyl-prolyl cis-trans isomerase FKBP6 isoform X2 [Pyg
XP_008396658	387	63	XP_018774793	PREDICTED: inactive peptidyl-prolyl cis-trans isomerase FKBP6 isoform X2 [Ser
XP_015736491	205	62	XP_010283169	PREDICTED: inactive peptidyl-prolyl cis-trans isomerase FKBP6, partial [Phaet
XP_013812139	214	62	XP_009643249	PREDICTED: inactive peptidyl-prolyl cis-trans isomerase FKBP6 [Egretta garzet
XP_009586283	221	62	KFQ72829	Peptidyl-prolyl cis-trans isomerase FKBP6, partial [Phaethon lepturus].
XP_012887159	225	62	XP_009940812	PREDICTED: inactive peptidyl-prolyl cis-trans isomerase FKBP6 [Opisthocomus h
EOB04024	243	62	XP_023675251	inactive peptidyl-prolyl cis-trans isomerase FKBP6 [Paramormyrops kingsleyae]
XP_020373313	298	62	XP_006640997	PREDICTED: inactive peptidyl-prolyl cis-trans isomerase FKBP6 [Lepisosteus oc
XP_024997748	311	62	KFQ35533	Peptidyl-prolyl cis-trans isomerase FKBP6, partial [Merops nubicus].
XP_018412624	208	61	XP_010211497	PREDICTED: inactive peptidyl-prolyl cis-trans isomerase FKBP6 [Tinamus guttat
XP_002187983	227	61	XP_009887190	PREDICTED: inactive peptidyl-prolyl cis-trans isomerase FKBP6 [Charadrius voc
XP_017553040	249	61	XP_021396070	inactive peptidyl-prolyl cis-trans isomerase FKBP6 [Lonchura striata domestic
XP_019903678	276	61	XP_014110391	PREDICTED: inactive peptidyl-prolyl cis-trans isomerase FKBP6 [Pseudopodoces
XP_019903676	279	61	XP_015502317	PREDICTED: inactive peptidyl-prolyl cis-trans isomerase FKBP6 isoform X2 [Par
NP_001122145	282	61	OWK52486	Inactive peptidyl-prolyl cis-trans isomerase FKBP6 [Lonchura striata domestic
XP_007245276	291	61	XP_023794776	inactive peptidyl-prolyl cis-trans isomerase FKBP6 isoform X1 [Cyanistes caer
KFO13846	297	61	XP_020373313	inactive peptidyl-prolyl cis-trans isomerase FKBP6 [Rhincodon typus].
XP_023285908	321	61	XP_015502316	PREDICTED: inactive peptidyl-prolyl cis-trans isomerase FKBP6 isoform X1 [Par
XP_018774793	388	61	XP_023794778	inactive peptidyl-prolyl cis-trans isomerase FKBP6 isoform X3 [Cyanistes caer
KFQ96759	308	60	XP_012685286	PREDICTED: inactive peptidyl-prolyl cis-trans isomerase FKBP6 [Clupea harengu
XP_020341240	281	59	NP_001122145	peptidyl-prolyl cis-trans isomerase FKBP6 [Danio rerio].
XP_024287707	285	59	AAH54673	Si:dkey-189h5.5 protein, partial [Danio rerio].
KFR16943	294	58	XP_009988051	PREDICTED: inactive peptidyl-prolyl cis-trans isomerase FKBP6, partial [Taura
KFV84016	213	57	XP_013812139	PREDICTED: inactive peptidyl-prolyl cis-trans isomerase FKBP6 [Apteryx austra
XP_010124464	217	57	XP_009321903	PREDICTED: inactive peptidyl-prolyl cis-trans isomerase FKBP6 [Pygoscelis ade
KFW66808	255	57	XP_018591809	PREDICTED: inactive peptidyl-prolyl cis-trans isomerase FKBP6, partial [Scler
XP_009093115	273	57	XP_023869471	inactive peptidyl-prolyl cis-trans isomerase FKBP6-like isoform X1 [Salvelinu
XP_015502317	280	57	XP_020341240	inactive peptidyl-prolyl cis-trans isomerase FKBP6 isoform X2 [Oncorhynchus k

OWK52486	283	57	XP_013992803	PREDICTED: inactive peptidyl-prolyl cis-trans isomerase FKBP6-like isoform X2
XP_013992803	284	57	XP_024287707	inactive peptidyl-prolyl cis-trans isomerase FKBP6 isoform X1 [Oncorhynchus t
KFP60963	287	57	XP_018958370	PREDICTED: inactive peptidyl-prolyl cis-trans isomerase FKBP6-like [Cyprinus
XP_023794776	292	57	XP_021479427	inactive peptidyl-prolyl cis-trans isomerase FKBP6-like isoform X2 [Oncorhync
XP_006640997	299	57	XP_020341239	inactive peptidyl-prolyl cis-trans isomerase FKBP6 isoform X1 [Oncorhynchus k
XP_008500186	302	57	XP_013992801	PREDICTED: inactive peptidyl-prolyl cis-trans isomerase FKBP6-like isoform X1
XP_022534491	306	57	XP_021479426	inactive peptidyl-prolyl cis-trans isomerase FKBP6-like isoform X1 [Oncorhync
XP_004542108	323	57	XP_023823730	E3 ubiquitin-protein ligase rififylin isoform X2 [Salvelinus alpinus].
XP_022046121	360	57	XP_019715044	PREDICTED: inactive peptidyl-prolyl cis-trans isomerase FKBP6 [Hippocampus co
XP_017553083	371	57	XP_023869472	inactive peptidyl-prolyl cis-trans isomerase FKBP6-like isoform X2 [Salvelinu
XP_023869472	372	57	XP_013992804	PREDICTED: inactive peptidyl-prolyl cis-trans isomerase FKBP6-like isoform X3
XP_020774925	374	57	XP_024287709	inactive peptidyl-prolyl cis-trans isomerase FKBP6 isoform X3 [Oncorhynchus t
XP_024287709	375	57	XP_023869475	inactive peptidyl-prolyl cis-trans isomerase FKBP6-like isoform X4 [Salvelinu
XP_023869475	376	57	XP_013992805	PREDICTED: inactive peptidyl-prolyl cis-trans isomerase FKBP6-like isoform X4
XP_013992805	377	57	XP_023869476	inactive peptidyl-prolyl cis-trans isomerase FKBP6-like isoform X5 [Salvelinu
XP_023869476	378	57	XP_024287708	inactive peptidyl-prolyl cis-trans isomerase FKBP6 isoform X2 [Oncorhynchus t
XP_024287708	379	57	XP_023869474	inactive peptidyl-prolyl cis-trans isomerase FKBP6-like isoform X3 [Salvelinu
XP_023869474	380	57	XP_011490382	inactive peptidyl-prolyl cis-trans isomerase FKBP6 isoform X3 [Oryzias latipe
XP_011490382	381	57	XP_020570387	inactive peptidyl-prolyl cis-trans isomerase FKBP6 isoform X2 [Oryzias latipe
XP_014851597	384	57	XP_020570386	inactive peptidyl-prolyl cis-trans isomerase FKBP6 isoform X1 [Oryzias latipe
KFQ87275	252	56	XP_010891852	PREDICTED: inactive peptidyl-prolyl cis-trans isomerase FKBP6 isoform X2 [Eso
KFV69563	261	56	XP_023823731	inactive peptidyl-prolyl cis-trans isomerase FKBP6 isoform X3 [Salvelinus alp
XP_023823731	262	56	XP_016346087	PREDICTED: inactive peptidyl-prolyl cis-trans isomerase FKBP6-like [Sinocyclo
OXB56102	267	56	XP_018958387	PREDICTED: LOW QUALITY PROTEIN: inactive peptidyl-prolyl cis-trans isomerase
XP_018958387	268	56	XP_019903677	PREDICTED: inactive peptidyl-prolyl cis-trans isomerase FKBP6 isoform X3 [Eso
KFU91562	270	56	XP_017314960	PREDICTED: inactive peptidyl-prolyl cis-trans isomerase FKBP6 isoform X1 [Ict
XP_023869471	274	56	XP_019903679	PREDICTED: inactive peptidyl-prolyl cis-trans isomerase FKBP6 isoform X5 [Eso
XP_019903679	275	56	XP_019903678	PREDICTED: inactive peptidyl-prolyl cis-trans isomerase FKBP6 isoform X4 [Eso
XP_014110391	277	56	XP_017314964	PREDICTED: inactive peptidyl-prolyl cis-trans isomerase FKBP6 isoform X2 [Ict
XP_017314964	278	56	XP_019903676	PREDICTED: inactive peptidyl-prolyl cis-trans isomerase FKBP6 isoform X1 [Eso
KGL96380	289	56	XP_016148031	PREDICTED: inactive peptidyl-prolyl cis-trans isomerase FKBP6-like [Sinocyclo
XP_016148031	290	56	XP_007245276	inactive peptidyl-prolyl cis-trans isomerase FKBP6 isoform X2 [Astyanax mexic
KFV47875	305	56	XP_022534491	inactive peptidyl-prolyl cis-trans isomerase FKBP6 isoform X1 [Astyanax mexic
KFQ35533	312	56	XP_020459257	inactive peptidyl-prolyl cis-trans isomerase FKBP6 isoform X1 [Monopterus alb
XP_023823730	324	56	XP_020459258	inactive peptidyl-prolyl cis-trans isomerase FKBP6 isoform X2 [Monopterus alb
XP_023794777	343	56	XP_015803473	PREDICTED: inactive peptidyl-prolyl cis-trans isomerase FKBP6 [Nothobranchius
KPP68264	345	56	XP_020488061	inactive peptidyl-prolyl cis-trans isomerase FKBP6 [Labrus bergylta].
XP_010793476	355	56	AWP19851	putative inactive peptidyl-prolyl cis-trans isomerase FKBP6 isoform 3 [Scopht
XP_009081377	364	56	AWP19849	putative inactive peptidyl-prolyl cis-trans isomerase FKBP6 [Scophthalmus max
XP_012734526	367	56	AWP19850	putative inactive peptidyl-prolyl cis-trans isomerase FKBP6 isoform 2 [Scopht
XP_017314960	271	55	XP_016393789	PREDICTED: inactive peptidyl-prolyl cis-trans isomerase FKBP6-like [Sinocyclo
XP_020459257	313	55	XP_017264554	inactive peptidyl-prolyl cis-trans isomerase FKBP6 isoform X2 [Kryptolebias m
XP_005725080	317	55	XP_010408101	inactive peptidyl-prolyl cis-trans isomerase FKBP6 isoform X1 [Corvus cornix
XP_020459258	325	55	XP_008634949	PREDICTED: inactive peptidyl-prolyl cis-trans isomerase FKBP6 isoform X1 [Cor
KFO85715	341	55	XP_015236136	PREDICTED: inactive peptidyl-prolyl cis-trans isomerase FKBP6 [Cyprinodon var
XP_023794778	389	55	XP_024147377	inactive peptidyl-prolyl cis-trans isomerase FKBP6 isoform X2 [Oryzias melast
XP_024147377	390	55	XP_024147375	inactive peptidyl-prolyl cis-trans isomerase FKBP6 isoform X1 [Oryzias melast
XP_024147375	391	55	XP_024147378	inactive peptidyl-prolyl cis-trans isomerase FKBP6 isoform X3 [Oryzias melast
XP_008396660	394	55	XP_017593619	PREDICTED: inactive peptidyl-prolyl cis-trans isomerase FKBP6 isoform X2 [Cor
XP_009998510	233	54	XP_009463122	PREDICTED: inactive peptidyl-prolyl cis-trans isomerase FKBP6 [Nipponia nippo
XP_013992801	303	54	XP_016350445	PREDICTED: inactive peptidyl-prolyl cis-trans isomerase FKBP6-like [Sinocyclo
XP_005917667	315	54	XP_022621130	inactive peptidyl-prolyl cis-trans isomerase FKBP6 [Seriola dumerili].
XP_005449739	320	54	XP_023285908	inactive peptidyl-prolyl cis-trans isomerase FKBP6 [Seriola lalandi dorsalis]
PWA27518	330	54	XP_003971664	PREDICTED: inactive peptidyl-prolyl cis-trans isomerase FKBP6 [Takifugu rubri
KFV20040	337	54	XP_018551539	PREDICTED: inactive peptidyl-prolyl cis-trans isomerase FKBP6 [Lates calcarif
XP_015803473	344	54	KPP68264	hypothetical protein Z043_113075 [Scleropages formosus].
XP_023134576	366	54	XP_012734526	inactive peptidyl-prolyl cis-trans isomerase FKBP6 [Fundulus heteroclitus].
KQK73326	301	53	XP_008500186	PREDICTED: inactive peptidyl-prolyl cis-trans isomerase FKBP6 [Calypte anna].
XP_017264554	314	53	XP_005917667	PREDICTED: inactive peptidyl-prolyl cis-trans isomerase FKBP6 isoform X2 [Hap
XP_022621130	316	53	XP_005725080	PREDICTED: inactive peptidyl-prolyl cis-trans isomerase FKBP6 isoform X4 [Pun
XP_008634949	326	53	XP_013883182	PREDICTED: inactive peptidyl-prolyl cis-trans isomerase FKBP6 [Austrofundulus

XP_003971664	331	53	XP_005917666	PREDICTED: inactive peptidyl-prolyl cis-trans isomerase FKBP6 isoform X1 [Hap
XP_005917666	332	53	XP_013764145	PREDICTED: inactive peptidyl-prolyl cis-trans isomerase FKBP6 isoform X3 [Pun
XP_013764145	333	53	XP_013764144	PREDICTED: inactive peptidyl-prolyl cis-trans isomerase FKBP6 isoform X2 [Pun
XP_015236136	342	53	XP_023794777	inactive peptidyl-prolyl cis-trans isomerase FKBP6 isoform X2 [Cyanistes caer
XP_015332442	347	53	XP_013764143	PREDICTED: inactive peptidyl-prolyl cis-trans isomerase FKBP6 isoform X1 [Pun
XP_024147378	392	53	XP_014851599	PREDICTED: inactive peptidyl-prolyl cis-trans isomerase FKBP6 isoform X3 [Poe
XP_008396661	396	53	XP_014851600	PREDICTED: inactive peptidyl-prolyl cis-trans isomerase FKBP6 isoform X4 [Poe
PFX19011	402	53	XP_006793207	PREDICTED: peptidyl-prolyl cis-trans isomerase FKBP6-like [Neolamprologus bri
XP_023675251	244	52	XP_009557002	PREDICTED: inactive peptidyl-prolyl cis-trans isomerase FKBP6 [Cuculus canoru
KFO74526	319	52	XP_005449739	PREDICTED: inactive peptidyl-prolyl cis-trans isomerase FKBP6 isoform X3 [Ore
XP_015502316	322	52	XP_004542108	inactive peptidyl-prolyl cis-trans isomerase FKBP6 isoform X2 [Maylandia zebr
XP_013883182	327	52	XP_019947232	PREDICTED: inactive peptidyl-prolyl cis-trans isomerase FKBP6 [Paralichthys o
CAG01803	335	52	XP_005449738	PREDICTED: inactive peptidyl-prolyl cis-trans isomerase FKBP6 isoform X2 [Ore
XP_018551539	338	52	XP_004542106	inactive peptidyl-prolyl cis-trans isomerase FKBP6 isoform X1 [Maylandia zebr
XP_013764143	348	52	XP_005449737	PREDICTED: inactive peptidyl-prolyl cis-trans isomerase FKBP6 isoform X1 [Ore
KFO61461	350	52	XP_008298046	PREDICTED: inactive peptidyl-prolyl cis-trans isomerase FKBP6 [Stegastes part
PKU35662	354	52	XP_010793476	PREDICTED: inactive peptidyl-prolyl cis-trans isomerase FKBP6 [Notothenia cor
AWP19849	365	52	XP_023134576	inactive peptidyl-prolyl cis-trans isomerase FKBP6 [Amphiprion ocellaris].
XP_014851599	393	52	XP_008396660	PREDICTED: inactive peptidyl-prolyl cis-trans isomerase FKBP6 isoform X4 [Poe
XP_017593619	395	52	XP_008396661	PREDICTED: inactive peptidyl-prolyl cis-trans isomerase FKBP6 isoform X5 [Poe
KFP85199	329	51	PWA27518	hypothetical protein CCH79_00000105, partial [Gambusia affinis].
XP_013764144	334	51	CAG01803	unnamed protein product, partial [Tetraodon nigroviridis].
KFP07119	352	51	XP_007561093	PREDICTED: inactive peptidyl-prolyl cis-trans isomerase FKBP6 isoform X2 [Poe
XP_010739761	359	51	XP_022046121	inactive peptidyl-prolyl cis-trans isomerase FKBP6 [Acanthochromis polyacanth
XP_013992804	373	51	XP_020774925	inactive peptidyl-prolyl cis-trans isomerase FKBP6 [Boleophthalmus pectiniros
XP_008396657	383	51	XP_014851597	PREDICTED: inactive peptidyl-prolyl cis-trans isomerase FKBP6 isoform X1 [Poe
XP_004542106	339	50	XP_005808385	inactive peptidyl-prolyl cis-trans isomerase FKBP6 isoform X2 [Xiphophorus ma
KFW82601	357	50	XP_008396659	PREDICTED: inactive peptidyl-prolyl cis-trans isomerase FKBP6 isoform X3 [Poe
XP_008396659	358	50	XP_010739761	PREDICTED: inactive peptidyl-prolyl cis-trans isomerase FKBP6 [Larimichthys c
XP_018875413	362	50	XP_014327714	inactive peptidyl-prolyl cis-trans isomerase FKBP6 isoform X1 [Xiphophorus ma
XP_020570387	382	50	XP_008396657	PREDICTED: inactive peptidyl-prolyl cis-trans isomerase FKBP6 isoform X1 [Poe
KFP89108	386	50	XP_008396658	PREDICTED: inactive peptidyl-prolyl cis-trans isomerase FKBP6 isoform X2 [Poe
XP_014851600	397	50	XP_019619874	PREDICTED: inactive peptidyl-prolyl cis-trans isomerase FKBP6-like [Branchios
XP_020627099	401	50	PFX19011	Inactive peptidyl-prolyl cis-trans isomerase FKBP6 [Stylophora pistillata].
XP_011402831	404	50	XP_022801008	inactive peptidyl-prolyl cis-trans isomerase FKBP6-like [Stylophora pistillat
EKC39444	426	50	XP_013405452	inactive peptidyl-prolyl cis-trans isomerase FKBP6-like isoform X2 [Lingula a
XP_011148430	428	50	XP_013405451	inactive peptidyl-prolyl cis-trans isomerase FKBP6-like isoform X1 [Lingula a
XP_023297605	533	50	XP_002594856	hypothetical protein BRAFLDRAFT_86023 [Branchiostoma floridae].
XP_023710706	400	49	XP_020627099	inactive peptidyl-prolyl cis-trans isomerase FKBP6-like [Orbicella faveolata]
XP_022254671	407	49	ELT98785	hypothetical protein CAPTEDRAFT_220934 [Capitella teleta].
XP_003700792	451	48	XP_023290805	inactive peptidyl-prolyl cis-trans isomerase FKBP6 isoform X2 [Orussus abieti
XP_023290805	452	48	XP_012270499	inactive peptidyl-prolyl cis-trans isomerase FKBP6 isoform X1 [Orussus abieti
XP_017001929	530	48	XP_009012598	hypothetical protein HELRODRAFT_168497 [Helobdella robusta].
XP_014790271	415	47	XP_001636239	predicted protein [Nematostella vectensis].
KOX75315	440	47	XP_014234164	inactive peptidyl-prolyl cis-trans isomerase FKBP6 [Trichogramma pretiosum].
XP_017148191	537	47	XP_002412109	FKBP-type peptidyl-prolyl cis-trans isomerase, putative, partial [Ixodes scap
ELT98785	408	46	XP_021353947	inactive peptidyl-prolyl cis-trans isomerase FKBP6-like [Mizuhopecten yessoen
XP_022108899	411	46	OWF50262	Peptidyl-prolyl cis-trans isomerase FKBP6 [Mizuhopecten yessoensis].
XP_011415211	424	46	XP_020909274	inactive peptidyl-prolyl cis-trans isomerase FKBP6-like [Exaiptasia pallida].
XP_014247946	470	46	XP_014270488	inactive peptidyl-prolyl cis-trans isomerase FKBP6 [Halyomorpha halys].
PFH33404	484	46	XP_019876143	PREDICTED: inactive peptidyl-prolyl cis-trans isomerase shutdown [Aethina tum
XP_006793207	403	45	XP_011402831	PREDICTED: inactive peptidyl-prolyl cis-trans isomerase FKBP6-like [Amphimedo
XP_002128893	418	45	XP_009051935	hypothetical protein LOTGIDRAFT_114969 [Lottia gigantea].
XP_009051935	419	45	XP_012221039	PREDICTED: inactive peptidyl-prolyl cis-trans isomerase FKBP6 [Linepithema hu
XP_013405451	429	45	PVD31214	hypothetical protein C0Q70_06626 [Pomacea canaliculata].
PIK39047	431	45	XP_022336187	inactive peptidyl-prolyl cis-trans isomerase FKBP6-like [Crassostrea virginic
XP_022336187	432	45	XP_022337811	inactive peptidyl-prolyl cis-trans isomerase FKBP6-like [Crassostrea virginic
XP_014475449	436	45	XP_014279621	inactive peptidyl-prolyl cis-trans isomerase FKBP6-like [Halyomorpha halys].
XP_022193504	438	45	XP_014204130	inactive peptidyl-prolyl cis-trans isomerase FKBP6 [Copidosoma floridanum].
XP_012062619	456	45	XP_012527508	PREDICTED: inactive peptidyl-prolyl cis-trans isomerase FKBP6-like [Monomoriu
XP_015185328	505	45	XP_024867102	inactive peptidyl-prolyl cis-trans isomerase FKBP6 [Temnothorax curvispinosus

XP_002167164	413	44	KOF62423	hypothetical protein OCBIM_22023537mg, partial [Octopus bimaculoides].
XP_015515854	423	44	XP_011415211	PREDICTED: inactive peptidyl-prolyl cis-trans isomerase FKBP6 [Crassostrea gi
XP_020909274	425	44	EKC39444	hypothetical protein CGI_10020047 [Crassostrea gigas].
XP_013405452	427	44	XP_011148430	inactive peptidyl-prolyl cis-trans isomerase FKBP6 [Harpegnathos saltator].
XP_014787871	434	44	EFN78601	FK506-binding protein 6 [Harpegnathos saltator].
AKN09689	455	44	XP_012062619	PREDICTED: inactive peptidyl-prolyl cis-trans isomerase FKBP6 [Atta cephalote
XP_005089179	458	44	XP_003424555	PREDICTED: inactive peptidyl-prolyl cis-trans isomerase FKBP6 [Nasonia vitrip
XP_014247948	466	44	XP_011064230	PREDICTED: inactive peptidyl-prolyl cis-trans isomerase FKBP6-like [Acromyrme
XP_018356457	476	44	XP_011173578	PREDICTED: inactive peptidyl-prolyl cis-trans isomerase FKBP6 [Solenopsis inv
XP_018333359	493	44	EFZ09302	hypothetical protein SINV_13600, partial [Solenopsis invicta].
XP_008560701	509	44	XP_011695993	PREDICTED: inactive peptidyl-prolyl cis-trans isomerase FKBP6-like [Wasmannia
XP_016801711	399	43	XP_023710706	inactive peptidyl-prolyl cis-trans isomerase FKBP6 [Cryptotermes secundus].
XP_022801008	405	43	XP_013786360	inactive peptidyl-prolyl cis-trans isomerase FKBP6-like isoform X2 [Limulus p
XP_013786360	406	43	XP_022254671	inactive peptidyl-prolyl cis-trans isomerase FKBP6-like isoform X1 [Limulus p
XP_009857551	422	43	XP_015515854	PREDICTED: inactive peptidyl-prolyl cis-trans isomerase FKBP6 [Neodiprion lec
XP_015586170	444	43	XP_003486736	inactive peptidyl-prolyl cis-trans isomerase FKBP6 [Bombus impatiens].
XP_019882220	446	43	XP_003402471	inactive peptidyl-prolyl cis-trans isomerase FKBP6 [Bombus terrestris].
XP_012270499	453	43	XP_018309319	PREDICTED: inactive peptidyl-prolyl cis-trans isomerase FKBP6 isoform X1 [Tra
XP_003424555	459	43	XP_018055399	PREDICTED: inactive peptidyl-prolyl cis-trans isomerase FKBP6-like [Atta colo
XP_017877748	461	43	XP_018399012	PREDICTED: inactive peptidyl-prolyl cis-trans isomerase FKBP6 [Cyphomyrmex co
XP_018399012	462	43	KYM99352	Peptidyl-prolyl cis-trans isomerase FKBP6 [Cyphomyrmex costatus].
KYM99352	463	43	XP_018309322	PREDICTED: inactive peptidyl-prolyl cis-trans isomerase FKBP6 isoform X2 [Tra
XP_011647883	465	43	XP_014247948	inactive peptidyl-prolyl cis-trans isomerase FKBP6-like isoform X2 [Cimex lec
XP_002430548	469	43	XP_014247946	inactive peptidyl-prolyl cis-trans isomerase FKBP6-like isoform X1 [Cimex lec
XP_011330216	473	43	EGI60945	FK506-binding protein 6, partial [Acromyrmex echinatior].
EGI60945	474	43	XP_011061927	PREDICTED: inactive peptidyl-prolyl cis-trans isomerase FKBP6-like [Acromyrme
XP_016917008	479	43	XP_018374414	PREDICTED: inactive peptidyl-prolyl cis-trans isomerase FKBP6 [Trachymyrmex c
AET50588	499	43	XP_020893545	inactive peptidyl-prolyl cis-trans isomerase FKBP6-like [Exaiptasia pallida].
XP_023237057	417	42	XP_002128893	PREDICTED: inactive peptidyl-prolyl cis-trans isomerase FKBP6 isoform X1 [Cio
XP_012264923	421	42	XP_009857551	PREDICTED: inactive peptidyl-prolyl cis-trans isomerase FKBP6 isoform X2 [Cio
PVD31214	430	42	PIK39047	putative inactive peptidyl-prolyl cis-trans isomerase FKBP6 [Apostichopus jap
XP_014234164	441	42	KII63942	Inactive peptidyl-prolyl cis-trans isomerase FKBP6 [Thelohanellus kitauei].
XP_012527508	457	42	XP_005089179	PREDICTED: inactive peptidyl-prolyl cis-trans isomerase FKBP6-like [Aplysia c
XP_018309322	464	42	XP_011647883	inactive peptidyl-prolyl cis-trans isomerase FKBP6 [Pogonomyrmex barbatus].
ORY30517	472	42	XP_011330216	PREDICTED: inactive peptidyl-prolyl cis-trans isomerase FKBP6 [Ooceraea biroi
XP_017070534	515	42	XP_001942690	PREDICTED: inactive peptidyl-prolyl cis-trans isomerase FKBP6 [Acyrthosiphon
XP_021353947	409	41	XP_022108900	inactive peptidyl-prolyl cis-trans isomerase FKBP6-like isoform X2 [Acanthast
XP_022108900	410	41	XP_022108899	inactive peptidyl-prolyl cis-trans isomerase FKBP6-like isoform X1 [Acanthast
OWF50262	412	41	XP_002167164	PREDICTED: inactive peptidyl-prolyl cis-trans isomerase FKBP6-like [Hydra vul
XP_001636239	416	41	XP_023237057	inactive peptidyl-prolyl cis-trans isomerase FKBP6-like [Centruroides sculptu
XP_012221039	420	41	XP_012264923	inactive peptidyl-prolyl cis-trans isomerase FKBP6 [Athalia rosae].
XP_014270488	471	41	ORY30517	hypothetical protein BCR33DRAFT_724326 [Rhizoclosmatium globosum].
XP_011061927	475	41	XP_018356457	PREDICTED: inactive peptidyl-prolyl cis-trans isomerase FKBP6-like isoform X2
XP_011173578	477	41	XP_018356456	PREDICTED: inactive peptidyl-prolyl cis-trans isomerase FKBP6-like isoform X1
XP_019876143	485	41	XP_014299498	PREDICTED: inactive peptidyl-prolyl cis-trans isomerase FKBP6-like [Microplit
XP_005186588	489	41	XP_011879049	PREDICTED: inactive peptidyl-prolyl cis-trans isomerase FKBP6 isoform X2 [Vol
XP_013109471	491	41	XP_011879048	PREDICTED: inactive peptidyl-prolyl cis-trans isomerase FKBP6 isoform X1 [Vol
XP_011206843	508	41	XP_008560701	PREDICTED: inactive peptidyl-prolyl cis-trans isomerase FKBP6 isoform X2 [Mic
XP_011695993	510	41	PIK55877	putative peptidyl-prolyl cis-trans isomerase FKBP6-like [Apostichopus japonic
XP_017120033	513	41	XP_022165811	inactive peptidyl-prolyl cis-trans isomerase FKBP6-like [Myzus persicae].
XP_014091483	518	41	XP_015376375	PREDICTED: inactive peptidyl-prolyl cis-trans isomerase FKBP6-like [Diuraphis
XP_015431627	521	41	ORX48915	FKBP-like protein [Piromyces finnis].
EFN78601	435	40	XP_014475449	PREDICTED: inactive peptidyl-prolyl cis-trans isomerase FKBP6 [Dinoponera qua
KII63942	442	40	XP_024936423	inactive peptidyl-prolyl cis-trans isomerase FKBP6 isoform X2 [Cephus cinctus
XP_024936423	443	40	XP_015586170	inactive peptidyl-prolyl cis-trans isomerase FKBP6 isoform X1 [Cephus cinctus
XP_003486736	445	40	XP_019882220	PREDICTED: inactive peptidyl-prolyl cis-trans isomerase FKBP6 isoform X1 [Cam
XP_003402471	447	40	XP_012134979	PREDICTED: inactive peptidyl-prolyl cis-trans isomerase FKBP6 isoform X2 [Meg
XP_017756606	449	40	XP_012134978	PREDICTED: inactive peptidyl-prolyl cis-trans isomerase FKBP6 isoform X1 [Meg
XP_012134978	450	40	XP_003700792	PREDICTED: inactive peptidyl-prolyl cis-trans isomerase FKBP6 isoform X3 [Meg
XP_018055399	460	40	XP_017877748	PREDICTED: inactive peptidyl-prolyl cis-trans isomerase FKBP6 [Ceratina calca
XP_016917007	481	40	XP_019882221	PREDICTED: inactive peptidyl-prolyl cis-trans isomerase shutdown isoform X2 [

KOC63519	487	40	XP_015126020	PREDICTED: inactive peptidyl-prolyl cis-trans isomerase FKBP6 [Diachasma allo
XP_011879048	492	40	XP_018333359	PREDICTED: inactive peptidyl-prolyl cis-trans isomerase FKBP6 [Agrilus planip
XP_018790058	498	40	AET50588	hypothetical protein [Eimeria tenella].
XP_024867102	506	40	XP_011130643	putative peptidyl-prolyl cis-trans isomerase [Gregarina niphandrodes].
XP_001942690	516	40	XP_003883131	Peptidylprolyl isomerase D-related [Neospora caninum Liverpoo
XP_016974015	526	40	KFG33606	peptidyl-prolyl cis-trans isomerase, partial [Toxoplasma gondii FOU].
XP_017045419	528	40	PUA87907	peptidyl-prolyl cis-trans isomerase, partial [Toxoplasma gondii TgCATBr9].
XP_009012598	531	40	XP_008888049	peptidyl-prolyl cis-trans isomerase [Hammondia hammondi].
XP_011298074	540	40	ESS30489	peptidyl-prolyl cis-trans isomerase [Toxoplasma gondii VEG].
ESS30489	541	40	EPR59296	peptidyl-prolyl cis-trans isomerase [Toxoplasma gondii GT1].
EPR59296	542	40	OAJ38225	hypothetical protein BDEG_22175 [Batrachochytrium dendrobatidis JEL423].
OAJ38225	543	40	XP_018637740	peptidyl-prolyl cis-trans isomerase [Toxoplasma gondii ME49].
XP_018637740	544	40	KYK63140	peptidyl-prolyl cis-trans isomerase [Toxoplasma gondii TgCatPRC2].
KYK63140	545	40	CEL73032	TPA: peptidyl-prolyl isomerase, putative [Toxoplasma gondii VEG].
XP_014279621	437	39	XP_022193504	inactive peptidyl-prolyl cis-trans isomerase FKBP6 [Nilaparvata lugens].
XP_014204130	439	39	KOX75315	Peptidyl-prolyl cis-trans isomerase FKBP6 [Melipona quadrifasciata].
XP_011064230	467	39	XP_014613253	PREDICTED: inactive peptidyl-prolyl cis-trans isomerase FKBP6 [Polistes canad
XP_006611831	496	39	XP_014245088	inactive peptidyl-prolyl cis-trans isomerase FKBP6-like [Cimex lectularius].
ORX48915	522	39	XP_001958659	uncharacterized protein Dana_GF12456 [Drosophila ananassae].
XP_001958659	523	39	XP_013077159	PREDICTED: inactive peptidyl-prolyl cis-trans isomerase FKBP6-like isoform X2
XP_013077159	524	39	XP_013077158	PREDICTED: inactive peptidyl-prolyl cis-trans isomerase FKBP6-like isoform X1
XP_022337811	433	38	XP_014787871	PREDICTED: inactive peptidyl-prolyl cis-trans isomerase FKBP6-like, partial [
XP_012134979	448	38	XP_017756606	PREDICTED: inactive peptidyl-prolyl cis-trans isomerase FKBP6 [Eufriesea mexi
XP_014613253	468	38	XP_002430548	FK506-binding protein, putative [Pediculus humanus corporis].
XP_011191641	483	38	PFH33404	peptidyl-prolyl cis-trans isomerase [Besnoitia besnoiti].
XP_014299498	486	38	KOC63519	Peptidyl-prolyl cis-trans isomerase FKBP6 [Habropoda laboriosa].
XP_002092689	501	38	XP_020293583	inactive peptidyl-prolyl cis-trans isomerase FKBP6-like [Pseudomyrmex gracili
XP_003689972	504	38	XP_015185328	PREDICTED: inactive peptidyl-prolyl cis-trans isomerase FKBP6 [Polistes domin
XP_022165811	514	38	XP_017070534	PREDICTED: inactive peptidyl-prolyl cis-trans isomerase shutdown [Drosophila
XP_015376375	519	38	KOF66856	hypothetical protein OCBIM_22011108mg, partial [Octopus bimaculoides].
XP_004517316	536	38	XP_017148191	PREDICTED: inactive peptidyl-prolyl cis-trans isomerase shutdown-like [Drosop
XP_018356456	478	37	XP_016917008	PREDICTED: inactive peptidyl-prolyl cis-trans isomerase FKBP6 isoform X2 [Api
XP_018374414	480	37	XP_016917007	PREDICTED: inactive peptidyl-prolyl cis-trans isomerase FKBP6 isoform X1 [Api
XP_016927793	495	37	XP_006611831	PREDICTED: peptidyl-prolyl cis-trans isomerase FKBP6-like [Apis dorsata].
PIK55877	511	37	XP_022218357	inactive peptidyl-prolyl cis-trans isomerase shutdown [Drosophila obscura].
KOF62423	414	36	XP_014790271	PREDICTED: inactive peptidyl-prolyl cis-trans isomerase FKBP6-like [Octopus b
XP_016952968	503	36	XP_003689972	PREDICTED: inactive peptidyl-prolyl cis-trans isomerase FKBP6 isoform X1 [Api
XP_002594856	534	36	XP_018896035	PREDICTED: inactive peptidyl-prolyl cis-trans isomerase FKBP6-like [Bemisia t
XP_014507068	539	36	XP_011298074	PREDICTED: inactive peptidyl-prolyl cis-trans isomerase shutdown [Fopius aris
NP_611837	549	36	XP_012342118	PREDICTED: inactive peptidyl-prolyl cis-trans isomerase FKBP6 isoform X2 [Api
XP_002040150	552	36	XP_001357527	uncharacterized protein Dpse_GA18393 [Drosophila pseudoobscura pseudoobscura]
XP_001357527	553	36	XP_002015409	GL11067 [Drosophila persimilis].
XP_017469679	555	36	XP_020810045	inactive peptidyl-prolyl cis-trans isomerase shutdown [Drosophila serrata].
KOF66856	520	35	XP_015431627	PREDICTED: inactive peptidyl-prolyl cis-trans isomerase shutdown [Dufourea no
XP_020293583	502	34	XP_016952968	PREDICTED: inactive peptidyl-prolyl cis-trans isomerase shutdown [Drosophila
XP_013077158	525	34	XP_016974015	PREDICTED: inactive peptidyl-prolyl cis-trans isomerase shutdown [Drosophila
EFZ09302	494	33	XP_016927793	PREDICTED: inactive peptidyl-prolyl cis-trans isomerase shutdown [Drosophila
XP_020893545	500	33	XP_002092689	uncharacterized protein Dyak_GE11527 [Drosophila yakuba].
XP_022218357	512	33	XP_017120033	PREDICTED: inactive peptidyl-prolyl cis-trans isomerase shutdown [Drosophila
KFG33606	527	33	XP_017045419	PREDICTED: inactive peptidyl-prolyl cis-trans isomerase shutdown [Drosophila
XP_002412109	538	33	XP_014507068	70 kDa peptidyl-prolyl isomerase [Vigna radiata var. radiata].
AAX51680	547	33	AFJ24780	peptidyl prolyl cis trans isomerase FKBP-2, partial [Schmidtea mediterranea].
AFJ24780	548	33	NP_611837	shutdown [Drosophila melanogaster].
XP_012342118	550	33	XP_002082854	uncharacterized protein Dsimw501_GD25009, isoform A [Drosophila simulans].
XP_002082854	551	33	XP_002040150	GM15507 [Drosophila sechellia].
XP_019882221	482	31	XP_011191641	PREDICTED: inactive peptidyl-prolyl cis-trans isomerase shutdown [Zeugodacus
PUA87907	529	31	XP_017001929	PREDICTED: inactive peptidyl-prolyl cis-trans isomerase shutdown [Drosophila
XP_015126020	488	30	XP_005186588	PREDICTED: inactive peptidyl-prolyl cis-trans isomerase shutdown [Musca domes
XP_011879049	490	30	XP_013109471	PREDICTED: inactive peptidyl-prolyl cis-trans isomerase shutdown [Stomoxys ca
XP_014245088	497	30	XP_018790058	PREDICTED: inactive peptidyl-prolyl cis-trans isomerase shutdown-like [Bactro
XP_011130643	507	30	XP_011206843	PREDICTED: inactive peptidyl-prolyl cis-trans isomerase shutdown [Bactrocera

XP_003883131	517	30	XP_014091483	PREDICTED: inactive peptidyl-prolyl cis-trans isomerase shutdown [Bactrocera
XP_018896035	535	30	XP_004517316	inactive peptidyl-prolyl cis-trans isomerase shutdown [Ceratitis capitata].
XP_002015409	554	29	XP_017469679	PREDICTED: inactive peptidyl-prolyl cis-trans isomerase shutdown-like [Rhagol
XP_008888049	532	28	XP_023297605	inactive peptidyl-prolyl cis-trans isomerase shutdown-like [Lucilia cuprina].
