# Supplementary material for: Compression of Large Sets of Sequence Data Reveals Fine Diversification of Functional Profiles in Multigene Families of Proteins: A Study for Peptidyl-Prolyl cis/trans Isomerases (PPIase)
Source: Biomolecules. 2019 Feb 11;9(2):59. doi: 10.3390/biom9020059 (PMC6406343; doi:10.3390/biom9020059)

Figure S1A. Numbers of FKBPs analyzed in this note that were extracted from Blast searches of the NCBI database with 1000 sequences limit cutoff.

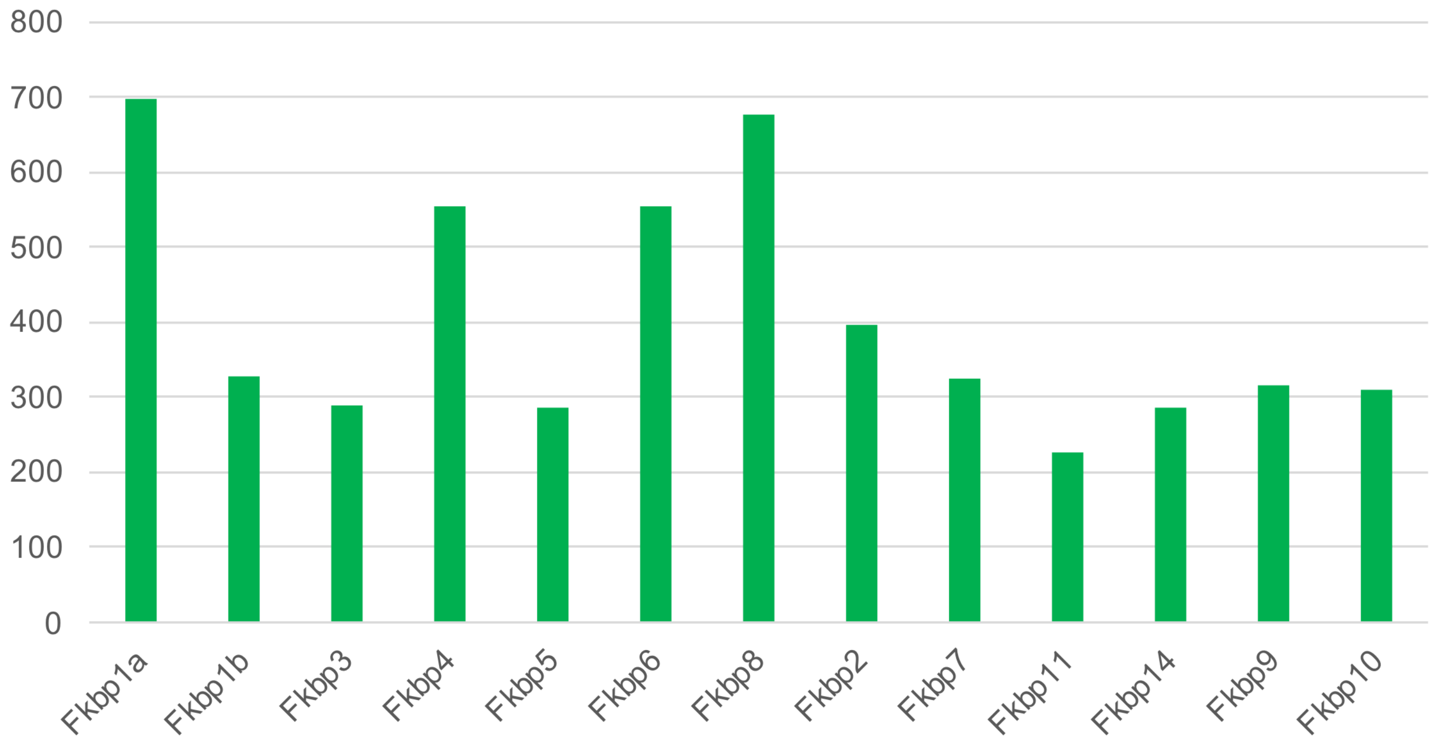

Figure S1B. Numbers of cyclophilins analyzed in this note that were extracted from Blast searches of the NCBI database with 1000 sequences limit cutoff.

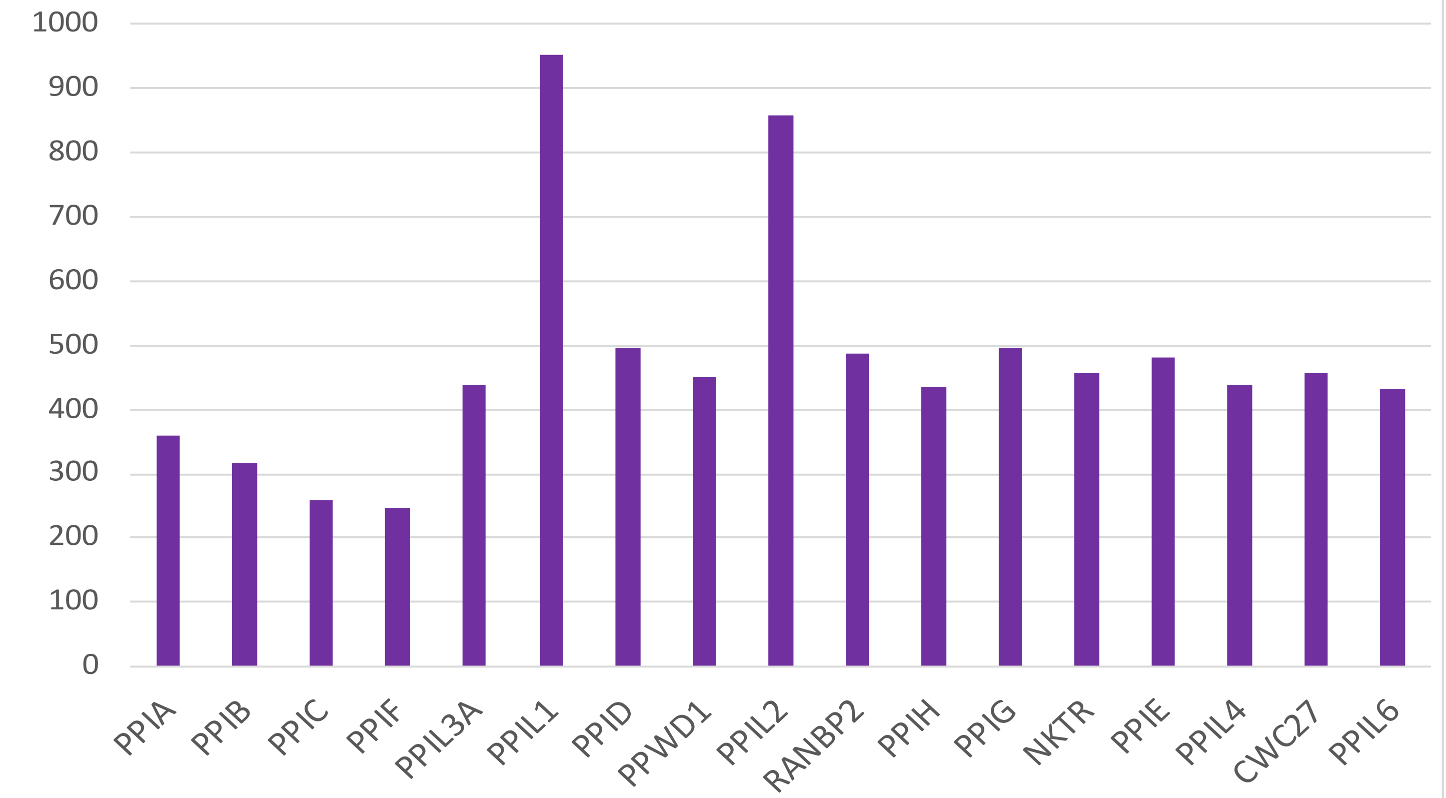

Supplement: Supplementary file 1 [file biomolecules-09-00059-s001.zip › Figure S1A and S1B -the numbers of FKBPs and cyclophilins analyzed..pdf]
