# Supplementary material for: Compression of Large Sets of Sequence Data Reveals Fine Diversification of Functional Profiles in Multigene Families of Proteins: A Study for Peptidyl-Prolyl cis/trans Isomerases (PPIase)
Source: Biomolecules. 2019 Feb 11;9(2):59. doi: 10.3390/biom9020059 (PMC6406343; doi:10.3390/biom9020059)
Supplement: Supplementary file 1 [file biomolecules-09-00059-s001.zip › Figure S2A - source code of Data_gen.pdf]

```
C                                  *
C                                *  *  *
C                              *  *  *  *  *
C            Data_Processing program 'Flat GenBank Format'
C           taken from Blast or Protein search in the NCBI
C++++++++++++++++++++++++++++++++++++++++++++++++++++++++++++++++++++++++++C
C                               *   *   *                                 C
C      Program for reading standard sequence entry Flat GenBank Format    C
C                              *    *    *                                C
C                     Programmed by Andrzej Galat                         C
C                Copyright (c) Andrzej Galat 21/6/2001                    C
C                        *   *   *   *   *   *   *                        C
C      The Data_gen program is made available under the following         C
C      conditions:                                                        C
C     1) The program shall be used for scientific purpose only,           C
C        excluding industrial or commercial purpose.                      C
C     2) Proper acknowledgement shall be made to the author of the        C
C        program in publications resulting from the use of Data_gen       C
C     3) The program shall not be made available to users outside         C
C        the recipient's laboratory, unless written consent is            C
C        obtained.                                                        C
C++++++++++++++++++++++++++++++++++++++++++++++++++++++++++++++++++++++++++C
C     This program is distributed in the hope that it will be useful, but
C     WITHOUT ANY WARRANTY; without even the implied warranty of
C     MERCHANTABILITY or FITNESS FOR A PARTICULAR PURPOSE!
C     -----------------------------------------------------------------
C     Overhauled 11/7/2001 & 25/2/2009 - 25-April-2014
C     |||||||||||||||||||||||||||||||||||||||||||||||||||||||||||||||||
      Program Data_gen
C/////////////////////////////////////////
C     Dimensioned for 10000 Codes/entries in given collection of entries
C     Sequence length = 50,000 AAs in a sequence
      Parameter (Lsq=50000, Mpas=50000)
      Character*1 Seqa(Lsq), AFF, Paxa(20), Paya(20), Chah(9)
      Character*90 Kopa, Fila, Line, Title, Source, Tito(100)
      Character*160 LIPA
      Character*25 Codett(Mpas), Word, Code, Pop, Coda(Mpas)
      Integer Lmaxa(Mpas), Jcant(4)
      Logical Definex, GotIt
      Data Chah/'1','2','3','4','5','6','7','8','9'/
      Data Paxa/'a','c','d','e','f','g','h','i','k','l','m','n',
     "          'p','q','r','s','t','v','w','y'/
      Data Paya/'A','C','D','E','F','G','H','I','K','L','M','N',
     "          'P','Q','R','S','T','V','W','Y'/
C     -----------------------------------------------------------
C     First executional statement
      Definex = .false.
      IPL = 0
      Jcont = 0
      JWRITE = 6
      Do J=1,4
      Jcant(J) = 0
      enddo
C     /////////////////
      Open(Unit=Jwrite,file='nnbb.out',status='unknown')
C     Open the output file (data.seq = library of sequences to hymapi or
datapx)
      Open(Unit=1,file='data.xry',status='unknown')
```

```
      Open(Unit=2,file='data.npp',status='unknown')
      Open(Unit=3,file='data.xpp',status='unknown')
      Open(Unit=4,file='data.div',status='unknown')
      Open(Unit=8,file='data.rem',status='unknown')
      Open(Unit=9,file='data.seq',status='unknown')
      Open(Unit=10,file='data.tit',status='unknown')
      Open(Unit=11,file='data.fas',status='unknown')
      Open(Unit=12,file='data.cod',status='unknown')
      Open(Unit=14,file='data.fun',status='unknown')
      Open(Unit=15,file='data.num',status='unknown')
C=====================================================================C
C          * * * * * * * * the Data_Proc header * * * * * * * * *      C
C=====================================================================C
      WRITE(JWRITE,8000)
 8000 FORMAT(/15X,'Analysis of standard sequence entries'/
     #       12X,'Data_gen program - Version V2.1 - Spring 2001'/)
C
      Jfun = 0
      J1 = 0
      J2 = 0
      J3 = 0
      J4 = 0
C     Above indexes for files
      Open(Unit=5,status='unknown')
      Read(5,10) Fila
      Read(5,10) Word
 10   Format(A)
      Close(5)

      Do J=1,80
      If(Fila(J:J) .ne. ' ') Then
      Ja = J
      Do K=80,1,-1
      If(Fila(K:K) .ne. ' ') Then
      Jb = K
      goto 120
      endif
      Enddo
      Endif
      enddo

 120  Write(Jwrite,12) JA, Jb, Fila(Ja:Jb)
 12   Format(5X,'Reading from datafile || Len =',I2,'-',I2,' || ',2X,A/)
      Write(9,14) Fila
 14   Format('!',1X,'This was created from:',2X,A)
C
      Do J=1,25
      If(Word(J:J) .ne. ' ') Then
      Jc = J
      Do K=25,1,-1
      If(Word(K:K) .ne. ' ') Then
      Jd = K-1
      write(Jwrite,12) Jc, Jd, Word
      goto 140
      endif
      Enddo
      Endif
      enddo
```

```
 140  Write(Jwrite,150) Jd-Jc+1, Word(Jc:Jd+1)
      Write(14,155) Word(Jc:Jd+1)
 155  Format('!',5X,'Word-in-database',1X,A)
 150  Format(5X,'Searching for word-length',1X,I2,1X,A/)
      Jdd = Jd-Jc+1
      Open(Unit=19,file=Fila,status='old')
 100  Read(19,1,Err=990,End=990) Line
      Jcont = Jcont + 1
      If(Line(1:1) .eq. '!') Write(Jwrite,1) Line
      If(Line(1:1) .eq. '!') Write(9,1) Line
      If(Line(1:10) .eq. 'DEFINITION') Then
      IPL = IPL + 1
      LIPA(1:40) = '                                        '
      LIPA(41:80) = '                                        '
      LIPA(81:120) = '                                        '
      LIPA(121:160) = '                                        '
      Do K = 80,1,-1
      La = K
      If(Line(K:K) .ne. ' ') goto 1255
      Enddo
 1255 Kopa = Line(13:La+1)
      LIPA(1:La-12) = Kopa
      Klos = 0
      Do J = 80,1,-1
      If(LIPA(J:J) .ne. ' ') Then
      Klos = J
      goto 1230
      Endif
      Enddo
 1230 Read(19,1,Err=990) Line
      If(Line(1:10) .eq. 'ACCESSION ') goto 1350
      Do KK = 80,1,-1
      Jbb = KK
      If(Line(KK:KK) .ne. ' ') goto 1285
      Enddo
 1285 LIPA = LIPA(1:La-11)//Line(13:Jbb)
      Klos = 0
      Do J = 160,1,-1
      If(LIPA(J:J) .ne. ' ') Then
      Klos = J
      goto 1240
      Endif
      Enddo
 1240 Continue
      GOTO 100
      Endif
C-----
      If(Line(1:6) .eq. 'SOURCE') Then
      Source = Line(1:80)
      Goto 100
      Endif
C
 1350 If(Line(1:10) .eq. 'ACCESSION ') Then
      Do Jp = 13,35
      If(Line(Jp:Jp) .eq. ' ') Then
      Code = Line(13:Jp)//'          '
      goto 160
```

```
      Endif
      Enddo
C-----
 160  Do L=1,9
      If(Code(1:1) .eq. Chah(L)) Then
      J1 = J1 + 1
      Jcant(1) = Jcant(1) + 1
      Write(1,170) J1, IPL, Code(1:12), Title
 170  Format(I4,1X,'(',I4,')',1X,A12,'|| ',A)
      goto 145
      endif
      enddo

      If(Code(1:2) .eq. 'NP') Then
      J2 = J2 + 1
      Write(2,170) J2, IPL, Code(1:12), Title
      Jcant(2) = Jcant(2) + 1
      goto 145
      endif

      If(Code(1:2) .eq. 'XP') Then
      J3 = J3 + 1
      Write(3,170) J3, IPL, Code(1:12), Title
      Jcant(3) = Jcant(3) + 1
      goto 145
      endif

      J4 = J4 + 1
      Write(4,170) J4, IPL, Code(1:12), Title
      Jcant(4) = Jcant(4)+1
 145  Do L = 1,24
      If(Code(L:L) .eq. ' ') then
      Pop = Code(1:L)
      goto 35
      Endif
      Enddo

 35   Code = Pop
      Do Kl = 1,80
      If(Title(Kl:Kl+Jdd-1) .eq. Word(Jc:Jd)) Then
      Jfun = Jfun + 1
      Codett(Jfun) = Code
      Write(Jwrite,220) Jfun, Code(1:12), Title
      Write(14,225) Code(1:15)
 225  Format(1X,A)
 220  Format(I5,1X,A,1X,A)
      goto 280
      endif
      enddo
 280  Definex = .false.
      GOTO 100
      Endif

C----- Reading the sequence records

      IM = 0
      IJJ = 0
      If(Line(1:10) .eq. 'ORIGIN    ') Then
```

```
 200  Read(19,1) Line
      Jcont = Jcont + 1
      If(Line(1:2) .eq. '//') Then
      IJJ = 60
      Goto 300
      Endif

      IL = 0
      DO J=11,75
      If(Line(J:J) .eq. ' ' .and. Line(J+1:J+1) .eq. ' ') goto 300
      If(Line(J:J) .eq. ' ') goto 400
      IL = IL + 1
      Seqa(IL+IM) = Line(J:J)
 400  Continue
      Enddo
C
      IM = IM + 60
      Goto 200
C
 300  IM = IM + IL - IJJ
      Seqa(IM+1) ='*'
      Write(15,*) IM

      If(IPL .gt. 10000) Then
      Write(Jwrite,60) IPL
 60   Format('!!!!!!! The number of entries too big',I6/)
      goto 990
      endif
C     ---------------
      Lmaxa(IPL) = IM
      Coda(IPL) = Code
C
 1    Format(A)
      Write(10,40)
 40   Format(/)
      Write(10,25) IPL, Code, IM
 25   Format(2X,'Entry #',I5,3X,'Code',1X,A,2X,'Naa',I6)
      write(9,20) Code
 20   Format('>',2X,A)
      write(9,21) LIPA(1:Klos)
      Write(10,21) LIPA(1:Klos)
 21   Format('>',2X,A)
      IF(IY .GE. 1) Then
      DO IB = 1,IY
      Write(10,355) Tito(IB)
 355  Format(A)
      Enddo
      Endif
C
      Write(10,355) Source
      Write(8,800) IPL, Code(1:14), IM, LIPA(1:Klos)
 800  Format(I5,1X,A,' || ',I4,1X,A)
      Write(11,350) Code,LIPA(1:Klos)
 350  Format('>',A,12X,A)
      Write(11,4000) (Seqa(I),I=1,IM)
 4000 format(70A1)
      write(12,115) Code
 115  Format(1X,A)
```

```
C-----
      Code = '                          '
      IY = 0
      DO J=1,IM
      AFF = Seqa(J)
      DO K=1,20
      If(AFF .eq. Paxa(K)) Seqa(J) = Paya(K)
      Enddo
      Enddo
      write(9,22) (Seqa(I),I=1,IM+1)
 22   Format(70A1)
      Endif
C-----
      If(Definex .and. Line(1:10) .eq. '            ') Then
      IY = IY + 1
      Tito(IY) = Line(13:77)
      elseif(Definex .and. Line(1:10) .ne. '            ') then
      Definex = .false.
      Endif
      GotIt = .false.
      goto 100

C     Close down all the activities

 990  Write(12,1550)
 1550 Format('CONSTR')
      Lpm = 1
      Do J=1,IPL
      write(12,1450) Coda(J), Lpm, Lmaxa(J)
      enddo
 1450 format(A,I3,5X,I6)
      Write(Jwrite,30) IPL
 30   Format(/10X,'Read-in sequence-entries ||',I5,' ||'/)
      Write(Jwrite,34)
C       10        20        30        40        50        60        70
C1234567890123456789012345678901234567890123456789012345678901234567890
 34   Format(4X,'X-ray code',2X,'NP_xxxx code',2X,'XP_xxxx code',2X,
     #   'Other_types')
      Write(Jwrite,36) (Jcant(J),J=1,4)
 36   Format(1X,4('|| ',I7,3X))
      Write(14,1550)
      Mm = 1
      Do J=1,Jfun
      Code = Codett(J)
      Do K = 1,IPL
      If(Code .eq. Coda(K)) Then
      Lend = Lmaxa(K)
      goto 49
      Endif
      Enddo
 49   Write(14,44) Code(1:15), Mm, Lend
      Enddo
 44   format(A,2I6)
      WRITE(JWRITE,1999) Jcont
 1999 FORMAT(/12X,'Datafile contains ',I7,' line-entries'/
     #        12X,'-> Data_gen ','( Job finished )'/)
C     Close files
      Close(6)
```

```
Close(1)
Close(2)
Close(3)
Close(4)
Close(11)
Close(12)
Close(9)
Close(10)
END
```
