# Supplementary material for: Compression of Large Sets of Sequence Data Reveals Fine Diversification of Functional Profiles in Multigene Families of Proteins: A Study for Peptidyl-Prolyl cis/trans Isomerases (PPIase)
Source: Biomolecules. 2019 Feb 11;9(2):59. doi: 10.3390/biom9020059 (PMC6406343; doi:10.3390/biom9020059)
Supplement: Supplementary file 1 [file biomolecules-09-00059-s001.zip › Figure S2B - source code of Seq_gen.pdf]

```
C                                   *
C                                  * *
C                                   *
C            T h e ><><>< Seq_Gen * * * P R O G R A M (2010)
C
C            This is a Mac version of Datapx.f October-2007
C                Overhaul Mars-22-2010 called Seq_Gen.f
C     |||||||||||||||||||||||||||||||||||||||||||||||||||||||||||||||||
C++++++++++++++++++++++++++++++++++++++++++++++++++++++++++++++++++++++++++C
C                               *  *  *                                   C
C      Program for reading standard sequence entry produced in the NCBI   C
C                              *   *   *                                  C
C                     Programmed by Andrzej Galat                         C
C                Copyright (c) Andrzej Galat 28/2/2002                    C
C                       *   *   *   *   *   *   *                         C
C      The Seq_Gen.f program is made available under the following        C
C      conditions and the GNU licence:                                    C
C     1) The program shall be used for scientific purpose only,           C
C        excluding industrial or commercial purpose.                      C
C     2) Proper acknowledgement shall be made to the author of the        C
C        program in publications resulting from the use of Seq_Gen        C
C     3) The program shall not be made available to users outside         C
C        the recipient's laboratory, unless written consent is            C
C        obtained.                                                        C
C++++++++++++++++++++++++++++++++++++++++++++++++++++++++++++++++++++++++++C
C     This program is distributed in the hope that it will be useful, but
C     WITHOUT ANY WARRANTY; without even the implied warranty of
C     MERCHANTABILITY or FITNESS FOR A PARTICULAR PURPOSE!
C     ----------------------------------------------------------------------
C     Modified 25/3/2010 ++++++ 10/3/2011 + 23-3-2015
C
C     |||||||||||||||||||||||||||||||||||||||||||||||||||||||||||||||||
C
C+++++++++++++++++++++++++++++++++++++++++++++++++++++++++++++++++++++++++++C
C     Subroutines called                                                   C
C     PROTDB - Read and manage protein data base                           C
C     DEDUCT - Read input file and set up the sequence trimming indexes    C
C---------------------------------------------------------------------------C
C     Version (1.21) 4/8/98 *** overhauled 4/01/2001 - Version 2005
      Program Seq_Gen
C     Dimensioned for 1000 sequences and 2500 codes
      PARAMETER (IPA=1000, JDD=1500)
      CHARACTER Decca*1, LINE*80, Ti*80, DATBAS*80
      Character*6 CONSTER(20)
      Integer Jbondo(20)
      Character*25 Codda(IPA), DECOD, CODEX, Work
      DIMENSION WMM(IPA), CODEX(IPA), IS(IPA), IE(IPA), DECOD(JDD)
C     //////////////////////////
C     First executabel statments
C     --------------------------
      Jwrite=2
C     Open general dump file
      Open(Unit=Jwrite,File='apex.out',Status='Unknown')
C=====================================================================C
C         * * write the Datapx header and time of execution * *       C
C=====================================================================C
      write(Jwrite,8000)
 8000 FORMAT(/16X,'Seq_Gen program - Version V2.1 - Spring 2002'/
```

```
     %        17X,'Programmed by A. Galat - Protein Profile'/)
c     WRITE(JWRITE,8010) ACDATE,ACTIME
c 8010 FORMAT(12X,'Run on:',2X,A,2X,'*+*  Started at:',2X,A/)
      KREAD  = 1
      NEX    = 0
C     ///////////////////////////////
C     Open a series of specific files
      Open(Unit=9,File='apex.num',Status='Unknown')
C     apex.dat contains sequences in the FASTA format (to Clustal)
      Open(Unit=14,File='apex.dat',Status='Unknown')
      Open(Unit=10,File='apex.dot',Status='Unknown')
      Open(Unit=11,File='apex.cod',Status='Unknown')
      Open(Unit=20,File='apex.nam',Status='Unknown')
      Open(Unit=27,File='apex.tor',Status='Unknown')
C     apex.aac contains AACs for each entry from apex.dat
      Open(Unit=17,File='apex.aac',status='unknown')
      Open(Unit=28,File='apex.ppm',status='unknown')
      Open(Unit=16,File='apex.ppp',status='unknown')
      Open(Unit=19,File='apex.com',status='unknown')
      Open(Unit=25,File='apex.ppa',status='unknown')
      Open(Unit=24,File='apex.org',status='unknown')
      OPEN(UNIT=18,FILE='apex.dbb',status='unknown')
C     Duplicate-regularization of the input file
      Open(Unit=3,File='apex.rep',status='unknown')
C     File with tabulated data according to certain format
      Open(Unit=33,File='apex.tab',status='unknown')
C|||||||||||||||||||||||||||||||||||||||||||||||||||||||||||||||||||
C     Open main-task file (UNIT=1), status = OLD, READONLY

      READ(KREAD,2) Ti(1:78)
      write(Jwrite,10) Ti(1:78)
      write(3,10) Ti(1:78)
      Write(28,10) Ti(1:77)
      Write(11,10) Ti(1:77)
      Write(10,10) Ti(1:77)
      Write(16,10) Ti(1:77)
      Write(19,10) Ti(1:77)
      Write(25,10) Ti(1:77)
C|||||||||||||||||||||||||||||||||||||||||||||||||||||||||||||||||||
C     Creation of possible database (for further elaboration by the user)
C
      Write(18,10) Ti(1:77)
      WRITE(19,12)
 12   FORMAT(3X,'Comments and structure of the input file'/)
 10   FORMAT(3X,A)
 2    FORMAT(1X,A)
      READ(KREAD,2) DATBAS
      write(3,2) DATBAS
      Read(KREAD,*) Jf
C     Decca is one-character entry (blank .OR. P)
C     If it is P full name of organisms is written in
      Read(KREAD,15) Decca, Jdiva
 15   Format(A1,I10)
      Write(Jwrite,150) Decca, Jdiva
 150  Format(10X,'|||| Chosen-option ','( ',A1,' )',' Cases ',I3/)
      If(Jdiva .gt. 0) Then
      Do K=1,Jdiva
      Read(KREAD,60) CONSTER(K), Jbondo(K)
```

```
 60   Format(A6,1X,I5)
      Enddo
      Do J = 1,Jdiva
      If(J .eq. 1) write(Jwrite,70) CONSTER(J), Jbondo(J), Jbondo(J)
      If(J .gt. 1) write(Jwrite,70) CONSTER(J), Jbondo(J), Jbondo(J)-
     #  (Jbondo(J-1) + 1)
 70   Format(5X,'Constraints-names >  ',A6,2X,'Ends at: ',I3,5X,
     #       '< Members count > ',I3)
      enddo
      Endif
C     ================================
C     Jf - length of the sliding frame
      If(Jf .lt. 5 .OR. Jf .gt. 35) Then
      Write(Jwrite,8100) Jf
 8100 format(//'S T O P !!! * Sliding frame out of range!!!'/)
      goto 1000
      endif
      Write(Jwrite,8020) Jf
 8020 Format(/6X,'Sliding frame =',I5/)
C     NCODE - number of chosen codes
      NCODE = 0

C|||||||||||||||||||||||||||||||||||||||||||||||||||||||||||||||||

 400  READ(KREAD,5,ERR=500) LINE
 5    FORMAT(A)
      IF(LINE(1:1) .EQ. '!'.OR.LINE(1:1) .eq. '>') WRITE(19,2) LINE
      IF(LINE(1:6) .EQ. 'CONSTR') GOTO 500
      IF(LINE(1:1) .EQ. ' ') THEN
      NCODE = NCODE + 1
      CODEX(NCODE) = LINE(2:26)
      ENDIF
      GOTO 400
C     Make sure that some codes are not repeated
 500  NN = 0
      Do 440 J = 1,NCODE
      Work = CODEX(J)
      Jot = J
      Do K = Jot+1,NCODE
      If(Work .EQ. CODEX(K)) Then
      Write(6,670) Work
 670  Format(6X,'Attention repeated code :',A)
      goto 440
      Endif
      ENDDO
      NN = NN + 1
      Codda(NN) = Work
 440  Continue
      Write(Jwrite,510) NCODE, NN
 510  Format(6X,'Attention =',I5,' codes and ',I4,' are unique'/)
      Do J = 1,NCODE
      CODEX(J) = '                         '
      Enddo
      NCODE = NN
      Do J=1,NCODE
      CODEX(J) = Codda(J)
      Write(3,33) CODEX(J)
 33   Format(1X,A)
```

```
      Enddo
C     ////////////////////////////////////////////
C     Read bundries for some sequences - if used
      NEX = NEX + 1
      Work = CODEX(1)
      Ncc = 1
 300  Do K = 1+Ncc,NCODE
      If(CODEX(K) .eq. Work) Write(6,200) K, Work
 200  format('At step',I5,2X,'repeated code',2X,A)
      Enddo
      Ncc = Ncc + 1
      Work = CODEX(Ncc)
      write(66,44) Ncc, Work
 44   format('555',I10,2X,A)
C     !!!!!!!!!!!!!!!!!!!!!!!!!!!
      If(Ncc .lt. NCODE) goto 300
C
      write(6,780) NCODE
 780  format('NCODE====',I6)
      DO J=1,NCODE
      write(11,17) J, CODEX(J)
 17   Format(I3,1X,A)
      Enddo
      If(NEX .GT. IPA) Write(Jwrite,215)
 215   Format(/10X,'Warning: There are more than 1000 codes used'/)
      CALL DEDUCT(KREAD,Isi,NCODE,IPA,NEX,CODEX,DECOD,IS,IE)
      If(Isi .eq. 1) goto 1000
      NEX = NEX - 1
      write(6,55) NEX
 55   format('Constr',I6)
C     Read protein data-bases
      do j=1,NEX
      write(36,80) IS(j), IE(j), DECOD(j)
 80   format('star',2I10,3X,A)
      enddo
C|||||||||||||||||||||||||||||||||||||||||||||||||||||||||||||||||
 600  CALL PROTDB(Jwrite,NCODE,CODEX,DATBAS,NEX,IS,IE,DECOD,WMM,Jf,
     # Jdiva,Decca,CONSTER,Jbondo)
C
 1000 write(Jwrite,1999)
 1999 FORMAT(/5X,'-> Datapx:  ','((( Job finished )))'/)
      CLOSE(KREAD)
      CLOSE(Jwrite)
      CLOSE(9)
      Close(10)
      Close(11)
      Close(14)
      Close(15)
      Close(33)
      Write(18,11)
 11   Format('ENDENDENDENDENDENDENDENDENDENDENDENDENDENDENDENDEND')
      Close(18)
      STOP
      END
C==================================================================C
C     Read protein data base and create the ordered data base      C
C       according to the structure given in the input file         C
C           Author: A. Galat, Version 1.0 * Dec 1990               C
```

```
C     Subroutines called:                                         C
C     HYDROP - hydrophobicity plot and HI calculation             C
C     MASSPR - nominal mass of protein                            C
C     TRANSS - transformation of sequence into integers           C
C     PICAL - pI calculation (see called routines)                C
C===========================================================================C
      SUBROUTINE PROTDB(Jw,NCODE,VCODE,DATBAS,NX,IS,IE,DECOD,WMM,Jsf,
     #  Jdiva,Decca,CONSTER,Jbondo)
C     ||||||||||||||||||||||||||||||||||||||||||||||||||||||||||||||||
      PARAMETER (NSQ=38000)
C     Dimensioned for Ixf = 1000 sequences for being aligned
C     Dimensioned for Itj = 4500 AA-long chain used in the alignment
C     and the length of input sequence cannot be longer than NSQ
      Parameter (Ixf=1000,Itj=4500)
C|||||||||||||||||||||||||||||||||||||||||||||||||||||||||||||||||||
      INTEGER Ltot(Ixf), ISEQ(NSQ), JKL(Ixf), IS(NX), IE(NX)
      CHARACTER*1 TER, SER, QSEQ(NSQ), TSEQ(NSQ)
      CHARACTER*25 Negat(700), Codmax(6), Codx(700), CODET
      Character*50 Tutu
      Character*25 Gigat(700), Mamez, Namex, Curttt, LINEF, VCODE, DECOD
      CHARACTER*100 Curyyy, LINEMX, CURTIT, PROTCO
      CHARACTER*80 TITLE(Ixf), DATBAS
      CHARACTER*1 Decca, Unders, ARRAY(Itj,Ixf)
      Character*6 CONSTER(20)
      Character*15 Lott(Ixf)
      Integer Lat(Ixf), Lyt(Ixf), Jbondo(20)
      DIMENSION PIST(Ixf),HIST(Ixf)
      Dimension VCODE(NCODE),DECOD(1500),CODET(Ixf)
      Real Hff, PII(Ixf), HII(Ixf), WMM(Ixf)
      Character*3 Rota(11)
C234567890123456789012345678901234567890123456789012345678901234567890
      Data Rota/'#Or','#DO','#St','#Re','#CL','#SA','#BP','#BM','#CP',
     #          '#EN','EoR'/
      Logical Trimmo
C     ////////////////////////
C     First executable statment
C     =========================
      Jwrite = Jw
      Jcod = 0
      Lmax = 0
      Lmin = 100000

      If(NCODE .gt. 1000) Then
      Write(2,6000)
 6000 Format(10X,'Too large number of sequences for alignment'/
     "       10X,'Change dimensions of some matrices !!!!!!!!'/)
      Goto 9999
      Endif
      Trimmo = .false.
      PIa = 0.0
      HIa = 0.0
      Pim = 0.0
      Pam = 100.0
      Him = 0.0
      Ham = 100.0
      LREAD = 8
      JRR = 0
      Neg = 0
```

```
C     ||||||||||||||||||||||||||||||||||||||||||||||||||||||||||
C     Pre-set three output matrices according to dimension NCODE
      DO J=1,NCODE
      TITLE(J) = '                                             '
      CODET(J) = '                         '
      JKL(J)   = 1
      PII(J) = 0.0
      HII(J) = 0.0
      WMM(J) = 0.0
      Lat(J) = 0
      Lyt(J) = 0
      Lott(J) = '               '
      DO I = 1,2
      ARRAY(I,J) = ' '
      ENDDO
      ENDDO

      Jhot = 0
      Do K=1,Ncode
      Gigat(K) = '                         '
      Enddo
      write(Jwrite,33)  NCODE
      write(6,33) NCODE
 33   FORMAT(5X,' Number of used codes ',I5/)
      write(Jwrite,34) DATBAS
 34   FORMAT(5X,' Database used ',2X,A/)

C||||||||||||||||||||||||||||||||||||||||||||||||||||||||||||||
C     Open current data-base

      OPEN(UNIT=LREAD,NAME=DATBAS,STATUS='OLD',FORM='FORMATTED')
C     Read two records (Code and title) or comments
 3    FORMAT(A)
 1    READ(LREAD,3,ERR=100) LINEMX
      IF(LINEMX .EQ. '!') GOTO 1
      DO J=1,NCODE
      KAPUT = J
      IF(LINEMX(4:24) .EQ. VCODE(J)) GOTO 500
      ENDDO
      GOTO 1
 500  CONTINUE
      KO     = 0
      PROTCO = LINEMX(1:80)
      READ(LREAD,3,ERR=100) LINEMX
      CURTIT = LINEMX(1:80)
C     Read sequence (multiple records)
  5   READ(LREAD,3,ERR=100) LINEMX
      DO J = 70,1,-1
      TER = LINEMX(J:J)
      IF(TER .NE. ' ') THEN
      KL = J
      GOTO 9
      ENDIF
      ENDDO
C     Set-up the sequence vector
 9    DO K        = 1,KL
      TER         = LINEMX(K:K)
      SER         = TER
```

```
      TSEQ(K+KO) = TER
      ENDDO
      KO = KO + KL
C     If end-sequence record was reached
      IF(SER .EQ. '*' .OR. SER .EQ. '/') GOTO 12
C     Read next sequence record
      GOTO 5
 12   KOUNT  = KO - 1
      write(36,551) KOUNT, PROTCO
 551  format('>>>>>>>>>>>>',I10,2X,A)
      IP = 1
      IH = KOUNT
      Do Kj = 1,KOUNT
C     ||||||||||||||||||||||||||||||||
C     Catch all sequences with X-entry

      If(TSEQ(Kj) .eq. 'X') Then
      Jcod = Jcod + 1
      Codx(Jcod) = PROTCO(3:27)
      Endif
      QSEQ(Kj) = TSEQ(Kj)
      Enddo

C     Calculate parameters of total sequence
C||||||||||||||||||||||||||||||||||||||||||||

      Ltot(KAPUT) = KOUNT
      Call PICAL(IH,PI,QSEQ)
      PII(KAPUT) = PI
      Call TRANSS(IH,ISEQ,QSEQ)
      Call HYDROP(IH,ISEQ,Hff,0,Jsf)
      HII(KAPUT) = Hff
      Call MASSPR(IH,ISEQ,WM,0)
      WMM(KAPUT) = WM
C     Store the captured sequence information
      TITLE(KAPUT) = CURTIT
      CODET(KAPUT) = PROTCO(1:25)
C||||||||||||||||||||||||||||||||||||||||||||
C     If trimming of sequence was requested

      DO J=1,NX
      IF(PROTCO(4:28) .EQ. DECOD(J)) THEN
      Trimmo = .true.
      IP = IS(J)
      IH = IE(J)
      LINEF = DECOD(J)
      WRITE(19,90) LINEF(1:10), KOUNT, IP, IH
      WRITE(36,90) LINEF(1:10), KOUNT, IP, IH
 90   FORMAT(1X,'Sequence encoded',1X,A,1X,'length',I6,
     "       2X,'trimmed',I6,' - ',I7/)
      Lott(KAPUT) = LINEF(1:15)
      Lat(KAPUT) = IP
      Lyt(KAPUT) = IH
      ENDIF
      ENDDO
C     If No trimming write apex.num
      If(.NOT.Trimmo) Then
      Lott(KAPUT) = PROTCO(4:18)
```

```
      Lat(KAPUT) = IP
      Lyt(KAPUT) = IH
      Endif

C||||||||||||||||||||||||||||
C     Write temporary file #80

      JRR = JRR + 1
      write(80,*) KAPUT, IH-IP+1
      write(80,80) (TSEQ(J),J=IP,IH)
 80   format(70A1)
      Trimmo = .false.
      GOTO 1
 100  REWIND LREAD
      CLOSE(LREAD)
      REWIND(80)
C     |||||||||||||||||||||||
C|||||||||||||||||||
      Do L=1,JRR
      Read(80,*) L1, L2
      JKL(L1) = L2
      READ(80,80) (TSEQ(N),N=1,L2)
      Do M=1,L2
      ARRAY(M,L1) = TSEQ(M)
      ENDDO
      ENDDO
      KM = 1
C
C     Final arrangement of sequences and their parameters
C     This is the final looping through the storage area with KAPUT
C
 300  Curttt = CODET(KM)
      IG = JKL(KM)
c      write(6,633) KM, IG

 633  format(2I6)
      DO Jk = 1,IG
      TSEQ(Jk) = ARRAY(Jk,KM)
      Enddo

C     ::::::::::::::::::::::

      Call PICAL(IG,PI,TSEQ)
c      write(6,634) IG,PI
 634  format(I7,2X,F10.5)
      Call TRANSS(IG,ISEQ,TSEQ)
      Call HYDROP(IG,ISEQ,HI,1,Jsf)
      Call MASSPR(IG,ISEQ,WM,1)

C     !!!!!!!!!!!!!!!!!!!!!!!!!

      PIa = PIa + PI
      HIa = HIa + HI

      If(Lmax .lt. IG) Then
      Lmax = IG
      Codmax(1) = CODET(KM)
      Endif
```

```
C     Catch all negative-length entries after Corrto5

      If(IG .lt. 0) Then
      Neg = Neg + 1
      Negat(Neg) = CODET(KM)
      Endif
C
      If(IG .lt. Lmin) Then
      Lmin = IG
      Codmax(2) = CODET(KM)
      endif
C     ||||||||||||||||||||||||
      If(Pim .lt. PI) Then
      Pim = PI
      Codmax(3) = CODET(KM)
      Endif
      If(Pam .gt. PI) Then
      Pam = PI
      Codmax(4) = CODET(KM)
      Endif
      If(Him .lt. HI) Then
      Him = HI
      Codmax(5) = CODET(KM)
      endif
      If(Ham .gt. HI) Then
      Ham = HI
      Codmax(6) = CODET(KM)
      Endif
C     ////////////////////////////////////////////////////////////
      Write(24,232)
      LINEMX = Title(KM)
      Tutu = '                                            '
      Do L = 1, 80
      Ser = LINEMX(L:L)
      If(Ser .eq. '[') Then
      Do M = 80,1,-1
      If(LINEMX(M:M) .ne. ' ') Then
      Mad = M
      Goto 72
      Endif
      Enddo
 72   Tutu = LINEMX(L:MAD)
      MM = MAD-L+1
      Goto 75
      Endif
      Enddo

 75   Write(24,235) KM, Curttt(4:15), Ltot(KM),Lat(KM),Lyt(KM),IG,
     #       Tutu(1:MM)
 235  Format(I3,1X,A,1X,I5,'AAs',1X,'|',I4,'-',I4,'/Alg =',I4,
     #  'AA ||',A)
      Write(33,233)
 233  Format(114('-'))
 232  Format(80('-'))
      Write(33,234) KM,Curttt(4:15),Ltot(KM),Lat(KM),Lyt(KM),IG,
     "   PI,PII(KM),HI,HII(KM),0.001*WM,0.001*WMM(KM),TITLE(KM)
```

```
C     Keep the record of whatis in the parantheses
      PIST(KM) = PII(KM)
      HIST(KM) = HII(KM)
C     Keep it for final step

      Write(25,64) KM, TITLE(KM)
      Write(25,66) PI, HI, WM, Curttt(4:15), Lat(KM), Lyt(KM)
C
 234  Format(I3,1X,A,1X,I5,'AAs',1X,'|',I4,'-',I4,'/Alg =',I4,'AA ||',
     #1X,'pI',F4.1,1X,'(',F4.1,')',' ||',1X,'HI',1X,F5.1,1X,'(',F5.1,')',
     #1X,'||',' Mass kDa',F5.1,1X,'(',F6.1,')',' ||'/A)
C
C     WARNING for border overflow - upperrange of the sequence
C
      If(Ltot(KM)-Lyt(KM) .lt. 0) Then
      Write(Jwrite,191) Lyt(KM), Ltot(KM)
      Write(55,55) Curttt(4:15),Lat(KM), Ltot(KM)
 55   format(A,10X,I4,5X,I5)
      endif

 191  Format('!!!!! Attention - chosen region out of length',2I6/)
      write(Jwrite,19) KM,IG,Ltot(KM),Lat(KM),Lyt(KM),Curttt(4:15),
     "      PI,PII(KM),HI,HII(KM),0.001*WM,WMM(KM),TITLE(KM)
      write(21,210) PI, HI
      write(23,210) PI, WM
 210  format(2(2X,F10.3))
C|||||||||||||||||||||||||||||||||||||||||||||||||||||||||||||||||
C     Filling up possible 'USER' database
C|||||||||||||||||||||||||||||||||||||||||||||||||||||||||||||||||
C     Filling up putative database for further 'USER' elaboration
      Write(18,11)
 11   Format('@')
      Write(18,14)
 14   Format('%')
      Write(18,40) CODET(KM)
      Write(18,44) TITLE(KM)
C|||||||||||||||||||||||||||||||||||||||||||||||||||||||||||||||||
 40   Format('>',2X,A25)
 44   Format('>',2X,A)
C
      Write(18,45) KM, WMM(KM), PII(KM), HII(KM)
 45   Format('#Dt',2X,I6,2X,F12.3,3X,F7.3,3X,F8.4)
      Write(18,46) Rota(1),Rota(2),Rota(3),Rota(4),Rota(5),Rota(4),
     # Rota(6),Rota(4),Rota(7),Rota(4),Rota(8),Rota(4),Rota(9),
     # Rota(4),Rota(10),Rota(4),Rota(11)
 46   Format(16(A3/),A3)
C|||||||||||||||||||||||||||||||||||||||||||||||||||||||||||||||||
C|||||||||||||||||||||||||||||||||||||||||||||||||||||||||||||||||
C|||||||||||||||||||||||||||||||||||||||||||||||||||||||||||||||||
C     ----- 12/6/2008 -----------------------------------
 19   Format(I3,'#',I4,'(',I5,'&',I5,I5,')',1X,A,F4.1,1X,
     "'(',F4.1,')',1X,F5.1,1X,'(',F5.1,')',1X,F5.1,1X,'(',F9.1,')'/A/)
      WRITE(12,20) Curttt(1:21)
      CURTIT = TITLE(KM)
      WRITE(12,20) CURTIT(1:78)
C
      Jhot = Jhot + 1
      Unders = '_'
```

```
      If(Decca .eq. 'P') Then
      Jot = 0
      Do J=1,80,1
      If(CURTIT(J:J) .eq. '[') Then
      Jot = J
      Lom = 11
      Do K = Jot+1,Jot+21
      If(CURTIT(K:K) .eq. ']') Then
      Lom = K
      GoTo 2240
      Endif
      Enddo

 2240 Mamez = CURTIT(Jot+1:Lom-1)
      Namex = Mamez
      Do N = 1,20
      If(Namex(N:N) .eq. ' ') Then
      Namex = Mamez(1:N-1)//'_'//Mamez(N+1:20)
      GOTO 7700
      Endif
      Enddo
 7700 Endif
      Enddo
C
      Gigat(Jhot) = Namex
      If(Jdiva .gt. 0) Then

      Do KK=1,Jdiva
      If(Jhot .le. Jbondo(KK)) Then
      Mamez = Namex
      Do J=1,25
      If(Mamez(J:J) .eq. ' ') Then
      Koq = J-1
      goto 5775
      endif
      enddo
 5775 Namex = Mamez(1:Koq)//CONSTER(KK)
      Gigat(Jhot) = Namex
      goto 7880
      endif
      Enddo
      Endif
      Else
      Do KK=1,Jdiva
      If(Jhot .le. Jbondo(KK)) Then
      Mamez = Codet(KM)
      DO Lv = 15,1,-1
      If(Mamez(Lv:Lv) .ne. ' ') Then
      Lov = Lv
      Goto 5730
      Endif
      enddo
 5730 Namex = Mamez(3:Lov)//Unders//CONSTER(KK)
      Write(66,662) Namex
 662  Format(1X,A)
      Goto 7880
      Endif
```

```
      Enddo
      Endif

 7880 Write(27,2020) Namex, CURTIT(1:55)
 2020 Format('>  ',A,4X,A)
C
      WRITE(14,202) Curttt(1:21), CURTIT(1:80)
      Curyyy = CURTIT(3:80)
      Write(20,225) KM, Curttt(3:20), Curyyy
 225  format('(',I3,')',1X,A15,2X,A)
      write(22,215) KM, Curttt(3:21), Namex, PI, HI
 215  format(I3,2X,A,3X,2X,A,1X,F5.2,3X,F7.2)
 202  format(A,4X,A)
      Write(10,24)
 24   Format('@')
      WRITE(10,20) Curttt(1:21)
      WRITE(10,20) CURTIT(1:78)
 20   FORMAT(A)
 25   FORMAT(70A1)
      write(12,25) (TSEQ(Jk),Jk=1,IG)
      write(27,25) (TSEQ(Jk),Jk=1,IG)
      write(14,25) (TSEQ(Jk),Jk=1,IG)
      Write(28,21) KM,PI,HI,WM,Curttt(4:18),CURTIT(3:80)
 21   Format(I4,2X,F5.2,2X,F5.1,2X,F8.1,2X,A,2X,A)
      KM = KM + 1
c      write(36,5100) KM
c 5100 format('Making code',I5)

C|||||||||||||||||||||||||||||||||||||||||||||||||||||||||
C     Go looping againuntil the endlist is reached (Codes)
C|||||||||||||||||||||||||||||||||||||||||||||||||||||||||

      IF(KM .LE. NCODE) GOTO 300

C
      PIa = PIa/Float(NCODE)
      HIa = HIa/Float(NCODE)
      Write(2,125) PIa, HIa, Pim, Codmax(3), Pam, Codmax(4),
     #  Him, Codmax(5), Ham, Codmax(6), Lmin, Codmax(2),
     #             Lmax, Codmax(1)
      Write(28,125) PIa, HIa, Pim, Codmax(3), Pam, Codmax(4),
     #  Him, Codmax(5), Ham, Codmax(6), Lmin, Codmax(2),
     #             Lmax, Codmax(1)

 125  Format(3X,'Average pI =',F5.2,3X,'Average HI =',F5.2/
     "       3X,'Maximal pI =',F5.2,3X,A/
     "       3X,'Minimal pI =',F5.2,3X,A/
     "       3X,'Maximal HI =',F5.2,3X,A/
     "       3X,'Minimal HI =',F5.2,3X,A/
     #       3X,'Minim-Code =',I5,2X,A/
     #       3X,'Maxim-Code =',I5,2X,A/)
      Do J=1,Neg
      Write(6,630) J, Negat(J)
 630  format(2X,'Warning >>>> Sequence with negative length',I5,2X,A)
      Enddo
      Do J=1,Jcod
      Write(6,620) J, Codx(J)
 620  Format(2X,'Warning >>>>> Sequence has X-residue',I5,2X,A)
```

```
      Enddo
C     ||||||||||||||||||||||||||||||||||||||||||||||||||
C     See if there are similar parameters pI - mass - HI
C|||||||||||||||||||||||||||||||||||||||||||||||||||||||
      Pip = PII(1)
      Hip = HII(1)
      Wip = WMM(1)
      I1 = 1
      DO j=2,NCODE
      If(Pip .eq. PII(j) .and. Hip .eq. HII(j) .and. Wip .eq. WMM(j))
     "   write(2,3000) I1,j
 3000 format(6X,'Sequences',1X,2I5,1X,'have similar data')
      Pip = PII(j)
      Hip = HII(j)
      Wip = WMM(j)
      I1 = j
      Enddo
C     ||||||||||||||||||||||||
C     ========================
C     Create an output file 16
C     ========================
      If(Decca .eq. 'P') Then
      Namex = Gigat(1)
      Loy = 1
 540  Do K=1+Loy,Ncode
      If(Namex .eq. Gigat(K)) Write(6,7500) Loy, K, Gigat(K)
 7500 format('Sequences',2I5,2X,'Have similar codes',A)
      Enddo
      Loy = Loy + 1
      Namex = Gigat(Loy)
      If(Loy .lt. Ncode) goto 540
      Endif
C
      DO J = 1,NCODE
      Curttt = CODET(J)
      Write(16,64) J, TITLE(J)
 64   Format(I4,1X,A)
      Write(16,66) PII(J), HII(J), WMM(J), Curttt(4:15), Lat(J), Lyt(J)
      Write(9,28) Lott(J), Lat(J), Lyt(J)
 28   Format(A,2X,2I10)
      ENDDO
 66   Format(3X,F7.2,3X,F5.2,3X,F10.2,2X,A,2(2X,I5))
      Write(Jw,545)
      write(28,545)
 545  Format(/2X,' Data-points ',1X,
     # 'Average || pI & sd   ||   HI & sd ||')
     F1 = 0.0
     H1 = 0.0
      Do L = 1,Ncode
      F1 = F1 + PIST(L)
      H1 = H1 + HIST(L)
      Enddo
C     Mean-values
      If(Ncode .gt. 0) Then
      F1 = F1/Float(Ncode)
      H1 = H1/Float(Ncode)
C     SD
      F2 = 0.0
```

```
      H2 = 0.0
      write(67,753) F1, H1
 753  format('Average',2F10.3)
      Do L = 1,Ncode
      F22 = PIST(L) - F1
      H22 = HIST(L) - H1
      write(67,677) F22, PIST(L), H22, HIST(L)
 677  format(4F10.3)
      F22 = F22**2
      H22 = H22**2
      F2 = F2 + F22
      H2 = H2 + H22
      Enddo
      F2 = Sqrt(F2/Float(Ncode))
      H2 = Sqrt(H2/Float(Ncode))
      write(67,653) F2, H2
 653  format('Final SD',2F10.5)
C     Skewness-AG
      Kol0 = 0
      Kol1 = 0
      Kol2 = 0
      Kol3 = 0
      Kol4 = 0
      Kol5 = 0
      Do L=1,Ncode
      F3 = PIST(L) - F1
      H3 = HIST(L) - H1
      write(67,622) F3, H3
 622  format('H3333',2F10.5)
      If(F3 .eq. 0.0) Kol0 = Kol0 + 1
      If(F3 .lt. 0.0) Kol1 = Kol1 + 1
      IF(F3 .gt. 0.0) Kol2 = Kol2 + 1
      If(H3 .eq. 0.0) Kol5 = Kol5 + 1
      If(H3 .lt. 0.0) Kol3 = Kol3 + 1
      If(H3 .gt. 0.0) Kol4 = Kol4 + 1
      Enddo
      write(67,679) Kol0, Kol1, Kol2, Kol5, Kol3, Kol4
 679  format(6I10)
      F4 = 0.0
      H4 = 0.0
      F5 = 0.0
      H5 = 0.0
      Nske = Ncode - Kol0
      Nkur = Ncode - Kol5
      write(67,543)
 543  Format('p-I-')
      Do L=1,Nske
      AAA = (PIST(L)-F1)**3
      F4 = F4 + AAA
      BBB = (PIST(L)-F1)**4
      F5 = F5 + BBB
      Write(67,388) PIST(L), F1, AAA, F4, BBB, F5
      Enddo
      Write(67,328)
 328  format('HIHI')
      Do L=1,Nkur
      AAA = (HIST(L)-H1)**3
      H4 = H4 + AAA
```

```
      BBB = (HIST(L)-H1)**4
      H5 = H5 + BBB
      write(67,388) AAA, H4, BBB, H5
 388  format(6F10.3)
      Enddo

      gif = F4/Float(Nske-1)
      hif = H4/Float(Nkur-1)
      gaf = F5/Float(Nske-1)
      haf = H5/Float(Nkur-1)
      giff = gif/F2**3
      hiff = hif/H2**3
      gaff = gaf/F2**4
      haff = haf/H2**4
      Endif

      Write(Jw,885) Ncode, F1, F2, H1, H2
      write(28,885) Ncode, F1, F2, H1, H2
 885  Format(7X,I5,13X,2F5.1,' ||||',2F6.1/)
      Write(Jw,880) Kol1, Kol2, Kol3, Kol4, giff, gaff, hiff, haff
      Write(28,880) Kol1, Kol2, Kol3, Kol4, giff, gaff, hiff, haff
 880  Format(3X,'pIs < (mean pI) ',I3,2X,'///',2X,'pIs > (mean pI) ',
     # I3,' || '//3X,'HIs < (mean HI) ',I3,2X,'///',2X,
     # 'HIs > (mean HI) ',I3,' || '/
     #/3X,'Skewness /||/ Kurtosis : pI',2F7.3,' | '/
     #/3X,'Skewness /||/ Kurtosis : HI',2F7.3,' | '/)
C
CCC   Insert July-2014
C
 9999 RETURN
      END
C     ||||||||||||||||||||||||||||||||||||||||||||||||||||||||||||
C++++++++++++++++++++++++++++++++++++++++++++++++++++++++++++++++C
C Subroutine for deduction of input data in the following form C
C Character *** Integer *** Integer introdued as LINE(1:80)    C
C This format is necessary if free space in LINE are used as a C
C principal attribute for finding the lower-upper limits for   C
C procesing given sets of sequences                            C
C Author A. Galat 6/8/98                                       C
C++++++++++++++++++++++++++++++++++++++++++++++++++++++++++++++++C
C     Version 1.1 6/8/98
      SUBROUTINE DEDUCT(KREAD,Isi,NCODE,IPA,NEX,CODEX,DECOD,IS,IE)
      Parameter (IDY=1500)
      CHARACTER*80 LINE, Terpy(IDY)
      CHARACTER*25 TEMPR(IDY), DECOD(IDY), CODEX(IPA), COP, KOF
      INTEGER IS(IPA), IE(IPA), IDD
C     ////////////////////////////
      Lcon = 1
      Isi = 0
 3000 READ(KREAD,10,End=4000,ERR=4000) LINE
      IF(Line(1:1) .eq. '!') goto 3000
      Terpy(Lcon) = LINE
      Lcon = Lcon + 1
      goto 3000

 4000 Write(6,530) Lcon
 530  format('Lcon=',I7)
      LLL = 1
```

```
      LRR = 0
 450  COP = CODEX(LLL)

      Do J = 1,Lcon
      Line = Terpy(J)
      KOF = Line(1:20)
      IF(COP(1:20) .eq. KOF) Then
      write(96,66) Line
 66   Format(A)
      LRR = LRR + 1
      goto 4500
      Endif
      enddo

 4500 LLL = LLL + 1
      IF(LLL .gt. NCODE) goto 5500
      Goto 450
C
 5500 Rewind(96)
      JR = 0
 1000 READ(96,10,END=2000,ERR=2000) LINE
 10   FORMAT(A)
      DECOD(NEX) = '                           '
      COP = '                         '
      J0 = 0
      KN = 0
      IRET = 0
C|||||||||||||||||||||||||||||||||||||||||||||||||||||||||||||
C
C     There is possibility to put the sign !!!!!!!!!!!!!!!!!!
C
      If(LINE(1:1) .eq. '!') goto 1000
 500  J0 = J0 + 1
      IF(LINE(J0:J0) .EQ. ' ') GOTO 500
C     Find the begining and ending 'WORD'
      IF(LINE(J0:J0) .NE. ' ') THEN
      K1 = J0
      COP(1:1) = LINE(K1:K1)
      DO I = K1+1,80
      KN = KN + 1
      IF(LINE(I:I) .EQ. ' ') GOTO 600
      COP(1:KN+1) = COP(1:KN)//LINE(I:I)
      KOP = I
      ENDDO
      ENDIF
C     ================
 600  KOF = DECOD(NEX)
      DECOD(NEX) = COP(1:KN)//KOF(KN+1:25)
      JR = JR + 1
      TEMPR(JR) = COP(1:KN)
C     See if repeated CODE was used
      If(JR .gt. 1) Then
      Do L1=1,JR-1
      IF(COP(1:KN) .eq. TEMPR(L1)) Then
      Write(6,60) L1, COP(1:KN)
 60   format('>>>>>>>>> Code repeated',2X,I5,3X,A)
      goto 70
      endif
```

```
      Enddo
      Write(3,10) LINE
      Endif
C
 70   Do KL=1,NCODE
      If(DECOD(NEX) .eq. CODEX(KL)) goto 650
      enddo
      GOTO 1000
 650  J0 = KOP
 700  J0 = J0 + 1
      IF(LINE(J0:J0) .EQ. ' ') GOTO 700
      IF(LINE(J0:J0) .NE. ' ') THEN
      K1  = J0
      KOP = K1
      DO I = K1 + 1,80
      IF(LINE(I:I) .EQ. ' ') GOTO 800
      KOP = I
      ENDDO
      ENDIF
C     Convert character data into an integer
 800  IP = KOP - K1
c      write(6,*) IP
      IF(IP .EQ. 0) WRITE(15,51) LINE(K1:KOP)
      IF(IP .EQ. 1) WRITE(15,52) LINE(K1:KOP)
      IF(IP .EQ. 2) WRITE(15,53) LINE(K1:KOP)
      IF(IP .EQ. 3) WRITE(15,54) LINE(K1:KOP)
      IF(IP .EQ. 4) WRITE(15,55) LINE(K1:KOP)
 51   FORMAT(A1)
 52   FORMAT(A2)
 53   FORMAT(A3)
 54   FORMAT(A4)
 55   format(A5)
c     REWIND(15)
      backspace(15)
      READ(15,*) IDD
c     CLOSE(15,DISPOSE='DELETE')
      IF(IRET .EQ. 0) IS(NEX) = IDD
      IF(IRET .EQ. 1) IE(NEX) = IDD
      IRET = IRET + 1
      J0 = KOP
      IF(IRET .LE. 1) GOTO 700
      NEX = NEX + 1
      GOTO 1000
 2000 RETURN
      END
C=================================================================C
C     Translate sequence into an array of integers (ISEQ)        C
C     Author: A. Galat (August, 2nd, 1991)                        C
C=================================================================C
      SUBROUTINE TRANSS(KOUNT,ISEQ,TSEQ)
C     Character data sequence processing codes (one-letter code)
      Parameter(NSQ=38000)
      CHARACTER*1 AMINOS(22)
      INTEGER ISEQ(NSQ)
      CHARACTER*1 TSEQ(NSQ)
      DATA AMINOS/'A','R','N','D','C','Q','E','G','H','I','L','K',
     +            'M','F','P','S','T','W','Y','V','/','X'/
      DO J = 1,KOUNT
```

```fortran
      DO I = 1,22
      IF(TSEQ(J) .EQ. AMINOS(I)) ISEQ(J) = I
      ENDDO
      ENDDO
      RETURN
      END
C==================================================================C
C     Calculation of protein hydrophobicity from its sequence     C
C     Author: A. Galat (October 1988)                              C
C==================================================================C
      SUBROUTINE HYDROP(KOUNT,ISEQ,HI,Ir,Jsf)
C     For extensive review of hydrophobicity SCALES see ref:
C     Cornette, J.L., Cease, K.B., Margalit, H., Spouge, J.L.,
C     Berzofsky, J.A. & DeLisi, Ch., J. Mol. Biol. 195:659-685:1987
      Parameter(NSQ=38000)
      REAL SCALE(22)
C     Hydrophobicity scale of Kyte and Doolittle (J. Kyte and
C     R.F. Doolittle, J. Mol. Biol. 157:105-132:1982)
      DATA (SCALE(J),J=1,22)/1.8,-4.5,-3.5,-3.5,2.5,-3.5,-3.5,-.4,
     # -3.2,4.5,3.8,-3.9,1.9,2.8,-1.6,-.8,-.7,-.9,-1.3,4.2,.0,.0/
      Integer ISEQ(KOUNT)
      Dimension VAR(NSQ), VAL(NSQ)
C     First executable statement (moving window = middle)
      Fic = Float(Jsf)
      MIDDLE = Jsf/2.0 + 1
      ISTART = MIDDLE
      IEND = KOUNT - MIDDLE + 1
C     Set-up hydrophobicity values vector VAR
      DO 2 I  = 1,KOUNT
      TEMP = SCALE(ISEQ(I))
      VAR(I) = TEMP
 2    CONTINUE
C     Calculate averaged hydrophobicity value for the central residue
      DO 5 I = ISTART,IEND
      RE     = 0.0
      DO 4 J = 1,Jsf
      RE     = RE + VAR(I-MIDDLE+J)
 4    CONTINUE
      VAL(I)  = RE/Fic
 5    CONTINUE
C     Calculate the percentage of hydrophobic segments (Hi)
      IL2  = 0
      DO J = ISTART,IEND
      IF(VAL(J) .GE. 0.0) IL2 = IL2 + 1
      ENDDO
      IF(KOUNT .GT. 0) HI = FLOAT(IL2)*100.0/FLOAT(KOUNT)
      RETURN
      END
C-----------------------------------------------------------C
C     Calculate mass and composition in % of amino acids C
C     Author A. Galat - October 1988                     C
C-----------------------------------------------------------C
      SUBROUTINE MASSPR(KOUNT,ISEQ,WM,Ir)
      Parameter(NSQ=38000)
      INTEGER ISEQ(NSQ), IPN(20)
      REAL PN(20)
C     Amino acid molecular mass - mass of water (for peptide bond formation)
      REAL AMIWEI(21)
```

```
      DATA AMIWEI/
     "  71.07884, 156.18764, 114.10392, 115.08863, 103.14484, 128.13081,
     " 129.11552,  57.05196, 137.14120, 113.15948, 113.15948, 128.17416,
     " 131.19861, 147.17661,  97.11672,  87.07824, 101.10512, 186.21327,
     " 163.17601,  99.13260,  18.01528/
      Character*4 AMINOT(21)
      DATA AMINOT
     #      /'Ala-','Arg-','Asn-','Asp-','Cys-','Gln-','Glu-','Gly-',
     #       'His-','Ile-','Leu-','Lys-','Met-','Phe-','Pro-','Ser-',
     #       'Thr-','Trp-','Tyr-','Val-','Ace-'/
C|||||||||||||||||||||||||||||||||||||||||||||||||||||||||||||||||
      WM = 0.0
      DO J = 1,20
      IPN(J) = 0
      ENDDO
      DO 2 J2 = 1,KOUNT
      DO 1 J1 = 1,20
      IF(ISEQ(J2) .EQ. J1) THEN
      WM = WM + AMIWEI(J1)
      IPN(J1) = IPN(J1) + 1
      GOTO 2
      ENDIF
 1    CONTINUE
 2    CONTINUE
      WM = WM + AMIWEI(21)
      If(Ir .eq. 0) goto 99
      DO J = 1,20
      PN(J) = 100.*FLOAT(IPN(J))/FLOAT(KOUNT)
      ENDDO
C     Forming an external bucket with AAC of each entry
      Write(17,90) (PN(J),J=1,20)
 90   format(10(F6.3,1X))
 99   RETURN
      END
C-----------------------------------------------------------C
C     A Fortran-77 program to calculate pI from sequence    C
C     See S. Skoog and A Wichman - Trends in Analytical     C
C     Chemistry Vol. 5, pp. 82-83, 1986                     C
C     Author: A. Galat, July 1995                           C
C-----------------------------------------------------------C
      SUBROUTINE PICAL(ITD,PIC,TSEQ)
      Parameter(NSQ=38000)
      EXTERNAL FUNC
      REAL MINIF
      Real zbrent
      CHARACTER*1 TSEQ(NSQ)
      CHARACTER*1 AMINOS(22)
      COMMON IC,ID,IE,IH,IK,IR,IY, PI(9)
      DIMENSION CTERM(20), CNTERM(20)
C               R    K    Y   C   H   D   E  NT  CT
      DATA PI/12.5,10.5,10.1,8.3,6.0,3.9,4.3,0.0,0.0/
      DATA CTERM/2.4,2.2,2.1,2.1,1.7,2.2,2.2,2.4,1.8,2.3,
     "           2.3,2.2,2.1,2.2,2.0,2.2,2.1,2.4,2.2,2.3/
      DATA CNTERM/9.9,9.0,8.8,9.8,10.8,9.1,9.7,9.8,9.2,9.8,
     x            9.7,9.0,9.3,9.2,10.6,9.2,9.1,9.4,9.1,9.7/
      DATA AMINOS/'A','R','N','D','C','Q','E','G','H','I','L','K',
     +            'M','F','P','S','T','W','Y','V','Z','X'/
C|||||||||||||||||||||||||||||||||||||||||||||||||||||||||||||||||
```

```
C     Preset starting parameters
      If(ITD .eq. 0) goto 99
      KOUNT = ITD
      KL  = 0
      KM  = 0
      PH1 = 1.0
      PH2 = 14.0
C    Calculate the integer number of charged AA
      IR = 0
      IK = 0
      IH = 0
      IC = 0
      ID = 0
      IE = 0
      IY = 0
      DO J = 1,KOUNT
      IF(TSEQ(J) .EQ. 'R') IR = IR + 1
      IF(TSEQ(J) .EQ. 'K') IK = IK + 1
      IF(TSEQ(J) .EQ. 'H') IH = IH + 1
      IF(TSEQ(J) .EQ. 'C') IC = IC + 1
      IF(TSEQ(J) .EQ. 'D') ID = ID + 1
      IF(TSEQ(J) .EQ. 'E') IE = IE + 1
      IF(TSEQ(J) .EQ. 'Y') IY = IY + 1
      ENDDO
C     Find the N- and C-terminal AA
      N9 = 0
      K9 = 0
      DO J = 1,20
      IF(TSEQ(1) .EQ. AMINOS(J)) N9 = J
      IF(TSEQ(KOUNT) .EQ. AMINOS(J)) K9 = J
      ENDDO
      PI(8) = CNTERM(N9)
      PI(9) = CTERM(K9)
C     Decison made search for pI
c     PIC = MINIF(FUNC,PH1,PH2,XACC,MAXEL)
c     PIC = rtsec(func,PH1,PH2,xacc,MAXEL)
      PIC = zbrent(FUNC,PH1,PH2,XACC,MAXEL)
 99   RETURN
      END
C-------------------------------------------C
C     Find charge on the protein            C
C-------------------------------------------C
      FUNCTION FUNC(PH)
C
      COMMON IC,ID,IE,IH,IK,IR,IY,PI(9)
      SUM = 0.0
      SUM = SUM - ID*PNCHARGE(6,PH,PI)
      SUM = SUM - IE*PNCHARGE(7,PH,PI)
      SUM = SUM - IC*PNCHARGE(4,PH,PI)
      SUM = SUM - IY*PNCHARGE(3,PH,PI)
      SUM = SUM + IH*PCHARGE(5,PH,PI)
      SUM = SUM + IK*PCHARGE(2,PH,PI)
      SUM = SUM + IR*PCHARGE(1,PH,PI)
      TEM = PCHARGE(8,PH,PI)
      SUM = SUM + TEM
      TEM = PNCHARGE(9,PH,PI)
      SUM = SUM - TEM
      FUNC = SUM
```

```
      RETURN
      END
      FUNCTION PCHARGE(N,PH,PI)
      DIMENSION PI(*)
      ALFA = EXP(2.3026*(PH-PI(N)))
      PCHARGE = 1.0/(1.0 + ALFA)
      RETURN
      END
      FUNCTION PNCHARGE(N,PH,PI)
      DIMENSION PI(*)
      ALFA = EXP(2.3026*(PH-PI(N)))
      PNCHARGE = ALFA/(1.0 + ALFA)
      RETURN
      END
C|||||||||||||||||||||||||||||||||||||||||||||||||||||||||||||
C------------------------------------------------------------C
C     The Brent's method of finding the root of a function  C
C     FUNC known to lie between X1 and X2                   C
C     Buy and copy it from the book by W.H. Press,          C
C     B.P. Flannery, S.A. Teukolsky and W.T. Vetterling     C
C     Numerical Recipes - The Art of Scientific Computing   C
C     Cambridge University Press, 1996.                     C
C------------------------------------------------------------C
      FUNCTION zbrent(FUNC,X1,X2,TOL,MAXEL)
      PARAMETER (ITMAX=100,EPS=3.E-8)
      COMMON IC,ID,IE,IH,IK,IR,IY, PI(9)
      EXTERNAL FUNC
```
