# Supplementary material for: Compression of Large Sets of Sequence Data Reveals Fine Diversification of Functional Profiles in Multigene Families of Proteins: A Study for Peptidyl-Prolyl cis/trans Isomerases (PPIase)
Source: Biomolecules. 2019 Feb 11;9(2):59. doi: 10.3390/biom9020059 (PMC6406343; doi:10.3390/biom9020059)
Supplement: Supplementary file 1 [file biomolecules-09-00059-s001.zip › Figure S3 - source code of Map_display.pdf]

```
C                               *
C                               *
C                             * * *
C                           * * * * *
C                         * * * * * * *
C                       * * * * * * * * *
C                     * * * * * * * * * * *                   *
C                   * * * * * * * * * * * * *                 *
C         *                     *                           * * *
C         *                     *                         * * * * *
C       * * *                   *                       * * * * * * *
C     * * * * *                 *                     * * * * * * * * *
C         *                     *                             *
C         *                   * * * * *                       *
C-----------------------------------------------------------------------C
C                      ((( Map_Display)))                               C
C                   *   *   *   *   *   *   *                           C
C      This is a Fortran-77 program which can be used for analyses      C
C      of multiple sequence alignment of protein sequences              C
C      The input file to this program msa9.inp                          C
C                                                                       C
C                   Programmed by Andrzej Galat                         C
C                                                                       C
C     1) The program shall be used for scientific purpose only,         C
C        excluding industrial or commercial purpose.                    C
C     2) Proper acknowledgement shall be made to the author of the      C
C        program in publications resulting from the use of Map_Display C
C     3) The program shall not be made available to users outside       C
C        the recipient's laboratory, unless written consent is          C
C        obtained.                                                      C
C+++++++++++++++++++++++++++++++++++++++++++++++++++++++++++++++++++++++C
C     This program is distributed in the hope that it will be useful,
C     but WITHOUT ANY WARRANTY; without even the implied warranty of
C     MERCHANTABILITY or FITNESS FOR A PARTICULAR PURPOSE!
C     -----------------------------------------------------------------
C     Overhauled on 17/6/2010 - 23-8-2017
C     |||||||||||||||||||||||||||||||||||||||||||||||||||||||||||||||||
C     Multiple MSAs are analysed and composite fort.24 created 21/1/2014
C     Version (1.21) 1/4/98 - Overhauled on 17/8/98 - 25-6-2014
C     Overhauled 27/6/2007 (AG)
C     Attention for the MSA from ClustalW (>500 sequences)
      PROGRAM Map_Displey
      PARAMETER (NROW=1000,NCOL=250)
      Character*90 LINE, KIN, TITLE
      Character*60 Filnam
      Character*76 Buff(2)
      Character*19 CODEX(NROW)
      Character*1 SeqRef, Assa(30), TSEQ, Pan, SEQ, REQ, STR(NCOL)
      Common/panty/Jshiff, TSEQ(NROW), Pan(NCOL,50)
      Common/Seqqqq/ SEQ(NROW,NCOL), REQ(NROW,NCOL)
C
C     SeqRef - reference sequence alignment for 'Heat-Maps'
C
      Common/kola/Jhut(30),Saxyy(NCOL),Saxzz(NCOL), DiSeQQ(NCOL),
     #  Tuna(NCOL,50),Sunn(NCOL,50), Bunn(Ncol,50)
      Common/Fory/ Kac, SeqRef(NCOL,40)
      Character*1 Tan(300,50), San(300,50), Star(1000), Seqq(1000)
      Character*1 AMINOS(22), SYY(NCOL)
```

```
      Character*1 AB, ABE(NCOL),Aminss(22)
      Character*3 Fogg(5)
      Character*5 Intr(25)
      Common ISEQ(NCOL,22), IBE(NCOL)
      Common/angel/ AMIWEI(22), HYDRO(22), FTOT(NCOL), Hot(300,50)
      Character Apos*5, Pats*1
      Common /final/ Joh(50), Apos(50,200), Pats(50,200)
      Real Panna(300,40)
C     Number of interactions taken from seqelf
c      Integer Inter1(300), Itera2(300)
      INTEGER Ipop(8), Ioto(6,50), ICHAR(22), JTOT(NCOL)
      Logical Search, Found
C     ---------------------------------------------------------
      DATA ICHAR/0,0,-1,-1,0,0,0,0,+1,0,0,0,0,0,+1,0,0,0,0,0,0,0/
      Data Fogg/'Tot','Sig','aa|','Frq','Ent'/
      DATA AMINOS/'A','C','D','E','F','G','H','I','K','L',
     r            'M','N','P','Q','R','S','T','V','W','Y','-','X'/
      Data Aminss/'a','c','d','e','f','g','h','i','k','l',
     z            'm','n','p','q','r','s','t','v','w','y','-','x'/
      DATA AMIWEI/ 71.07884, 103.14484, 115.08863, 129.11552, 147.17661,
     z             57.05196, 137.14120, 113.15948, 128.17416, 113.15948,
     z            131.19861, 114.10392,  97.11672, 128.13081, 156.18764,
     z             87.07824, 101.10512,  99.13260, 186.21327, 163.17601,
     z              0.00000,0.00000/
C     Inverse of Alog(2)
      Data Aln2/1.442695042/
C     Aln(20.0) = 2.995732300 its inverse 0.333808200
      Data Aln20/0.333808200/
      DATA HYDRO/1.8,2.5,-3.5,-3.5,2.8,-0.4,-3.2,4.5,-3.9,3.8,1.9,-3.5,
     r           -1.6,-3.5,-4.5,-0.8,-0.7,4.2,-0.9,-1.3,0.0,0.0/
C     -----------------
C     Open output files
C     -----------------
      Do J=1,NCOL
      Seq(1,J) = ' '
      Enddo

      OPEN(UNIT=2,FILE='msa9.out',status='unknown')
      Open(Unit=4,File='msaent.int',status='unknown')
      OPEN(UNIT=9,FILE='msaent.dss',status='unknown')
      OPEN(UNIT=10,FILE='msaent.hyd',status='unknown')
      OPEN(UNIT=11,FILE='msaent.mas',status='unknown')
      OPEN(UNIT=14,FILE='msaent.hydt',status='unknown')
      OPEN(UNIT=15,FILE='msaent.mast',status='unknown')
      Open(Unit=17,File='msa.som',status='unknown')
      OPEN(UNIT=1,FILE='msa9.inp',status='old')
C     |||||||||||||||||||||||||||||||||||||||||||||||||||||||||||||||||
      Job = 1
      Do J = 1,30
      Jhut(J) = 0
      Enddo
      Jhitmax = 0
C     -------------------------------------------------------------------
C     Read number of aligned sequneces (NLINE) and volume of pages (NPAGE)
C     Entropy/sequence dump file 24

      Jstep = 0
      Write(24,240)
```

```
      Write(19,240)
 240  Format(/20X,'Shannon entropy (Ie) coding system'/
     #10X,' -----------------------------------------------------------'/
     #10X,' 0.0  eq * / 0.01-0.25 eq + / 0.251-0.5 eq = / 0.51-1 eq |'/)

      READ(1,1) LINE
      WRITE(2,11) LINE
      WRITE(9,11) LINE
      WRITE(10,11) LINE
      WRITE(11,11) LINE
      Read(1,110) Ncase
      Nlarge = 0
      Jshiff = 0
      Jff = 0

C     Grande - Grande - Grande Loop

 9800 READ(1,110) NLINE
      Jstep = Jstep + 1
      IAC = 0
      IBC = 0
      Search = .True.
      Found = .False.
      Write(2,1110) Job
 1110 Format(//10X,'ATTENTION ::: Job stream |||||| Pass: ' I5//)
      READ(1,110) NPAGE
      Read(1,112) Filnam
 112  Format(A)
 110  format(I10)
C     ||||||||||||||||||||||||||||||||||||||||||||||
      WRITE(2,120) Ncase, NLINE, NPAGE, Filnam
 120  FORMAT(10X,'Number of cases to be treated',I6/
     1       10X,'Number of sequences aligned =',I6/
     r       10X,'Number of pages occupied    =',I6/
     #       10X,'File-name:::',A)
      Jbuf = 0
C     ||||||||||||||||||||||||||||||||||||||||||||||||||
C     Read in the firts two line from clustal_W output
      Open(Unit=3,File=Filnam,status='old')
C     Then go on with MSA and establish its length and coding range
  5   READ(3,1,END=600) TITLE
      READ(3,1,END=600) LINE
      Buff(1) = TITLE(1:76)
      Buff(2) = LINE(1:76)
 4    format(A)
 1    FORMAT(A)
 11   FORMAT(A/)
 10   Read(3,1,End=600) Line
      IP = 1

 100  READ(3,1,END=600) LINE
      If(Search) then
      Do J=90,1,-1
      If(LINE(J:J) .ne. ' ' .AND. .not.Found) then
      Found = .true.
      Lbeg = J
      Endif
      IF(Found .and. LINE(J:J) .eq. ' ') Then
```

```
      Jbeg = J
      Lend = Lbeg - Jbeg
      Write(2,7) Lend, Jbeg
 7    format(/10X,'MSA-block-length',I3,2X,'Seq # 1 starts at',I4)
      Rewind(3)
      Search =.false.
      GOTO 5
      Endif
      Enddo
      Endif
C====================
      KIN = LINE
      CODEX(IP) = LINE(1:Jbeg)
      DO J=1,Lend
      AB = LINE(J+Jbeg:J+Jbeg)
      SEQ(IP,J+IAC) = AB
C
C     Set-up reference sequence
C
      If(Job .eq. 1) SeqRef(J+IAC,IP) = AB

      If(Jshiff .eq. 0) Pan(J+IAC,IP) = AB

      ENDDO
      IP = IP + 1
      IF(IP .LE. NLINE+1) GOTO 100
      IAC = IAC + Lend
      IBC = IBC + NLINE
      IF(IBC .LT. NLINE*NPAGE) GOTO 10
 600  CLOSE(3)
C     ||||||||||||||||||||||||||||||||
C     ---------------------
      If(Jstep .gt. 1) goto 400
      If(Jstep .gt. 1) goto 400
      write(97,97)
 97   Format('Stating length')
      Do j=1,NCOL
      write(97,987) j, Seq(1,j)
 987  format(I5,2X,A1)
      enddo

      Klun = 0
      Do K=1,NCOL
      If(Seq(1,K) .eq. ' ')Then
      Kac = K-1
      goto 565
      Endif
      Enddo
 565  write(57,570) Kac, IAC, NCOL
      write(57,57) (Seq(1,Kop),Kop=1,NCOL)
 57    Format(60A1)
 570   format('Kac,  IAC=',3I8)
      Do J = 1,Kac
      write(57,572) J, (SeqRef(J,L),L=1,IP-1)
      enddo
      Do J=1,Kac
      write(57,572) J, (Pan(J,L),L=1,IP-1)
 572   format(I5,1X,30(A1,1X))
```

```
      enddo
C     Real length of sequence plus gaps

 400  Do K=1,500
      If(Seq(1,K) .eq. ' ')Then
      Kat = K-1
      goto 5650
      Endif
      Enddo
 5650 SEQ(1,IAC+1) = ' '

C     Calculate the pIs of each protein fragment

      Write(88,549) Job, NLINE
 549  Format('######',2X,'This is step ',I5,' entries :',2I5)
      Do J = 1,NLINE
      write(88,888) (SEQ(J,K),K=1,Kat)
 888  format(70A1)
      enddo

      If(Jstep .eq. 1) Kat = Kac
      Do J = 1,NLINE
      Jak = J
      Krel = 0
      do 280 K = 1,Kat
      AB = SEQ(Jak,K)
      IF(AB .eq. '-' .OR. AB .eq. ' ') goto 280
      Krel = Krel + 1
       write(69,*) Job, Krel
      TSEQ(Krel) = SEQ(Jak,K)
 280  Continue
      If(Job .eq. 2) Then
      write(89,765) Krel
 765  format('Krel',I5)
      write(89,89) (TSEQ(Ln),Ln=1,Krel)
 89   format(60A1)
      Endif
C
      Write(6,672) NLINE, Kat, Krel
 672  format('NLINE',3I6)
      PIC = 0.0
      write(6,871) (TSEQ(I),I=1,Krel)
 871   format(60A1)
      Call PICAL(Job,Jak,Krel,PIC,TSEQ)
      write(88,551) Jak, PIC
 551  Format(I6,2X,F6.3)
      Enddo

C     Real length of Letter-code in the first sequence

      Lmm = 0
      Do J=1,IAC+1
      AB = SEQ(1,J)
      Jakk = J
      If(AB .eq. ' ') Then
      Lmm = J-1
      GOTO 990
      Endif
```

```
      Enddo
 990  IM = Lmm
      IX = 1

      Write(66,766)IAC, IBC,  NLINE+1, Jakk, IM
 766  format('NLINE',5I8)
       Do K=1,NLINE+1
      write(66,778) CODEX(K), (SEQ(K,J),J=1,IM)
 778  format(A,2X,60A1)
      enddo

C     Set up counter matrix
      DO J=1,IM
      DO I=1,22
      ISEQ(J,I) = 0
      ENDDO
      ENDDO

 1000 DO J = 1,NLINE
      JA   = J
      DO I = 1,22
      IF(SEQ(JA,IX) .EQ. AMINOS(I) .OR. SEQ(JA,IX) .EQ. Aminss(I))
     # ISEQ(IX,I) = ISEQ(IX,I) + 1
      ENDDO
      ENDDO
      IX = IX + 1
      IF(IX .LE. IM) GOTO 1000
      Write(6,5617) IX, IM
 5617  format('IXIM',2I10)
C     --------------------------------
C     Chose the highest score for AA

C     making new paretheses for future MSA

      IGF = 0
      JGH = IM

C     Beginning of first sequence-Letter in the MSA
      Do J=1,IM
      If(SEQ(1,J) .ne. '-') then
      IGF = J
      goto 465
      endif
      enddo

 465  Write(2,517) IAC, JGH
 517  format(10X,'Total block-length',I5/10X,'Coding-length',I5/)

C     Create new input data to damma

      Do J = 1,IM
      JTOT(J) = 0
      FTOT(J) = 0.0
      STR(J) = ' '
      SYY(J) = ' '
      IBE(J) = 0
      ABE(J) = ' '
      Enddo
```

```
      Do K=1,NLINE
      Kot = K
      Lbox = 0

      Do J=1,IM
      If(SEQ(Kot,J) .ne. '-' .AND. J .LT. IGF) Lbox = Lbox + 1
      ENDDO

      Kbox = 0
      Nbox = 0

      Do J=IM,1,-1
      If(SEQ(Kot,J) .ne. '-' .AND. J .GT. JGH) Nbox = Nbox + 1
      Enddo

      If(Kbox .gt. 0 .OR. Mbox .gt. 0) then
         If(Kbox .eq. 0) Kbox = 1
         write(43,44) CODEX(Kot), Kbox, Mbox
      endif
      If(Lbox .gt. 0 .OR. Nbox .gt. 0) then
         write(45,45) CODEX(Kot)
         write(44,44) CODEX(Kot), Lbox, Nbox
 44   format(A16,2X,I3,3X,I3)
 45   format(1X,A16)
      endif
      Enddo

C     Ending phase

      IU = 0
      DO J=1,IM
      JA = J
      IU = IU + 1
      IJ = 0
      DO K=1,22
      IF(ISEQ(JA,K) .GT. IJ) THEN
      IJ = ISEQ(JA,K)
      IBE(IU) = IJ
      ABE(IU) = AMINOS(K)
c      write(6,5511) J, IJ, AMINOS(K)
      ENDIF
      ENDDO
      ENDDO
C     Calculate some statistical data
      AHYDP = 0.0
      AHYDM = 0.0
      AMASP = 0.0
      AMASM = 0.0
      IAHP  = 0
      IAHM  = 0
      IAMP  = 0
      IAMM  = 0
      ISTOT = 0
      KSTOT = 0
      WRITE(9,9)
 9    FORMAT(3X,' ',1X,'#  AAC',3X,'Tot',1X,'+',2X,'-',1X,2X,'Hy+',
     e       2X,'Hy-',2X,'Tot',3X,'+',4X,'-',3X,'Ma+',2X,'Ma-')
```

```
      KOL = 0
      DO K = 1,IM
      DO L = 1,20
      KOL  = KOL + ISEQ(K,L)
      ENDDO
      ENDDO
      WRITE(2,14) KOL
 14   FORMAT(10X,'Number of residues aligned  =',I8/)
      write(6,521) IU, IBE(IU), ABE(IU)
 521  format('IU',I4,1X,I4,1X,A1)

C     Grand looping

      DO 9000 J=1,IM+1
      JA = J
      AB = ABE(JA)
      DO K = 1,20
      IF(AB .EQ. AMINOS(K)) IYY=K
      ENDDO
      HYDREF = HYDRO(IYY)
      VMASRE = AMIWEI(IYY)
      KX   = 0
      ITOT = 0
      KMXX = 0
      DO K=1,20
      IF(ISEQ(JA,K) .GT. 0) ITOT = ITOT + ISEQ(JA,K)
      IF(ISEQ(JA,K) .GT. KMXX) THEN
      KMXX = ISEQ(JA,K)
      KX = K
      ENDIF
      ENDDO
      JTOT(JA) = ITOT
      FTOT(JA) = FLOAT(KMXX)/FLOAT(JTOT(JA))
      STR(JA) = ' '
      SYY(JA) = ' '

C     If consensus seqeunce was found
      IF(ITOT .EQ. IBE(JA)) Then
      STR(JA) = '*'
      SYY(JA) = '*'
      Endif

 550  IYPP  = 0
      IIPP  = 0
      IMMA  = 0
      JMMA  = 0
      IUPS  = 0
      DO 200 K=1,20
      IF(K .EQ. IYY) GOTO 200
      IF(ISEQ(JA,K) .GT. 0) THEN
      IUPS = IUPS + 1
C     Less hydrophobic than the reference (most populated) amino acid
      IF(HYDREF .GE. HYDRO(K))  IYPP = IYPP + ISEQ(JA,K)
C     More hydrophobic than the reference amino acid
      IF(HYDREF .LT. HYDRO(K))  IIPP = IIPP + ISEQ(JA,K)
C     Less bulky amino acid than the reference
      IF(VMASRE .GE. AMIWEI(K)) IMMA = IMMA + ISEQ(JA,K)
C     More bulky amino acid than the reference
```

```
      IF(VMASRE .LT. AMIWEI(K)) JMMA = JMMA + ISEQ(JA,K)
      ENDIF
 200  CONTINUE
      ISTAN = ISEQ(JA,IYY) + IIPP + IYPP
      KSTAN = ISEQ(JA,IYY) + IMMA + JMMA
C     HYP corresponds to less hydrophobic HIP to more hydrophobic
      IF(ISTAN .GT. 0) HYP = FLOAT(IYPP)/FLOAT(ISTAN)
      IF(ISTAN .GT. 0) HIP = FLOAT(IIPP)/FLOAT(ISTAN)
      IF(KSTAN .GT. 0) VIP = FLOAT(IMMA)/FLOAT(KSTAN)
      IF(KSTAN .GT. 0) FIP = FLOAT(JMMA)/FLOAT(KSTAN)
      IAHP  = IAHP + IYPP
      IAHM  = IAHM + IIPP
      IAMP  = IAMP + IMMA
      IAMM  = IAMM + JMMA
      ISTOT = ISTOT + ISTAN
      KSTOT = KSTOT + KSTAN
      AHYDP = AHYDP + HYP
      AHYDM = AHYDM + HIP
      AMASP = AMASP + VIP
      AMASM = AMASM + FIP
      FUPS = FLOAT(IUPS)/20.0
C     -----------------------------------------------------------
      write(9,66) J, IYY, AMINOS(IYY), ISTAN, IYPP, IIPP, HIP, HYP,
     e            KSTAN, IMMA, JMMA, FIP, VIP, FUPS
      write(10,67) J, -HYP, HIP
 69   Format(I10,2X,F10.4)
      HHY = HYP - HIP
      write(14,6771) J, -HYP, HIP, HHY
 6771 format(I5,3F12.4)
      VVF = VIP - FIP
      write(11,67) J, -VIP, FIP
 67   FORMAT(I5,2F12.4)
      write(15,6771) J, -VIP, FIP, VVF
      If(J .ge. IM) goto 1250
 9000 Continue
C     ============================================================
 1250 WRITE(2,900) IAHP, IAHM, AHYDP-AHYDM, IAMP, IAMM, AMASP-AMASM,
     r             ISTOT, KSTOT
 900  FORMAT(10X,'Volume of AAs (>-) hydrophobicity =',I5/
     r       10X,'Volume of AAs (>+) hydrophobicity =',I5/
     r       10X,'Mean fractional hydrophobicity    =',F10.4/
     r       10X,'Volume of AAs (-) bulkiness       =',I5/
     r       10X,'Volume of AAs (+) bulkiness       =',I5/
     r       10X,'Mean fractional bulkiness         =',F10.4/
     r       10X,'Total AAs in hydrophobicity       =',I7/
     r       10X,'Total AAs in bulkiness            =',I7/)
 66   format(I4,1X,I3,1X,A1,1X,3I4,1X,2F6.3,3I4,1X,3F6.3)
      Do J=1,IM
      Write(620,879) J, JTOT(J)
 879  format('33',2I8)
      enddo

C     <<<<<<<<<<<<<<<<<<<<<<<<<<<<<<<<<<<<<<<<<<<<<<<<<<<<<
C     Entropy computing
C     |||||||||||||||||||||||||||||||||||||||||||||||||||||
      LM = 0
      Jhit = 0
      Jhh = 0
```

```
      Do J=1,JGH
      Star(J) = ' '
      Seqq(J) = ' '
      Enddo
      IH = 0
      Jpp = 0
      Write(21,210) Job, Filnam
      If(Job .ge. 2) Then
      Write(56,210) Job, Filnam
      Endif

      If(Job .ge. 2) Write(560,210) Job, Filnam
      Write(22,210) Job, Filnam
 210  format('##### This is Job-step',I5,' fro file: ',A)
C
 500  WRITE(2,2) (AMINOS(I),I=1,21), (Fogg(L),L=1,5)
 2    FORMAT(4X,21(3X,A1),3X,3(1X,A3),2(3X,A3))

      DO J=1+IH,36+IH
      Jak = J
      Bagg = Float(JTOT(J))
      Sip = 0.0
      Sax = 0.0

      Do M=1,8
      Ipop(M) = 0
      Enddo

      Do M=1,20
      If(M .eq. 1 .OR. M .eq. 8 .OR. M .eq. 10 .OR. M .eq. 11
     #   .OR. M .eq. 18) Ipop(1) = Ipop(1) + ISEQ(J,M)
      If(M .eq. 13) Ipop(2) = Ipop(2) + ISEQ(J,M)
      If(M .eq. 6) Ipop(3) = Ipop(3) + ISEQ(J,M)
      If(M .eq. 2) Ipop(4) = Ipop(4) + ISEQ(J,M)
      If(M .EQ. 7 .OR. M .EQ. 9 .OR. M .EQ. 15)
     # Ipop(5) = Ipop(5) + ISEQ(J,M)
      If(M .eq. 3 .OR. M .EQ. 4 .OR. M .EQ. 12 .OR. M .EQ. 14)
     #  Ipop(6) = Ipop(6) + ISEQ(J,M)
      If(M .eq. 16 .OR. M .eq. 17) Ipop(7) = Ipop(7) + ISEQ(J,M)
      If(M .EQ. 5 .OR. M .GE. 19) Ipop(8) = Ipop(8) + ISEQ(J,M)
      Enddo

C     Simpson diversity population

      Divers = 0.0
      Bagicz = Bagg*(Bagg-1.0)
      Stagic = 0.0

      Do M = 1,8
      Stagic = Stagic + Float(Ipop(M)*(Ipop(M)-1))
      enddo

      If(Bagicz .gt. 0.0) Divers = 1.0 - Stagic/Bagicz
      write(577,577) (Ipop(Lmm),Lmm=1,8), JTOT(J), Bagg, Bagicz, Stagic,
     #  Divers
 577  format(8I4,2X,I5,2X,4F10.3)

      Do K=1,20
```

```
      Pagg = Float(ISEQ(Jak,K))
      If(Bagg .gt. 0.0) Fagg = Pagg/Bagg
      If(Bagg .gt. 0.0 .AND. Pagg .gt. 0.0) Then
      Sip = Sip + Fagg*Aln2*Alog(Fagg)
      Sax = Sax + Fagg*Aln20*Alog(Fagg)
      Endif
      enddo

      Sopa = Abs(Sip)
      Saxa = Abs(Sax)
      If(Sopa .gt. 0.0  .and. Sopa .le. 0.25) STR(J) = '+'
      If(Sopa .gt. 0.25 .and. Sopa .le. 0.5) STR(J) = '='
      If(Sopa .gt. 0.5  .and. Sopa .le. 1.0) STR(J) = '|'
      If(Saxa .gt. 0.0  .and. Saxa .le. 0.062) SYY(J) = '+'
      If(Saxa .gt. 0.062 .and. Saxa .le. 0.125) SYY(J) = '='
      If(Saxa .gt. 0.125 .and. Saxa .le. 0.25) SYY(J) = '|'
C     >>>>>>>>>>>>>>>>>>>>>>>>>>>>>>>>>>>>>>>>>>>>>>>>>>>>>>>>>
      WRITE(2,3) J, (ISEQ(J,I),I=1,21), JTOT(J), IBE(J), ABE(J),
     t            STR(J), FTOT(J), -Sip
      Write(4,33) J, JTOT(Jak)
 33   format(2I5)
 3    FORMAT(I4,21(1X,I3),3X,I3,1X,I3,2X,2A1,2X,F5.3,2X,F5.3)
      write(156,769) SEQ(1,J), Jhit, Jhh, Jhit +Jhh, Job
 769  format(2X,A1,2X,'Jhit=',I4,2X,3I4)
C
C     Attention HERE SEQ(1,J) correspond to leading (top) entry in given MSA
C
      If(SEQ(1,J) .ne. '-') then
      Jhit = Jhit + 1
      Jpp = Jpp + 1

 543  If(SeqRef(Jpp,Job-1) .eq. '-') Then
      Jpp = Jpp + 1
      goto 543
      Endif

      Star(Jhit) = STR(Jak)
      Seqq(Jhit) = SEQ(1,Jak)
      If(Jshiff .gt. 0) Then
      San(Jhit,Jshiff+Jff) = Seqq(Jhit)
      San(Jhit,Jshiff+Jff+1) = Star(Jhit)
C      Hot(Jhit+Jhh,Jshiff+Jff) = Saxa
      Hot(Jhit+Jhh,Jshiff) = Saxa
      write(560,56) Job,J,Jhit,Seqq(Jhit),SeqRef(Jpp,Job-1),Saxa
C     TEMPORARY BLOCK
       write(56,56) Job,J,Jhit,Seqq(Jhit),SeqRef(Jpp,Job-1),Saxa, Sopa,
     # Divers
        write(6,56)
     #   Job,J,Jhit,Seqq(Jhit),SeqRef(Jpp,Job-1),Saxa, Sopa, Divers
 56   Format(3I5,1X,2(1X,A1),2X,3F6.3)
      Endif

      write(21,338) Float(J-IGF+1), Float(Jhit), JTOT(J),
     #              -Sip, -Sax, SEQ(1,Jak), STR(Jak)
      write(211,338) Float(J-IGF+1), Float(Jhit), JTOT(J),
     #              -Sip, -Sax, SEQ(1,Jak), STR(Jak)
      write(22,337) Float(Jhit), -Sax
      Endif
```

```
C
C     Ending entropy for 'long' sequence
C     Below if thereis a gap
C
      If(SEQ(1,J) .eq. '-') then
      write(156,8711)
 8711 format('we arein job -------')
      Saxon = -1.0
      Jhh = Jhh + 1
      Hot(Jhit+Jhh,Jshiff) = Saxon
      write(211,338) Float(J-IGF+1), Float(Jhit+Jhh), JTOT(J),
     #              -Sip, -Sax, SEQ(1,Jak), STR(Jak)
      Endif


 337  format(2F10.3)
 338  format(2F10.3,2X,I4,1X,F10.5,3X,F5.2,2X,2A1)
      LM = LM + 1
      If(LM .ge. JGH) goto 1350
      ENDDO

      IH = IH + 36
      IF(LM .LE. IM+1) GOTO 500
C     ---------------------------
C     Make special output of TIPS
 1350 Write(6,671) Job, LM
  671 format('In job made steps',2I8)
      If(Job .eq. 1) Jhitmax = LM

      JJx = 0
      KKx = 70
      KKP = Jhit/70
      KKV = 0
      Do K=1,KKP
      KKV = KKV + 70
      Enddo
      KKC = Jhit - KKV
      write(6,876) Jhit, KKP, KKC, KKV, JGH
 876  format('Jhit',I5,' KPP',I5,' KKC',I5,' KKV',I5,' JGH',I5)
      Write(24,21) Job, Filnam
 21   Format(/10X,'Passing Job',I5,' from ',1X,A//)
C
 700  Write(24,24) (Seqq(L),L=1+JJx,KKx+JJx)
      Write(24,24) (Star(J),J=1+JJx,KKx+JJx)
      Write(24,244)
 244  Format(/)
      JJx = JJx + 70
 24   format(5X,70A1)
      If(JJx .lt. KKV) goto 700
      JJx = JJx - 70
      Write(24,24) (Seqq(L),L=KKV+1,KKV+KKC)
      Write(24,24) (Star(J),J=KKV+1,KKV+KKC)
      Write(24,245)
 245  Format(/)

      Jstar = 0
      Jplus = 0
      Jequa = 0
```

```
      Jvert = 0
      Stt = 0.0
      Plu = 0.0
      Equ = 0.0
      Ver = 0.0
      Do J = 1,Jhit
      If(Star(J) .eq. '*') Jstar=Jstar+1
      If(Star(J) .eq. '+') Jplus=Jplus+1
      If(Star(J) .eq. '=') Jequa=Jequa+1
      If(Star(J) .eq. '|') Jvert=Jvert+1
      enddo
      If(Jhit .gt. 0) Then
      pp = Float(Jhit)
      Stt = Float(Jstar)/pp
      If(Jplus .gt. 0) Plu = Float(Jplus)/pp
      If(Jequa .gt. 0) Equ = Float(Jequa)/pp
      Ver = Float(Jvert)/pp
      Endif
      Top = 1.0 - (Stt+Plu+Equ+Ver)
      If(Job .eq. 1) write(6,6688) Job, Stt, Plu, Equ, Ver, Top
 6688 format('Job etc',I5,5F10.3)
C23456789012345678901234567890123456789012345678901234567890123456789012345678
90
      Write(24,295) NLINE,Jhit,Jstar,Stt,Jplus,Plu,Jequa,Equ,
     #              Jvert,Ver,Top
 295  Format('Seq-Resi',I4,'|',I3,1X,'///','  *  ',I3,1X,F4.3,
     # 2X,' / + ',I3,1X,F4.3,' / = ',I3,1X,F4.3,' / | ',I3,1X,
     # F4.3,2X,'///','  Rest = ',F4.3)
      Write(24,285)
 285  Format('||----------|----------|----------|----------|----------|
     #----------|----------|----------||'/)
      Nlarge = Nlarge + NLINE
C     Make small-letters sequence
      Do J=1,IM
      REQ(1,J) = SEQ(1,J)
      Enddo
C     ++++++++++++
      Do K=2,NLINE
      Kot = K
      Do J=1,IM
      AB = SEQ(Kot,J)
      If(AB .eq. SEQ(1,J)) REQ(Kot,J) = SEQ(Kot,J)
      If(AB .ne. SEQ(1,J)) then
      Do M=1,22
      IF(AB .eq. AMINOS(M)) Then
      REQ(Kot,J) = Aminss(M)
      goto 50
      endif
 50   enddo
      EndIF
      Enddo
      Enddo

C     Insert recent

      Do J = 1,IM
      REQ(NLINE+1,J) = SEQ(NLINE+1,J)
      Enddo
```

```
      Nqq = IM/60
      Kb = 1
      Kc = 60
      N1 = 0
      L = 0
C|||||||||||||||||||||||||||||||||||||||||||||||||||
      Write(55,31)
 31   Format(/)
C|||||||||||||||||||||||||||||||||||||||||||||||||||
      Write(55,32) Jstep, Buff(1)
 40   Write(55,30) Buff(2)
 32   Format('Step no:',I5,2X,A)
 30   Format(A)
      Do J=1,NLINE+1
      write(55,35) CODEX(J), (REQ(J,M),M=Kb,Kc)
      enddo
 35   format(A,60A1)
      N1 = N1 + 1
      If(N1 .lt. Nqq) Then
      Kb = Kb + 60
      Kc = Kc + 60
      elseIf(N1 .eq. Nqq) Then
      Imma = IM - Kc
      Kb = Kb + 60
      Kc = Kc + Imma
      Endif
      IF(N1 .lt. NPAGE) goto 40

      Job = Job + 1
      Do J=1,IM
      JTOT(J) = 0
      IBE(J) = 0
      ABE(J) = ' '
      Enddo
      Jshiff = Jshiff + 1
      If(Jshiff .ge. 2) Jff = Jff + 1
      If(Job .gt. 2) Then
      Write(67,777) Jhit
      Jhut(Job) = Jhit
      Endif
 777  Format(I10)
C
C     Cleaning SEQ(+,+)
C
      Write(88,7651) NLINE, Kat
 7651 format('Attention NLINE-Kac cleaning', I5,2X,I6)
      Do J = 1,NLINE
      Do K = 1,Kat
      SEQ(J,K) = ' '
      Enddo
      Enddo
      IF(Job .le. Ncase) goto 9800
C
C     Returning point for Job :: GOTO 9800
C
      Klotz = 0
      Do J = 1,Jhit
      If(SeqRef(J,1) .eq. '-') Klotz = Klotz + 1
```

```
      Enddo
C     Set-up matrix
      Jhit = Jhit + Klotz

      Write(6,679) Jshiff, Jhit, Klotz
 679  format(10X,'Jshiff - Jhit - There are skipped positions',3I4)

      Do M = 1,Jhit
      Do K = 1,2*Jshiff
      Tan(M,K) = ' '
      Enddo
      Enddo
C
      Kola = 2*Jshiff - 2
      Kolb = Jshiff - 1

C     Kolb === Number of Job - the first MSA-matrix

      write(6,54) Kolb, Jhit, Jhitmax
 54   format('Width-Length of unit=18,',I5,2x,2I4)
C
      Do K=1,Jhitmax
      write(18,675) K, (SeqRef(K,L),L=1,Kolb)
 675  Format(5X,I3,2X,30(1X,A1))
      Enddo
c      Do K = 1,Jhitmax+Jhh
      Do K = 1,Jhitmax
      AB = SEQ(1,K)
      write(182,677) K, AB, (Hot(K,L),L=1,Kolb)
 677  Format(2X,I3,1X,A1,1X,2X,30F5.2)
      Enddo
C
      Ku = 0
      Kg = 0
      Do M = 1,Kolb
      MM = M
      Do 6900 K = 1,Jhitmax
      If(SeqRef(K,MM) .eq. '-') Then
      Tan(K,MM+Ku) = '-'
      Tan(K,MM+Ku+1) = ' '
      Kg = Kg + 1
      Goto 6900
      Endif
      Tan(K,MM+Ku) = San(K-Kg,MM+Ku)
      Tan(K,MM+Ku+1) = San(K-Kg,MM+Ku+1)
 6900 Continue
      Kg = 0
      Ku = Ku + 1
      Enddo

      Do K=1,Jhitmax
      Write(17,685) (San(K,L),L=1,Kola)
      enddo
      Do K=1,Jhitmax
      Write(19,685) K, (Tan(K,L),L=1,Kola)
 685  Format(9X,I3,2X,40(2A1,3X))
      enddo
C
```

```
      Write(24,2440) Nlarge - Ncase + 1
 2440 Format(/10X'The total number of analyzed sequences',I5)
      CLOSE(2)
      CLOSE(9)
      CLOSE(10)
      CLOSE(11)
      Rewind(21)
      Jks = 0
      Jjj = 0

 5500 Read(21,1,Err=8230,End=8230) Line

      IF(Line(1:1) .eq. '#') Then
      Joh(Jks+1) = Jjj
      Jks = Jks + 1
      Jjj = 0
      goto 5500
      Endif

      Jjj = Jjj + 1
      Apos(Jks,Jjj) = Line(41:45)
      Pats(Jks,Jjj) = Line(48:48)
      goto 5500
 8230 Write(6,641) Jks
 641  Format('Jks='I6)

      do J = 1,Jks
      write(6,641) Joh(J)
      Enddo

      Do K=1,Jhitmax
      If(SeqRef(K,M) .eq. '-') Panna(K,M) = '-'
      write(181,6751) K, Apos(2,K), (SeqRef(K,L),L=1,Kolb)
 6751 Format(5X,I3,2X,A5,2X,30(1X,A1))
      Enddo
C
C     Ending phase with heatmap calculation
C
      Rewind(67)
      Rewind(56)
      Close(56)
      Close(67)

      Jx = 1

      Open(Unit=67,status='old')
      Open(Unit=56,status='old')

      Do J = 1,30
      Write(6,734) Jhut(J)
 734  Format('Jhut',I6)
      enddo

      Do Jm = 1,Ncase-1

      Read(67,*) Ipost
      Write(6,673) Ipost
 673  format('Ipost=',I5)
```

```
      Read(56,1) LINE
      Write(6,1) LINE
      Kost = Ipost

      Do L = 1,Kost
      Read(56,56) Job,J,Jhit,AB,AB,Saxa,Sopa,Divers
      write(6,56) Job,J,Jhit,AB,AB,Saxa,Sopa,Divers
      Saxyy(L) = Saxa
      Saxzz(L) = Sopa
      DiSeQQ(L) = Divers
      Enddo
1300  Continue
      Ican = 0
      Jcan = 1
      Do M=1,Kac
      Ican = Ican + 1

      If(SeqRef(M,Jx) .ne. '-') Then
      Sunn(Ican,Jx) = Saxyy(Jcan)
      Tuna(Ican,Jx) = Saxzz(Jcan)
      Bunn(Ican,Jx) = DiSeQQ(Jcan)
      Jcan = Jcan + 1
      Elseif (SeqRef(M,Jx) .eq. '-') Then
      Sunn(Ican,Jx) = -3.00
      Tuna(Ican,Jx) = -5.00
      Bunn(Ican,Jx) = -6.00
      Endif
      Enddo
      write(677,988) Jx
988   format('Jx=',I6)
      Jx = Jx+ 1
      Enddo

      write(6,7810) Jx-1
7810   format('Jx=',I4)

      Open(Unit=72,file='Names.dat',status='old')
      Read(72,1) Line
      Write(71,1) Line
      Do J=1,Jx-1
      Read(72,2450) Kl, Intr(J)
2450  Format(I3,A5)
      Enddo
      Write(71,2460) (Intr(J),J=1,Jx-1)
      Write(73,2460) (Intr(J),J=1,Jx-1)
2460  Format(8X,20(A5))

      Do L = 1,Jx-1
      Do M = 1,6
      Ioto(L,M) = 0
      Enddo
      Enddo

      Do L=1,Kac
      Kot = 0
      Min = 0

      Do I = 1,Jx-1
```

```
      Assa(I) = ' '
      Enddo
C
      Do J=1,Jx-1
      If(Sunn(L,J) .gt. 0.5) Then
      Assa(J) = ' '
      Ioto(1,J) = Ioto(1,J) + 1
      Endif
      If(Sunn(L,J) .gt. 0.25 .AND. Sunn(L,J) .le. 0.5)Then
      Assa(J) = ' '
      Ioto(2,J) = Ioto(2,J) + 1
      Endif
      If(Sunn(L,J) .gt. 0.125 .AND. Sunn(L,J) .le. 0.25)Then
      Assa(J) = '!'
      Ioto(3,J) = Ioto(3,J) + 1
      Endif
      If(Sunn(L,J) .gt. 0.065 .AND. Sunn(L,J) .le. 0.125)Then
      Assa(J) = '|'
      Ioto(4,J) = Ioto(4,J) + 1
      Endif
      If(Sunn(L,J) .GE. 0.0 .AND. Sunn(L,J) .LE. 0.065) Then
      Ioto(5,J) = Ioto(5,J) + 1
      Assa(J) = '*'
      Endif
      If(Sunn(L,J) .Eq. -1.000) Then
      Assa(J) = '-'
      Ioto(6,J) = Ioto(6,J) + 1
      Endif
      Enddo

      write(71,233) L, (SeqRef(L,J),Assa(J),J=1,Jx-1)
 233  format(I4,5X,25(2X,2A1,1X))
      write(73,234) L, (Assa(J),J=1,Jx-1)
 234  format(I4,3X,25(1X,A1,2X))
      Do K=Jx-1
      Write(6,631) L, (Sunn(L,K),K=1,Jx-1)
      Write(61,631) L, (Sunn(L,K),K=1,Jx-1)
      Write(62,631) L, (Tuna(L,K),K=1,Jx-1)
      Write(63,631) L, (Bunn(L,K),K=1,Jx-1)
 631  Format(I5,1X,25F7.3)
      enddo
      Write(73,2490) (Intr(J),J=1,Jx-1)
 2490 Format(/1X,'MMPs',20A5)
      Write(73,2495)
C1234567890123456789012345678901234567890123456789012345678901234567890
 2495 Format('-----------------------------------------------------------
     #---------------------------------------------')
     Do I=1,5
      Write(73,749) (Ioto(I,J),J=1,Jx-1)
 749  Format(5X,30I4)
      Enddo
      STOP
      END
C------------------------------------------------------------C
C     A Fortran-77 program to calculate pI from sequence   C
C     See S. Skoog and A Wichman – Trends in Analytical    C

C     Chemistry Vol. 5, pp. 82-83, 1986 C                  C
```

```
C-----------------------------------------------------------C
      SUBROUTINE PICAL(Kob,NNN,ITD,PIC,TSEQ)
      EXTERNAL FUNC
      REAL MINIF
      Real zbrent
      CHARACTER*1 TSEQ(500)
      CHARACTER*1 AMINOS(22)
      COMMON/PIPIA/ IC,ID,IE,IH,IK,IR,IY, PI(9)
      DIMENSION CTERM(20), CNTERM(20)
C               R    K    Y   C   H   D   E  NT  CT
      DATA PI/12.5,10.5,10.1,8.3,6.0,3.9,4.3,0.0,0.0/
      DATA CTERM/2.4,2.2,2.1,2.1,1.7,2.2,2.2,2.4,1.8,2.3,
     "           2.3,2.2,2.1,2.2,2.0,2.2,2.1,2.4,2.2,2.3/
      DATA CNTERM/9.9,9.0,8.8,9.8,10.8,9.1,9.7,9.8,9.2,9.8,
     x            9.7,9.0,9.3,9.2,10.6,9.2,9.1,9.4,9.1,9.7/
      DATA AMINOS/'A','R','N','D','C','Q','E','G','H','I','L','K',
     +            'M','F','P','S','T','W','Y','V','Z','X'/
C|||||||||||||||||||||||||||||||||||||||||||||||||||||||||||||||||
C     Preset starting parameters
      write(889,78) Kob, NNN, ITD
 78   format('Step - NLINE - ITD',3I6)
      write(889,67) (TSEQ(I),I=1,ITD)
 67   format(60A1)
      If(ITD .eq. 0) goto 99
      KOUNT = ITD
      KL  = 0
      KM  = 0
      PH1 = 1.0
      PH2 = 14.0
C    Calculate the integer number of charged AA
      IR = 0
      IK = 0
      IH = 0
      IC = 0
      ID = 0
      IE = 0
      IY = 0
      DO J = 1,KOUNT
      IF(TSEQ(J) .EQ. 'R') IR = IR + 1
      IF(TSEQ(J) .EQ. 'K') IK = IK + 1
      IF(TSEQ(J) .EQ. 'H') IH = IH + 1
      IF(TSEQ(J) .EQ. 'C') IC = IC + 1
      IF(TSEQ(J) .EQ. 'D') ID = ID + 1
      IF(TSEQ(J) .EQ. 'E') IE = IE + 1
      IF(TSEQ(J) .EQ. 'Y') IY = IY + 1
      ENDDO
C     Find the N- and C-terminal AA
      N9 = 0
      K9 = 0
      DO J = 1,20
      IF(TSEQ(1) .EQ. AMINOS(J)) N9 = J
      IF(TSEQ(KOUNT) .EQ. AMINOS(J)) K9 = J
      ENDDO
      PI(8) = CNTERM(N9)
      PI(9) = CTERM(K9)
C     Decison made search for pI
c     PIC = MINIF(FUNC,PH1,PH2,XACC,MAXEL)
c     PIC = rtsec(func,PH1,PH2,xacc,MAXEL)
```

```
      PIC = zbrent(FUNC,PH1,PH2,XACC,MAXEL)
 99   RETURN
      END
C--------------------------------------------C
C     Find charge on the protein             C
C     Author: A. Galat, July 1995            C
C--------------------------------------------C
      FUNCTION FUNC(PH)
C
      COMMON/PIPIA/ IC,ID,IE,IH,IK,IR,IY,PI(9)
      SUM = 0.0
      SUM = SUM - ID*PNCHARGE(6,PH,PI)
      SUM = SUM - IE*PNCHARGE(7,PH,PI)
      SUM = SUM - IC*PNCHARGE(4,PH,PI)
      SUM = SUM - IY*PNCHARGE(3,PH,PI)
      SUM = SUM + IH*PCHARGE(5,PH,PI)
      SUM = SUM + IK*PCHARGE(2,PH,PI)
      SUM = SUM + IR*PCHARGE(1,PH,PI)
      TEM = PCHARGE(8,PH,PI)
      SUM = SUM + TEM
      TEM = PNCHARGE(9,PH,PI)
      SUM = SUM - TEM
      FUNC = SUM
      RETURN
      END
      FUNCTION PCHARGE(N,PH,PI)
      DIMENSION PI(100)
      ALFA = EXP(2.3026*(PH-PI(N)))
      PCHARGE = 1.0/(1.0 + ALFA)
      RETURN
      END
      FUNCTION PNCHARGE(N,PH,PI)
      DIMENSION PI(100)
      ALFA = EXP(2.3026*(PH-PI(N)))
      PNCHARGE = ALFA/(1.0 + ALFA)
      RETURN
      END
C||||||||||||||||||||||||||||||||||||||||||||||||||||||||||||
C------------------------------------------------------------C
C     The Brent's method of finding the root of a function  C
C     FUNC known to lie between X1 and X2                    C
C     Buy and copy it from the book by W.H. Press,           C

C     B.P. Flannery, S.A. Teukolsky and W.T. Vetterling      C

C     Numerical Recipes - The Art of Scientific Computing    C

C     Cambridge University Press, 1996.                      C

C------------------------------------------------------------C
      FUNCTION zbrent(FUNC,X1,X2,TOL,MAXEL)
      PARAMETER (ITMAX=100,EPS=3.E-8)
      COMMON IC,ID,IE,IH,IK,IR,IY, PI(9)
```
