# Supplementary material for: Compression of Large Sets of Sequence Data Reveals Fine Diversification of Functional Profiles in Multigene Families of Proteins: A Study for Peptidyl-Prolyl cis/trans Isomerases (PPIase)
Source: Biomolecules. 2019 Feb 11;9(2):59. doi: 10.3390/biom9020059 (PMC6406343; doi:10.3390/biom9020059)
Supplement: Supplementary file 1 [file biomolecules-09-00059-s001.zip › Figure S4-FKBPs_Simpson.pdf]

	Fk1a	Fk1b	Fk3	Fk4a	Fk4b	Fk5a	Fk5b	Fk6	Fk8	Fk2	Fk7	Fk11	Fk14	Fk9a	Fk9b	Fk9c	Fk9d	Fk10a	Fk10b	Fk10c	Fk10d
1	0,328	0,198	0,034	0,109	0	0	0	0,386	0,006	0,162	0,171	0,388	0,266	0,684	0,027	0,368	0,175	0,036	0,535	0,69	0,165
2	0,076	0,135	0,007	0,088	0,44	0,402	0,061	0,04	0,26	0	0,08	0,057	0,051	0,054	0,052	0	0,213	0	0,669	0,026	0,019
3	0,577	0,503	0,281	0,208	0,011	0	0	0,409	0,048	0,054	0,014	0,13	0,036	0,054	0,499	0,222	0,49	0	0,517	0,107	0,525
4	0,221	0,211	0,007	0,202	0,541	0	0	0,028	0,045	0	0,02	0,133	0,007	0,116	0,02	0,201	0,085	0,015	0	0,701	0,468
5	0,285	0,358	0,246	0,589	0,367	0	0,131	0,506	0,498	0,015	0,086	0,175	0	0,521	0,013	0,513	0,182	0,058	0,517	0,49	0,424
6	0,61	0,611	0,041	0	0,571	0	0,007	0,134	0,017	0,26	0,407	0,154	0,512	0,228	0,276	0,618	0,495	0,688	0,508	0,314	0,676
7	0,134	0,208	0,028	0,515	0,722	0,148	0,511	0,348	0,611	0,088	0,445	0,599	0,007	0,247	0,289	0	0,639	0,029	0,56	0,02	0,111
8	0,618	0,583	0,055	0,225	0,602	0,703	0,007	0,477	0,676	0,372	0,033	0,16	0,007	0,032	0,007	0	0,157	0,007	0,503	0,02	0,032
9	0,489	0,45	0,055	0,156	0,167	0	0	0,655	0,183	0,194	0,153	0,266	0,097	0,192	0,631	0,139	0,006	0,153	0,512	0,697	0,628
10	0,1	0,199	0,007	0,016	0,312	0,64	0	0,082	0,669	0,621	0,305	0,152	0,021	0,429	0,648	0,615	0,013	0,355	0,502	0,662	0,164
11	0,114	0,222	0,048	0,541	-----	0,489	-----	0,676	0,742	-----	-----	0,641	-----	-----	-----	-----	-----	-----	-----	-----	0,779
12	0,207	0,239	0,068	0,118	0,419	0,384	0	0,198	0,766	0,005	0,04	0,106	0	0,083	0	0	0,663	0	0,65	0	0
13	0,719	0,662	0,211	0,528	0,36	0,09	0	-----	0,71	0,549	0,201	0,589	0,236	0,554	0,513	0,344	0,14	0,518	0,707	0,68	0,063
14	0,413	0,282	0,158	0,261	0,643	0,6	0,145	0,563	0,475	0,259	0,559	0,533	0,007	0,042	0	0	0	0,007	0,78	0	0,547
15	0,106	0,077	0,075	0,587	0,573	0	0,177	0,593	0,109	0,073	0,502	0,73	0,014	0,558	0,323	0,28	0,29	0,029	0,374	0,033	0,045
16	0,083	0,048	0	0,038	0,018	0	0,007	0,158	0,039	0,136	0,12	0,503	0,021	0,083	0,065	0,006	0,322	0,043	0,686	0,457	0,351
17	0,27	0,178	0,068	0,547	0,272	0	0,007	0,658	0,72	0,144	0,052	0,602	0,007	0,198	0,007	0,312	0,282	0,304	0,695	0,091	0,548
18	0,682	0,632	0	0,629	0,06	0	0,007	0,594	0,409	0,13	0,026	0,623	0,544	0,618	0	0,435	0,085	0,532	0,645	0,497	0,629
19	0,08	0,09	0,014	0,029	0,231	0	0,014	0,399	0,285	0,01	0,026	0,086	0	0,103	0	0	0,025	0,19	0,662	0	0,642
20	0,167	0,09	0,041	0,527	0,28	0	0,1	0,505	0,056	0	0,039	0,095	0,014	0,062	0	0	0,013	0,014	0,705	0	0
21	0,585	0,423	0,577	0,424	0,516	0	0,217	0,49	0,547	0,454	0,026	0,157	0,007	0,083	0	0	0,013	0,095	0,607	0	0,407
22	0,312	0,465	0	0	0,046	0,407	0	0,671	0,239	0	0,026	0,086	0	0,072	0	0	0	0	0	0	0,013
23	0,592	0,475	0,428	0,682	0,43	0	0,191	0,081	0,344	0,503	0,052	0,522	0	0,082	0	0	0,006	0	0	0	0,285
24	0,101	0,057	0,087	0,004	0,011	0	0	0,061	0,003	0,015	0,046	0,111	0	0,093	0	0,013	0	0,007	0,007	0	0
25	0,057	0,039	0,061	0,007	0,738	0	0,127	0,166	0,526	0,015	0,064	0,162	0	0,073	0	0	0	0,021	0,021	0,013	0,044
26	0,054	0,039	0,021	0,004	0,346	0,435	0,061	0,011	0,263	0,01	0,064	0,207	0,258	0,093	0,02	0	0	0,014	0,014	0	0
27	0,53	0,446	0,061	0,415	0,601	0	0,101	0,386	0,663	0,239	0,057	0,216	0,035	0,424	0	0	0,006	0,028	0,028	0,007	0
28	0,072	0,087	0,007	0,004	0,074	0,124	0,014	0,187	0,685	0,005	0,094	0,016	0,028	0,083	0	0,031	0	0,252	0,252	0,033	0,006
29	0,333	0,502	0,458	0,611	0,71	0	0,636	0,114	0,651	0,47	0,063	0,484	0	0,47	0	0	0	0,021	0,021	0,013	0
30	0,053	0,035	0,054	0,025	0,71	0,5	0,259	0,296	0,034	0,005	0,051	0	0,183	0,119	0,335	0,006	0,05	0,062	0,062	0,428	0
31	0,599	0,486	0,157	0,249	0,673	0,206	0,285	0,131	0,347	0,376	0,094	0,36	0,028	0,267	0,013	0	0	0,689	0,689	0,256	0,006
32	0,087	0,09	-----	0,014	0,499	0	0,404	0,659	0,602	-----	0,456	-----	0,375	-----	-----	-----	-----	-----	-----	-----	-----
33	0,026	0,031	0,007	0,018	-----	0,131	-----	0,719	0,18	0,037	0,095	0,046	0	0,15	0	0	0,006	0,028	0,028	0,007	0,013
34	0,569	0,488	0,014	0,117	-----	0,024	-----	0,186	0,258	0,01	0,124	0	0,007	0,121	0	0	0,019	0,014	0,014	0,007	0,013
35	0,224	0,122	0,1	0,389	0,506	0,008	0,381	0,598	0,531	0,088	0,122	0,154	0,007	0,656	0	0	0	0,201	0,201	0,335	0,032
36	-----	-----	0	-----	0,08	-----	0,492	0,123	0,031	0,327	0,349	0,032	0,08	0,158	0,007	0,505	0,013	0,343	0,343	0,447	0,537
37	0,072	0,072	0	0,007	0,063	0,008	0	0,014	0,042	0,01	0,112	0,248	0,027	0,11	0	0	0	0,1	0,1	0,026	0,006
38	0,009	0,009	0	0	0,094	0,179	0	0,283	0,418	0	0,037	0,016	0,148	0,119	0	0	0,352	0,041	0,041	0,039	0,013
39	0,014	0,026	0,014	0,025	0,471	0,016	0,321	0,029	0,331	0,059	0,139	0,032	0,041	0,163	0	0	0,006	0,081	0,081	0,007	0,006
40	0,003	0,018	0,014	0,018	-----	0,316	-----	0,513	-----	0,01	0,012	0,024	0	0,087	0	0	0,145	0,014	0,014	0,007	0,006
41	0,117	0,026	0,055	0,508	-----	0,016	-----	0,779	-----	0,334	0,006	0,055	0,099	0	0,007	0	0,019	0,338	0,338	0,013	0,337
42	0,138	0,068	0,467	0,233	-----	0,016	-----	0,724	-----	0,556	0,036	0,138	0,328	0,009	0,318	0,062	0,013	0,02	0,02	0,452	0,146
43	0,014	0	0,355	0,025	-----	0,438	-----	0,581	-----	0,458	0,12	0,071	0,344	0	0	0,019	0,593	0,014	0,014	0,033	0,094
44	0,513	0,507	0,355	0,647	-----	0,125	-----	0,424	-----	0,379	0,286	-----	0,393	0,537	0,007	0,025	0,53	0,604	0,604	0	0,525
45	-----	-----	0,52	-----	-----	-----	-----	-----	-----	-----	0,2	-----	0,007	-----	-----	-----	-----	-----	-----	-----	-----

46	-----	-----	0,049	-----	-----	-----	-----	-----	-----	-----	-----	-----	-----	-----	-----	-----	-----	-----	-----	-----	-----
47	-----	-----	0,042	-----	-----	-----	-----	-----	-----	-----	-----	-----	-----	-----	-----	-----	-----	-----	-----	-----	-----
48	-----	-----	0,035	-----	-----	-----	-----	-----	-----	-----	-----	-----	-----	-----	-----	-----	-----	-----	-----	-----	-----
49	-----	-----	0,022	-----	-----	-----	-----	-----	-----	-----	-----	-----	-----	-----	-----	-----	-----	-----	-----	-----	-----
50	-----	-----	0,532	-----	-----	-----	-----	-----	-----	-----	-----	-----	-----	-----	-----	-----	-----	-----	-----	-----	-----
51	-----	-----	0,352	-----	-----	-----	-----	-----	-----	-----	0	-----	0,437	-----	-----	-----	-----	-----	-----	-----	-----
52	0,645	0,539	0,148	0,245	0,039	0,154	0,014	0,713	0,62	0,317	0,047	0,055	0,093	0,018	0,013	0,038	0,329	0,621	0,621	0,573	0,738
53	0,059	0,056	0,048	0,556	0,148	0,016	0	0,295	0,583	0,107	0	0,047	0,087	0	0	0	0,044	0,454	0,454	0	0,319
54	0,011	0	0,035	0,011	0,036	0,322	0	0,555	0,214	0,228	0,207	0,063	0,061	0	0,007	0	0	0,505	0,505	0	0,274
55	0,606	0,403	0,213	0,544	0,638	0,023	0,703	0,434	0,625	0,611	0	0,306	0	0,009	0,007	0	0,006	0,171	0,171	0	0,247
56	0,45	0,23	0	0,004	0,018	0,418	0	0,287	0,38	0,04	0,024	0,071	0,067	0	0	0	0,038	0,596	0,596	0,007	0,305
57	0,7	0,471	0,035	0,336	0,518	0,023	0,327	0,264	0,583	0,161	0	0,152	0,014	0,027	0,007	0	0,006	0,395	0,395	0	0,287
58	0,009	0,018	0	0,018	0,135	0,008	0	0,099	0,04	0	0,006	0	0	0	0,007	0	0,006	0,04	0,04	0	0
59	0,014	0,004	0,007	0,097	0,053	0,046	0,007	0,158	0,083	0,005	0	0,016	0	0	0	0	0	0	0	0	0
60	0,586	0,401	0,163	0,239	0,011	0,067	0	0,562	-----	0,441	0,012	0,529	0,014	0,044	0,007	0,501	0	0,279	0,279	0,652	0,351
61	0,453	0,555	0,035	0,198	0,108	0,023	0,246	0,338	-----	0,02	0	0,078	0,483	0,391	0	0,006	0	0,047	0,047	0	0,312
62	-----	-----	-----	-----	0,448	-----	0	-----	-----	-----	-----	-----	-----	-----	-----	-----	-----	-----	-----	-----	-----
63	-----	-----	-----	-----	0,606	-----	0,014	-----	0,026	-----	-----	-----	-----	-----	-----	-----	-----	-----	-----	-----	-----
64	-----	-----	-----	-----	0,632	-----	0,258	-----	0,665	-----	-----	-----	-----	-----	-----	-----	-----	-----	-----	-----	-----
65	0,322	0,2	0,068	0,519	0,531	0	0,314	0,314	0	0,074	0,102	0,455	0	0,009	0	0,006	0,377	0,053	0,053	0,013	0,1
66	0	0	0	0	0,004	0	0	0,401	0,051	0	0	0,016	0,048	0	0	0	0,218	0,06	0,06	0	0
67	0,003	0,004	0	0,495	0,492	0	0	0,032	0,189	0,02	0	0,008	0	0	0	0	0,013	0	0	0	0
68	0,083	0,048	0,021	0,018	0,795	0,074	0,101	0,758	0,089	0,121	0,012	0,351	0,094	0,433	0,33	0,431	0	0,329	0,329	0,503	0,227
69	0,034	0,026	0,014	0,26	0,032	0,074	0	0,004	0,347	0	0	0	0	0	0	0	0	0	0	0	0
70	0,014	0,009	0	0	0,007	0	0	0,111	0,085	0,005	0	0,07	0	0	0	0	0,239	0	0	0	0
71	0,003	0	0,014	0,004	0,004	0,015	0	0,06	0,003	0,005	0	0,008	0	0	0	0	0	0	0	0	0
72	0,105	0,048	0	0,018	0,49	0,194	0,014	0,07	0,216	0,064	0,041	0,07	0,505	0,499	0,521	0,013	0	0	0	0,388	0,657
73	0,051	0,022	0,25	0,503	0,091	0	0,028	0,301	0,65	0	0,395	0,245	0	0,027	0	0,031	0	0,007	0,007	0,357	0,063
74	0,02	0,077	0	0	0,011	0,253	0	0	0,039	0	0	0,016	0	0	0,007	0	0,283	0	0	0	0,006
75	0,479	0,438	0,021	0,074	0,427	0	0,028	0,371	0,689	0,025	0,278	0,024	0,455	0,009	0,007	0	0,031	0,51	0,51	0,211	0,667
76	0,364	0,257	0,048	0,004	0,358	0	0,035	0,039	0,211	0,36	0,228	0,459	0,494	0,018	0,013	0,025	0	0	0	0	0,356
77	0	0	0	0	0,431	0,291	0	0	0,006	0,005	0	0,008	0	0	0,007	0	0,614	0	0	0,19	0
78	0,171	0,177	0,055	0,053	0,531	0,122	0,007	0,267	0,135	0,005	0	0,008	0	0	0,007	0	0,091	0,007	0,007	0	0
79	0,077	0,056	0,106	0,456	0,552	0,015	0,007	0,147	0,305	0,358	0,168	0,407	0,23	0	0,007	0,444	0	0	0	0,114	0,051
80	0,034	0,013	0,007	0,046	0,499	0	0,554	0,287	0,302	0,119	0	0,008	0	0	0	0	0	0,073	0,073	0,007	0
81	0,07	0,039	0,007	0	0	0,008	0	0	0,003	0,01	0	0,016	0,007	0	0	0	0	0,007	0,007	0	0
82	0,169	0,113	0,317	0,247	0,617	0	0,633	0,513	0,617	0,165	0	0,008	0,014	0	0,007	0	0,013	0,014	0,014	0,209	0,013
83	0,089	0,047	0,034	0,442	0,256	0,622	0,1	0,413	0,289	0,025	0	0,413	0,007	0,221	0	0	0	0,007	0,007	0,013	0,019
84	0,397	0,207	0,443	0,679	0,519	0,045	0,028	0,395	0,436	0,116	0	0,008	0,048	0,402	0,027	0,193	0,013	0,097	0,097	0,007	0,614
85	0,011	0	0	0,128	0,429	0,102	0,068	0,079	0,036	0	0	0,139	0	0	0	0	0	0,007	0,007	0	0,006
86	0,415	0,323	0,182	0,344	0,728	0,03	0,271	0,107	0,697	0,319	0,432	0,408	0,494	0,18	0,013	0,268	0,359	0,442	0,442	0,494	0,212
87	0,498	0,245	0	0,096	0,014	0,023	0	0,468	0,406	0,015	0	0	0	0	0	0	0	0	0	0	0
88	0,156	0,022	0,007	0,476	0,311	0,008	0,533	0,669	0,243	0	0	0	0	0,009	0	0	0	0,007	0,007	0	0
89	0,334	0,174	0,198	0,221	0,455	0,045	0,068	0,19	0,64	0,527	0,119	0,525	0,047	0,086	0	0	0	0,007	0,007	0	0,006
90	0,086	0,013	0,007	0,137	0,639	0	0,164	0,635	0,272	0,286	0,194	0,147	0,385	0,434	0,254	0	0	0,053	0,053	0	0,006
91	0,15	0,491	0,021	0,025	0,08	0,023	0	0,221	0,036	0,005	0,501	0,016	0,007	0	0	0	0	0,007	0,007	0	0,192

92	0,25	0,146	0,048	0,08	0,512	0	0,021	0,089	0,606	0,217	0,018	0,076	0,479	0,078	0,331	0,247	0	0,447	0,447	0,089	0,5
93	-----	-----	-----	-----	-----	-----	-----	-----	-----	-----	-----	-----	-----	-----	-----	-----	-----	-----	-----	-----	-----
94	0	0,004	0,007	0	0,011	0	0,007	0,004	0,006	0,005	0	0,016	0	0	0,007	0,006	0,05	0,02	0,02	0	0,013
95	0	0	0,007	0,014	0,314	0,303	0	0,186	0,028	0	0	0	0	0	0	0	0,108	0	0	0	0,006
96	0,711	0,632	0,048	0,684	0,612	0,141	0,366	0,735	0,744	0,243	0,529	0,047	0,027	0,461	0,052	0	0	0,073	0,073	0	0,006
97	0,461	0,571	0,014	0,515	0,6	0	0,397	0,384	0,617	0,36	0,277	0,023	0,014	0,133	0,128	0,013	0	0,254	0,254	0,269	0,599
98	0,009	0,013	0	0,025	0,077	0,276	0	0	0,034	0,005	0,018	0,008	0	0,018	0	0	0	0,007	0,007	0	0,051
99	-----	-----	0,431	-----	0,617	-----	0,035	-----	0,624	-----	0,357	-----	-----	-----	-----	-----	-----	-----	-----	-----	-----
100	0,599	0,444	0,061	0,105	0,613	0,402	0,279	0,116	0,409	0,323	0,43	0,35	-----	0,383	0	0,32	0,636	0,202	0,202	0,502	0,032
101	0,128	0,081	0,034	0,145	0,546	0,193	0,374	0,213	0,562	0,173	0,015	0,031	0,08	0,567	0,007	0,019	0,006	0,484	0,484	0,027	0,463
102	0,406	0,262	0,365	0,046	0,28	0,015	0,095	0,049	0,187	0,144	0,19	0,008	0	0,443	0,319	0,517	0	0,37	0,37	0,157	0,112
103	0,043	0,031	0,127	0,425	0,46	0	0,405	0,529	0,635	0,261	0,028	0,168	0,041	0,053	0,02	-----	0,273	0,08	0,08	0,515	-----
104	0,014	0,026	0,055	0	0	0,008	0	0,004	0,003	0	0	0	0,007	0	0,013	0	0,142	0,007	0,007	0	0
105	0,011	0	0,007	0,018	0,007	0,45	0,44	0,109	0,075	0,005	0	0	0	0	0,02	0	0	0,007	0,007	0	0
106	0,228	0,188	0,049	0,229	0,31	0,074	0,492	0,332	0,399	0,408	0,024	0,498	0,027	0,151	0,165	0	0	0,151	0,151	0,243	0,371
107	0,195	0,142	0,089	0,167	0,327	0,266	0	0,461	0,637	0,493	0,012	0,024	0,216	0	0,027	0	0	0	0	0,013	0,013
108	0,453	0,375	0,174	0,287	0,183	0,137	0,241	0,555	0,248	0,273	0,058	0,149	0,014	0,459	0,013	0	0,038	0,371	0,371	0,007	0
109	0,222	0,201	0,197	0,063	0,557	0	0,042	0,491	0,726	0,262	0,018	0,334	0,014	0,018	0,013	0	0	0,2	0,2	0,013	0
110	0,009	0,013	0,035	0	0,007	0,412	0	0,073	0,014	0,04	0	0,047	0,007	0	0	0	0	0	0	0	0
111	0,439	0,298	0,47	0,409	0,483	0	0,163	0,091	0,71	0,267	0	0,428	0,208	0,053	0	0	0	0,455	0,455	0	0,545
112	0,014	0,018	0,028	0	0,029	0	0	0,059	0,495	0,055	0	0,039	0,007	0	0	0	0	0,007	0,007	0	0
113	0,017	0,009	0,035	0,014	0,62	0	0,028	0,333	0,316	0,13	0	0,032	0,23	0	0	0,013	0,006	0	0	0,095	0,082
114	0,006	0,009	0,021	0	0	0,108	0	0,007	0,006	0,121	0,006	0,127	0	0	0	0	0	0	0	0	0
115	0,031	0,018	0,036	0,11	0,539	0,053	0,158	0,247	0,537	0,093	0	0,055	0,014	0	0,02	0,122	0	0,216	0,216	0,139	0
116	0,017	0,022	0,007	0,004	0,014	0,124	0,007	0,004	0	0,015	0	0,024	0	0	0	0	0	0	0	0	0
117	0,017	0,009	0,05	0,481	0,508	0,278	0,068	0,07	0,516	0,065	0,139	0,016	0,014	0,018	0	0	0,013	0,041	0,041	0	0,013
118	0,569	0,517	0,422	0,331	0,567	0,054	0,328	0,196	0,661	0,534	0,281	0,626	0,007	0	0	0	0	0	0	0	0
119	0,179	0,124	0,007	0,004	0,161	0,256	0,007	0,094	0,244	0,035	0	0,008	0,007	0	0,02	0,006	0,044	0	0	0	0
120	0,272	0,221	0,084	0,397	0,12	0,397	0,088	0,241	0,727	0,287	0,418	0,541	0,061	0	0	0	-----	0	0	0,007	0,007
	Fk1a	Fk1b	Fk3	Fk4a	Fk4b	Fk5a	Fk5b	Fk6	Fk8	Fk2	Fk7	Fk11	Fk14	Fk9a	Fk9b	Fk9c	Fk9d	Fk10a	Fk10b	Fk10c	Fk10d
