# Supplementary material for: Compression of Large Sets of Sequence Data Reveals Fine Diversification of Functional Profiles in Multigene Families of Proteins: A Study for Peptidyl-Prolyl cis/trans Isomerases (PPIase)
Source: Biomolecules. 2019 Feb 11;9(2):59. doi: 10.3390/biom9020059 (PMC6406343; doi:10.3390/biom9020059)
Supplement: Supplementary file 1 [file biomolecules-09-00059-s001.zip › Figure S5A-FKBP36.aln.pdf]

CLUSTAL 2.0.9 multiple sequence alignment of FKBP36s

```
NP_003593           GVLKDVIREGAG--DLVAPDA-SVL-VKYSGYLEHMDRPFDSN-YFRKT--PRLMKLGE-
XP_003815624        GVLKDVIREGAG--DLVAPDA-SVL-VKYSGYLEHMDRPFDSN-YFRKT--PRLMKLGE-
XP_003777068        GVLKDVIREGGG--DLVAPDA-SVL-VKYSGYLEHMDRPFDSN-YFRKT--PRLMKLGE-
XP_003276647        GVLKDVIREGAG--DLVAPDA-SVL-VKYSGYLEHMDRPFDSN-YFRKT--PRLMKLGE-
XP_003895916        GVLKDVIREGAG--DLVAPDA-SVL-VKYSGYLEHMDRPFDSN-YFRKT--PRLMKLGE-
EHH17242            GVLKDVIREGAG--DLVAPDA-SVL-VKYSGYLEHMDRPFDSN-YFRKT--PRLMKLGE-
XP_001110932        GVLKDVIREGAG--DLVAPDA-SVL-VKYSGYLEHMDRPFDSN-YFRKT--PRLMKLGE-
XP_011785750        GVLKDVIREGAG--DLVAPDA-SVL-VKYSGYLEHMDRPFDSN-YFRKT--PRLMKLGE-
NP_001128683        GVLKDVIREGAG--DLVAPDA-SVL-VKYSGYLEHMDRPFDSN-YFRKT--PRLMKLGE-
XP_017713171        GVLKDVIREGAG--DLVVPDA-SVL-VKYSGYLEHMDRPFDSN-YFRKT--PRLMKLGE-
XP_010385752        GVLKDVIREGAG--DLVVPDA-SVL-VKYSGYLEHMDRPFDSN-YFRKT--PRLMKLGE-
XP_002817917        GVLKDVIREGGG--DLVAPDA-SVL-VKYSGYLEHMDRPFDSN-YFRKT--PRLMKLGE-
XP_004045599        GVLKDVIREGAG--DLVAPDA-SVL-VKYSGYLEHLDRPFDSN-YFRKT--PRLMKLGE-
XP_008979905        GVLKDVIREGAG--DLVAPDA-TVL-VKYSGYLEHMDRPFDSN-CHRRT--PKLMKLGE-
XP_016868230        GVLKDVIREGAG--DLVAPDA-SVL-VKYSGYLEHMDRPFDSN-YFRKT--PRLMKLGE-
XP_003794239        GVLKDVIREGAG--ELVTPDA-SVL-VKYSGYLEHMDRPFDSN-CFRKT--PRLMKLGE-
XP_012597698        GVLKDVIREGAG--DLVTPDA-SVL-VKYSGYLEHMDKPFDSN-CFRKT--PRLMKLGE-
XP_011931834        GVLKDVIREGAG--DLVAPDA-SVL-VKYSGYLEHMDRPFDSN-YFRKT--PRLMKLGE-
XP_003895917        GVLKDVIREGAG--DLVAPDA-SVL-VKYSGYLEHMDRPFDSN-YFRKT--PRLMKLGE-
XP_008016547        GVLKDVIREGAG--DLVAPDA-SVL-VKYSGYLEHMDRPFDSN-YFRKT--PRLMKLGE-
XP_003934167        GVLKDVIREGAG--DLVAPDA-TVL-VKYSGYLEHMDRPFDSN-CHRRT--PKLMKLGE-
XP_012495716        GVLKDVIREGAG--DLVTPDA-SVI-VKYSGYLEHMDKPFDSN-CFRKT--PRLMKLGE-
XP_012304459        GVLKDVIREGAG--DLVAPDA-TVL-VKYSGYLEHMDRPFDSN-CHRRT--PKLMKLGE-
XP_022357643        GVLKDVIREGAG--ELVTPDA-SVL-VKYSGYLEHMDKPFDSN-CFRRT--PRLMKLGE-
XP_010385754        GVLKDVIREGAG--DLVVPDA-SVL-VKYSGYLEHMDRPFDSN-YFRKT--PRLMKLGE-
XP_017895560        GVLKDVIREGAG--ELVTPDA-SVL-VKYSGYLEHMDKPFDSN-CFRKT--PRLMKLGE-
XP_005892900        GVLKDVIREGAG--ELVTPDA-SVL-VKYSGYLEHMDKPFDSN-CFRKT--PRLMKLGE-
XP_010852603        GVLKDVIREGAG--ELVTPDA-SVL-VKYSGYLEHMDKPFDSN-CFRKT--PRLMKLGE-
XP_546928           GVLKDVIREGAG--ELVTPDA-SVL-VKYSGYLEHMDKPFDSN-CFRKT--PRLMKLGE-
XP_005225237        GVLKDVIREGAG--ELVTPDA-SVL-VKYSGYLEHMDKPFDSN-CFRKT--PRLMKLGE-
XP_021556246        GVLKDVIREGAG--ELVTPDA-SVL-VKYSGYLEHMDKPFDSN-CFRKT--PRLMKLGE-
XP_011978492        GVLKDVIREGAG--ELVTPDA-SVL-VKYSGYLEHMDKPFDSN-CFRKT--PRLMKLGE-
NP_001296390        GVLKDVIREGAG--ELVTPDA-SVL-VKYSGYLEHMDKPFDSN-CFRKT--PRLMKLGE-
XP_011959859        GVLKDVIREGAG--ELVTPDA-SVL-VKYSGYLEHMDKPFDSN-CFRKT--PRLMKLGE-
XP_006942097        GVLKDVIREGAG--ELVTPDA-SVL-VKYSGYLEHMDKPFDSN-CFRKT--PRLMKLGE-
XP_007100028        GVLKDVIREGSG--ELVTPDA-SVL-VKYSGYLEHMDKAFDSN-CFRKT--PRLMKLGE-
XP_005972253        GVLKDVIREGAG--ELVTPDA-SVL-VKYSGYLEHMDKPFDSN-CFRKT--PRLMKLGE-
XP_019277206        GVLKDVIREGAG--ELVTPDA-SVL-VKYSGYLEHMHKPFDSN-CFRKT--PRLMKLGE-
XP_014705926        GVLKDVIREGAG--ELVTPDA-SVL-VKYSGYLEHMDKPFDSN-CFRKT--PRLMKLGE-
XP_008540619        GVLKDVIREGAG--ELVTPDA-SVL-VKYSGYLEHMDKPFDSN-CFRKT--PRLMKLGE-
XP_020752493        GVLKDVIREGAG--ELVTPDA-SVL-VKYSGYLEHMDKPFDSN-CFRKT--PRLMKLGE-
XP_014988800        GVLKDVIREGAG--DLVAPDA-SVL-VKYSGYLEHMDRPFDSN-YFRKT--PRLMKLGE-
XP_011785755        GVLKDVIREGAG--DLVAPDA-SVL-VKYSGYLEHMDRPFDSN-YFRKT--PRLMKLGE-
XP_022444356        GVLKDVIREGSG--ELVTPDA-SVL-VKYSGYLEHMDKPFDSN-CFRKT--PRLMKLGE-
XP_008979887        GVLKDVIREGAG--DLVAPDA-TVL-VKYSGYLEHMDRPFDSN-CHRRT--PKLMKLGE-
XP_004399150        GVLKDVIREGAG--ELVTPDA-SVL-VKYSGYLEHMDKPFDSN-WFRKT--PRLMKLGE-
XP_004268847        GVLKDVIREGSG--ELVTPDA-SVL-VKYSGYLEHMDKPFDSN-CFRKT--PRLMKLGE-
XP_019659608        GVLKDVIREGAG--ELVTPDA-AVL-VKYSGYLEHMDKPFASN-CFRKT--PRLMKLGE-
XP_024602986        GVLKDVIQEGSG--ELVTPDA-SVL-VKYSGYLEHMDKPFDSN-CFRKT--PRLMKLGE-
XP_007186614        GVLKDVIREGSG--ELVTPDA-SVL-VKYSGYLEHMDKPFDSN-CFRKT--PRLMKLGE-
XP_014652016        GVLKDVIREGAG--ELVTQDA-SVL-VKYSGYLEHMDKPFDSN-CFRKT--PRLMKLGE-
XP_012597701        GVLKDVIREGAG--DLVTPDA-SVL-VKYSGYLEHMDKPFDSN-CFRKT--PRLMKLGE-
XP_004761711        GVLKDVIREGAG--ELVTPDA-SVL-VKYSGYLEHMDKPFDSN-CFRKT--PRLMKLGE-
XP_004317177        GVLKDVIREGSG--ELVTPDA-SVL-VKYSGYLEHMDKPFDSN-CFRKT--PRLMKLGE-
XP_005892901        GVLKDVIREGAG--ELVTPDA-SVL-VKYSGYLEHMDKPFDSN-CFRKT--PRLMKLGE-
```

XP_017895559	GVLKDVIREGAG--ELVTPDA-SVL-VKYSGYLEHMDKPFDSN-CFRKT--PRLMKLGE-
XP_005662030	GVLKDVIREGTG--ELVTPDA-SVL-VKYSGYLEHMDKPFDSN-CFRKT--PRLMKLGE-
XP_010852604	GVLKDVIREGAG--ELVTPDA-SVL-VKYSGYLEHMDKPFDSN-CFRKT--PRLMKLGE-
XP_010852602	GVLKDVIREGAG--ELVTPDA-SVL-VKYSGYLEHMDKPFDSN-CFRKT--PRLMKLGE-
EPY83956	GVLKDVIREGAG--ELVTPDA-SVL-VKYSGYLEHMDKPFDSN-CFRKT--LRLMKLGE-
NP_001095679	GVLKDVIREGAG--ELVTPDA-SVL-VKYSGYLEHMDKPFDSN-CFRKT--PRLMKLGE-
XP_008139950	GVLKDVIREGAG--ELVMPDA-SVL-VKYSGYLEHMDKPFDSN-CFRKT--PRLMKLGV-
XP_010970644	GVLKDVIREGAG--ELVTPDA-SVL-VKYSGYLEHMDKPFDSN-CFRKT--LRLMKLGE-
XP_004021020	GVLKDVIREGAG--ELVTPDA-SVL-VKYSGYLEHMDKPFDSN-CFRKT--PRLMKLGE-
XP_015450070	GVLKDIIREGAG--ELVTPDA-SVL-VKYSGYLEHMDKPFDSN-CLRKT--PRLMKLGE-
XP_017516100	GVLKAVIREGAG--ELVTPDA-SVL-VKYSGYLEHMDKPFDSN-YFRKT--PRLMKLGE-
XP_010342933	GVLKDVIREGAG--DLVAPDA-TVL-VKYSGYLEHMDRPFDSN-CHRRT--PKLMKLGE-
XP_006057167	GVLKDVIREGAG--ELVTPDA-SVL-VKYSGYLEHMDKPFDSN-CFRKT--PRLMKLGE-
XP_019843968	GVLKDVIREGAG--ELVTPDA-SVL-VKYSGYLEHMDKPFDSN-CFRKT--PRLMKLGE-
XP_006201442	GVLKDVIREGAG--ELVTPDA-SVL-VKYSGYLEHMDKPFDSN-CFRKT--LRLMKLGE-
XP_005972252	GVLKDVIREGAG--ELVTPDA-SVL-VKYSGYLEHMDKPFDSN-CFRKT--PRLMKLGE-
XP_004380900	GVLKDVLREGTG--GLVTPDA-SVL-VKYSGYLEHMDRPFDSN-CFRKT--PRLMKLGE-
XP_007468850	GVLKDVIREGSG--ELVTPDA-SVL-VKYSGYLEHMDKPFDSN-CFRKT--PRLMKLGE-
XP_007100027	GVLKDVIREGSG--ELVTPDA-SVL-VKYSGYLEHMDKAFDSN-CFRKT--PRLMKLGE-
XP_008966786	GVLKDVIREGAG--DLVAPDA-SVL-VKYSGYLEHMDRPFDSN-YFRKT--PRLMKLGE-
XP_020017458	GVLKDVIREGAG--DLVTPDA-SVL-VKYSGYLEHMDKPFDSN-CYRKT--PRLMKLGE-
XP_006088351	GVLKDVIREGAG--ELVVPDA-SVL-VKYSGYLEHMDKPFDSN-CFRKT--PRLMKLGV-
XP_020752496	GVLKDVIREGAG--ELVTPDA-SVL-VKYSGYLEHMDKPFDSN-CFRKT--PRLMKLGE-
XP_022444355	GVLKDVIREGSG--ELVTPDA-SVL-VKYSGYLEHMDKPFDSN-CFRKT--PRLMKLGE-
XP_006773483	GVLKDVIREGAG--ELVVPDA-SVL-VKYSGYLEHMDKPFDSN-CFRKT--PRLMKLGV-
XP_012304461	GVLKDVIREGAG--DLVAPDA-TVL-VKYSGYLEHMDRPFDSN-CHRRT--PKLMKLGE-
XP_008591583	GVLKDVIREGAG--ELVTPDA-SVL-VKYSGYLEHMDKPFDSN-CFRKT--PRLMKLGE-
XP_016054558	GVLKDVIREGAG--ELVTPDA-SVL-VKYSGYLEHMDKPFDSN-CFRKT--PRLMKLGV-
XP_011369980	GVLKDIIREGAG--ELVTPDA-SVL-VKYSGYLEHMDKPFDSN-CLRKT--PRLMKLGE-
XP_004268849	GVLKDVIREGSG--ELVTPDA-SVL-VKYSGYLEHMDKPFDSN-CFRKT--PRLMKLGE-
XP_024602987	GVLKDVIQEGSG--ELVTPDA-SVL-VKYSGYLEHMDKPFDSN-CFRKT--PRLMKLGE-
XP_019610492	GVLKDVVREGAG--ELVTPDA-SVL-VKYSGYLEHMDKPFDSN-CFRKT--PRLMKLGE-
XP_004317179	GVLKDVIREGSG--ELVTPDA-SVL-VKYSGYLEHMDKPFDSN-CFRKT--PRLMKLGE-
XP_006889852	GVLKDVLREGVG--DLVTPDA-SVL-VKYSGYLEHMDKPFDSN-CYRKT--PRLMKLGE-
XP_007940604	GVLKDVLQEGAG--DLVTPDA-SVL-VKYSGYLEHMDKPFDSN-CFRKT--PRLMKLGE-
XP_014411577	GVLKDVIREGAG--ELVTPDA-SVL-VKYSGYLEHMDKPFDSN-CFRKT--LRLMKLGE-
XP_007073943	GVLKDVIREGAG--ELVTPDA-SVL-VKYSGYLEHMDKPFDSN-CFRKT--PRLMKLGE-
XP_006155025	GVLKHVIREGAG--ELVPPDA-SVL-VKYSGYLEHMDKPFDSN-CFRKT--PRLMKLGE-
XP_005328656	GVLKDVIREGTG--DLVTPDA-SVL-VKYSGYLEHMDKPFDSN-CFRKT--PRLMKLGE-
XP_011765609	GVLKDVIREGAG--DLVAPDA-SVLGVPWGG--------LSAG-SMSSH--SLFLCLLT-
XP_010979058	GVLKDVIREGTG--ELVTPDA-SVL-VKYSGYLEHMDKPFDSN-CFRKT--LRLMKLGE-
XP_006249193	GVLKDIIREGAG--DPVTPDA-SVL-VKYSGYLEHMDKPFDSN-CFRKT--PRLMKLGE-
XP_002722023	GVLKDVIREGVG--ELVTPDA-SVL-VKYSGYLEHMDKPFDSN-CFRKS--PRLMKLGE-
XP_005344683	GVLKDIIREGAG--DPVTPDA-SVL-VKYSGYLEHMDKPFDSN-YFRKA--PRLMKLGE-
XP_020941978	GVLKDVIREGTG--ELVTPDA-SVL-VKYSGYLEHMDKPFDSN-CFRKT--PRLMKLGE-
XP_006859898	GVLKDVLREGTG--DLVTPDA-AVL-VKYSGYLEHMDKPFDSN-CFRKT--PRLMKLGE-
XP_005549416	GVLKDVIREGAG--DLVAPDA-SVL-VKYSGYLEHMDRPFDSN-YFRKT--PRLMKLGE-
XP_024427494	GVLKDVIREGAG--ELVTPDA-SVL-VKYSGYLEHMDKPFDSN-CFRKT--PRLMKLGE-
XP_016001971	GVLKDIIREGAG--ELVTPDA-SVL-VKYSGYLEHMDKPFDSN-CLRKT---RLMKLGE-
XP_007468849	GVLKDVIREGSG--ELVTPDA-SVL-VKYSGYLEHMDKPFDSN-CFRKT--PRLMKLGE-
XP_014988802	GVLKDVIREGAG--DLVAPDA-SVL-VKYSGYLEHMDRPFDSN-YFRKT--PRLMKLGE-
XP_012584338	GVLKDVIREGAG--ELVTPEA-SVL-VKYSGYLEHMDKPFDSN-CFRRV--PRLMKLGE-
XP_006971358	GVLKDVIREGTG--DPVTPDA-SVL-VKYSGYLEHMDKPFDSN-CFRKT--PRLMKLGE-
XP_004620930	GVLKDVIREGVG--ELVTPDA-SVL-VKYSGYLEHMDKPFDSN-CSRKT--PRLMKLGE-
XP_008696858	GVLKDVIREGAG--ELVTPDA-AVL-VKYSGYLEHMDKPFASN-CFRKT--PRLMKLGE-
XP_021018812	GVLKDIIREGTG--DTVTPDA-SVL-VKYSGYLEHMDKPFDSN-CFRKT--PRLMKLGE-
XP_012782940	GVLKDVIREGVG--ELVTPDA-SVL-VKYSGYLEHMDKPFDSN-CFRKT--PRLMKLGE-
XP_019502388	GVLKDVVREGAG--ELVTPDA-SVL-VKYSGYLEHMDKPFDSN-CFRKV--PRLMKLGE-

XP_021042681	GVLKDVIREGAG--DPVTPDA-SVL-VKYSGYLEHMDKPFDSN-CFRKT--HRLMKLGE-
NP_001264821	GVLKDIIREGTG--DTVTPDA-SVL-VKYSGYLEHMDKPFDSN-CFRKT--PRLMKLGE-
XP_021519217	GVLKDIIREGAG--DPVTPDA-SVL-VKYSGYLEHMDKPFDSN-CFRKA--PRLMKLGE-
XP_005344682	GVLKDIIREGAG--DPVTPDA-SVL-VKYSGYLEHMDKPFDSN-YFRKA--PRLMKLGE-
NP_001349718	GVLKDVIREGAG--DLVAPDA-SVL-VKYSGYLEHMDRPFDSN-YFRKT--PRLMKLGE-
NP_001099392	GVLKDIIREGAG--DPVTPDA-SVL-VKYSGYLEHMDKPFDSN-CFRKT--PRLMKLGE-
XP_005396843	GVLKDVIREGAG--DLVTPDA-SVL-VKYSGYLEHMDKPFDSN-CFRKT--PRLMKLGE-
XP_019502390	GVLKDVVREGAG--ELVTPDA-SVL-VKYSGYLEHMDKPFDSN-CFRKV--PRLMKLGE-
XP_006971357	GVLKDVIREGTG--DPVTPDA-SVL-VKYSGYLEHMDKPFDSN-CFRKT--PRLMKLGE-
XP_008966787	GVLKDVIREGAG--DLVAPDA-SVL-VKYSGYLEHMDRPFDSN-YFRKT--PRLMKLGE-
XP_003500991	GVLKDIIREGAG--DTVTPDA-SVL-VKYSGYLEHMDKPFDSN-CFRKT--PRLMKLGE-
XP_008846596	GVLKNVIREGAG--DPVTPDA-SVL-VKYSGYLEHMDKPFDSN-CFRKT--PRLMKLGE-
XP_021563404	GVLKDVLRRGSG--DLVTPDA-SVL-VKYSGYLEHVDKPFDSN-CFRKT--PQLMKLGM-
XP_018887307	GVLKDVIREGAG--DLVAPDA-SVL-VKYSGYLEHLDRPFDSN-YFRKT--PRLMKLGE-
XP_021018806	GVLKDIIREGTG--DTVTPDA-SVL-VKYSGYLEHMDKPFDSN-CFRKT--PRLMKLGE-
XP_017176678	GVLKDIIREGTG--DTVTPDA-SVL-VKYSGYLEHMDKPFDSN-CFRKT--PRLMKLGE-
NP_291049	GVLKDIIREGTG--DTVTPDA-SVL-VKYSGYLEHMDKPFDSN-CFRKT--PRLMKLGE-
XP_021042679	GVLKDVIREGAG--DPVTPDA-SVL-VKYSGYLEHMDKPFDSN-CFRKT--HRLMKLGE-
XP_010617304	GVLKDVIREGAG--DLVTPDA-SVL-VKYSAYLEHMDKPFDSN-CFRKT--PRLMKLGE-
XP_021519216	GVLKDIIREGAG--DPVTPDA-SVL-VKYSGYLEHMDKPFDSN-CFRKA--PRLMKLGE-
XP_023420891	GVLKDVIREGAA--DLXTPTP-LXL-VKYSGYLEHMDKPFDSN-CFRKT--PRLMKLGE-
XP_006155027	GVLKHVIREGAG--ELVPPDA-SVL-VKYSGYLEHMDKPFDSN-CFRKT--PRLMKLGE-
XP_011765605	GRWRQLERNDEC--LLVAPEWNAAI-VKYSGYLEHMDRPFDSN-YFRKT--PRLMKLGE-
XP_015302699	GRWRQLERNDEC--LLVAPEWNAAI-VKYSGYLEHMDRPFDSN-YFRKT--PRLMKLGE-
XP_011765611	GVLKDVIREGAG--DLVAPDA-SVL-VKYSGYLEHMDRPFDSN-YFRKT--PRLMKLGE-
XP_012597702	GVLKDVIREGAG--DLVTPDA-SVL-VKYSGYLEHMDKPFDSN-CFRKT--PRLMKLGE-
XP_014988803	GVLKDVIREGAG--DLVAPDA-SVL-VKYSGYLEHMDRPFDSN-YFRKT--PRLMKLGE-
XP_010617307	GVLKDVIREGAG--DLVTPDA-SVL-VKYSAYLEHMDKPFDSN-CFRKT--PRLMKLGE-
XP_004666244	GVLKDVIREGTG--DAVTPDA-TVL-VKYSGYLEHMDKPFDSN-YFRKT--PRLMKLGE-
XP_004630630	GVLKDVIREGAG--DLVTREA-SVL-VKYSGYLEHMDKPFDSN-CFRKT--PRLMKLGE-
XP_004840130	GVLKDVIREGAG--DLVTPDA-SVL-VKYSGYLEHMDKPFDSN-CFRKT--PRLMKLGE-
XP_014403986	KRRRDVTEVRVL--VHFSVSVPSVL-VKYSGYLEHMDKPFDSN-CFRKT--PRLMKLGV-
XP_016800790	GVLKDVIREGAG--DLVAPDA-SVL-VKYSGYLEHMDRPFDSN-YFRKT--PRLMKLGE-
XP_014988804	----------------------------------MDRPFDSN-YFRKT--PRLMKLGE-
ELK06863	GVLKDIIREGAG--ELVTPDA-SVL-VKYSGYLEHMDKPFDSN-CLRKT--PRLMKLGE-
EHB08283	GVLKDVIREGAG--DLVTPDA-SVL-VKYSGYLEHMDKPFDSN-CFRKT--PRLMKLGE-
XP_004706046	GVLKDVLREGTG--DLVAPDS-SVL-VKYSGYLEHMDKPFDSN-YFRKT--PRLMKLGE-
XP_021110082	GVLKDVIREGAG--DLVTPDA-SVL-VKYSGYLEHMDKPFDSN-CFRKT--PRLMKLGE-
XP_007518064	GVLKDVIREGAG--ELVAPDA-SVL-VRYSGYLEHMDKPFDSN-SYRKT--PRLMKLGE-
PNI92595	GVLKDVIREGAG--DLVAPDA-SVL-VKYSGYLEHMDRPFDSN-YFRKT--PRLMKLGE-
XP_020861399	GVLKEVIREGAG--DLVMPDA-SVL-VKYSGYLEHMDKPFDTN-CFRKI--PRLMKLGE-
XP_016284889	GVLKEVIREGSG--DLVTPDA-SVL-VKYSGYLEHMDKPFDSN-CYRKI--PRLMKLGE-
XP_016284888	GVLKEVIREGSG--DLVTPDA-SVL-VKYSGYLEHMDKPFDSN-CYRKI--PRLMKLGE-
XP_001519210	GVLKEIIREGAG--ELVPPDA-SVS-VKYAGYLEHMDQPFDTN-WYRKL--PRLMKLGE-
XP_020861404	GVLKEVIREGAG--DLVMPDA-SVL-VKYSGYLEHMDKPFDTN-CFRKI--PRLMKLGE-
XP_014425414	GVLKEVIRAGTG--EMVPQDA-SVV-VKYSGYLEYMDKPFDTN-SYRRN--PRLLKLGE-
XP_004761713	GVLKDVIREGAG--ELVTPDA-SVL-VKYSGYLEHMDKPFDSN-CFRKT--PRLMKLGE-
XP_007441600	GVLKEIIRNGSG--ETVPPDA--SVLVKFSGYLEHMDRPFDTN-CFRKN--PKLMKLGD-
XP_016080873	GVLKEIIREGAG--ELVPPDA-SVS-VKYAGYLEHMDQPFDTN-WYRKL--PRLMKLGE-
XP_024066689	GVLKEVIRAGAG--EIVPQDA-SVL-VKYSGYLEYVDKPFDTN-WYRRN--PRLMKLGE-
XP_019365238	GVLKEVVNTGSG--ELVPHDA-SVL-VKYSGYLEYMDKPFDTN-RYRRS--SRLMKLGK-
XP_019401968	GVLKEVVRTGSG--ELVPRDA-SVL-VKYSGYLEYMDKPFDTN-RYRRS--SRLMKLGK-
XP_013926437	GVLKEIIRDGSG--ETVPPDS--SVLVKFSGYLEHTDRPFDTN-WFKKN--SKLMKLGE-
XP_019401969	GVLKEVVRTGSG--ELVPRDA-SVL-VKYSGYLEYMDKPFDTN-RYRRS--SRLMKLGK-
XP_015269029	GVLKEVIRAGAG--DVVPAEA--SVLVKFSGYLEHMDRPFDTN-CFKKN--LRLMKLGE-
XP_014464913	GVLKEVVRVGSG--ELVAPDA-SVL-VKYSGYLEYMDKPFETN-CYRRS--SRLMKLGK-
XP_006258869	GVLKEVVRVGSG--ELVAPDA-SVL-VKYSGYLEYMDKPFETN-CYRRS--SRLMKLGK-
XP_005344684	GVLKDIIREGAG--DPVTPDA-SVL-VKYSGYLEHMDKPFDSN-YFRKA--PRLMKLGE-

```
XP_015670323      GVLKEIIRNGSG--ETVPPDS--SVLVKFSGYLEHTDRPFDTN-WFKKN--PKLMKLGE-
XP_020662988      GVLKEIIRAGYG--EVVPQDA--SVLVKFSGYLEHMDRPFDTN-CFRKN--PKLMKLGK-
XP_007065519      GVLKEVIRAGAG--EMVPQDA-SVL-VKYSGYLEYMDKPFDTN-WYRRN--PRLMKLGE-
XP_020662989      GVLKEIIRAGYG--EVVPQDA--SVLVKFSGYLEHMDRPFDTN-CFRKN--PKLMKLGK-
XP_020662990      GVLKEIIRAGYG--EVVPQDA--SVLVKFSGYLEHMDRPFDTN-CFRKN--PKLMKLGK-
XP_025051231      GVLKEVVHVGSG--ELVARDA-SVL-VKYSGYLEYMDKPFETN-CYRRS--SRLMKLGK-
KYO33272          GVLKEVVRVGSG--ELVAPDA-SVL-VKYSGYLEYMDKPFETN-CYRRS--SRLMKLGK-
XP_021018814      GVLKDIIREGTG--DTVTPDA-SVL-VKYSGYLEHMDKPFDSN-CFRKT--PRLMKLGE-
OBS57796          GVLKDIIREGAG--DPVTPDA-S-----------YMDKPFDSN-CFRKT--PRLMKLGE-
NP_001264822      GVLKDIIREGTG--DTVTPDA-SVL-VKYSGYLEHMDKPFDSN-CFRKT--PRLMKLGE-
XP_021042683      GVLKDVIREGAG--DPVTPDA-SVL-VKYSGYLEHMDKPFDSN-CFRKT--HRLMKLGE-
XP_021519218      GVLKDIIREGAG--DPVTPDA-SVL-VKYSGYLEHMDKPFDSN-CFRKA--PRLMKLGE-
XP_024653358      GRWRQLERNDEC--LLVAPEWNAAI-VKYSGYLEHMDRPFDSN-YFRKT--PRLMKLGE-
XP_009678488      GVLKEVLRRGAG--ELVPPDA--SVAVKYSGYLEYAEEPFCTN-CYRKL--PRLMKLGK-
XP_015302701      GRWRQLERNDEC--LLVAPEWNAAI-VKYSGYLEHMDRPFDSN-YFRKT--PRLMKLGE-
XP_003770271      -PSRSQDPGAAA--GWPRSDS-GSS-VKYSGYLEHMDKPFDTN-CYRKV--PRLMKLGE-
ETE73718          GVLKEIIQDGSG--ETVPPDS--SVLVKFSGYLEHTDRPFDTN-WFKKN--PKLMKLGE-
NP_989052         GVLKEVIRPGKG--GKVPCDA-TVI-LKYSGYLEHADKPFDTS-CYRRH--PKMMKLGE-
NP_001085204      GVLKEVIRPGKG--GKVPCDA-TVI-LKYSGYLEHADKPFDTS-CYRRH--PRMMKLGQ-
XP_011592867      GVLKEELRPGSG--QAVPPAA--SVAVKYSGYLEHWDEPFCTN-YYSKF--PGLMKLGK-
XP_010584273      GVLKEELRPGTG--QAVPPAA--SVAVKYSGYLEHWDEPFCTN-YYSRF--PGLMKLGK-
XP_017946629      GVLKEVIRPGKG--GKVPCDA-TVI-LKYSGYLEHADKPFDTS-CYRRH--PKMMKLGE-
XP_008109385      GVLKEILRDGCG--ETVPPGA--SVLIKFSGYLEHMDRPFD---CSWKD--PKLMKLGQ-
XP_021271278      GVRKELLRHGTG--QPVPPAA--TVAVKYSGYLEHMEKPFCTN-CTKKL--PRLMKLGE-
XP_023074397      GVLKDVIREGAG--DLVAPDA-SVL-VKYSGYLEHMDRPFDSN-YFRKT--PRLMKLGE-
XP_015151391      GVRKQVLRPGTG--QPVPPAA--TVAVKYSGYLEHMEKPFCTN-CTKKL--PRLMKLGK-
XP_018100492      GVLKEVIRPGKG--GKVPCDA-TVI-LKYSGYLEHADKPFDTS-CYRRH--PRMMKLGQ-
POI24993          GVRKQVLRPGTG--QPVPPAA--TVAVKYSGYLEHMEKPFCTN-CTKKL--PRLMKLGK-
PNI92596          GVLKDVIREGAG--DLVAPDA-SVL-VKYSGYLEHMDRPFDSN-YFRKT--PRLMKLGE-
XP_015736491      GVRKELLRPGTG--QLVPPAA--TVAVKYFGYLENMEKPFCTN-CTKKL--PKLMKLGE-
XP_010283169      GVRKEELRPGIG--QAVPPAA--SVAVKYSGYLENGDEPFCTN-CYSKS--PGLMKLGK-
PNJ85879          GVLKDVIREGGG--DLVAPDA-SVL-VKYSGYLEHMDRPFDSN-YFRKT--PRLMKLGE-
XP_018412624      GVLKEIIRAGCG--ETVPIDS-TII-AKYSGYLEHSDKPFDTN-WFRRN--PRLMKLGE-
XP_010211497      PVSERELRVLVR--EPCRFGD--ALAVKYSGYLEYADEPFCTN-CYRKV--PRLMKLGK-
XP_017388864      GVLKDVIREGAG--DLVAPDA-TVL-VKYSGYLEHMDRPFDSN-CHRRT--PKLMKLGE-
XP_008109386      GVLKEILRDGCG--ETVPPGA--SVLS----YLEHMDRPFD---CSWKD--PKLMKLGQ-
XP_014342809      GVLKEVIQAGAG--EMVPPSA-SVA-VNFSGYLEYLDKPFDTN-CNKRN--PRLMKLGE-
KFV84016          GVLKEVLRRGAG--ELVPPDA--SVAVKYSGYLEYAEEPFCTN-CYRKL--PRLMKLGK-
XP_013812139      ARGPQQPAADGP--CSGTGER--FATVKYSGYLEYADEPFCTN-CYRKL--PRLMKLGK-
XP_009643249      ---------------MSEDRS--SAPVKYSGYLEHRDEPFCTN-CYSKS--PGLMKLGK-
XP_009928379      ---------------------------KYSGYLEHWDEPFCTN-YYSRF--PGLMKLGK-
XP_010124464      ---------------------------TYSGFLEHLDEPFCIN-RYGAS--PGLLKLGK-
XP_009321903      ---------MLV--VTRDGFG--LWRLKYTGYLEHWDEPFCTN-CYSKS--PGLMKLGK-
XP_009807780      ---------------------------KYSGYLEHCNEPFCTN-GYSKS--PGLMKLGK-
XP_009512874      ---------------------------KYSGYLEHWNEPFCTN-CYSKS--PGLMKLGK-
XP_009586283      ---------------------------KYSGYLEHWDKPFCTN-CYSKS--PRLMKLGK-
KFQ72829          GVRKEELRPGIG--QAVPPAA--SVAVKYSGYLENGDEPFCTN-CYSKS--PGLMKLGK-
XP_009698973      ---------------------------KYSGYLEHGDEPFCTN-CYSKS--PGLMKLGK-
XP_009901738      GVCKHVLRSGTG--QLVPPNS--SVEVKYSGYLEDSDKPFCTN-RNHKV--PELMKLGK-
XP_012887159      GVLKDIIREGAG--DLVAPDA-LVL-VKFSGYLEHMDKPFDSN-CYRKT--PRLMKLGE-
XP_009940812      -----------M--DILSRTG--ISSMNYSGYLEHGDEPFCTN-SYSKF--PRLMKLGK-
XP_002187983      GVRKEQLRPGNG--QPVPPDA--SVAVKYSGYLGNWNKLFCSN-GNSKY--PRLMKLGK-
XP_009887190      --------------MSREAHW--GFLMKYSGYLEHRDEPFCTS-GYSKS--PRLMKLGK-
XP_009486380      ---------------------------KYSGYLEHWDKPFCTN-CYSKS--SGLMKLGK-
KGL76252          -------------------------AVKYSGYLEYADEPFCTN-CYRKV--PRLMKLGK-
XP_010306733      ---------------------------KYSGYLEHRDKPFCTN-CYSKF--PGLMKLGK-
XP_008979919      GVLKDVIREGAG--DLVAPDA-TVL-VKYSGYLEHMDRPFDSN-CHRRT--PKLMKLGE-
XP_009998510      ----------------MLLMA--LSLVNYFGYLEHRDEPF-TK-CCSKF--PKLMKLGK-
```

XP_009463122	PSGAFAWPACQT--ACSPPQG--RPAVKYSGYLEQCDEPFCTN-CYSKF--PGLMKLGK-
XP_007894626	GALKETLRSGSG--PVVPADA--LVSVYYSAYVEHANEPFDSN-VHRKV--PGFMKLGR-
XP_010022120	---------------------------KFSGYLEDQHKPFCTN-CYSKS--PGLMKLGK-
XP_010083598	---------------------------RYSGYLEGSDEPFCTS-CNRKY--PRLMKLGR-
XP_010194269	---------------------------KFSGYLEQSDEPFCTN-CKS----PRLVKLGK-
XP_009962683	---------------------------KYSGYLEHSDEPFCTN-CYSKF--PELMKLGK-
KFP29338	--------------------------VKFSGYLEQSDEPFCTN-CKS----PRLVKLGK-
XP_010147897	------------------MNA--SVAVKYSGYLEQQDEPFCTN-CNSRS--PGLMKLGR-
KFQ39479	GVCKEELYPGSG--QVVPPNA--SVEVKYSGYLDHWDKPFCTN-RH-----PELLKLGK-
EOB04024	--------------------------VKYSGYLEHADEPFCTN-CTTKL--PRLMKLGE-
XP_023675251	GVLKEVIQNGEG--QLIPMDA--SVLIHFSGYLEYSDRPFESN-NRSKH--PPMMKLGR-
XP_009557002	----MADSARLR--VSVRDWL--RAGVKYTGYFEHKEEPFCTN-CHRRF--PGLMKLGK-
XP_008944700	---------------------------KYSGYLEGSDEPFCTN-GNSKF--PELMKLGK-
KFP93140	--------------------------VKYSGYLEHWDEPFCTN-YYSRF--PGLMKLGK-
KFP44723	-------------------------AVTYSGFLEHLDEPFCIN-RYGAS--PGLLKLGK-
XP_017553040	GILKEVIRAGDG--SPVPMDA--SVSVHYSGFLEYSDRPFDST-THLKF--PRMVKLGK-
XP_021396070	GVRKEQLRPGTG--QPVPPGA--SVAVKYSGYLGNWNKLFCSN-GNSKY--PRLMKLGK-
XP_010143455	---------------------------KYSGYLEHSDEPFCTN-YHHKV--SKLMKLGR-
KFQ87275	-------------------------AVKYSGYLEHWDEPFCTN-CYSKS--PGLMKLGK-
XP_010891852	GILKEVVQPGEG--QPVPRDS--SVSIHFSGFLEYSDRPFETT-NQLKY--PRMMKLGR-
XP_009863922	---------------------------RYSGYLEHQDEPFCTN-WYDRS--HGLMKLGK-
KFW66808	--------------------------VKYTGYLEHWDEPFCTN-CYSKS--PGLMKLGK-
XP_018591809	GVLKELVHGGDGGGRLCPRNA--TVHIQYSGYLEYSDRPFEST-AHFKH--PQMMKLGT-
KFP21064	--------------------------VKYSGYLEHRDEPFCTN-CYSKS--PGLMKLGK-
KFZ64979	-------------------------AVKYSGYLEHWDEPFCTN-CHSKS--PGLMKLGK-
KFW73903	--------------------------VKYSGYLEHWNEPFCTN-CYSKS--PGLMKLGK-
KFM05579	--------------------------VKYTGYLEHWDEPFCTN-CYSKS--PGLMKLGK-
KFV69563	GVCKHVLRSGTG--QLVPPNS--SVEVKYSGYLEDSDKPFCTN-RNHKV--PELMKLGK-
XP_023823731	GIKKEVVQPGEG--PPVPRDS--SVSINFSGFLEYSDRPFETT-SYLKY--PRMMKLGR-
XP_016346087	GILKEVVHAGEG--PPVPKHA--SVSIHFSGFIEYSDAPFETT-SHLKF--PRMMKLGR-
KFV51798	--------------------------VKYSGYLEHCNEPFCTN-GYSKS--PGLMKLGK-
KFW03341	GVRKKELWPGAG--PTVPMNA--SVAVKYSGYLEQQDEPFCTN-CNSRS--PGLMKLGR-
KFV99355	--------------------------VKYSGYLEHWDKPFCTN-CYSKS--PRLMKLGK-
OXB56102	GIRKEVLRPGSG--ELVPPDA--TVAVKYSGYLEGMEEPFCTY-QTKGF--PRLMKLGV-
XP_018958387	GILKEVVHAGEG--PPVPKHA--SVSIHFSGFIEYSDAPFETT-NHLKF--PRMMKLGR-
XP_019903677	GILKEVVQPGEG--QPVPRDS--SVSIHFSGFLEYSDRPFETT-NQLKY--PRMMKLGR-
KFU91562	--------------------------VNYFGYLEHRDEPF-TK-CCSKF--PKLMKLGK-
XP_017314960	GIRKEVVREGEG--PLVPRDA--SVSVHFSGFLEYADQPFEST-RGSKF--PKMMKLGK-
XP_016393789	GILKEVVHAGEG--PPLPKHA--SVSIHFSGFIEYSDAPFETT-SLLKF--PRMMKLGR-
XP_009093115	GVRKEQLRPGTG--QPVPPSA--SVAVKYSGYLGNWNKLFCSN-GNSKY--PRLMKLGK-
XP_023869471	GILKEVVQPGEG--PPVPRDS--SVSIHFSGFLEYSDRPFETT-SHLKY--PRMMKLGR-
XP_019903679	GILKEVVQPGEG--QPVPRDS--SVSIHFSGFLEYSDRPFETT-NQLKY--PRMMKLGR-
XP_019903678	GILKEVVQPGEG--QPVPRDS--SVSIHFSGFLEYSDRPFETT-NQLKY--PRMMKLGR-
XP_014110391	GVRKERLCPGTG--QPVPPSA--SVAVKYSGYLGNWNKLFCSN-GNSKY--PRLMKLGK-
XP_017314964	GIRKEVVREGEG--PLVPRDA--SVSVHFSGFLEYADQPFEST-RGSKF--PKMMKLGK-
XP_019903676	GILKEVVQPGEG--QPVPRDS--SVSIHFSGFLEYSDRPFETT-NQLKY--PRMMKLGR-
XP_015502317	GVRKEQLCPGTG--QPVPPSA--SVAVKYSGYLGNWNKLFCSN-GNSKY--PRLMKLGK-
XP_020341240	GILKEVVQPGEG--PPVPRDS--SVSIHFSGFLEYSDRPFETT-SHLKY--PRMMKLGR-
NP_001122145	GVLKEVIHEGEG--PPVSMHA--SVSINFSGFIEYTDAPFETT-NHLKY--PRMMKLGK-
OWK52486	GVRKEQLRPGTG--QPVPPGA--SVAVKYSGYLGNWNKLFCSN-GNSKY--PRLMKLGK-
XP_013992803	GILKEVVQPGEG--PPVPRDS--SVSIHFSGFLEYSDRPFETT-SHLKY--PRMMKLGR-
XP_024287707	GILKEVVQPGEG--PPVPRDS--SVSIHFSGFLEYSDRPFETT-SHLKY--PRMMKLGR-
AAH54673	GVLKEVIHEGEG--PPVPMHA--SVSINFSGFIEYTDAPFETT-NHLKY--PRMMKLGK-
KFP60963	--------------------------VKYSGYLEHGDEPFCTN-CYSKS--PGLMKLGK-
XP_018958370	GILKEVVHAGEG--PPVPKHA--SVSIHFSGFLEYSDTPFETT-SHLKY--PRMMKLGR-
KGL96380	-------------------------AVKYSGYLEHRDEPFCTS-GYSKS--PRLMKLGK-
XP_016148031	GILKEVVHAGEG--PPVPKHA--SVSIHFSGFIEYSDAPFETT-SHLKF--PRMMKLGR-
XP_007245276	GIFKEVIRAGDG--PAVQRDS--TVSVNFSAFLEYADQPFDSS-THLKF--PKMMKLGK-

```
XP_023794776      GVRKERLCPGTG--QPVPPSA--SVAVKYSGYLGNWNKLFCSN-GNSKY--PRLMKLGK-
XP_021479427      GILKEVVQPGEG--PPVPRDS--SVSIHFSGFLEYSDRPFETT-SHLKY--PRMMKLGR-
KFR16943          -------------------------AVNYSGYLEHGDEPFCTN-SYSKF--PRLMKLGK-
XP_009988051      ---------MLV--LTRDRFG--LWRLKYSGYLEHTGEPFCTT-YNSKS--PELMKLGK-
KFV16869          -------------------------SVRYSGYLEGSDEPFCTS-CNRKY--PRLMKLGR-
KFO13846          --------------------------VKYSGYLEHRDKPFCTN-CYSKF--PGLMKLGK-
XP_020373313      GVLKEILRPGSG--SVIPSTA--SVSVHYSAYSEYTDKPFDSN-VQRDV--PRFMKLGR-
XP_006640997      GILKEVIHPGVG--QLVPENA--SVSFRYAGFLEYSDSPFESN-SYLKH--PRLMRLGR-
XP_020341239      GILKEVVQPGEG--PPVPRDS--SVSIHFSGFLEYSDRPFETT-SHLKY--PRMMKLGR-
KQK73326          ----------------MCPAC--RHPVKFSGYLEHQDKPFCTN-CYSKS--PGLMKLGK-
XP_008500186      ------MPTLRQ--AGFPPPT--YACMNYFGFLEDKDKPFCTH-LDSKS--SKLMKLGT-
XP_013992801      GILKEVVQPGEG--PPVPRDS--SVSIHFSGFLEYSDRPFETT-SHLKY--PRMMKLGR-
XP_016350445      GILKEVVHAGEG--PPVPKHA--SVSIHFSGFIEYSDTPFETT-SHLKY--PRMMKLGR-
KFV47875          --------------------------VKYSGYLEHSDEPFCTN-CYSKF--PELMKLGK-
XP_022534491      GIFKEVIRAGDG--PAVQRDS--TVSVNFSAFLEYADQPFDSS-THLKF--PKMMKLGK-
XP_021479426      GILKEVVQPGEG--PPVPRDS--SVSIHFSGFLEYSDRPFETT-SHLKY--PRMMKLGR-
KFQ96759          -------------------------AVKYSGYLEQCDEPFCTN-CYSKF--PGLMKLGK-
XP_012685286      GILKRVIQPGDG--PLVPRDA--SVSVNLSGYLEYCDDPFETT-KKLKH--PRMMKLGR-
KFQ41466          --------------------------VKFSGYLEDQHKPFCTN-CYSKS--PGLMKLGK-
XP_024997748      GVRKQVLRPGTG--QPVPPAA--TVAVKYSGYLEHMEKPFCTN-CTKKL--PRLMKLGK-
KFQ35533          ---------PLP--LTLG------CTVKYSGYLEGSDEPFCTN-GNSKF--PELMKLGK-
XP_020459257      GILKEVVQPGEG--ASIPPNA--SILIYYSGFLEYSDQPFETN-RDFKY--PRMMKLGR-
XP_017264554      GILKEVVQPGEG--PPVPQNA--SVLMYYSGFLEYSDQPFDTT-THLKY--PPMMKLGR-
XP_005917667      GILKEVVQPGEG--PPVPENA--SVLIHYSGFLEYSDQPFETT-THLKY--PRMMKLGR-
XP_022621130      GILKEVVQPGEG--PPVPQNA--SVLIHYSGFLEYSDQPFETT-TNLKY--PQMMKLGR-
XP_005725080      GILKEVVQPGDG--PPVPENA--SVLIHYSGFLEYSDQPFETT-THLKY--PRMMKLGR-
XP_010408101      --YEIKLLLFPF--SPLLALG---CTVKYSGYLGNWNNLFCSN-ESSKY--PRLMKLGK-
KFO74526          --------------------------VKYTGYFEHKEEPFCTN-CHRRF--PGLMKLGK-
XP_005449739      GILKEVVQPGEG--PPVPENA--SVLIHYSGFLEYSDQPFETT-TNLKY--PRMMKLGR-
XP_023285908      GILKEVVQPGEG--SPVPQNA--SVLIHYSGFLEYSDQPFETT-TNFKY--PQMMKLGR-
XP_015502316      GVRKEQLCPGTG--QPVPPSA--SVAVKYSGYLGNWNKLFCSN-GNSKY--PRLMKLGK-
XP_004542108      GILKEVVQPGEG--PPVPENA--SVLIHYSGFLEYSDQPFETT-THLKY--PQMMKLGR-
XP_023823730      GIKKEVVQPGEG--PPVPRDS--SVSR----FLEYSDRPFETT-SYLKY--PRMMKLGR-
XP_020459258      GILKEVVQPGEG--ASIPPNA--SILIYYSGFLEYSDQPFETN-RDFKY--PRMMKLGR-
XP_008634949      --YEIKLLLFPF--SPLLALG---CTVKYSGYLGNWNNLFCSN-ESSKY--PRLMKLGK-
XP_013883182      GILKQVVLPGEG--PPVPQNS--SVLMYYSGFLEYSDQAFDST-THLKY--PPMMKLGR-
XP_019947232      GIMKEVVQPGEG--PPISQNA--SVLIHYSGFLEYSDQPFETT-TNFKH--PRLMKLGK-
KFP85199          --------------------------VRYSGYLEHQDEPFCTN-WYDRS--HGLMKLGK-
PWA27518          GILKEVVQPGEG--PPVPQNA--SVIMYYSGYLEYSNQPFEST-AHLQY--PPLMKLGR-
XP_003971664      GILKELVQAGDG--PPVPYNA--SVLMHYSGYLEYSNVPFETN-TYQRF--PKIMKLGR-
XP_005917666      GILKEVVQPGEG--PPVPENA--SVLIHYSGFLEYSDQPFETT-THLKY--PRMMKLGR-
XP_013764145      GILKEVVQPGDG--PPVPENA--SVLIHYSGFLEYSDQPFETT-THLKY--PRMMKLGR-
XP_013764144      GILKEVVQPGDG--PPVPENA--SVLIHYSGFLEYSDQPFETT-THLKY--PRMMKLGR-
CAG01803          GILKEVVQAGDG--PPVPHNA--SVLMHYSAYLDYSNVPFETN-THQRF--PSIMKLGR-
XP_005449738      GILKEVVQPGEG--PPVPENA--SVLIHYSGFLEYSDQPFETT-TNLKY--PRMMKLGR-
KFV20040          --------------------------VKYSGYLEHTGEPFCTT-YNSKS--PELMKLGK-
XP_018551539      GILKEVVHPGEG--PPVPQNA--SVLIHYSGFLEYSDHPFETT-VNFKY--PQMMKLGR-
XP_004542106      GILKEVVQPGEG--PPVPENA--SVLIHYSGFLEYSDQPFETT-THLKY--PQMMKLGR-
XP_005808385      GILKEVVQPGEG--PPVPQNA--SVIMYYSGYLEYSNQPFEST-SHLQY--PPMMKLGR-
KFO85715          --------------------------VKYSGYLEHSDEPFCTN-YHHKV--SKLMKLGR-
XP_015236136      GILKEVVRSGEG--PPVPQNA--SVIMYYSGYLEYSSHPFEST-AHLKY--PPMMKLGR-
XP_023794777      --TRRRYPSIGM--ALKLSQN--HLAVKYSGYLGNWNKLFCSN-GNSKY--PRLMKLGK-
XP_015803473      GILKEVVHPGEG--PPVPLNA--SVLMYYSGFLEYSDQPYESN-TYIKH--PPLMKLGK-
KPP68264          --LAGVTHRLD----LC------VFAVQYSGYLEYSDRPFEST-AHFKH--PQMMKLGT-
XP_020488061      GILKEVIQPGDG--PPVPRNA--SVLIHYSGFLEYSDHPFETT-TNLKY--PQMMKLGR-
XP_015332442      GVLKDVIREGTG--DLVTPDA-SVL-VKYSGYLEHMDKPFDSN-CFRKT--PRLMKLGE-
XP_013764143      GILKEVVQPGDG--PPVPENA--SVLIHYSGFLEYSDQPFETT-THLKY--PRMMKLGR-
XP_005449737      GILKEVVQPGEG--PPVPENA--SVLIHYSGFLEYSDQPFETT-TNLKY--PRMMKLGR-
```

```
KFO61461        --------------------------VKYSGYLGNWNNLFCSN-ESSKY--PRLMKLGK-
XP_008298046    GILKQVVQPGEG--PPVPQNA--SVLIHYSGFLEYSDQPFETT-TNFRF--PQMMKLGR-
KFP07119        --------------------------VNYFGFLEDKDKPFCTH-LDSKS--SKLMKLGT-
XP_007561093    GILKEVVQPGEG--PPVPQNA--SVIMYYSGYLEYSNQPFEST-AHLKY--PPMMKLGR-
PKU35662        GVRKEELRPGSG--RAVPPGA--SVAVKYSGYLEHCDEPFCTN-CYSKS--PRLMKLGK-
XP_010793476    GILKEVIQPGDG--PPVPENA--SVIAHYSGFLEYSDQPFETT-TNLKY--PRMMKLGK-
AWP19851        GILKEVVQSGEG--PPVPQNA--SVLVHYSGFLEYSDRPFDTT-TNFRH--PRMMKLGR-
KFW82601        -----------------PHSS--LCTVKYSGYLGNWNNPFCSY-GHGKY--PKLMKLGR-
XP_008396659    GILKEVVQPGEG--PPVPQNA--SVIMYYSGFLEYSNQPFEST-AHLKY--PPMMKLGR-
XP_010739761    GIMKEVVQPGDG--PPVPQNA--SVLIHYSGFLEYSDRPFETT-THLRY--PRMLKLGR-
XP_022046121    GILKEVVQPGDG--PPVPQNA--SVLMHYSGFLEYSDQPFETT-TNFRY--PPMMKLGR-
XP_019715044    GIFKEVISPGYG--PPVSPSA--SVLIHYSGFLEYSDQPFETT-TRLKH--PRLMKLGR-
XP_018875413    GVLKDVIREGAG--DLVAPDA-SVL-VKYSGYLEHLDRPFDSN-YFRKT--PRLMKLGE-
XP_014327714    GILKEVVQPGEG--PPVPQNA--SVIMYYSGYLEYSNQPFEST-SHLQY--PPMMKLGR-
XP_009081377    ---------------------------KYCGYLKNSTKPFSSN-WNSKY--PKLMKLGR-
AWP19849        GILKEVVQSGEG--PPVPQNA--SVLVHYSGFLEYSDRPFDTT-TNFRH--PRMMKLGR-
XP_023134576    GILKEVVQPGEG--PPVPQNA--SVLIHYSGFLEYSDQPFETT-TNFRY--PVMMKLGR-
XP_012734526    GILKEVVKPGEG--PPVPQNA--SVLMYYSGFLEYSNRPFEST-THFKY--PPMMKLGR-
XP_014403985    ERRRDVTEVRVL--VHFSVSVPSVL-VKYSGYLEHMDKPFDSN-CFRKT--PRLMKLGV-
XP_017553083    GILKEVIRAGDG--SPVPMDA--SVSVHYSGFLEYSDRPFDST-THLKF--PRMVKLGK-
XP_023869472    GILKEVVQPGEG--PPVPRDS--SVSIHFSGFLEYSDRPFETT-SHLKY--PRMMKLGR-
XP_013992804    GILKEVVQPGEG--PPVPRDS--SVSIHFSGFLEYSDRPFETT-SHLKY--PRMMKLGR-
XP_020774925    GIMKQVVQPGEG--PPVPENA--SVIMHYSGFLEYSDQPFETT-LNVKH--PRIMKLGR-
XP_024287709    GILKEVVQPGEG--PPVPRDS--SVSIHFSGFLEYSDRPFETT-SHLKY--PRMMKLGR-
XP_023869475    GILKEVVQPGEG--PPVPRDS--SVSIHFSGFLEYSDRPFETT-SHLKY--PRMMKLGR-
XP_013992805    GILKEVVQPGEG--PPVPRDS--SVSIHFSGFLEYSDRPFETT-SHLKY--PRMMKLGR-
XP_023869476    GILKEVVQPGEG--PPVPRDS--SVSIHFSGFLEYSDRPFETT-SHLKY--PRMMKLGR-
XP_024287708    GILKEVVQPGEG--PPVPRDS--SVSIHFSGFLEYSDRPFETT-SHLKY--PRMMKLGR-
XP_023869474    GILKEVVQPGEG--PPVPRDS--SVSIHFSGFLEYSDRPFETT-SHLKY--PRMMKLGR-
XP_011490382    GVLKEVLHPGEG--PPVPQNA--SVLVHYSGYLEYSDQPFETT-THLKH--PRLLKMGR-
XP_020570387    GVLKEVLHPGEG--PPVPQNA--SVLVHYSGYLEYSDQPFETT-THLKH--PRLLKMGR-
XP_008396657    GILKEVVQPGEG--PPVPQNA--SVIMYYSGFLEYSNQPFEST-AHLKY--PPMMKLGR-
XP_014851597    GILKEVVQPGEG--PPVPQNA--SVIMYYSGYLEYSNQPFEST-AHLKY--PPMMKLGR-
XP_020570386    GVLKEVLHPGEG--PPVPQNA--SVLVHYSGYLEYSDQPFETT-THLKH--PRLLKMGR-
XP_008396658    GILKEVVQPGEG--PPVPQNA--SVIMYYSGFLEYSNQPFEST-AHLKY--PPMMKLGR-
XP_018774793    GVRKEQLRPGTG--QPVPPSA--SVAVKYSGYLGNWNKLFCSN-GNSKY--PRLMKLGK-
XP_023794778    GVRKERLCPGTG--QPVPPSA--SVAVKYSGYLGNWNKLFCSN-GNSKY--PRLMKLGK-
XP_024147377    GIFKEVLHPGEG--PPVPQNA--TVSVHYSGYLEYADQPFETT-TNLLH--PRLLKTGR-
XP_024147375    GIFKEVLHPGEG--PPVPQNA--TVSVHYSGYLEYADQPFETT-TNLLH--PRLLKTGR-
XP_024147378    GIFKEVLHPGEG--PPVPQNA--TVSVHYSGYLEYADQPFETT-TNLLH--PRLLKTGR-
XP_014851599    GILKEVVQPGEG--PPVPQNA--SVIMYYSGYLEYSNQPFEST-AHLKY--PPMMKLGR-
XP_008396660    GILKEVVQPGEG--PPVPQNA--SVIMYYSGFLEYSNQPFEST-AHLKY--PPMMKLGR-
XP_017593619    --YEIKLLLFPF--SPLLALG---CTVKYSGYLGNWNNLFCSN-ESSKY--PRLMKLGK-
XP_008396661    --------------------------MYYSGFLEYSNQPFEST-AHLKY--PPMMKLGR-
XP_014851600    --------------------------MYYSGYLEYSNQPFEST-AHLKY--PPMMKLGR-
XP_019619874    GVVKKVLKQGVG--DVVPAGA--YVRVHYNGYLEYSDEPFDSS-RLRNE--ELRFRLAT-
XP_016801711    ----------------------SIL-VKYSGYLEHLDRPFYSN-YFRKT--PRLMKLGE-
XP_023710706    KIKKREIRPGIG--CVVPEKA--VVTVHYNAYVEYSDEPYDSTWLRG-Y--PMVGRLGQ-
XP_020627099    GVLKQIVRQGTG--PVVPQGA--TVRYHYNGYLEFNDEPFDSS-RLRGR--PDVGKL---
XP_006793207    GILKEVVQPGEG--PPVPENA--SVLIHYSGFLEYSDQPFETT-TNLKY--PRMMKLGR-
XP_011402831    MVFKKIIRPGIG--LSIPEGA--TVKVHYNGYLEYSDEPYDST-RLRNK--PLTIRLGT-
XP_022801008    GVFKRMIRKGTG--PVVPEGA--TVRYHYNGYLEFNDEPFDSS-RLRNK--PEIHKL---
XP_013786360    GVLKKEVKPGIG--PVVSKGA--FVTVHYNSYLEYSEEPFDST-RLRNL--PFKFLLDR-
XP_022254671    GVLKKEVKPGIG--PVVSKGA--FVTVHYNSYLEYSEEPFDST-RLRNL--PFKFLLDR-
ELT98785        GVKKEVLQQGVG--DVVDPKS--YVTVHYNGRFEYSDEPFDST-TLRGQ--PRKFALGE-
XP_021353947    GVVKKILTHGAG--NVIPEGA--IVSIHYNGFLEYSDEPFDSS-RLRNA--PIKKQLGK-
XP_022108900    GVKKRVKKVGSG--EVFQEGW--TATIHYEGYFEYCDVPYDSS-KLRNK--PQKITLGE-
XP_022108899    GVKKRVKKVGSG--EVFQEGW--TATIHYEGYFEYCDVPYDSS-KLRNK--PQKITLGE-
```

```
XP_002167164      LVLKKIMKQGSG--PVIPPNS--LCRVHYNGYIEYSDEPFDSS-RLRGK--QHQFKLGS-
KOF62423          -------------------------SPVHYTGFFEFADEPFDSS-RLRGE--VFKFRLRK-
XP_014790271      SDLRECSEAMF----ELKKSE--IQHVHYTGFFEFADEPFDSS-RLRGE--VFKFRLRK-
XP_001636239      GVLKKIIRQGTG--PVVPKTA--TVRFHSNGYKEFCDEPYDSS-RFRGK--PEQMRLG--
XP_023237057      QIFKKELKTGFG--IELPSKA--FVTIHYNAYLEYSDEPFDST-RLRGT--PLKFVLGE-
XP_002128893      GVKKMIRKEGVG--SVVPSGS--KVRIHYNGYMEDLDEPFDSS-RIRNQ--PLQITLEQ-
XP_009051935      GVMKTVLKPGAG--NVVPAQS--LVRIHYNLYLEGSDEPFDSS-RLRGE--ICKFRLGQ-
XP_012221039      KVMKYIKQKGVG--EVVP--HNAQVTIHYIGYFEHRDEPFDST-YISGR--PKIMRVGQ-
XP_012264923      KVFKAVKKPGVG--DVVP--KDAMVTITYWGYFEYRDEPFDSS-VFRQS--SERIRLNQ-
XP_009857551      GVKKMIRKEGVG--SVVPSGS--KVRIHYNGYMEDLDEPFDSS-RIRNQ--PLQITLEQ-
XP_015515854      KIFKSVKQVGVG--EVVP--MDAMVYVQYMGYFEYQDEPFDSS-YLRQT--SERVRLNK-
XP_011415211      MVFKKVLQQGSG--AIVPEGA--IVRIHYNGYLEFGDEPFDST-RLRNS--PYKVKLQT-
XP_020909274      GVLKKILRPGVG--PVVPRTA--TLRFHYNAYREFGDEPYDSS-RFRGK--PESARLG--
EKC39444          MVFKKVLQQGSG--AIVPEGA--IVRIHYNGYLEFGDEPFDST-RLRNS--PYKVKLQT-
XP_013405452      GVLKQVLKPGTG--DVVPKEA--FVQVHYNGYLEYSGEPFDST-RLRDR--QFRFQLGK-
XP_011148430      KVMKFTKQKGIG--EVVP--YNAQVTVHYIGYFEDNDEPFDSS-YASGK--PRTLRLGQ-
XP_013405451      GVLKQVLKPGTG--DVVPKEA--FVQVHYNGYLEYSGEPFDST-RLRDR--QFRFQLGK-
PVD31214          GVFKKVLRPGAG--PVVPRNS--LVRVHYNAYKEGEEEPFDSS-RLRTE--VDKFHLFR-
PIK39047          GVWKMVVNNGTG--PVVPNGY--RIQVHYNSYLEYSDEPMDST-RLRSE--TKKFILGN-
XP_022336187      MVFKKILQQGTG--NVVPEGA--IVRIHYNGYLEFGDEPFDST-RLRNT--PYKVKLQT-
XP_022337811      MVFKKILQQGTG--NVVPEGA--IVRIHYNGYLEFGDEPFDST-RLRNT--PYKVKLQT-
XP_014787871      GVFKETKHPGYG--PGITENS--VVEVHYAGFFEYRDEPFDSS-RLRDE--TFKYRVGS-
EFN78601          KVMKFTKQKGIG--EVVP--YNAQVTVHYIGYFEDNDEPFDSS-YASGK--PRTLRLGQ-
XP_014475449      KVMKFIKQKGVG--EVIP--NNAQVTIHYLGYLEDNDEPFDST-YICGK--PKTLRLEQ-
XP_014279621      KVSKLILRFGNG--NVVPPKS--VVYFHYNSYLEMEEIPFDSSYLRNEK--PLRMQLGH-
XP_022193504      RIMKRMKKQGMG--EMISNAG--VVYYHYSAYVNLQEEPFDNS-YLRRHRRPERLVLGK-
XP_014204130      EILKLIKTEGHG--ETIP--KDAQVTVEYVGYFEHQDEPFDSTFYMRDK--KAILRLGR-
KOX75315          KIMKLIKQKGVG--NCVP--FDAQVTIKYIGHVEHVDEPFDSS-FARGE--AESFCLNE-
XP_014234164      KILKIIKRDGVG--DVVP--PDAQVTVEYVGYFEYSDQPFDSTLFHPSK--RMVIRLGK-
KII63942          GVLKTIIRRGQG--PVVPENS--VCRVHYSAYIQGDLEPFDST-RARRG--HAMITIGKN
XP_024936423      KVMKVVRQNGVG--EIIP--CNAQVTIKYIGYFEYQDEPFDST-YNNRN--AYTLNLSC-
XP_015586170      KVMKVVRQNGVG--EIIP--CNAQVTIKYIGYFEYQDEPFDST-YNNRN--AYTLNLSC-
XP_003486736      KVMKLIKQEGVG--TVIP--CDAQVSIKYIAHFEFSDEPFDSS-FAHGD--TETLHLGE-
XP_019882220      KVMKYVKQKGVG--EVVP--ANGQITFHYIGYFEDRDEPFDST-YSSGK--PRTLRLGQ-
XP_003402471      KVMKLIKQEGVG--TVIP--CDAQVSIKYIAHFEFSDEPFDSS-FAHGD--TETLHLGE-
XP_012134979      KVMKLIKQKGVG--EPVP--YDAQVTVKYIGHFEYRDEPFDSS-FTRGG--TDTFCLNQ-
XP_017756606      KVMKLIKQKGVG--NVVP--HDAQVTIKYVGHFEYRDEPFDSS-FIRGE--AETFYLNQ-
XP_012134978      KVMKLIKQKGVG--EPVP--YDAQVTVKYIGHFEYRDEPFDSS-FTRGG--TDTFCLNQ-
XP_003700792      KVMKLIKQKGVG--EPVP--YDAQVTVKYIGHFEYRDEPFDSS-FTRGG--TDTFCLNQ-
XP_023290805      QVLKLVKKEGVG--ETMP--YNSQVTVHYIGYLEHQDDPFDST-YIRGK--PAVLRLNE-
XP_012270499      QVLKLVKKEGVG--ETMP--YNSQVTVHYIGYLEHQDDPFDST-YIRGK--PAVLRLNE-
XP_018309319      KVMKYVKQRGVG--EIIP--NNAQAIVHYIGYFEYRDEPFDST-YSIGK--PRSLRLGQ-
XP_012062619      KVMKYVKQKGAG--ELIP--NNAQAIIHYIGYFEYRDEPFDST-YSVGK--PRSLRLGQ-
XP_012527508      KVMKLVKQKGVG--DVVP--DNALATVHYIGYFEYRDEPFDST-YSSGK--PRSLRLGK-
XP_005089179      GVLKEMKTPGIG--GLVAEGA--LVSFHYNAYTDVGQAPYDSSRLRD-Q--YSKLRLGT-
XP_003424555      EILKLIKKEGIG--EVIP--KDAQATVEYVGYFEFQDEPFDSTLFHREK--RATLNLGK-
XP_018055399      KVMKYVKQKGTG--ELIP--NNAQAIIHYIGYFEYRDEPFDST-YSVGK--PRSLRLGQ-
XP_017877748      KIMKLIKKEGIG--NTVP--KDAQVTVKYIGYFEYRDDPFDSS-FTRGG--SDILRLGQ-
XP_018399012      KVMKYVKQKGVG--VIIP--ENAQAIVHYIGYFEYRDEPFDST-YSVGR--PRSLRLGQ-
KYM99352          KVMKYVKQKGVG--VIIP--ENAQAIVHYIGYFEYRDEPFDST-YSVGR--PRSLRLGQ-
XP_018309322      VVMKYVKQRGVG--EIIP--NNAQAIVHYIGYFEYRDEPFDST-YSIGK--PRSLRLGQ-
XP_011647883      KVMKFVKQKGIG--EVVP--CNAQITIHYIGYFEYRDEPFDST-YSTGN--PRTLRLGQ-
XP_014247948      KVKKKILRQG-T--VQLPPNA--AVYIQYNSYLELEEVPFDSSYLRSKH--PFRLQLGL-
XP_011064230      KVMKYVKQKGVG--KIIP--PNAQAIVHYVGYFEYRDEPFDSA-YCVGK--PRSLRLGQ-
XP_014613253      KIMKLIKRKGAG--AMVS--FKSQVTIKYISFFEYSDEPFDST-FAQGK--PKTIRLDH-
XP_002430548      FVKKQIIVCGIG--AVVPENS--YVTVHYNLYKEHHDLPFDSTYFRN-K--PEKFRLDQ-
XP_014247946      KVKKKILRQG-T--VQLPPNA--AVYIQYNSYLELEEVPFDSSYLRSKH--PFRLQLGL-
XP_014270488      EIKKMILRNGRG--ELVSSDA--VVYLHYNCYLEMQDIPFDSSYQRN-K--PLHIQMGT-
```

ORY30517	KLRKRIIQEGTGD-LISR-YS--DVSVHYTGRLHPSGTKFDSS-LDRNK--PLEFKVSE-
XP_011330216	KVMKYIKQKGTG--KGVP--DNAQVTVHYIGYFEYRDEPFDSS-YSSGK--PRVLRLNE-
EGI60945	KVMKYVKQKGVG--EIIP--NNAQAIVHYIGYFEYRDEPFDST-YSVGR--PRALRLGQ-
XP_001201219	KVLKSLLKQGTG--ALPIVGM--TLTVHYNCYVEYSDEPYDST-RLRNR--PERCKLGA-
XP_011061927	KVMKYVKQKGVG--EIIP--NNAQAIVHYIGYFEYRDEPFDST-YSVGR--PRALRLGQ-
XP_018356457	KVMKYIKQKGVG--EIIP--NNAQAIVHYIGYFEYRDEPFDST-YSVGR--PRSLRLGQ-
XP_011173578	KVMKFVKQKGVG--EVIP--SNAHATVHYIGYFEYRDEPFDST-YSCGK--PRSLRLGQ-
XP_018356456	KVMKYIKQKGVG--EIIP--NNAQAIVHYIGYFEYRDEPFDST-YSVGR--PRSLRLGQ-
XP_016917008	KVMKLIKQKGVG--SVVP--YDAQITIKYIGHFEYRDEPFDSS-FARGG--AETFRLNQ-
XP_018374414	KVMKYVKQKGVG--VIIP--NNAQAIVHYIGYFEYRDEPFDST-YAIGK--PRSLRLGQ-
XP_016917007	KVMKLIKQKGVG--SVVP--YDAQITIKYIGHFEYRDEPFDSS-FARGG--AETFRLNQ-
XP_019882221	TVMKYVKQKGVG--EVVP--ANGQITFHYIGYFEDRDEPFDST-YSSGK--PRTLRLGQ-
XP_011191641	YIYKKITQPGVAE-AGEVPL-NARVCIRYNAYWESEGAPFDSS-FLRGS--SYHFYTGRN
PFH33404	GVFKKILKEGDG--PTPQPGE--EVVVHYTGTLKD-GTKFDSS-RDRNA--PFKFVIGE-
XP_019876143	GVKKRVLREGQG--ETPASMA--LVKVDYNAYIEFEPTPFDSTYARR-K--SLKFVVNN-
XP_014299498	KIKKLILKNGIG--ETVA--KDSQIVIKYSGYLEGQDNPFDST-YQRHC--ADRYRLNR-
KOC63519	NIMKLIKQNGIG--EIIP--HDAQVSIKYIGHFEYRDEPFDSS-FTRGG--VETFCLNV-
XP_015126020	KVKKLIRQKGVG--PVIP--PDALVTIKYSGYFEGEDEPYDSS-HARGN--PDRYQLND-
XP_005186588	NMWKKITKVGDES-EGVVPPVNARACIRYNAYWEGEGTPFDST-FLRGS--SYSFYTGRN
XP_011879049	KVMKFIKQNGVG--EIIP--QDAQVSVHYLGNLEYRDEPFDST-YSSGK--PRTLRLNQ-
XP_013109471	YIWKKITKTGNEE-ESVVPAVNARACIRYNAYWEGEGAPFDSS-FLRGS--SHSFYTGRN
XP_011879048	KVMKFIKQNGVG--EIIP--QDAQVSVHYLGNLEYRDEPFDST-YSSGK--PRTLRLNQ-
XP_018333359	KVKKRILRKGYG--DTLPDLC--QVTIHYNAYIEFQDEPFDSTYARK-R--PYTFRLNN-
EFZ09302	--MKFVKQKGVG--EVIP--SNAHATVHYIGYFEYRDEPFDST-YSCGK--PRSLRLGQ-
XP_016927793	FMHKRTTREGHVD-RDVVPD-KARVSVRYSFYWEGMGAPFDSS-MLRGT--KFEFETGQK
XP_006611831	KVMKLIKQKGVG--SIVP--YDAQITIKYIGHFEYRDEPFDSS-FARGG--AETFRLNQ-
XP_014245088	SLRKKIIRHG-T--DKVPSYA--AVYIHYNSYLELEEVPFDSSFLRSEK--PVRFQLGA-
XP_018790058	HIYKKITRPGLAE-NGEVPL-NARVCIRYNAYWEGEGAPFDSS-FLRGS--SYQFYTGRN
XP_020893545	GVLKKILRPGVG--PVVSRTA--TLRFHDNAYREFGDEPYDSS-RFRGK--PESARLG--
XP_002092689	HIYKRITRTGHVD-RDAVPI-KARVSVRYSGYWEGESAPFDSS-LLRGT--KFEFETGQG
XP_020293583	KVMKYVKKNGVE--ESVSNAHNPQLTFHYVAYYEHGDTPFDST-YAAGR--PRCLRLN--
XP_016952968	FMYKRVTREGHVD-RDLVPD-KARVSVRYSFYWEGMGAPFDSS-MLRGT--KFEFETGQK
XP_003689972	KIMKLIKQKGVG--STVP--YDAQITIKYIGHFEYRDEPFDSS-FARGG--AETFRLNQ-
XP_015185328	KIMKLIKRKGVG--ATVS--YNSQVTIKYISYFEYSDEPFDST-FAQGK--PKTLRLDH-
XP_024867102	KVMKYVKQKGVG--EVVP--QNAQVTVNYIGYFEYRDEPFDST-YSYGR--PRSMRLGQ-
XP_011130643	KVVKTILLEGSG--ETPAVGC--QVKVHYTGTLTD-GKKFDSS-IDRGT--PFEFALGQ-
XP_011206843	HIYKKITQPGLAE-SGEVPL-NARVCIRYNAYWEGEGAPFDSS-FLRGS--SYQFYTGRN
XP_008560701	KIKKLVLQKGIG--EIIP--ADSQVTIKYSGYLEGQDNPFDST-YQRHS--VNRCRLNQ-
XP_011695993	KVMKFVKQKGVG--EVIP--KNAQVTVHYVGYFEYRDEPFDST-YSSGR--PRTLRLGQ-
PIK55877	----MVVNNGTG--PVVPNGY--RIQVHYNSYLEYSDEPMDST-RLRSE--TKKFILGN-
XP_022218357	NIFKRITREGHQD-RGLVPD-KARVAVRYSGYWEGENSPFDSS-LMRRS--KFYFETGAG
XP_017120033	HIYKRITREGHVD-RDLVPE-KARVSIRYSGYWEGEGHPFDSS-MLRGT--KFEFETGQK
XP_022165811	KVLKILLEPGIG--QVVPENY--VVLIHYIAYISNLQEPFDVTYLQGRK--PKRFTLGN-
XP_017070534	HIYKRIIREGHVE-RDMVPD-KARVTVRYSGYWEGEGAPFDSS-MLRGT--KFEFETGQR
XP_001942690	KVLKILLNSGIG--QVVPENY--VVLIHYIAYISNLQEPFDVTYLQGRR--PKRFTLGN-
XP_003883131	GVFKKILKEGDG--PTPQPGE--EVVVHYTGTLLD-GTKFDSS-RDRDS--PFKFIIGE-
XP_014091483	YIYKKITQPGLAE-NGEVPL-NARVCIRYNAYWEGEGAPFDSS-FLRGS--SYHFYTGRN
XP_015376375	KVLKILLNQGIG--QVVPDNY--VVLIHYIAYISNLQEPFDVTYLQGRK--PKRFTLGN-
KOF66856	GVFKETKHPGYG--PGITENS--VVEVHYAGFFEYRDEPFDSS-RLRDE--TFKYRVGS-
XP_015431627	KIMKLLKQKGVG--EIIP--SNAQVTIKYMGHFEYNDEPFDSS-FTRGG--AETFLLGQ-
ORX48915	GVKKKIIKEGTGD-FPPA-GS--KVTVHYDGYLYPSGDEFDSS-RKRHS--PFTFNLSK-
XP_001958659	YIYKRVIREGHPE-REVVPE-RARVAVRYSGYWEGEGAPFDSS-MLRGT--QFEFVTGQC
XP_013077159	GVLKQMKKPGFG--KTVPEKA--LVQIHYNAYSEVGQPPYDSTRMRG-E--PMKLRLGQ-
XP_013077158	GVLKQMKKPGFG--KTVPEKA--LVQIHYNAYSEVGQPPYDSTRMRG-E--PMKLRLGQ-
XP_016974015	YIYKRITREGHVE-RDLVPE-KARVSIRYSGYWEGDSHPFDSS-MLRGT--KFEFETGQK
KFG33606	GVFKKILKEGDG--PQPQPGE--EVVVHYTGTLLD-GTKFDSS-RDRDS--PFKFIIGE-
XP_017045419	FIYKRITREGHVD-RDLVPQ-KARVSVRYSGYWEGEGAPFDSS-MLRGA--KFEFETGQN
PUA87907	GVFKKILKEGDG--PQPQPGE--EVVVHYTGTLLD-GTKFDSS-RDRDS--PFKFIIGE-

XP_017001929	YMYKRITREGHVE-REVVPE-KARVSVRYSFYWEGSGAPFDSS-MLRGT--KFEFETGQK
XP_009012598	GIKKLLIKRGTG--PIVPPTS--NVTIMYSAYTEYSDVPFD----FTRR--PMHMDIG--
XP_008888049	GVFKKILKEGDG--PQPQPGE--EVVVHYTGTLLD-GTKFDSS-RDRDS--PFKFIIGE-
XP_023297605	HVWKKITKVGLEQ-EPVVPN-NARVCIRYNAYWEGEGAPFDSS-FLRGS--SYSFYTGRN
XP_002594856	GVVKKVVKQGVG--DVVPAGA--YVRVHYNGYLEYSDEPFDSS-RLRNE--ELRFRLAT-
XP_018896035	GVMKRIIKEGSG--EVVPPNS--HIKIHYQAYTEYQEVPFDVSILRSKK--PLSFKLG--
XP_004517316	HIYKKITQPGLPE-EGEVPL-NARVCIRYNAYWEGEGAPFDSS-FLRGS--SYHFYTGRN
XP_017148191	NIFKRITREGHQG-RGLVPD-KARVAVRYSGYWEGESSPFDSS-LMRRT--KLYFETGAG
XP_002412109	GVLKKVLHPGVG--AVVPSDS--CVTFHYSAYLEMSDEPFDST-RMRNR--PHRSLLC--
XP_014507068	GLKKKLLKEGEGW-DRPNNGD--QVEVHYTGTLLD-GTQFDSS-RDTGT--PFKFNLGQ-
XP_011298074	KIKKIIRQKGVG--SVIP--PDCLVTIKYAGYFDGQDEPYDSS-HARGK--PDRYQLGV-
ESS30489	GVFKKILKEGDG--PQPQPGE--EVVVHYTGTLLD-GTKFDSS-RDRDS--PFKFIIGE-
EPR59296	GVFKKILKEGDG--PQPQPGE--EVVVHYTGTLLD-GTKFDSS-RDRDS--PFKFIIGE-
OAJ38225	HVCKKLLSEGSGD-LIDRPGT--TMRVHYTGSLYPSGEVFDSS-LDRDE--PIQIRLGQ-
XP_018637740	GVFKKILKEGDG--PQPQPGE--EVVVHYTGTLLD-GTKFDSS-RDRDS--PFKFIIGE-
KYK63140	GVFKKILKEGDG--PQPQPGE--EVVVHYTGTLLD-GTKFDSS-RDRDS--PFKFIIGE-
CEL73032	GVFKKILKEGDG--PQPQPGE--EVVVHYTGTLLD-GTKFDSS-RDRDS--PFKFIIGE-
AAX51680	GVFKKILKEGDG--PQPQPGE--EVVVHYTGTLLD-GTKFDSS-RDRDS--PFKFIIGE-
AFJ24780	LIFKRLISKGEG--SKIPIGS--LVIYNYKFYHEDQIEPFDST-RFKRH--PDRQRVGV-
NP_611837	NIYKRITRTGHVD-REAVPN-KARVSVRYSGYWEGETAPFDSS-LLRGS--KFVFETGQG
XP_012342118	KIMKLIKQKGVG--STVP--YDAQITIKYIGHFEYRDEPFDSS-FARGG--AETFRLNQ-
XP_002082854	NIYKRITRTGHVD-REAVPN-KARVSVRYSGYWEGETAPFDSS-LLRGT--KFEFETGQG
XP_002040150	NIYKRITRTGHVD-REAVPN-KARVSVRYSGYWEGETAPFDSS-LLRGT--KFEFETGQG
XP_021954336	GIKKQVMKEGSE-TELIPPEG--YIWMHYITFVEGNEEPIDST-GSREL--LGSHKLGK-
XP_001357527	NIFKRITREGHQG-RGLVPD-KARVAVRYSGYWEGESSPFDSS-LMRRN--KFYFETGAG
XP_002015409	NIFKRITREGHQG-RGLVPD-KARVAVRYSGYWEGESSPFDSS-LMRRN--KFYFETGAG
XP_017469679	HIYKKITQPGLTE-YGEVPL-NARVCIRYNAYWEGEGAPFDSS-FLRGS--SYHFYTGRN
XP_020810045	DIYKRIIRQGHAE-RDPVPD-KARVSIRYSGYWEGVGAPFDSS-LLRGT--KFEFETGTK

NP_003593	DITLWGMELGLLSMRRGELARFLFKPNYAYGTLGCPPLIPPNTTVLFEIELLDF
XP_003815624	DITLWGMELGLLSMRRGELARFLFKPNYAYGTLGCPPLIPPNTTVLFEIELLDF
XP_003777068	DITLWGMELGLLSMRRGELARFLFKPNYAYGTLGCPPLIPPNTTVLFEIELLDF
XP_003276647	DITLWGMELGLLSMRRGELARFLFKPNYAYGTLGCPPLIPPNTTVLFEIELLDF
XP_003895916	DITLWGMELGLLSMRRGELARFLFKPNYAYGTLGCPPLIPPNTTVLFEIELLDF
EHH17242	DITLWGMELGLLSMRRGELARFLFKPNYAYGTLGCPPLIPPNTTVLFEIELLDF
XP_001110932	DITLWGMELGLLSMRRGELARFLFKPNYAYGTLGCPPLIPPNTTVLFEIELLDF
XP_011785750	DITLWGMELGLLSMRRGELARFLFKPNYAYGTLGCPPLIPPNTTVLFEIELLDF
NP_001128683	DITLWGMELGLLSMRRGELARFLFKPNYAYGTLGCPPLIPPNTTVLFEIELLDF
XP_017713171	DITLWGMELGLLSMRRGELARFLFKPNYAYGTLGCPPLIPPNTTVLFEIELLDF
XP_010385752	DITLWGMELGLLSMRRGELARFLFKPNYAYGTLGCPPLIPPNTTVLFEIELLDF
XP_002817917	DITLWGMELGLLSMRRGELARFLFKPNYAYGTLGCPPLIPPNTTVLFEIELLDF
XP_004045599	DITLWGMELGLLSMRRGELARFLFKPNYAYGTLGCPPLIPPNTTVLFEIELLDF
XP_008979905	DITLWGMELGLLSMRRGELARFLFKPTYAYGTLGCPPLIPPDTTVLFEIELLDF
XP_016868230	DITLWGMELGLLSMRRGELARFLFKPNYAYGTLGCPPLIPPNTTVLFEIELLDF
XP_003794239	DITLWGMELGLLSMRRGELARFLFKPTYAYGTLGCPPLIPPNTTVLFEIELLDF
XP_012597698	DITLWGMELGLLSMRRGELARFLFRPNYAYGTLGCPPLIPPNATVLFEIELLDF
XP_011931834	DITLWGMELGLLSMRRGELARFLFKPNYAYGTLGCPPLIPPNTTVLFEIELLDF
XP_003895917	DITLWGMELGLLSMRRGELARFLFKPNYAYGTLGCPPLIPPNTTVLFEIELLDF
XP_008016547	DITLWGMELGLLSMRRGELARFLFKPNXAYGTLGCPPLIPPNTTVLFEIELLDF
XP_003934167	DITLWGMELGLLSMRRGELARFLFKPTYAYGTLGCPPLIPPDTTVLFEIELLDF
XP_012495716	DITLWGMELGLLSMRRGELARFLFKPNYAYGTLGCPPLIPPNATVLFEIELLNF
XP_012304459	DITLWGMELGLLSMRRGELARFLFKPTYAYGTLGCPPLIPPDTTVLFEIELLDF
XP_022357643	DITLWGMELGLLSMRRGELARFLFKPTYAYGALGCPPLIPPNTTVLFEIELLDF
XP_010385754	DITLWGMELGLLSMRRGELARFLFKPNYAYGTLGCPPLIPPNTTVLFEIELLDF
XP_017895560	DITLWGMELGLLSMRRGELARFLFKPTYAYGTLGCPPLIPPNTTVLFEIELLDF
XP_005892900	DITLWGMELGLLSMRRGELARFLFKPTYAYGTLGCPPLIPPNTTVLFEIELLDF
XP_010852603	DITLWGMELGLLSMRRGELARFLFKPTYAYGTLGCPPLIPPNTTVLFEIELLDF

XP_546928	DITLWGMELGLLSMRRGELARFLFKPTYAYGTLGCPPLIPPNTTVLFEIELLDF
XP_005225237	DITLWGMELGLLSMRRGELARFLFKPTYAYGTLGCPPLIPPNTTVLFEIELLDF
XP_021556246	DITLWGMELGLLSMRRGELARFLFKPTYAYGTLGCPPLIPPNTTVLFEIELLDF
XP_011978492	DITLWGMELGLLSMRRGELARFLFKPTYAYGTLGCPPLIPPNTTVLFEIELLDF
NP_001296390	DITLWGMELGLLSMRRGELARFLFKPTYAYGTLGCPPLIPPNTTVLFEIELLDF
XP_011959859	DITLWGMELGLLSMRRGELARFLFKPTYAYGTLGCPPLIPPNTTVLFEIELLDF
XP_006942097	DITLWGMELGLLSMRRGELARFLFKPAYAYGTLGCPPLIPPNTTVLFEIELLDF
XP_007100028	DITLWGMELGLLSMRRGELARFLFKPTYAYGMLGCPPLIPPNSTVLFEIELLDF
XP_005972253	DITLWGMELGLLSMRRGELARFLFKPTYAYGTLGCPPLIPPNATVLFEIELLDF
XP_019277206	DITLWGMELGLLSMRRGELARFLFKPAYAYGTLGCPPLIPPNTTVLFEIELLDF
XP_014705926	DITLWGMELGLLSMRRGELARFLFKPTYAYGTLGCPPLIPPNTTVLFEIELLDF
XP_008540619	DITLWGMELGLLSMRRGELARFLFKPTYAYGTLGCPPLIPPNTTVLFEIELLDF
XP_020752493	DITLWGMELGLLSMRRGELARFLFKPTYAYGTLGCPPLIPPNTTVLFEIELLDF
XP_014988800	DITLWGMELGLLSMRRGELARFLFKPNYAYGTLGCPPLIPPNTTVLFEIELLDF
XP_011785755	DITLWGMELGLLSMRRGELARFLFKPNYAYGTLGCPPLIPPNTTVLFEIELLDF
XP_022444356	DITLWGMELGLLSMRRGEVARFLFKPTYAYGMLGCPPLIPPNTTVLFEIELLDF
XP_008979887	DITLWGMELGLLSMRRGELARFLFKPTYAYGTLGCPPLIPPDTTVLFEIELLDF
XP_004399150	DITLWGMELGLLSMRRGELARFLFTPPYAYGTLGCPPLIPPNTTVLFEIELLDF
XP_004268847	DITLWGMELGLLSMRRGEVARFLFKPTYAYGMLGCPPLIPPNATVLFEIELLDF
XP_019659608	DITLWGMELGLLSMRRGELARFLFKPTYAYGTLGCPPLIPPNTTVLFEIELLDF
XP_024602986	DITLWGMELGLLSMRRGEVARFLFKPTYAYGMLGCPPLIPPNTTVLFEIELLDF
XP_007186614	DITLWGMELGLLSMRRGELARFLFKPTYAYGMLGCPPLIPPNTTVLFEIELLDF
XP_014652016	DITLWGMELGLLSMRRGELARFLFKPTYAYGTLGCPPLIPPNTTVLFEIELLDF
XP_012597701	DITLWGMELGLLSMRRGELARFLFRPNYAYGTLGCPPLIPPNATVLFEIELLDF
XP_004761711	DITLWGMELGLLSMRRGELARFLFKPTYAYGTMGCPPLIPPNTTVLFEIELLDF
XP_004317177	DITLWGMELGLLSMRRGEVARFLFKPTYAYGMLGCPPLIPPNATVLFEIELLDF
XP_005892901	DITLWGMELGLLSMRRGELARFLFKPTYAYGTLGCPPLIPPNTTVLFEIELLDF
XP_017895559	DITLWGMELGLLSMRRGELARFLFKPTYAYGTLGCPPLIPPNTTVLFEIELLDF
XP_005662030	DITLWGMELGLLSMRRGELARFLFKPTYAYGTLGCPPLIPPNTTVLFEIELLDF
XP_010852604	DITLWGMELGLLSMRRGELARFLFKPTYAYGTLGCPPLIPPNTTVLFEIELLDF
XP_010852602	DITLWGMELGLLSMRRGELARFLFKPTYAYGTLGCPPLIPPNTTVLFEIELLDF
EPY83956	DITLWGMELGLLSMRKGELARFLFTPTYAYGTLGCPPLIPPNTTVLFEIELLDF
NP_001095679	DITLWGMELGLLSMRRGELARFLFKPTYAYGTLGCPPLIPPNTTVLFEIELLDF
XP_008139950	DITLYGMELGLLTMRRGELARFLFKPPYAYGTLGCPPLIPPNSTVLFEIELIDF
XP_010970644	DITLWGMELGLLSMRKGELARFLFTPTYAYGTLGCPPLIPPNTTVLFEIELLDF
XP_004021020	DITLWGMELGLLSMRRGELARFLFKPTYAYGTLGCPPLIPPNTTVLFEIELLDF
XP_015450070	DITLWGMELGLLSMRRGELARFLFKPTYAYGTLGCPPLIPPNTTVLFEIELLDF
XP_017516100	DITLWGMELGLLSMQRGELARFLFKPSYAYGTLGCPPLIPANTTVLFEIELLDF
XP_010342933	DITLWGMELGLLSMRRGELARFLFKPTYAYGTLGCPPLIPPDTTVLFEIELLDF
XP_006057167	DITLWGMELGLLSMRRGELARFLFKPTYAYGMLGCPPLIPPNTTVLFEIELLDF
XP_019843968	DITLWGMELGLLSMRRGELARFLFKPTYAYGTLGCPPLIPPNTTVLFEIELLDF
XP_006201442	DITLWGMELGLLSMRKGELARFLFTPTYAYGTLGCPPLIPPNTTVLFEIELLDF
XP_005972252	DITLWGMELGLLSMRRGELARFLFKPTYAYGTLGCPPLIPPNATVLFEIELLDF
XP_004380900	DITLWGMELGLLSMRKGELARFLFKPTYAYGTLGCPPLIPPNTTVLFEIELLDF
XP_007468850	DITLWGMELGLLSMRRGELARFLFKPTYAYGMLGCPPLIPPNTTVLFEIELLDF
XP_007100027	DITLWGMELGLLSMRRGELARFLFKPTYAYGMLGCPPLIPPNSTVLFEIELLDF
XP_008966786	DITLWGMELGLLSMRRGELARFLFKPNYAYGTLGCPPLIPPNTTVLFEIELLDF
XP_020017458	DITLWGMELGLLSMRRGELARFLFKPTYAYGTLGCPPLIPPNSTILFEIELLDF
XP_006088351	DITLWGMELGLLTMRRGELARFLFKPPYAYGTLGCPPLIPPNSTVLFEIELIDF
XP_020752496	DITLWGMELGLLSMRRGELARFLFKPTYAYGTLGCPPLIPPNTTVLFEIELLDF
XP_022444355	DITLWGMELGLLSMRRGEVARFLFKPTYAYGMLGCPPLIPPNTTVLFEIELLDF
XP_006773483	DITLWGMELGLLTMRRGELARFLFKPPYAYGTLGCPPLIPPNSTVLFEIELIDF
XP_012304461	DITLWGMELGLLSMRRGELARFLFKPTYAYGTLGCPPLIPPDTTVLFEIELLDF
XP_008591583	DITLRGMELGLLSMRRGELARFLFRPSYAYGALGCPPLFPPNTTVLFEIELLDF
XP_016054558	DITLWGMELGLLSMRRGELARFLFKPTYAYGTLGCPPLIPPKATVLFEIELIDF
XP_011369980	DITLWGMELGLLSMRRGELARFLFKPTYAYGTLGCPPLIPPNTTVLFEIELLDF
XP_004268849	DITLWGMELGLLSMRRGEVARFLFKPTYAYGMLGCPPLIPPNATVLFEIELLDF
XP_024602987	DITLWGMELGLLSMRRGEVARFLFKPTYAYGMLGCPPLIPPNTTVLFEIELLDF

XP_019610492	DITLWGMELGLLSMRRGEVARFLFKPTYAYGKLGCPPLIPPNTTVLFEIELLDF
XP_004317179	DITLWGMELGLLSMRRGEVARFLFKPTYAYGMLGCPPLIPPNATVLFEIELLDF
XP_006889852	DITLWGMELGLLSMRKGELARFLFKPSYAYGTLGCPPMIPPNSTVLFEIELLDF
XP_007940604	DITLWGMELGLLSMRRGELARFLFRPAYAYGTLGCPPLIPPNSTVLFEIELLDF
XP_014411577	DITLWGMELGLLSMRKGELARFLFTPTYAYGTLGCPPLIPPNTTVLFEIELLDF
XP_007073943	DITLWGMELGLLSMRRGELARFLFKPAYAYGTLGCPPLIPPNTTVLFEIELLDF
XP_006155025	DITLWGMELGLLTMRRGELARFLFKPPYAYGTLGCPPLIPPNTTVLFEIELLDF
XP_005328656	DITLWGMELGLLSMRRGELARFLFKPTYAYGTLGCPPLIPPNTTVLFEIELLDF
XP_011765609	DITLWGMELGLLSMRRGELARFLFKPNYAYGTLGCPPLIPPNTTVLFEIELLDF
XP_010979058	DITLWGMELGLLSMRKGELARFLFTPTYAYGTLGCPPLIPPNTTVLFEIELLDF
XP_006249193	DITLWGMELGLLSMRRGELARFLFKPTYAYGTLGCPPLIPPNATVLFEIELIDF
XP_002722023	DITLWGMELGLLSMRRGELARFLFTPTYAYGALGCPPLIPPNTTVLFEIELLDF
XP_005344683	DITLWGMELGLLSMRRGELARFLFKPTYAYGTLGCPPLIPPNATVLFEIELIDF
XP_020941978	DITLWGMELGLLSMRRGELARFLFKPTYAYGTLGCPPLIPPNTTVLFEIELLDF
XP_006859898	DITLWGMELGLLTMRRGELARFLFKPTYAYGMLGCPPLIPPNTTVLFEIELLDF
XP_005549416	DITLWGMELGLLSMRRGELARFLFKPNYAYGTLGCPPLIPPNTTVLFEIELLDF
XP_024427494	DITLWGMELGILSMRRGELARFLFKPTYAYGTLGCPPLIPPNSTVLFEIELLDF
XP_016001971	DITLWGMELGLLSMRRGELARFLFKPTYAYGTLGCPPLIPRDATVLFEIELLDF
XP_007468849	DITLWGMELGLLSMRRGELARFLFKPTYAYGMLGCPPLIPPNTTVLFEIELLDF
XP_014988802	DITLWGMELGLLSMRRGELARFLFKPNYAYGTLGCPPLIPPNTTVLFEIELLDF
XP_012584338	DITLWGMELGLLSMRKGELARFLFTPTYAYGTLGCPPLIPPNTTVLFEIELLDF
XP_006971358	DITLWGMELGLLSMRRGELARFLFKPTYAYGTLGCPPLIPPNATVLFEIELIDF
XP_004620930	DITLWGMELGLLTMRRGELARFLFTPTYAYGALGCPPLIPPNTTVLFEIELLDF
XP_008696858	DITLWGMELGLLSMRRGELARFLFKPTYAYGTLGCPPLIPPNTTVLFEIELLDF
XP_021018812	DITLWGMELGLLSMRRGELARFLFKPAYAYGTLGCPPLIPPNATVLFEIELIDF
XP_012782940	DITLWGMELGLLSMRRGELARFLFTPNYAYGALGCPPLIPPNSTVLFEIELLDF
XP_019502388	DITLWGMELGLLSMRRGELARFLFKPAYAYGKLGCPPLIPPNTTVLFEIELLDF
XP_021042681	DITLWGMELGLLSMRRGELARFLFKPAYAYGTLGCPPLIPPNATVLFEIELIDF
NP_001264821	DITLWGMELGLLSMRKGELARFLFKPAYAYGTLGCPPLIPPNATVLFEIELIDF
XP_021519217	DITLWGMELGLLSMRRGELARFLFKPTYAYGTLGCPPLIPPNATVLFEIELIDF
XP_005344682	DITLWGMELGLLSMRRGELARFLFKPTYAYGTLGCPPLIPPNATVLFEIELIDF
NP_001349718	DITLWGMELGLLSMRRGELARFLFKPNYAYGTLGCPPLIPPNTTVLFEIELLDF
NP_001099392	DITLWGMELGLLSMRRGELARFLFKPTYAYGTLGCPPLIPPNATVLFEIELIDF
XP_005396843	DITLWGMELGLLSMRKGELARFLFKPTYAYGALGCPPLIPPNATVLFEIELLDF
XP_019502390	DITLWGMELGLLSMRRGELARFLFKPAYAYGKLGCPPLIPPNTTVLFEIELLDF
XP_006971357	DITLWGMELGLLSMRRGELARFLFKPTYAYGTLGCPPLIPPNATVLFEIELIDF
XP_008966787	DITLWGMELGLLSMRRGELARFLFKPNYAYGTLGCPPLIPPNTTVLFEIELLDF
XP_003500991	DITLWGMELGLLSMRRGELARFLFKPTYAYGTLGCPPLIPPNATVLFEIELIDF
XP_008846596	DITLCGMELGLLSMRKGELARFLFRPTYAYGTLGCPPLIPPNATVLFEIELLDF
XP_021563404	DITLWGMELGLLSMRRGELARFLFKPTYAYGALGCPPRIPPNATVLFEIELLDF
XP_018887307	DITLWGMELGLLSMRRGELARFLFKPNYAYGTLGCPPLIPPNTTVLFEIELLDF
XP_021018806	DITLWGMELGLLSMRRGELARFLFKPAYAYGTLGCPPLIPPNATVLFEIELIDF
XP_017176678	DITLWGMELGLLSMRKGELARFLFKPAYAYGTLGCPPLIPPNATVLFEIELIDF
NP_291049	DITLWGMELGLLSMRKGELARFLFKPAYAYGTLGCPPLIPPNATVLFEIELIDF
XP_021042679	DITLWGMELGLLSMRRGELARFLFKPAYAYGTLGCPPLIPPNATVLFEIELIDF
XP_010617304	DITLWGMELGLLSMRKGELARFLFKPPYAYGALGCPPLIPPNATVLFEIELLDF
XP_021519216	DITLWGMELGLLSMRRGELARFLFKPTYAYGTLGCPPLIPPNATVLFEIELIDF
XP_023420891	DITLWGMELGLLTMRKGELARFLFKPTYAYGALGCPPLIPPNATVLFEIELLDF
XP_006155027	DITLWGMELGLLTMRRGELARFLFKPPYAYGTLGCPPLIPPNTTVLFEIELLDF
XP_011765605	DITLWGMELGLLSMRRGELARFLFKPNYAYGTLGCPPLIPPNTTVLFEIELLDF
XP_015302699	DITLWGMELGLLSMRRGELARFLFKPNYAYGTLGCPPLIPPNTTVLFEIELLDF
XP_011765611	DITLWGMELGLLSMRRGELARFLFKPNYAYGTLGCPPLIPPNTTVLFEIELLDF
XP_012597702	DITLWGMELGLLSMRRGELARFLFRPNYAYGTLGCPPLIPPNATVLFEIELLDF
XP_014988803	DITLWGMELGLLSMRRGELARFLFKPNYAYGTLGCPPLIPPNTTVLFEIELLDF
XP_010617307	DITLWGMELGLLSMRKGELARFLFKPPYAYGALGCPPLIPPNATVLFEIELLDF
XP_004666244	DITLWGMELGLLSMRKGELARFLFKPTYAYGALGCPPLIPPSATVLFEIELLDF
XP_004630630	DITLWGMELGLLSMRRGELARFLFKPTYAYGALGCPPLIPPNATVLFEIELLDF
XP_004840130	DITLWGMELGLLSMRKGELARFLFKPTYAYGALGCPPLIPPNATVLFEIELLDF

XP_014403986	DITLWGMELGLLTMRRGELARFLFKPPYAYGTLGCPPLIPPNSTVLFEIELIDF
XP_016800790	DITLWGMELGLLSMRRGELARFLFKPNYAYGTLGCPPLIPPNTTVLFEIELLDF
XP_014988804	DITLWGMELGLLSMRRGELARFLFKPNYAYGTLGCPPLIPPNTTVLFEIELLDF
ELK06863	DITLWGMELGLLSMRRGELARFLFKPTYAYGTLGCPPLIPPNTTVLFEIELLDF
EHB08283	DITLWGMELGLLSMRKGELARFLFKPTYAYGALGCPPLIPPNATVLFEIELLDF
XP_004706046	DITLWGMELGLLTMRRGELARFLFKPTYAYGVLGCPPLIPPNTTVLFEIELLDF
XP_021110082	DITLWGMELGLLSMRKGELARFLFKPTYAYGALGCPPLIPPNATVLFEIELLDF
XP_007518064	DITLWGMELGLLSMRRGELARFLLSPRYAYGALGCPPLIPPEATVLFEIELLDF
PNI92595	DITLWGMELGLLSMRRGELARFLFKPNYAYGTLGCPPLIPPNTTVLFEIELLDF
XP_020861399	EITLWGMEVGLLTMRKGELARFLFKPRYAYGALGCPPLIPPNTTVLFELELLDF
XP_016284889	EITLWGMEVGLLTMRKGELARFLFKPRYAYGALGCPPLIPPNTTVLFELELLDF
XP_016284888	EITLWGMEVGLLTMRKGELARFLFKPRYAYGALGCPPLIPPNTTVLFELELLDF
XP_001519210	EITLGGMEVALLTMRKGELARFLFKPAYAYGRLGCPPLIPADATVLFEMELLDF
XP_020861404	EITLWGMEVGLLTMRKGELARFLFKPRYAYGALGCPPLIPPNTTVLFELELLDF
XP_014425414	DITLLGMEVGLLTMKKGELARFLFMPSYAYGALGCPPLIPPNATVLFEIELLDF
XP_004761713	DITLWGMELGLLSMRRGELARFLFKPTYAYGTMGCPPLIPPNTTVLFEIELLDF
XP_007441600	DITLWGMEVGLLTMRKGELARFLFKPNYAFGTLGCPPLIPPNSTVLFEVELLDF
XP_016080873	EITLGGMEVALLTMRKGELARFLFKPAYAYGRLGCPPLIPADATVLFEMELLDF
XP_024066689	EITLQGMEVGLLTMKKGESSRFLFTPSYAYGALGCPPLIPPNATVLFEMELLDF
XP_019365238	EITLWGMEIGLLTMKKGELARFLFMPRYAYGTLGCLPLIPPNATVLFEIELMDF
XP_019401968	EITLWGMEIGLLTMKKGELARFLFMPHYAYGTLGCPPLIPPNATVLFEIELMDF
XP_013926437	DITLRGLEVGLLTMRKGELARFLFKPNYAFGTLGCPPLIPPNSTVLFEVELLDF
XP_019401969	EITLWGMEIGLLTMKKGELARFLFMPHYAYGTLGCPPLIPPNATVLFEIELMDF
XP_015269029	EITLRGMIVGLLTMKRGELARFLFRSPYAFGMTGCPPLIPPNATVLFEIELLDF
XP_014464913	EITLWGMEIGLLTMKKGELARFLFMPHYAYGALGCLPLIPPNATVLFEIALLDF
XP_006258869	EITLWGMEIGLLTMKKGELARFLFMPHYAYGALGCLPLIPPNATVLFEIALLDF
XP_005344684	DITLWGMELGLLSMRRGELARFLFKPTYAYGTLGCPPLIPPNATVLFEIELIDF
XP_015670323	DITLRGMEVGLLTMRKGELARFLFQPNYAFGTLGCPPLIPPNATVLFEVELLDF
XP_020662988	DITLRGMEAGLLTMKKGELARYLFKPSYAFGALGCPPLIPPNATVMFEIELLDF
XP_007065519	EITLQGMEVGLLTMKKGELARFLFTPSYAYGALGCPPLIPPNATVLFEMELLDF
XP_020662989	DITLRGMEAGLLTMKKGELARYLFKPSYAFGALGCPPLIPPNATVMFEIELLDF
XP_020662990	DITLRGMEAGLLTMKKGELARYLFKPSYAFGALGCPPLIPPNATVMFEIELLDF
XP_025051231	GITLWGMEIGLLTMKKGELARFLFMPHYAYGALGCLPLIPPNATVLFEIELLDF
KYO33272	EITLWGMEIGLLTMKKGELARFLFMPHYAYGALGCLPLIPPNATVLFEIALLDF
XP_021018814	DITLWGMELGLLSMRRGELARFLFKPAYAYGTLGCPPLIPPNATVLFEIELIDF
OBS57796	DITLWGMELGLLSMRRGELARFLFKPTYAYGTLGCPPLIPPNATVLFEIELIDF
NP_001264822	DITLWGMELGLLSMRKGELARFLFKPAYAYGTLGCPPLIPPNATVLFEIELIDF
XP_021042683	DITLWGMELGLLSMRRGELARFLFKPAYAYGTLGCPPLIPPNATVLFEIELIDF
XP_021519218	DITLWGMELGLLSMRRGELARFLFKPTYAYGTLGCPPLIPPNATVLFEIELIDF
XP_024653358	DITLWGMELGLLSMRRGELARFLFKPNYAYGTLGCPPLIPPNTTVLFEIELLDF
XP_009678488	DITLGGLEIGLLTMKKGEMARFVFMPNYAYGKLGCPPLIPLNATVLFEVEVLDF
XP_015302701	DITLWGMELGLLSMRRGELARFLFKPNYAYGTLGCPPLIPPNTTVLFEIELLDF
XP_003770271	EITLWGMEVGLLTMRKGELARFLFKPRYAYGALGCPPLIPPNTTVLFELELLDF
ETE73718	DITLRGLEIGLLTMRKGELARFLFKPNYAFGTLGCPPLIPPNSTVLFEVELLDF
NP_989052	DITLSGMEIGLLTMQRGELSRFLFSPKYAYGTLGCSPLIPPSATVLFEIELLDF
NP_001085204	DITLAGMEIGLLTMQRGELSRFLFSPKYAYGTLGCPSLIPPSATALFEIELLDF
XP_011592867	DITLGGLEIGLLTMKKGEVARFVFTPDYAYGRQGCPPLIPPNATVMFEVEVLDF
XP_010584273	DITLWGLEIGLLTMKKGEVARFVFTPDYAYGRQGCPPLIPPNATVMFEVEVLDF
XP_017946629	DITLSGMEIGLLTMQRGELSRFLFSPKYAYGTLGCSPLIPPSATVLFEIELLDF
XP_008109385	DITLRGMELGLLTMKKQEVARYLFKPNYAFGRMGCPPLIPPDATVMFEIELLDF
XP_021271278	DITLEGLEIGLLTMRKGEVARFIFTPNYAYGQQGCLPLIPPNAMVLFEVELIDF
XP_023074397	DITLWGMELGLLSMRRGELARFLFKPNYAYGTLGCPPLIPPNTTVLFEIELLDF
XP_015151391	DITLGGLEIGVLTMKKGEVARFIFSPSYAYGQQGCLPLIPPDATVLFEVELIDF
XP_018100492	DITLAGMEIGLLTMQRGELSRFLFSPKYAYGTLGCPSLIPPSATALFEIELLDF
POI24993	DITLGGLEIGVLTMKKGEVARFIFSPSYAYGQQGCLPLIPPDATVLFEVELIDF
PNI92596	DITLWGMELGLLSMRRGELARFLFKPNYAYGTLGCPPLIPPNTTVLFEIELLDF
XP_015736491	DITLQGLEIGVLTMKKGEVARFIFAPSYAYGQQGCPPLIPPNATVLFEVELIDF
XP_010283169	DITLAGLEIGLLTMKKGEVSRFVFTPDYAYGRQGCPPLIPPNAMVMFEVEVLDF

PNJ85879	DITLWGMELGLLSMRRGELARFLFKPNYAYGTLGCPPLIPPNTTVLFEIELLDF
XP_018412624	DITLIGMEVSVLTMQRGELSRFLYSPAYAYGALGCPPLIPASATVLFEIEMLDF
XP_010211497	DITLWGLEVGLLTMRKGELARFVFAPDYAYGKLGCPPLIPPNATVLFEVELLDF
XP_017388864	DITLWGMELGLLSMRRGELARFLFKPTYAYGTLGCPPLIPPDTTVLFEIELIDF
XP_008109386	DITLRGMELGLLTMKKQEVARYLFKPNYAFGRMGCPPLIPPDATVMFEIELLDF
XP_014342809	DITLLGMEIGLHTMKKGEFSRFLFKPEYAYGEMGCPPLIPPHSTVLFEVELQDF
KFV84016	DITLGGLEIGLLTMKKGEMARFVFMPNYAYGKLGCPPLIPLNATVLFEVEVLDF
XP_013812139	DITLGGLEIGLLTMKKGELARFVFTPDYAYGKLGCPPLIPLNAVVLFEVELLDF
XP_009643249	DITLGGLEIGLLTMKKGEVARFVFTPEYAYGRQGCPPLVPPNATVMFEVEVLDF
XP_009928379	DITLWGLEIGLLTMKKGEVARFVFTPDYAYGRQGCPPLIPPNATVMFEVEVLDF
XP_010124464	DITLRGLEIGILTMKKGEVARFVFMPNYAYGQMGCPPLIPPNATVMFEVEVLDF
XP_009321903	DITLGGLEIGLLTMKKGEVARFVFTPDYAYGRQGCPPLIPPNATVVFEVEVLDF
XP_009807780	DITLGGLEIGLLTMKKGEVARFVFMPDYAYGRQGCPPLIPPNATVMFEVEVLDF
XP_009512874	DITLGGLEIGLLTMKKGEAARFVFTPDYAYGRQGCPPLIPPNATVMFEVELLDF
XP_009586283	DITLGGLEIGLLTMKKGEVARFVFTPDYAYGRQGCPPLIPPNAMVLFEVEVLDF
KFQ72829	DITLAGLEIGLLTMKKGEVSRFVFTPDYAYGRQGCPPLIPPNAMVMFEVEVLDF
XP_009698973	DITLGGLEIGLLTMKKGEAARFVFMPDYAYGRQGCPPLIPPNAMVMFEVEVLDF
XP_009901738	DITLWGLEIGLLTMRKGEAARFIFKPCYAYGQQGCHPVIPPNATVTFEVELRDF
XP_012887159	DITLWGMELGLLSMRRGELARFLFKPTYAYGTLGCPPLIPPNSTVLFEIELLDF
XP_009940812	DITLGGLEVGLLTMKKGEVARFVFTPAYAYGQQGCPPLIPPNATVMFEVELLDF
XP_002187983	DITLWGLEIGLLSMTKGEAAQFILAPKYAYGQLGCPPLIPPNTTVLFKVEVLDF
XP_009887190	DITLVGLEIGLLTMKKGEVARFVFRPDYAYGQQGCPPLIPPNATVMFEVEVLDF
XP_009486380	DITLGGLEIGLLTMKKGEVARFVFTPDYAYGRQGCPPLIPPNATVMFEVEVLDF
KGL76252	DITLWGLEVGLLTMRKGELARFVFAPDYAYGKLGCPPLIPPNATVLFEVELLDF
XP_010306733	DITLRGLVIGLLTMKKGEGAKFVFTPDYAYGRQGCPPLIPPNATVMFEVEVLDF
XP_008979919	DITLWGMELGLLSMRRGELARFLFKPTYAYGTLGCPPLIPPDTTVLFEIELLDF
XP_009998510	DITLWGLEIGLLTMRKGEVARFFLMPKYAYGQLGCPPLIPPNSTVTFEVELLDF
XP_009463122	DITLGGLEIGLLTMKKGEVARFVFTPYYAYGRQGCPPLIPPNATVMFEVEVLDF
XP_007894626	DITLVGMELGVRTMREGEFARFLLKPEYAYGRLGCPPRIPPNATVLLELEMLGF
XP_010022120	DITLLGLEVGLLTMKKGELSRFVFTPGYAYGQRGCPPLIPPSATVIFEVEVLDF
XP_010083598	DITLWGLEIGLLTMKVGEAARFFFLPGYAYGKMGCPPLIPPNAMATFEVEVVDF
XP_010194269	EITLLGLKVGLLTMKKEEMAIFIFLPEYAYGSMGCPPLIPPNATVMFEVELLDF
XP_009962683	DITLQGLAVGLLTMKKGEAARFVFMPDYAYGQQGCPPLIPPSATVMFEVELLDF
KFP29338	EITLLGLKVGLLTMKKEEMAIFIFLPEYAYGSMGCPPLIPPNATVMFEVELLDF
XP_010147897	DITLWGLEIGLLTMKKGEVARFVFMPGYAYGRQGCLPLIPPNATVLFEVEVLDF
KFQ39479	DITLGGLEIGLLTMKKGEVARFVFTPPYAYGQQGCPPLIPPNATVMFEVEVLDF
EOB04024	DITLRGLEIGLLTMKKGEVARFVFTPVYAYGQQGCPPLIPPNATVLFEVEMLDF
XP_023675251	DVTLPGLELGLLTMKKGEFSRFLFLAKYAYGEMGCPPLIPPSATVLYEVQVLDF
XP_009557002	DITLQGLEIGLLTMKKGEVAKFVFAPDYAYGQRGCPPLIPPSATVMFEVELLDF
XP_008944700	DITLLGLEISLLTMRKGEAARFVFSPKYAYGPQGCPPLIPPEATVLFEVELLDF
KFP93140	DITLWGLEIGLLTMKKGEVARFVFTPDYAYGRQGCPPLIPPNATVMFEVEVLDF
KFP44723	DITLRGLEIGILTMKKGEVARFVFMPNYAYGQMGCPPLIPPNATVMFEVEVLDF
XP_017553040	DVTLCGLELGLLSMRKGEFSRFLFQPDYAFGEMGCPPLIPPAATVLYEVQVLEF
XP_021396070	DITLWGLEIGLLSMTKGEAALFVLAPEYAYGQRGCPPFIPSNTMVLFKVEVLDF
XP_010143455	DITLRGLEIGLLTMTKGEAARFVFTPDYAYGQRGCYPLIPCNATVVFEVEVLDF
KFQ87275	DITLGGLEIGLMTMKKGEVARFVFTPDYAYGRQGCPPLIPPNATVMFEVEVLDF
XP_010891852	DVTLYGLELGILTMKKGEFSRFLFLPEYAYGDMGCPPLIPPFATVLYEVQVLDF
XP_009863922	DITLGGVEVGLLTMKKGEVARFYFSPEYAYGHQGCPPLIPPNTSVVFDVEVLDF
KFW66808	DITLGGLEIGLLTMKKGEVARFVFTPDYAYGRQGCPPLIPPNATVVFEVEVLDF
XP_018591809	DVTLPGLEVGLLTMKSGEFSRFLFLPKYAYGDMGCPPLIPPSATLLYEVHLLDF
KFP21064	DITLGGLEIGLLTMKKGEVARFVFTPEYAYGRQGCPPLVPPNATVMFEVEVLDF
KFZ64979	DITLAGLEIGLLTMKKGEVARFIFMPDYAYGKQGCPPLIPPNATVMFEVEVLDF
KFW73903	DITLGGLEIGLLTMKKGEAARFVFTPDYAYGRQGCPPLIPPNATVMFEVELLDF
KFM05579	DITLGGLEIGLLTMKKGEVARFVFTPDYAYGRQGCPPLIPPNATVMFEVEVLDF
KFV69563	DITLWGLEIGLLTMRKGEAARFIFKPCYAYGQQGCHPVIPPNATVTFEVELRDF
XP_023823731	DVTLCGLELGILTMRKGEFSRFLFLPAYAYGEMGCPPLIPPVATVLYEVQVLDF
XP_016346087	DVTLYGLELGLLTMKKGEFSRFLFKPKYAYGDLGCPPHIPPLATVLYEVQVLDF
KFV51798	DITLGGLEIGLLTMKKGEVARFVFMPDYAYGRQGCPPLIPPNATVMFEVEVLDF

KFW03341	DITLWGLEIGLLTMKKGEVARFVFMPGYAYGRQGCLPLIPPNATVLFEVEVLDF
KFV99355	DITLGGLEIGLLTMKKGEVARFVFTPDYAYGRQGCPPLIPPNAMVLFEVEVLDF
OXB56102	DITLLGLEIGLLTMKKGELARFIFTPNYAYGKQGCLPLIPKNATVLFIVELIDF
XP_018958387	DVTLYGLELGLLTMKKGEFSRFLFKPKYAYGDLGCPPHIPPLATVLYEVQVLDF
XP_019903677	DVTLYGLELGILTMKKGEFSRFLFLPEYAYGDMGCPPLIPPFATVLYEVQVLDF
KFU91562	DITLWGLEIGLLTMRKGEVARFFLMPKYAYGQLGCPPLIPPNSTVTFEVELLDF
XP_017314960	DVTLWGLDISLRTMRRGEFSRFLLQPRYAYGELGCPPIIPPGATVLYEVQVFDY
XP_016393789	DVTLYGLELGLLTMKKGEFSRFLFKPKYAYGDLGCPPHIPPLATVLYEVQVLDF
XP_009093115	DITLWGLEIGLLSMTKGEAALFVLTPEYAYGQRGCPPSIPPNTTVLFKVEVLDF
XP_023869471	DVTLCGLELGILTMRKGEFSRFLFLPEYAYGAMGCPPLIPPVATVLYEVQVLDF
XP_019903679	DVTLYGLELGILTMKKGEFSRFLFLPEYAYGDMGCPPLIPPFATVLYEVQVLDF
XP_019903678	DVTLYGLELGILTMKKGEFSRFLFLPEYAYGDMGCPPLIPPFATVLYEVQVLDF
XP_014110391	DITLCGLEIGLLSMTKGEAALFILTPEYAYGQRGCPPSIPPNTTVLFKVEVLDF
XP_017314964	DVTLWGLDISLRTMRRGEFSRFLLQPRYAYGELGCPPIIPPGATVLYEVQVFDY
XP_019903676	DVTLYGLELGILTMKKGEFSRFLFLPEYAYGDMGCPPLIPPFATVLYEVQVLDF
XP_015502317	DITLCGLEIGLLSMTKGEAALFILTPEYAYGQRGCPPSIPPNTTVLFKVEVLDF
XP_020341240	DVTLCGLELGILTMRKGEFSRFLFLPEYAYGAMGCPPLIPPVATVLYEVQVLDF
NP_001122145	DVTLYGLELGLLTMKKGEFSRFLFKPKYAYGDLGCPPHIPPCATVLYEVQVLDF
OWK52486	DITLWGLEIGLLSMTKGEAALFVLAPEYAYGQRGCPPFIPSNTMVLFKVEVLDF
XP_013992803	DVTLCGLELGILTMRKGEYSRFLFLPEYAYGAMGCPPLIPPVATVLYEVQVLDF
XP_024287707	DVTLCGLELGILTMRKGEFSRFLFLPEYAYGAMGCPPLIPPVATVLYEVQVLDF
AAH54673	DVTLYGLELGLLTMKKGEFSRFLFKPKYAYGDLGCPPHIPPCATVLYEVQVLDF
KFP60963	DITLGGLEIGLLTMKKGEAARFVFMPDYAYGRQGCPPLIPPNAMVMFEVEVLDF
XP_018958370	DVTLYGLELGLLTMKKGEFSRFLFKPKYAYGDLGCPPHIPPLATVLYEVQVLDF
KGL96380	DITLVGLEIGLLTMKKGEVARFVFRPDYAYGQQGCPPLIPPNATVMFEVEVLDF
XP_016148031	DVTLYGLELGLLTMKKGEFSRFLFKPKYAYGDLGCPPHIPPLATVLYEVQVLDF
XP_007245276	DMTLCGLELGLLTMRKGECSRFLFKHDYAFGEMGCPPLIPPLAMVLYEVQVLDF
XP_023794776	DITLCGLEIGLLSMTKGEAALFILTPKYAYGQRGCPPSIPPNTTVLFKVEVLDF
XP_021479427	DVTLCGLELGILTMRKGEFSRFLFLPEYAYGAMGCPPLIPPVATVLYEVQVLDF
KFR16943	DITLGGLEVGLLTMKKGEVARFVFTPAYAYGQQGCPPLIPPNATVMFEVELLDF
XP_009988051	DITLRGLELGLKTMKKGEAARFVFVPDYAYGRLGCPPLIPPNATVTFEVEVLDF
KFV16869	DITLWGLEIGLLTMKVGEAARFFFLPGYAYGKMGCPPLIPPNAMATFEVEVVDF
KFO13846	DITLRGLVIGLLTMKKGEGAKFVFTPDYAYGRQGCPPLIPPNATVMFEVEVLDF
XP_020373313	DITLLGMELAIQTMRKGEFARFLFKPQYAYGQMGCPPRIPANATVMFELEILHF
XP_006640997	EVTLVGLEVGLLTMRKGEFARFLFLPDYAYGAMGCPPLIPPAATVLFEVHLLDF
XP_020341239	DVTLCGLELGILTMRKGEFSRFLFLPEYAYGAMGCPPLIPPVATVLYEVQVLDF
KQK73326	DITLLGLEVGLLTMKKGELSRFVFAPGYAYGQLGCPPLIPPSATVIFEVEVLDF
XP_008500186	DITLWGLEIGLLTMRKGERARFFFTPSYAYGAMGCPPLLPPNAMVMFEVELLDF
XP_013992801	DVTLCGLELGILTMRKGEYSRFLFLPEYAYGAMGCPPLIPPVATVLYEVQVLDF
XP_016350445	DVTLYGLELGLLTMKKGEFSRFLFMPKYAYGDLGCPPHIPPLAMVLYEVQVLDF
KFV47875	DITLQGLAVGLLTMKKGEAARFVFMPDYAYGQQGCPPLIPPSATVMFEVELLDF
XP_022534491	DMTLCGLELGLLTMRKGECSRFLFKHDYAFGEMGCPPLIPPLAMVLYEVQVLDF
XP_021479426	DVTLCGLELGILTMRKGEFSRFLFLPEYAYGAMGCPPLIPPVATVLYEVQVLDF
KFQ96759	DITLGGLEIGLLTMKKGEVARFVFTPYYAYGRQGCPPLIPPNATVMFEVEVLDF
XP_012685286	DVTLWGLEVGLLTMKKGELAHFLFKPKYAYGEMGCPPLIPSSATVFYEVHVIDF
KFQ41466	DITLLGLEVGLLTMKKGELSRFVFTPGYAYGQRGCPPLIPPSATVIFEVEVLDF
XP_024997748	DITLGGLEIGVLTMKKGEVARFIFSPSYAYGQQGCLPLIPPDATVLFEVELIDF
KFQ35533	DITLLGLEISLLTMRKGEAARFVFSPKYAYGPQGCPPLIPPEATVLFEVELLDF
XP_020459257	DMTLVGLEQGLLTMQKGEFSRFLFQPLYAYGDIGCPPLIPAAAVVLYEVEILDF
XP_017264554	DVTLAGLELGLLTMKRGEFSRFLFQPQYAYGDMGCPPFIPAAAAVLYEVQVLDY
XP_005917667	DVTLAGLELGLLTMKKGEFSRFLLQPQYAYGEMGCPPFIPAAAVVLYEVQILDY
XP_022621130	DVTLAGLELGLLTMQKGEFSRFLFQPHYAYGDMGCPPFIPAAAVVLYEVQILDF
XP_005725080	DVTLAGLELGLLTMKKGEFSRFLLQPQYAYGEMGCPPFIPAAAVVLYEVQILDY
XP_010408101	DITLWGLEIGLLSMTKGETALFVLRPEYAYGQRGCPPSIPPNTTVLFKVELLDF
KFO74526	DITLQGLEIGLLTMKKGEVAKFVFAPDYAYGQRGCPPLIPPSATVMFEVELLDF
XP_005449739	DVTLAGLEVGLLTMKKGEFSRFLLQPQYAYGEMGCPPFIPAAAVVLYEVQILDY
XP_023285908	DVTLVGLELGLLTMQKGEFSRFLFQPHYAYGDMGCPPFIPAAAVVLYEVQILDF
XP_015502316	DITLCGLEIGLLSMTKGEAALFILTPEYAYGQRGCPPSIPPNTTVLFKVEVLDF

XP_004542108	DVTLAGLELGLLTMKKGEFSRFLLQPQYAYGEMGCPPFIPTAAVVLYEVQILDY
XP_023823730	DVTLCGLELGILTMRKGEFSRFLFLPAYAYGEMGCPPLIPPVATVLYEVQVLDF
XP_020459258	DMTLVGLEQGLLTMQKGEFSRFLFQPLYAYGDIGCPPLIPAAAVVLYEVEILDF
XP_008634949	DITLWGLEIGLLSMTKGETALFVLRPEYAYGQRGCPPSIPPNTTVLFKVELLDF
XP_013883182	DVTVAGLELGLLTMKKGEFSRFLFQPQYAYGDMGCPPFIPGAATVLYEVHVLDY
XP_019947232	DVTLAGLQLGLLTMKKGEFSRFLFQPQYAYGDMGCPPIIPAAAKILYEVQILDF
KFP85199	DITLGGVEVGLLTMKKGEVARFYFSPEYAYGHQGCPPLIPPNTSVVFDVEVLDF
PWA27518	DVTLAGLELGLLTMKKGEFSRFLLQPQYAYGEMGCPPFIPAAAAILYEVHILDY
XP_003971664	DVTLTGLEMGLLTMKKGEFSRFLFEPQYAYGDLGCPPLIPAFSVVLYEVHILDF
XP_005917666	DVTLAGLELGLLTMKKGEFSRFLLQPQYAYGEMGCPPFIPAAAVVLYEVQILDY
XP_013764145	DVTLAGLELGLLTMKKGEFSRFLLQPQYAYGEMGCPPFIPAAAVVLYEVQILDY
XP_013764144	DVTLAGLELGLLTMKKGEFSRFLLQPQYAYGEMGCPPFIPAAAVVLYEVQILDY
CAG01803	NVTLAGLEMGLLTMKKGEFSRFLFEPQYAYGDLGCPPLIPAFAEILYEVHILDF
XP_005449738	DVTLAGLEVGLLTMKKGEFSRFLLQPQYAYGEMGCPPFIPAAAVVLYEVQILDY
KFV20040	DITLRGLELGLKTMKKGEAARFVFVPDYAYGRLGCPPLIPPNATVTFEVEVLDF
XP_018551539	DVTLVGLELGLLTMQKGEFSRFLFQPQYAYGDMGCPPFIPSSAVVLYEVQILDF
XP_004542106	DVTLAGLELGLLTMKKGEFSRFLLQPQYAYGEMGCPPFIPTAAVVLYEVQILDY
XP_005808385	DVTLAGLELGLLTMKKGEFSRFLLQPQYAYGEMGCPPFIPAAAAILYEVHVLDY
KFO85715	DITLRGLEIGLLTMTKGEAARFVFTPDYAYGQRGCYPLIPCNATVVFEVEVLDF
XP_015236136	DVTLAGLELGLLTMKKGEFSRFLFDPQYAYGEMGCPPLIPAAATVLYEVQIVDY
XP_023794777	DITLCGLEIGLLSMTKGEAALFILTPKYAYGQRGCPPSIPPNTTVLFKVEVLDF
XP_015803473	EVTLAGLEMGLLTMKRGEFSRFLFQPRYAYGDMGCPPLIPASATILYEVQLLDY
KPP68264	DVTLPGLEVGLLTMKSGEFSRFLFLPKYAYGDMGCPPLIPPSATLLYEVHLLDF
XP_020488061	DVTLAGLELGLLTMKKGEFSRFLFQPQYAYGDMGCPPFIPTAATVLYEVHILDF
XP_015332442	DITLWGMELGLLSMRRGELARFLFKPTYAYGALGCPPLIPPNTTVLFEIELLDF
XP_013764143	DVTLAGLELGLLTMKKGEFSRFLLQPQYAYGEMGCPPFIPAAAVVLYEVQILDY
XP_005449737	DVTLAGLEVGLLTMKKGEFSRFLLQPQYAYGEMGCPPFIPAAAVVLYEVQILDY
KFO61461	DITLWGLEIGLLSMTKGETALFVLRPEYAYGQRGCPPSIPPNTTVLFKVELLDF
XP_008298046	DVTLAGLELGLLTMKKGEFSRFLLQPRYAYGDMGCPPLIPAAAAVLYEVQILDY
KFP07119	DITLWGLEIGLLTMRKGERARFFFTPSYAYGAMGCPPLLPPNAMVMFEVELLDF
XP_007561093	DVTLAGLELGLLTMKKGEFSRFLLQPQYAYGEMGCPPFIPAAAAILYEVHVLDY
PKU35662	DITLLGLEIGLLTMKKGEVARFVFMPDYAYGRQGCPPLIPPNATVMFEVEVLDF
XP_010793476	DLTLAGLELGLMTMRKGEFSRFLLQPKYAYGEMGCPPLVPAAAVILYEVHILEF
AWP19851	DMTLAGLELGVLTMQNGEFSRFLFQPQYAYGDLGCPPFIPGAAAVLYEVQILDI
KFW82601	DITLWGLELGLMSMARGEVARFWFSPRYAYGPRGCPPHIPPDTTVLFHVELLDF
XP_008396659	DVTLAGLELGLLTMKKGEFSRFLLQPQYAYGEMGCPPFIPAAAAILYEVQVLDY
XP_010739761	EVTLAGLDLCLLTMKKGEFSRFLLEPQYAYGDLGCPPLIPAAAVVLYEIQIIDY
XP_022046121	DVTLAGLELGLLTMKKGEFSRFLLQPQYAYGDMGCPSLIPRAAVVLYEVQILDY
XP_019715044	DMTLMGMEMGLLTMRKGESSRFLLQPRYAYGDMGCPPLIPAAARILFEIQIHDV
XP_018875413	DITLWGMELGLLSMRRGELARFLFKPNYAYGTLGSPPLIPPNTTVLFEIELLDF
XP_014327714	DVTLAGLELGLLTMKKGEFSRFLLQPQYAYGEMGCPPFIPAAAAILYEVHVLDY
XP_009081377	DITLWGLELGLLSMAKGELAWFIFVPKYAYGQMGCPPCIPPNTTVLFLVLLLDF
AWP19849	DMTLAGLELGVLTMQNGEFSRFLFQPQYAYGDLGCPPFIPGAAAVLYEVQILDI
XP_023134576	DMTLAGLELGLLTMKKGEFCRFLLQPQYAYGDMGCPPLIPAAAVVLYEVHILDY
XP_012734526	DVTVAGLELGLLTMKKGEFARFLLHHQYAYGEMGCPPFIPPSSTVLYEVHILDY
XP_014403985	DITLWGMELGLLTMRRGELARFLFKPPYAYGTLGCPPLIPPNSTVLFEIELIDF
XP_017553083	DVTLCGLELGLLSMRKGEFSRFLFQPDYAFGEMGCPPLIPPAATVLYEVQVLEF
XP_023869472	DVTLCGLELGILTMRKGEFSRFLFLPEYAYGAMGCPPLIPPVATVLYEVQVLDF
XP_013992804	DVTLCGLELGILTMRKGEYSRFLFLPEYAYGAMGCPPLIPPVATVLYEVQVLDF
XP_020774925	DVTLGGLELGLLTMKKGEFSRFLLQPHYAYGDMGCPPFIPAHAVILYEVHVLDF
XP_024287709	DVTLCGLELGILTMRKGEFSRFLFLPEYAYGAMGCPPLIPPVATVLYEVQVLDF
XP_023869475	DVTLCGLELGILTMRKGEFSRFLFLPEYAYGAMGCPPLIPPVATVLYEVQVLDF
XP_013992805	DVTLCGLELGILTMRKGEYSRFLFLPEYAYGAMGCPPLIPPVATVLYEVQVLDF
XP_023869476	DVTLCGLELGILTMRKGEFSRFLFLPEYAYGAMGCPPLIPPVATVLYEVQVLDF
XP_024287708	DVTLCGLELGILTMRKGEFSRFLFLPEYAYGAMGCPPLIPPVATVLYEVQVLDF
XP_023869474	DVTLCGLELGILTMRKGEFSRFLFLPEYAYGAMGCPPLIPPVATVLYEVQVLDF
XP_011490382	DLTLWGLELGLLTMKRGEHSRVLLQPQYAYGDLGCPPLIPAAAVVLYEVQIVDY
XP_020570387	DLTLWGLELGLLTMKRGEHSRVLLQPQYAYGDLGCPPLIPAAAVVLYEVQIVDY

XP_008396657	DVTLAGLELGLLTMKKGEFSRFLLQPQYAYGEMGCPPFIPAAAAILYEVQVLDY
XP_014851597	DVTLAGLELGLLTMKKGEFSRFLLQPQYAYGEMGCPPFIPAAAAILYEVHVLDY
XP_020570386	DLTLWGLELGLLTMKRGEHSRVLLQPQYAYGDLGCPPLIPAAAVVLYEVQIVDY
XP_008396658	DVTLAGLELGLLTMKKGEFSRFLLQPQYAYGEMGCPPFIPAAAAILYEVQVLDY
XP_018774793	DITLWGLEIGLLSMTKGEAALFVLTPEYAYGQRGCPPSIPPNTTVLFKVEVLDF
XP_023794778	DITLCGLEIGLLSMTKGEAALFILTPKYAYGQRGCPPSIPPNTTVLFKVEVLDF
XP_024147377	ELTLWGLELGLLTMKRGELSRFLFQPEYAYGDLGCPPLIPAAARVLYEVQILDY
XP_024147375	ELTLWGLELGLLTMKRGELSRFLFQPEYAYGDLGCPPLIPAAARVLYEVQILDY
XP_024147378	ELTLWGLELGLLTMKRGELSRFLFQPEYAYGDLGCPPLIPAAARVLYEVQILDY
XP_014851599	DVTLAGLELGLLTMKKGEFSRFLLQPQYAYGEMGCPPFIPAAAAILYE------
XP_008396660	DVTLAGLELGLLTMKKGEFSRFLLQPQYAYGEMGCPPFIPAAAAILYE------
XP_017593619	DITLWGLEIGLLSMTKGETALFVLRPEYAYGQRGCPPSIPPNTTVLFKVELLDF
XP_008396661	DVTLAGLELGLLTMKKGEFSRFLLQPQYAYGEMGCPPFIPAAAAILYEVQVLDY
XP_014851600	DVTLAGLELGLLTMKKGEFSRFLLQPQYAYGEMGCPPFIPAAAAILYEVHVLDY
XP_019619874	GSVVPGMEIAISSMKKGERSRFLVQPNYGYGKFGCPPRIPGNATVLYEIEMLNF
XP_016801711	DITLWGMELGLLSMRRGQLARFLFKPNYAYGTPGSPPLIPPNTTVLFEIELLDF
XP_023710706	G-FITGLDIAIASMKKGETSRFLIHPDKAYGKLGCPPRIPQNAELLFEVELVKF
XP_020627099	EELLPGWYIGVSTMRRGEKSKFLLSPEYAFKDLGSPPRVPPSATVLFEVELLSY
XP_006793207	DVTLAGLELGLLTMKKGEFSRFLLQPQYAYGEMGCPPFIPAAAVVLYEVQILDY
XP_011402831	HQVIEGLEVGVASMRKEEVSQFLVKSPYAFGDMGCPPRIPPAASILFEVEVLSF
XP_022801008	EENILGLYYGLRTMRKGEKSKFLVTPEYAYLEMGCTPRIPASAKVLFEVELLSF
XP_013786360	ERVILGLDVGVGTMKKGEVAQFLIRPEYAFGQMGCPPRIPPNATVMYEVELISY
XP_022254671	ERVILGLDVGVGTMKKGEVAQFLIRPEYAFGQMGCPPRIPPNATVMYEVELISY
ELT98785	EEVTPGFEIAVASMRKREEARFLVQPRYFLGTVGCPPRIPGNEPVLFWIQMVDF
XP_021353947	GQLVEGLELGLSTMKKGELSRFIITYDYAFGKLGCAPRIPPESTVMYEVELLSF
XP_022108900	GQVIPGLEIAVSTMRKGEKSDFLVSPEYAFGPRGVPPRIPENATILFVIELLAI
XP_022108899	GQVIPGLEIAVSTMRKGEKSDFLVSPEYAFGPRGVPPRIPENATILFVIELLAI
XP_002167164	GEGIEGWQIAISTMKRGEISKFLLHPTVAFGKMGCPPRIPSNAEVLFEIELISY
KOF62423	DMVIPGWVIAVTTMQVGELSRFLISPEYGFGDFGCPPRIPKAITALFEIELLSF
XP_014790271	DMVIPGWVIAVTTMQVGELSRFLISPEYGFGDFGCPPRIPKAITALFEIELLSF
XP_001636239	EGAFPGLDIGVSTMRKGELSRFLFDKEYVFKDLGCEPRVPG-ATVMWEVELLSF
XP_023237057	NRVVEGLDIAVATMKKGEISQFLIKYLYAYGEMGSPPRIPPSATILYEVELLSF
XP_002128893	GQVIHGLDIGISTMRKFEIARFLIESTYAYGNMGCPPRIPGHALVCFEVELLGY
XP_009051935	GAVIIGLEIAVSSMKKGEKSRYLFTPEYAYGRMGCGERIPGNSTVLAEVELLSF
XP_012221039	NCLIPGLELGILSMKKHEIAMFLIHPDLAYKALGCPPRIPPNEEVVFIVHLIDI
XP_012264923	GMLIPGLDIGIQTMKKYEQSVFVINSDYAYGAMGCPPRIPPNEDVLFIVILESF
XP_009857551	GQVIHGLDIGISTMRKFEIARFLIESTYAYGNMGCPPRIPGHALVCFEVELLGY
XP_015515854	GVLLPGLEIGIQTMKKHEISHFIIHPDYAFGPMGCPPRIPPNEEVMFAVYLSDF
XP_011415211	GGLIVGLDLAVSSMKKGELARYIIRPQYAFGEMGTPPRIPKDATVMYEVELLNF
XP_020909274	GGAFPGLDIALSTMRKGELAKFLFKKEYVFKDLGCEPRVPG-GTVLWEIELLGF
EKC39444	GGLIVGLDLAVSSMKKGELARYIIRPQYAFGEMGTPPRIPKDATVMYEVELLNF
XP_013405452	GDTIMGLEIAVSSMRKGELARFIFSYKYAYGERGCPPRIGEKADVMFEVELLNF
XP_011148430	NIIIPGLEIAICSMRKHEVAVFWLHPDYAYRAIGCLPRIPPNVEVAFIVHLTDF
XP_013405451	GDTIMGLEIAVSSMRKGELARFIFSYKYAYGERGCPPRIGEKADVMFEVELLNF
PVD31214	DSRVLGFHVAVSTMKKGEISRFLFKPTYFYGAMGCGQRIPPNCTALFDIELLSF
PIK39047	GEVIEGMELAISTMRQGELSKFLIAPEFAYGKYGCGKRIPPDSEILMEIELISF
XP_022336187	GGLIVGLDLAVSSMKKGELARVIIRPQYAFGEMGTPPRIPKDATVLYEVELLNF
XP_022337811	GGLIVGLDLAVSSMKKGELARVIIRPQYAFGEMGTPPRIPKDATVLYEVELLNF
XP_014787871	GKAIPGWEIALLTMKKGEISWFLIQPEYGFQETGCPPRVPRNATALFEIEILSI
EFN78601	NIIIPGLEIAICSMRKHEVAVFWLHPDYAYRAIGCLPRIPPNVEVAFIVHLTDF
XP_014475449	GMVIPGLELGIASMRKHEISVFWLHPDYAYKAIGCQPRIPPNAEIVFIVHLIDF
XP_014279621	GDLYLGFEKSLLTMREGEKSQFLVHSDYAFGKLGAPPRITPNATILLEIELIKI
XP_022193504	GEALIGVELALKTMKKGESSQFIVEPEYAFGKLGCPPRVPPESTVLFDIEVIDY
XP_014204130	GQVLQGLEIAICSMKKFEKALFIIQPSLAYGELGCLPRVPPNAEVLFKIDLIDF
KOX75315	GALIPGLEIGISTMQKHEIAIFIIHPDFAYGKYGCAPRIPPNEEILFIVHLVDY
XP_014234164	GAVIPGLEIGISSMKKFEKSLFIIQSELAYGELGCMPRIPPNAEVLFKVELVDF
KII63942	CDGLPGLQIGLSTMRKDEEAKFILRPDYAYGRFGCPPRIPPNSVVIFDVELISY
XP_024936423	GECIVGLEIGIRSMRKHEVAHFIIHPDLAFGKMGCLPRIPPNEDVLFAVHVVDF

XP_015586170	GECIVGLEIGIRSMRKHEVAHFIIHPDLAFGKMGCLPRIPPNEDVLFAVHVVDF
XP_003486736	DSLLPGLEIGITSMKKHEIAMFLIHPDLAYGKYGCAPRIPPNEEVLFIVHLTDY
XP_019882220	GYIIPGLEIGISTMQKHEIAIFLIHPDLAYKAFGCPPRIPPNEEVVFVVHLIDY
XP_003402471	DSLLPGLEIGIASMKKHEIAMFLIHPDLAYGKYGCAPRIPPNEEVLFIVHLTDY
XP_012134979	GMLIPGLEIAIASMRKHEIAIFIIHPDLAYGKYGCAPRIPPNEEILFVVHLVDY
XP_017756606	GMLIPGLEIAIASMQKHEIAIFIIHPDLAYGKYGCPPRVPPNEEILFIVHLVDY
XP_012134978	GMLIPGLEIAIASMRKHEIAIFIIHPDLAYGKYGCAPRIPPNEEILFVVHLVDY
XP_003700792	GMLIPGLEIAIASMRKHEIAIFIIHPDLAYGKYGCAPRIPPNEEILFVVHLVDY
XP_023290805	GQLLPGLEIAIASMKKHETSLFLIQPSLAYMELGCMPLVPPNSEVLFAIKLLDY
XP_012270499	GQLLPGLEIAIASMKKHETSLFLIQPSLAYMELGCMPLVPPNSEVLFAIKLLDY
XP_018309319	SFIIPGLEIGIRSMQKHEIAIFLIHPDYAYKAVGCPPRIPPNEEVIFVVQLVDY
XP_012062619	SFIIPGLEIGVRSMQKHEIAVFLIHPDYAYKAVGCPPRIPPNEEVVFVVHLVDY
XP_012527508	DCILPGLEIGIRSMQKHEIAVFLIHPDLAFKSLGCMPRIPPNEEVVFVVHLLHY
XP_005089179	GQCIRGLELALLSMKKHETARFLIHHDYAYGEMGCPPRIPKSTSLIYDVEILYF
XP_003424555	GGMIEGLEIAISTMRKYEKAIFIIQPHLAYGKLGCLPRIPPDSEVLFKIELIDF
XP_018055399	SFIIPGLEIGVRSMQKHEIAVFLIHPDYAYKAVGCPPRIPPNEEVVFVVHLVDY
XP_017877748	GTLIPGLDYAISSMKKHEVAVFIIHPDLGYGQYGCPPRIPPNEEILFVVQLDDY
XP_018399012	SFIISGLEIGIRSMQKHEIAVFLIHPDYAFKAIGCPPRIPPNEEVIFVVHLVDF
KYM99352	SFIISGLEIGIRSMQKHEIAVFLIHPDYAFKAIGCPPRIPPNEEVIFVVHLVDF
XP_018309322	SFIIPGLEIGIRSMQKHEIAIFLIHPDYAYKAVGCPPRIPPNEEVIFVVQLVDY
XP_011647883	SCIISGLEIGISTMQKHEIAVFLIHPDLAYKAFGCPPRIPPNEEVVFVVQLLDY
XP_014247948	QVVYEGFELSICTMKKGEKSQFLIDHTYAFGKMGAPPRIPPCATILVEIELINF
XP_011064230	NCIISGLEIGIRSMQKHEIAVFLIHPDYAYKAIGCPPRIPPNEEVVFVVTLVDY
XP_014613253	GLLIPGLELALTTMEKHEISIFIIHPDLAYGRLGCPPRILPNTEVMFIVHLVDF
XP_002430548	VETILGLNYAVKTMKKSENSRFFIKWEYAFGSLGCPDRIEPKCDIVADITLVDY
XP_014247946	QVVYEGFELSICTMKKGEKSQFLIDHTYAFGKMGAPPRIPPCATILVEIELINF
XP_014270488	GALYPGFEYSLSSMKLNEHSRFLVSPKYGFGDMGCPPRIPPNATLLLEIELLKI
ORY30517	GRVIKGWDEGLVTMRVGEKAELLIDPEYGYGVEGNPPTIPPNSTLLFEVEVISS
XP_011330216	ANIIPGLHMSICSMQKHEIAVFLIHPDLAYKTFGCPPRIPPNEEVVFIVHLIDY
EGI60945	SFIIPGLEIGIRSMQKHEIAVFLIHPDYAYKAVGCPPRIPPNEEVVFIVHLVDY
XP_001201219	GSVIPGMDLALSTMRTGEMSKFLIHPDHAYGKLGVPPRIP--------------
XP_011061927	SFIIPGLEIGIRSMQKHEIAVFLIHPDYAYKAVGCPPRIPPNEEVVFIVHLVDY
XP_018356457	SFIIPGLEIGIRTMQKHEIAVFLIHPDYAYKAVGCPPRIPPNEEVVFVVHLVDY
XP_011173578	NIVIPGLEIGIRSMQKHEIAIFVIHPDFAFGALGCLPRIPPNEEVAFVVHLLDF
XP_018356456	SFIIPGLEIGIRTMQKHEIAVFLIHPDYAYKAVGCPPRIPPNEEVVFVVHLVDY
XP_016917008	GMLIPGLEIGITTMQKHEIAMFIIHPDLAYGKYGCAPRIPPNEEILFIVHLTDY
XP_018374414	SFIIPGLEMGIRSMQKHEIAVFLIHPDYAYKAVGCPPRIPPNEEVVFVVHLVDY
XP_016917007	GMLIPGLEIGITTMQKHEIAMFIIHPDLAYGKYGCAPRIPPNEEILFIVHLTDY
XP_019882221	GYIIPGLEIGISTMQKHEIAIFLIHPDLAYKAFGCPPRIPPNEEVVFVVHLIDY
XP_011191641	E-VIEGLEVAVRTMQRGEQAQFVISHQLLFGEIGCPPRIKPSADGLFIIELVSF
PFH33404	GQVIRGWDVGVMKMKRGERAMLTIQSNYGYGSRGAPPTIPANSVLKFDVELIDS
XP_019876143	GEVLLGLDLAVQSMKAGEKSQFLFSPTYAYGKMGCLGRIPANTQVLFEIELKEI
XP_014299498	GELIHGLELSIQSMKKHEVSAFLIDPDLAYGEIGCWPNIPGNSEVLFIVNLIDY
KOC63519	GRLIPGLEIALTTMRRHEIAMFIVHPDLAYGKFGCAPRIPPNEQILFVVHLVDY
XP_015126020	GQLIPGFELAIQSMKKHEISIFWVHPDYAFGELGCAPLIPPSAEIMYVISLIDY
XP_005186588	E-VLQGLEDAVCTMHKGENAQFLISYNLLFREMGCPPRVKPKADGLFIIEVVSY
XP_011879049	NFILPGLEIAIRSMRKHEKSVFIIHPDLAYKAIGCPPRIPPNEEVAFIVHLIDY
XP_013109471	E-VLEGLEHAVCTMRKGENAQFVISYHLLFREMGCPPRVKPKADGLFIIELVGF
XP_011879048	NFILPGLEIAIRSMRKHEKSVFIIHPDLAYKAIGCPPRIPPNEEVAFIVHLIDY
XP_018333359	GEVILGLDMAVQSMKINEKSQFLVHPDYAYGRRGCLERIPENATILYEIELKNF
EFZ09302	NIVIPGLEIGIRSMQKHEIAIFVIHPDFAFGALGCLPRIPPNEEVAFVVHLLDF
XP_016927793	S-VVMGLEAAVLTMRPYEQAEFIISYKLLFGELGCPPRIKPMADALLKVEVIDY
XP_006611831	GMLIPGLEIGITTMQKHEIAMFIIHPDLAYGKYGCAPRIPPNEEILFIVHLTDY
XP_014245088	GVLYKGMEIAIATMSKGERSQFLVEPQLAFGKYGVPPRIPPNATILNVIDLIDF
XP_018790058	E-VIEGLEEAVRTMYRGEQAQFVISHHLLFREIGCPPRIKPSADGLFIIELVSF
XP_020893545	GGAFPGLDIDLSTMGKGELSKFLFKKEYVFKDLGCEPRVPG-GTVLWEIELLGF
XP_002092689	T-VVEGLEAAVRTMRPYEQAEFIISYKLLFGELGCPPRIKPKADALFKVEVIDY
XP_020293583	-EVLPGLALGLQTMLKDEIAAFLIHPDLAYKALGCAPTIPPNSVVLFVVHLLSF

```
XP_016952968      S-VIMGLEAAVLTMRPYEQAEFIISYKMLFGELGCPPRIKPMADALLKVEVIDY
XP_003689972      GMLIPGLEIGITTMQKHEIAMFIIHPDLAYGKYGCAPRIPPNEEILFIVHLTDY
XP_015185328      GYLIPGLELAITTMEKHEISIFIIHPDLAYGQLGCPPRILPNTEVMFIVHLVDF
XP_024867102      GYIIPGLEFGICSMKKHEIAVFLIHPDLAYKAIGCPPRIPPNEEVVFVVHLIDY
XP_011130643      GQVIKGWDVCVMSMKKGEKCRVELQPDFAYGEHGSPPTIPPNAVLVFEIELLEF
XP_011206843      E-VIEGLEEAVRTMYRGEQAQFVISHHLLFREIGCPPRIKPSADGLFIIELVSF
XP_008560701      GELIWGLDLSIQSMLKHEVSIFLIDPSLAYGEMGCWPTIPGNSEVMFIVHLLDY
XP_011695993      HFVIPGLEIGLCSMQKHEIAVFLMHPDYAYKAKGCPPRVPPNEEVVFVVHLIDY
PIK55877          GEVIEGMELAISTMRQGELSKFLIAPEFAYGKYGCGKRIPPDSEILMEIELISF
XP_022218357      CDVLEGLQAAVLTMRTYEKAEFIISYKLLFHEMGCPPRIKPRADGLFKIEVLHF
XP_017120033      M-VIEGLEAAVRTMRPYEQAEFIISYKLLFRELGCPPRIKPMADGLFKVEVIDF
XP_022165811      GELLPGLEIGIKTMTIGENARFIVKPELAYRELGCPPRIPPNATILFDVHLVSY
XP_017070534      K-VLEGLEAAVRTMRPYEQAEFIISHKLLFRELGCPPRIKPKADGLFKVEVIDY
XP_001942690      GELIPGLEIGIKTMTTGENARFIIKPELAYRELGCPPRIPPNATVLFDVHLVSF
XP_003883131      GQVISGWDLGVMKMKRGERAMLTIQPGYGYGASGSPPVIPPNAVLKFDVELLDS
XP_014091483      E-VIEGLEEAVRTMYRGEQAQFVISHHLLFREIGCPPRIKPSADGLFIIELVSF
XP_015376375      GELLPGLEIGIKTMTTGENSRFIIKPELAYRELGCPPRIPPNATILFDVHLVSF
KOF66856          GKAIPGWEIALLTMKKGEISWFLIQPEYGFQETGCPPRVPRNATALFEIEILSI
XP_015431627      GSLLPGLEIAISTMQKHEIAIFIVHSDLAYGRFGCAPRIPPNEQILFIVHLIDY
ORX48915          GEVIRAWDIGIATIRRGEQASLLCAPEYAYGESGSPPSIPGNSTLRFEIEGLNI
XP_001958659      K-VIEGLEAAVLTMRPYEQAEFIISYKLLFRELGCPPRIKPKADGLFKVEVIDF
XP_013077159      GASILGLELAILSMAKHEISRFLIHYSYGYGEMGCPPRIPPKATLIIDLEVMSF
XP_013077158      GASILGLELAILSMAKHEISRFLIHYSYGYGEMGCPPRIPPKATLIIDLEVMSF
XP_016974015      M-VLEGLEAAVRTMRPYEQAEFIISYKLLFRELGCPPRIKPKADGLFKVEVIDF
KFG33606          GQVIRGWDLGVMKMKRGERAMLTIQPDYGYGASGSPPVIPPNSVLKFDVELLDS
XP_017045419      M-VIEGLEAAVRTMRPYEQAEFIISYQLLFRELGCPPRIKPKADGLFKVEVIDY
PUA87907          GQVIRGWDLGVMKMKRGERAMLTIQPDYGYGASGSPPVIPPNSVLKFDVELLDS
XP_017001929      M-VIVGLESAVRTMRPYEQAEFIISYKLLFGELGCPPRIKPKADGLLKVEVIDY
XP_009012598      MTTLVGFSKALESMRKGEKASFYIAPQYAFGKLGCPKKIPPNATCLYIIELIDY
XP_008888049      GQVIRGWDLGVMKMKRGERAMLTIQPDYGYGASGSPPVIPPNSVLKFDVELLDS
XP_023297605      E-VLEGLEVAVRTMHKGEHAQFVISYHLLFREMGCPPRVKPKADGLFIIELVSY
XP_002594856      GSVVPGMEIAISSMRKGERSRFLVQPNYGYGKFGCPPRIPGNATILYEIEMLNF
XP_018896035      GPLLRGLDIGVGTMKTGEVSWFLVHHDYAYGKLGCGNRIPKEATILFEVTLVSY
XP_004517316      E-VIEGLEAAVRTMYRGEQAQFVISYHLLFREFGCPPRIKPRADGLFIIELVSF
XP_017148191      CDVLEGLQAAVLTMRPYEKAEFIISYKLLFHEIGCPPRIKPRSDGLFKIEVLDF
XP_002412109      DVMVLGLAKALMTMRKGERARVLVQPEYAFGKMGCAPRIPGNATILYEVELLHF
XP_014507068      GQVIKGWDEGIKTMKKGENALFTIPPELAYGESGSPPTIPPNATLQFDVELLSW
XP_011298074      GQLIPGFELAIKSMKKHEVSLFWVDFDYAFGELGCAPLIPPRADIIFLVSLIDY
ESS30489          GQVIRGWDLGVMKMKRGERAMLTIQPDYGYGASGSPPVIPPNSVLKFDVELLDS
EPR59296          GQVIRGWDLGVMKMKRGERAMLTIQPDYGYGASGSPPVIPPNSVLKFDVELLDS
OAJ38225          GMVIKGWDVGLATMRVGEKASLLIYPEYGYGPQGSPPKIPGNSTLLFDVELVSA
XP_018637740      GQVIRGWDLGVMKMKRGERAMLTIQPDYGYGASGSPPVIPPNSVLKFDVELLDS
KYK63140          GQVIRGWDLGVMKMKRGERAMLTIQPDYGYGASGSPPVIPPNSVLKFDVELLDS
CEL73032          GQVIRGWDLGVMKMKRGERAMLTIQPDYGYGASGSPPVIPPNSVLKFDVELLDS
AAX51680          GQVIRGWDLGVMKMKRGERAMLTIQPDYGYGASGSPPVIPPNSVLKFDVELLDS
AFJ24780          GQLLTGLDIALQSMCKKERANFVFDSSVMFGELGVPPRIPGGADIYAEVEVIDF
NP_611837         T-VVEGLEVAVRSMRPYEQAEFIISYKLLFGELGCPPRIKPKADALFKVEVIDY
XP_012342118      GMLIPGLEIGITTMQKHEIAMFIIHPDLAYGKYGCAPRIPPNEEILFIVHLTDY
XP_002082854      T-VVEGLEAAVRSMRPYEQAEFIISYKLLFGELGCPPRIKPKADALFKVEVIDY
XP_002040150      T-VVEGLEAAVRSMRPYEQAEFIISYKLLFGELGCPPRIKPKADALFKVEVIDY
XP_021954336      GELLLGVEFGVKTMRVGEISKFLFEPEYAYGKLGCPTVIPSNSRILMVIEMVRA
XP_001357527      CDVLEGLQAAVLTMRPYEKAEFIISYKLLFHEMGCPPRIKPRSDGLFKIEVLHF
XP_002015409      CDVLEGLQAAVLTMRPYEKAEFIISYKLLFHEMGCPPRIKPRSDGLFKIEVLHF
XP_017469679      E-VIEGLEAAVRTMYRGEQAQFVISYQLLFREIGCPPRVKPRADGLFIIELVSF
XP_020810045      M-VLEGLEAAVRTMRPYEQAEFIISYKLLFRELGCPPRIKPKADGLFKVEVIDY
                       .    : .:   : .             *    .
```
