# Supplementary material for: Compression of Large Sets of Sequence Data Reveals Fine Diversification of Functional Profiles in Multigene Families of Proteins: A Study for Peptidyl-Prolyl cis/trans Isomerases (PPIase)
Source: Biomolecules. 2019 Feb 11;9(2):59. doi: 10.3390/biom9020059 (PMC6406343; doi:10.3390/biom9020059)
Supplement: Supplementary file 1 [file biomolecules-09-00059-s001.zip › Figure S5B-FKBP36.nam.pdf]

List of entries for analyses of FKBP36 – only FKBD domains

(1) NP_003593 inactive peptidyl-prolyl cis-trans isomerase FKBP6 isoform a [Homo sapiens].
(2) XP_003815624 inactive peptidyl-prolyl cis-trans isomerase FKBP6 isoform X1 [Pan paniscus].
(3) XP_003777068 inactive peptidyl-prolyl cis-trans isomerase FKBP6 isoform X1 [Pongo abelii].
(4) XP_003276647 PREDICTED: inactive peptidyl-prolyl cis-trans isomerase FKBP6 [Nomascus leuco
(5) XP_003895916 inactive peptidyl-prolyl cis-trans isomerase FKBP6 isoform X1 [Papio anubis].
(6) EHH17242 Peptidyl-prolyl cis-trans isomerase FKBP6 [Macaca mulatta].
(7) XP_001110932 PREDICTED: inactive peptidyl-prolyl cis-trans isomerase FKBP6 isoform X1 [Mac
(8) XP_011785750 PREDICTED: inactive peptidyl-prolyl cis-trans isomerase FKBP6 isoform X1 [Col
(9) NP_001128683 inactive peptidyl-prolyl cis-trans isomerase FKBP6 isoform b [Homo sapiens].
(10) XP_017713171 PREDICTED: inactive peptidyl-prolyl cis-trans isomerase FKBP6 isoform X1 [Rhi
(11) XP_010385752 PREDICTED: inactive peptidyl-prolyl cis-trans isomerase FKBP6 isoform X1 [Rhi
(12) XP_002817917 inactive peptidyl-prolyl cis-trans isomerase FKBP6 isoform X2 [Pongo abelii].
(13) XP_004045599 PREDICTED: inactive peptidyl-prolyl cis-trans isomerase FKBP6 isoform X1 [Gor
(14) XP_008979905 PREDICTED: inactive peptidyl-prolyl cis-trans isomerase FKBP6 isoform X2 [Cal
(15) XP_016868230 inactive peptidyl-prolyl cis-trans isomerase FKBP6 isoform X1 [Homo sapiens].
(16) XP_003794239 inactive peptidyl-prolyl cis-trans isomerase FKBP6 isoform X1 [Otolemur garne
(17) XP_012597698 inactive peptidyl-prolyl cis-trans isomerase FKBP6 isoform X1 [Microcebus mur
(18) XP_011931834 PREDICTED: inactive peptidyl-prolyl cis-trans isomerase FKBP6 isoform X2 [Cer
(19) XP_003895917 inactive peptidyl-prolyl cis-trans isomerase FKBP6 isoform X2 [Papio anubis].
(20) XP_008016547 PREDICTED: LOW QUALITY PROTEIN: inactive peptidyl-prolyl cis-trans isomerase
(21) XP_003934167 PREDICTED: inactive peptidyl-prolyl cis-trans isomerase FKBP6 isoform X1 [Sai
(22) XP_012495716 PREDICTED: inactive peptidyl-prolyl cis-trans isomerase FKBP6 isoform X1 [Pro
(23) XP_012304459 inactive peptidyl-prolyl cis-trans isomerase FKBP6 isoform X1 [Aotus nancymaa
(24) XP_022357643 inactive peptidyl-prolyl cis-trans isomerase FKBP6 isoform X1 [Enhydra lutris
(25) XP_010385754 PREDICTED: inactive peptidyl-prolyl cis-trans isomerase FKBP6 isoform X3 [Rhi
(26) XP_017895560 PREDICTED: inactive peptidyl-prolyl cis-trans isomerase FKBP6 isoform X2 [Cap
(27) XP_005892900 PREDICTED: inactive peptidyl-prolyl cis-trans isomerase FKBP6 isoform X2 [Bos
(28) XP_010852603 PREDICTED: inactive peptidyl-prolyl cis-trans isomerase FKBP6 isoform X2 [Bis
(29) XP_546928 inactive peptidyl-prolyl cis-trans isomerase FKBP6 [Canis lupus familiaris].
(30) XP_005225237 inactive peptidyl-prolyl cis-trans isomerase FKBP6 isoform X1 [Bos taurus].
(31) XP_021556246 inactive peptidyl-prolyl cis-trans isomerase FKBP6 isoform X1 [Neomonachus sc
(32) XP_011978492 PREDICTED: inactive peptidyl-prolyl cis-trans isomerase FKBP6 isoform X1 [Ovi
(33) NP_001296390 inactive peptidyl-prolyl cis-trans isomerase FKBP6 [Equus caballus].
(34) XP_011959859 PREDICTED: inactive peptidyl-prolyl cis-trans isomerase FKBP6 isoform X1 [Ovi
(35) XP_006942097 inactive peptidyl-prolyl cis-trans isomerase FKBP6 [Felis catus].
(36) XP_007100028 inactive peptidyl-prolyl cis-trans isomerase FKBP6 isoform X2 [Physeter catod
(37) XP_005972253 PREDICTED: peptidyl-prolyl cis-trans isomerase FKBP6 isoform X2 [Pantholops h
(38) XP_019277206 PREDICTED: inactive peptidyl-prolyl cis-trans isomerase FKBP6 [Panthera pardu
(39) XP_014705926 PREDICTED: inactive peptidyl-prolyl cis-trans isomerase FKBP6 [Equus asinus].
(40) XP_008540619 PREDICTED: inactive peptidyl-prolyl cis-trans isomerase FKBP6 [Equus przewals
(41) XP_020752493 inactive peptidyl-prolyl cis-trans isomerase FKBP6 isoform X1 [Odocoileus vir
(42) XP_014988800 PREDICTED: inactive peptidyl-prolyl cis-trans isomerase FKBP6 isoform X2 [Mac
(43) XP_011785755 PREDICTED: inactive peptidyl-prolyl cis-trans isomerase FKBP6 isoform X2 [Col
(44) XP_022444356 inactive peptidyl-prolyl cis-trans isomerase FKBP6 isoform X2 [Delphinapterus
(45) XP_008979887 PREDICTED: inactive peptidyl-prolyl cis-trans isomerase FKBP6 isoform X1 [Cal
(46) XP_004399150 PREDICTED: inactive peptidyl-prolyl cis-trans isomerase FKBP6 isoform X1 [Odo
(47) XP_004268847 PREDICTED: inactive peptidyl-prolyl cis-trans isomerase FKBP6 isoform X1 [Orc
(48) XP_019659608 PREDICTED: inactive peptidyl-prolyl cis-trans isomerase FKBP6 [Ailuropoda mel
(49) XP_024602986 inactive peptidyl-prolyl cis-trans isomerase FKBP6 isoform X1 [Neophocaena as
(50) XP_007186614 PREDICTED: peptidyl-prolyl cis-trans isomerase FKBP6 isoform X1 [Balaenoptera
(51) XP_014652016 PREDICTED: inactive peptidyl-prolyl cis-trans isomerase FKBP6 [Ceratotherium
(52) XP_012597701 inactive peptidyl-prolyl cis-trans isomerase FKBP6 isoform X2 [Microcebus mur
(53) XP_004761711 PREDICTED: inactive peptidyl-prolyl cis-trans isomerase FKBP6 isoform X1 [Mus
(54) XP_004317177 PREDICTED: inactive peptidyl-prolyl cis-trans isomerase FKBP6 isoform X1 [Tur

(55) XP_005892901 PREDICTED: inactive peptidyl-prolyl cis-trans isomerase FKBP6 isoform X1 [Bos
(56) XP_017895559 PREDICTED: inactive peptidyl-prolyl cis-trans isomerase FKBP6 isoform X1 [Cap
(57) XP_005662030 inactive peptidyl-prolyl cis-trans isomerase FKBP6 isoform X1 [Sus scrofa].
(58) XP_010852604 PREDICTED: inactive peptidyl-prolyl cis-trans isomerase FKBP6 isoform X3 [Bis
(59) XP_010852602 PREDICTED: inactive peptidyl-prolyl cis-trans isomerase FKBP6 isoform X1 [Bis
(60) EPY83956 peptidyl-prolyl cis-trans isomerase FKBP6 [Camelus ferus].
(61) NP_001095679 inactive peptidyl-prolyl cis-trans isomerase FKBP6 [Bos taurus].
(62) XP_008139950 PREDICTED: inactive peptidyl-prolyl cis-trans isomerase FKBP6 [Eptesicus fusc
(63) XP_010970644 PREDICTED: inactive peptidyl-prolyl cis-trans isomerase FKBP6 [Camelus bactri
(64) XP_004021020 PREDICTED: inactive peptidyl-prolyl cis-trans isomerase FKBP6 isoform X2 [Ovi
(65) XP_015450070 inactive peptidyl-prolyl cis-trans isomerase FKBP6 [Pteropus alecto].
(66) XP_017516100 PREDICTED: inactive peptidyl-prolyl cis-trans isomerase FKBP6 [Manis javanica
(67) XP_010342933 PREDICTED: inactive peptidyl-prolyl cis-trans isomerase FKBP6 isoform X2 [Sai
(68) XP_006057167 PREDICTED: peptidyl-prolyl cis-trans isomerase FKBP6 [Bubalus bubalis].
(69) XP_019843968 PREDICTED: inactive peptidyl-prolyl cis-trans isomerase FKBP6 [Bos indicus].
(70) XP_006201442 PREDICTED: inactive peptidyl-prolyl cis-trans isomerase FKBP6 [Vicugna pacos]
(71) XP_005972252 PREDICTED: peptidyl-prolyl cis-trans isomerase FKBP6 isoform X1 [Pantholops h
(72) XP_004380900 inactive peptidyl-prolyl cis-trans isomerase FKBP6 [Trichechus manatus latiro
(73) XP_007468850 PREDICTED: peptidyl-prolyl cis-trans isomerase FKBP6 isoform X2 [Lipotes vexi
(74) XP_007100027 inactive peptidyl-prolyl cis-trans isomerase FKBP6 isoform X1 [Physeter catod
(75) XP_008966786 inactive peptidyl-prolyl cis-trans isomerase FKBP6 isoform X2 [Pan paniscus].
(76) XP_020017458 inactive peptidyl-prolyl cis-trans isomerase FKBP6 [Castor canadensis].
(77) XP_006088351 inactive peptidyl-prolyl cis-trans isomerase FKBP6 [Myotis lucifugus].
(78) XP_020752496 inactive peptidyl-prolyl cis-trans isomerase FKBP6 isoform X2 [Odocoileus vir
(79) XP_022444355 inactive peptidyl-prolyl cis-trans isomerase FKBP6 isoform X1 [Delphinapterus
(80) XP_006773483 PREDICTED: inactive peptidyl-prolyl cis-trans isomerase FKBP6 [Myotis davidii
(81) XP_012304461 inactive peptidyl-prolyl cis-trans isomerase FKBP6 isoform X3 [Aotus nancymaa
(82) XP_008591583 PREDICTED: inactive peptidyl-prolyl cis-trans isomerase FKBP6 [Galeopterus va
(83) XP_016054558 PREDICTED: inactive peptidyl-prolyl cis-trans isomerase FKBP6 [Miniopterus na
(84) XP_011369980 inactive peptidyl-prolyl cis-trans isomerase FKBP6 [Pteropus vampyrus].
(85) XP_004268849 PREDICTED: inactive peptidyl-prolyl cis-trans isomerase FKBP6 isoform X2 [Orc
(86) XP_024602987 inactive peptidyl-prolyl cis-trans isomerase FKBP6 isoform X2 [Neophocaena as
(87) XP_019610492 PREDICTED: inactive peptidyl-prolyl cis-trans isomerase FKBP6 [Rhinolophus si
(88) XP_004317179 PREDICTED: inactive peptidyl-prolyl cis-trans isomerase FKBP6 isoform X2 [Tur
(89) XP_006889852 PREDICTED: peptidyl-prolyl cis-trans isomerase FKBP6 [Elephantulus edwardii].
(90) XP_007940604 PREDICTED: inactive peptidyl-prolyl cis-trans isomerase FKBP6 [Orycteropus af
(91) XP_014411577 PREDICTED: inactive peptidyl-prolyl cis-trans isomerase FKBP6 [Camelus ferus]
(92) XP_007073943 PREDICTED: inactive peptidyl-prolyl cis-trans isomerase FKBP6 [Panthera tigri
(93) XP_006155025 PREDICTED: inactive peptidyl-prolyl cis-trans isomerase FKBP6 isoform X1 [Tup
(94) XP_005328656 inactive peptidyl-prolyl cis-trans isomerase FKBP6 [Ictidomys tridecemlineatu
(95) XP_011765609 inactive peptidyl-prolyl cis-trans isomerase FKBP6 isoform X5 [Macaca nemestr
(96) XP_010979058 PREDICTED: inactive peptidyl-prolyl cis-trans isomerase FKBP6 [Camelus dromed
(97) XP_006249193 PREDICTED: inactive peptidyl-prolyl cis-trans isomerase FKBP6 isoform X2 [Rat
(98) XP_002722023 PREDICTED: inactive peptidyl-prolyl cis-trans isomerase FKBP6 [Oryctolagus cu
(99) XP_005344683 PREDICTED: inactive peptidyl-prolyl cis-trans isomerase FKBP6 isoform X2 [Mic
(100) XP_020941978 inactive peptidyl-prolyl cis-trans isomerase FKBP6 isoform X2 [Sus scrofa].
(101) XP_006859898 PREDICTED: peptidyl-prolyl cis-trans isomerase FKBP6 [Chrysochloris asiatica]
(102) XP_005549416 PREDICTED: inactive peptidyl-prolyl cis-trans isomerase FKBP6 isoform X5 [Mac
(103) XP_024427494 inactive peptidyl-prolyl cis-trans isomerase FKBP6 [Desmodus rotundus].
(104) XP_016001971 PREDICTED: inactive peptidyl-prolyl cis-trans isomerase FKBP6 [Rousettus aegy
(105) XP_007468849 PREDICTED: peptidyl-prolyl cis-trans isomerase FKBP6 isoform X1 [Lipotes vexi
(106) XP_014988802 PREDICTED: inactive peptidyl-prolyl cis-trans isomerase FKBP6 isoform X3 [Mac
(107) XP_012584338 PREDICTED: inactive peptidyl-prolyl cis-trans isomerase FKBP6 [Condylura cris
(108) XP_006971358 PREDICTED: inactive peptidyl-prolyl cis-trans isomerase FKBP6 isoform X2 [Per
(109) XP_004620930 PREDICTED: inactive peptidyl-prolyl cis-trans isomerase FKBP6 [Sorex araneus]
(110) XP_008696858 PREDICTED: inactive peptidyl-prolyl cis-trans isomerase FKBP6 [Ursus maritimu

(111) XP_021018812 inactive peptidyl-prolyl cis-trans isomerase FKBP6 isoform X2 [Mus caroli].
(112) XP_012782940 PREDICTED: inactive peptidyl-prolyl cis-trans isomerase FKBP6 [Ochotona princ
(113) XP_019502388 PREDICTED: inactive peptidyl-prolyl cis-trans isomerase FKBP6 isoform X1 [Hip
(114) XP_021042681 inactive peptidyl-prolyl cis-trans isomerase FKBP6 isoform X2 [Mus pahari].
(115) NP_001264821 inactive peptidyl-prolyl cis-trans isomerase FKBP6 isoform 2 [Mus musculus].
(116) XP_021519217 inactive peptidyl-prolyl cis-trans isomerase FKBP6 isoform X2 [Meriones ungui
(117) XP_005344682 PREDICTED: inactive peptidyl-prolyl cis-trans isomerase FKBP6 isoform X1 [Mic
(118) NP_001349718 inactive peptidyl-prolyl cis-trans isomerase FKBP6 isoform d [Homo sapiens].
(119) NP_001099392 inactive peptidyl-prolyl cis-trans isomerase FKBP6 [Rattus norvegicus].
(120) XP_005396843 PREDICTED: inactive peptidyl-prolyl cis-trans isomerase FKBP6 [Chinchilla lan
(121) XP_019502390 PREDICTED: inactive peptidyl-prolyl cis-trans isomerase FKBP6 isoform X2 [Hip
(122) XP_006971357 PREDICTED: inactive peptidyl-prolyl cis-trans isomerase FKBP6 isoform X1 [Per
(123) XP_008966787 inactive peptidyl-prolyl cis-trans isomerase FKBP6 isoform X3 [Pan paniscus].
(124) XP_003500991 PREDICTED: inactive peptidyl-prolyl cis-trans isomerase FKBP6 [Cricetulus gri
(125) XP_008846596 PREDICTED: inactive peptidyl-prolyl cis-trans isomerase FKBP6 [Nannospalax ga
(126) XP_021563404 inactive peptidyl-prolyl cis-trans isomerase FKBP6 [Carlito syrichta].
(127) XP_018887307 PREDICTED: inactive peptidyl-prolyl cis-trans isomerase FKBP6 isoform X2 [Gor
(128) XP_021018806 inactive peptidyl-prolyl cis-trans isomerase FKBP6 isoform X1 [Mus caroli].
(129) XP_017176678 PREDICTED: inactive peptidyl-prolyl cis-trans isomerase FKBP6 isoform X2 [Mus
(130) NP_291049 inactive peptidyl-prolyl cis-trans isomerase FKBP6 isoform 1 [Mus musculus].
(131) XP_021042679 inactive peptidyl-prolyl cis-trans isomerase FKBP6 isoform X1 [Mus pahari].
(132) XP_010617304 PREDICTED: inactive peptidyl-prolyl cis-trans isomerase FKBP6 isoform X1 [Fuk
(133) XP_021519216 inactive peptidyl-prolyl cis-trans isomerase FKBP6 isoform X1 [Meriones ungui
(134) XP_023420891 LOW QUALITY PROTEIN: inactive peptidyl-prolyl cis-trans isomerase FKBP6 [Cavi
(135) XP_006155027 PREDICTED: inactive peptidyl-prolyl cis-trans isomerase FKBP6 isoform X2 [Tup
(136) XP_011765605 inactive peptidyl-prolyl cis-trans isomerase FKBP6 isoform X1 [Macaca nemestr
(137) XP_015302699 PREDICTED: inactive peptidyl-prolyl cis-trans isomerase FKBP6 isoform X1 [Mac
(138) XP_011765611 inactive peptidyl-prolyl cis-trans isomerase FKBP6 isoform X7 [Macaca nemestr
(139) XP_012597702 inactive peptidyl-prolyl cis-trans isomerase FKBP6 isoform X3 [Microcebus mur
(140) XP_014988803 PREDICTED: inactive peptidyl-prolyl cis-trans isomerase FKBP6 isoform X4 [Mac
(141) XP_010617307 PREDICTED: inactive peptidyl-prolyl cis-trans isomerase FKBP6 isoform X2 [Fuk
(142) XP_004666244 PREDICTED: inactive peptidyl-prolyl cis-trans isomerase FKBP6 [Jaculus jaculu
(143) XP_004630630 inactive peptidyl-prolyl cis-trans isomerase FKBP6 [Octodon degus].
(144) XP_004840130 inactive peptidyl-prolyl cis-trans isomerase FKBP6 isoform X1 [Heterocephalus
(145) XP_014403986 PREDICTED: inactive peptidyl-prolyl cis-trans isomerase FKBP6 [Myotis brandti
(146) XP_016800790 inactive peptidyl-prolyl cis-trans isomerase FKBP6 isoform X3 [Pan troglodyte
(147) XP_014988804 PREDICTED: inactive peptidyl-prolyl cis-trans isomerase FKBP6 isoform X5 [Mac
(148) ELK06863 FK506-binding protein 6 [Pteropus alecto].
(149) EHB08283 FK506-binding protein 6 [Heterocephalus glaber].
(150) XP_004706046 PREDICTED: inactive peptidyl-prolyl cis-trans isomerase FKBP6 [Echinops telfa
(151) XP_021110082 inactive peptidyl-prolyl cis-trans isomerase FKBP6 isoform X2 [Heterocephalus
(152) XP_007518064 PREDICTED: inactive peptidyl-prolyl cis-trans isomerase FKBP6 [Erinaceus euro
(153) PNI92595 FKBP6 isoform 5, partial [Pan troglodytes].
(154) XP_020861399 inactive peptidyl-prolyl cis-trans isomerase FKBP6 isoform X1 [Phascolarctos
(155) XP_016284889 PREDICTED: inactive peptidyl-prolyl cis-trans isomerase FKBP6 isoform X2 [Mon
(156) XP_016284888 PREDICTED: inactive peptidyl-prolyl cis-trans isomerase FKBP6 isoform X1 [Mon
(157) XP_001519210 PREDICTED: inactive peptidyl-prolyl cis-trans isomerase FKBP6 isoform X2 [Orn
(158) XP_020861404 inactive peptidyl-prolyl cis-trans isomerase FKBP6 isoform X2 [Phascolarctos
(159) XP_014425414 inactive peptidyl-prolyl cis-trans isomerase FKBP6, partial [Pelodiscus sinen
(160) XP_004761713 PREDICTED: inactive peptidyl-prolyl cis-trans isomerase FKBP6 isoform X2 [Mus
(161) XP_007441600 inactive peptidyl-prolyl cis-trans isomerase FKBP6 [Python bivittatus].
(162) XP_016080873 PREDICTED: inactive peptidyl-prolyl cis-trans isomerase FKBP6 isoform X1 [Orn
(163) XP_024066689 inactive peptidyl-prolyl cis-trans isomerase FKBP6 isoform X1 [Terrapene mexi
(164) XP_019365238 PREDICTED: inactive peptidyl-prolyl cis-trans isomerase FKBP6, partial [Gavia
(165) XP_019401968 PREDICTED: inactive peptidyl-prolyl cis-trans isomerase FKBP6 isoform X1 [Cro
(166) XP_013926437 PREDICTED: inactive peptidyl-prolyl cis-trans isomerase FKBP6 isoform X1 [Tha

(167) XP_019401969 PREDICTED: inactive peptidyl-prolyl cis-trans isomerase FKBP6 isoform X2 [Cro
(168) XP_015269029 PREDICTED: inactive peptidyl-prolyl cis-trans isomerase FKBP6 [Gekko japonicu
(169) XP_014464913 PREDICTED: inactive peptidyl-prolyl cis-trans isomerase FKBP6 isoform X2 [All
(170) XP_006258869 PREDICTED: inactive peptidyl-prolyl cis-trans isomerase FKBP6 isoform X1 [All
(171) XP_005344684 PREDICTED: inactive peptidyl-prolyl cis-trans isomerase FKBP6 isoform X3 [Mic
(172) XP_015670323 PREDICTED: inactive peptidyl-prolyl cis-trans isomerase FKBP6 [Protobothrops
(173) XP_020662988 inactive peptidyl-prolyl cis-trans isomerase FKBP6 isoform X1 [Pogona vittice
(174) XP_007065519 PREDICTED: peptidyl-prolyl cis-trans isomerase FKBP6 [Chelonia mydas].
(175) XP_020662989 inactive peptidyl-prolyl cis-trans isomerase FKBP6 isoform X2 [Pogona vittice
(176) XP_020662990 inactive peptidyl-prolyl cis-trans isomerase FKBP6 isoform X3 [Pogona vittice
(177) XP_025051231 inactive peptidyl-prolyl cis-trans isomerase FKBP6, partial [Alligator sinens
(178) KYO33272 inactive peptidyl-prolyl cis-trans isomerase FKBP6 [Alligator mississippiensi
(179) XP_021018814 inactive peptidyl-prolyl cis-trans isomerase FKBP6 isoform X3 [Mus caroli].
(180) OBS57796 hypothetical protein A6R68_11069 [Neotoma lepida].
(181) NP_001264822 inactive peptidyl-prolyl cis-trans isomerase FKBP6 isoform 3 [Mus musculus].
(182) XP_021042683 inactive peptidyl-prolyl cis-trans isomerase FKBP6 isoform X4 [Mus pahari].
(183) XP_021519218 inactive peptidyl-prolyl cis-trans isomerase FKBP6 isoform X3 [Meriones ungui
(184) XP_024653358 inactive peptidyl-prolyl cis-trans isomerase FKBP6 isoform X3 [Macaca nemestr
(185) XP_009678488 PREDICTED: inactive peptidyl-prolyl cis-trans isomerase FKBP6 [Struthio camel
(186) XP_015302701 PREDICTED: inactive peptidyl-prolyl cis-trans isomerase FKBP6 isoform X2 [Mac
(187) XP_003770271 inactive peptidyl-prolyl cis-trans isomerase FKBP6 [Sarcophilus harrisii].
(188) ETE73718 Peptidyl-prolyl cis-trans isomerase FKBP6, partial [Ophiophagus hannah].
(189) NP_989052 inactive peptidyl-prolyl cis-trans isomerase FKBP6 [Xenopus tropicalis].
(190) NP_001085204 FK506 binding protein 6 L homeolog [Xenopus laevis].
(191) XP_011592867 PREDICTED: inactive peptidyl-prolyl cis-trans isomerase FKBP6 [Aquila chrysae
(192) XP_010584273 PREDICTED: inactive peptidyl-prolyl cis-trans isomerase FKBP6 [Haliaeetus leu
(193) XP_017946629 PREDICTED: inactive peptidyl-prolyl cis-trans isomerase FKBP6 isoform X1 [Xen
(194) XP_008109385 PREDICTED: inactive peptidyl-prolyl cis-trans isomerase FKBP6 isoform X1 [Ano
(195) XP_021271278 inactive peptidyl-prolyl cis-trans isomerase FKBP6 [Numida meleagris].
(196) XP_023074397 inactive peptidyl-prolyl cis-trans isomerase FKBP6 [Piliocolobus tephrosceles
(197) XP_015151391 inactive peptidyl-prolyl cis-trans isomerase FKBP6 isoform X1 [Gallus gallus]
(198) XP_018100492 PREDICTED: FK506 binding protein 6 L homeolog isoform X1 [Xenopus laevis].
(199) POI24993 hypothetical protein CIB84_011258 [Bambusicola thoracicus].
(200) PNI92596 FKBP6 isoform 6 [Pan troglodytes].
(201) XP_015736491 PREDICTED: inactive peptidyl-prolyl cis-trans isomerase FKBP6 [Coturnix japon
(202) XP_010283169 PREDICTED: inactive peptidyl-prolyl cis-trans isomerase FKBP6, partial [Phaet
(203) PNJ85879 FKBP6 isoform 6 [Pongo abelii].
(204) XP_018412624 PREDICTED: inactive peptidyl-prolyl cis-trans isomerase FKBP6, partial [Nanor
(205) XP_010211497 PREDICTED: inactive peptidyl-prolyl cis-trans isomerase FKBP6 [Tinamus guttat
(206) XP_017388864 PREDICTED: inactive peptidyl-prolyl cis-trans isomerase FKBP6 [Cebus capucinu
(207) XP_008109386 PREDICTED: inactive peptidyl-prolyl cis-trans isomerase FKBP6 isoform X2 [Ano
(208) XP_014342809 PREDICTED: inactive peptidyl-prolyl cis-trans isomerase FKBP6 [Latimeria chal
(209) KFV84016 Peptidyl-prolyl cis-trans isomerase FKBP6, partial [Struthio camelus australi
(210) XP_013812139 PREDICTED: inactive peptidyl-prolyl cis-trans isomerase FKBP6 [Apteryx austra
(211) XP_009643249 PREDICTED: inactive peptidyl-prolyl cis-trans isomerase FKBP6 [Egretta garzet
(212) XP_009928379 PREDICTED: inactive peptidyl-prolyl cis-trans isomerase FKBP6, partial [Halia
(213) XP_010124464 PREDICTED: inactive peptidyl-prolyl cis-trans isomerase FKBP6, partial [Chlam
(214) XP_009321903 PREDICTED: inactive peptidyl-prolyl cis-trans isomerase FKBP6 [Pygoscelis ade
(215) XP_009807780 PREDICTED: inactive peptidyl-prolyl cis-trans isomerase FKBP6, partial [Gavia
(216) XP_009512874 PREDICTED: inactive peptidyl-prolyl cis-trans isomerase FKBP6, partial [Phala
(217) XP_009586283 PREDICTED: inactive peptidyl-prolyl cis-trans isomerase FKBP6, partial [Fulma
(218) KFQ72829 Peptidyl-prolyl cis-trans isomerase FKBP6, partial [Phaethon lepturus].
(219) XP_009698973 PREDICTED: inactive peptidyl-prolyl cis-trans isomerase FKBP6, partial [Caria
(220) XP_009901738 PREDICTED: inactive peptidyl-prolyl cis-trans isomerase FKBP6, partial [Picoi
(221) XP_012887159 PREDICTED: inactive peptidyl-prolyl cis-trans isomerase FKBP6 [Dipodomys ordi
(222) XP_009940812 PREDICTED: inactive peptidyl-prolyl cis-trans isomerase FKBP6 [Opisthocomus h

(223) XP_002187983 PREDICTED: inactive peptidyl-prolyl cis-trans isomerase FKBP6 [Taeniopygia gu
(224) XP_009887190 PREDICTED: inactive peptidyl-prolyl cis-trans isomerase FKBP6 [Charadrius voc
(225) XP_009486380 PREDICTED: inactive peptidyl-prolyl cis-trans isomerase FKBP6, partial [Pelec
(226) KGL76252 Peptidyl-prolyl cis-trans isomerase FKBP6, partial [Tinamus guttatus].
(227) XP_010306733 PREDICTED: inactive peptidyl-prolyl cis-trans isomerase FKBP6, partial [Balea
(228) XP_008979919 PREDICTED: inactive peptidyl-prolyl cis-trans isomerase FKBP6 isoform X3 [Cal
(229) XP_009998510 PREDICTED: inactive peptidyl-prolyl cis-trans isomerase FKBP6 [Chaetura pelag
(230) XP_009463122 PREDICTED: inactive peptidyl-prolyl cis-trans isomerase FKBP6 [Nipponia nippo
(231) XP_007894626 PREDICTED: inactive peptidyl-prolyl cis-trans isomerase FKBP6 [Callorhinchus
(232) XP_010022120 PREDICTED: inactive peptidyl-prolyl cis-trans isomerase FKBP6, partial [Nesto
(233) XP_010083598 PREDICTED: inactive peptidyl-prolyl cis-trans isomerase FKBP6, partial [Ptero
(234) XP_010194269 PREDICTED: inactive peptidyl-prolyl cis-trans isomerase FKBP6, partial [Coliu
(235) XP_009962683 PREDICTED: inactive peptidyl-prolyl cis-trans isomerase FKBP6, partial [Tyto
(236) KFP29338 Peptidyl-prolyl cis-trans isomerase FKBP6, partial [Colius striatus].
(237) XP_010147897 PREDICTED: inactive peptidyl-prolyl cis-trans isomerase FKBP6 [Eurypyga helia
(238) KFQ39479 Peptidyl-prolyl cis-trans isomerase FKBP6, partial [Mesitornis unicolor].
(239) EOB04024 FK506-binding protein 6, partial [Anas platyrhynchos].
(240) XP_023675251 inactive peptidyl-prolyl cis-trans isomerase FKBP6 [Paramormyrops kingsleyae]
(241) XP_009557002 PREDICTED: inactive peptidyl-prolyl cis-trans isomerase FKBP6 [Cuculus canoru
(242) XP_008944700 PREDICTED: inactive peptidyl-prolyl cis-trans isomerase FKBP6, partial [Merop
(243) KFP93140 Peptidyl-prolyl cis-trans isomerase FKBP6, partial [Haliaeetus albicilla].
(244) KFP44723 Peptidyl-prolyl cis-trans isomerase FKBP6, partial [Chlamydotis macqueenii].
(245) XP_017553040 PREDICTED: inactive peptidyl-prolyl cis-trans isomerase FKBP6 isoform X1 [Pyg
(246) XP_021396070 inactive peptidyl-prolyl cis-trans isomerase FKBP6 [Lonchura striata domestic
(247) XP_010143455 PREDICTED: inactive peptidyl-prolyl cis-trans isomerase FKBP6, partial [Bucer
(248) KFQ87275 Peptidyl-prolyl cis-trans isomerase FKBP6, partial [Phoenicopterus ruber rube
(249) XP_010891852 PREDICTED: inactive peptidyl-prolyl cis-trans isomerase FKBP6 isoform X2 [Eso
(250) XP_009863922 PREDICTED: inactive peptidyl-prolyl cis-trans isomerase FKBP6, partial [Apalo
(251) KFW66808 Peptidyl-prolyl cis-trans isomerase FKBP6, partial [Pygoscelis adeliae].
(252) XP_018591809 PREDICTED: inactive peptidyl-prolyl cis-trans isomerase FKBP6, partial [Scler
(253) KFP21064 Peptidyl-prolyl cis-trans isomerase FKBP6, partial [Egretta garzetta].
(254) KFZ64979 Peptidyl-prolyl cis-trans isomerase FKBP6, partial [Podiceps cristatus].
(255) KFW73903 Peptidyl-prolyl cis-trans isomerase FKBP6, partial [Phalacrocorax carbo].
(256) KFM05579 Peptidyl-prolyl cis-trans isomerase FKBP6, partial [Aptenodytes forsteri].
(257) KFV69563 Peptidyl-prolyl cis-trans isomerase FKBP6, partial [Picoides pubescens].
(258) XP_023823731 inactive peptidyl-prolyl cis-trans isomerase FKBP6 isoform X3 [Salvelinus alp
(259) XP_016346087 PREDICTED: inactive peptidyl-prolyl cis-trans isomerase FKBP6-like [Sinocyclo
(260) KFV51798 Peptidyl-prolyl cis-trans isomerase FKBP6, partial [Gavia stellata].
(261) KFW03341 Peptidyl-prolyl cis-trans isomerase FKBP6, partial [Eurypyga helias].
(262) KFV99355 Peptidyl-prolyl cis-trans isomerase FKBP6, partial [Fulmarus glacialis].
(263) OXB56102 hypothetical protein ASZ78_007956 [Callipepla squamata].
(264) XP_018958387 PREDICTED: LOW QUALITY PROTEIN: inactive peptidyl-prolyl cis-trans isomerase
(265) XP_019903677 PREDICTED: inactive peptidyl-prolyl cis-trans isomerase FKBP6 isoform X3 [Eso
(266) KFU91562 Peptidyl-prolyl cis-trans isomerase FKBP6, partial [Chaetura pelagica].
(267) XP_017314960 PREDICTED: inactive peptidyl-prolyl cis-trans isomerase FKBP6 isoform X1 [Ict
(268) XP_016393789 PREDICTED: inactive peptidyl-prolyl cis-trans isomerase FKBP6-like [Sinocyclo
(269) XP_009093115 PREDICTED: inactive peptidyl-prolyl cis-trans isomerase FKBP6 isoform X1 [Ser
(270) XP_023869471 inactive peptidyl-prolyl cis-trans isomerase FKBP6-like isoform X1 [Salvelinu
(271) XP_019903679 PREDICTED: inactive peptidyl-prolyl cis-trans isomerase FKBP6 isoform X5 [Eso
(272) XP_019903678 PREDICTED: inactive peptidyl-prolyl cis-trans isomerase FKBP6 isoform X4 [Eso
(273) XP_014110391 PREDICTED: inactive peptidyl-prolyl cis-trans isomerase FKBP6 [Pseudopodoces
(274) XP_017314964 PREDICTED: inactive peptidyl-prolyl cis-trans isomerase FKBP6 isoform X2 [Ict
(275) XP_019903676 PREDICTED: inactive peptidyl-prolyl cis-trans isomerase FKBP6 isoform X1 [Eso
(276) XP_015502317 PREDICTED: inactive peptidyl-prolyl cis-trans isomerase FKBP6 isoform X2 [Par
(277) XP_020341240 inactive peptidyl-prolyl cis-trans isomerase FKBP6 isoform X2 [Oncorhynchus k
(278) NP_001122145 peptidyl-prolyl cis-trans isomerase FKBP6 [Danio rerio].

(279) OWK52486 Inactive peptidyl-prolyl cis-trans isomerase FKBP6 [Lonchura striata domestic
(280) XP_013992803 PREDICTED: inactive peptidyl-prolyl cis-trans isomerase FKBP6-like isoform X2
(281) XP_024287707 inactive peptidyl-prolyl cis-trans isomerase FKBP6 isoform X1 [Oncorhynchus t
(282) AAH54673 Si:dkey-189h5.5 protein, partial [Danio rerio].
(283) KFP60963 Peptidyl-prolyl cis-trans isomerase FKBP6, partial [Cariama cristata].
(284) XP_018958370 PREDICTED: inactive peptidyl-prolyl cis-trans isomerase FKBP6-like [Cyprinus
(285) KGL96380 Peptidyl-prolyl cis-trans isomerase FKBP6, partial [Charadrius vociferus].
(286) XP_016148031 PREDICTED: inactive peptidyl-prolyl cis-trans isomerase FKBP6-like [Sinocyclo
(287) XP_007245276 inactive peptidyl-prolyl cis-trans isomerase FKBP6 isoform X2 [Astyanax mexic
(288) XP_023794776 inactive peptidyl-prolyl cis-trans isomerase FKBP6 isoform X1 [Cyanistes caer
(289) XP_021479427 inactive peptidyl-prolyl cis-trans isomerase FKBP6-like isoform X2 [Oncorhync
(290) KFR16943 Peptidyl-prolyl cis-trans isomerase FKBP6, partial [Opisthocomus hoazin].
(291) XP_009988051 PREDICTED: inactive peptidyl-prolyl cis-trans isomerase FKBP6, partial [Taura
(292) KFV16869 Peptidyl-prolyl cis-trans isomerase FKBP6, partial [Pterocles gutturalis].
(293) KFO13846 Peptidyl-prolyl cis-trans isomerase FKBP6, partial [Balearica regulorum gibbe
(294) XP_020373313 inactive peptidyl-prolyl cis-trans isomerase FKBP6 [Rhincodon typus].
(295) XP_006640997 PREDICTED: inactive peptidyl-prolyl cis-trans isomerase FKBP6 [Lepisosteus oc
(296) XP_020341239 inactive peptidyl-prolyl cis-trans isomerase FKBP6 isoform X1 [Oncorhynchus k
(297) KQK73326 Peptidyl-prolyl cis-trans isomerase FKBP6 [Amazona aestiva].
(298) XP_008500186 PREDICTED: inactive peptidyl-prolyl cis-trans isomerase FKBP6 [Calypte anna].
(299) XP_013992801 PREDICTED: inactive peptidyl-prolyl cis-trans isomerase FKBP6-like isoform X1
(300) XP_016350445 PREDICTED: inactive peptidyl-prolyl cis-trans isomerase FKBP6-like [Sinocyclo
(301) KFV47875 Peptidyl-prolyl cis-trans isomerase FKBP6, partial [Tyto alba].
(302) XP_022534491 inactive peptidyl-prolyl cis-trans isomerase FKBP6 isoform X1 [Astyanax mexic
(303) XP_021479426 inactive peptidyl-prolyl cis-trans isomerase FKBP6-like isoform X1 [Oncorhync
(304) KFQ96759 Peptidyl-prolyl cis-trans isomerase FKBP6, partial [Nipponia nippon].
(305) XP_012685286 PREDICTED: inactive peptidyl-prolyl cis-trans isomerase FKBP6 [Clupea harengu
(306) KFQ41466 Peptidyl-prolyl cis-trans isomerase FKBP6, partial [Nestor notabilis].
(307) XP_024997748 inactive peptidyl-prolyl cis-trans isomerase FKBP6 isoform X2 [Gallus gallus]
(308) KFQ35533 Peptidyl-prolyl cis-trans isomerase FKBP6, partial [Merops nubicus].
(309) XP_020459257 inactive peptidyl-prolyl cis-trans isomerase FKBP6 isoform X1 [Monopterus alb
(310) XP_017264554 inactive peptidyl-prolyl cis-trans isomerase FKBP6 isoform X2 [Kryptolebias m
(311) XP_005917667 PREDICTED: inactive peptidyl-prolyl cis-trans isomerase FKBP6 isoform X2 [Hap
(312) XP_022621130 inactive peptidyl-prolyl cis-trans isomerase FKBP6 [Seriola dumerili].
(313) XP_005725080 PREDICTED: inactive peptidyl-prolyl cis-trans isomerase FKBP6 isoform X4 [Pun
(314) XP_010408101 inactive peptidyl-prolyl cis-trans isomerase FKBP6 isoform X1 [Corvus cornix
(315) KFO74526 Peptidyl-prolyl cis-trans isomerase FKBP6, partial [Cuculus canorus].
(316) XP_005449739 PREDICTED: inactive peptidyl-prolyl cis-trans isomerase FKBP6 isoform X3 [Ore
(317) XP_023285908 inactive peptidyl-prolyl cis-trans isomerase FKBP6 [Seriola lalandi dorsalis]
(318) XP_015502316 PREDICTED: inactive peptidyl-prolyl cis-trans isomerase FKBP6 isoform X1 [Par
(319) XP_004542108 inactive peptidyl-prolyl cis-trans isomerase FKBP6 isoform X2 [Maylandia zebr
(320) XP_023823730 E3 ubiquitin-protein ligase rififylin isoform X2 [Salvelinus alpinus].
(321) XP_020459258 inactive peptidyl-prolyl cis-trans isomerase FKBP6 isoform X2 [Monopterus alb
(322) XP_008634949 PREDICTED: inactive peptidyl-prolyl cis-trans isomerase FKBP6 isoform X1 [Cor
(323) XP_013883182 PREDICTED: inactive peptidyl-prolyl cis-trans isomerase FKBP6 [Austrofundulus
(324) XP_019947232 PREDICTED: inactive peptidyl-prolyl cis-trans isomerase FKBP6 [Paralichthys o
(325) KFP85199 Peptidyl-prolyl cis-trans isomerase FKBP6, partial [Apaloderma vittatum].
(326) PWA27518 hypothetical protein CCH79_00000105, partial [Gambusia affinis].
(327) XP_003971664 PREDICTED: inactive peptidyl-prolyl cis-trans isomerase FKBP6 [Takifugu rubri
(328) XP_005917666 PREDICTED: inactive peptidyl-prolyl cis-trans isomerase FKBP6 isoform X1 [Hap
(329) XP_013764145 PREDICTED: inactive peptidyl-prolyl cis-trans isomerase FKBP6 isoform X3 [Pun
(330) XP_013764144 PREDICTED: inactive peptidyl-prolyl cis-trans isomerase FKBP6 isoform X2 [Pun
(331) CAG01803 unnamed protein product, partial [Tetraodon nigroviridis].
(332) XP_005449738 PREDICTED: inactive peptidyl-prolyl cis-trans isomerase FKBP6 isoform X2 [Ore
(333) KFV20040 Peptidyl-prolyl cis-trans isomerase FKBP6, partial [Tauraco erythrolophus].
(334) XP_018551539 PREDICTED: inactive peptidyl-prolyl cis-trans isomerase FKBP6 [Lates calcarif

(335) XP_004542106 inactive peptidyl-prolyl cis-trans isomerase FKBP6 isoform X1 [Maylandia zebr
(336) XP_005808385 inactive peptidyl-prolyl cis-trans isomerase FKBP6 isoform X2 [Xiphophorus ma
(337) KFO85715 Peptidyl-prolyl cis-trans isomerase FKBP6, partial [Buceros rhinoceros silves
(338) XP_015236136 PREDICTED: inactive peptidyl-prolyl cis-trans isomerase FKBP6 [Cyprinodon var
(339) XP_023794777 inactive peptidyl-prolyl cis-trans isomerase FKBP6 isoform X2 [Cyanistes caer
(340) XP_015803473 PREDICTED: inactive peptidyl-prolyl cis-trans isomerase FKBP6 [Nothobranchius
(341) KPP68264 hypothetical protein Z043_113075 [Scleropages formosus].
(342) XP_020488061 inactive peptidyl-prolyl cis-trans isomerase FKBP6 [Labrus bergylta].
(343) XP_015332442 PREDICTED: inactive peptidyl-prolyl cis-trans isomerase FKBP6, partial [Marmo
(344) XP_013764143 PREDICTED: inactive peptidyl-prolyl cis-trans isomerase FKBP6 isoform X1 [Pun
(345) XP_005449737 PREDICTED: inactive peptidyl-prolyl cis-trans isomerase FKBP6 isoform X1 [Ore
(346) KFO61461 Peptidyl-prolyl cis-trans isomerase FKBP6, partial [Corvus brachyrhynchos].
(347) XP_008298046 PREDICTED: inactive peptidyl-prolyl cis-trans isomerase FKBP6 [Stegastes part
(348) KFP07119 Peptidyl-prolyl cis-trans isomerase FKBP6, partial [Calypte anna].
(349) XP_007561093 PREDICTED: inactive peptidyl-prolyl cis-trans isomerase FKBP6 isoform X2 [Poe
(350) PKU35662 peptidyl-prolyl cis-trans isomerase hypothetical protein [Limosa lapponica ba
(351) XP_010793476 PREDICTED: inactive peptidyl-prolyl cis-trans isomerase FKBP6 [Notothenia cor
(352) AWP19851 putative inactive peptidyl-prolyl cis-trans isomerase FKBP6 isoform 3 [Scopht
(353) KFW82601 Peptidyl-prolyl cis-trans isomerase FKBP6, partial [Manacus vitellinus].
(354) XP_008396659 PREDICTED: inactive peptidyl-prolyl cis-trans isomerase FKBP6 isoform X3 [Poe
(355) XP_010739761 PREDICTED: inactive peptidyl-prolyl cis-trans isomerase FKBP6 [Larimichthys c
(356) XP_022046121 inactive peptidyl-prolyl cis-trans isomerase FKBP6 [Acanthochromis polyacanth
(357) XP_019715044 PREDICTED: inactive peptidyl-prolyl cis-trans isomerase FKBP6 [Hippocampus co
(358) XP_018875413 PREDICTED: uncharacterized protein LOC101136274 isoform X1 [Gorilla gorilla g
(359) XP_014327714 inactive peptidyl-prolyl cis-trans isomerase FKBP6 isoform X1 [Xiphophorus ma
(360) XP_009081377 PREDICTED: inactive peptidyl-prolyl cis-trans isomerase FKBP6, partial [Acant
(361) AWP19849 putative inactive peptidyl-prolyl cis-trans isomerase FKBP6 [Scophthalmus max
(362) XP_023134576 inactive peptidyl-prolyl cis-trans isomerase FKBP6 [Amphiprion ocellaris].
(363) XP_012734526 inactive peptidyl-prolyl cis-trans isomerase FKBP6 [Fundulus heteroclitus].
(364) XP_014403985 PREDICTED: inactive peptidyl-prolyl cis-trans isomerase FKBP6-like [Myotis br
(365) XP_017553083 PREDICTED: inactive peptidyl-prolyl cis-trans isomerase FKBP6 isoform X2 [Pyg
(366) XP_023869472 inactive peptidyl-prolyl cis-trans isomerase FKBP6-like isoform X2 [Salvelinu
(367) XP_013992804 PREDICTED: inactive peptidyl-prolyl cis-trans isomerase FKBP6-like isoform X3
(368) XP_020774925 inactive peptidyl-prolyl cis-trans isomerase FKBP6 [Boleophthalmus pectiniros
(369) XP_024287709 inactive peptidyl-prolyl cis-trans isomerase FKBP6 isoform X3 [Oncorhynchus t
(370) XP_023869475 inactive peptidyl-prolyl cis-trans isomerase FKBP6-like isoform X4 [Salvelinu
(371) XP_013992805 PREDICTED: inactive peptidyl-prolyl cis-trans isomerase FKBP6-like isoform X4
(372) XP_023869476 inactive peptidyl-prolyl cis-trans isomerase FKBP6-like isoform X5 [Salvelinu
(373) XP_024287708 inactive peptidyl-prolyl cis-trans isomerase FKBP6 isoform X2 [Oncorhynchus t
(374) XP_023869474 inactive peptidyl-prolyl cis-trans isomerase FKBP6-like isoform X3 [Salvelinu
(375) XP_011490382 inactive peptidyl-prolyl cis-trans isomerase FKBP6 isoform X3 [Oryzias latipe
(376) XP_020570387 inactive peptidyl-prolyl cis-trans isomerase FKBP6 isoform X2 [Oryzias latipe
(377) XP_008396657 PREDICTED: inactive peptidyl-prolyl cis-trans isomerase FKBP6 isoform X1 [Poe
(378) XP_014851597 PREDICTED: inactive peptidyl-prolyl cis-trans isomerase FKBP6 isoform X1 [Poe
(379) XP_020570386 inactive peptidyl-prolyl cis-trans isomerase FKBP6 isoform X1 [Oryzias latipe
(380) XP_008396658 PREDICTED: inactive peptidyl-prolyl cis-trans isomerase FKBP6 isoform X2 [Poe
(381) XP_018774793 PREDICTED: inactive peptidyl-prolyl cis-trans isomerase FKBP6 isoform X2 [Ser
(382) XP_023794778 inactive peptidyl-prolyl cis-trans isomerase FKBP6 isoform X3 [Cyanistes caer
(383) XP_024147377 inactive peptidyl-prolyl cis-trans isomerase FKBP6 isoform X2 [Oryzias melast
(384) XP_024147375 inactive peptidyl-prolyl cis-trans isomerase FKBP6 isoform X1 [Oryzias melast
(385) XP_024147378 inactive peptidyl-prolyl cis-trans isomerase FKBP6 isoform X3 [Oryzias melast
(386) XP_014851599 PREDICTED: inactive peptidyl-prolyl cis-trans isomerase FKBP6 isoform X3 [Poe
(387) XP_008396660 PREDICTED: inactive peptidyl-prolyl cis-trans isomerase FKBP6 isoform X4 [Poe
(388) XP_017593619 PREDICTED: inactive peptidyl-prolyl cis-trans isomerase FKBP6 isoform X2 [Cor
(389) XP_008396661 PREDICTED: inactive peptidyl-prolyl cis-trans isomerase FKBP6 isoform X5 [Poe
(390) XP_014851600 PREDICTED: inactive peptidyl-prolyl cis-trans isomerase FKBP6 isoform X4 [Poe

(391) XP_019619874 PREDICTED: inactive peptidyl-prolyl cis-trans isomerase FKBP6-like [Branchios
(392) XP_016801711 inactive peptidyl-prolyl cis-trans isomerase FKBP6-like isoform X3 [Pan trogl
(393) XP_023710706 inactive peptidyl-prolyl cis-trans isomerase FKBP6 [Cryptotermes secundus].
(394) XP_020627099 inactive peptidyl-prolyl cis-trans isomerase FKBP6-like [Orbicella faveolata]
(395) XP_006793207 PREDICTED: peptidyl-prolyl cis-trans isomerase FKBP6-like [Neolamprologus bri
(396) XP_011402831 PREDICTED: inactive peptidyl-prolyl cis-trans isomerase FKBP6-like [Amphimedo
(397) XP_022801008 inactive peptidyl-prolyl cis-trans isomerase FKBP6-like [Stylophora pistillat
(398) XP_013786360 inactive peptidyl-prolyl cis-trans isomerase FKBP6-like isoform X2 [Limulus p
(399) XP_022254671 inactive peptidyl-prolyl cis-trans isomerase FKBP6-like isoform X1 [Limulus p
(400) ELT98785 hypothetical protein CAPTEDRAFT_220934 [Capitella teleta].
(401) XP_021353947 inactive peptidyl-prolyl cis-trans isomerase FKBP6-like [Mizuhopecten yessoen
(402) XP_022108900 inactive peptidyl-prolyl cis-trans isomerase FKBP6-like isoform X2 [Acanthast
(403) XP_022108899 inactive peptidyl-prolyl cis-trans isomerase FKBP6-like isoform X1 [Acanthast
(404) XP_002167164 PREDICTED: inactive peptidyl-prolyl cis-trans isomerase FKBP6-like [Hydra vul
(405) KOF62423 hypothetical protein OCBIM_22023537mg, partial [Octopus bimaculoides].
(406) XP_014790271 PREDICTED: inactive peptidyl-prolyl cis-trans isomerase FKBP6-like [Octopus b
(407) XP_001636239 predicted protein [Nematostella vectensis].
(408) XP_023237057 inactive peptidyl-prolyl cis-trans isomerase FKBP6-like [Centruroides sculptu
(409) XP_002128893 PREDICTED: inactive peptidyl-prolyl cis-trans isomerase FKBP6 isoform X1 [Cio
(410) XP_009051935 hypothetical protein LOTGIDRAFT_114969 [Lottia gigantea].
(411) XP_012221039 PREDICTED: inactive peptidyl-prolyl cis-trans isomerase FKBP6 [Linepithema hu
(412) XP_012264923 inactive peptidyl-prolyl cis-trans isomerase FKBP6 [Athalia rosae].
(413) XP_009857551 PREDICTED: inactive peptidyl-prolyl cis-trans isomerase FKBP6 isoform X2 [Cio
(414) XP_015515854 PREDICTED: inactive peptidyl-prolyl cis-trans isomerase FKBP6 [Neodiprion lec
(415) XP_011415211 PREDICTED: inactive peptidyl-prolyl cis-trans isomerase FKBP6 [Crassostrea gi
(416) XP_020909274 inactive peptidyl-prolyl cis-trans isomerase FKBP6-like [Exaiptasia pallida].
(417) EKC39444 hypothetical protein CGI_10020047 [Crassostrea gigas].
(418) XP_013405452 inactive peptidyl-prolyl cis-trans isomerase FKBP6-like isoform X2 [Lingula a
(419) XP_011148430 inactive peptidyl-prolyl cis-trans isomerase FKBP6 [Harpegnathos saltator].
(420) XP_013405451 inactive peptidyl-prolyl cis-trans isomerase FKBP6-like isoform X1 [Lingula a
(421) PVD31214 hypothetical protein C0Q70_06626 [Pomacea canaliculata].
(422) PIK39047 putative inactive peptidyl-prolyl cis-trans isomerase FKBP6 [Apostichopus jap
(423) XP_022336187 inactive peptidyl-prolyl cis-trans isomerase FKBP6-like [Crassostrea virginic
(424) XP_022337811 inactive peptidyl-prolyl cis-trans isomerase FKBP6-like [Crassostrea virginic
(425) XP_014787871 PREDICTED: inactive peptidyl-prolyl cis-trans isomerase FKBP6-like, partial [
(426) EFN78601 FK506-binding protein 6 [Harpegnathos saltator].
(427) XP_014475449 PREDICTED: inactive peptidyl-prolyl cis-trans isomerase FKBP6 [Dinoponera qua
(428) XP_014279621 inactive peptidyl-prolyl cis-trans isomerase FKBP6-like [Halyomorpha halys].
(429) XP_022193504 inactive peptidyl-prolyl cis-trans isomerase FKBP6 [Nilaparvata lugens].
(430) XP_014204130 inactive peptidyl-prolyl cis-trans isomerase FKBP6 [Copidosoma floridanum].
(431) KOX75315 Peptidyl-prolyl cis-trans isomerase FKBP6 [Melipona quadrifasciata].
(432) XP_014234164 inactive peptidyl-prolyl cis-trans isomerase FKBP6 [Trichogramma pretiosum].
(433) KII63942 Inactive peptidyl-prolyl cis-trans isomerase FKBP6 [Thelohanellus kitauei].
(434) XP_024936423 inactive peptidyl-prolyl cis-trans isomerase FKBP6 isoform X2 [Cephus cinctus
(435) XP_015586170 inactive peptidyl-prolyl cis-trans isomerase FKBP6 isoform X1 [Cephus cinctus
(436) XP_003486736 inactive peptidyl-prolyl cis-trans isomerase FKBP6 [Bombus impatiens].
(437) XP_019882220 PREDICTED: inactive peptidyl-prolyl cis-trans isomerase FKBP6 isoform X1 [Cam
(438) XP_003402471 inactive peptidyl-prolyl cis-trans isomerase FKBP6 [Bombus terrestris].
(439) XP_012134979 PREDICTED: inactive peptidyl-prolyl cis-trans isomerase FKBP6 isoform X2 [Meg
(440) XP_017756606 PREDICTED: inactive peptidyl-prolyl cis-trans isomerase FKBP6 [Eufriesea mexi
(441) XP_012134978 PREDICTED: inactive peptidyl-prolyl cis-trans isomerase FKBP6 isoform X1 [Meg
(442) XP_003700792 PREDICTED: inactive peptidyl-prolyl cis-trans isomerase FKBP6 isoform X3 [Meg
(443) XP_023290805 inactive peptidyl-prolyl cis-trans isomerase FKBP6 isoform X2 [Orussus abieti
(444) XP_012270499 inactive peptidyl-prolyl cis-trans isomerase FKBP6 isoform X1 [Orussus abieti
(445) XP_018309319 PREDICTED: inactive peptidyl-prolyl cis-trans isomerase FKBP6 isoform X1 [Tra
(446) XP_012062619 PREDICTED: inactive peptidyl-prolyl cis-trans isomerase FKBP6 [Atta cephalote

(447) XP_012527508 PREDICTED: inactive peptidyl-prolyl cis-trans isomerase FKBP6-like [Monomoriu
(448) XP_005089179 PREDICTED: inactive peptidyl-prolyl cis-trans isomerase FKBP6-like [Aplysia c
(449) XP_003424555 PREDICTED: inactive peptidyl-prolyl cis-trans isomerase FKBP6 [Nasonia vitrip
(450) XP_018055399 PREDICTED: inactive peptidyl-prolyl cis-trans isomerase FKBP6-like [Atta colo
(451) XP_017877748 PREDICTED: inactive peptidyl-prolyl cis-trans isomerase FKBP6 [Ceratina calca
(452) XP_018399012 PREDICTED: inactive peptidyl-prolyl cis-trans isomerase FKBP6 [Cyphomyrmex co
(453) KYM99352 Peptidyl-prolyl cis-trans isomerase FKBP6 [Cyphomyrmex costatus].
(454) XP_018309322 PREDICTED: inactive peptidyl-prolyl cis-trans isomerase FKBP6 isoform X2 [Tra
(455) XP_011647883 inactive peptidyl-prolyl cis-trans isomerase FKBP6 [Pogonomyrmex barbatus].
(456) XP_014247948 inactive peptidyl-prolyl cis-trans isomerase FKBP6-like isoform X2 [Cimex lec
(457) XP_011064230 PREDICTED: inactive peptidyl-prolyl cis-trans isomerase FKBP6-like [Acromyrme
(458) XP_014613253 PREDICTED: inactive peptidyl-prolyl cis-trans isomerase FKBP6 [Polistes canad
(459) XP_002430548 FK506-binding protein, putative [Pediculus humanus corporis].
(460) XP_014247946 inactive peptidyl-prolyl cis-trans isomerase FKBP6-like isoform X1 [Cimex lec
(461) XP_014270488 inactive peptidyl-prolyl cis-trans isomerase FKBP6 [Halyomorpha halys].
(462) ORY30517 hypothetical protein BCR33DRAFT_724326 [Rhizoclosmatium globosum].
(463) XP_011330216 PREDICTED: inactive peptidyl-prolyl cis-trans isomerase FKBP6 [Ooceraea biroi
(464) EGI60945 FK506-binding protein 6, partial [Acromyrmex echinatior].
(465) XP_001201219 PREDICTED: inactive peptidyl-prolyl cis-trans isomerase FKBP6 [Strongylocentr
(466) XP_011061927 PREDICTED: inactive peptidyl-prolyl cis-trans isomerase FKBP6-like [Acromyrme
(467) XP_018356457 PREDICTED: inactive peptidyl-prolyl cis-trans isomerase FKBP6-like isoform X2
(468) XP_011173578 PREDICTED: inactive peptidyl-prolyl cis-trans isomerase FKBP6 [Solenopsis inv
(469) XP_018356456 PREDICTED: inactive peptidyl-prolyl cis-trans isomerase FKBP6-like isoform X1
(470) XP_016917008 PREDICTED: inactive peptidyl-prolyl cis-trans isomerase FKBP6 isoform X2 [Api
(471) XP_018374414 PREDICTED: inactive peptidyl-prolyl cis-trans isomerase FKBP6 [Trachymyrmex c
(472) XP_016917007 PREDICTED: inactive peptidyl-prolyl cis-trans isomerase FKBP6 isoform X1 [Api
(473) XP_019882221 PREDICTED: inactive peptidyl-prolyl cis-trans isomerase shutdown isoform X2 [
(474) XP_011191641 PREDICTED: inactive peptidyl-prolyl cis-trans isomerase shutdown [Zeugodacus
(475) PFH33404 peptidyl-prolyl cis-trans isomerase [Besnoitia besnoiti].
(476) XP_019876143 PREDICTED: inactive peptidyl-prolyl cis-trans isomerase shutdown [Aethina tum
(477) XP_014299498 PREDICTED: inactive peptidyl-prolyl cis-trans isomerase FKBP6-like [Microplit
(478) KOC63519 Peptidyl-prolyl cis-trans isomerase FKBP6 [Habropoda laboriosa].
(479) XP_015126020 PREDICTED: inactive peptidyl-prolyl cis-trans isomerase FKBP6 [Diachasma allo
(480) XP_005186588 PREDICTED: inactive peptidyl-prolyl cis-trans isomerase shutdown [Musca domes
(481) XP_011879049 PREDICTED: inactive peptidyl-prolyl cis-trans isomerase FKBP6 isoform X2 [Vol
(482) XP_013109471 PREDICTED: inactive peptidyl-prolyl cis-trans isomerase shutdown [Stomoxys ca
(483) XP_011879048 PREDICTED: inactive peptidyl-prolyl cis-trans isomerase FKBP6 isoform X1 [Vol
(484) XP_018333359 PREDICTED: inactive peptidyl-prolyl cis-trans isomerase FKBP6 [Agrilus planip
(485) EFZ09302 hypothetical protein SINV_13600, partial [Solenopsis invicta].
(486) XP_016927793 PREDICTED: inactive peptidyl-prolyl cis-trans isomerase shutdown [Drosophila
(487) XP_006611831 PREDICTED: peptidyl-prolyl cis-trans isomerase FKBP6-like [Apis dorsata].
(488) XP_014245088 inactive peptidyl-prolyl cis-trans isomerase FKBP6-like [Cimex lectularius].
(489) XP_018790058 PREDICTED: inactive peptidyl-prolyl cis-trans isomerase shutdown-like [Bactro
(490) XP_020893545 inactive peptidyl-prolyl cis-trans isomerase FKBP6-like [Exaiptasia pallida].
(491) XP_002092689 uncharacterized protein Dyak_GE11527 [Drosophila yakuba].
(492) XP_020293583 inactive peptidyl-prolyl cis-trans isomerase FKBP6-like [Pseudomyrmex gracili
(493) XP_016952968 PREDICTED: inactive peptidyl-prolyl cis-trans isomerase shutdown [Drosophila
(494) XP_003689972 PREDICTED: inactive peptidyl-prolyl cis-trans isomerase FKBP6 isoform X1 [Api
(495) XP_015185328 PREDICTED: inactive peptidyl-prolyl cis-trans isomerase FKBP6 [Polistes domin
(496) XP_024867102 inactive peptidyl-prolyl cis-trans isomerase FKBP6 [Temnothorax curvispinosus
(497) XP_011130643 putative peptidyl-prolyl cis-trans isomerase [Gregarina niphandrodes].
(498) XP_011206843 PREDICTED: inactive peptidyl-prolyl cis-trans isomerase shutdown [Bactrocera
(499) XP_008560701 PREDICTED: inactive peptidyl-prolyl cis-trans isomerase FKBP6 isoform X2 [Mic
(500) XP_011695993 PREDICTED: inactive peptidyl-prolyl cis-trans isomerase FKBP6-like [Wasmannia
(501) PIK55877 putative peptidyl-prolyl cis-trans isomerase FKBP6-like [Apostichopus japonic
(502) XP_022218357 inactive peptidyl-prolyl cis-trans isomerase shutdown [Drosophila obscura].

(503) XP_017120033 PREDICTED: inactive peptidyl-prolyl cis-trans isomerase shutdown [Drosophila
(504) XP_022165811 inactive peptidyl-prolyl cis-trans isomerase FKBP6-like [Myzus persicae].
(505) XP_017070534 PREDICTED: inactive peptidyl-prolyl cis-trans isomerase shutdown [Drosophila
(506) XP_001942690 PREDICTED: inactive peptidyl-prolyl cis-trans isomerase FKBP6 [Acyrthosiphon
(507) XP_003883131 Peptidylprolyl isomerase D (Cyclophilin D),related [Neospora caninum Liverpoo
(508) XP_014091483 PREDICTED: inactive peptidyl-prolyl cis-trans isomerase shutdown [Bactrocera
(509) XP_015376375 PREDICTED: inactive peptidyl-prolyl cis-trans isomerase FKBP6-like [Diuraphis
(510) KOF66856 hypothetical protein OCBIM_22011108mg, partial [Octopus bimaculoides].
(511) XP_015431627 PREDICTED: inactive peptidyl-prolyl cis-trans isomerase shutdown [Dufourea no
(512) ORX48915 FKBP-like protein [Piromyces finnis].
(513) XP_001958659 uncharacterized protein Dana_GF12456 [Drosophila ananassae].
(514) XP_013077159 PREDICTED: inactive peptidyl-prolyl cis-trans isomerase FKBP6-like isoform X2
(515) XP_013077158 PREDICTED: inactive peptidyl-prolyl cis-trans isomerase FKBP6-like isoform X1
(516) XP_016974015 PREDICTED: inactive peptidyl-prolyl cis-trans isomerase shutdown [Drosophila
(517) KFG33606 peptidyl-prolyl cis-trans isomerase, partial [Toxoplasma gondii FOU].
(518) XP_017045419 PREDICTED: inactive peptidyl-prolyl cis-trans isomerase shutdown [Drosophila
(519) PUA87907 peptidyl-prolyl cis-trans isomerase, partial [Toxoplasma gondii TgCATBr9].
(520) XP_017001929 PREDICTED: inactive peptidyl-prolyl cis-trans isomerase shutdown [Drosophila
(521) XP_009012598 hypothetical protein HELRODRAFT_168497 [Helobdella robusta].
(522) XP_008888049 peptidyl-prolyl cis-trans isomerase [Hammondia hammondi].
(523) XP_023297605 inactive peptidyl-prolyl cis-trans isomerase shutdown-like [Lucilia cuprina].
(524) XP_002594856 hypothetical protein BRAFLDRAFT_86023 [Branchiostoma floridae].
(525) XP_018896035 PREDICTED: inactive peptidyl-prolyl cis-trans isomerase FKBP6-like [Bemisia t
(526) XP_004517316 inactive peptidyl-prolyl cis-trans isomerase shutdown [Ceratitis capitata].
(527) XP_017148191 PREDICTED: inactive peptidyl-prolyl cis-trans isomerase shutdown-like [Drosop
(528) XP_002412109 FKBP-type peptidyl-prolyl cis-trans isomerase, putative, partial [Ixodes scap
(529) XP_014507068 70 kDa peptidyl-prolyl isomerase [Vigna radiata var. radiata].
(530) XP_011298074 PREDICTED: inactive peptidyl-prolyl cis-trans isomerase shutdown [Fopius aris
(531) ESS30489 peptidyl-prolyl cis-trans isomerase [Toxoplasma gondii VEG].
(532) EPR59296 peptidyl-prolyl cis-trans isomerase [Toxoplasma gondii GT1].
(533) OAJ38225 hypothetical protein BDEG_22175 [Batrachochytrium dendrobatidis JEL423].
(534) XP_018637740 peptidyl-prolyl cis-trans isomerase [Toxoplasma gondii ME49].
(535) KYK63140 peptidyl-prolyl cis-trans isomerase [Toxoplasma gondii TgCatPRC2].
(536) CEL73032 TPA: peptidyl-prolyl isomerase, putative [Toxoplasma gondii VEG].
(537) AAX51680 dual-family immunophilin 57 kDa [Toxoplasma gondii].
(538) AFJ24780 peptidyl prolyl cis trans isomerase FKBP-2, partial [Schmidtea mediterranea].
(539) NP_611837 shutdown [Drosophila melanogaster].
(540) XP_012342118 PREDICTED: inactive peptidyl-prolyl cis-trans isomerase FKBP6 isoform X2 [Api
(541) XP_002082854 uncharacterized protein Dsimw501_GD25009, isoform A [Drosophila simulans].
(542) XP_002040150 GM15507 [Drosophila sechellia].
(543) XP_021954336 inactive peptidyl-prolyl cis-trans isomerase FKBP6-like [Folsomia candida].
(544) XP_001357527 uncharacterized protein Dpse_GA18393 [Drosophila pseudoobscura pseudoobscura]
(545) XP_002015409 GL11067 [Drosophila persimilis].
(546) XP_017469679 PREDICTED: inactive peptidyl-prolyl cis-trans isomerase shutdown-like [Rhagol
(547) XP_020810045 inactive peptidyl-prolyl cis-trans isomerase shutdown [Drosophila serrata].
