# Supplementary material for: Compression of Large Sets of Sequence Data Reveals Fine Diversification of Functional Profiles in Multigene Families of Proteins: A Study for Peptidyl-Prolyl cis/trans Isomerases (PPIase)
Source: Biomolecules. 2019 Feb 11;9(2):59. doi: 10.3390/biom9020059 (PMC6406343; doi:10.3390/biom9020059)
Supplement: Supplementary file 1 [file biomolecules-09-00059-s001.zip › Figure S6-FKBD2 from FKBP51.aln.pdf]

CLUSTAL 2.0.9 multiple sequence alignment of FKBP2 from FKBP51s

```
NP_004108B          DLFE--DGGIIRRTKRKGEGYSNPNEGATVEIHLEGRCGGRMFDCRDVAFTVGEGEDHDI
XP_518427B          DLFE--DGGIIRRTKRKGEGYSNPNEGATVEIHLEGRCGGRMFDCRDVAFTVGEGEDHDI
XP_004043925B       DLFE--DGGIIRRTKRKGEGYSNPNEGATVEIHLEGRCGGRMFDCRDVAFTVGEGEDHDI
XP_003278903B       DLFE--DGGIIRRTKRKGEGYSNPNEGATVEIHLEGHCGGRMFDCRDVAFTVGEGEDHDI
NP_001124689B       DLFE--DGGIIRRTKRKGEGYSNPNEGATVEIHLEGRCGGRMFDCRDVAFTVGEGEDHVI
XP_010387961B       DLFE--DGGIIRRTKRKGEGYSNPNEGATVEIHLEGRCGERMFDCRDVAFTVGEGEDHDI
XP_023068393B       DLFE--DGGIIRRTKRKGEGYSNPNEGATVEIHLEGRCGERMFDCRDVAFTVGEGEDHDI
AAA86245B           DLFE--DGGIIRRTKRKGEGYSNPNEGATVEIHLEGRCGGRMFDCRDVAFTVGEGEDHDI
XP_003897547B       DLFE--DGGIIRRTKRKGEGYSNPNEGATVEIHLEGRCGERMFDCRDVAFTVGEGEDHDI
XP_011841956B       DLFE--DGGIIRRTKRKGEGYSNPNEGATVEIHLEGRCGERMFDCRDVAFTVGEGEDHDI
XP_011800768B       DLFE--DGGIIRRTKRKGEGYSNPNEGATVEIHLEGRCGERMFDCRDVAFTVGEGEDHDI
XP_005553272B       DLFE--DGGIIRRTKRKGEGYSNPNEGATVEIHLEGRCGERMFDCRDVAFTVGEGEDHDI
XP_007971022B       DLLE--DGGIIRRTKRKGEGYSNPNEGATVEIHLEGRCGERMFDCRDVAFTVGEGEDHDI
Q95L05B             DLLE--DGGIIRRTKRKGEGYSNPNEGATVEIHLEGRCGERMFDCRDVAFTVGEGEDHDI
XP_011930726B       DLFE--DGGIIRRTKRKGEGYSNPNEGATVEIHLEGRCGERMFDCRDVAFTVGEGEDHDI
XP_022437414B       DLFE--DGGIIRRIKRKGEGYSNPNEGATVEIHLEGRCGGRMFDCRDVGFIVGEGEDHDI
XP_004267730B       DLFE--DGGIIRRIKRKGEGYSNPNEGATVEIHLEGRCGGRMFDCRDVGFIVGEGEDHDI
XP_007467316B       DLFE--DGGIIRRIKRKGEGYSNPNEGATVEIHLEGRCGGRMFDCRDVGFIVGEGEDHDI
XP_006202142B       DLFE--DGGIIRRIKRKGEGYSNPNEGATVEIHLEGRCGGRMFDCRDVGFIVGEGEDHDI
XP_020024507B       DLFE--DAGIIRRIKRKGEGYSNPNEGATVEIHLEGCCGGRMFDCRDVVFTVGEGEDHDI
XP_013833281B       DLFE--DGGIIRRIKRKGEGYSNPNEGATVEIHLEGRCGGRVFDCRDVGFIVGEGEDHDI
XP_020024506B       DLFE--DAGIIRRIKRKGEGYSNPNEGATVEIHLEGCCGGRMFDCRDVVFTVGEGEDHDI
XP_020953710B       DLFE--DGGIIRRIKRKGEGYSNPNEGATVEIHLEGRCGGRVFDCRDVGFIVGEGEDHDI
NP_001302540B       DLFE--DGGIIRRIKRKGEGYSNPNEGATVEIHLEGRCGGRVFDCRDVGFIVGEGEDHDI
XP_020142566B       DLFE--DGGIIRRIKRKGEGYSNPNEGATVEIHLEGRCGERMFDCRNVVFTVGEGEDHDI
XP_006180531B       DLFE--DGGIIRRIKRKGEGYSNPNEGATVEIHLEGRCGGRMFDCRDVGFIVGEGEDHDI
XP_015343090B       DLFE--DAGIIRRIKRKGEGYSNPNEGATVEIHLEGCCGGRMFDCRDVVFTVGEGEDHDI
XP_005318831B       DLFE--DAGIIRRIKRKGEGYSNPNEGATVEIHLEGCCGGRMFDCRDVVFTVGEGEDHDI
XP_010947329B       DLFE--DGGIIRRIKRKGEGYSNPNEGATVEIHLEGRCGGRMFDCRDVGFIVGEGEDHDI
XP_023977801B       DLFE--DGGIIRRIKRKGEGYSNPNEGATVEIHLEGCCGGRMFDCRDVGFIVGEGEDHDI
XP_020742361B       DLFE--DGGIIRRIKRKGEGYSNPNEGATVEIHLEGRCGGRMFDCRDVGFIVGEGEDHDI
XP_007198040B       DLFE--DGGIIRRIKRKGEGYSNPNEGATVEIHLEGRCGGRMFDCRDVGFIVGEGEDHDI
XP_017894445B       DLFE--DGGIIRRIKRKGEGYSNPNEGATVEIHLEGRCGGRMFDCRDVGFIVGEGEDHDI
XP_002714685B       DLFE--DAGIIRRIKRKGEGYSNPNEGATVEIHLEGCCGGRMFDCRDVVFVVGEGEDHDI
XP_012502082B       DLFE--DGGIIRRIKQKGEGYSNPNEGATVEIHLEGHCGERMFDCRNVVFTVGEGEDHDI
XP_004379454B       DLFE--DGGIIRRIKQKGEGYSNPNEGATVEIHLIGRCGGRLFDCRDVIFIVGEGEDHDI
XP_023368573B       DLLE--DGGIIRRIKRKGEGYSNPNEGATVEVHLEGHCGGRMFDRRNVVFTVGEGEDHDI
XP_014958158B       DLFE--DGGIIRRIKRKGEGYSNPNEGATVEIHLEGRCGGRMFDCRDVGFIVGEGEDHDI
NP_001295455B       DLLE--DGGIIRRTKRRGEGYSNPNEGARVQIHLEGRCGGRVFDCRDVAFTVGEGEDHDI
XP_003789178B       DLLE--DGGIIRRIKRKGEGYSNPNEGATVEVHLEGHCGGRMFDRRNVVFTVGEGEDHDI
XP_006050442B       DLFE--DGGIIRRIKRKGEGYSNPNEGATVEIHLEGRCGGRTFDCRDVGFIVGEGEDHDI
XP_005979785B       DLFE--DGGIIRRIKRKGEGYSNPNEGATVEIHLEGRCGGRTFDCRDVGFIVGEGEDHDI
XP_004770745B       DLFE--DGGIIRRIKRKGEGYSNPNEGATVEIHLEGRCDGRMFDCRDVVFIVGEGEDHDI
XP_011967348B       DLFE--DGGIIRRIKRKGEGYSNPNEGATVEIHLEGRCGGRMFDCRDVGFIVGEGEDHDI
NP_001179791B       DLFE--DGGIIRRIKRKGEGYSNPNEGATVEIHLEGRCGGRTFDCRDVGFIVGEGEDHDI
XP_017826524B       DLLE--DGGIIRRTKRRGEGYSNPNEGARVQIHLEGRCGGRVFDCRDVAFTVGEGEDHDI
XP_004424279B       DLFE--DGGIIRRIKQKGEGYSNPNEGATVEIHLEGRCAGKIFDCRDVVFIVGEGEDHDI
NP_001266943B       DLLE--DGGIIRRTKRRGEGYSNPNEGARVQIHLEGRCGGRVFDCRDVAFTVGEGEDHDI
XP_022366563B       DLFE--DGGIIRRIKRKGEGYSNPNEGATVEIHLEGRYDGRMFDCRDVVFIVGEGEDHDI
XP_017378122B       DLLE--DGGIIRRTKRRGEGYSNPNEGARVQIHLEGRCGGRVFDCRDVAFTVGEGEDHDI
XP_023480581B       DLFE--DGGIIRRIKQKGEGYSNPNEGATVEIHLEGRCGERTFDCRDVVFIVGEGEDHDI
XP_006144244B       DLFE--DGGIIRRIKRKGEGYSNPNEGATVEIHLEGRCDGRMFDCRDVVFVVGEGEDHDI
XP_006882111B       DLFE--DGGIIRRIKQKGEGYSNPNEGATVEIHLVGRCGGQVFDCRDVIFIVGEGEDHDI
XP_008528289B       DLFE--DGGIIRRIKQKGEGYSNPNEGATVEIHLEGRCGERTFDCRDVVFIVGEGEDHDI
XP_538880B          DLFE--DGGIIRRIKQKGEGYSNPNEGATVEIHLEGRCDGRMFDCRDVVFIVGEGEDHDI
XP_012885079B       DLFE--DAGIIRRIKRKGEGYSNPNEGATVEIHLEGRCGGRMFDCRDVVFTVGEGEDHDI
XP_012420443B       DLFE--DGGIIRRIKQKGEGYSNPNEGATVEIHLEGHYDGRMFDCRDVVFIVGEGEDHDI
Q9XSI2B             DLLE--DGGIIRRTKRRGEGYSNPNEGARVQIHLEGRCGGRVFDCRDVAFTVGEGEDHDI
XP_006739186B       DLFE--DGGIIRRIKQKGEGYSNPNEGATVEIHLEGRYDGRMFDCRDVVFIVGEGEDHDI
XP_003404000B       DLFE--DGGIIRRIKQKGEGYSNPNEGATVEIHLIGHCGGRLFDCRDVTFIVGEGEDHDI
XP_021540340B       DLFE--DGGIIRRIKQKGEGYSNPNEGATVEIHLEGRYDGRMFDCRDVVFIVGEGEDHDI
XP_002914343B       DLFE--DGGIIRRIKQKGEGYSNPNEGATVEIHLEGRYGGRMFDCRDVVFIVGEGEDHDI
XP_006860458B       DLFD--DGGIIRRIKQKGEGYSNPNEGATVEIHLVGHCGGQLFDCRDVVFIVGEGEDHDI
XP_008691621B       DLFE--DGGIIRRIKQKGEGYSNPNEGATVEIHLEGRYDGRMFDCRDVVFIVGEGEDHDI
XP_004624255B       DLFE--DGGIIRRIKRKGEGYSNPNEGATVDIHLEGRYGGRMFDCRDVVFIVGEGEDHDI
XP_006907392B       DLFE--DGGIIRRIKRKGEGYSNPNEGATVEIHLEGHCGGKMFDCRDVIFIVGEGEDHDI
XP_006759086B       DLFE--DGGIIRRIKRKGEGYSNPNEGATVEIHLEGRCGERMFDCRDVVFIVGEGEDHDI
XP_007534316B       DLFE--DGGIIRRIKQKGEGYSNPNEGATVEIHLEGCCGGRMFDCRDVVFIVGEGEDHDI
XP_004590364B       DLFE--DAGIIRRIKRKGEGYSNPNEGATVEIHLEGCCGGRMFDCRDVVFVVGEGEDHDI
XP_005389619B       DLFE--DGGIIRRIKRKGEGYSNPNEGARVDIHLEGRYGGRTFDCRDVIFIVGEGEDHDI
```

XP_013372357B	DLFE--DGGIIRRIKRKGEGYSNPNEGARVDIHLEGRYGGRTFDCRDVIFIVGEGEDHDI
XP_004846760B	DLFE--DSGIIRRIKRKGEGYSNPNEGATVNIHLEGCCGGRMFDCRDVVFIVGEGEDHDI
XP_006101307B	DLFE--DGGIIRRIKQKGEGYSNPNEGATVEIHLEGRCGERMFDCRDVVFIVGEGEDHDI
XP_011280550B	DLFE--DGGIIRRIKQKGEGYSNPNEGATVEIHLEGRYNGRMFDRRDVVFIVGEGEDHDI
XP_019312428B	DLFE--DGGIIRRIKQKGEGYSNPNEGATVEIHLEGRYNGRMFDCRDVVFIVGEGEDHDI
XP_014925310B	DLFE--DGGIIRRIKQKGEGYSNPNEGATVEIHLEGRYNGRMFDCRDVVFIVGEGEDHDI
XP_016079225B	DLFE--DGGIIRRIKRKGEGYSYPNEGATVEVHLEGRCGDRTFDCRDVVFTVGEGEDHDI
XP_010603642B	DLFE--DAGIIRRIKRKGEGYSNPNEGATVNIHLEGRCGRRLFDCRDVVFVVGEGEDHDI
XP_005881801B	DLFE--DGGIIRRIKQKGEGYSNPNEGATVEIHLEGRCGERMFDCRDVVFIVGEGEDHDI
XP_012577888B	DLFE--DGGIIRRIKRKGEGYSNPNEGATVEIHLEGCYGGKMFDCRDVVFVVGEGEDHDI
XP_007085336B	DLFE--DGGIIRRIKQKGEGYSNPNEGATVEIHLEGRYNGRMFDCRDVVFIVGEGEDHDI
XP_003473945B	DLFE--DGGIVRRIKRKGEGYSNPNEGATVNIHLEGHCGGRMFDCRDVAFIVGEGEDHDI
XP_019594281B	DLFE--DGGIIRRIKQKGEGYSNPNEGATVEVHLEGRCGERMFDCRDVVFIVGEGEDHDI
XP_004710782B	DLFE--DGGIIRRIKQKGEGYSNPNEGATVEIHLVGHCGGQLFDSRDVIFIVGEGEDHDI
XP_015975694B	DLFE--DGGIIRRIRRKGEGYSNPNEGATVEIHLEGHCGGKMFDCRDVIFIVGEGEDHDI
XP_006256284B	DLFE--DSGIIRRIKRKGEGYSNPNEGATVEVHLEGCCAGRVFDCRDVVFVIGEGEDHDI
NP_001012174B	DLFE--DSGIIRRIKRKGEGYSNPNEGATVEVHLEGCCAGRVFDCRDVVFVIGEGEDHDI
XP_019523002B	DLFE--DGGIIRRIKQKGEGYSYPNEGATVEVHLEGRCGERMFDCRDVVFIVGEGEDHDI
XP_003508661B	DLFE--DSGIIRRIKRKGEGYSNPNEGATVEVHLEGCCGGRMFDCRDVVFIVGEGEDHDI
XP_008838320B	DLFE--DSGIIRRIKRKGEGYSNPNEGATVEIHLEGCCGGRLFDCRDVVFIVGEGEDHDI
XP_021792868B	DLFE--DGGIIRRTKRKGEGYSNPNEGATVEIHLEGRCGERMFDCRDVAFTVGEGEDHDI
BAH13948B	DLFE--DGGIIRRTKRKGEGYSNPNEGATVEIHLEGRCGERMFDCRDVAFTVGEGEDHDI
XP_005084951B	DLFE--DSGIIRRIKRKGEGYSNPNEGATVEVHLEGCCGGRMFDCRDVVFVVGEGEDHDI
XP_005360413B	DLFE--DSGIIRRIKRKGEGYSNPNEGATVEVHLEGCCGGRMFDCRDVVFTVGEGEDHDI
XP_024839424B	DLFE--DGGIIRRIKRKGEGYSNPNEGATVEIHLEGRCGGRTFDCRDVGFIVGEGEDHDI
XP_004617764B	DLFE--DGGIIRRIKQKGEGYSNPNEGATVEIHLEGCCGGRRFDCRDVVFIVGEGEDHDI
XP_021485845B	DLFE--DSGIIRRIKRKGEGYSNPNEGATVEVHLEGCCAGRRFDCRDVVFVVGEGEDHNI
XP_006983174B	DLFE--DSGIIRRIKRKGEGYSNPNEGATVEVHLEGCCGGRLFDCRDVVFVVGEGEDHDI
XP_004649837B	DLFD--DSGIIRRIKRKGEGYSNPNEGATVEIHLEGCCGGRTFDCRDVVFVVGEGEDHDI
EFB14016B	DLFE--DGGIIRRIKQKGEGYSNPNEGATVEIHLEGRYGGRMFDCRDVVFIVGEGEDHDI
XP_007483815B	DLFE--DGGIIRRIKQKGEGYSNPNEGATVEIHLEGSCGSRTFDCRDVVFVVGEGEDHDI
XP_014958162B	DLFE--DGGIIRRIKRKGEGYSNPNEGATVEIHLEGRCGGRMFDCRDVGFIVGEGEDHDI
XP_020855250B	DLFE--DGGIIRRIKQKGEGYSNPNEGATVEIHLEGSCGNRTFDCRDVVFVVGEGEDHDI
XP_011967352B	DLFE--DGGIIRRIKRKGEGYSNPNEGATVEIHLEGRCGGRMFDCRDVGFIVGEGEDHDI
XP_021076628B	DLFE--DSGVIRRIKRKGEGYSNPNEGATVKIHLEGCCGGRMFDCRDVVFIVGEGEDHDI
XP_003769030B	DLFE--DGGIIRRIKQKGEGYSNPNEGATVEIHLEGSCGNRTFDCRDVVFVVGEGEDHDI
XP_023589897B	DLFE--DGGIIRRIKQKGEGYSNPNEGATVEIHLIGRCGGRLFDCRDVIFIVGEGEDHDI
XP_004458369B	DMFE--DGGIIRRIKQKGEGYSNPNEGARVEIHLEGHQGGRKFDCRDVVFIVGEGEDHDI
XP_004770751B	DLFE--DGGIIRRIKRKGEGYSNPNEGATVEIHLEGRCDGRMFDCRDVVFIVGEGEDHDI
NP_034350B	DLFE--DSGVIRRIKRKGEGYSNPNEGATVKVHLEGCCGGRTFDCRDVVFVVGEGEDHDI
BAE35044B	DLFE--DSGVIRRIKRKGEGYSNPNEGATVKVHLEGCCGGRTFDCRDVVFVVGEGEDHDI
XP_006882112B	DLFE--DGGIIRRIKQKGEGYSNPNEGATVEIHLVGRCGGQVFDCRDVIFIVGEGEDHDI
XP_021005468B	DLFE--DSGVIRRIKRKGEGYSNPNEGATVKVHLEGCCGGRTFDCRDVVFIVGEGEDHDI
XP_012420445B	DLFE--DGGIIRRIKQKGEGYSNPNEGATVEIHLEGHYDGRMFDCRDVVFIVGEGEDHDI
XP_004590366B	DLFE--DAGIIRRIKRKGEGYSNPNEGATVEIHLEGCCGGRMFDCRDVVFVVGEGEDHDI
XP_024413417B	DLLG--DRGIIRRIKHKGEGYSLPNEGATVDIHLEGHCGERMFDCRDVVFIVGEGEDHDV
XP_007063551B	DLFE--DGGIIRRIKRKGEGYSNPNEGATVEIHLEGFCSGRIFDCRDVTFIVGEGEDHDI
XP_005059495B	DLFE--DGGIIRRIKRKGEGYSNPNEGATVEIHLEGFCGGRRFDCKDVKFVVGEGEDHDI
XP_010135429B	DLFE--DGGIIRRIKRKGEGYSNPNEGATVEIHLEGFCGGTRFDCKDVKFVVGEGEDHDI
XP_024066292B	DLFE--DGGIVRRIKRKGEGYSNPNEGATVEIHLEGFYGGRIFDCRDVTFVVGEGEDHDI
XP_013802188B	DLFE--DGGIIRRIKRKGEGYSNPNEGATVEIHLEGFCGGTRFDCKDVKFVVGEGEDHDI
XP_014376143B	DLFE--DGGIIRRIKKKGEGYSNPNEGATVEIHLEGFYGDRKFDSRDVKFVVGEGEDHDI
XP_005311709B	DLFE--DGGIIRRIKRKGEGYSNPNEGATVEIHLEGFYGGRIFDCRDVTFVVGEGEDHDI
XP_019401755B	DLFE--DGGIIRRIKKKGEGYSNPNEGATVEIHLEGFYGDRKFDSRDVKFVVGEGEDHDI
XP_019369315B	DLFE--DGGIIRRIKKKGEGYSNPNEGATVEIHLEGFYGDRKFDSRDVKFVVGEGEDHDI
XP_014728245B	DLFE--DGGIIRRIKRKGEGYSNPNEGATVEIHLEGFCGGRRFDCKDVKFVVGEGEDHDI
XP_025036815B	DLFE--DGGIIRRIKRKGEGYSNPNEGATVQIHLEGFYGGRIFDCRDVTFVVGEGEDHDI
XP_017582683B	DLFE--DGGIIRRIKRKGEGYSNPNEGATVEIHLEGFCGGRRFDCKDVKFVVGEGEDHDI
XP_011590477B	DLFE--DGGIIRRIKRKGEGYSNPNEGATVEIHLEGFCGGTRFDCKDVKFVVGEGEDHDI
XP_009565641B	DLFE--DGGIIRRIKRKGEGYSNPNEGATVEIRLEGFCGSTRFDCRDVKFVVGEGEDHDI
XP_006276953B	DLFE--DGGIIRRIKKKGEGYSNPNEGATVEIHLEGFYGDRKFDSRDVKFVVGEGEDHDI
XP_014809120B	DLFE--DGGIIRRIKRKGEGYSNPNEGATVEIHLEGFCGGTRFDCKDVKFVVGEGEDHDI
XP_014728244B	DLFE--DGGIIRRIKRKGEGYSNPNEGATVEIHLEGFCGGRRFDCKDVKFVVGEGEDHDI
XP_010014743B	DLFE--DGGIIRRIKRKGEGYSNPNEGATVEIHLEGFCGGTRFDCKDVKFIVGEGEDHDI
XP_021391674B	DLFE--DGGIIRRIKSKGEGYSNPNEGATVEIHLEGFCGGRRFDCKDVKFVVGEGEDHDI
XP_014728242B	DLFE--DGGIIRRIKRKGEGYSNPNEGATVEIHLEGFCGGRRFDCKDVKFVVGEGEDHDI
XP_010402716B	DLFE--DGGIIRRIKRKGEGYSNPNEGATVEIHLEGFCGGRRFDCKDVKFVVGEGEDHDI
XP_009665812B	DLFE--DGGIIRRIKRKGEGYSNPNEGATVEIHLEGFCGGTRFDCKDVKFVVGEGEDHDI
XP_005023535B	DLFE--DGGIIRRIKRKGEGYSNPNEGATVEIHLEGFCGGTRFDCKDVKFVVGEGEDHDI
XP_010582940B	DLFE--DGGIIRRIKKKGEGYSNPNEGATVEIHLEGFCGGTRFDCKDVKFVVGEGEDHDI
XP_002199016B	DLFE--DGGIIRRIKRKGEGYSNPNEGATVEIHLEGFCGGRRFDCKDVKFVVGEGEDHDI
XP_023798001B	DLFE--DGGIIRRIKRKGEGYSNPNEGATVEIHLEGFCGGRRFDCKDVKFVVGEGEDHDI
XP_015505964B	DLFE--DGGIIRRIKRKGEGYSNPNEGATVEIHLEGFCGGHRFDCKDVKFVVGEGEDHDI

XP_023798002B	DLFE--DGGIIRRIKRKGEGYSNPNEGATVEIHLEGFCGGRRFDCKDVKFVVGEGEDHDI
XP_015505967B	DLFE--DGGIIRRIKRKGEGYSNPNEGATVEIHLEGFCGGHRFDCKDVKFVVGEGEDHDI
XP_005529445B	DLFE--DGGIIRRIKRKGEGYSNPNEGATVEIHLEGFCGTRRFDCKDVKFVVGEGEDHDI
XP_014113637B	DLFE--DGGIIRRIKRKGEGYSNPNEGATVEIHLEGFCGTRRFDCKDVKFVVGEGEDHDI
XP_009941273B	DLFE--DGGIIRRIKRKGEGYSNPNEGATVEIHLEGFCGGTRFDCKDVKFVVGEGEDHDI
XP_014113638B	DLFE--DGGIIRRIKRKGEGYSNPNEGATVEIHLEGFCGTRRFDCKDVKFVVGEGEDHDI
XP_017691276B	DLFE--DGGIIRRIKRKGEGYSNPNEGATVEIHLEGFCGGSRFDCKDVKFIVGEGEDHDI
XP_009904479B	DLFE--DGGIIRRIKRKGEGYSNPNEGATVEIHLEGFCSGTRFDCRDVKFVVGEGEDYDI
PKK17972B	DLFE--DGGIIRRIKRKGEGYSNPNEGATVEIHLEGFCGGTRFDCKDVKFVVGEGEDHDI
XP_013052473B	DLFE--DGGIIRRIKRKGEGYSNPNEGATVEIHLEGFCGGTRFDCKDVKFVVGEGEDHDI
XP_017691275B	DLFE--DGGIIRRIKRKGEGYSNPNEGATVEIHLEGFCGGSRFDCKDVKFIVGEGEDHDI
XP_008932530B	DLFE--DGGIIRRIKRKGEGYSNPNEGATVEIHLEGFCGGSRFDCKDVKFIVGEGEDHDI
NP_001005431B	DLFE--DGGIIRRIKRKGEGYSNPNEGATVEIHLEGFCGGTRFDCKDVKFVVGEGEDHDI
XP_010180828B	DLFE--DGGIIRRIKRKGEGYSNPNEGATVEIHLEGFCGGTRFDCKDVKFVVGEGEDHDI
XP_010149712B	DLFE--DGGIIRRIKRKGEGYSNPNEGATVEIHLEGFCGGTRFDCKDVKFVVGEGEDHDI
XP_010722399B	DLFE--DGGIIRRIKRKGEGYSNPNEGATVEIHLEGFCGGTRFDCKDVKFVVGEGEDHDI
XP_021136783B	DLFE--DGGIIRRIKRKGEGYSNPNEGATVEIHLEGFCGGTRFDCKDVKFVVGEGEDHDI
XP_010722398B	DLFE--DGGIIRRIKRKGEGYSNPNEGATVEIHLEGFCGGTRFDCKDVKFVVGEGEDHDI
XP_008497681B	DLFE--DGGIIRRIKRKGEGYSNPNEGATVEIHLEGFWGGTRFDCQDVKFVVGEGEDHDI
OPJ88842B	DLFE--DGGIIRRIKRKGEGYSNPNEGATVEIHLEGFCGGTRFDCKDVKFVVGEGEDHDI
XP_015740263B	DLFE--DGGIIRRIKRKGEGYSNPNEGATVEIHLEGFCGGTRFDCKDVKFVVGEGEDHDI
XP_015740262B	DLFE--DGGIIRRIKRKGEGYSNPNEGATVEIHLEGFCGGTRFDCKDVKFVVGEGEDHDI
EMP32325B	DLFE--DGGIIRRIKRKGEGYSNPNEGATVEIHLEGFCSGRIFDCRDVTFIVGEGEDHDI
XP_009096587B	DLFE--DGGIIRRIKRKGEGYSNPNEGATVEIHLEGFCGGRRFDCKDVKFVVGEGEDHDI
XP_007483818B	DLFE--DGGIIRRIKQKGEGYSNPNEGATVEIHLEGSCGSRTFDCRDVVFVVGEGEDHDI
XP_009636479B	DLFE--DGGIIRRIKRKGEGYSNPNEGATVEIHLEGFCGGTRFDCKDVKFVVGEGEDHDI
XP_005429420B	DLFE--DGGIIRRIKRKGEGYSNPNEGATVEIHLEGFCGGHRFDCKDVKFVVGEGEDHDI
XP_021233042B	DLFE--DGGIIRRIKRKGEGYSNPNEGATVEIHLEGFCGGTRFDCKDVKFVVGEGEDHDI
XP_009463941B	DLFE--DGGIIRRIKRKGEGYSNPNEGATVEIHLEGFCGGTRFDCKDVKFVVGEGEDHDI
XP_021233043B	DLFE--DGGIIRRIKRKGEGYSNPNEGATVEIHLEGFCGGTRFDCKDVKFVVGEGEDHDI
XP_005492860B	DLFE--DGGIIRRIKRKGEGYSNPNEGATVEIHLEGFCGGHRFDCKDVKFVVGEGEDHDI
XP_005492861B	DLFE--DGGIIRRIKRKGEGYSNPNEGATVEIHLEGFCGGHRFDCKDVKFVVGEGEDHDI
XP_019478743B	DLFE--DGGIIRRIKRKGEGYSNPNEGATVEIHLEGFCGGTRFDCKDVKFVVGEGEDHDI
XP_009074619B	DLFE--DGGIIRRIKRKGEGYSNPNEGATVEIHLEGFCGGTRFDCKDVKFVVGEGEDHDI
XP_010005403B	DLFE--DGGIIRRIKRKGEGYSNPNEGATVEIHLEGFCGGTRFDCKDVKFVVGEGEDHDI
XP_009271140B	DLFE--DGGIIRRIKRKGEGYSNPNEGATVEIHLEGFCGGTRFDCKDVKFVVGEGEDHDI
XP_015267313B	DLFE--DGGIIRRIKKKGEGYSNPNEGAAVQIHLKGFCGGRMFDCRDVAFTVGEGEDHDI
XP_015677434B	DLFD--NGGIIRRIKQKGEGYSNPNEGAAVQIHLKGFCDGRMFDCRDVAFTVGEGEDHDI
XP_007426634B	DLFD--NGGIIRRIKQKGEGYSNPNEGAAVQIHLKGFCDGKLFDCRDVAFTVGEGEDHDI
XP_005239689B	DLFE--DGGIIRRIKRKGEGYSNPNEGATVEIHLEGFCGGTRFDCKDVKFIVGEGEDHDI
KFV77537B	DLFE--DGGIIRRIKRKGEGYSNPNEGATVEIHLEGFCGGTRFDCKDVKFVVGEGEDHDI
XP_013909334B	DLFD--NGGIIRRIKQKGEGYSNPNEGAAVQIQLKGFCDGRMFDCRDVAFTVGEGEDHDI
XP_003226528B	DLFE--NGGIIRRIKQKGEGYSNPNEGAAVQIHLKGFCDGRMFDCRDVAFTVGEGEDHDI
XP_005447082B	DLFE--DGGIIRRIKRKGEGYSNPNEGATVEIHLEGFCGGTRFDCKDVKFIVGEGEDHDI
KFO90341B	DLFE--DGGIIRRIKRKGEGYSNPNEGATVEIHLEGFCGGTRFDCKDVKFVVGEGEDHDI
KFO54980B	DLFE--DGGIIRRIKRKGEGYSNPNEGATVEIHLEGFCGGRRFDCKDVKFVVGEGEDHDI
KFQ51336B	DLFE--DGGIIRRIKRKGEGYSNPNEGATVEIHLEGFCGGTRFDCKDVKFIVGEGEDHDI
KFO80933B	DLFE--DGGIIRRIKRKGEGYSNPNEGATVEIRLEGFCGSTRFDCRDVKFVVGEGEDHDI
KFQ22164B	DLFE--DGGIIRRIKRKGEGYSNPNEGATVEIHLEGFCGGTRFDCKDVKFVVGEGEDHDI
KFW76630B	DLFE--DGGIIRRIKRKGEGYSNPNEGATVEIHLEGFCGGSRFDCKDVKFIVGEGEDHDI
KFP15431B	DLFE--DGGIIRRIKRKGEGYSNPNEGATVEIHLEGFCGGTRFDCKDVKFVVGEGEDHDI
XP_006101308B	DLFE--DGGIIRRIKQKGEGYSNPNEGATVEIHLEGRCGERMFDCRDVVFIVGEGEDHDI
KFP81725B	DLFE--DGGIIRRIKRKGEGYSNPNEGATVEIHLEGFCGGTRFDCKDVKFVVGEGEDHDI
XP_005881802B	DLFE--DGGIIRRIKQKGEGYSNPNEGATVEIHLEGRCGERMFDCRDVVFIVGEGEDHDI
KFW05050B	DLFE--DGGIIRRIKRKGEGYSNPNEGATVEIHLEGFCGGTRFDCKDVKFVVGEGEDHDI
KFQ97435B	DLFE--DGGIIRRIKRKGEGYSNPNEGATVEIHLEGFCGGTRFDCKDVKFVVGEGEDHDI
KFP05242B	DLFE--DGGIIRRIKRKGEGYSNPNEGATVEIHLEGFWGGTRFDCQDVKFVVGEGEDHDI
XP_005023534B	DLFE--DGGIIRRIKRKGEGYSNPNEGATVEIHLEGFCGGTRFDCKDVKFVVGEGEDHDI
XP_020650733B	DLFE--NGGIIRRIKQKGEGYSNPNEGAAVQIHLKGFCDGRMFDCRDVAFTVGEGEDHDI
XP_008589828B	DLFE--DGGIIRRIKRKGEGYSNPNEGATVEIHLEGCCGGKMFDCRDVVFIVGEGEDHDI
XP_013052474B	DLFE--DGGIIRRIKRKGEGYSNPNEGATVEIHLEGFCGGTRFDCKDVKFVVGEGEDHDI
KFP55426B	DLFE--DGGIIRRIKRKGEGYSNPNEGATVEIHLEGFCGGTRFDCKDVKFVVGEGEDHDI
KFP95835B	DLFE--DGGIIRRIKKKGEGYSNPNEGATVEIHLEGFCGGTRFDCKDVKFVVGEGEDHDI
XP_021233044B	DLFE--DGGIIRRIKRKGEGYSNPNEGATVEIHLEGFCGGTRFDCKDVKFVVGEGEDHDI
KFV71660B	DLFE--DGGIIRRIKRKGEGYSNPNEGATVEIHLEGFCSGTRFDCRDVKFVVGEGEDYDI
XP_014385088B	DLFE--DGGIIRRIKQKGEGYSNPNEGATVEIHLEGRCGERMFDCRDVVFIVGEGEDHDI
KFU96756B	DLFE--DGGIIRRIKRKGEGYSNPNEGATVEIHLEGFCGGTRFDCKDVKFVVGEGEDHDI
XP_023576318B	DLFE--DGGIIRRIKRKGEGYSNPNEGATVDIHLEGRYGGRMFDCRDVVFIVGEGEDHDI
XP_023576325B	DLFE--DGGIIRRIKRKGEGYSNPNEGATVDIHLEGRYGGRMFDCRDVVFIVGEGEDHDI
KFM03271B	DLFE--DGGIIRRIKRKGEGYSNPNEGATVEIHLEGFCGGTRFDCKDVKFVVGEGEDHDI
XP_014352291B	DLYE--DGGIIRRIKVKGEGYSNPNEGATVEISLEAKCYNRVFDCRDVKFVVGEGEDQGI
XP_015197960B	ELTA--DGGVVRRIKEKGEGFNNPNEGATVEVHLEGSCGGRVFDSRDVRFVVGEAEEKGV
XP_015197959B	ELTA--DGGVVRRIKEKGEGFNNPNEGATVEVHLEGSCGGRVFDSRDVRFVVGEAEEKGV

```
XP_017334936B       ELTE--DSGILRRIKVKGEGYSNPNDGATVHVHLEGRCCGRLFDSRDATFVVGESEDMGV
XP_017334935B       ELTE--DSGILRRIKVKGEGYSNPNDGATVHVHLEGRCCGRLFDSRDATFVVGESEDMGV
XP_017334933B       ELTE--DSGILRRIKVKGEGYSNPNDGATVHVHLEGRCCGRLFDSRDATFVVGESEDMGV
XP_020371994B       DLTECEDGGIIRRIKKKGQGYSTPNEGATVEVYLEGRTNAVLFDQRDVTFVVGEGEDQDI
XP_023669222B       ELTE--DGGIVRRIKVKGEGFSNPNEGATVSVHIQGSCNGRVFDKRDVCFVVGEAEDVSV
XP_017541827B       ELTE--DGGILRRIKVKGEGYNNPNEGATVHVHLEGRCGGRLFEARDITFVVGESEDMSV
XP_016372735B       ELTE--DGGIVRRIKVKGEGYYNPNEGATVHVHLEGRCGGRLFDSRDVTFVVGESEDVGV
XP_016322567B       ELTE--DGGIVRRIKVKGEGYYNPNEGATVHVHLEGRCGGRLFDSRDVTFVVGESEDVGV
XP_018602419B       ELTE--DGGIVRRIKVKGQGFRYPNVGATVSVHLQGSCDGKIFDSRDVSFVVGEGDDVNV
XP_019805261B       DLFE--DGGIIRRIKRKGEGYSNPNEGATVEIHLEGRCGGRMFDCRDVGFIVGEGEDHDI
XP_013987820B       VLTD--DGGITRRIKIKGEGYNNPNDGSTVHVHLEGRCEGRLFDSRDVHFVVGESEDMGV
XP_023843348B       VLTD--DGGITRRIKVKGEGYNNPNDGSTVYVHLEGRCEERLFDSRDVQFVVGESEDMGV
XP_021332470B       ELTE--DGGIVRRIKVKGEGYSNPNEGATVHVHLEGWCGGRLFDSRDVTFAVGESEDVGV
XP_005166078B       ELTE--DGGIVRRIKVKGEGYSNPNEGATVHVHLEGWCGGRLFDSRDVTFAVGESEDVGV
XP_021424491B       VLTD--DGGITRRIKVKGEGYNNPNDGSTVHVHLEGRCEERLFDSRDVHFVVGESEDMGV
NP_998314B          ELTE--DGGIVRRIKVKGEGYSNPNEGATVHVHLEGWCGGRLFDSRDVTFAVGESEDVGV
XP_016314457B       ELLE--DGGIVRRIKVKGEGYNNPNEGATVHVHLEGWCGGWLFDSRDVTFVVGESEDVGV
XP_024283464B       VLTD--DGGITRRIKVKGEGYNSPNDGSTVHVHLEGRCGERLFDSRNVHFIVGESEDMGV
XP_012670459B       ELTE--DGGIVRRIKVKGEGYNNPNDGASVHVHLKGSYGGRVFDTRDVHFIVGESEDAGV
XP_021465979B       VLTD--DGGITRRIKVKGEGYNSPNDGSTVHVHLEGRCGERLFDSRNVHFVVGESEDMGV
XP_020339278B       VLTD--DGGITRRIKVKGEGYNSPNDGSTVHVHLEGRCGERLFDSRNVHFVVGESEDMGV
XP_005142250B       DLFE--DGGIIRRIKRKGEGYSNPNEGATVEIHLEGFCGGTRFDCKDVKFIVGEGEDHDI
XP_024229852B       VLTD--DGGITRRIKVKGEGYNNPNDGSTVHVHLEGRCEERLFDSRDVHFVVGESEDMGV
XP_023852739B       VLTD--DGGITRRIKVKGEGYNSPNDGSTVHVHLEGRCGERLFDSRNVHFVVGESEDMGV
XP_020342285B       VLTD--DGGITRRIKVKGEGYNNPNDGSTVHVHLEGRCEERLFDTRDVHFVVGESEDMGV
XP_016108165B       ELLE--DGGIVRRIKVKGEGYNNPNEGATVHVHLEGWCGGWLFDSRDVTFVVGESEDVGV
XP_023270066B       MLTD--DGGIMRRIKVKGDGYTNPNDGASVDVHLEGSCGGRLFDCRDVTFIVGEAEDKGV
XP_018541261B       MLTD--DGGIIRRIKVKGDGYTNPNDGASVDVHLEGSCGGRLFDCRDVSFIVGEAEDKGV
XP_007899824B       DLTEGEDGGIIRRIKKKGEGYFSPNEGATVEVSIQGKCQNVLFDRREVKFVVGEGDDRDI
XP_022602390B       MLTD--DGGIMRRIKVKGDGYTNPNDGASVDVHLEGSCGGRLFDCRDVTFIVGEAEDKGI
XP_020507498B       TLTH--DSGILRRIKVKGDGYTNPNDGASVDVHLEGSCGGRLFDCRDVSFIVGEAEDKGV
XP_022602392B       MLTD--DGGIMRRIKVKGDGYTNPNDGASVDVHLEGSCGGRLFDCRDVTFIVGEAEDKGI
XP_022602391B       MLTD--DGGIMRRIKVKGDGYTNPNDGASVDVHLEGSCGGRLFDCRDVTFIVGEAEDKGI
XP_016384571B       ELLE--DGGIVRRIKVKGEGYNNPNEGATVHVHLEGWCGGWLFDSRDVTFVVGESEDVGV
XP_007937638B       DLFE--DGGIIRRIKRKGEGYSNPNEGATVEVHLVGHCGGQLFDCRDVVFIVGEGEDHDI
XP_009894963B       DLTDDEDGGIIRRIHKKGEGYSKPNEGALVEIQFEGRCGDRVFDRRELQFEIGEGDDYDI
XP_014843613B       ILTA--DGGIMRRIKVKGEGYSNPNDGATVDVHLEGRCGDRLFDCRDVSFIVGQGEDKGI
XP_020789713B       LLTD--DGGIMRRIKVKGDGFLSPNDGATVHVHLEGRCDGQLFDCRDVTFSVGEGEDKGI
XP_006783146B       VLTG--DGGIVRRIKVKGEGYTNPNDGSIVNVHLEGRCGDRLFDCRDVSFIVGQAEDKSI
XP_004548817B       VLTG--DGGIVRRIKVKGEGYTNPNDGSIVNVHLEGRCGDRLFDCRDVSFIVGQAEDKSI
XP_003448395B       ALTG--DGGIVRRIKVKGEGYTNPNDGSVVNVHLEGRCGDRLFDCRDVSFIVGQAEDKSI
KFV64423B           DLTDDEDGGIIRRIHKKGEGYSKPNEGALVEIQFEGRCGDRVFDRRELQFEIGEGDDYDI
XP_007903293B       DLTEDEDGGIMRRIRKKGEGYSKPNEGALVEIHLEGQYQGTVFDSRDVSFVLGDAEENDI
XP_005927577B       VLTG--DGGIVRRIKVKGEGYTNPNDGSIVNVHLEGRCGDRLFDCRDVSFIVGQAEDKSI
XP_006265443B       DLTDDEDGGIIRRIHKKGEGYSKPNEGALVEIQLEGRHGERVFDKRELRFEIGEGENHDL
XP_014795643B       DLTDEEDGGIIRRIRKKGEGYSKPNEGAIVEIQFEGQYGGRVFDRRELQFEIGEGDNYDL
XP_006035184B       DLTDDEDGGIIRRIHKKGEGYSKPNEGALVEIQLEGRHGERVFDKRELQFEIGEGENHDL
XP_021177252B       ILTT--DGGIVRRIKVKGEGYSNPNDGASVDVHLEGRCGDRLFDCRDVSFVVGEGEDKGI
XP_005724522B       VLTG--DGGIVRRIKVKGEGYTNPNDGSIVNVHLEGRCGDRLFDCRDVSFIVSQAEDKSI
XP_007551477B       ILTA--DGGIMRRIKVKGEGYSNPNDGATVDVHLEGRCGDRLFDCRDVSFIVGQGEDKGI
XP_017160280B       ILTA--DGGIMRRIKVKGEGYSNPNDGATVDVHLEGRCGDRLFDCRDVSFIVGQGEDKGI
XP_008406990B       ILTA--DGGIMRRIKVKGEGYSNPNDGATVDVHLEGRCGDRLFDCRDVSFIVGQGEDKGI
XP_010573701B       DLTDDEDGGIIRRIRKKGEGYSKPNEGALVEIQFEGRYGDRVFDRRELRFEIGEGDNYDL
XP_022531443B       ELTE--DGGILRRIKVKGEGYNNPNEGATVHVHLEGRCGDRLFQSRDITFVVGESEDKGI
XP_005810495B       ILTA--DGGIMRRIKVKGEGYSNPNDGASVDVHLEGRCGDRLFDCRDVGFIVGQGEDKGI
XP_022044943B       ALTD--DGGIIRRIKVKGDGYTNPNDGATVDVHLQGSCGGRLFDCRDVSFIVGEGEDKSI
XP_014897893B       ILTA--DGGIMRRIKVKGEGYSNPNDGATVDVHLEGRCGDRLFDCRDVSFIVGQGEDKGI
XP_023136030B       ALTD--DGGIVRRIKVKGDGYTNPNDGASVDVHLQGSCGGRLFDCRDVSFVVGEGEDKGI
OPJ69275B           DLTDEEDGGIIRRIRKKGEGYSKPNEGAVVEIQFEGRCGDRVFDSRELRFEIGEGDNYDL
XP_008109533B       DLTDDEDGGIIRRIRKKGEGYSKPNEGALVEIEVEGWHGNRVFDKRELRFEVGEGENYDL
XP_014349043B       DVTVEEDRGIIRRIHMSGEGYSKPHEGSTVEIHLEAECAGRVFDSRDLTFEIGDGDSYDI
XP_008283913B       ALTD--DGGIIRRIKVKGDGYTNPNDGASVDVRLQGSCGGRLFDCRDVSFIVGEGEDKGI
XP_006633816B       DLTDDEDGGIIRRIATKGEGYSKANEGATVEVSLEGTFEGRVFDQRELKFEIGDGESLGI
XP_008071742B       DLFE--DGGIIRRIRRKGEGYSNPNEGATVEIYLEGHCGERMFDCRDVVFTVGEGEDHDI
XP_006035186B       DLTDDEDGGIIRRIHKKGEGYSKPNEGALVEIQLEGRHGERVFDKRELQFEIGEGENHDL
XP_019964054B       TLTD--DGGIVRRIKVKGEGYTNPNDGATVEVHLEGSCDGRLFDCRDVTFVVGEAEDKGI
XP_017685573B       DLTDEEDGGIIRRIRKKGEGYSKPNEDALVEIQFEGRYGDRVFDKRELRFEIGEGENFDL
XP_005531080B       DLTDEEDGGIIRRIRKKGEGYSRPNEDALVEIQFEGRHGDRVFDKRELRFEIGEGENFDI
XP_015814339B       VLSE--DGGIIRRIKVKGDGYSNPNDGANVDVHVEGRCGDRLFDCRDVSFTVGQGEDKGI
XP_013811689B       DLTDDEDGGIIRRIRKKGEGYSKPNEGALVEIQFEGRYGDRMFDRRELQFEIGEGDNYDL
XP_021249927B       DLTDDEDGGIIRRIRKKGEGYSKPNEGALVEIQFEGRYRDRVFDRRELRFEVGEGDNYDL
                     :    : *: **    *:*:  .: .: * : . .      *: ::  * :.:.:.  :
```

NP_004108B	PIGIDKALEKMQREEQCILYLGPRY-GFGEAGKPKFGIEPNAELIY
XP_518427B	PIGIDKALEKMQREEQCILYLGPRY-GFGEAGKPKFGIEPNAELIY
XP_004043925B	PIGIDKALEKMQREEQCILYLGPRY-GFGEAGKPKFGIEPNAELIY
XP_003278903B	PIGIDKALEKMQREEQCILYLGPRY-GFGETGKPKFGIEPNAELIY
NP_001124689B	PIGIDKALEKMQREEQCILYLGPRY-GFGEAGKPKFGIEPNAELIY
XP_010387961B	PIGIDKALEKMQREEQCILYLGPRY-GFGEAGKPKFGIEPNAELIY
XP_023068393B	PIGIDKALEKMQREEQCILYLGPRY-GFGEAGKPKFGIEPNAELIY
AAA86245B	PIGIDKALEKMQREEQCILYLGPRY-GFGEAGKPKFGIEPNAELIY
XP_003897547B	PIGIDKALEKMQREEQCILYLGPRY-GFGEAGKPKFGIEPNAELIY
XP_011841956B	PIGIDKALEKMQREEQCILYLGPRY-GFGEAGKPKFGIEPNAELIY
XP_011800768B	PIGIDKALEKMQREEQCILYLGPRY-GFGEAGKPKFGIEPNAELIY
XP_005553272B	PIGIDKALEKMQREEQCILYLGPRY-GFGEAGKPKFGIEPNAELIY
XP_007971022B	PIGIDKALEKMQREEQCILYLGPRY-GFGEAGKPKFGIEPNAELIY
Q95L05B	PIGIDKALEKMQREEQCILYLGPRY-GFGEAGKPKFGIEPNAELIY
XP_011930726B	PIGIDKALEKMQREEQCILYLGPRYMGFGEAGKPKFGIEPNAELIY
XP_022437414B	PIGIDKALEKMQREEQCILYLGPRY-GFGEAGKPAFGIEPNAELIY
XP_004267730B	PIGIDKALEKMQREEQCILYLGPRY-GFGEAGKPEFGIEPNAELIY
XP_007467316B	PIGIDKALEKMQREEQCILYLGPRY-GFGEAGKPEFGVEPNAELIY
XP_006202142B	PIGIDKALEKMQREEQCILYLGPRY-GFGEAGKPKFGIEPNAELIY
XP_020024507B	PIGIDKALEKMQREEQCILYLGPRY-GFGEAGKPKFGIEPNAELIY
XP_013833281B	PIGIDKALEKMQREEQCILYLGPRY-GFGEAGKPKFGIEPNAELIY
XP_020024506B	PIGIDKALEKMQREEQCILYLGPRY-GFGEAGKPKFGIEPNAELIY
XP_020953710B	PIGIDKALEKMQREEQCILYLGPRY-GFGEAGKPKFGIEPNAELIY
NP_001302540B	PIGIDKALEKMQREEQCILYLGPRY-GFGEAGKPKFGIEPNAELIY
XP_020142566B	PIGIDKALEKMQREEQCILYLGPRY-GFGEAGKPKFGIEPNAELIY
XP_006180531B	PIGIDKALEKMQREEQCILYLGPRY-GFGEAGKPKFGIEPNAELIY
XP_015343090B	PIGIDKALEKMQREEQCILYLGPRY-GFGEAGKPKFGIEPNAELIY
XP_005318831B	PIGIDKALEKMQREEQCILYLGPRY-GFGEAGKPKFGIEPNAELVY
XP_010947329B	PIGIDKALEKMQREEQCILYLGPRY-GFGEAGKPKFGIEPNAELTY
XP_023977801B	PIGIDKALEKMQREEQCILYLGPRY-GFGEAGKPKFGIEPNAELIY
XP_020742361B	PIGIDKALEKMQREEQCILYLGPRY-GFGEAGKPKFGIEPNAELIY
XP_007198040B	PIGIDKALEKMQREEQCILYLGPRY-GFGEAGKPNFGIEPNAELIY
XP_017894445B	PIGIDKALEKMQREEQCILYLGPRY-GFGEAGKPKFGIEPNAELIY
XP_002714685B	PIGIDKALEKMQREEQCILYLGPRY-GFGEAGKPKFGIEPNAELIY
XP_012502082B	PIGIDKALEKMQREEQCILYLGPRY-GFGEAGKPKFGIEPNAELIY
XP_004379454B	PIGIDKALEKMQREEQCILYLGPRY-GFGEAGKPKFGIEPNAELIY
XP_023368573B	PIGIDKALEKMQREEQCILYLGPRY-GFGEAGKPKFGIEPNAELIY
XP_014958158B	PIGIDKALEKMQREEQCILYLGPRY-GFGEAGKPKFGIEPNAELIY
NP_001295455B	PIGIDKALEKMQREEQCILHLGPRY-GFGEAGKPKFGIEPNAELIY
XP_003789178B	PIGIDKALEKMQREEQCILYLGPRY-GFGEAGKPKFGIEPNAELIY
XP_006050442B	PIGIDKALEKMQREEQCILYLGPRY-GFGEAGKPKFGIEPNAELIY
XP_005979785B	PIGIDKALEKMQREEQCILYLGPRY-GFGEAGKPKFGIEPNAELIY
XP_004770745B	PIGIDKALEKMQREEQCILYLGPRY-GFGEAGKPKFGIEPNAELMY
XP_011967348B	PIGIDKALEKMQREEQCILYLGPRY-GFGEAGKPKFGIEPNAELIY
NP_001179791B	PIGIDKALEKMQREEQCILYLGPRY-GFGEAGKPKFGIEPNAELIY
XP_017826524B	PIGIDKALEKMQREEQCILHLGPRY-GFGEAGKPKFGIEPNAELIY
XP_004424279B	PIGIDKALEKMQREEQCILYLGPRY-GFGEAGKPEFGIEPNAELIY
NP_001266943B	PIGIDKALEKMQREEQCILHLGPRY-GFGEAGKPKFGIEPNAELIY
XP_022366563B	PIGIDKALEKMQREEQCILYLGPRY-GFGEAGKPKFGIDPNAELMY
XP_017378122B	PIGIDKALERMQREEQCILHLGPRY-GFGEAGKPKFGIEPNAELIY
XP_023480581B	PIGIDKALEKMQREEQCILYLGPRY-GFGEAGKPKFGIEPNAELIY
XP_006144244B	PIGIDKALEKMQREEQCILCLGPRY-GFGEAGKPKFGIEPNAELLY
XP_006882111B	PIGIDKALEKMQREEQCILYLGPRY-GFGEAGKPKFGIKPNADLIY
XP_008528289B	PIGIDKALEKMQREEQCILYLGPRY-GFGEAGKPKFGIEPNAELIY
XP_538880B	PIGIDKALEKMQREEQCILYLGPRY-GFGEAGKPKFGIEPNAELIY
XP_012885079B	PIGIDKALEKMQREEQCILYLGPRY-GFGEAGKPKFGIEPNAELIY
XP_012420443B	PIGIDKALEKMQREEQCILYLGPRY-GFGEAGKPKFGIEPNAELIY
Q9XSI2B	PIGIDKALEKMQREEQCILHLGPRY-GFGEAGKPKFGIEPNAELIY
XP_006739186B	PIGIDKALEKMQREEQCILHLGPRY-GFGEAGKPKFGIEPNAELTY
XP_003404000B	PIGIDKALEKMQREEQCILYLGPRY-GFGEAGKPKFGIEPNAELIY
XP_021540340B	PIGIDKALEKMQREEQCILHLGPRY-GFGEAGKPKFGIEPNAELIY
XP_002914343B	PIGIDKALEKMQREEQCILYLGPRY-GFGEAGKPKFGIEPNAELIY
XP_006860458B	PIGIDKALEKMQREEQCILYLGPRY-GFGEAGKPKFGIEPNADLIY
XP_008691621B	PIGIDKALEKMQREEQCILYLGPRY-GFGEAGKPKFGIDPNAELIY
XP_004624255B	PIGIDKAMEKMQREEQCILYLGPRY-GFGEAGKPKFGIEPNAELIY
XP_006907392B	PIGIDKALEKMQREEQCILYLGPRY-GFGEAGKPKFGIEPNAELMY
XP_006759086B	PIGIDKALEKMQREEQCVLCLGPRY-GFGEAGKPKFGIEPNAELIY
XP_007534316B	PIGIDKALEKMQREEQCILYVGPRY-GFGEAGKPKFGIEPNAELIY
XP_004590364B	PIGIDKALEKMQREEQCILYLGPRY-GFGEAGKPKFGIEPNAELIY
XP_005389619B	PIGIDKALEKMQREEQCILYLGPRY-GFGEAGKPKFGIEPNAELIY
XP_013372357B	PIGIDKALEKMQREEQCILYLGPRY-GFGEAGKPKFGIEPNAELIY
XP_004846760B	PIGIDKALEKMQREEQCILYLGPRY-GFGEAGKPKFGIEPNAELIY
XP_006101307B	PIGIDKALEKMQREEQCILCLGPRY-GFGEAGKPKFGIEPNAELIY

XP_011280550B	PIGIDKALEKMQREEQCILYLGPRY-GFGEAGKPKFGIEPNAELIY
XP_019312428B	PIGIDKALEKMQREEQCILYLGPRY-GFGEAGKPKFGIEPNAELIY
XP_014925310B	PIGIDKALEKMQREEQCILYLGPRY-GFGEAGKPKFGIEPNAELMY
XP_016079225B	PIGIDKALEKMQREEQCILHVGPRY-GFGEAGKPKFGIEPNAELIY
XP_010603642B	PIGIDKALEKMQREEQCVLHLGPRY-GFGEAGKPKFGIEPNAELIY
XP_005881801B	PIGIDKALEKMQREEQCILCLGPRY-GFGEAGKPKFGIEPNAELIY
XP_012577888B	PIGIDKALEKMQREEQCILFLGPRY-GFGEAGKPKFGIEPNAELIY
XP_007085336B	PIGIDKALEKMQREEQCILYLGPRY-GFGEAGKPKFGIEPNAELIY
XP_003473945B	PLGIDKALEKMQREEQCILFLRPQY-GFGEAGKPKFGIEPNAELMY
XP_019594281B	PIGIDKALEKMQREEQCILRLDPRY-GFGEAGKPKFGIEPNAELIY
XP_004710782B	PIGIDKALEKMQREEQCLLYLGPRY-GFGEAGKPKFGIEPNAELTY
XP_015975694B	PIGIDRALEKMQREEQCILYLGPRY-GFGEAGKPKFGIEPNVELMY
XP_006256284B	PIGIDKALEKMQREEQCILYLGPQY-GFGEAGKPKFGIEPNAELMY
NP_001012174B	PIGIDKALEKMQREEQCILYLGPQY-GFGEAGKPKFGIEPNAELMY
XP_019523002B	PIGIDKALEKMQREEQCILHLGPRY-GFGEAGKPKFGIEPNAELLY
XP_003508661B	PIGIDKALEKMQREEQCVLYLGPRY-GFGEAGKPKFGIEPNAELMY
XP_008838320B	PIGIDKALEKMQREEQCILYLGPRY-GFGEAGKPKFGIEPNAELIY
XP_021792868B	PIGIDKALEKMQREEQCILYLGPRY-GFGEAGKPKFGIEPNAELIY
BAH13948B	PIGIDKALEKMQREEQCILYLGPRY-GFGEAGKPKFGIEPNAELIY
XP_005084951B	PIGIDKALEKMQREEQCVLYLGPRY-GFGESGKPKFGIEPNAELIY
XP_005360413B	PIGVDKALEKMQREEQCVLYLGPRY-GFGEAGKPKFGIEPNAELTY
XP_024839424B	PIGIDKALEKMQREEQCILYLGPRY-GFGEAGKPKFGIEPNAELIY
XP_004617764B	PIGIDKALEKMQREEQCVLYLGPRY-GFGEAGKPKFGIEPNAELIY
XP_021485845B	PIGIDRALEKMQRGEQCILYLGPRY-GFGEAGKPEFDIKPNAELTY
XP_006983174B	PIGIDKALEKMQREEQCVLYLGPRY-GFGEAGKPKFGIEPNAELMY
XP_004649837B	PIGIDKALQKMQREEQCLLHLSPRY-GFGEAGKPKFGIEPNAELMY
EFB14016B	PIGIDKALEKMQREEQCILYLGPRY-GFGEAGKPKFGIEPNAELIY
XP_007483815B	PIGIDKALEKMQREEHCILYLGPRY-GFGETGKPEFGIEPNAELVY
XP_014958162B	PIGIDKALEKMQREEQCILYLGPRY-GFGEAGKPKFGIEPNAELIY
XP_020855250B	PIGIDKALEKMQREEHCILYLGPRY-GFGEAGKPEFGIEPNAELVY
XP_011967352B	PIGIDKALEKMQREEQCILYLGPRY-GFGEAGKPKFGIEPNAELIY
XP_021076628B	PIGIDKALVKMQREEQCILYLGPRY-GFGEAGKPKFGIDPNAELMY
XP_003769030B	PIGIDKALEKMQREEHCILYLGPRY-GFGESGKPEFGIEPNAELVY
XP_023589897B	PIGIDKALEKMQREEQCILYLGPRY-GFGEAGKPKFGIEPNAELIY
XP_004458369B	PIGIDKALERMQREEHCILHLGPRY-GFGEAGKPKFGIEPNAALTY
XP_004770751B	PIGIDKALEKMQREEQCILYLGPRY-GFGEAGKPKFGIEPNAELMY
NP_034350B	PIGIDKALVKMQREEQCILYLGPRY-GFGEAGKPKFGIDPNAELMY
BAE35044B	PIGIDKALVKMQREEQCILYLGPRY-GFGEAGKPKFGIDPNAELMY
XP_006882112B	PIGIDKALEKMQREEQCILYLGPRY-GFGEAGKPKFGIKPNADLIY
XP_021005468B	PIGIDKALVKMQREEQCILYLGPRY-GFGEAGKPKFGIDPNAELMY
XP_012420445B	PIGIDKALEKMQREEQCILYLGPRY-GFGEAGKPKFGIEPNAELIY
XP_004590366B	PIGIDKALEKMQREEQCILYLGPRY-GFGEAGKPKFGIEPNAELIY
XP_024413417B	PIGIDKALEKMQRGEQCILFLGSRY-GFGESGKPSFGIEPNAELMY
XP_007063551B	PIGIDKALEKMQREEYCILYLGPRY-GFGEAGKPKFGIEANAELVY
XP_005059495B	PIGIDKALEKMQRGEHCILYLSPRY-GFGEAGKPKYGIQGNVELVY
XP_010135429B	PIGIDKALEKMQRGEQCILYLGPRY-GFGEAGKPKYGIQANAELVY
XP_024066292B	PIGIDKALEKMQREEYCILYLGPRY-GFGEAGKPKFGIEANAELVY
XP_013802188B	PIGIDKALEKMQRGEHCILYLGPRY-GFGEAGKPKYGIQANAELVY
XP_014376143B	PIGIDKALEKMQRGEHCILYLGPRY-GFGEAGKPKFGIEANAELAY
XP_005311709B	PIGIDKALEKMQREEYCILYLGPRY-GFGEAGKPKFGIKANAELVY
XP_019401755B	PIGIDKALEKMQRGEHCILYLGPRY-GFGEAGKPKFGIEANAELAY
XP_019369315B	PIGIDKALEKMQRGEHCILYIGPRY-GFGEAGKPKFGIEANAELAY
XP_014728245B	PIGIDKALEKMQRGEHCILYLSPRY-GFGEAGKPKYGIQGNTELVY
XP_025036815B	PIGIDKALEKMQREEHCILYLGPRY-GFGESGKPKFGIEANAELVY
XP_017582683B	PIGIDKALEKMQRGEHCILYLSPRY-GFGEAGKPKYGIQGNAELVY
XP_011590477B	PIGIDKALEKMQRGEHCILYLGPRY-GFGEAGKPKYGIQANAELVY
XP_009565641B	PIGIDKALEKMQRGEHCILYLGPRY-GFGEGGKPKYGIQPNAELVY
XP_006276953B	PIGIDKALEKMQRGEHCILYLGPRY-GFGEAGKPKFGIEANAELAY
XP_014809120B	PIGIDKALEKMQRGEHCILYLGPRY-GFGEAGKPKYGIQANAELVY
XP_014728244B	PIGIDKALEKMQRGEHCILYLSPRY-GFGEAGKPKYGIQGNTELVY
XP_010014743B	PIGIDKALEKMQRGEHCILYLAPRY-GFGEAGKPKYGIQANAELVY
XP_021391674B	PIGIDKALEKMQRGEHCILYLSPRY-GFGEAGKPKFGIQGNAELVY
XP_014728242B	PIGIDKALEKMQRGEHCILYLSPRY-GFGEAGKPKYGIQGNTELVY
XP_010402716B	PIGIDKALEKMQRGEHCILYLSPRY-GFGEAGKPKYGIQGNVELVY
XP_009665812B	PIGIDKALEKMQRGEHCILYLGPRY-GFGEAGKPKYGIQANAELVY
XP_005023535B	PIGIDKALEKMQRGEHCVLYLGPRY-GFGEAGKPKYGIQANAELVY
XP_010582940B	PIGIDKALEKMQRGEHCILYLGPRY-GFGEAGKPKYGIQANAELVY
XP_002199016B	PIGIDKALEKMQRGEHCILYLSPRY-GFGEAGKPKFGIQGNAELVY
XP_023798001B	PIGIDKALEKMQRGEHCILYLSPRY-GFGEAGKPKYGIQGNAKLVY
XP_015505964B	PIGIDKALEKMQRGEHCILYLSPRY-GFGEAGKPKYGIQGNAELVY
XP_023798002B	PIGIDKALEKMQRGEHCILYLSPRY-GFGEAGKPKYGIQGNAKLVY
XP_015505967B	PIGIDKALEKMQRGEHCILYLSPRY-GFGEAGKPKYGIQGNAELVY
XP_005529445B	PIGIDKALEKMQRGEHCILYLSPRY-GFGEAGKPKYGIQGNAELVY

```
XP_014113637B      PIGIDKALEKMQRGEHCILYLSPRY-GFGEAGKPKYGIQGNAELVY
XP_009941273B      PIGIDKALEKMQRGEHCVLYLGPRY-GCGEAGKPKYGIQANAELVY
XP_014113638B      PIGIDKALEKMQRGEHCILYLSPRY-GFGEAGKPKYGIQGNAELVY
XP_017691276B      PIGIDKALEKMQRGEHCILYLSPRY-GFGEAGKPKYGIQANAELVY
XP_009904479B      PIGIDKALEKMQRAEHCILYLAPRY-GFGEAGKPKYGIQANAELVY
PKK17972B          PIGIDKALEKMQRGEHCVLYLGPRY-GFGETGKAKYGIQANAELVY
XP_013052473B      PIGIDKALEKMQRGEHCVLYLGPRY-GFGEAGKPKYGIQANAELVY
XP_017691275B      PIGIDKALEKMQRGEHCILYLSPRY-GFGEAGKPKYGIQANAELVY
XP_008932530B      PIGIDKALEKMQRGEHCILYLSPRY-GFGEAGKPKYGIQANAELVY
NP_001005431B      PIGIDKALEKMQRGEHCILYLGPRY-GFGEAGKPKYGIQANAELVY
XP_010180828B      PIGIDKALEKMQRGEQCILYLGPRY-GFGEAGKPKYGIQANAELVY
XP_010149712B      PIGIDKALEKMQRGEHCILYLGPRY-GFGEAGKPKYGIPANAELVY
XP_010722399B      PIGIDKALEKMQRGEHCVLYLGPRY-GFGEAGKPKYGIQANAELVY
XP_021136783B      PIGIDKALEKMQRGEHCVLYLGPRY-GFGETGKAKYGIQANAELVY
XP_010722398B      PIGIDKALEKMQRGEHCVLYLGPRY-GFGEAGKPKYGIQANAELVY
XP_008497681B      PIGIDKALEKMQRGEHCVLHLGPRY-GFGELGKPKFGIPGNAELVY
OPJ88842B          PIGIDKALEKMQRGEHCVLYLGPRY-GFGETGKAKYGIQANAELVY
XP_015740263B      PIGIDKALEKMQRGEHCILYLGPRY-GFGEAGKPKYGIQANAELVY
XP_015740262B      PIGIDKALEKMQRGEHCILYLGPRY-GFGEAGKPKYGIQANAELVY
EMP32325B          PIGIDKALEKMQREEYCILYLGPRY-GFGEAGKPKFGIEANAELVY
XP_009096587B      PIGIDKALEKMQRGEHCILYLSPRY-GFGEAGKPKFGIQGNAELVY
XP_007483818B      PIGIDKALEKMQREEHCILYLGPRY-GFGETGKPEFGIEPNAELVY
XP_009636479B      PIGIDKALEKMQRGEHCVLYLGPRY-GFGEAGKPKYGIQANAELVY
XP_005429420B      PIGIDKALEKMQRGEHCILYLSPRY-GFGEAGKPKFGIQGNAELVY
XP_021233042B      PIGIDKALEKMQRGEHCILHLGPRY-GFGEAGKPKYGIQANAELVY
XP_009463941B      PIGIDKALEKMQRGEHCVLYLGPRY-GFGEAGKPKYGIQANAELVY
XP_021233043B      PIGIDKALEKMQRGEHCILHLGPRY-GFGEAGKPKYGIQANAELVY
XP_005492860B      PIGIDKALEKMQRGEHCILYLSPRY-GFGEAGKPKFGIQGNAELVY
XP_005492861B      PIGIDKALEKMQRGEHCILYLSPRY-GFGEAGKPKFGIQGNAELVY
XP_019478743B      PIGIDKALEKMQRGEHCVLYLGPRY-GFGEAGKPKYGIQANAELVY
XP_009074619B      PIGIDKALEKMQRGEHCILYLSSRY-GFGEAGKPKYGIQANAELVY
XP_010005403B      PIGIDKALEKMQRGEHCVLHLGPRY-GFGEAGKPKYGIPGNAELVY
XP_009271140B      PIGIDKALEKMQRGEHCVLYLGPRY-GFGEAGKPKYGIQANAELVY
XP_015267313B      PIGIDKALEKMQRGEHCILHIGAPY-GFGEAGKRKFGIEPNIELIY
XP_015677434B      PIGIDKALEKMQRGEHCILHIGTQY-GFGEAGKPVFGIEPNADLVY
XP_007426634B      PIGIDKALEKMQRGEHCILHIGTQY-GFGEAGKPIFGIEPNAELVY
XP_005239689B      PIGIDKALEKMQRGEHCILYLGPRY-GFGEAGKPKYGIQANAELVY
KFV77537B          PIGIDKALEKMQRGEHCILYLGPRY-GFGEAGKPKYGIQANAELVY
XP_013909334B      PIGIDKALEKMQRGEHCILHIGTQY-GFGEGGKPVFGIEPNAELVY
XP_003226528B      PIGIDKALEKMQRGEHCILHIGAQY-GFGEAGKLAFGIGPNTDLVY
XP_005447082B      PIGIDKALEKMQRGEHCILYLGPRY-GFGEAGKPKYGIQANAELVY
KFO90341B          PIGIDKALEKMQRGEQCILYLGPRY-GFGEAGKPKYGIQANAELVY
KFO54980B          PIGIDKALEKMQRGEHCILYLSPRY-GFGEAGKPKYGIQGNAELVY
KFQ51336B          PIGIDKALEKMQRGEHCILYLAPRY-GFGEAGKPKYGIQANAELVY
KFO80933B          PIGIDKALEKMQRGEHCILYLGPRY-GFGEGGKPKYGIQPNAELVY
KFQ22164B          PIGIDKALEKMQRGEQCILYLGPRY-GFGEAGKPKYGIQANAELVY
KFW76630B          PIGIDKALEKMQRGEHCILYLSPRY-GFGEAGKPKYGIQANAELVY
KFP15431B          PIGIDKALEKMQRGEHCVLYLGPRY-GFGEAGKPKYGIQANAELVY
XP_006101308B      PIGIDKALEKMQREEQCILCLGPRY-GFGEAGKPKFGIEPNAELIY
KFP81725B          PIGIDKALEKMQRGEHCILYLSSRY-GFGEAGKPKYGIQANAELVY
XP_005881802B      PIGIDKALEKMQREEQCILCLGPRY-GFGEAGKPKFGIEPNAELIY
KFW05050B          PIGIDKALEKMQRGEHCILYLGPRY-GFGEAGKPKYGIPANAELVY
KFQ97435B          PIGIDKALEKMQRGEHCVLYLGPRY-GFGEAGKPKYGIQANAELVY
KFP05242B          PIGIDKALEKMQRGEHCVLHLGPRY-GFGELGKPKFGIPGNAELVY
XP_005023534B      PIGIDKALEKMQRGEHCVLYLGPRY-GFGEAGKPKYGIQANAELVY
XP_020650733B      PIGIDKALEKMQRGEQCILHIGAQY-GFGEAGKRKFGIGPNADLVY
XP_008589828B      PIGIDKALEKMQREEQCILYLGPRY-GFGEAGKPKFGIEPNAELIY
XP_013052474B      PIGIDKALEKMQRGEHCVLYLGPRY-GFGEAGKPKYGIQANAELVY
KFP55426B          PIGIDKALEKMQRGEHCILYLGPRY-GFGEAGKPKYGIQANAELVY
KFP95835B          PIGIDKALEKMQRGEHCILYLGPRY-GFGEAGKPKYGIQANAELVY
XP_021233044B      PIGIDKALEKMQRGEHCILHLGPRY-GFGEAGKPKYGIQANAELVY
KFV71660B          PIGIDKALEKMQRAEHCILYLAPRY-GFGEAGKPKYGIQANAELVY
XP_014385088B      PIGIDKALEKMQREEQCILCLGPRY-GFGEAGKPKFGIEPNAELIY
KFU96756B          PIGIDKALEKMQRGEHCVLHLGPRY-GFGEAGKPKYGIPGNAELVY
XP_023576318B      PIGIDKAMEKMQREEQCILYLGPRY-GFGEAGKPKFGIEPNAELIY
XP_023576325B      PIGIDKAMEKMQREEQCILYLGPRY-GFGEAGKPKFGIEPNAELIY
KFM03271B          PIGIDKALEKMQRGEHCVLYLGPRY-GFGEAGKPKYGIQANAELVY
XP_014352291B      PIGIDKALEKMQRGESCLLHLKPQY-GFGDTGKPEFNIEPNVELDY
XP_015197960B      PLGVDRAMEKMQKGECCLLYLKPKY-GFGKEGKPEFSIEPNAELVY
XP_015197959B      PLGVDRAMEKMQKGECCLLYLKPKY-GFGKEGKPEFSIEPNAELVY
XP_017334936B      PLGVDRAMEKMQKGECCLLYLKPKY-GYGKEGKPELDIGPNAELLY
XP_017334935B      PLGVDRAMEKMQKGECCLLYLKPKY-GYGKEGKPELDIGPNAELLY
XP_017334933B      PLGVDRAMEKMQKGECCLLYLKPKY-GYGKEGKPELDIGPNAELLY
```

```
XP_020371994B       PIGVDKALEKMRRGEHCILHLMPEY-GFGDLGKPHLNVPPNAELDY
XP_023669222B       PLGVDRAMEKMQKGECCLLYLKPKY-GFGKEGRPKMDVGPNAELVY
XP_017541827B       PLGVDRAMEKMQKGECCLLYLKPKY-GYGKEGKPEYDIGPNAELLY
XP_016372735B       PLGVDRAMEKIQKGECCLLYLKPKY-GYGKEGKKEYDIGSNAELLY
XP_016322567B       PLGVDRAMEKIQKGECCLLYLKPKY-GYGKEGKKEYDIGSNAGLLY
XP_018602419B       PLGVDRAMEKMQEGECCLLYLKPKY-GFGKEGCPNLNIGPNAELMY
XP_019805261B       PIGIDKALEKMQREEQCILYLGPRY-GFGEAGKPEFGIEPNAELIY
XP_013987820B       PLGVDRAMEKMQKGECCMLYLKPKY-GFGKEGRAQYKIGPNSDLVY
XP_023843348B       PLGVDRAMEKMQKGECCMLYLKPKY-GFGKEGRAHYKIGPNSDLVY
XP_021332470B       PLGVDRAMEKLQKGECCLLYLKPKY-AFGKEGRKEHDIGSNAELLY
XP_005166078B       PLGVDRAMEKLQKGECCLLYLKPKY-AFGKEGRKEHDIGSNAELLY
XP_021424491B       PLGVDRAMEKMQKGECCMLYLKPKY-GFGKEGRAQYKIGPNSDLVY
NP_998314B          PLGVDRAMEKLQKGECCLLYLKPKY-AFGKEGRKEHDIGSNAELLY
XP_016314457B       PLGVDRAMENIQKGECCLLYLKPKY-GFGKEGKKEYDIGSNAELLY
XP_024283464B       PLGVDRAMEKMQKGECCMLYLKPKY-GFGKDGRAQHKIGPNSDLMY
XP_012670459B       PLGVDRSMEKMQKGECCLLYLKPKY-GFGTEGKQQYAIGPNANVVY
XP_021465979B       PLGVDRAMEKMQKGECCMLYLKPKY-GFGKDGRAQHKIGPNSDLMY
XP_020339278B       PLGVDRAMEKMQKGECCMLYLKPKY-GFGKDGRAQHKIGPNSDLMY
XP_005142250B       PIGIDKALEKMQRGEHCILYLAPRY-GFGEAGKPKYGIQGNMELVY
XP_024229852B       PLGVDRAMEKMQKGECCMLYLKPKY-GFGKEGRAQYKIGPNSDLVY
XP_023852739B       PLGVDRAMEKMQKGECCMLYLKPKY-GFGKDGRAQHKIGPNSDLMY
XP_020342285B       PLGVDRAMEKMQKGECCMLYLKPKY-GFGKEGRAQYKIGPNSDLVY
XP_016108165B       PLGVDRAMENIQKGECCLLYLKPKY-GFGKEGKKEYDIGSNAELLY
XP_023270066B       PLGVDRAMDKMQRGESCELHLKPKY-GFGSEGRPEYKIGPDKDIVY
XP_018541261B       PLGVDRAMDKMQRGECCVLYLKPNY-GFGSEGKAEYKIGPDKDIVY
XP_007899824B       PIGVDKAVEKMQRGEQCLVHLKSKY-GFGEKGRPEFNIPPNCELDY
XP_022602390B       PLGVDRAMDKMQRGESCELHLKPKY-GFGSEGRPEYKIGPDKDIVY
XP_020507498B       PLGVDRAMDKMQKGECCLLYLQPKY-GFGSEGKPEYKIGPDKEIVY
XP_022602392B       PLGVDRAMDKMQRGESCELHLKPKY-GFGSEGRPEYKIGPDKDIVY
XP_022602391B       PLGVDRAMDKMQRGESCELHLKPKY-GFGSEGRPEYKIGPDKDIVY
XP_016384571B       PLGVDRAMENIQKGECCLLYLKPKY-GFGKEGKKEYDIGSNAELLY
XP_007937638B       PIGIDKALEKMQREEQCILYLGPRY-GFGEAGKPKFGIEPNADLIY
XP_009894963B       PHGLEKAIQKMEKSEESVFYLKPNY-GFGSAGKEKFQIPPDAELQY
XP_014843613B       PLGVDRAMDKMQRGECCLLYLKPKY-GFGSEGKPEYKVGPDKEIVY
XP_020789713B       PLGVDRAMDKMQKSERCVLHLQPKY-GFGSEGQPEFKIGPDKELEY
XP_006783146B       PLGVDRAMDKMQKGECCLLYLKPKY-GFGSEGKPEYKIGPDKDIVY
XP_004548817B       PLGVDRAMDKMQKGECCLLYLKPKY-GFGSEGKPEYKIGPDKDIVY
XP_003448395B       PLGVDRAMDKMQKGECCLLYLKPKY-GFGSEGKPEYKIGPDKDIVY
KFV64423B           PHGLEKAIQKMEKSEESVFYLKPNY-GFGSAGKEKFQIPPDAELQY
XP_007903293B       PVGIEQALQSMEKGEDAMISLKPKY-GFGEAGKSKFSIPANASLVY
XP_005927577B       PLGVDRAMDKMQKGECCLLYLKPKY-GFGSEGKPEYKIGPDKDIVY
XP_006265443B       PPGLEKTIQRMEKLEESVIYLKPSY-GFGSAGKEKFQIPPDAELQY
XP_014795643B       PHGLEKAIQKMEKSEESVFYLKPNY-GFGSAGKEKFQIPPDAELQY
XP_006035184B       PPGLEKTIQRMEKLEESVIYLKPSY-GFGSAGKEKFQIPPDAELQY
XP_021177252B       PLGVDRAMDKMQRGECCLLYLKPKY-GFGSEGKSEYKVGPDKDIVY
XP_005724522B       PLGVDRAMDKMQKGECCLLYLKPKY-GFGSEGKPEYKIGPDKDIVY
XP_007551477B       PLGVDRAMDKMQRGECCLLYLKPKY-GFGSEGKPEYKVGPDKEIVY
XP_017160280B       PLGVDRAMDKMQRGECCLLYLKPKY-GFGNEGKPEYKVGPDKEIVY
XP_008406990B       PLGVDRAMDKMQRGECCLLYLKPKY-GFGNEGKPEYKVGPDKEIVY
XP_010573701B       PHGLEKAIQKMEKSEESVFYLKPNY-GFGSAGKEKFQIPPDAELQY
XP_022531443B       PLGVDRAMEKMQKGECCLLYLKPRY-GFGKEGKAEYGIGPNAELLY
XP_005810495B       PLGVDRAMDKMQRGECCLLYLKPKY-GFGNEGKPEYKVGPDKDIVY
XP_022044943B       PLGVDRALDKMQRGECCILYLKPKY-GFGSEGKPEYKIGPDKDVVY
XP_014897893B       PLGVDRAMDKMQRGECCLLYLKPKY-GFGSEGKPEYKVGPDKEIVY
XP_023136030B       PLGVDRALDKMQKGECCVLYLKPKY-GFGSEGKPEYNIGPDKDVVY
OPJ69275B           PHGLEKAIQKMEKLEESVFYLKPNY-GFGSSGKEKFQIPPDVELQY
XP_008109533B       PPGLDKALQKMEKLEECVIYLKPSY-GFGSAGKQKFQIPPDAELQY
XP_014349043B       PPGIEMAIQKMEKGEESIFYLKPKY-GFGASGHEKFNIPPDANLQY
XP_008283913B       PLGVDRALDKMQRGECCLLYLKPKY-GFGSEGKPECKIGPDKDVVY
XP_006633816B       PPGVEKALQSMEKGEESLITLSPKY-GFGTAGNSQFNIPPDAMLNY
XP_008071742B       PIGIDKALEKMQREEQCILYLGPRY-GFGEAGKPKFGIEP------
XP_006035186B       PPGLEKTIQRMEKLEESVIYLKPSY-GFGSAGKEKFQIPPDAELQY
XP_019964054B       PLGVDRAMDKMQRGECCVLYLKPKY-GFGSEGKSEYKVGPDKDIVY
XP_017685573B       PHGLEKAIQKMEKSEESVFYLKPSY-GFGSTGNEKFQIPPDAELQY
XP_005531080B       PHGLEKAIQKMEKSEESVFYLKPSY-GFGSAGNEKFKIPPDAELQY
XP_015814339B       PLGVDRAMDKMQKGECCLLQLKPKY-GFGNEGKPEFKIGPDKEIAY
XP_013811689B       PHGLEKAIQKMEKLEESVFYLKPNY-GFGSAGKEKFHIPPDAELQY
XP_021249927B       PHGLEKAIQKMEKSEESIFYLKPSY-GFGSTGKEKFQIPPDAELQY
                    * *:: ::  :.. * . . : . * . *  *     :
```
