# Supplementary material for: Compression of Large Sets of Sequence Data Reveals Fine Diversification of Functional Profiles in Multigene Families of Proteins: A Study for Peptidyl-Prolyl cis/trans Isomerases (PPIase)
Source: Biomolecules. 2019 Feb 11;9(2):59. doi: 10.3390/biom9020059 (PMC6406343; doi:10.3390/biom9020059)
Supplement: Supplementary file 1 [file biomolecules-09-00059-s001.zip › Figure S7 - Cyclophilins -Simpson PDI.pdf]

	PPIA	PPIB	PPIC	PPIF	PPIL3A	PPIL1	PPID	PPWD1	PPIL2	RANBP	PPIH	PPIG	NKTR	PPIE	PPIL4	CWC27	PPIL6	Structure	
1	0,185	0,086	0,324	0,745	-	-	0,788	0,188	-	0,048	0,676	0,087	0,036	0,628	-	0,033	0,445		
2	0,188	0,155	0,38	0,261	-	-	0,414	0,16	-	0	0,052	0,009	0,053	0,004	-	0,101	0,038		
3	0,143	0,64	0,489	0,196	-	-	0,15	0,241	-	0,012	0,029	0,104	0,053	0,018	-	0,052	0,617		
4	0,374	0,166	0,467	0,465	-	-	0,141	0,201	0,07	0,154	0,01	0,371	0,049	0,035	-	0,051	0,043	β	
5	0,061	0	0	0,152	-	-	0,332	0,039	0,049	0	0,014	0,104	0,053	0,004	-	0,101	0	β	
6	0,118	0,025	0	0,196	-	-	0,101	0,004	0,497	0	0,014	0,082	0,52	0	-	0,1	0	β	
7	0,067	0,006	0,039	0,509	-	0,296	0,426	0,182	0,158	0,217	0,131	0,082	0,027	0,276	-	0,461	0,047	β	1
8	0,026	0,013	0	0,168	0,299	0,649	0,156	0,073	0,083	0,215	0,019	0	0,014	0	-	0,078	0,01	β	
9	0,067	0,013	0	0,159	0,503	0,676	0,132	0,11	0,031	0	0,005	0,016	0,009	0	0,042	0,042	0,005	β	
10	0,37	0,429	0,304	0,64	0,005	0,038	0,544	0,048	0,016	0,592	0,221	0,609	0,027	0,254	0,418	0	0,744	β	
11	0,101	0,17	0	0,149	0,029	0,574	0,107	0,089	0,113	0,008	0,019	0,004	0,027	0	0,061	0,009	0,043	τ	
12	0,117	0,006	0,097	0,234	0,038	0,292	0,296	0	0,053	0,004	0,14	0,352	0,031	0,033	0,076	0	0,481	τ	
13	0,347	0,193	0,247	0,635	0,088	0,097	0,348	0,004	0,707	0,494	0,642	0,489	0,031	0,3	0,056	0,057	0,802	τ	
14	0,295	0,203	0,061	0,357	0,01	0,04	0,413	0	0,019	0,029	0,547	0,532	0,022	0,244	0,138	0	0,682	τ	
15	0,197	0,404	0	0,323	0,019	0,068	0,609	0,073	0,349	0,111	0,497	0,259	0,014	0,35	0,081	0,009	0,44	β	
16	0,114	0,05	0	0,176	0,083	0,048	0,342	0,044	0,648	0,004	0,005	0,008	0	0,021	0,09	0,057	0,143	β	
17	0,223	0	0	0,273	0,024	0	0,004	0	0	0	0,005	0,004	0	0,004	0,094	0	0,038	β	
18	0,294	0,062	0	0,221	-	0,693	0,008	0	0,515	0,189	0,074	0	0	0,025	0,095	0,004	0,174	β	2
19	0,062	0	0	0,185	0,038	0,36	0,012	0	0	0	0,009	0,004	0	0,004	0,114	0	0,024	β	
20	0,137	0,218	0,061	0,481	0,033	0,608	0,093	0,091	0,066	0,113	0,38	0,02	0	0,567	0,346	0,018	0,098	β	
21	0,256	0,262	0	0,526	0,024	0,104	0,47	0,236	0,05	0	0,355	0,22	0,009	0,234	0,374	0	0,175	β	
22	0,201	0	0,008	0,099	0,162	0	0,012	0,082	0	0,008	0,005	0,008	0,009	0,072	0,114	0	0,023	β	
23	0,022	0,006	0	0	0,024	0	0	0	0	0	0,005	0,004	0,004	0	0,076	0	0	β	
24	0,034	0,013	0	0,348	0,009	0,002	0	0,004	0,57	0,052	0,009	0,012	0,004	0,008	0,071	0	0,005	α	
25	0,061	0,006	0,016	0,056	0,083	0,529	0,43	0,141	0,416	0,22	0,216	0,144	0,004	0,446	0,179	0,158	0,518	α	
26	0,033	0,119	0,225	0	0,028	0,625	0,059	0,215	0,077	0,235	0,009	0,004	0,004	0	0,409	0	0,014	α	1
27	0,163	0,131	0	0	0,024	0,041	0,052	0,004	0,659	0	0,126	0,028	0,004	0	0,369	0	0,313	α	
28	0,195	0,025	0	0,008	0,683	0,085	0,136	0,035	0,491	0	0,233	0,04	0,004	0,008	0,189	0,293	0,093	α	
29	0,039	0,006	0	0	0,663	0,002	0,004	0,018	0,037	0,016	0,009	0,004	0	0	0,269	0	0,005	α	
30	0,033	0,013	0,209	0,008	0,069	0,316	0,004	0,004	0,032	0,383	0,036	0,136	0,004	0,281	0,103	0,069	0,019	α	
31	0,065	0	0	0	0,065	0,035	0,004	0,022	0,49	0	0,032	0	0	0	0,422	0,026	0	α	
32	0,06	0,124	0	0,024	0,418	0,041	0,028	0	0,455	0,032	0,26	0,177	0,268	0,041	0,549	0,04	0,224	α	
33	0,022	0,169	0,105	0,008	0,339	0,335	0,016	0	0,429	0	0,014	0,37	0,022	0	0,393	0,004	0,517	α	
34	0,065	0	0	0	0,097	0,008	0,016	0,004	0	0,004	0	0	0	0	0,349	0	0	α	2
35	0,054	0	0,008	0,008	0,027	0,035	0	0	0,002	0	0,018	0	0	0	0,005	0	0	α	
36	0,175	0,349	0	0,008	0,7	0,414	0,016	0,069	0,081	0	0,005	0,369	0	0,004	0	0,031	0,466	α	
37	0,038	0,408	0,154	0,008	0,4	0,431	0,067	0,044	0,434	0	0,028	0,2	0,088	0,199	0	0,004	0,347	α	
38	0,033	0	0	0	0,7	0	0	0	0,313	0	0,322	0	0	0	0	0	0	α	
39	0,128	0,067	0	0	0,023	0,467	0,016	0,048	0,442	0	0,018	0	0	0	0	0,044	0,014	α	
40	0,033	0,257	0,023	0	0,005	0,4	0,016	0,018	0,501	0	0,018	0,012	0,004	0,029	0	0,035	0,287	α	
41	0,065	0,199	0	0	0,018	0,054	0	0,009	0,438	0	0,018	0,012	0	0,045	0,005	0,022	0,005	τ	
42	0,017	0,219	0,008	0	0,023	0,137	0	0,031	0,555	0,004	0,005	0	0	0	0,101	0,65	0,682	τ	
43	0,011	0,149	0,031	0,071	0,093	-	0,032	-	-	0,052	0,149	0,028	0,009	0,004	0,009	-	0,413	τ	
44	0,038	0,098	0	0	0,067	-	0	-	-	0	0,043	0,008	0,013	0,008	-	-	0,437	τ	
45	-	-	-	-	-	-	0,45	-	-	-	0,137	0,499	0,221	-	-	-	0,673		
46	-	-	-	-	-	-	0,02	-	-	-	-	0,004	0,013	-	-	-	0,411		
47	-	-	-	-	-	-	0,538	-	-	-	-	0,036	0	-	-	-	0,504		
48	-	-	-	-	-	-	0,487	-	-	-	-	0,353	0,236	-	-	-	-		
49	-	-	-	-	-	-	0,162	-	-	-	0,429	0	0	-	-	-	0,584		
50	-	-	-	-	-	-	0,036	-	-	-	0,086	0,459	0,089	-	-	-	0,236		
51	-	-	-	-	-	-	0,189	-	-	-	0,136	0	0	-	-	-	0,507		
52	-	-	-	-	0,089	-	0,052	-	-	-	0,019	0,409	0,03	-	-	-	0,631		
53	0,033	0,236	0,008	0,016	0,478	-	0,004	-	-	0,008	0,21	0	0	0	-	-	0,014		
54	0,138	0,074	0,038	0,008	0,014	0,004	0,134	0	0,025	0,016	0,009	0,375	0,022	0	0,045	0	0,568		
55	0,028	0,006	0	0	0,032	0	0,02	0	0	0,188	0,005	0,004	0	0	0	0	0	β	
56	0,022	0,225	0,046	0,016	0,227	0,082	0	0	0,347	0,092	0,009	0,004	0,004	0,24	0	0,093	0,416	β	
57	0,102	0,509	0	0	0,36	0,576	0,024	0,135	0,452	0,17	0,307	0,528	0	0,012	0,208	0,353	0,533	β	
58	0,038	0,006	0	0,008	0,331	0,525	0,436	0,031	0,446	0,012	0,631	0,501	0	0,012	0,436	0,026	0,032	β	
59	0,312	0,197	0,342	0,236	0,186	0,537	0,533	0,492	0,521	0,514	0,635	0,406	0	0,065	0,135	0,126	0,115	β	3
60	0,06	0	0	0,008	0,067	0,071	0	0,06	0,009	0,004	0,005	0,028	0	0,064	0,486	0	0,122	β	
61	0,028	0	0	0	0,162	0	0	0	0,005	0,04	0	0,008	0	0	0,329	0,004	0,009	β	
62	0,022	0	0	0	0,032	0,002	0,004	0	0,002	0	0,005	0,004	0	0	0,496	0	0,028	β	
63	0,028	0	0	0	0,036	0,002	0,004	0,009	0,541	0,004	0,005	0	0	0	0	0	0,028	β	
64	0,011	0	0	0	0,032	0	0,012	0	0,002	0,004	0,014	0	0	0	0,063	0,013	0,005		
65	0,06	0,121	0	0,008	0,105	0,619	0,051	0	0,081	0,406	0,005	0,004	0	0	0,376	0,354	0,635		
66	0,112	0,025	0	0,508	0,05	0,07	0,644	0,106	0,305	0,134	0,027	0,032	0,043	0,025	0,058	0,304	0,18	β	

67	0,017	0	0	0,008	0	0,017	0	0	0,002	0,025	0	0	0	0	0,079	0,004	0,144	β	
68	0,017	0	0	0	0,032	0,002	0,004	0	0,002	0,02	0,005	0	0	0	0,032	0,013	0	β	4
69	0,028	0,013	0	0,016	0,041	0,006	0,028	0	0,005	0,004	0,018	0,004	0	0,037	0,036	0	0	β	
70	0,017	0	0	0,008	0,027	0,004	0,008	0	0,005	0,044	0	0,004	0	0	0	0	0,005		
71	0,038	0	0	0,491	0	0,313	0,024	0,004	0,021	0,004	0,397	0,032	0	0,325	0,128	0	0,046		
72	0,055	0	0	0	0,209	0,002	0,004	0	0,002	0	0,005	0	0	0	0	0	0,005		
73	0,028	0	0	0	0,032	0	0,004	0,018	0	0	0,005	0	0	0	0	0	0		
74	0,049	0	0,22	0	0,027	0,008	0,206	0	0	0	0,058	0,004	0	0,008	0	0	0,01		
75	0,055	0,019	0,008	0,008	0,036	0,002	0,02	0,123	0,025	0,004	0,032	0	0	0	0,362	0,213	0,498		
76	0,353	0,137	0,41	0,04	0,32	0,058	0,438	0	0,14	0,462	0,483	0,143	0	0,037	0,233	0,017	0,781		
77	0,049	0,037	0,09	0,016	0,416	-	0,587	-	-	0,36	0,163	0,004	0	0,053	-	-	0,33		
78	0,06	0	0,008	0	0,018	0,002	0,012	0,138	0,041	0	0	0,012	0,035	0,012	0,045	0,209	0,27		
79	0,044	0	0	0	0,009	0	0,004	0	0,002	0	0	0	0	0	0,014	0	0		
80	0,044	0,019	0,031	0	0	0,01	0,04	0,208	0,628	0,004	0,023	0,16	0	0	0,147	0,357	0,106		
81	0,033	0	0	0	0,132	0,004	0,004	0,004	0	0	0	0	0	0,004	0,027	0,004	0,527	τ	
82	0,107	0	0	0	0,005	0,002	0,004	0	0	0,016	0,512	0	0	0	0	0	0,005	τ	
83	0,011	0	0,544	0,016	0,572	0,645	0,004	0,009	0,319	0,39	0,496	0,004	0	0,004	0,124	0,004	0,019	τ	
84	0,065	0,006	0	0,008	0	0,004	0,008	0,004	0,002	0	0,032	0,004	0	0	0,023	0,004	0,01	τ	
85	0,027	0	0,008	0	0,009	0,002	0	0	0,549	0	0,005	0	0	0	0,014	0,004	0		
86	0,028	0	0	0,008	0,005	0,006	0,004	0	0	0	0,005	0,004	0	0	0,014	0	0,024		
87	0,127	0,006	0	0,008	0,263	0,006	0,008	0	0,197	0,004	0,522	0,008	0	0,004	0,671	0,022	0,005		
88	-	-	-	-	-	-	-	-	-	-	-	-	-	-	0,672	-	-		
89	-	-	-	-	-	-	-	-	-	-	-	-	-	-	0,005	-	-		
90	-	-	-	-	-	-	-	-	-	-	-	-	-	-	0,278	-	-		
91	-	-	-	-	-	-	-	-	-	-	-	-	-	-	0,005	-	-		
92	-	-	-	-	-	-	-	-	-	-	-	-	-	-	0,085	-	-		
93	-	-	-	-	-	-	-	-	-	-	-	-	-	-	0,246	-	-		
94	-	-	-	-	-	-	-	-	-	-	-	-	-	-	0,124	-	-		
95	0,097	0,05	0,247	0,415	0,302	0,672	0,304	0,018	0,165	0,532	0,74	0,012	0,004	0,209	0,402	0,559	0,176		
96	0,028	0,006	0,546	0,071	0,093	0,579	0,055	0	0,557	0,578	0,693	0,175	0	0,004	0,023	0,047	0,259		
97	0,033	0	0	0	0,005	0,002	0,004	0	0,009	0	0,014	0,008	0	0	0,027	0	0		
98	0,332	0,385	0,199	0,131	0,102	0,329	0,108	0,031	0,614	0,008	0,677	0,379	0,267	0,025	0,084	0,026	0,033		
99	0,006	0,006	0	0	0	0	0,004	0	0	0	0,005	0	0	0	0,335	0	0,006		
100	0,022	0,006	0	0	0	0	0,004	0	0	0	0	0,004	0	0	0	0	0,023		
101	-	-	-	-	0,32	0,029	-	0,031	0,282	-	-	-	-	-	0,553	0,035	-		
102	0,044	0	0,008	0,008	0,547	0,526	0,156	0,026	0,499	0,068	0,153	0,465	0	0	0,342	0	0,461		
103	0,096	0	0	0	0,152	0,598	0,036	0,377	0,606	0	0,005	0,004	0,009	0	0,081	0,056	0,018		
104	0,071	0,031	0,016	0,69	0,739	0,396	0,621	0,146	0,541	0,163	0,723	0,359	0,197	0,266	0,218	0	0,493		
105	0,07	0,006	0	0	0,023	0	0,47	0	0,212	0	0,102	0,024	0	0	0,005	0	0,028		
106	0,065	0,086	0,023	0,048	0,005	0,013	0,119	0,004	0,48	0	0,102	0,051	0	0	0,138	0,004	0,598		
107	0,038	0	0	0	0	0,418	0	0	0	0	0,023	0,004	0	0	0	0,004	0,029		
108	0,044	0,219	0,21	0,569	0,504	0,087	0,067	0	0,506	0,008	0,424	0,051	0	0,025	0,502	0,42	0,537		
109	0,055	0,135	0,008	0,008	0,368	0	0,222	0,013	0,507	0,141	0,674	0,024	0,004	0,251	0,067	0	0,311		
110	0,106	0,405	0,046	0,04	0	0,008	0,685	0,009	0	0	0,166	0,492	0,013	0,1	0,468	0	0,018		
111	0,117	0,086	0,068	0	0,005	0,002	0,31	0	0	0	0,005	0,012	0	0	0	0	0	β	
112	0,022	0,2	0	0	0,076	0	0,016	0	0,146	0,024	0,05	0	0	0,374	0,448	0	0,101	β	
113	0,022	0	0	0	0	0,002	0	0,009	0	0	0,005	0	0	0	0	0	0	β	
114	0,033	0	0	0	0,088	0,418	0,004	0	0,009	0,008	0,045	0,004	0	0	0,097	0,123	0,009	β	5
115	0,038	0	0	0	0	0,004	0,004	0	0	0	0,014	0	0	0	0,1	0,022	0,005	β	
116	0,017	0	0	0	0	0	0	0	0	0	0,005	0	0	0	0	0	0,009	β	
117	0,033	0,006	0	0	0,027	0,002	0	0	0,002	0,04	0	0	0	0,004	0,163	0,009	0,009	β	
118	0,017	0,108	0,487	0	0,344	0,501	0,28	0,093	0,467	0,252	0,1	0,008	0	0,012	0,366	0,22	0,178		
119	0,028	0	0	0	0,005	0,002	0,004	0	0	0	0	0	0	0,004	0,076	0,022	0		
120	0,055	0,138	0,008	0,016	0,05	0,087	0,459	0,551	0,508	0,423	0,471	0,02	0,004	0,2	0,554	0,545	0,366		
121	0,028	0,006	0,023	0	0,005	0,025	0,473	0,235	0,162	0,004	0,506	0,254	0,258	0,004	0,213	0,38	0,005		
122	0,038	0	0	0	0,116	0,008	0	0	0,041	0,024	0,005	0	0	0	0,507	0,009	0		
123	0,028	0	0	0	0,009	0	0	0	0,002	0	0	0	0	0,008	0,44	0	0,005		
124	0,152	0	0	0	0,328	0,048	0,008	0	0,647	0,137	0	0	0	0,021	0,121	0,13	0,275		
125	0,038	0	0	0	0,005	0	0	0,004	0,002	0	0,071	0	0	0	0	0	0		
126	0,049	0	0	0	0	0,002	0	0	0	0	0	0	0	0	0,014	0	0	β	
127	0,055	0	0	0	0	0	0	0	0	0	0,005	0	0	0	0	0	0,005	β	
128	0,044	0	0	0	0	0	0	0	0	0,052	0,005	0	0	0	0,474	0	0	β	6
129	0,044	0	0	0	0,244	0,029	0	0,004	0,044	0	0,009	0	0	0,004	0,058	0,056	0	β	
130	0,017	0,025	0,164	0,032	0,058	0,008	0,024	0,004	0,242	0,068	0	0,024	0	0,276	0,009	0	0,009	β	
131	0,065	0,061	0,097	0	0,005	0,017	0,067	0	0,005	0	0,023	0,004	0	0,432	0,471	0,123	0,089		
132	0,282	0,169	0,125	0,277	0,561	0,208	0,254	0,026	0,048	0,033	0,53	0,161	0	0,264	0,404	0,282	0,032		

133	0,038	0,489	0	0,048	0,482	0,008	0,291	0	0,508	0,008	0,186	0,176	0,288	0,017	0,234	0,404	0,53		
134	-	-	-	-	-	-	-	-	-	-	-	-	-	-	0,119	-	-		
135	0,06	0,019	0,274	0,016	0,124	0,288	0,044	0,052	0,446	0,052	0,084	0,46	0	0	0,058	0,365	0,504	α	
136	0,038	0,652	0,282	0,071	0,173	0,505	0,401	0	0,719	0	0,009	0,008	0,004	0,008	0,187	0,363	0,264	α	
137	0,076	0	0	0,008	0,232	0,271	0,024	0	0,554	0,13	0,005	0	0	0	0,241	0,013	0,018	α	3
138	0,011	0,006	0,006	0	0	0	0	0	0,002	0	0	0	0	0	0,005	0	0	α	
139	0,038	0	0	0	0	0,002	0	0	0,002	0	0,005	0	0	0	0,005	0,013	0	α	
140	0,033	0,013	0,013	0,085	0,259	0,216	0,112	0,009	0,528	0,064	0,525	0,132	0,018	0,087	0,303	0,106	0,196	α	
141	0,033	0	0	0	0,112	0,029	0	0	0,028	0	0,005	0,509	0	0,012	0,501	0,056	0,305		
142	0,081	0	0	0	0,005	0,002	0,004	0	0,005	0	0,027	0,004	0,004	0	0,054	0,127	0,12		
143	0,011	0	0	0	0,005	0,094	0,004	0	0,05	0	0,009	0,016	0	0,004	0,27	0	0,005	β	
144	0,017	0,006	0,006	0	0,005	0	0,004	0	0	0	0,005	0	0	0	0	0	0,014	β	
145	0,065	0	0	0,008	0,023	0,002	0	0	0,002	0	0,005	0	0	0	0,005	0,009	0,005	β	
146	0,033	0	0	0	0,071	0,002	0,004	0	0,059	0,004	0	0	0	0	0,014	0,039	0,005	β	7
147	0,184	0,17	0,17	0,436	0,005	0,004	0,336	0	0,082	0,004	0,041	0,497	0,004	0,279	0,072	0	0,161	β	
148	0,011	0	0	0	0,005	0	0	0,196	0,002	0	0	0	0	0	0	0	0,056	β	
149	0,033	0,013	0,013	0,146	0,005	0,753	0,02	0,202	0,009	0,016	0,005	0,004	0	0,44	0,292	0,165	0,089	β	
150	-	-	-	-	-	-	-	-	-	-	-	-	-	-	-	0,018	-		
151	0,039	0,16	0,16	0,008	0,164	0,664	0,272	0,013	0,215	0,008	0,034	0,036	0	0,257	0	0,031	0,071	β	
152	0,134	0	0	0	0,005	0,004	0,004	0	0,157	0	0,089	0	0	0	0	0,004	0,066	α	
153	0,06	0,149	0,149	0,048	0,442	0	0,328	0	0,625	0	0,036	0,398	0	0,012	0,31	0,004	0,297	α	
154	0,112	0,062	0,062	0,008	0	0,761	0,327	0	0,265	0,064	0,018	0,012	0	0	0	0,009	0,206	α	
155	0,07	0	0	0	0,381	0,133	0,016	0,018	0,414	0,501	0,183	0,004	0	0,004	0,175	0,004	0,079	α	
156	0,017	0	0	0	0	0,01	0,059	0,031	0,002	0	0,005	0	0	0	0	0,004	0,033	α	5
157	0,138	0,284	0,284	0,048	0	0,547	0,02	0,009	0,579	0,152	0,009	0,458	0,276	0,077	0,683	0,022	0,54	α	
158	0,168	0,109	0,109	0,063	0,263	0,047	0,544	0,18	0,572	0,162	0,014	0,42	0,249	0,494	0,363	0,004	0,552	α	
159	0,055	0,013	0,013	0	0,018	0	0,02	0	0,016	0	0,005	0	0	0,008	0,178	0,018	0,01	α	
160	0,033	0	0	0	0	0,096	0	0,082	0,002	0,252	0,005	0,004	0	0,008	0,018	0,641	0,056	α	
161	0,367	0,668	0,668	0,168	0,018	0,339	0,467	0,131	0,627	0,258	0,094	0,375	0,274	0,344	0,041	0,004	0,559		
162	0,277	0,195	0,195	0,164	0,119	0,006	0,153	0,052	0,135	0,257	0,123	0,432	0,004	0,388	0,488	0,282	0,221		
163	0,05	0,354	0,354	0	0,288	0,615	0,437	0	0,459	0,004	0,52	0,146	0	0,098	0,42	0,319	0,4		
164	0,133	0,138	0,138	0,008	0,154	0,155	0,496	0,202	0,492	0,02	0,21	0,165	0,004	0,205	0,475	0,266	0,075	τ	
165	-	0,344	0,344	-	0,224	0,301	-	0,082	0,087	-	0,18	0	0	-	0,014	0,19	-		
166	0,06	0,71	0,71	0,129	0,487	0,798	0,539	0,057	0,591	0,279	0,261	0,543	0,5	0,068	0,508	0,6	0,664	τ	
167	0,118	0,388	0,388	0,178	0,166	0,242	0,488	0,004	0,549	0,432	0,117	0,588	0	0,403	0,152	0,018	0,111	τ	
168	-	-	-	-	0,573	-	-	0,039	0,37	-	-	-	-	-	-	-	-		
169	0,118	0,049	0,049	0	0,277	0,002	0,072	0	0,048	0	0,101	0,032	0	0,102	0,581	0,004	0,08	τ	
170	0,028	0,043	0,043	0,04	0,018	0,054	0,644	0,004	0,07	0,586	0,06	0	0	0,326	0,506	0,013	0,071		
171	0,112	0,006	0,006	0,273	0,014	0,021	0,049	0,004	0,002	0,254	0,038	0,004	0	0,255	0	0	0,071		
172	0,087	0,33	0,33	0,094	0,193	0,368	0,342	0,065	0,614	0,625	0,037	0,207	0	0,378	0,058	0,571	0,62	β	
173	0,039	0,289	0,289	0,048	0,651	0,306	0,455	0	0,411	0,482	0,033	0,194	0	0,133	0,239	0,617	0,763	β	
174	0,076	0,177	0,177	0,008	0,041	0,223	0,409	0	0,222	0,088	0,004	0	0	0,083	0,009	0,123	0,63	β	
175	0,044	0,055	0,055	0,024	0,442	0,009	0,511	0	0,05	0,008	0,004	0	0	0,083	0	0,278	0,137	β	
176	0,066	0,551	0,551	0,32	0,041	0,131	0,099	0,173	0,584	0,51	0,02	0,063	0	0,161	0,032	0,022	0,577	β	
177	0,071	0,006	0,006	0,016	0,014	0,006	0,207	0	0,035	0,004	0,029	0	0	0,087	0,005	0,004	0,089	β	
178	0,45	0,359	0,359	0,199	0,54	0,298	0,106	0	0,642	0,147	0,099	0,063	0	0,481	0,583	0,412	0,54	β	8
179	0,033	0,215	0,215	0,056	0,582	0,168	0,463	0,18	0,704	0,033	0,207	0,507	0,004	0,277	0,009	0,363	0,389	β	
180	0,082	0,404	0,404	0,016	0,036	0,41	-	0,004	0,519	0,004	0,106	0,008	0	0,099	0,032	0,558	0,535	β	
181	0,066	0,074	0,074	0	0,072	0,712	-	0,013	0,641	0,004	0,463	0	0	0,052	0,089	0,088	0,112	β	
182	0,076	0,666	0,666	0,016	0,009	0,362	-	0,009	0,021	0,02	0,021	0,261	0,27	0,043	0,005	0	0,636	β	
183	0,129	0,125	0,125	0,016	0,018	0,712	-	0,009	0,055	0,029	0,009	0,004	0	0,542	0,005	0,005	0,61		
184	0,185	0,086	0,324	0,745	-	-	0,788	0,188	-	0,048	0,676	0,087	0,036	0,628	-	0,033	0,445		
