# Supplementary material for: Compression of Large Sets of Sequence Data Reveals Fine Diversification of Functional Profiles in Multigene Families of Proteins: A Study for Peptidyl-Prolyl cis/trans Isomerases (PPIase)
Source: Biomolecules. 2019 Feb 11;9(2):59. doi: 10.3390/biom9020059 (PMC6406343; doi:10.3390/biom9020059)
Supplement: Supplementary file 1 [file biomolecules-09-00059-s001.zip › Figure S8A-MSA of CyP_157s.aln.pdf]

CLUSTAL 2.0.9 multiple sequence alignment cyp157

```
NP_005376          DRPQCHFDIEINREPVGRIMFQLFSDICPKTCKNFLCLCSGEKGLGKTTGKKLCYKGSTF
XP_014646452       DRPQCHFDIEINREPVGRIMFQLFSDICPKTCKNFLCLCSGEKGLGKTTGKKLCYKGSTF
XP_017657797       DRPQCHFDIEINREPVGRIMFQLFSDICPKTCKNFLCLCSGEKGLGKTTGKKLCYKGSTF
XP_012885690       DRPQCHFDIEINREPVGRIMFQLFSDICPKTCKNFLCLCSGEKGRGKTTGKKLCYKGSTF
XP_008530969       DRPQCHFDIEINREPVGRIMFQLFSDICPKTCKNFLCLCSGEKGLGKTTGKKLCYKGSTF
XP_015423871       DRPQCHFDIEINREPVGRIMFQLFSDICPKTCKNFLCLCSGEKGLGKTTGKKLCYKGSTF
XP_015091833       DRPQCHFDIEINREPVGRIMFQLFSDICPKTCKNFLCLCSGEKGLGKTTGKKLCYKGSTF
XP_010973060       DRPQCHFDIEINREPVGRIMFQLFSDICPKTCKNFLCLCSGEKGLGKTTGKKLCYKGSTF
XP_019583096       DRPQCHFDIEINREPVGRIMFQLFSDICPKTCKNFLCLCSGEKGLGKTTGKKLCYKGSTF
XP_011231111       DRPQCHFDIEINREPVGRIMFQLFSDICPKTCKNFLCLCSGEKGLGKTTGKKLCYKGSTF
XP_014646457       DRPQCHFDIEINREPVGRIMFQLFSDICPKTCKNFLCLCSGEKGLGKTTGKKLCYKGSTF
XP_023988069       DRPQCHFDIEINREPVGRIMFQLFSDICPKTCKNFLCLCSGEKGLGKTTGKKLCYKGSTF
XP_008530970       DRPQCHFDIEINREPVGRIMFQLFSDICPKTCKNFLCLCSGEKGLGKTTGKKLCYKGSTF
XP_017497119       DRPQCHFDIEINREPVGRIMFQLFSDICPKTCKNFLCLCSGEKGLGKTTGKKLCYKGSTF
XP_021503072       DRPQCHFDIEINREPVGRIMFQLFSDICPKTCKNFLCLCSGEKGLGKTTGKKLCYKGSTF
XP_021503071       DRPQCHFDIEINREPVGRIMFQLFSDICPKTCKNFLCLCSGEKGLGKTTGKKLCYKGSTF
XP_020041828       DRPQCHFDIEINREPVGRIMFQLFSDICPKTCKNFLCLCSGEKGLGKTTGKKLCYKGSTF
XP_021503070       DRPQCHFDIEINREPVGRIMFQLFSDICPKTCKNFLCLCSGEKGLGKTTGKKLCYKGSTF
XP_012590780       DRPQCHFDIEINREPVGRIMFQLFSDICPKTCKNFLCLCSGEKGLGKTTGKKLCYKGSTF
XP_019274390       DRPQCHFDIEINREPVGRIMFQLFSDICPKTCKNFLCLCSGEKGLGKTTGKKLCYKGSTF
XP_019489071       DRPQCHFDIEINREPVGRIMFQLFSDICPKTCKNFLCLCSGEKGLGKTTGKKLCYKGSTF
XP_019489063       DRPQCHFDIEINREPVGRIMFQLFSDICPKTCKNFLCLCSGEKGLGKTTGKKLCYKGSTF
XP_010588206       DRPQCHFDIEINREPVGRIMFQLFSDICPKTCKNFLCLCSGEKGLGKTTGKKLCYKGSTF
XP_023403600       DRPQCHFDIEINREPVGRIMFQLFSDICPKTCKNFLCLCSGEKGLGKTTGKKLCYKGSTF
XP_009982730       DRPQCFFEIEINREPVGRIMFQLFSDICPKTCKNFLCLCSGEKGIGKTTGKKLCYKGTTF
XP_019839655       DRPQCHFDIEINREPVGRIMFQLFSDICPKTCKNFLCLCSGEKGLGKTTGKKLCYKGSTF
XP_006512034       DRPQCHFDIEINREPVGRIMFQLFSDICPKTCKNFLCLCSGEKGLGKTTGKKLCYKGSTF
XP_024100264       DRPQCHFDIEINREPVGRIMFQLFSDICPKTCKNFLCLCSGEKGLGKTTGKKLCYKGSTF
XP_019666422       DRPQCHFDIEINREPVGRIMFQLFSDICPKTCKNFLCLCSGEKGLGKTTGKKLCYKGSTF
XP_008530962       DRPQCHFDIEINREPVGRIMFQLFSDICPKTCKNFLCLCSGEKGLGKTTGKKLCYKGSTF
XP_008530967       DRPQCHFDIEINREPVGRIMFQLFSDICPKTCKNFLCLCSGEKGLGKTTGKKLCYKGSTF
XP_010944204       DRPQCHFDIEINREPVGRIMFQLFSDICPKTCKNFLCLCSGEKGLGKTTGKKLCYKGSTF
XP_015091830       DRPQCHFDIEINREPVGRIMFQLFSDICPKTCKNFLCLCSGEKGLGKTTGKKLCYKGSTF
XP_014411884       DRPQCHFDIEINREPVGRIMFQLFSDICPKTCKNFLCLCSGEKGLGKTTGKKLCYKGSTF
XP_010944201       DRPQCHFDIEINREPVGRIMFQLFSDICPKTCKNFLCLCSGEKGLGKTTGKKLCYKGSTF
XP_009443554       DRPQCHFDIEINREPVGRIMFQLFSDICPKTCKNFLCLCSGEKGLGKTTGKKLCYKGSTF
XP_021241242       DRPQCFFDIEINREPVGRIMFQLFSDICPKTCKNFLCLCSGEKGIGKTTGKKLCYKGTTF
XP_020927360       DRPQCHFDIEINREPVGRIMFQLFSDICPKTCKNFLCLCSGEKGLGKTTGKKLCYKGSTF
XP_004581770       DRPQCHFDIEINREPVGRIMFQLFSDICPKTCKNFLCLCSGEKGLGKTTGKKLCYKGSTF
XP_005669469       DRPQCHFDIEINREPVGRIMFQLFSDICPKTCKNFLCLCSGEKGLGKTTGKKLCYKGSTF
XP_009443555       DRPQCHFDIEINREPVGRIMFQLFSDICPKTCKNFLCLCSGEKGLGKTTGKKLCYKGSTF
XP_005669468       DRPQCHFDIEINREPVGRIMFQLFSDICPKTCKNFLCLCSGEKGLGKTTGKKLCYKGSTF
XP_008708042       DRPQCHFDIEINREPVGRIMFQLFSDICPKTCKNFLCLCSGEKGLGKTTGKKLCYKGSTF
AAA37500           DRPQCHFDIEINREPVGRIMFQLFSDICPKTCKNFLCLCSGEKGLGKTTGKKLCYKGSTF
XP_004676331       DRPQCHFDIEINREPVGRIMFQLFSDICPKTCKNFLCLCSGEKGLGKTTGKKLCYKGSTF
XP_006748020       DRPQCHFDIEINREPVGRIMFQLFSDICPKTCKNFLCLCSGEKGLGKTTGKKLCYKGSTF
XP_006770577       DRPQCHFDIEINREPVGRIMFQLFSDICPKTCKNFLCLCSGEKGLGKTTGKKLCYKGSTF
XP_019583095       DRPQCHFDIEINREPVGRIMFQLFSDICPKTCKNFLCLCSGEKGLGKTTGKKLCYKGSTF
XP_015423867       DRPQCHFDIEINREPVGRIMFQLFSDICPKTCKNFLCLCSGEKGLGKTTGKKLCYKGSTF
XP_005383594       DRPQCHFDIEINREPVGRIMFQLFSDICPKTCKNFLCLCSGEKGLGKTTGKKLCYKGSTF
XP_019583089       DRPQCHFDIEINREPVGRIMFQLFSDICPKTCKNFLCLCSGEKGLGKTTGKKLCYKGSTF
XP_014445953       DRPQCHFDIEINREPVGRIMFQLFSDICPKTCKNFLCLCSGEKGLGKTTGKKLCYKGSTF
XP_005891140       DRPQCHFDIEINREPVGRIMFQLFSDICPKTCKNFLCLCSGEKGLGKTTGKKLCYKGSTF
XP_019489104       DRPQCHFDIEINREPVGRIMFQLFSDICPKTCKNFLCLCSGEKGLGKTTGKKLCYKGSTF
XP_012004520       DRPQCHFDIEINREPVGRIMFQLFSDICPKTCKNFLCLCSGEKGLGKTTGKKLCYKGSTF
```

XP_006051863	DRPQCHFDIEINREPVGRIMFQLFSDICPKTCKNFLCLCSGEKGLGKTTGKKLCYKGSTF
XP_005954297	DRPQCHFDIEINREPVGRIMFQLFSDICPKTCKNFLCLCSGEKGLGKTTGKKLCYKGSTF
XP_005695639	DRPQCHFDIEINREPVGRIMFQLFSDICPKTCKNFLCLCSGEKGLGKTTGKKLCYKGSTF
XP_011954828	DRPQCHFDIEINREPVGRIMFQLFSDICPKTCKNFLCLCSGEKGLGKTTGKKLCYKGSTF
XP_003992255	DRPQCHFDIEINREPVGRIMFQLFSDICPKTCKNFLCLCSGEKGLGKTTGKKLCYKGSTF
XP_002702773	DRPQCHFDIEINREPVGRIMFQLFSDICPKTCKNFLCLCSGEKGLGKTTGKKLCYKGSTF
XP_019666421	DRPQCHFDIEINREPVGRIMFQLFSDICPKTCKNFLCLCSGEKGLGKTTGKKLCYKGSTF
XP_019489077	DRPQCHFDIEINREPVGRIMFQLFSDICPKTCKNFLCLCSGEKGLGKTTGKKLCYKGSTF
XP_012004518	DRPQCHFDIEINREPVGRIMFQLFSDICPKTCKNFLCLCSGEKGLGKTTGKKLCYKGSTF
XP_011954826	DRPQCHFDIEINREPVGRIMFQLFSDICPKTCKNFLCLCSGEKGLGKTTGKKLCYKGSTF
XP_006051860	DRPQCHFDIEINREPVGRIMFQLFSDICPKTCKNFLCLCSGEKGLGKTTGKKLCYKGSTF
XP_005695638	DRPQCHFDIEINREPVGRIMFQLFSDICPKTCKNFLCLCSGEKGLGKTTGKKLCYKGSTF
XP_005196454	DRPQCHFDIEINREPVGRIMFQLFSDICPKTCKNFLCLCSGEKGLGKTTGKKLCYKGSTF
XP_851976	DRPQCHFDIEINREPVGRIMFQLFSDICPKTCKNFLCLCSGEKGLGKTTGKKLCYKGSTF
XP_012520957	DRPQCHFDIEINREPVGRIMFQLFSDICPKTCKNFLCLCSGEKGLGKTTGKKLCYKGSTF
XP_005634360	DRPQCHFDIEINREPVGRIMFQLFSDICPKTCKNFLCLCSGEKGLGKTTGKKLCYKGSTF
XP_003132182	DRPQCHFDIEINREPVGRIMFQLFSDICPKTCKNFLCLCSGEKGLGKTTGKKLCYKGSTF
XP_020139644	DRPQCHFDIEINREPVGRIMFQLFSDICPKTCKNFLCLCSGEKGLGKTTGKKLCYKGSTF
XP_014715584	DRPQCHFDIEINREPVGRIMFQLFSDICPKTCKNFLCLCSGEKGLGKTTGKKLCYKGSTF
XP_007193513	DRPQCHFDIEINREPVGRIMFQLFSDICPKTCKNFLCLCSGEKGLGKTTGKKLCYKGSTF
XP_005669463	DRPQCHFDIEINREPVGRIMFQLFSDICPKTCKNFLCLCSGEKGLGKTTGKKLCYKGSTF
XP_004419619	DRPQCHFDIEINREPVGRIMFQLFSDICPKTCKNFLCLCSGEKGLGKTTGKKLCYKGSTF
XP_003363180	DRPQCHFDIEINREPVGRIMFQLFSDICPKTCKNFLCLCSGEKGLGKTTGKKLCYKGSTF
XP_014986734	DRPQCHFDIEINREPVGRIMFQLFSDICPKTCKNFLCLCSGEKGLGKTTGKKLCYKGSTF
XP_014715581	DRPQCHFDIEINREPVGRIMFQLFSDICPKTCKNFLCLCSGEKGLGKTTGKKLCYKGSTF
XP_014646446	DRPQCHFDIEINREPVGRIMFQLFSDICPKTCKNFLCLCSGEKGLGKTTGKKLCYKGSTF
XP_006893048	DRPQCHFDIEINREPVGRIMFQLFSDICPKTCKNFLCLCSGEKGLGKTTGKKLCYKGSTF
XP_012625718	DRPQCHFDIEINREPVGRIMFQLFSDICPKTCKNFLCLCSGEKGLGKTTGKKLCYKGSTF
XP_010973061	DRPQCHFDIEINREPVGRIMFQLFSDICPKTCKNFLCLCSGEKGLGKTTGKKLCYKGSTF
XP_010944206	DRPQCHFDIEINREPVGRIMFQLFSDICPKTCKNFLCLCSGEKGLGKTTGKKLCYKGSTF
XP_007193512	DRPQCHFDIEINREPVGRIMFQLFSDICPKTCKNFLCLCSGEKGLGKTTGKKLCYKGSTF
XP_015091829	DRPQCHFDIEINREPVGRIMFQLFSDICPKTCKNFLCLCSGEKGLGKTTGKKLCYKGSTF
XP_014411882	DRPQCHFDIEINREPVGRIMFQLFSDICPKTCKNFLCLCSGEKGLGKTTGKKLCYKGSTF
XP_006770574	DRPQCHFDIEINREPVGRIMFQLFSDICPKTCKNFLCLCSGEKGLGKTTGKKLCYKGSTF
XP_006770573	DRPQCHFDIEINREPVGRIMFQLFSDICPKTCKNFLCLCSGEKGLGKTTGKKLCYKGSTF
XP_003809334	DRPQCHFDIEINREPVGRIMFQLFSDICPKTCKNFLCLCSGEKGLGKTTGKKLCYKGSTF
ELK25111	DRPQCHFDIEINREPVGRIMFQLFSDICPKTCKNFLCLCSGEKGLGKTTGKKLCYKGSTF
XP_023087260	DRPQCHFDIEINREPVGRIMFQLFSDICPKTCKNFLCLCSGEKGLGKTTGKKLCYKGSTF
XP_005546833	DRPQCHFDIEINREPVGRIMFQLFSDICPKTCKNFLCLCSGEKGLGKTTGKKLCYKGSTF
XP_008258471	DRPQCHFDIEINREPVGRIMFQLFSDICPKTCKNFLCLCSGEKGLGKTTGKKLCYKGSTF
XP_008258470	DRPQCHFDIEINREPVGRIMFQLFSDICPKTCKNFLCLCSGEKGLGKTTGKKLCYKGSTF
XP_004708608	DRPQCHFDIEINREPVGRIMFQLFSDICPKTCKNFLCLCSGEKGLGKTTGKKLCYKGSTF
XP_003309783	DRPQCHFDIEINREPVGRIMFQLFSDICPKTCKNFLCLCSGEKGLGKTTGKKLCYKGSTF
XP_009443557	DRPQCHFDIEINREPVGRIMFQLFSDICPKTCKNFLCLCSGEKGLGKTTGKKLCYKGSTF
XP_011889503	DRPQCHFDIEINREPVGRIMFQLFSDICPKTCKNFLCLCSGEKGLGKTTGKKLCYKGSTF
XP_019274386	DRPQCHFDIEINREPVGRIMFQLFSDICPKTCKNFLCLCSGEKGLGKTTGKKLCYKGSTF
XP_015392541	DRPQCHFDIEINREPVGRIMFQLFSDICPKTCKNFLCLCSGEKGLGKTTGKKLCYKGSTF
XP_021573498	DRPQCHFDIEINREPVGRIMFQLFSDICPKTCKNFLCLCSGEKGLGKTTGKKLCYKGSTF
XP_017396461	DRPQCHFDIEINREPVGRIMFQLFSDICPKTCKNFLCLCSGEKGLGKTTGKKLCYKGSTF
XP_005546834	DRPQCHFDIEINREPVGRIMFQLFSDICPKTCKNFLCLCSGEKGLGKTTGKKLCYKGSTF
XP_014986732	DRPQCHFDIEINREPVGRIMFQLFSDICPKTCKNFLCLCSGEKGLGKTTGKKLCYKGSTF
XP_018879079	DRPQCHFDIEINREPVGRIMFQLFSDICPKTCKNFLCLCSGEKGLGKTTGKKLCYKGSTF
XP_014986735	DRPQCHFDIEINREPVGRIMFQLFSDICPKTCKNFLCLCSGEKGLGKTTGKKLCYKGSTF
XP_009443553	DRPQCHFDIEINREPVGRIMFQLFSDICPKTCKNFLCLCSGEKGLGKTTGKKLCYKGSTF
XP_016861963	DRPQCHFDIEINREPVGRIMFQLFSDICPKTCKNFLCLCSGEKGLGKTTGKKLCYKGSTF
XP_011839703	DRPQCHFDIEINREPVGRIMFQLFSDICPKTCKNFLCLCSGEKGLGKTTGKKLCYKGSTF
XP_006713234	DRPQCHFDIEINREPVGRIMFQLFSDICPKTCKNFLCLCSGEKGLGKTTGKKLCYKGSTF
XP_017396460	DRPQCHFDIEINREPVGRIMFQLFSDICPKTCKNFLCLCSGEKGLGKTTGKKLCYKGSTF

XP_018879080	DRPQCHFDIEINREPVGRIMFQLFSDICPKTCKNFLCLCSGEKGLGKTTGKKLCYKGSTF
XP_010372621	DRPQCHFDIEINREPVGRIMFQLFSDICPKTCKNFLCLCSGEKGLGKTTGKKLCYKGSTF
XP_007981992	DRPQCHFDIEINREPVGRIMFQLFSDICPKTCKNFLCLCSGEKGLGKTTGKKLCYKGSTF
XP_009199351	DRPQCHFDIEINREPVGRIMFQLFSDICPKTCKNFLCLCSGEKGLGKTTGKKLCYKGSTF
PNJ74565	DRPQCHFDIEINREPVGRIMFQLFSDICPKTCKNFLCLCSGEKGLGKTTGKKLCYKGSTF
XP_023087259	DRPQCHFDIEINREPVGRIMFQLFSDICPKTCKNFLCLCSGEKGLGKTTGKKLCYKGSTF
XP_009237437	DRPQCHFDIEINREPVGRIMFQLFSDICPKTCKNFLCLCSGEKGLGKTTGKKLCYKGSTF
XP_023087256	DRPQCHFDIEINREPVGRIMFQLFSDICPKTCKNFLCLCSGEKGLGKTTGKKLCYKGSTF
XP_010372620	DRPQCHFDIEINREPVGRIMFQLFSDICPKTCKNFLCLCSGEKGLGKTTGKKLCYKGSTF
XP_004414069	DRPQCHFDIEINREPVGRIMFQLFSDICPKTCKNFLCLCSGEKGLGKTTGKKLCYKGSTF
XP_011231109	DRPQCHFDIEINREPVGRIMFQLFSDICPKTCKNFLCLCSGEKGLGKTTGKKLCYKGSTF
XP_015392540	DRPQCHFDIEINREPVGRIMFQLFSDICPKTCKNFLCLCSGEKGLGKTTGKKLCYKGSTF
XP_011231110	DRPQCHFDIEINREPVGRIMFQLFSDICPKTCKNFLCLCSGEKGLGKTTGKKLCYKGSTF
XP_015392538	DRPQCHFDIEINREPVGRIMFQLFSDICPKTCKNFLCLCSGEKGLGKTTGKKLCYKGSTF
XP_004754311	DRPQCHFDIEINREPVGRIMFQLFSDICPKTCKNFLCLCSGEKGLGKTTGKKLCYKGSTF
XP_011806342	DRPQCHFDIEINREPVGRIMFQLFSDICPKTCKNFLCLCSGEKGLGKTTGKKLCYKGSTF
XP_005546832	DRPQCHFDIEINREPVGRIMFQLFSDICPKTCKNFLCLCSGEKGLGKTTGKKLCYKGSTF
XP_005546835	DRPQCHFDIEINREPVGRIMFQLFSDICPKTCKNFLCLCSGEKGLGKTTGKKLCYKGSTF
XP_023384831	DRPQCHFDIEINREPVGRIMFQLFSDICPKTCKNFLCLCSGEKGLGKTTGKKLCYKGSTF
XP_015447191	DRPQCHFDIEINREPVGRIMFQLFSDICPKTCKNFLCLCSGEKGLGKTTGKKLCYKGSTF
XP_006868826	DRPQCHFDIEINREPVGRIMFQLFSDICPKTCKNFLCLCSGEKGLGKTTGKKLCYKGSTF
XP_006102363	DRPQCHFDIEINREPVGRIMFQLFSDICPKTCKNFLCLCSGEKGLGKTTGKKLCYKGSTF
XP_005860445	DRPQCHFDIEINREPVGRIMFQLFSDICPKTCKNFLCLCSGEKGLGKTTGKKLCYKGSTF
XP_019274385	DRPQCHFDIEINREPVGRIMFQLFSDICPKTCKNFLCLCSGEKGLGKTTGKKLCYKGSTF
XP_014323127	DRPQCHFDIEINREPVGRIMFQLFSDICPKTCKNFLCLCSGEKGLGKTTGKKLCYKGSTF
XP_005860444	DRPQCHFDIEINREPVGRIMFQLFSDICPKTCKNFLCLCSGEKGLGKTTGKKLCYKGSTF
XP_023988067	DRPQCHFDIEINREPVGRIMFQLFSDICPKTCKNFLCLCSGEKGLGKTTGKKLCYKGSTF
XP_019274384	DRPQCHFDIEINREPVGRIMFQLFSDICPKTCKNFLCLCSGEKGLGKTTGKKLCYKGSTF
XP_017396459	DRPQCHFDIEINREPVGRIMFQLFSDICPKTCKNFLCLCSGEKGLGKTTGKKLCYKGSTF
XP_008152845	DRPQCHFDIEINREPVGRIMFQLFSDICPKTCKNFLCLCSGEKGLGKTTGKKLCYKGSTF
NP_001336053	DRPQCHFDIEINREPVGRIMFQLFSDICPKTCKNFLCLCSGEKGLGKTTGKKLCYKGSTF
XP_007106821	DRPQCHFDIEINREPVGRIMFQLFSDICPKTCKNFLCLCSGEKGLGKTTGKKLCYKGSTF
XP_018879078	DRPQCHFDIEINREPVGRIMFQLFSDICPKTCKNFLCLCSGEKGLGKTTGKKLCYKGSTF
XP_017396458	DRPQCHFDIEINREPVGRIMFQLFSDICPKTCKNFLCLCSGEKGLGKTTGKKLCYKGSTF
XP_012315533	DRPQCHFDIEINREPVGRIMFQLFSDICPKTCKNFLCLCSGEKGLGKTTGKKLCYKGSTF
XP_007981991	DRPQCHFDIEINREPVGRIMFQLFSDICPKTCKNFLCLCSGEKGLGKTTGKKLCYKGSTF
XP_003256959	DRPQCHFDIEINREPVGRIMFQLFSDICPKTCKNFLCLCSGEKGLGKTTGKKLCYKGSTF
XP_003735136	DRPQCHFDIEINREPVGRIMFQLFSDICPKTCKNFLCLCSGEKGLGKTTGKKLCYKGSTF
XP_019802415	DRPQCHFDIEINREPVGRIMFQLFSDICPKTCKNFLCLCSGEKGLGKTTGKKLCYKGSTF
XP_018879076	DRPQCHFDIEINREPVGRIMFQLFSDICPKTCKNFLCLCSGEKGLGKTTGKKLCYKGSTF
XP_012315532	DRPQCHFDIEINREPVGRIMFQLFSDICPKTCKNFLCLCSGEKGLGKTTGKKLCYKGSTF
XP_011737181	DRPQCHFDIEINREPVGRIMFQLFSDICPKTCKNFLCLCSGEKGLGKTTGKKLCYKGSTF
XP_011889502	DRPQCHFDIEINREPVGRIMFQLFSDICPKTCKNFLCLCSGEKGLGKTTGKKLCYKGSTF
XP_010372619	DRPQCHFDIEINREPVGRIMFQLFSDICPKTCKNFLCLCSGEKGLGKTTGKKLCYKGSTF
XP_007981990	DRPQCHFDIEINREPVGRIMFQLFSDICPKTCKNFLCLCSGEKGLGKTTGKKLCYKGSTF
XP_004277919	DRPQCHFDIEINREPVGRIMFQLFSDICPKTCKNFLCLCSGEKGLGKTTGKKLCYKGSTF
XP_003894654	DRPQCHFDIEINREPVGRIMFQLFSDICPKTCKNFLCLCSGEKGLGKTTGKKLCYKGSTF
XP_019802412	DRPQCHFDIEINREPVGRIMFQLFSDICPKTCKNFLCLCSGEKGLGKTTGKKLCYKGSTF
XP_017811031	DRPQCHFDIEINREPVGRIMFQLFSDICPKTCKNFLCLCSGEKGLGKTTGKKLCYKGSTF
XP_008982345	DRPQCHFDIEINREPVGRIMFQLFSDICPKTCKNFLCLCSGEKGLGKTTGKKLCYKGSTF
XP_003477052	DRPQCHFDIEINREPVGRIMFQLFSDICPKTCKNFLCLCSGEKGLGKTTGKKLCYKGSTF
XP_013015643	DRPQCHFDIEINREPVGRIMFQLFSDICPKTCKNFLCLCSGEKGLGKTTGKKLCYKGSTF
XP_007499863	DRPQCHFDIEINREPVGRIMFQLFSDICPKTCKNFLCLCSGEKGLGKTTGKKLCYKGSTF
XP_023581750	DRPQCHFDIEINREPVGRIMFQLFSDICPKTCKNFLCLCSGEKGLGKTTGKKLCYKGSTF
XP_002813940	DRPQCHFDIEINREPVGRIMFQLFSDICPKTCKNFLCLCSGEKGLGKTTGKKLCYKGSTF
XP_014390380	DRPQCHFDIEINREPVGRIMFQLFSDICPKTCKNFLCLCSGEKGLGKTTGKKLCYKGSTF
XP_014323126	DRPQCHFDIEINREPVGRIMFQLFSDICPKTCKNFLCLCSGEKGLGKTTGKKLCYKGSTF
XP_024100262	DRPQCHFDIEINREPVGRIMFQLFSDICPKTCKNFLCLCSGEKGLGKTTGKKLCYKGSTF

XP_022409524	DRPQCHFDIEINREPVGRIMFQLFSDICPKTCKNFLCLCSGEKGLGKTTGKKLCYKGSTF
XP_022409523	DRPQCHFDIEINREPVGRIMFQLFSDICPKTCKNFLCLCSGEKGLGKTTGKKLCYKGSTF
XP_019802414	DRPQCHFDIEINREPVGRIMFQLFSDICPKTCKNFLCLCSGEKGLGKTTGKKLCYKGSTF
XP_021062318	DRPQCHFDIEINREPVGRIMFQLFSDICPKTCKNFLCLCSGEKGLGKTTGKKLCYKGSTF
XP_014390377	DRPQCHFDIEINREPVGRIMFQLFSDICPKTCKNFLCLCSGEKGLGKTTGKKLCYKGSTF
XP_014323123	DRPQCHFDIEINREPVGRIMFQLFSDICPKTCKNFLCLCSGEKGLGKTTGKKLCYKGSTF
XP_011889501	DRPQCHFDIEINREPVGRIMFQLFSDICPKTCKNFLCLCSGEKGLGKTTGKKLCYKGSTF
XP_015344917	DRPQCHFDIEINREPVGRIMFQLFSDICPKTCKNFLCLCSGEKGLGKTTGKKLCYKGSTF
XP_011889500	DRPQCHFDIEINREPVGRIMFQLFSDICPKTCKNFLCLCSGEKGLGKTTGKKLCYKGSTF
XP_016018800	DRPQCHFDIEINREPVGRIMFQLFSDICPKTCKNFLCLCSGEKGLGKTTGKKLCYKGSTF
XP_019802413	DRPQCHFDIEINREPVGRIMFQLFSDICPKTCKNFLCLCSGEKGLGKTTGKKLCYKGSTF
XP_023581752	DRPQCHFDIEINREPVGRIMFQLFSDICPKTCKNFLCLCSGEKGLGKTTGKKLCYKGSTF
XP_008847679	DRPQCHFDIEINREPVGRIMFQLFSDICPKTCKNFLCLCSGEKGLGKTTGKKLCYKGSTF
XP_016018799	DRPQCHFDIEINREPVGRIMFQLFSDICPKTCKNFLCLCSGEKGLGKTTGKKLCYKGSTF
XP_016018798	DRPQCHFDIEINREPVGRIMFQLFSDICPKTCKNFLCLCSGEKGLGKTTGKKLCYKGSTF
XP_017497112	DRPQCHFDIEINREPVGRIMFQLFSDICPKTCKNFLCLCSGEKGLGKTTGKKLCYKGSTF
XP_023371677	DRPQCHFDIEINREPVGRIMFQLFSDICPKTCKNFLCLCSGEKGVGKTTGKKLCYKGSTF
XP_007947878	DRPQCHFDIEINREPVGRIMFQLFSDICPKTCKNFLCLCSGEKGLGKTTGKKLCYKGSTF
XP_007445750	DRPQCHFDIEINREPVGRIMFQLFSDICPKTCKNFLCLCSGEKGLGKTTGKKLCYKGSTF
XP_011889497	DRPQCHFDIEINREPVGRIMFQLFSDICPKTCKNFLCLCSGEKGLGKTTGKKLCYKGSTF
XP_020854834	DRPQCHFDIEINREPVGRIMFQLFSDICPKTCKNFLCLCSGEKGLGKTTGKKLCYKGSTF
XP_012315531	DRPQCHFDIEINREPVGRIMFQLFSDICPKTCKNFLCLCSGEKGLGKTTGKKLCYKGSTF
XP_020854833	DRPQCHFDIEINREPVGRIMFQLFSDICPKTCKNFLCLCSGEKGLGKTTGKKLCYKGSTF
XP_012315530	DRPQCHFDIEINREPVGRIMFQLFSDICPKTCKNFLCLCSGEKGLGKTTGKKLCYKGSTF
XP_022409521	DRPQCHFDIEINREPVGRIMFQLFSDICPKTCKNFLCLCSGEKGLGKTTGKKLCYKGSTF
XP_023384830	DRPQCHFDIEINREPVGRIMFQLFSDICPKTCKNFLCLCSGEKGLGKTTGKKLCYKGSTF
XP_020041827	DRPQCHFDIEINREPVGRIMFQLFSDICPKTCKNFLCLCSGEKGLGKTTGKKLCYKGSTF
XP_015344916	DRPQCHFDIEINREPVGRIMFQLFSDICPKTCKNFLCLCSGEKGLGKTTGKKLCYKGSTF
XP_008847678	DRPQCHFDIEINREPVGRIMFQLFSDICPKTCKNFLCLCSGEKGLGKTTGKKLCYKGSTF
XP_015344914	DRPQCHFDIEINREPVGRIMFQLFSDICPKTCKNFLCLCSGEKGLGKTTGKKLCYKGSTF
XP_008847677	DRPQCHFDIEINREPVGRIMFQLFSDICPKTCKNFLCLCSGEKGLGKTTGKKLCYKGSTF
XP_005337989	DRPQCHFDIEINREPVGRIMFQLFSDICPKTCKNFLCLCSGEKGLGKTTGKKLCYKGSTF
XP_021590709	DRPQCHFDIEINREPVGRIMFQLFSDICPKTCKNFLCLCSGEKGLGKTTGKKLCYKGSTF
XP_022409522	DRPQCHFDIEINREPVGRIMFQLFSDICPKTCKNFLCLCSGEKGLGKTTGKKLCYKGSTF
XP_021590708	DRPQCHFDIEINREPVGRIMFQLFSDICPKTCKNFLCLCSGEKGLGKTTGKKLCYKGSTF
XP_021027182	DRPQCHFDIEINREPVGRIMFQLFSDICPKTCKNFLCLCSGEKGLGKTTGKKLCYKGSTF
XP_006914886	DRPQCHFDIEINREPVGRIMFQLFSDICPKTCKNFLCLCSGEKGLGKTTGKKLCYKGSTF
XP_011355685	DRPQCHFDIEINREPVGRIMFQLFSDICPKTCKNFLCLCSGEKGLGKTTGKKLCYKGSTF
XP_023384828	DRPQCHFDIEINREPVGRIMFQLFSDICPKTCKNFLCLCSGEKGLGKTTGKKLCYKGSTF
XP_006914885	DRPQCHFDIEINREPVGRIMFQLFSDICPKTCKNFLCLCSGEKGLGKTTGKKLCYKGSTF
XP_012663507	DRPQCHFDIEINREPVGRIMFQLFSDICPKTCKNFLCLCSGEKGVGKTTGKKLCYKGSTF
XP_012663506	DRPQCHFDIEINREPVGRIMFQLFSDICPKTCKNFLCLCSGEKGVGKTTGKKLCYKGSTF
XP_017497113	DRPQCHFDIEINREPVGRIMFQLFSDICPKTCKNFLCLCSGEKGLGKTTGKKLCYKGSTF
XP_016051563	DRPQCHFDIEINREPVGRIMFQLFSDICPKTCKNFLCLCSGEKGLGKTTGKKLCYKGSTF
XP_017497111	DRPQCHFDIEINREPVGRIMFQLFSDICPKTCKNFLCLCSGEKGLGKTTGKKLCYKGSTF
XP_017497110	DRPQCHFDIEINREPVGRIMFQLFSDICPKTCKNFLCLCSGEKGLGKTTGKKLCYKGSTF
XP_012315529	DRPQCHFDIEINREPVGRIMFQLFSDICPKTCKNFLCLCSGEKGLGKTTGKKLCYKGSTF
XP_020728074	DRPQCHFDIEINREPVGRIMFQLFSDICPKTCKNFLCLCSGEKGFGKTTGKKLCYKGSTF
XP_012315528	DRPQCHFDIEINREPVGRIMFQLFSDICPKTCKNFLCLCSGEKGLGKTTGKKLCYKGSTF
XP_020728073	DRPQCHFDIEINREPVGRIMFQLFSDICPKTCKNFLCLCSGEKGFGKTTGKKLCYKGSTF
XP_021062319	DRPQCHFDIEINREPVGRIMFQLFSDICPKTCKNFLCLCSGEKGLGKTTGKKLCYKGSTF
XP_021027181	DRPQCHFDIEINREPVGRIMFQLFSDICPKTCKNFLCLCSGEKGLGKTTGKKLCYKGSTF
XP_021027180	DRPQCHFDIEINREPVGRIMFQLFSDICPKTCKNFLCLCSGEKGLGKTTGKKLCYKGSTF
XP_004835151	DRPQCHFDIEINREPVGRIMFQLFSDICPKTCKNFLCLCSGEKGLGKTTGKKLCYKGSTF
XP_012928271	DRPQCHFDIEINREPVGRIMFQLFSDICPKTCKNFLCLCSGEKGLGKTTGKKLCYKGSTF
XP_021590705	DRPQCHFDIEINREPVGRIMFQLFSDICPKTCKNFLCLCSGEKGLGKTTGKKLCYKGSTF
XP_011241241	DRPQCHFDIEINREPVGRIMFQLFSDICPKTCKNFLCLCSGEKGLGKTTGKKLCYKGSTF
NP_035048	DRPQCHFDIEINREPVGRIMFQLFSDICPKTCKNFLCLCSGEKGLGKTTGKKLCYKGSTF

XP_017168694	DRPQCHFDIEINREPVGRIMFQLFSDICPKTCKNFLCLCSGEKGLGKTTGKKLCYKGSTF
XP_005348185	DRPQCHFDIEINREPVGRIMFQLFSDICPKTCKNFLCLCSGEKGLGKTTGKKLCYKGSTF
XP_005082370	DRPQCHFDIEINREPVGRIMFQLFSDICPKTCKNFLCLCSGEKGLGKTTGKKLCYKGSTF
XP_021503066	DRPQCHFDIEINREPVGRIMFQLFSDICPKTCKNFLCLCSGEKGLGKTTGKKLCYKGSTF
XP_021503065	DRPQCHFDIEINREPVGRIMFQLFSDICPKTCKNFLCLCSGEKGLGKTTGKKLCYKGSTF
XP_021503062	DRPQCHFDIEINREPVGRIMFQLFSDICPKTCKNFLCLCSGEKGLGKTTGKKLCYKGSTF
XP_021503063	DRPQCHFDIEINREPVGRIMFQLFSDICPKTCKNFLCLCSGEKGLGKTTGKKLCYKGSTF
XP_008568057	DRPQCHFDIEINREPVGRIMFQLFSDICPKTCKNFLCLCSGEKGLGKTTGKKLCYKGSTF
XP_008568056	DRPQCHFDIEINREPVGRIMFQLFSDICPKTCKNFLCLCSGEKGLGKTTGKKLCYKGSTF
XP_006120664	DRPQCFFDIEINREPVGRIMFQLFSDVCPKTCKNFLCLCSGEKGIGKTTGKKLCYKGSTF
XP_007055629	DRPQCFFDIEINREPVGRIMFQLFSDVCPKTCKNFLCLCSGEKGIGKTTGKKLCYKGSTF
XP_006120661	DRPQCFFDIEINREPVGRIMFQLFSDVCPKTCKNFLCLCSGEKGIGKTTGKKLCYKGSTF
XP_006120659	DRPQCFFDIEINREPVGRIMFQLFSDVCPKTCKNFLCLCSGEKGIGKTTGKKLCYKGSTF
XP_007106819	DRPQCHFDIEINREPVGRIMFQLFSDICPKTCKNFLCLCSGEKGLGKTTGKKLCYKGSTF
XP_015708506	DRPQCYFDIEINREPVGRIMFQLFSDICPKTCKNFLCLCSGEKGIGKTTGKKLCYKGTTF
XP_009682494	DRPQCFFDIEINREPVGRIMFQLFSDICPKTCKNFLCLCSGEKGLGKTTGKKLCYKGTTF
XP_009682493	DRPQCFFDIEINREPVGRIMFQLFSDICPKTCKNFLCLCSGEKGLGKTTGKKLCYKGTTF
XP_009682492	DRPQCFFDIEINREPVGRIMFQLFSDICPKTCKNFLCLCSGEKGLGKTTGKKLCYKGTTF
XP_009682491	DRPQCFFDIEINREPVGRIMFQLFSDICPKTCKNFLCLCSGEKGLGKTTGKKLCYKGTTF
XP_019471734	DRPQCFFDIEINREPVGRIMFQLFSDICPKTCKNFLCLCSGEKGIGKTTGKKLCYKGTTF
XP_010710549	DRPQCFFDIEINREPVGRIMFQLFSDICPKTCKNFLCLCSGEKGIGKTTGKKLCYKGTTF
XP_010710548	DRPQCFFDIEINREPVGRIMFQLFSDICPKTCKNFLCLCSGEKGIGKTTGKKLCYKGTTF
XP_015136632	DRPQCFFDIEINREPVGRIMFQLFSDICPKTCKNFLCLCSGEKGIGKTTGKKLCYKGTTF
XP_004939132	DRPQCFFDIEINREPVGRIMFQLFSDICPKTCKNFLCLCSGEKGIGKTTGKKLCYKGTTF
XP_004939131	DRPQCFFDIEINREPVGRIMFQLFSDICPKTCKNFLCLCSGEKGIGKTTGKKLCYKGTTF
XP_013795593	DRPQCFFDIEINREPVGRIMFQLFSDICPKTCKNFLCLCSGEKGIGKTTGKKLCYKGTTF
XP_019363610	DRPQCFFDIEINREPVGRIMFQLFSDICPKTCKNFLCLCSGEKGIGKTTGKKLCYKGTTF
XP_014376468	DRPQCFFDIEINREPVGRIMFQLFSDICPKTCKNFLCLCSGEKGIGKTTGKKLCYKGTTF
XP_007659513	DRPQCHFDIEINREPVGRIMFQLFSDICPKTCKNFLSLCSGERGLGKTTGKKLCYKGSTF
XP_021241246	DRPQCFFDIEINREPVGRIMFQLFSDICPKTCKNFLCLCSGEKGIGKTTGKKLCYKGTTF
XP_021241236	DRPQCFFDIEINREPVGRIMFQLFSDICPKTCKNFLCLCSGEKGIGKTTGKKLCYKGTTF
XP_021241235	DRPQCFFDIEINREPVGRIMFQLFSDICPKTCKNFLCLCSGEKGIGKTTGKKLCYKGTTF
XP_017731869	DRPQCHFDIEINREPVGRIMFQLFSDICPKXMQNFLCLCSGEKGLGKTTGKKLCYKGSTF
XP_012426793	DRPQCFFEIEINREPVGRIMFQLFSDICPKTCKNFLCLCSGEKGIGKTTGKKLCYKGTTF
XP_017944741	DRPQCFFEIEINREPVGRIMFQLFSDICPKTCKNFLCLCSGEKGIGKTTGKKLCYKGTTF
XP_017944740	DRPQCFFEIEINREPVGRIMFQLFSDICPKTCKNFLCLCSGEKGIGKTTGKKLCYKGTTF
XP_010393085	DRPQCFFEIEINREPVGRIMFQLFSDICPKTCKNFLCLCSGEKGIGKTTGKKLCYKGTTF
XP_017685518	DRPQCFFEIEINREPVGRIMFQLFSDICPKTCKNFLCLCSGEKGIGKTTGKKLCYKGTTF
XP_017602246	DRPQCFFEIEINREPVGRIMFQLFSDICPKTCKNFLCLCSGEKGIGKTTGKKLCYKGTTF
XP_016151723	DRPQCFFEIEINREPVGRIMFQLFSDICPKTCKNFLCLCSGEKGIGKTTGKKLCYKGTTF
XP_019138709	DRPQCFFEIEINREPVGRIMFQLFSDICPKTCKNFLCLCSGEKGIGKTTGKKLCYKGTTF
XP_017685517	DRPQCFFEIEINREPVGRIMFQLFSDICPKTCKNFLCLCSGEKGIGKTTGKKLCYKGTTF
XP_017602245	DRPQCFFEIEINREPVGRIMFQLFSDICPKTCKNFLCLCSGEKGIGKTTGKKLCYKGTTF
XP_005040539	DRPQCFFEIEINREPVGRIMFQLFSDICPKTCKNFLCLCSGEKGIGKTTGKKLCYKGTTF
XP_005040538	DRPQCFFEIEINREPVGRIMFQLFSDICPKTCKNFLCLCSGEKGIGKTTGKKLCYKGTTF
XP_005040537	DRPQCFFEIEINREPVGRIMFQLFSDICPKTCKNFLCLCSGEKGIGKTTGKKLCYKGTTF
XP_014800495	DRPQCFFEIEINREPVGRIMFQLFSDICPKTCKNFLCLCSGEKGIGKTTGKKLCYKGTTF
XP_008168983	DRPQCFFDIEINREPVGRIMFQLFSDVCPKTCKNFLCLCSGEKGIGKTTRKKLCYKGSTF
XP_017944739	DRPQCFFEIEINREPVGRIMFQLFSDICPKTCKNFLCLCSGEKGIGKTTGKKLCYKGTTF
XP_014729203	DRPQCFFEIEINREPVGRIMFQLFSDICPKTCKNFLCLCSGEKGIGKTTGKKLCYKGTTF
XP_017944736	DRPQCFFEIEINREPVGRIMFQLFSDICPKTCKNFLCLCSGEKGIGKTTGKKLCYKGTTF
XP_014729201	DRPQCFFEIEINREPVGRIMFQLFSDICPKTCKNFLCLCSGEKGIGKTTGKKLCYKGTTF
XP_010393084	DRPQCFFEIEINREPVGRIMFQLFSDICPKTCKNFLCLCSGEKGIGKTTGKKLCYKGTTF
XP_017944735	DRPQCFFEIEINREPVGRIMFQLFSDICPKTCKNFLCLCSGEKGIGKTTGKKLCYKGTTF
XP_017685520	DRPQCFFEIEINREPVGRIMFQLFSDICPKTCKNFLCLCSGEKGIGKTTGKKLCYKGTTF
XP_017602244	DRPQCFFEIEINREPVGRIMFQLFSDICPKTCKNFLCLCSGEKGIGKTTGKKLCYKGTTF
XP_014729200	DRPQCFFEIEINREPVGRIMFQLFSDICPKTCKNFLCLCSGEKGIGKTTGKKLCYKGTTF
XP_010393082	DRPQCFFEIEINREPVGRIMFQLFSDICPKTCKNFLCLCSGEKGIGKTTGKKLCYKGTTF

XP_017685515	DRPQCFFEIEINREPVGRIMFQLFSDICPKTCKNFLCLCSGEKGIGKTTGKKLCYKGTTF
XP_017602242	DRPQCFFEIEINREPVGRIMFQLFSDICPKTCKNFLCLCSGEKGIGKTTGKKLCYKGTTF
XP_017602241	DRPQCFFEIEINREPVGRIMFQLFSDICPKTCKNFLCLCSGEKGIGKTTGKKLCYKGTTF
KFP58102	DRPQCFFEIEINREPVGRIMFQLFSDICPKTCKNFLCLCSGEKGIGKTTGKKLCYKGTTF
XP_010393081	DRPQCFFEIEINREPVGRIMFQLFSDICPKTCKNFLCLCSGEKGIGKTTGKKLCYKGTTF
XP_017685514	DRPQCFFEIEINREPVGRIMFQLFSDICPKTCKNFLCLCSGEKGIGKTTGKKLCYKGTTF
XP_017602240	DRPQCFFEIEINREPVGRIMFQLFSDICPKTCKNFLCLCSGEKGIGKTTGKKLCYKGTTF
XP_010559721	DRPQCFFEIEINREPVGRIMFQLFSDICPKTCKNFLCLCSGEKGIGKTTGKKLCYKGTTF
XP_014800494	DRPQCFFEIEINREPVGRIMFQLFSDICPKTCKNFLCLCSGEKGIGKTTGKKLCYKGTTF
XP_011571332	DRPQCFFEIEINREPVGRIMFQLFSDICPKTCKNFLCLCSGEKGIGKTTGKKLCYKGTTF
XP_010559712	DRPQCFFEIEINREPVGRIMFQLFSDICPKTCKNFLCLCSGEKGIGKTTGKKLCYKGTTF
XP_009331567	DRPQCFFEIEINREPVGRIMFQLFSDICPKTCKNFLCLCSGEKGIGKTTGKKLCYKGTTF
XP_008492672	DRPQCFFEIEINREPVGRIMFQLFSDICPKTCKNFLCLCSGEKGIGKTTGKKLCYKGTTF
XP_014800492	DRPQCFFEIEINREPVGRIMFQLFSDICPKTCKNFLCLCSGEKGIGKTTGKKLCYKGTTF
XP_011571324	DRPQCFFEIEINREPVGRIMFQLFSDICPKTCKNFLCLCSGEKGIGKTTGKKLCYKGTTF
XP_009697766	DRPQCFFEIEINREPVGRIMFQLFSDICPKTCKNFLCLCSGEKGIGKTTGKKLCYKGTTF
XP_009331566	DRPQCFFEIEINREPVGRIMFQLFSDICPKTCKNFLCLCSGEKGIGKTTGKKLCYKGTTF
XP_009274213	DRPQCFFEIEINREPVGRIMFQLFSDICPKTCKNFLCLCSGEKGIGKTTGKKLCYKGTTF
XP_014800491	DRPQCFFEIEINREPVGRIMFQLFSDICPKTCKNFLCLCSGEKGIGKTTGKKLCYKGTTF
XP_010301634	DRPQCFFEIEINREPVGRIMFQLFSDICPKTCKNFLCLCSGEKGIGKTTGKKLCYKGTTF
XP_008168980	DRPQCFFDIEINREPVGRIMFQLFSDVCPKTCKNFLCLCSGEKGIGKTTRKKLCYKGSTF
XP_023963187	DRPQCFFDIEINREPVGRIMFQLFSDVCPKTCKNFLCLCSGEKGIGKTTRKKLCYKGSTF
XP_005150066	DRPQCFFEIEINREPVGRIMFQLFSDICPKTCKNFLCLCSGEKGIGKTTGKKLCYKGTTF
XP_009473612	DRPQCFFEIEINREPVGRIMFQLFSDICPKTCKNFLCLCSGEKGIGKTTGKKLCYKGTTF
XP_021393646	DRPQCFFEIEINREPVGRIMFQLFSDICPKTCKNFLCLCSGEKGIGKTTGKKLCYKGTTF
XP_021393642	DRPQCFFEIEINREPVGRIMFQLFSDICPKTCKNFLCLCSGEKGIGKTTGKKLCYKGTTF
XP_021393643	DRPQCFFEIEINREPVGRIMFQLFSDICPKTCKNFLCLCSGEKGIGKTTGKKLCYKGTTF
XP_009878793	DRPQCFFEIEINREPVGRIMFQLFSDICPKTCKNFLCLCSGEKGIGKTTGKKLCYKGTTF
XP_021393641	DRPQCFFEIEINREPVGRIMFQLFSDICPKTCKNFLCLCSGEKGIGKTTGKKLCYKGTTF
XP_018764676	DRPQCFFEIEINREPVGRIMFQLFSDICPKTCKNFLCLCSGEKGIGKTTGKKLCYKGTTF
XP_018764674	DRPQCFFEIEINREPVGRIMFQLFSDICPKTCKNFLCLCSGEKGIGKTTGKKLCYKGTTF
XP_018764673	DRPQCFFEIEINREPVGRIMFQLFSDICPKTCKNFLCLCSGEKGIGKTTGKKLCYKGTTF
KFV02250	DRPQCFFEIEINREPVGRIMFQLFSDICPKTCKNFLCLCSGEKGIGKTTGKKLCYKGTTF
XP_014125627	DRPQCFFEIEINREPVGRIMFQLFSDICPKTCKNFLCLCSGEKGIGKTTGKKLCYKGTTF
XP_010083448	DRPQCFFEIEINREPVGRIMFQLFSDICPKTCKNFLCLCSGEKGIGKTTGKKLCYKGTTF
XP_014125626	DRPQCFFEIEINREPVGRIMFQLFSDICPKTCKNFLCLCSGEKGIGKTTGKKLCYKGTTF
XP_014112250	DRPQCFFEIEINREPVGRIMFQLFSDICPKTCKNFLCLCSGEKGIGKTTGKKLCYKGTTF
XP_014112249	DRPQCFFEIEINREPVGRIMFQLFSDICPKTCKNFLCLCSGEKGIGKTTGKKLCYKGTTF
XP_014125625	DRPQCFFEIEINREPVGRIMFQLFSDICPKTCKNFLCLCSGEKGIGKTTGKKLCYKGTTF
XP_014112248	DRPQCFFEIEINREPVGRIMFQLFSDICPKTCKNFLCLCSGEKGIGKTTGKKLCYKGTTF
XP_005428264	DRPQCFFEIEINREPVGRIMFQLFSDICPKTCKNFLCLCSGEKGIGKTTGKKLCYKGTTF
XP_015474510	DRPQCFFEIEINREPVGRIMFQLFSDICPKTCKNFLCLCSGEKGIGKTTGKKLCYKGTTF
XP_023777798	DRPQCFFEIEINREPVGRIMFQLFSDICPKTCKNFLCLCSGEKGIGKTTGKKLCYKGTTF
XP_015474508	DRPQCFFEIEINREPVGRIMFQLFSDICPKTCKNFLCLCSGEKGIGKTTGKKLCYKGTTF
XP_023777797	DRPQCFFEIEINREPVGRIMFQLFSDICPKTCKNFLCLCSGEKGIGKTTGKKLCYKGTTF
XP_023777796	DRPQCFFEIEINREPVGRIMFQLFSDICPKTCKNFLCLCSGEKGIGKTTGKKLCYKGTTF
XP_015474507	DRPQCFFEIEINREPVGRIMFQLFSDICPKTCKNFLCLCSGEKGIGKTTGKKLCYKGTTF
XP_023777795	DRPQCFFEIEINREPVGRIMFQLFSDICPKTCKNFLCLCSGEKGIGKTTGKKLCYKGTTF
KFQ42818	DRPQCFFEIEINREPVGRIMFQLFSDICPKTCKNFLCLCSGEKGIGKTTGKKLCYKGTTF
XP_019355067	DRPQCFFDIEINREPVGRIMFQLFSDICPKTCKNFLCLCSGEKGIGKTTGKKLCYKGTTF
XP_010014722	DRPQCFFEIEINREPVGRIMFQLFSDICPKTCKNFLCLCSGEKGIGKTTGKKLCYKGTTF
XP_009556564	DRPQCFFEIEINREPVGRIMFQLFSDVCPKTCKNFLCLCSGEKGIGKTTGKKLCYKGTTF
XP_009993002	DRPQCFFEVEINREPVGRIMFQLFSDICPKTCKNFLCLCSGEKGIGKTTGKKLCYKGTTF
XP_009896433	DRPQCFFEIEINREPVGRIMFQLFSDICPKTCKNFLCLCSGEKGIGKTTGKKLCYKGTTF
XP_010213065	DRPQCFFDIEINREPVGRIMFQLFSDICPKTCKNFLCLCSGEKGIGKTTGKKLYYKGTTF
XP_019384731	DRPQCFFDIEINREPVGRIMFQLFSDICPKTCKNFLCLCSGEKGIGKTTGKKLYYKGTTF
XP_008110438	DRPQCFFDIEINREPVGRIVFQLFSDVCPRTCKNFLCLCSGEKGIGKTTEKKLCYKGSTF
XP_008110436	DRPQCFFDIEINREPVGRIVFQLFSDVCPRTCKNFLCLCSGEKGIGKTTEKKLCYKGSTF

XP_015264278	DRPQCFFDIEINREPVGRIIFQLFSDVCPRTCKNFLCLCTGEKGIGKTTGKKLCYEGSTF
XP_013917336	DRPQCFFDIEINREPVGRIIFQLFSDVCPRTCKNFLCLCTGEKGIGKTTGKKLCYKGSTF
XP_020670421	DRPQCFFDIEINREPVGRIVFQLFSDVCPRTCKNFLSLCTGENGIGKTTGKKLCYKGSTF
XP_020670420	DRPQCFFDIEINREPVGRIVFQLFSDVCPRTCKNFLSLCTGENGIGKTTGKKLCYKGSTF
XP_015667896	DRPQCFFDIEINREPVGRIIFQLFSDVCPRTCKNFLCLCTGEKGIGKTTGKKLCYKGSTF
XP_007443413	DRPQCFFDIEINREPVGRIIFQLFSDVCPRTCKNFLCLCTGEKGIGKTTGKKLWYKGSTF
XP_007443411	DRPQCFFDIEINREPVGRIIFQLFSDVCPRTCKNFLCLCTGEKGIGKTTGKKLWYKGSTF
XP_017451684	DRPQCHFDIEINGSPVGRIMFSSSQTYVPKTCKNFLCLCNGEKGLGKTTGKKLCYKGSTF
XP_014918330	-PAEVPLDIEINREPVGRIMFQLFSDICPKTCKNFLCLCSGEKGLGKTTGKKLCYKGSTF
XP_002939352	ARPQCYFDVEINREPVGRIVFQLFSDVCPKTCMNFLCLCTGEKGIGKVTGKKLCYKGSTF
XP_018124467	ARPQCYFDVEINREPIGRIVFQLFSDVCPKTCMNFLCLCTGEKGIGKVTGKKLCYKGSTF
XP_017572959	DRPQCYFDVEINREPVGRIVFKLFSDVCPKTSKNFLCLCTGEKDTGKTTGKKLCYKGSTF
XP_018607018	DRPQCYFDVEVNREPVGRIVFQLFSDICPKTSKNFLCLCTGEKGTGKTTGKYLCYKGSTF
XP_018607016	DRPQCYFDVEVNREPVGRIVFQLFSDICPKTSKNFLCLCTGEKGTGKTTGKYLCYKGSTF
XP_007544596	DRPQCYFDVELNREPVGRIVFQLFSDVCPKTSKNFLCLCTGEKGTGKVTRKKLCYKGSTF
XP_018607022	DRPQCYFDVEVNREPVGRIVFQLFSDICPKTSKNFLCLCTGEKGTGKTTGKYLCYKGSTF
XP_009443552	DRPQCHFDIEINREPVGRIMFQLFSDICPKTCKNFLCLCSGEKGLGKTTGKKLCYKGSTF
PNJ74568	DRPQCHFDIEINREPVGRIMFQLFSDICPKTCKNFLCLCSGEKGLGKTTGKKLCYKGSTF
PNJ74566	DRPQCHFDIEINREPVGRIMFQLFSDICPKTCKNFLCLCSGEKGLGKTTGKKLCYKGSTF
XP_018122561	ARPQCYFNVEVNREPIGRIVFQLFSDVCPETCMNFLCLCTGEKGIGKVTGKKLCYKGSTF
XP_012735608	DRPQCYFDVELNREPVGRIVFQLFSDVCPKTSKNFLCLCTGEKGTGKVTGKKLCYKGSTF
XP_022065410	DRPQCYFDVELNREPVGRIVFQLFSDVCPKTSKNFLCLCTGERGIGKITGKKLCYKGSTF
XP_023122049	DRPQCYFDVELNREPVGRIVFQLFSDVCPKTSKNFLCLCTGERGIGKITGKKLCYKGSTF
XP_022065408	DRPQCYFDVELNREPVGRIVFQLFSDVCPKTSKNFLCLCTGERGIGKITGKKLCYKGSTF
XP_023122045	DRPQCYFDVELNREPVGRIVFQLFSDVCPKTSKNFLCLCTGERGIGKITGKKLCYKGSTF
XP_022065407	DRPQCYFDVELNREPVGRIVFQLFSDVCPKTSKNFLCLCTGERGIGKITGKKLCYKGSTF
XP_022065406	DRPQCYFDVELNREPVGRIVFQLFSDVCPKTSKNFLCLCTGERGIGKITGKKLCYKGSTF
XP_023122044	DRPQCYFDVELNREPVGRIVFQLFSDVCPKTSKNFLCLCTGERGIGKITGKKLCYKGSTF
XP_022065405	DRPQCYFDVELNREPVGRIVFQLFSDVCPKTSKNFLCLCTGERGIGKITGKKLCYKGSTF
XP_023122043	DRPQCYFDVELNREPVGRIVFQLFSDVCPKTSKNFLCLCTGERGIGKITGKKLCYKGSTF
XP_018414151	ARPQCYFDVEINRDPVGRIVFQLFSDVCPKTCMNFLSLCTGEKGIGKVTGKKLWYKGSTF
XP_023490562	-MCFIS---------VGRIMFQLFSDICPKTCKNFLCLCSGEKGLGKTTGKKLCYKGSTF
XP_008304546	DRPQCYFDVELNREPVGRIVFQLFSDICPKTSKNFLCLCTGERGTGKITGKKLCYKGSTF
KKF16876	DRPQCYFDVELNREPVGRIVFQLFSDICPKTSKNFLCLCTGEKGTGKITGKKLCYKGSTF
XP_008304545	DRPQCYFDVELNREPVGRIVFQLFSDICPKTSKNFLCLCTGERGTGKITGKKLCYKGSTF
XP_015249666	DRPQCYFDVELNREPVGRIVFQLFSDVCPKTSKNFLCLCTGEKGTGKVTGKKLCYKGSTF
XP_018529332	DRPQCYFDVELNREPVGRIVFQLFSDVCPKTSKNFLCLCTGEKGTGKITGKKLCYKGSTF
XP_018529331	DRPQCYFDVELNREPVGRIVFQLFSDVCPKTSKNFLCLCTGEKGTGKITGKKLCYKGSTF
XP_010751380	DRPQCYFDVELNREPVGRIVFQLFSDICPKTSKNFLCLCTGEKGTGKITGKKLCYKGSTF
XP_010751371	DRPQCYFDVELNREPVGRIVFQLFSDICPKTSKNFLCLCTGEKGTGKITGKKLCYKGSTF
XP_019745550	DRPQCYFDVELNREPVGRIVFQLFSDICPKTSKNFLCLCTGEKGAGKITGKKLCYKGSTF
XP_010751365	DRPQCYFDVELNREPVGRIVFQLFSDICPKTSKNFLCLCTGEKGTGKITGKKLCYKGSTF
XP_019745545	DRPQCYFDVELNREPVGRIVFQLFSDICPKTSKNFLCLCTGEKGAGKITGKKLCYKGSTF
XP_023263705	DRPQCYFDVELNREPVGRIVFQLFSDVCPKTSKNFLCLCTGEKGTGKITGKKLCYKGSTF
XP_023263706	DRPQCYFDVELNREPVGRIVFQLFSDVCPKTSKNFLCLCTGEKGTGKITGKKLCYKGSTF
XP_001338072	DRPQCYFDVEINREPVGRIVFQLFSDICPKTSKNFLCLCTGEKGSGKATGKKLCYKGSTF
XP_022599954	DRPQCYFDVELNREPVGRIVFQLFSDVCPKTSKNFLCLCTGEKGTGKITGKKLCYKGSTF
XP_019897364	ERPQCYFDVEINREPVGRIVFQLFSDVCPKTSKNFLSLCTGDRGAGKTTGKKLCFKGSTF
XP_010900666	ERPQCYFDVEINREPVGRIVFQLFSDVCPKTSKNFLSLCTGDRGAGKTTGKKLCFKGSTF
XP_010900665	ERPQCYFDVEINREPVGRIVFQLFSDVCPKTSKNFLSLCTGDRGAGKTTGKKLCFKGSTF
XP_010900664	ERPQCYFDVEINREPVGRIVFQLFSDVCPKTSKNFLSLCTGDRGAGKTTGKKLCFKGSTF
XP_019897363	ERPQCYFDVEINREPVGRIVFQLFSDVCPKTSKNFLSLCTGDRGAGKTTGKKLCFKGSTF
XP_010167755	----------------GRIMFQLFSDVCPKTCKNFLCLCSGEKGIGKTTGKKLCYKGTTF
XP_010900668	ERPQCYFDVEINREPVGRIVFQLFSDVCPKTSKNFLSLCTGDRGAGKTTGKKLCFKGSTF
XP_016427080	DRPQCYFDVEVNREPVGRIVFQLFSDICPKTSKNFLCLCTGEKSSGKTTGKKLCYKGSTF
XP_016318510	DRPQCYFDVEINREPVGRIVFQLFSDICPKTSKNFLCLCTGEKSSGKTTGKKLCYKGSTF
XP_015209664	DRPQCYFDVEINREPVGRIVFQLFSDICPKTSKNFLCLCTGEKGTGKVTGKKLCYKGSTF
XP_015209662	DRPQCYFDVEINREPVGRIVFQLFSDICPKTSKNFLCLCTGEKGTGKVTGKKLCYKGSTF

```
XP_015209665        DRPQCYFDVEINREPVGRIVFQLFSDICPKTSKNFLCLCTGEKGTGKVTGKKLCYKGSTF
XP_005912346        DRPQCYFDVELNREPIGRIVFQLFSDICPKTSKNFLCLCTGERGTGKVTRKKLCYKGSTF
XP_005722738        DRPQCYFDVELNREPIGRIVFQLFSDICPKTSKNFLCLCTGERGTGKVTRKKLCYKGSTF
XP_020457914        DRPQCYFDVELNREPVGRIVFQLFSDVCPKTSKNFLSLCTGERGTGRITGKKLCYKGSTF
XP_013132609        DRPQCYFDVELNREPIGRIVFQLFSDICPKTSKNFLCLCTGERGTGKVTRKKLCYKGSTF
XP_004546791        DRPQCYFDVELNREPIGRIVFQLFSDICPKTSKNFLCLCTGERGTGKVTRKKLCYKGSTF
XP_006795195        DRPQCYFDVELNREPIGRIVFQLFSDICPKTSKNFLCLCTGERGTGKVTRKKLCYKGSTF
XP_005912345        DRPQCYFDVELNREPIGRIVFQLFSDICPKTSKNFLCLCTGERGTGKVTRKKLCYKGSTF
XP_005722737        DRPQCYFDVELNREPIGRIVFQLFSDICPKTSKNFLCLCTGERGTGKVTRKKLCYKGSTF
XP_021475699        ERPQCYFDVEINREPVGRIVYQLFSDVCPKTSKNFLSLCTGERGTGKSTGKKLCFKGSTF
XP_004546790        DRPQCYFDVELNREPIGRIVFQLFSDICPKTSKNFLCLCTGERGTGKVTRKKLCYKGSTF
XP_003439241        DRPQCYFDVELNREPIGRIVFQLFSDICPKTSKNFLCLCTGERGTGKVTRKKLCYKGSTF
XP_006795194        DRPQCYFDVELNREPIGRIVFQLFSDICPKTSKNFLCLCTGERGTGKVTRKKLCYKGSTF
XP_023490560        -MCFIS---------VGRIMFQLFSDICPKTCKNFLCLCSGEKGLGKTTGKKLCYKGSTF
XP_015209666        DRPQCYFDVEINREPVGRIVFQLFSDICPKTSKNFLCLCTGEKGTGKVTGKKLCYKGSTF
XP_005797888        DRPQCYFDVELNREPVGRIVFQLFSDVCPKTSKNFLCLCTGEKGTGKVTRKKLCYKGSTF
CBN81984            DRPQCYFDVELNREPVGRIVFQLFSDVCPKTSKNFLSLCTGEKGTGKITGKKLCYKGSTF
XP_010559731        DRPQCFFEIEINREPVGRIMFQLFSDICPKTCKNFLCLCSGEKGIGKTTGKKLCYKGTTF
XP_007544415        DRPQCYFDVELNREPVGRIVFQLFSDVCPKTSKNFLCLCTGEKGTGKVTRKKLCYKGSTF
XP_014870682        DRPQCYFDVELNREPVGRIVFQLFSDVCPKTSKNFLCLCTGEKGTGKVTRKKLCYKGSTF
XP_011606242        DHPQCYFDVELNREPVGRIVFQLFSEICPKTCKNFLSLCTGERGSGKITGKKLCYKGSTF
CAG04245            DRPQCYFDVELNREPVGRIVFQLFSDICPKTCKNFLSLCTGERGSGKITGKKLCYKGSTF
XP_016887118        DRPQCYFDVELNREPVGRIVFQLFSDICPKTSKNFLSLCTGERGVGKVTGKKLCFKGSTF
XP_008306161        DRPQCYFDVELNREPVGRIVFQLFSDICPKTSKNFLSLCTGERGVGKVTGKKLCFKGSTF
XP_008394441        DRPQCYFDVELNREPVGRIVFQLFSDVCPKTSKNFLCLCTGEKGTGKVTRKKLCYKGSTF
XP_014825423        DRPQCYFDVELNREPVGRIVFQLFSDVCPKTSKNFLCLCTGEKGTGKVTRKKLCYKGSTF
XP_012398954        -------------------MFQLFSDICPKTCKNFLCLCSGEKGLGKTTGKKLCYKGSTF
XP_019063096        -------------------MFQLFSDICPKTCKNFLCLCSGEKGLGKTTGKKLCYKGSTF
XP_009073803        ----------------GRIMFQLFSDICPKTCKNFLCLCSGEKGIGKTTGKKLCYKGTTF
XP_016400109        DRPQCYFDVEINREPVGRIVCQLFSDVCPKTSKNFLCLCTGEKGSGKTTGKNLCYKGSTF
XP_016359192        DRPQCYFDVEINREPVGRIVCQLFSDVCPKTSKNFLCLCTGEKGSGKTTGKHLCYKGSTF
XP_004081327        DRPQCYFDVELNRDPVGRIVFQLYSDICPKTSKNFLCLCTGERGSGKVTGKKLCYKGSTF
XP_020568300        DRPQCYFDVELNRDPVGRIVFQLYSDICPKTSKNFLCLCTGERGSGKVTGKKLCYKGSTF
XP_016041020        MSGGET-----WHEEVGRIMFQLFSDICPKTCKNFLCLCSGEKGLGKTTGKKLCYKGSTF
XP_017942181        -------------------MFQLFSDICPKTCKNFLCLCSGEKGIGKTTGKKLCYKGTTF
XP_017942183        -------------------MFQLFSDICPKTCKNFLCLCSGEKGIGKTTGKKLCYKGTTF
XP_017942182        -------------------MFQLFSDICPKTCKNFLCLCSGEKGIGKTTGKKLCYKGTTF
XP_020501265        DRPQCYFDVELNREPVGRIVFQLFSDVCPKTSKNFLSLCTGEKGTGKITGKKLCYKGSTF
KFP01329            --------------PVGRIMFQLFSDICPKTCKNFLCLCSGEKGIGKTTGKKLCYKGTTF
XP_015856948        SPAGRSPALTGAVRPVGRIMFQLFSDICPKTCKNFLCLCSGEKGLGKTTGKKLCYKGSTF
XP_020139631        -MLLDVRQCLSIEELVGRIMFQLFSDICPKTCKNFLCLCSGEKGLGKTTGKKLCYKGSTF
XP_020139628        -MLLDVRQCLSIEELVGRIMFQLFSDICPKTCKNFLCLCSGEKGLGKTTGKKLCYKGSTF
XP_006982975        SPAGRSPALTGAVRPVGRIMFQLFSDICPKTCKNFLCLCSGEKGLGKTTGKKLCYKGSTF
XP_015856947        SPAGRSPALTGAVRPVGRIMFQLFSDICPKTCKNFLCLCSGEKGLGKTTGKKLCYKGSTF
XP_014427753        ---------PNGGSPVGRIMFQLFSDVCPKTCKNFLCLCSGEKGIGKTTGKKLCYKGSTF
XP_013857648        DRPQCYFDVELNREPVGRIVFQLFSDICPRTSKNFLCLCTGEKGTGKITGKKLCYKGSTF
XP_023656013        DRPQCYFDVEVNREPVGRIVFQLFSDICPKTSKNFLSLCTGEKGTSKVTGKNLCYKGSTF
XP_023656011        DRPQCYFDVEVNREPVGRIVFQLFSDICPKTSKNFLSLCTGEKGTSKVTGKNLCYKGSTF
XP_023656014        DRPQCYFDVEVNREPVGRIVFQLFSDICPKTSKNFLSLCTGEKGTSKVTGKNLCYKGSTF
XP_004448689        -MYFIS---------VGRIMFQLFSDICPKTCKNFLCLCSGEKGLGKTTGKKLCYKGSTF
XP_004448688        -MYFIS---------VGRIMFQLFSDICPKTCKNFLCLCSGEKGLGKTTGKKLCYKGSTF
XP_023476440        -MCFIS---------VGRIMFQLFSDICPKTCKNFLCLCSGEKGLGKTTGKKLCYKGSTF
                                       : .  .   *.   ***.**.*:.. .: * * * ::*:**

NP_005376           HRVVKNFMIQGGDFSEGNGKGGESIYGGYFK-----DENFILKHDRAFLLSMANRGKHTN
XP_014646452        HRVVKNFMIQGGDFSEGNGKGGESIYGGYFK-----DENFILKHDRAFLLSMANRGKHTN
XP_017657797        HRVVKNFMIQGGDFSEGNGKGGESIYGGYFK-----DENFILKHDRAFLLSMANRGKHTN
XP_012885690        HRVVKNFMIQGGDFSEGNGKGGESIYGGYFK-----DENFILKHDRAFLLSMANRGKHTN
```

```
XP_008530969       HRVVKNFMIQGGDFSEGNGKGGESIYGGYFK-----DENFILKHDRAFLLSMANRGKHTN
XP_015423871       HRVVKNFMIQGGDFSEGNGKGGESIYGGYFK-----DENFILKHDRAFLLSMANRGKHTN
XP_015091833       HRVVKNFMIQGGDFSEGNGKGGESIYGGYFK-----DENFILKHDRAFLLSMANRGKHTN
XP_010973060       HRVVKNFMIQGGDFSEGNGKGGESIYGGYFK-----DENFILKHDRAFLLSMANRGKHTN
XP_019583096       HRVVKNFMIQGGDFSEGNGKGGESIYGGYFK-----DENFILKHDRAFLLSMANRGKHTN
XP_011231111       HRVVKNFMIQGGDFSEGNGKGGESIYGGYFK-----DENFILKHDRAFLLSMANRGKHTN
XP_014646457       HRVVKNFMIQGGDFSEGNGKGGESIYGGYFK-----DENFILKHDRAFLLSMANRGKHTN
XP_023988069       HRVVKNFMIQGGDFSEGNGKGGESIYGGYFK-----DENFILKHDRAFLLSMANRGKHTN
XP_008530970       HRVVKNFMIQGGDFSEGNGKGGESIYGGYFK-----DENFILKHDRAFLLSMANRGKHTN
XP_017497119       HRVVKNFMIQGGDFSEGNGKGGESIYGGYFK-----DENFILKHDRAFLLSMANRGKHTN
XP_021503072       HRVVKNFMIQGGDFSEGNGKGGESIYGGYFK-----DENFILKHDRAFLLSMANRGKHTN
XP_021503071       HRVVKNFMIQGGDFSEGNGKGGESIYGGYFK-----DENFILKHDRAFLLSMANRGKHTN
XP_020041828       HRVVKNFMIQGGDFSEGNGKGGESIYGGYFK-----DENFILKHDRAFLLSMANRGKHTN
XP_021503070       HRVVKNFMIQGGDFSEGNGKGGESIYGGYFK-----DENFILKHDRAFLLSMANRGKHTN
XP_012590780       HRVVKNFMIQGGDFSEGNGKGGESIYGGYFK-----DENFILKHDRAFLLSMANRGKHTN
XP_019274390       HRVVKNFMIQGGDFSEGNGKGGESIYGGYFK-----DENFILKHDRAFLLSMANRGKHTN
XP_019489071       HRVVKNFMIQGGDFSEGNGKGGESIYGGYFK-----DENFILKHDRAFLLSMANRGKHTN
XP_019489063       HRVVKNFMIQGGDFSEGNGKGGESIYGGYFK-----DENFILKHDRAFLLSMANRGKHTN
XP_010588206       HRVVKNFMIQGGDFSEGNGKGGESIYGGYFK-----DENFILKHDRAFLLSMANRGKHTN
XP_023403600       HRVVKNFMIQGGDFSEGNGKGGESIYGGYFK-----DENFILKHDRAFLLSMANRGKHTN
XP_009982730       HRVVKNFMIQGGDFSEGNGKGGESIYGGYFK-----DENFILKHDRAFLLSMANRGKHTN
XP_019839655       HRVVKNFMIQGGDFSEGNGKGGESIYGGYFK-----DENFILKHDRAFLLSMANRGKHTN
XP_006512034       HRVVKNFMIQGGDFSEGNGKGGESIYGGYFK-----DENFILKHDRAFLLSMANRGKHTN
XP_024100264       HRVVKNFMIQGGDFSEGNGKGGESIYGGYFK-----DENFILKHDRAFLLSMANRGKHTN
XP_019666422       HRVVKNFMIQGGDFSEGNGKGGESIYGGYFK-----DENFILKHDRAFLLSMANRGKHTN
XP_008530962       HRVVKNFMIQGGDFSEGNGKGGESIYGGYFK-----DENFILKHDRAFLLSMANRGKHTN
XP_008530967       HRVVKNFMIQGGDFSEGNGKGGESIYGGYFK-----DENFILKHDRAFLLSMANRGKHTN
XP_010944204       HRVVKNFMIQGGDFSEGNGKGGESIYGGYFK-----DENFILKHDRAFLLSMANRGKHTN
XP_015091830       HRVVKNFMIQGGDFSEGNGKGGESIYGGYFK-----DENFILKHDRAFLLSMANRGKHTN
XP_014411884       HRVVKNFMIQGGDFSEGNGKGGESIYGGYFK-----DENFILKHDRAFLLSMANRGKHTN
XP_010944201       HRVVKNFMIQGGDFSEGNGKGGESIYGGYFK-----DENFILKHDRAFLLSMANRGKHTN
XP_009443554       HRVVKNFMIQGGDFSEGNGKGGESIYGGYFK-----DENFILKHDRAFLLSMANRGKHTN
XP_021241242       HRVVKNFMIQGGDFSEGNGKGGESIYGGYFK-----DENFILKHDRAFLLSMANRGKHTN
XP_020927360       HRVVKNFMIQGGDFSEGNGKGGESIYGGYFK-----DENFILKHDRAFLLSMANRGKHTN
XP_004581770       HRVVKNFMIQGGDFSEGNGKGGESIYGGYFK-----DENFILKHDRAFLLSMANRGKHTN
XP_005669469       HRVVKNFMIQGGDFSEGNGKGGESIYGGYFK-----DENFILKHDRAFLLSMANRGKHTN
XP_009443555       HRVVKNFMIQGGDFSEGNGKGGESIYGGYFK-----DENFILKHDRAFLLSMANRGKHTN
XP_005669468       HRVVKNFMIQGGDFSEGNGKGGESIYGGYFK-----DENFILKHDRAFLLSMANRGKHTN
XP_008708042       HRVVKNFMIQGGDFSEGNGKGGESIYGGYFK-----DENFILKHDRAFLLSMANRGKHTN
AAA37500           HRVVKNFMIQGGDFSEGNGKGGESIYGGYFK-----DENFILKHDRAFLLSMANRGKHTN
XP_004676331       HRVVKNFMIQGGDFSEGNGKGGESIYGGYFK-----DENFILKHDRAFLLSMANRGKHTN
XP_006748020       HRVVKNFMIQGGDFSEGNGKGGESIYGGYFK-----DENFILKHDRAFLLSMANRGKHTN
XP_006770577       HRVVKNFMIQGGDFSEGNGKGGESIYGGYFK-----DENFILKHDRAFLLSMANRGKHTN
XP_019583095       HRVVKNFMIQGGDFSEGNGKGGESIYGGYFK-----DENFILKHDRAFLLSMANRGKHTN
XP_015423867       HRVVKNFMIQGGDFSEGNGKGGESIYGGYFK-----DENFILKHDRAFLLSMANRGKHTN
XP_005383594       HRVVKNFMIQGGDFSEGNGKGGESIYGGYFK-----DENFILKHDRAFLLSMANRGKHTN
XP_019583089       HRVVKNFMIQGGDFSEGNGKGGESIYGGYFK-----DENFILKHDRAFLLSMANRGKHTN
XP_014445953       HRVVKNFMIQGGDFSEGNGKGGESIYGGYFK-----DENFILKHDRAFLLSMANRGKHTN
XP_005891140       HRVVKNFMIQGGDFSEGNGKGGESIYGGYFK-----DENFILKHDRAFLLSMANRGKHTN
XP_019489104       HRVVKNFMIQGGDFSEGNGKGGESIYGGYFK-----DENFILKHDRAFLLSMANRGKHTN
XP_012004520       HRVVKNFMIQGGDFSEGNGKGGESIYGGYFK-----DENFILKHDRAFLLSMANRGKHTN
XP_006051863       HRVVKNFMIQGGDFSEGNGKGGESIYGGYFK-----DENFILKHDRAFLLSMANRGKHTN
XP_005954297       HRVVKNFMIQGGDFSEGNGKGGESIYGGYFK-----DENFILKHDRAFLLSMANRGKHTN
XP_005695639       HRVVKNFMIQGGDFSEGNGKGGESIYGGYFK-----DENFILKHDRAFLLSMANRGKHTN
XP_011954828       HRVVKNFMIQGGDFSEGNGKGGESIYGGYFK-----DENFILKHDRAFLLSMANRGKHTN
XP_003992255       HRVVKNFMIQGGDFSEGNGKGGESIYGGYFK-----DENFILKHDRAFLLSMANRGKHTN
XP_002702773       HRVVKNFMIQGGDFSEGNGKGGESIYGGYFK-----DENFILKHDRAFLLSMANRGKHTN
XP_019666421       HRVVKNFMIQGGDFSEGNGKGGESIYGGYFK-----DENFILKHDRAFLLSMANRGKHTN
```

```
XP_019489077       HRVVKNFMIQGGDFSEGNGKGGESIYGGYFK-----DENFILKHDRAFLLSMANRGKHTN
XP_012004518       HRVVKNFMIQGGDFSEGNGKGGESIYGGYFK-----DENFILKHDRAFLLSMANRGKHTN
XP_011954826       HRVVKNFMIQGGDFSEGNGKGGESIYGGYFK-----DENFILKHDRAFLLSMANRGKHTN
XP_006051860       HRVVKNFMIQGGDFSEGNGKGGESIYGGYFK-----DENFILKHDRAFLLSMANRGKHTN
XP_005695638       HRVVKNFMIQGGDFSEGNGKGGESIYGGYFK-----DENFILKHDRAFLLSMANRGKHTN
XP_005196454       HRVVKNFMIQGGDFSEGNGKGGESIYGGYFK-----DENFILKHDRAFLLSMANRGKHTN
XP_851976          HRVVKNFMIQGGDFSEGNGKGGESIYGGYFK-----DENFILKHDRAFLLSMANRGKHTN
XP_012520957       HRVVKNFMIQGGDFSEGNGKGGESIYGGYFK-----DENFILKHDRAFLLSMANRGKHTN
XP_005634360       HRVVKNFMIQGGDFSEGNGKGGESIYGGYFK-----DENFILKHDRAFLLSMANRGKHTN
XP_003132182       HRVVKNFMIQGGDFSEGNGKGGESIYGGYFK-----DENFILKHDRAFLLSMANRGKHTN
XP_020139644       HRVVKNFMIQGGDFSEGNGKGGESIYGGYFK-----DENFILKHDRAFLLSMANRGKHTN
XP_014715584       HRVVKNFMIQGGDFSEGNGKGGESIYGGYFK-----DENFILKHDRAFLLSMANRGKHTN
XP_007193513       HRVVKNFMIQGGDFSEGNGKGGESIYGGYFK-----DENFILKHDRAFLLSMANRGKHTN
XP_005669463       HRVVKNFMIQGGDFSEGNGKGGESIYGGYFK-----DENFILKHDRAFLLSMANRGKHTN
XP_004419619       HRVVKNFMIQGGDFSEGNGKGGESIYGGYFK-----DENFILKHDRAFLLSMANRGKHTN
XP_003363180       HRVVKNFMIQGGDFSEGNGKGGESIYGGYFK-----DENFILKHDRAFLLSMANRGKHTN
XP_014986734       HRVVKNFMIQGGDFSEGNGKGGESIYGGYFK-----DENFILKHDRAFLLSMANRGKHTN
XP_014715581       HRVVKNFMIQGGDFSEGNGKGGESIYGGYFK-----DENFILKHDRAFLLSMANRGKHTN
XP_014646446       HRVVKNFMIQGGDFSEGNGKGGESIYGGYFK-----DENFILKHDRAFLLSMANRGKHTN
XP_006893048       HRVVKNFMIQGGDFSEGNGKGGESIYGGYFK-----DENFILKHDRAFLLSMANRGKHTN
XP_012625718       HRVVKNFMIQGGDFSEGNGKGGESIYGGYFK-----DENFILKHDRAFLLSMANRGKHTN
XP_010973061       HRVVKNFMIQGGDFSEGNGKGGESIYGGYFK-----DENFILKHDRAFLLSMANRGKHTN
XP_010944206       HRVVKNFMIQGGDFSEGNGKGGESIYGGYFK-----DENFILKHDRAFLLSMANRGKHTN
XP_007193512       HRVVKNFMIQGGDFSEGNGKGGESIYGGYFK-----DENFILKHDRAFLLSMANRGKHTN
XP_015091829       HRVVKNFMIQGGDFSEGNGKGGESIYGGYFK-----DENFILKHDRAFLLSMANRGKHTN
XP_014411882       HRVVKNFMIQGGDFSEGNGKGGESIYGGYFK-----DENFILKHDRAFLLSMANRGKHTN
XP_006770574       HRVVKNFMIQGGDFSEGNGKGGESIYGGYFK-----DENFILKHDRAFLLSMANRGKHTN
XP_006770573       HRVVKNFMIQGGDFSEGNGKGGESIYGGYFK-----DENFILKHDRAFLLSMANRGKHTN
XP_003809334       HRVVKNFMIQGGDFSEGNGKGGESIYGGYFK-----DENFILKHDRAFLLSMANRGKHTN
ELK25111           HRVVKNFMIQGGDFSEGNGKGGESIYGGYFK-----DENFILKHDRAFLLSMANRGKHTN
XP_023087260       HRVVKNFMIQGGDFSEGNGKGGESIYGGYFK-----DENFILKHDRAFLLSMANRGKHTN
XP_005546833       HRVVKNFMIQGGDFSEGNGKGGESIYGGYFK-----DENFILKHDRAFLLSMANRGKHTN
XP_008258471       HRVVKNFMIQGGDFSEGNGKGGESIYGGYFK-----DENFILKHDRAFLLSMANRGKHTN
XP_008258470       HRVVKNFMIQGGDFSEGNGKGGESIYGGYFK-----DENFILKHDRAFLLSMANRGKHTN
XP_004708608       HRVVKNFMIQGGDFSEGNGKGGESIYGGYFK-----DENFILKHDRAFLLSMANRGKHTN
XP_003309783       HRVVKNFMIQGGDFSEGNGKGGESIYGGYFK-----DENFILKHDRAFLLSMANRGKHTN
XP_009443557       HRVVKNFMIQGGDFSEGNGKGGESIYGGYFK-----DENFILKHDRAFLLSMANRGKHTN
XP_011889503       HRVVKNFMIQGGDFSEGNGKGGESIYGGYFK-----DENFILKHDRAFLLSMANRGKHTN
XP_019274386       HRVVKNFMIQGGDFSEGNGKGGESIYGGYFK-----DENFILKHDRAFLLSMANRGKHTN
XP_015392541       HRVVKNFMIQGGDFSEGNGKGGESIYGGYFK-----DENFILKHDRAFLLSMANRGKHTN
XP_021573498       HRVVKNFMIQGGDFSEGNGKGGESIYGGYFK-----DENFILKHDRAFLLSMANRGKHTN
XP_017396461       HRVVKNFMIQGGDFSEGNGKGGESIYGGYFK-----DENFILKHDRAFLLSMANRGKHTN
XP_005546834       HRVVKNFMIQGGDFSEGNGKGGESIYGGYFK-----DENFILKHDRAFLLSMANRGKHTN
XP_014986732       HRVVKNFMIQGGDFSEGNGKGGESIYGGYFK-----DENFILKHDRAFLLSMANRGKHTN
XP_018879079       HRVVKNFMIQGGDFSEGNGKGGESIYGGYFK-----DENFILKHDRAFLLSMANRGKHTN
XP_014986735       HRVVKNFMIQGGDFSEGNGKGGESIYGGYFK-----DENFILKHDRAFLLSMANRGKHTN
XP_009443553       HRVVKNFMIQGGDFSEGNGKGGESIYGGYFK-----DENFILKHDRAFLLSMANRGKHTN
XP_016861963       HRVVKNFMIQGGDFSEGNGKGGESIYGGYFK-----DENFILKHDRAFLLSMANRGKHTN
XP_011839703       HRVVKNFMIQGGDFSEGNGKGGESIYGGYFK-----DENFILKHDRAFLLSMANRGKHTN
XP_006713234       HRVVKNFMIQGGDFSEGNGKGGESIYGGYFK-----DENFILKHDRAFLLSMANRGKHTN
XP_017396460       HRVVKNFMIQGGDFSEGNGKGGESIYGGYFK-----DENFILKHDRAFLLSMANRGKHTN
XP_018879080       HRVVKNFMIQGGDFSEGNGKGGESIYGGYFK-----DENFILKHDRAFLLSMANRGKHTN
XP_010372621       HRVVKNFMIQGGDFSEGNGKGGESIYGGYFK-----DENFILKHDRAFLLSMANRGKHTN
XP_007981992       HRVVKNFMIQGGDFSEGNGKGGESIYGGYFK-----DENFILKHDRAFLLSMANRGKHTN
XP_009199351       HRVVKNFMIQGGDFSEGNGKGGESIYGGYFK-----DENFILKHDRAFLLSMANRGKHTN
PNJ74565           HRVVKNFMIQGGDFSEGNGKGGESIYGGYFK-----DENFILKHDRAFLLSMANRGKHTN
XP_023087259       HRVVKNFMIQGGDFSEGNGKGGESIYGGYFK-----DENFILKHDRAFLLSMANRGKHTN
XP_009237437       HRVVKNFMIQGGDFSEGNGKGGESIYGGYFK-----DENFILKHDRAFLLSMANRGKHTN
```

XP_023087256	HRVVKNFMIQGGDFSEGNGKGGESIYGGYFK-----DENFILKHDRAFLLSMANRGKHTN
XP_010372620	HRVVKNFMIQGGDFSEGNGKGGESIYGGYFK-----DENFILKHDRAFLLSMANRGKHTN
XP_004414069	HRVVKNFMIQGGDFSEGNGKGGESIYGGYFK-----DENFILKHDRAFLLSMANRGKHTN
XP_011231109	HRVVKNFMIQGGDFSEGNGKGGESIYGGYFK-----DENFILKHDRAFLLSMANRGKHTN
XP_015392540	HRVVKNFMIQGGDFSEGNGKGGESIYGGYFK-----DENFILKHDRAFLLSMANRGKHTN
XP_011231110	HRVVKNFMIQGGDFSEGNGKGGESIYGGYFK-----DENFILKHDRAFLLSMANRGKHTN
XP_015392538	HRVVKNFMIQGGDFSEGNGKGGESIYGGYFK-----DENFILKHDRAFLLSMANRGKHTN
XP_004754311	HRVVKNFMIQGGDFSEGNGKGGESIYGGYFK-----DENFILKHDRAFLLSMANRGKHTN
XP_011806342	HRVVKNFMIQGGDFSEGNGKGGESIYGGYFK-----DENFILKHDRAFLLSMANRGKHTN
XP_005546832	HRVVKNFMIQGGDFSEGNGKGGESIYGGYFK-----DENFILKHDRAFLLSMANRGKHTN
XP_005546835	HRVVKNFMIQGGDFSEGNGKGGESIYGGYFK-----DENFILKHDRAFLLSMANRGKHTN
XP_023384831	HRVVKNFMIQGGDFSEGNGKGGESIYGGYFK-----DENFVLKHDRAFLLSMANRGKHTN
XP_015447191	HRVVKNFMIQGGDFSEGNGKGGESIYGGYFK-----DENFVLKHDRAFLLSMANRGKHTN
XP_006868826	HRVVKNFMIQGGDFSEGNGKGGESIYGGYFK-----DENFILKHDRAFLLSMANRGKHTN
XP_006102363	HRVVKNFMIQGGDFSEGNGKGGESIYGGYFK-----DENFILKHDRAFLLSMANRGKHTN
XP_005860445	HRVVKNFMIQGGDFSEGNGKGGESIYGGYFK-----DENFILKHDRAFLLSMANRGKHTN
XP_019274385	HRVVKNFMIQGGDFSEGNGKGGESIYGGYFK-----DENFILKHDRAFLLSMANRGKHTN
XP_014323127	HRVVKNFMIQGGDFSEGNGKGGESIYGGYFK-----DENFILKHDRAFLLSMANRGKHTN
XP_005860444	HRVVKNFMIQGGDFSEGNGKGGESIYGGYFK-----DENFILKHDRAFLLSMANRGKHTN
XP_023988067	HRVVKNFMIQGGDFSEGNGKGGESIYGGYFK-----DENFILKHDRAFLLSMANRGKHTN
XP_019274384	HRVVKNFMIQGGDFSEGNGKGGESIYGGYFK-----DENFILKHDRAFLLSMANRGKHTN
XP_017396459	HRVVKNFMIQGGDFSEGNGKGGESIYGGYFK-----DENFILKHDRAFLLSMANRGKHTN
XP_008152845	HRVVKNFMIQGGDFSEGNGKGGESIYGGYFK-----DENFILKHDRAFLLSMANRGKHTN
NP_001336053	HRVVKNFMIQGGDFSEGNGKGGESIYGGYFK-----DENFILKHDRAFLLSMANRGKHTN
XP_007106821	HRVVKNFMIQGGDFSEGNGKGGESIYGGYFK-----DENFILKHDRAFLLSMANRGKHTN
XP_018879078	HRVVKNFMIQGGDFSEGNGKGGESIYGGYFK-----DENFILKHDRAFLLSMANRGKHTN
XP_017396458	HRVVKNFMIQGGDFSEGNGKGGESIYGGYFK-----DENFILKHDRAFLLSMANRGKHTN
XP_012315533	HRVVKNFMIQGGDFSEGNGKGGESIYGGYFK-----DENFILKHDRAFLLSMANRGKHTN
XP_007981991	HRVVKNFMIQGGDFSEGNGKGGESIYGGYFK-----DENFILKHDRAFLLSMANRGKHTN
XP_003256959	HRVVKNFMIQGGDFSEGNGKGGESIYGGYFK-----DENFILKHDRAFLLSMANRGKHTN
XP_003735136	HRVVKNFMIQGGDFSEGNGKGGESIYGGYFK-----DENFILKHDRAFLLSMANRGKHTN
XP_019802415	HRVVKNFMIQGGDFSEGNGKGGESIYGGYFK-----DENFILKHDRAFLLSMANRGKHTN
XP_018879076	HRVVKNFMIQGGDFSEGNGKGGESIYGGYFK-----DENFILKHDRAFLLSMANRGKHTN
XP_012315532	HRVVKNFMIQGGDFSEGNGKGGESIYGGYFK-----DENFILKHDRAFLLSMANRGKHTN
XP_011737181	HRVVKNFMIQGGDFSEGNGKGGESIYGGYFK-----DENFILKHDRAFLLSMANRGKHTN
XP_011889502	HRVVKNFMIQGGDFSEGNGKGGESIYGGYFK-----DENFILKHDRAFLLSMANRGKHTN
XP_010372619	HRVVKNFMIQGGDFSEGNGKGGESIYGGYFK-----DENFILKHDRAFLLSMANRGKHTN
XP_007981990	HRVVKNFMIQGGDFSEGNGKGGESIYGGYFK-----DENFILKHDRAFLLSMANRGKHTN
XP_004277919	HRVVKNFMIQGGDFSEGNGKGGESIYGGYFK-----DENFILKHDRAFLLSMANRGKHTN
XP_003894654	HRVVKNFMIQGGDFSEGNGKGGESIYGGYFK-----DENFILKHDRAFLLSMANRGKHTN
XP_019802412	HRVVKNFMIQGGDFSEGNGKGGESIYGGYFK-----DENFILKHDRAFLLSMANRGKHTN
XP_017811031	HRVVKNFMIQGGDFSEGNGKGGESIYGGYFK-----DENFILKHDRAFLLSMANRGKHTN
XP_008982345	HRVVKNFMIQGGDFSEGNGKGGESIYGGYFK-----DENFILKHDRAFLLSMANRGKHTN
XP_003477052	HRVVKNFMIQGGDFSEGNGKGGESIYGGYFK-----DENFILKHDRAFLLSMANRGKHTN
XP_013015643	HRVVKNFMIQGGDFSEGNGKGGESIYGGYFK-----DENFILKHDRAFLLSMANRGKHTN
XP_007499863	HRVVKNFMIQGGDFSEGNGKGGESIYGGYFK-----DENFVLKHDRAFLLSMANRGKHTN
XP_023581750	HRVVKNFMIQGGDFSEGNGKGGESIYGGYFK-----DENFILKHDRAFLLSMANRGKHTN
XP_002813940	HRVVKNFMIQGGDFSEGNGKGGESIYGGYFK-----DENFILKHDRAFLLSMANRGKHTN
XP_014390380	HRVVKNFMIQGGDFSEGNGKGGESIYGGYFK-----DENFILKHDRAFLLSMANRGKHTN
XP_014323126	HRVVKNFMIQGGDFSEGNGKGGESIYGGYFK-----DENFILKHDRAFLLSMANRGKHTN
XP_024100262	HRVVKNFMIQGGDFSEGNGKGGESIYGGYFK-----DENFILKHDRAFLLSMANRGKHTN
XP_022409524	HRVVKNFMIQGGDFSEGNGKGGESIYGGYFK-----DENFILKHDRAFLLSMANRGKHTN
XP_022409523	HRVVKNFMIQGGDFSEGNGKGGESIYGGYFK-----DENFILKHDRAFLLSMANRGKHTN
XP_019802414	HRVVKNFMIQGGDFSEGNGKGGESIYGGYFK-----DENFILKHDRAFLLSMANRGKHTN
XP_021062318	HRVVKNFMIQGGDFSEGNGKGGESIYGGYFK-----DENFILKHDRAFLLSMANRGKHTN
XP_014390377	HRVVKNFMIQGGDFSEGNGKGGESIYGGYFK-----DENFILKHDRAFLLSMANRGKHTN
XP_014323123	HRVVKNFMIQGGDFSEGNGKGGESIYGGYFK-----DENFILKHDRAFLLSMANRGKHTN
XP_011889501	HRVVKNFMIQGGDFSEGNGKGGESIYGGYFK-----DENFILKHDRAFLLSMANRGKHTN

XP_015344917	HRVVKNFMIQGGDFSEGNGKGGESIYGGYFK-----DENFILKHDRAFLLSMANRGKHTN
XP_011889500	HRVVKNFMIQGGDFSEGNGKGGESIYGGYFK-----DENFILKHDRAFLLSMANRGKHTN
XP_016018800	HRVVKNFMIQGGDFSEGNGKGGESIYGGYFK-----DENFVLKHDRAFLLSMANRGKHTN
XP_019802413	HRVVKNFMIQGGDFSEGNGKGGESIYGGYFK-----DENFILKHDRAFLLSMANRGKHTN
XP_023581752	HRVVKNFMIQGGDFSEGNGKGGESIYGGYFK-----DENFILKHDRAFLLSMANRGKHTN
XP_008847679	HRVVKNFMIQGGDFSEGNGKGGESIYGGYFK-----DENFILKHDRAFLLSMANRGKHTN
XP_016018799	HRVVKNFMIQGGDFSEGNGKGGESIYGGYFK-----DENFVLKHDRAFLLSMANRGKHTN
XP_016018798	HRVVKNFMIQGGDFSEGNGKGGESIYGGYFK-----DENFVLKHDRAFLLSMANRGKHTN
XP_017497112	HRVVKNFMIQGGDFSEGNGKGGESIYGGYFK-----DENFILKHDRAFLLSMANRGKHTN
XP_023371677	HRVVKNFMIQGGDFSEGNGKGGESIYGGYFK-----DENFILKHDRAFLLSMANRGKHTN
XP_007947878	HRVVKNFMIQGGDFSEGNGKGGESIYGGYFK-----DENFILKHDRAFLLSMANRGKHTN
XP_007445750	HRVVKNFMIQGGDFSEGNGKGGESIYGGYFK-----DENFILKHDRAFLLSMANRGKHTN
XP_011889497	HRVVKNFMIQGGDFSEGNGKGGESIYGGYFK-----DENFILKHDRAFLLSMANRGKHTN
XP_020854834	HRVVKNFMIQGGDFSEGNGKGGESIYGGYFK-----DENFVLKHDRAFLLSMANRGKHTN
XP_012315531	HRVVKNFMIQGGDFSEGNGKGGESIYGGYFK-----DENFILKHDRAFLLSMANRGKHTN
XP_020854833	HRVVKNFMIQGGDFSEGNGKGGESIYGGYFK-----DENFVLKHDRAFLLSMANRGKHTN
XP_012315530	HRVVKNFMIQGGDFSEGNGKGGESIYGGYFK-----DENFILKHDRAFLLSMANRGKHTN
XP_022409521	HRVVKNFMIQGGDFSEGNGKGGESIYGGYFK-----DENFILKHDRAFLLSMANRGKHTN
XP_023384830	HRVVKNFMIQGGDFSEGNGKGGESIYGGYFK-----DENFVLKHDRAFLLSMANRGKHTN
XP_020041827	HRVVKNFMIQGGDFSEGNGKGGESIYGGYFK-----DENFILKHDRAFLLSMANRGKHTN
XP_015344916	HRVVKNFMIQGGDFSEGNGKGGESIYGGYFK-----DENFILKHDRAFLLSMANRGKHTN
XP_008847678	HRVVKNFMIQGGDFSEGNGKGGESIYGGYFK-----DENFILKHDRAFLLSMANRGKHTN
XP_015344914	HRVVKNFMIQGGDFSEGNGKGGESIYGGYFK-----DENFILKHDRAFLLSMANRGKHTN
XP_008847677	HRVVKNFMIQGGDFSEGNGKGGESIYGGYFK-----DENFILKHDRAFLLSMANRGKHTN
XP_005337989	HRVVKNFMIQGGDFSEGNGKGGESIYGGYFK-----DENFILKHDRAFLLSMANRGKHTN
XP_021590709	HRVVKNFMIQGGDFSEGNGKGGESIYGGYFK-----DENFILKHDRAFLLSMANRGKHTN
XP_022409522	HRVVKNFMIQGGDFSEGNGKGGESIYGGYFK-----DENFILKHDRAFLLSMANRGKHTN
XP_021590708	HRVVKNFMIQGGDFSEGNGKGGESIYGGYFK-----DENFILKHDRAFLLSMANRGKHTN
XP_021027182	HRVVKNFMIQGGDFSEGNGKGGESIYGGYFK-----DENFILKHDRAFLLSMANRGKHTN
XP_006914886	HRVVKNFMIQGGDFSEGNGKGGESIYGGYFK-----DENFVLKHDRAFLLSMANRGKHTN
XP_011355685	HRVVKNFMIQGGDFSEGNGKGGESIYGGYFK-----DENFVLKHDRAFLLSMANRGKHTN
XP_023384828	HRVVKNFMIQGGDFSEGNGKGGESIYGGYFK-----DENFVLKHDRAFLLSMANRGKHTN
XP_006914885	HRVVKNFMIQGGDFSEGNGKGGESIYGGYFK-----DENFVLKHDRAFLLSMANRGKHTN
XP_012663507	HRVVKNFMIQGGDFSEGNGKGGESIYGGYFK-----DENFILKHDRAFLLSMANRGKHTN
XP_012663506	HRVVKNFMIQGGDFSEGNGKGGESIYGGYFK-----DENFILKHDRAFLLSMANRGKHTN
XP_017497113	HRVVKNFMIQGGDFSEGNGKGGESIYGGYFK-----DENFILKHDRAFLLSMANRGKHTN
XP_016051563	HRVVKNFMIQGGDFSEGNGKGGESIYGGYFK-----DENFILKHDRAFLLSMANRGKHTN
XP_017497111	HRVVKNFMIQGGDFSEGNGKGGESIYGGYFK-----DENFILKHDRAFLLSMANRGKHTN
XP_017497110	HRVVKNFMIQGGDFSEGNGKGGESIYGGYFK-----DENFILKHDRAFLLSMANRGKHTN
XP_012315529	HRVVKNFMIQGGDFSEGNGKGGESIYGGYFK-----DENFILKHDRAFLLSMANRGKHTN
XP_020728074	HRVVKNFMIQGGDFSEGNGKGGESIYGGYFK-----DENFILKHDRAFLLSMANRGKHTN
XP_012315528	HRVVKNFMIQGGDFSEGNGKGGESIYGGYFK-----DENFILKHDRAFLLSMANRGKHTN
XP_020728073	HRVVKNFMIQGGDFSEGNGKGGESIYGGYFK-----DENFILKHDRAFLLSMANRGKHTN
XP_021062319	HRVVKNFMIQGGDFSEGNGKGGESIYGGYFK-----DENFILKHDRAFLLSMANRGKHTN
XP_021027181	HRVVKNFMIQGGDFSEGNGKGGESIYGGYFK-----DENFILKHDRAFLLSMANRGKHTN
XP_021027180	HRVVKNFMIQGGDFSEGNGKGGESIYGGYFK-----DENFILKHDRAFLLSMANRGKHTN
XP_004835151	HRVVKNFMIQGGDFSEGNGKGGESIYGGYFK-----DENFILKHDRAFLLSMANRGKHTN
XP_012928271	HRVVKNFMIQGGDFSEGNGKGGESIYGGYFK-----DENFILKHDRAFLLSMANRGKHTN
XP_021590705	HRVVKNFMIQGGDFSEGNGKGGESIYGGYFK-----DENFILKHDRAFLLSMANRGKHTN
XP_011241241	HRVVKNFMIQGGDFSEGNGKGGESIYGGYFK-----DENFILKHDRAFLLSMANRGKHTN
NP_035048	HRVVKNFMIQGGDFSEGNGKGGESIYGGYFK-----DENFILKHDRAFLLSMANRGKHTN
XP_017168694	HRVVKNFMIQGGDFSEGNGKGGESIYGGYFK-----DENFILKHDRAFLLSMANRGKHTN
XP_005348185	HRVVKNFMIQGGDFSEGNGKGGESIYGGYFK-----DENFILKHDRAFLLSMANRGKHTN
XP_005082370	HRVVKNFMIQGGDFSEGNGKGGESIYGGYFK-----DENFILKHDRAFLLSMANRGKHTN
XP_021503066	HRVVKNFMIQGGDFSEGNGKGGESIYGGYFK-----DENFILKHDRAFLLSMANRGKHTN
XP_021503065	HRVVKNFMIQGGDFSEGNGKGGESIYGGYFK-----DENFILKHDRAFLLSMANRGKHTN
XP_021503062	HRVVKNFMIQGGDFSEGNGKGGESIYGGYFK-----DENFILKHDRAFLLSMANRGKHTN
XP_021503063	HRVVKNFMIQGGDFSEGNGKGGESIYGGYFK-----DENFILKHDRAFLLSMANRGKHTN

XP_008568057	HRVVKNFMIQGGDFSEGNGKGGESIYGGFFK-----DENFILKHDRAFLLSMANRGKHTN
XP_008568056	HRVVKNFMIQGGDFSEGNGKGGESIYGGFFK-----DENFILKHDRAFLLSMANRGKHTN
XP_006120664	HRVVKNFMIQGGDFSEGNGKGGESIYGGYFK-----DENFVLKHDRAFLLSMANRGKHTN
XP_007055629	HRVVKNFMIQGGDFSEGNGKGGESIYGGYFK-----DENFVLKHDRAFLLSMANRGKHTN
XP_006120661	HRVVKNFMIQGGDFSEGNGKGGESIYGGYFK-----DENFVLKHDRAFLLSMANRGKHTN
XP_006120659	HRVVKNFMIQGGDFSEGNGKGGESIYGGYFK-----DENFVLKHDRAFLLSMANRGKHTN
XP_007106819	HRVVKNFMIQGGDFSEGNGKGGESIYGGYFKEACSQDENFILKHDRAFLLSMANRGKHTN
XP_015708506	HRVVKNFMIQGGDFSEGNGKGGESIYGGYFK-----DENFILKHDRAFLLSMANRGKHTN
XP_009682494	HRVVKNFMIQGGDFSEGNGKGGESIYGGYFK-----DENFILKHDRAFLLSMANRGKHTN
XP_009682493	HRVVKNFMIQGGDFSEGNGKGGESIYGGYFK-----DENFILKHDRAFLLSMANRGKHTN
XP_009682492	HRVVKNFMIQGGDFSEGNGKGGESIYGGYFK-----DENFILKHDRAFLLSMANRGKHTN
XP_009682491	HRVVKNFMIQGGDFSEGNGKGGESIYGGYFK-----DENFILKHDRAFLLSMANRGKHTN
XP_019471734	HRVVKNFMIQGGDFSEGNGKGGESIYGGYFK-----DENFILKHDRAFLLSMANRGKHTN
XP_010710549	HRVVKNFMIQGGDFSEGNGKGGESIYGGYFK-----DENFILKHDRAFLLSMANRGKHTN
XP_010710548	HRVVKNFMIQGGDFSEGNGKGGESIYGGYFK-----DENFILKHDRAFLLSMANRGKHTN
XP_015136632	HRVVKNFMIQGGDFSEGNGKGGESIYGGYFK-----DENFILKHDRAFLLSMANRGKHTN
XP_004939132	HRVVKNFMIQGGDFSEGNGKGGESIYGGYFK-----DENFILKHDRAFLLSMANRGKHTN
XP_004939131	HRVVKNFMIQGGDFSEGNGKGGESIYGGYFK-----DENFILKHDRAFLLSMANRGKHTN
XP_013795593	HRVVKNFMIQGGDFSEGNGKGGESIYGGYFK-----DENFILKHDRAFLLSMANRGKHTN
XP_019363610	HRVVKNFMIQGGDFSEGNGKGGESIYGGYFK-----DENFILKHDRAFLLSMANRGKHTN
XP_014376468	HRVVKNFMIQGGDFSEGNGKGGESIYGGYFK-----DENFILKHDRAFLLSMANRGKHTN
XP_007659513	HRVVKNFMIQGGDFSEGNGKGGESIYGGYFK-----DENFVLKHDRAFLLSMANRGKHTN
XP_021241246	HRVVKNFMIQGGDFSEGNGKGGESIYGGYFK-----DENFILKHDRAFLLSMANRGKHTN
XP_021241236	HRVVKNFMIQGGDFSEGNGKGGESIYGGYFK-----DENFILKHDRAFLLSMANRGKHTN
XP_021241235	HRVVKNFMIQGGDFSEGNGKGGESIYGGYFK-----DENFILKHDRAFLLSMANRGKHTN
XP_017731869	HRVVKNFMIQGGDFSEGNGKGGESIYGGYFK-----DENFILKHDRAFLLSMANRGKHTN
XP_012426793	HRVVKNFMIQGGDFSEGNGKGGESIYGGYFK-----DENFILKHDRAFLLSMANRGKHTN
XP_017944741	HRVVKNFMIQGGDFSEGNGKGGESIYGGYFK-----DENFILKHDRAFLLSMANRGKHTN
XP_017944740	HRVVKNFMIQGGDFSEGNGKGGESIYGGYFK-----DENFILKHDRAFLLSMANRGKHTN
XP_010393085	HRVVKNFMIQGGDFSEGNGKGGESIYGGYFK-----DENFILKHDRAFLLSMANRGKHTN
XP_017685518	HRVVKNFMIQGGDFSEGNGKGGESIYGGYFK-----DENFILKHDRAFLLSMANRGKHTN
XP_017602246	HRVVKNFMIQGGDFSEGNGKGGESIYGGYFK-----DENFILKHDRAFLLSMANRGKHTN
XP_016151723	HRVVKNFMIQGGDFSEGNGKGGESIYGGYFK-----DENFILKHDRAFLLSMANRGKHTN
XP_019138709	HRVVKNFMIQGGDFSEGNGKGGESIYGGYFK-----DENFILKHDRAFLLSMANRGKHTN
XP_017685517	HRVVKNFMIQGGDFSEGNGKGGESIYGGYFK-----DENFILKHDRAFLLSMANRGKHTN
XP_017602245	HRVVKNFMIQGGDFSEGNGKGGESIYGGYFK-----DENFILKHDRAFLLSMANRGKHTN
XP_005040539	HRVVKNFMIQGGDFSEGNGKGGESIYGGYFK-----DENFILKHDRAFLLSMANRGKHTN
XP_005040538	HRVVKNFMIQGGDFSEGNGKGGESIYGGYFK-----DENFILKHDRAFLLSMANRGKHTN
XP_005040537	HRVVKNFMIQGGDFSEGNGKGGESIYGGYFK-----DENFILKHDRAFLLSMANRGKHTN
XP_014800495	HRVVKNFMIQGGDFSEGNGKGGESIYGGYFK-----DENFILKHDRAFLLSMANRGKHTN
XP_008168983	HRVVKNFMIQGGDFSEGNGKGGESIYGGYFK-----DENFVLKHDRAFLLSMANRGKHTN
XP_017944739	HRVVKNFMIQGGDFSEGNGKGGESIYGGYFK-----DENFILKHDRAFLLSMANRGKHTN
XP_014729203	HRVVKNFMIQGGDFSEGNGKGGESIYGGYFK-----DENFILKHDRAFLLSMANRGKHTN
XP_017944736	HRVVKNFMIQGGDFSEGNGKGGESIYGGYFK-----DENFILKHDRAFLLSMANRGKHTN
XP_014729201	HRVVKNFMIQGGDFSEGNGKGGESIYGGYFK-----DENFILKHDRAFLLSMANRGKHTN
XP_010393084	HRVVKNFMIQGGDFSEGNGKGGESIYGGYFK-----DENFILKHDRAFLLSMANRGKHTN
XP_017944735	HRVVKNFMIQGGDFSEGNGKGGESIYGGYFK-----DENFILKHDRAFLLSMANRGKHTN
XP_017685520	HRVVKNFMIQGGDFSEGNGKGGESIYGGYFK-----DENFILKHDRAFLLSMANRGKHTN
XP_017602244	HRVVKNFMIQGGDFSEGNGKGGESIYGGYFK-----DENFILKHDRAFLLSMANRGKHTN
XP_014729200	HRVVKNFMIQGGDFSEGNGKGGESIYGGYFK-----DENFILKHDRAFLLSMANRGKHTN
XP_010393082	HRVVKNFMIQGGDFSEGNGKGGESIYGGYFK-----DENFILKHDRAFLLSMANRGKHTN
XP_017685515	HRVVKNFMIQGGDFSEGNGKGGESIYGGYFK-----DENFILKHDRAFLLSMANRGKHTN
XP_017602242	HRVVKNFMIQGGDFSEGNGKGGESIYGGYFK-----DENFILKHDRAFLLSMANRGKHTN
XP_017602241	HRVVKNFMIQGGDFSEGNGKGGESIYGGYFK-----DENFILKHDRAFLLSMANRGKHTN
KFP58102	HRVVKNFMIQGGDFSEGNGKGGESIYGGYFK-----DENFILKHDRAFLLSMANRGKHTN
XP_010393081	HRVVKNFMIQGGDFSEGNGKGGESIYGGYFK-----DENFILKHDRAFLLSMANRGKHTN
XP_017685514	HRVVKNFMIQGGDFSEGNGKGGESIYGGYFK-----DENFILKHDRAFLLSMANRGKHTN
XP_017602240	HRVVKNFMIQGGDFSEGNGKGGESIYGGYFK-----DENFILKHDRAFLLSMANRGKHTN

XP_010559721	HRVVKNFMIQGGDFSEGNGKGGESIYGGYFK-----DENFILKHDRAFLLSMANRGKHTN
XP_014800494	HRVVKNFMIQGGDFSEGNGKGGESIYGGYFK-----DENFILKHDRAFLLSMANRGKHTN
XP_011571332	HRVVKNFMIQGGDFSEGNGKGGESIYGGYFK-----DENFILKHDRAFLLSMANRGKHTN
XP_010559712	HRVVKNFMIQGGDFSEGNGKGGESIYGGYFK-----DENFILKHDRAFLLSMANRGKHTN
XP_009331567	HRVVKNFMIQGGDFSEGNGKGGESIYGGYFK-----DENFILKHDRAFLLSMANRGKHTN
XP_008492672	HRVVKNFMIQGGDFSEGNGKGGESIYGGYFK-----DENFILKHDRAFLLSMANRGKHTN
XP_014800492	HRVVKNFMIQGGDFSEGNGKGGESIYGGYFK-----DENFILKHDRAFLLSMANRGKHTN
XP_011571324	HRVVKNFMIQGGDFSEGNGKGGESIYGGYFK-----DENFILKHDRAFLLSMANRGKHTN
XP_009697766	HRVVKNFMIQGGDFSEGNGKGGESIYGGYFK-----DENFILKHDRAFLLSMANRGKHTN
XP_009331566	HRVVKNFMIQGGDFSEGNGKGGESIYGGYFK-----DENFILKHDRAFLLSMANRGKHTN
XP_009274213	HRVVKNFMIQGGDFSEGNGKGGESIYGGYFK-----DENFILKHDRAFLLSMANRGKHTN
XP_014800491	HRVVKNFMIQGGDFSEGNGKGGESIYGGYFK-----DENFILKHDRAFLLSMANRGKHTN
XP_010301634	HRVVKNFMIQGGDFSEGNGKGGESIYGGYFK-----DENFILKHDRAFLLSMANRGKHTN
XP_008168980	HRVVKNFMIQGGDFSEGNGKGGESIYGGYFK-----DENFVLKHDRAFLLSMANRGKHTN
XP_023963187	HRVVKNFMIQGGDFSEGNGKGGESIYGGYFK-----DENFVLKHDRAFLLSMANRGKHTN
XP_005150066	HRVVKNFMIQGGDFSEGNGKGGESIYGGYFK-----DENFILKHDRAFLLSMANRGKHTN
XP_009473612	HRVVKNFMIQGGDFSEGNGKGGESIYGGYFK-----DENFILKHDRAFLLSMANRGKHTN
XP_021393646	HRVVKNFMIQGGDFSEGNGKGGESIYGGYFK-----DENFILKHDRAFLLSMANRGKHTN
XP_021393642	HRVVKNFMIQGGDFSEGNGKGGESIYGGYFK-----DENFILKHDRAFLLSMANRGKHTN
XP_021393643	HRVVKNFMIQGGDFSEGNGKGGESIYGGYFK-----DENFILKHDRAFLLSMANRGKHTN
XP_009878793	HRVVKNFMIQGGDFSEGNGKGGESIYGGYFK-----DENFILKHDRAFLLSMANRGKHTN
XP_021393641	HRVVKNFMIQGGDFSEGNGKGGESIYGGYFK-----DENFILKHDRAFLLSMANRGKHTN
XP_018764676	HRVVKNFMIQGGDFSEGNGKGGESIYGGYFK-----DENFILKHDRAFLLSMANRGKHTN
XP_018764674	HRVVKNFMIQGGDFSEGNGKGGESIYGGYFK-----DENFILKHDRAFLLSMANRGKHTN
XP_018764673	HRVVKNFMIQGGDFSEGNGKGGESIYGGYFK-----DENFILKHDRAFLLSMANRGKHTN
KFV02250	HRVVKNFMIQGGDFSEGNGKGGESIYGGYFK-----DENFILKHDRAFLLSMANRGKHTN
XP_014125627	HRVVKNFMIQGGDFSEGNGKGGESIYGGYFK-----DENFILKHDRAFLLSMANRGKHTN
XP_010083448	HRVVKNFMIQGGDFSEGNGKGGESIYGGYFK-----DENFILKHDRAFLLSMANRGKHTN
XP_014125626	HRVVKNFMIQGGDFSEGNGKGGESIYGGYFK-----DENFILKHDRAFLLSMANRGKHTN
XP_014112250	HRVVKNFMIQGGDFSEGNGKGGESIYGGYFK-----DENFILKHDRAFLLSMANRGKHTN
XP_014112249	HRVVKNFMIQGGDFSEGNGKGGESIYGGYFK-----DENFILKHDRAFLLSMANRGKHTN
XP_014125625	HRVVKNFMIQGGDFSEGNGKGGESIYGGYFK-----DENFILKHDRAFLLSMANRGKHTN
XP_014112248	HRVVKNFMIQGGDFSEGNGKGGESIYGGYFK-----DENFILKHDRAFLLSMANRGKHTN
XP_005428264	HRVVKNFMIQGGDFSEGNGKGGESIYGGYFK-----DENFILKHDRAFLLSMANRGKHTN
XP_015474510	HRVVKNFMIQGGDFSEGNGKGGESIYGGYFK-----DENFILKHDRAFLLSMANRGKHTN
XP_023777798	HRVVKNFMIQGGDFSEGNGKGGESIYGGYFK-----DENFILKHDRAFLLSMANRGKHTN
XP_015474508	HRVVKNFMIQGGDFSEGNGKGGESIYGGYFK-----DENFILKHDRAFLLSMANRGKHTN
XP_023777797	HRVVKNFMIQGGDFSEGNGKGGESIYGGYFK-----DENFILKHDRAFLLSMANRGKHTN
XP_023777796	HRVVKNFMIQGGDFSEGNGKGGESIYGGYFK-----DENFILKHDRAFLLSMANRGKHTN
XP_015474507	HRVVKNFMIQGGDFSEGNGKGGESIYGGYFK-----DENFILKHDRAFLLSMANRGKHTN
XP_023777795	HRVVKNFMIQGGDFSEGNGKGGESIYGGYFK-----DENFILKHDRAFLLSMANRGKHTN
KFQ42818	HRVVKNFMIQGGDFSEGNGKGGESIYGGYFK-----DENFILKHDRAFLLSMANRGKHTN
XP_019355067	HRVVKNFMIQGGDFSEGNGKGGESIYGGYFK-----DENFILKHDRAFLLSMANRGKHTN
XP_010014722	HRVVKNFMIQGGDFSEGNGKGGESIYGGYFK-----DENFILKHDRAFLLSMANRGKHTN
XP_009556564	HRVVKNFMIQGGDFSEGNGKGGESIYGGYFK-----DENFILKHDRAFLLSMANRGKHTN
XP_009993002	HRVVKNFMIQGGDFSEGNGKGGESIYGGYFK-----DENFILKHDRAFLLSMANRGKHTN
XP_009896433	HRVVKNFMIQGGDFSEGNGKGGESIYGGYFN-----DENFILKHDRAFLLSMANRGKHTN
XP_010213065	HRVVKNFMIQGGDFSEGNGKGGESIYGGYFK-----DENFILKHDRAFLLSMANRGKHTN
XP_019384731	HRVVKNFMIQGGDFSEGNGKGGESIYGGYFK-----DENFILKHDRAFLLSMANRGKHTN
XP_008110438	HRVVKNFMIQGGDFSEGNGKGGESIYGGYFK-----DENFILKHDRAFLLSMANRGKHTN
XP_008110436	HRVVKNFMIQGGDFSEGNGKGGESIYGGYFK-----DENFILKHDRAFLLSMANRGKHTN
XP_015264278	HRVVKNFMIQGGDFSEGNGKGGESIYGGYFK-----DENFILKHDRAFLLSMANRGKHTN
XP_013917336	HRVVKSFIIQGGDFSEGNGKGGESIYGGYFK-----DENFILKHDRAFLLSMANRGKHTN
XP_020670421	HRVVKNFMIQGGDFSEGNGKGGESIYGGYFK-----DENFILKHDRAFLLSMANRGKHTN
XP_020670420	HRVVKNFMIQGGDFSEGNGKGGESIYGGYFK-----DENFILKHDRAFLLSMANRGKHTN
XP_015667896	HRVVKSFIIQGGDFSEGNGKGGESIYGGYFK-----DENFILKHDRAFLLSMANRGKHTN
XP_007443413	HRVVKSFIIQGGDFSEGNGKGGESIYGGYFK-----DENFILKHDRAFLLSMANRGKHTN
XP_007443411	HRVVKSFIIQGGDFSEGNGKGGESIYGGYFK-----DENFILKHDRAFLLSMANRGKHTN

XP_017451684	HRVVKNFMIQGGDFSEGNGKGGESIYGGYFK-----DENFILKHDRAFLLSMANRGKHTN
XP_014918330	HRVVKNFMIQGGDFSEGNGKGGESIYGGYFK-----DENFILKHDRAFLLSMANRGKHTN
XP_002939352	HRVVKNFMIQGGDFSEGNGKGGESIYGGYFK-----DENFILKHDRAFLLSMANRGKNTN
XP_018124467	HRVVKNFMIQGGDFSEGNGKGGESIYGSYFR-----DENFILKHDRAFLLSMANRGENTN
XP_017572959	HRVVKNFMIQGGDFTEGNGRGGESIYGGYFE-----DENFILKHDRAFLLSMANRGKDTN
XP_018607018	HRVVKNFMIQGGDFTEGNGKGGESIYGGYFE-----DENFTLKHDRAFLLSMANRGKDTN
XP_018607016	HRVVKNFMIQGGDFTEGNGKGGESIYGGYFE-----DENFTLKHDRAFLLSMANRGKDTN
XP_007544596	HRVVKNFMIQGGDFTEGTGRGGESIYGGYFE-----DENFILKHDKAFLLSMANRGKNTN
XP_018607022	HRVVKNFMIQGGDFTEGNGKGGESIYGGYFE-----DENFTLKHDRAFLLSMANRGKDTN
XP_009443552	HRVVKNFMIQGGDFSEGNGKGGESIYGGYFK-----DENFILKHDRAFLLSMANRGKHTN
PNJ74568	HRVVKNFMIQGGDFSEGNGKGGESIYGGYFK-----DENFILKHDRAFLLSMANRGKHTN
PNJ74566	HRVVKNFMIQGGDFSEGNGKGGESIYGGYFK-----DENFILKHDRAFLLSMANRGKHTN
XP_018122561	HRVVKNFMIQGGDFSEGNGKGGESIYGGYFK-----DENFILKHDRAFLLSMANRGKNTN
XP_012735608	HRVVKKFMIQGGDFTEGNGRGGESIYGGYFE-----DENFILKHDRAFLLSMANRGKNTN
XP_022065410	HRVVKNFMIQGGDFTEGNGRGGESIYGGYFE-----DENFTLKHDRAFLLSMANRGKDTN
XP_023122049	HRVVKNFMIQGGDFTEGNGRGGESIYGGYFE-----DENFTLKHDRAFLLSMANRGKDTN
XP_022065408	HRVVKNFMIQGGDFTEGNGRGGESIYGGYFE-----DENFTLKHDRAFLLSMANRGKDTN
XP_023122045	HRVVKNFMIQGGDFTEGNGRGGESIYGGYFE-----DENFTLKHDRAFLLSMANRGKDTN
XP_022065407	HRVVKNFMIQGGDFTEGNGRGGESIYGGYFE-----DENFTLKHDRAFLLSMANRGKDTN
XP_022065406	HRVVKNFMIQGGDFTEGNGRGGESIYGGYFE-----DENFTLKHDRAFLLSMANRGKDTN
XP_023122044	HRVVKNFMIQGGDFTEGNGRGGESIYGGYFE-----DENFTLKHDRAFLLSMANRGKDTN
XP_022065405	HRVVKNFMIQGGDFTEGNGRGGESIYGGYFE-----DENFTLKHDRAFLLSMANRGKDTN
XP_023122043	HRVVKNFMIQGGDFTEGNGRGGESIYGGYFE-----DENFTLKHDRAFLLSMANRGKDTN
XP_018414151	HRVVKHFMIQGGDFSEGNGKGGESIYGGFFK-----DENFILKHDRAFLLSMANRGKHTN
XP_023490562	HRVVKNFMIQGGDFSEGNGKGGESIYGGYFK-----DENFILKHDRAFLLSMANRGKHTN
XP_008304546	HRVVKNFMIQGGDFTEGNGRGGESIYGGYFE-----DENFTLKHDRAFLLSMANRGKDTN
KKF16876	HRVVKNFMVQGGDFTEGNGRGGESIYGGYFE-----DENFTLKHDRAFLLSMANRGKDTN
XP_008304545	HRVVKNFMIQGGDFTEGNGRGGESIYGGYFE-----DENFTLKHDRAFLLSMANRGKDTN
XP_015249666	HRVVKNFMVQGGDFTEGNGRGGESIYGGYFE-----DENFILKHDRAFLLSMANRGKDTN
XP_018529332	HRVVKNFMIQGGDFTEGNGRGGESIYGGYFE-----DENFTLKHDRAFLLSMANRGKDTN
XP_018529331	HRVVKNFMIQGGDFTEGNGRGGESIYGGYFE-----DENFTLKHDRAFLLSMANRGKDTN
XP_010751380	HRVVKNFMVQGGDFTEGNGRGGESIYGGYFE-----DENFTLKHDRAFLLSMANRGKDTN
XP_010751371	HRVVKNFMVQGGDFTEGNGRGGESIYGGYFE-----DENFTLKHDRAFLLSMANRGKDTN
XP_019745550	HRVVKNFMIQGGDFTEGNGRGGESIYGGYFE-----DENLTLKHDRAFLLSMANRGKDTN
XP_010751365	HRVVKNFMVQGGDFTEGNGRGGESIYGGYFE-----DENFTLKHDRAFLLSMANRGKDTN
XP_019745545	HRVVKNFMIQGGDFTEGNGRGGESIYGGYFE-----DENLTLKHDRAFLLSMANRGKDTN
XP_023263705	HRVVKNFMVQGGDFTEGNGRGGESIYGGYFE-----DENFTLKHDRAFLLSMANRGKDTN
XP_023263706	HRVVKNFMVQGGDFTEGNGRGGESIYGGYFE-----DENFTLKHDRAFLLSMANRGKDTN
XP_001338072	HRVVKNFMIQGGDFTEGNGRGGESIFGGFFE-----DENFTLKHDRAFLLSMANRGKDTN
XP_022599954	HRVVKNFMVQGGDFTEGNGRGGESIYGGYFE-----DENFTLKHDRAFLLSMANRGKDTN
XP_019897364	HRVVKNFMIQGGDFTEGNGRGGESIFGGYFE-----DENFILKHDRAFLLSMANRGKHTN
XP_010900666	HRVVKNFMIQGGDFTEGNGRGGESIFGGYFE-----DENFILKHDRAFLLSMANRGKHTN
XP_010900665	HRVVKNFMIQGGDFTEGNGRGGESIFGGYFE-----DENFILKHDRAFLLSMANRGKHTN
XP_010900664	HRVVKNFMIQGGDFTEGNGRGGESIFGGYFE-----DENFILKHDRAFLLSMANRGKHTN
XP_019897363	HRVVKNFMIQGGDFTEGNGRGGESIFGGYFE-----DENFILKHDRAFLLSMANRGKHTN
XP_010167755	HRVVKNFMIQGGDFSEGNGKGGESIYGGYFK-----DENFILKHDRAFLLSMANRGKHTN
XP_010900668	HRVVKNFMIQGGDFTEGNGRGGESIFGGYFE-----DENFILKHDRAFLLSMANRGKHTN
XP_016427080	HRVVKNFMIQGGDFTEGNGRGGESIYGGFFE-----DENFTLKHDRAFLLSMANRGKDTN
XP_016318510	HRVVKNFMIQGGDFTEGNGRGGESIYGGFFE-----DENFTLKHDRAFLLSMANRGKDTN
XP_015209664	HRVVKSFMIQGGDFTEGNGRGGESIFGGYFE-----DENFSLKHDRAFLLSMANRGKDTN
XP_015209662	HRVVKSFMIQGGDFTEGNGRGGESIFGGYFE-----DENFSLKHDRAFLLSMANRGKDTN
XP_015209665	HRVVKSFMIQGGDFTEGNGRGGESIFGGYFE-----DENFSLKHDRAFLLSMANRGKDTN
XP_005912346	HRVVKNFMIQGGDFTEGNGRGGESIYGGYFE-----DENFTLKHDRAFLLSMANRGKDTN
XP_005722738	HRVVKNFMIQGGDFTEGNGRGGESIYGGYFE-----DENFTLKHDRAFLLSMANRGKDTN
XP_020457914	HRVVKNFMIQGGDFTEGNGRGGESIYGGYFE-----DENFILKHDRAFLLSMANRGKNTN
XP_013132609	HRVVKNFMIQGGDFTEGNGRGGESIYGGYFE-----DENFTLKHDRAFLLSMANRGKDTN
XP_004546791	HRVVKNFMIQGGDFTEGNGRGGESIYGGYFE-----DENFTLKHDRAFLLSMANRGKDTN
XP_006795195	HRVVKNFMIQGGDFTEGNGRGGESIYGGYFE-----DENFTLKHDRAFLLSMANRGKDTN

```
XP_005912345        HRVVKNFMIQGGDFTEGNGRGGESIYGGYFE-----DENFTLKHDRAFLLSMANRGKDTN
XP_005722737        HRVVKNFMIQGGDFTEGNGRGGESIYGGYFE-----DENFTLKHDRAFLLSMANRGKDTN
XP_021475699        HRVVKNFMIQGGDFTEGNGRGGESIYGGYFE-----DENFILKHDRAFLLSMANRGKHTN
XP_004546790        HRVVKNFMIQGGDFTEGNGRGGESIYGGYFE-----DENFTLKHDRAFLLSMANRGKDTN
XP_003439241        HRVVKNFMIQGGDFTEGNGRGGESIYGGYFE-----DENFTLKHDRAFLLSMANRGKDTN
XP_006795194        HRVVKNFMIQGGDFTEGNGRGGESIYGGYFE-----DENFTLKHDRAFLLSMANRGKDTN
XP_023490560        HRVVKNFMIQGGDFSEGNGKGGESIYGGYFK-----DENFILKHDRAFLLSMANRGKHTN
XP_015209666        HRVVKSFMIQGGDFTEGNGRGGESIFGGYFE-----DENFSLKHDRAFLLSMANRGKDTN
XP_005797888        HRVVKNFMIQGGDFTEGTGRGGESIYGGYFE-----DENFILKHDKAFLLSMANRGKNTN
CBN81984            HRVVKNFMIQGGDFTEGNGRGGESIYGGYFE-----DENFILKHDRPFLLSMANRGKDTN
XP_010559731        HRVVKNFMIQGGDFSEGNGKGGESIYGGYFK-----DENFILKHDRAFLLSMANRGKHTN
XP_007544415        HRVVKNFMIQGGDFTEGTGRGGESIYGGYFE-----DENFILKHDKAFLLSMANRGKNTN
XP_014870682        HRVVKNFMIQGGDFTEGTGRGGESIYGGYFE-----DENFILKHDKAFLLSMANRGKNTN
XP_011606242        HRVVKNFMIQGGDFTEGNGRGGESIYGGFFE-----DENFVLKHDRAFLLSMANRGKDTN
CAG04245            HRVVKNFMIQGGDFTEGNGRGGESIYGGYFE-----DENFVLKHDSAFLLSMANRGKDTN
XP_016887118        HRVVKNFMIQGGDFTEGNGRGGESIYGGYFE-----DENFTLKHDKAFLLSMANRGKDTN
XP_008306161        HRVVKNFMIQGGDFTEGNGRGGESIYGGYFE-----DENFTLKHDKAFLLSMANRGKDTN
XP_008394441        HRVVKNFMIQGGDFTEGTGRGGESIYGGYFE-----DENFILKHDKAFLLSMANRGKNTN
XP_014825423        HRVVKNFMIQGGDFTEGTGRGGESIYGGYFE-----DENFILKHDKAFLLSMANRGKNTN
XP_012398954        HRVVKNFMIQGGDFSEGNGKGGESIYGGYFK-----DENFVLKHDRAFLLSMANRGKHTN
XP_019063096        HRVVKNFMIQGGDFSEGNGKGGESIYGGYFK-----DENFILKHDRAFLLSMANRGKHTN
XP_009073803        HRVVKNFMIQGGDFSEGNGKGGESIYGGYFK-----DENFILKHDRAFLLSMANRGKHTN
XP_016400109        HRVVKNFMIQGGDFTEGNGRGGESIYGGFFE-----DENFTLKHDRAFLLSMANRGKDTN
XP_016359192        HRVVKNFMIQGGDFTEGNGRGGESIYGGFFE-----DENFTLKHDRAFLLSMANRGKDTN
XP_004081327        HRVVKNFMIQGGDFTEGTGRGGESIYGGYFK-----DENFILKHDRAFLLSMANRGKDTN
XP_020568300        HRVVKNFMIQGGDFTEGTGRGGESIYGGYFK-----DENFILKHDRAFLLSMANRGKDTN
XP_016041020        HRVVKNFMIQGGDFSEGNGKGGESIYGGYFK-----DENFILKHDRAFLLSMANRGKHTN
XP_017942181        HRVVKNFMIQGGDFSEGNGKGGESIYGGYFK-----DENFILKHDRAFLLSMANRGKHTN
XP_017942183        HRVVKNFMIQGGDFSEGNGKGGESIYGGYFK-----DENFILKHDRAFLLSMANRGKHTN
XP_017942182        HRVVKNFMIQGGDFSEGNGKGGESIYGGYFK-----DENFILKHDRAFLLSMANRGKHTN
XP_020501265        HRVVKNFMVQGGDFTEGNGRGGESIYGGYFE-----DENFTLKHDRPFLLSMANRGKDTN
KFP01329            HRVVKNFMIQGGDFSEGNGKGGESIYGGYFK-----DENFILKHDRAFLLSMANRGKHTN
XP_015856948        HRVVKNFMIQGGDFSEGNGKGGESIYGGYFK-----DENFILKHDRAFLLSMANRGKHTN
XP_020139631        HRVVKNFMIQGGDFSEGNGKGGESIYGGYFK-----DENFILKHDRAFLLSMANRGKHTN
XP_020139628        HRVVKNFMIQGGDFSEGNGKGGESIYGGYFK-----DENFILKHDRAFLLSMANRGKHTN
XP_006982975        HRVVKNFMIQGGDFSEGNGKGGESIYGGYFK-----DENFILKHDRAFLLSMANRGKHTN
XP_015856947        HRVVKNFMIQGGDFSEGNGKGGESIYGGYFK-----DENFILKHDRAFLLSMANRGKHTN
XP_014427753        HRVVKNFMIQGGDFSEGNGKGGESIYGGYFK-----DENFVLKHDRAFLLSMANRGKHTN
XP_013857648        HRVVKNFMIQGGDFTEGNGRGGESIYGGYFE-----DENFILKHNRPFLLSMANRGKNTN
XP_023656013        HRVVKNFMIQGGDFTEGNGRGGESIYGGYFE-----DENFKLKHDRAFLLSMANRGKDTN
XP_023656011        HRVVKNFMIQGGDFTEGNGRGGESIYGGYFE-----DENFKLKHDRAFLLSMANRGKDTN
XP_023656014        HRVVKNFMIQGGDFTEGNGRGGESIYGGYFE-----DENFKLKHDRAFLLSMANRGKDTN
XP_004448689        HRVVKNFMIQGGDFSEGNGKGGESIYGGYFK-----DENFILKHDRAFLLSMANRGKHTN
XP_004448688        HRVVKNFMIQGGDFSEGNGKGGESIYGGYFK-----DENFILKHDRAFLLSMANRGKHTN
XP_023476440        HRVVKNFMIQGGDFSEGNGKGGESIYGGYFK-----DENFILKHDRAFLLSMANRGKHTN
                    ***** *::*****:**.*:*****:*.:*.     ***: ***: .*********:.**

NP_005376           GSQFFITTKPAPHLDGVHVVFGLVISGFEVIEQIENLKTDAASRPYADVRVIDCGVLA
XP_014646452        GSQFFITTKPAPHLDGVHVVFGLVISGFEVIEQIENLKTDAASRPYADVRVIDCGVLA
XP_017657797        GSQFFITTKPAPHLDGVHVVFGLVISGFEVIEQIENLKTDAASRPYADVRVIDCGVLA
XP_012885690        GSQFFITTKPAPHLDGVHVVFGLVISGFEVIEQIENLKTDAASRPYADVRVIDCGVLA
XP_008530969        GSQFFITTKPAPHLDGVHVVFGLVISGFEVIEQIENLKTDAASRPYADVRVIDCGVLA
XP_015423871        GSQFFITTKPAPHLDGVHVVFGLVISGFEVIEQIENLKTDAASRPYADVRVIDCGVLA
XP_015091833        GSQFFITTKPAPHLDGVHVVFGLVISGFEVIEQIENLKTDAASRPYADVRVIDCGVLA
XP_010973060        GSQFFITTKPAPHLDGVHVVFGLVISGFEVIEQIENLKTDAASRPYADVRVIDCGVLA
XP_019583096        GSQFFITTKPAPHLDGVHVVFGLVISGFEVIEQIENLKTDAASRPYADVRVIDCGVLA
XP_011231111        GSQFFITTKPAPHLDGVHVVFGLVISGFEVIEQIENLKTDAASRPYADVRVIDCGVLA
XP_014646457        GSQFFITTKPAPHLDGVHVVFGLVISGFEVIEQIENLKTDAASRPYADVRVIDCGVLA
```

XP_023988069	GSQFFITTKPAPHLDGVHVVFGLVISGFEVIEQIENLKTDAASRPYADVRVIDCGVLA
XP_008530970	GSQFFITTKPAPHLDGVHVVFGLVISGFEVIEQIENLKTDAASRPYADVRVIDCGVLA
XP_017497119	GSQFFITTKPAPHLDGVHVVFGLVISGFEVIEQIENLKTDAASRPYADVRVIDCGVLA
XP_021503072	GSQFFITTKPAPHLDGVHVVFGLVISGFEVIEQIENLKTDAASRPYADVRVIDCGVIA
XP_021503071	GSQFFITTKPAPHLDGVHVVFGLVISGFEVIEQIENLKTDAASRPYADVRVIDCGVIA
XP_020041828	GSQFFITTKPAPHLDGVHVVFGLVISGFEVIEQIENLKTDAASRPYADVRVIDCGVLA
XP_021503070	GSQFFITTKPAPHLDGVHVVFGLVISGFEVIEQIENLKTDAASRPYADVRVIDCGVIA
XP_012590780	GSQFFITTKPAPHLDGVHVVFGLVISGFEVIEQIENLKTDAASRPYADVRVIDCGVLA
XP_019274390	GSQFFITTKPAPHLDGVHVVFGLVISGFEVIEQIENLKTDAASRPYADVRVIDCGVLA
XP_019489071	GSQFFITTKPAPHLDGVHVVFGLVISGFEVIEQIENLKTDAASRPYADVRVIDCGVLA
XP_019489063	GSQFFITTKPAPHLDGVHVVFGLVISGFEVIEQIENLKTDAASRPYADVRVIDCGVLA
XP_010588206	GSQFFITTKPAPHLDGVHVVFGLVISGFEVIEQIENLKTDAASRPYADVRVIDCGVIA
XP_023403600	GSQFFITTKPAPHLDGVHVVFGLVISGFEVIEQIENLKTDAASRPYADVRVIDCGVIA
XP_009982730	GSQFFITTKPAPHLDGVHVVFGLVISGFEVIEQIENLKTDTASRPYADVRVIDCGVLV
XP_019839655	GSQFFITTKPAPHLDGVHVVFGLVISGFEVIEQIENLKTDAASRPYADVRVIDCGVLA
XP_006512034	GSQFFITTKPAPHLDGVHVVFGLVISGFEVIEQIENLKTDAASRPYADVRVIDCGVLA
XP_024100264	GSQFFITTKPAPHLDGVHVVFGLVISGFEVIEQIENLKTDAASRPYADVRVIDCGVLA
XP_019666422	GSQFFITTKPAPHLDGVHVVFGLVISGFEVIEQIENLKTDAASRPYADVRVIDCGVLA
XP_008530962	GSQFFITTKPAPHLDGVHVVFGLVISGFEVIEQIENLKTDAASRPYADVRVIDCGVLA
XP_008530967	GSQFFITTKPAPHLDGVHVVFGLVISGFEVIEQIENLKTDAASRPYADVRVIDCGVLA
XP_010944204	GSQFFITTKPAPHLDGVHVVFGLVISGFEVIEQIENLKTDAASRPYADVRVIDCGVLA
XP_015091830	GSQFFITTKPAPHLDGVHVVFGLVISGFEVIEQIENLKTDAASRPYADVRVIDCGVLA
XP_014411884	GSQFFITTKPAPHLDGVHVVFGLVISGFEVIEQIENLKTDAASRPYADVRVIDCGVLA
XP_010944201	GSQFFITTKPAPHLDGVHVVFGLVISGFEVIEQIENLKTDAASRPYADVRVIDCGVLA
XP_009443554	GSQFFITTKPAPHLDGVHVVFGLVISGFEVIEQIENLKTDAASRPYADVRVIDCGVLA
XP_021241242	GSQFFITTKPAPHLDGVHVVFGLVISGFEVIEQIENLKTDTASRPYADVRVIDCGVLV
XP_020927360	GSQFFITTKPAPHLDGVHVVFGLVISGFEVIEQIENLKTDAASRPYADVRVIDCGVLA
XP_004581770	GSQFFITTKPAPHLDGVHVVFGLVISGFEVIEQIENLKTDAASRPYADVRVIDCGVIA
XP_005669469	GSQFFITTKPAPHLDGVHVVFGLVISGFEVIEQIENLKTDAASRPYADVRVIDCGVLA
XP_009443555	GSQFFITTKPAPHLDGVHVVFGLVISGFEVIEQIENLKTDAASRPYADVRVIDCGVLA
XP_005669468	GSQFFITTKPAPHLDGVHVVFGLVISGFEVIEQIENLKTDAASRPYADVRVIDCGVLA
XP_008708042	GSQFFITTKPAPHLDGVHVVFGLVISGFEVIEQIENLKTDAASRPYADVRVIDCGVLA
AAA37500	GSQFFITTKPAPHLDGVHVVFGLVISGFEVIEQIENLKTDAASRPYADVRVIDCGVLA
XP_004676331	GSQFFITTKPAPHLDGVHVVFGLVISGFEVIEQIENLKTDAASRPYADVRVIDCGVLA
XP_006748020	GSQFFITTKPAPHLDGVHVVFGLVISGFEVIEQIENLKTDAASRPYADVRVIDCGVLA
XP_006770577	GSQFFITTKPAPHLDGVHVVFGLVISGFEVIEQIENLKTDAASRPYADVRVIDCGVLA
XP_019583095	GSQFFITTKPAPHLDGVHVVFGLVISGFEVIEQIENLKTDAASRPYADVRVIDCGVLA
XP_015423867	GSQFFITTKPAPHLDGVHVVFGLVISGFEVIEQIENLKTDAASRPYADVRVIDCGVLA
XP_005383594	GSQFFITTKPAPHLDGVHVVFGLVISGFEVIEQIENLKTDAASRPYADVRVIDCGVLA
XP_019583089	GSQFFITTKPAPHLDGVHVVFGLVISGFEVIEQIENLKTDAASRPYADVRVIDCGVLA
XP_014445953	GSQFFITTKPAPHLDGVHVVFGLVISGFEVIEQIENLKTDAASRPYADVRVIDCGVLA
XP_005891140	GSQFFITTKPAPHLDGVHVVFGLVISGFEVIEQIENLKTDAASRPYADVRVIDCGVLA
XP_019489104	GSQFFITTKPAPHLDGVHVVFGLVISGFEVIEQIENLKTDAASRPYADVRVIDCGVLA
XP_012004520	GSQFFITTKPAPHLDGVHVVFGLVISGFEVIEQIENLKTDAASRPYADVRVIDCGVLA
XP_006051863	GSQFFITTKPAPHLDGVHVVFGLVISGFEVIEQIENLKTDAASRPYADVRVIDCGVLA
XP_005954297	GSQFFITTKPAPHLDGVHVVFGLVISGFEVIEQIENLKTDAASRPYADVRVIDCGVLA
XP_005695639	GSQFFITTKPAPHLDGVHVVFGLVISGFEVIEQIENLKTDAASRPYADVRVIDCGVLA
XP_011954828	GSQFFITTKPAPHLDGVHVVFGLVISGFEVIEQIENLKTDAASRPYADVRVIDCGVLA
XP_003992255	GSQFFITTKPAPHLDGVHVVFGLVISGFEVIEQIENLKTDAASRPYADVRVIDCGVLA
XP_002702773	GSQFFITTKPAPHLDGVHVVFGLVISGFEVIEQIENLKTDAASRPYADVRVIDCGVLA
XP_019666421	GSQFFITTKPAPHLDGVHVVFGLVISGFEVIEQIENLKTDAASRPYADVRVIDCGVLA
XP_019489077	GSQFFITTKPAPHLDGVHVVFGLVISGFEVIEQIENLKTDAASRPYADVRVIDCGVLA
XP_012004518	GSQFFITTKPAPHLDGVHVVFGLVISGFEVIEQIENLKTDAASRPYADVRVIDCGVLA
XP_011954826	GSQFFITTKPAPHLDGVHVVFGLVISGFEVIEQIENLKTDAASRPYADVRVIDCGVLA
XP_006051860	GSQFFITTKPAPHLDGVHVVFGLVISGFEVIEQIENLKTDAASRPYADVRVIDCGVLA
XP_005695638	GSQFFITTKPAPHLDGVHVVFGLVISGFEVIEQIENLKTDAASRPYADVRVIDCGVLA
XP_005196454	GSQFFITTKPAPHLDGVHVVFGLVISGFEVIEQIENLKTDAASRPYADVRVIDCGVLA
XP_851976	GSQFFITTKPAPHLDGVHVVFGLVISGFEVIEQIENLKTDAASRPYADVRVIDCGVLA

XP_012520957	GSQFFITTKPAPHLDGVHVVFGLVISGFEVIEQIENLKTDAASRPYADVRVIDCGVLA
XP_005634360	GSQFFITTKPAPHLDGVHVVFGLVISGFEVIEQIENLKTDAASRPYADVRVIDCGVLA
XP_003132182	GSQFFITTKPAPHLDGVHVVFGLVISGFEVIEQIENLKTDAASRPYADVRVIDCGVLA
XP_020139644	GSQFFITTKPAPHLDGVHVVFGLVISGFEVIEQIENLKTDAASRPYADVRVIDCGVLA
XP_014715584	GSQFFITTKPAPHLDGVHVVFGLVISGFEVIEQIENLKTDAASRPYADVRVIDCGVLA
XP_007193513	GSQFFITTKPAPHLDGVHVVFGLVISGFEVIEQIENLKTDAASRPYADVRVIDCGVLA
XP_005669463	GSQFFITTKPAPHLDGVHVVFGLVISGFEVIEQIENLKTDAASRPYADVRVIDCGVLA
XP_004419619	GSQFFITTKPAPHLDGVHVVFGLVISGFEVIEQIENLKTDAASRPYADVRVIDCGVLA
XP_003363180	GSQFFITTKPAPHLDGVHVVFGLVISGFEVIEQIENLKTDAASRPYADVRVIDCGVLA
XP_014986734	GSQFFITTKPAPHLDGVHVVFGLVISGFEVIEQIENLKTDAASRPYADVRVIDCGVLA
XP_014715581	GSQFFITTKPAPHLDGVHVVFGLVISGFEVIEQIENLKTDAASRPYADVRVIDCGVLA
XP_014646446	GSQFFITTKPAPHLDGVHVVFGLVISGFEVIEQIENLKTDAASRPYADVRVIDCGVLA
XP_006893048	GSQFFITTKPAPHLDGVHVVFGLVISGFEVIEQIENLKTDAASRPYADVRVIDCGVLA
XP_012625718	GSQFFITTKPAPHLDGVHVVFGLVISGFEVIEQIENLKTDAASRPYADVRVIDCGVLA
XP_010973061	GSQFFITTKPAPHLDGVHVVFGLVISGFEVIEQIENLKTDAASRPYADVRVIDCGVLA
XP_010944206	GSQFFITTKPAPHLDGVHVVFGLVISGFEVIEQIENLKTDAASRPYADVRVIDCGVLA
XP_007193512	GSQFFITTKPAPHLDGVHVVFGLVISGFEVIEQIENLKTDAASRPYADVRVIDCGVLA
XP_015091829	GSQFFITTKPAPHLDGVHVVFGLVISGFEVIEQIENLKTDAASRPYADVRVIDCGVLA
XP_014411882	GSQFFITTKPAPHLDGVHVVFGLVISGFEVIEQIENLKTDAASRPYADVRVIDCGVLA
XP_006770574	GSQFFITTKPAPHLDGVHVVFGLVISGFEVIEQIENLKTDAASRPYADVRVIDCGVLA
XP_006770573	GSQFFITTKPAPHLDGVHVVFGLVISGFEVIEQIENLKTDAASRPYADVRVIDCGVLA
XP_003809334	GSQFFITTKPAPHLDGVHVVFGLVISGFEVIEQIENLKTDAASRPYADVRVIDCGVLA
ELK25111	GSQFFITTKPAPHLDGVHVVFGLVISGFEVIEQIENLKTDAASRPYADVRVIDCGVLA
XP_023087260	GSQFFITTKPAPHLDGVHVVFGLVISGFEVIEQIENLKTDAASRPYADVRVIDCGVLA
XP_005546833	GSQFFITTKPAPHLDGVHVVFGLVISGFEVIEQIENLKTDAASRPYADVRVIDCGVLA
XP_008258471	GSQFFITTKPAPHLDGVHVVFGLVISGFEVIEQIENLKTDAASRPYADVRVIDCGVLA
XP_008258470	GSQFFITTKPAPHLDGVHVVFGLVISGFEVIEQIENLKTDAASRPYADVRVIDCGVLA
XP_004708608	GSQFFITTKPAPHLDGVHVVFGLVISGFEVIEQIENLKTDAASRPYADVRVIDCGVIA
XP_003309783	GSQFFITTKPAPHLDGVHVVFGLVISGFEVIEQIENLKTDAASRPYADVRVIDCGVLA
XP_009443557	GSQFFITTKPAPHLDGVHVVFGLVISGFEVIEQIENLKTDAASRPYADVRVIDCGVLA
XP_011889503	GSQFFITTKPAPHLDGVHVVFGLVISGFEVIEQIENLKTDAASRPYADVRVIDCGVLA
XP_019274386	GSQFFITTKPAPHLDGVHVVFGLVISGFEVIEQIENLKTDAASRPYADVRVIDCGVLA
XP_015392541	GSQFFITTKPAPHLDGVHVVFGLVISGFEVIEQIENLKTDAASRPYADVRVIDCGVLA
XP_021573498	GSQFFITTKPAPHLDGVHVVFGLVISGFEVIEQIENLKTDAASRPYADVRVIDCGVLA
XP_017396461	GSQFFITTKPAPHLDGVHVVFGLVISGFEVIEQIENLKTDAASRPYADVRVIDCGVLA
XP_005546834	GSQFFITTKPAPHLDGVHVVFGLVISGFEVIEQIENLKTDAASRPYADVRVIDCGVLA
XP_014986732	GSQFFITTKPAPHLDGVHVVFGLVISGFEVIEQIENLKTDAASRPYADVRVIDCGVLA
XP_018879079	GSQFFITTKPAPHLDGVHVVFGLVISGFEVIEQIENLKTDAASRPYADVRVIDCGVLA
XP_014986735	GSQFFITTKPAPHLDGVHVVFGLVISGFEVIEQIENLKTDAASRPYADVRVIDCGVLA
XP_009443553	GSQFFITTKPAPHLDGVHVVFGLVISGFEVIEQIENLKTDAASRPYADVRVIDCGVLA
XP_016861963	GSQFFITTKPAPHLDGVHVVFGLVISGFEVIEQIENLKTDAASRPYADVRVIDCGVLA
XP_011839703	GSQFFITTKPAPHLDGVHVVFGLVISGFEVIEQIENLKTDAASRPYADVRVIDCGVLA
XP_006713234	GSQFFITTKPAPHLDGVHVVFGLVISGFEVIEQIENLKTDAASRPYADVRVIDCGVLA
XP_017396460	GSQFFITTKPAPHLDGVHVVFGLVISGFEVIEQIENLKTDAASRPYADVRVIDCGVLA
XP_018879080	GSQFFITTKPAPHLDGVHVVFGLVISGFEVIEQIENLKTDAASRPYADVRVIDCGVLA
XP_010372621	GSQFFITTKPAPHLDGVHVVFGLVISGFEVIEQIENLKTDAASRPYADVRVIDCGVLA
XP_007981992	GSQFFITTKPAPHLDGVHVVFGLVISGFEVIEQIENLKTDAASRPYADVRVIDCGVLA
XP_009199351	GSQFFITTKPAPHLDGVHVVFGLVISGFEVIEQIENLKTDAASRPYADVRVIDCGVLA
PNJ74565	GSQFFITTKPAPHLDGVHVVFGLVISGFEVIEQIENLKTDAASRPYADVRVIDCGVLA
XP_023087259	GSQFFITTKPAPHLDGVHVVFGLVISGFEVIEQIENLKTDAASRPYADVRVIDCGVLA
XP_009237437	GSQFFITTKPAPHLDGVHVVFGLVISGFEVIEQIENLKTDAASRPYADVRVIDCGVLA
XP_023087256	GSQFFITTKPAPHLDGVHVVFGLVISGFEVIEQIENLKTDAASRPYADVRVIDCGVLA
XP_010372620	GSQFFITTKPAPHLDGVHVVFGLVISGFEVIEQIENLKTDAASRPYADVRVIDCGVLA
XP_004414069	GSQFFITTKPAPHLDGVHVVFGLVISGFEVIEQIENLKTDAASRPYADVRVIDCGVLA
XP_011231109	GSQFFITTKPAPHLDGVHVVFGLVISGFEVIEQIENLKTDAASRPYADVRVIDCGVLA
XP_015392540	GSQFFITTKPAPHLDGVHVVFGLVISGFEVIEQIENLKTDAASRPYADVRVIDCGVLA
XP_011231110	GSQFFITTKPAPHLDGVHVVFGLVISGFEVIEQIENLKTDAASRPYADVRVIDCGVLA
XP_015392538	GSQFFITTKPAPHLDGVHVVFGLVISGFEVIEQIENLKTDAASRPYADVRVIDCGVLA

XP_004754311	GSQFFITTKPAPHLDGVHVVFGLVISGFEVIEQIENLKTDAASRPYADVRVIDCGVLA
XP_011806342	GSQFFITTKPAPHLDGVHVVFGLVISGFEVIEQIENLKTDAASRPYADVRVIDCGVLA
XP_005546832	GSQFFITTKPAPHLDGVHVVFGLVISGFEVIEQIENLKTDAASRPYADVRVIDCGVLA
XP_005546835	GSQFFITTKPAPHLDGVHVVFGLVISGFEVIEQIENLKTDAASRPYADVRVIDCGVLA
XP_023384831	GSQFFITTKPAPHLDGVHVVFGLVISGFEVIEQIENLKTDAASRPYADVRVIDCGVLA
XP_015447191	GSQFFITTKPAPHLDGVHVVFGLVISGFEVIEQIENLKTDAASRPYADVRVIDCGVLA
XP_006868826	GSQFFITTKPAPHLDGVHVVFGLVISGFEVIEQIENLKTDAASRPYADVRVIDCGVIA
XP_006102363	GSQFFITTKPAPHLDGVHVVFGLVISGFEVIEQIENLKTDAASRPYADVRVIDCGVLA
XP_005860445	GSQFFITTKPAPHLDGVHVVFGLVISGFEVIEQIENLKTDAASRPYADVRVIDCGVLA
XP_019274385	GSQFFITTKPAPHLDGVHVVFGLVISGFEVIEQIENLKTDAASRPYADVRVIDCGVLA
XP_014323127	GSQFFITTKPAPHLDGVHVVFGLVISGFEVIEQIENLKTDAASRPYADVRVIDCGVLA
XP_005860444	GSQFFITTKPAPHLDGVHVVFGLVISGFEVIEQIENLKTDAASRPYADVRVIDCGVLA
XP_023988067	GSQFFITTKPAPHLDGVHVVFGLVISGFEVIEQIENLKTDAASRPYADVRVIDCGVLA
XP_019274384	GSQFFITTKPAPHLDGVHVVFGLVISGFEVIEQIENLKTDAASRPYADVRVIDCGVLA
XP_017396459	GSQFFITTKPAPHLDGVHVVFGLVISGFEVIEQIENLKTDAASRPYADVRVIDCGVLA
XP_008152845	GSQFFITTKPAPHLDGVHVVFGLVISGFEVIEQIENLKTDAASRPYADVRVIDCGVLA
NP_001336053	GSQFFITTKPAPHLDGVHVVFGLVISGFEVIEQIENLKTDAASRPYADVRVIDCGVLA
XP_007106821	GSQFFITTKPAPHLDGVHVVFGLVISGFEVIEQIENLKTDAASRPYADVRVIDCGVLA
XP_018879078	GSQFFITTKPAPHLDGVHVVFGLVISGFEVIEQIENLKTDAASRPYADVRVIDCGVLA
XP_017396458	GSQFFITTKPAPHLDGVHVVFGLVISGFEVIEQIENLKTDAASRPYADVRVIDCGVLA
XP_012315533	GSQFFITTKPAPHLDGVHVVFGLVISGFEVIEQIENLKTDAASRPYADVRVIDCGVLA
XP_007981991	GSQFFITTKPAPHLDGVHVVFGLVISGFEVIEQIENLKTDAASRPYADVRVIDCGVLA
XP_003256959	GSQFFITTKPAPHLDGVHVVFGLVISGFEVIEQIENLKTDAASRPYADVRVIDCGVLA
XP_003735136	GSQFFITTKPAPHLDGVHVVFGLVISGFEVIEQIENLKTDAASRPYADVRVIDCGVLA
XP_019802415	GSQFFITTKPAPHLDGVHVVFGLVISGFEVIEQIENLKTDAASRPYADVRVIDCGVLA
XP_018879076	GSQFFITTKPAPHLDGVHVVFGLVISGFEVIEQIENLKTDAASRPYADVRVIDCGVLA
XP_012315532	GSQFFITTKPAPHLDGVHVVFGLVISGFEVIEQIENLKTDAASRPYADVRVIDCGVLA
XP_011737181	GSQFFITTKPAPHLDGVHVVFGLVISGFEVIEQIENLKTDAASRPYADVRVIDCGVLA
XP_011889502	GSQFFITTKPAPHLDGVHVVFGLVISGFEVIEQIENLKTDAASRPYADVRVIDCGVLA
XP_010372619	GSQFFITTKPAPHLDGVHVVFGLVISGFEVIEQIENLKTDAASRPYADVRVIDCGVLA
XP_007981990	GSQFFITTKPAPHLDGVHVVFGLVISGFEVIEQIENLKTDAASRPYADVRVIDCGVLA
XP_004277919	GSQFFITTKPAPHLDGVHVVFGLVISGFEVIEQIENLKTDAASRPYADVRVIDCGVLA
XP_003894654	GSQFFITTKPAPHLDGVHVVFGLVISGFEVIEQIENLKTDAASRPYADVRVIDCGVLA
XP_019802412	GSQFFITTKPAPHLDGVHVVFGLVISGFEVIEQIENLKTDAASRPYADVRVIDCGVLA
XP_017811031	GSQFFITTKPAPHLDGVHVVFGLVISGFEVIEQIENLKTDAASRPYADVRVIDCGVLA
XP_008982345	GSQFFITTKPAPHLDGVHVVFGLVISGFEVIEQIENLKTDAASRPYADVRVIDCGVLA
XP_003477052	GSQFFITTKPAPHLDGVHVVFGLVISGFEVIEQIENLKTDAASRPYADVRVIDCGVLA
XP_013015643	GSQFFITTKPAPHLDGVHVVFGLVISGFEVIEQIENLKTDAASRPYADVRVIDCGVLA
XP_007499863	GSQFFITTKPAPHLDGVHVVFGLVISGFEVIEQIENLKTDAASRPYADVRVIDCGVLV
XP_023581750	GSQFFITTKPAPHLDGVHVVFGLVISGFEVIEQIENLKTDAASRPYADVRVIDCGVIA
XP_002813940	GSQFFITTKPAPHLDGVHVVFGLVISGFEVIEQIENLKTDAASRPYADVRVIDCGVLA
XP_014390380	GSQFFITTKPAPHLDGVHVVFGLVISGFEVIEQIENLKTDAASRPYADVRVIDCGVLA
XP_014323126	GSQFFITTKPAPHLDGVHVVFGLVISGFEVIEQIENLKTDAASRPYADVRVIDCGVLA
XP_024100262	GSQFFITTKPAPHLDGVHVVFGLVISGFEVIEQIENLKTDAASRPYADVRVIDCGVLA
XP_022409524	GSQFFITTKPAPHLDGVHVVFGLVISGFEVIEQIENLKTDAASRPYADVRVIDCGVLA
XP_022409523	GSQFFITTKPAPHLDGVHVVFGLVISGFEVIEQIENLKTDAASRPYADVRVIDCGVLA
XP_019802414	GSQFFITTKPAPHLDGVHVVFGLVISGFEVIEQIENLKTDAASRPYADVRVIDCGVLA
XP_021062318	GSQFFITTKPAPHLDGVHVVFGLVISGFEVIEQIENLKTDAASRPYADVRVIDCGVLA
XP_014390377	GSQFFITTKPAPHLDGVHVVFGLVISGFEVIEQIENLKTDAASRPYADVRVIDCGVLA
XP_014323123	GSQFFITTKPAPHLDGVHVVFGLVISGFEVIEQIENLKTDAASRPYADVRVIDCGVLA
XP_011889501	GSQFFITTKPAPHLDGVHVVFGLVISGFEVIEQIENLKTDAASRPYADVRVIDCGVLA
XP_015344917	GSQFFITTKPAPHLDGVHVVFGLVISGFEVIEQIENLKTDAASRPYADVRVIDCGVLA
XP_011889500	GSQFFITTKPAPHLDGVHVVFGLVISGFEVIEQIENLKTDAASRPYADVRVIDCGVLA
XP_016018800	GSQFFITTKPAPHLDGVHVVFGLVISGFEVIEQIENLKTDAASRPYADVRVIDCGVLA
XP_019802413	GSQFFITTKPAPHLDGVHVVFGLVISGFEVIEQIENLKTDAASRPYADVRVIDCGVLA
XP_023581752	GSQFFITTKPAPHLDGVHVVFGLVISGFEVIEQIENLKTDAASRPYADVRVIDCGVIA
XP_008847679	GSQFFITTKPAPHLDGVHVVFGLVISGFEVIEQIENLKTDAASRPYADVRVIDCGVLA
XP_016018799	GSQFFITTKPAPHLDGVHVVFGLVISGFEVIEQIENLKTDAASRPYADVRVIDCGVLA

XP_016018798	GSQFFITTKPAPHLDGVHVVFGLVISGFEVIEQIENLKTDAASRPYADVRVIDCGVLA
XP_017497112	GSQFFITTKPAPHLDGVHVVFGLVISGFEVIEQIENLKTDAASRPYADVRVIDCGVLA
XP_023371677	GSQFFITTKPAPHLDGVHVVFGLVISGFEVIEQIENLKTDAASRPYADVRVIDCGVLA
XP_007947878	GSQFFITTKPAPHLDGVHVVFGLVISGFEVIEQIENLKTDAASRPYADVRVIDCGVIA
XP_007445750	GSQFFITTKPAPHLDGVHVVFGLVISGFEVIEQIENLKTDAASRPYADVRVIDCGVLA
XP_011889497	GSQFFITTKPAPHLDGVHVVFGLVISGFEVIEQIENLKTDAASRPYADVRVIDCGVLA
XP_020854834	GSQFFITTKPAPHLDGVHVVFGLVISGFEVIEQIENLKTDAASRPYADVRVIDCGVLV
XP_012315531	GSQFFITTKPAPHLDGVHVVFGLVISGFEVIEQIENLKTDAASRPYADVRVIDCGVLA
XP_020854833	GSQFFITTKPAPHLDGVHVVFGLVISGFEVIEQIENLKTDAASRPYADVRVIDCGVLV
XP_012315530	GSQFFITTKPAPHLDGVHVVFGLVISGFEVIEQIENLKTDAASRPYADVRVIDCGVLA
XP_022409521	GSQFFITTKPAPHLDGVHVVFGLVISGFEVIEQIENLKTDAASRPYADVRVIDCGVLA
XP_023384830	GSQFFITTKPAPHLDGVHVVFGLVISGFEVIEQIENLKTDAASRPYADVRVIDCGVLA
XP_020041827	GSQFFITTKPAPHLDGVHVVFGLVISGFEVIEQIENLKTDAASRPYADVRVIDCGVLA
XP_015344916	GSQFFITTKPAPHLDGVHVVFGLVISGFEVIEQIENLKTDAASRPYADVRVIDCGVLA
XP_008847678	GSQFFITTKPAPHLDGVHVVFGLVISGFEVIEQIENLKTDAASRPYADVRVIDCGVLA
XP_015344914	GSQFFITTKPAPHLDGVHVVFGLVISGFEVIEQIENLKTDAASRPYADVRVIDCGVLA
XP_008847677	GSQFFITTKPAPHLDGVHVVFGLVISGFEVIEQIENLKTDAASRPYADVRVIDCGVLA
XP_005337989	GSQFFITTKPAPHLDGVHVVFGLVISGFEVIEQIENLKTDAASRPYADVRVIDCGVLA
XP_021590709	GSQFFITTKPAPHLDGVHVVFGLVISGFEVIEQIENLKTDAASRPYADVRVIDCGVLA
XP_022409522	GSQFFITTKPAPHLDGVHVVFGLVISGFEVIEQIENLKTDAASRPYADVRVIDCGVLA
XP_021590708	GSQFFITTKPAPHLDGVHVVFGLVISGFEVIEQIENLKTDAASRPYADVRVIDCGVLA
XP_021027182	GSQFFITTKPAPHLDGVHVVFGLVISGFEVIEQIENLKTDAASRPYADVRVIDCGVLA
XP_006914886	GSQFFITTKPAPHLDGVHVVFGLVISGFEVIEQIENLKTDAASRPYADVRVIDCGVLA
XP_011355685	GSQFFITTKPAPHLDGVHVVFGLVISGFEVIEQIENLKTDAASRPYADVRVIDCGVLA
XP_023384828	GSQFFITTKPAPHLDGVHVVFGLVISGFEVIEQIENLKTDAASRPYADVRVIDCGVLA
XP_006914885	GSQFFITTKPAPHLDGVHVVFGLVISGFEVIEQIENLKTDAASRPYADVRVIDCGVLA
XP_012663507	GSQFFITTKPAPHLDGVHVVFGLVISGFEVIEQIENLKTDAASRPYADVRVIDCGVLA
XP_012663506	GSQFFITTKPAPHLDGVHVVFGLVISGFEVIEQIENLKTDAASRPYADVRVIDCGVLA
XP_017497113	GSQFFITTKPAPHLDGVHVVFGLVISGFEVIEQIENLKTDAASRPYADVRVIDCGVLA
XP_016051563	GSQFFITTKPAPHLDGVHVVFGLVISGFEVIEQIENLKTDAASRPYADVRVIDCGVLA
XP_017497111	GSQFFITTKPAPHLDGVHVVFGLVISGFEVIEQIENLKTDAASRPYADVRVIDCGVLA
XP_017497110	GSQFFITTKPAPHLDGVHVVFGLVISGFEVIEQIENLKTDAASRPYADVRVIDCGVLA
XP_012315529	GSQFFITTKPAPHLDGVHVVFGLVISGFEVIEQIENLKTDAASRPYADVRVIDCGVLA
XP_020728074	GSQFFITTKPAPHLDGVHVVFGLVISGFEVIEQIENLKTDAASRPYADVRVIDCGVLA
XP_012315528	GSQFFITTKPAPHLDGVHVVFGLVISGFEVIEQIENLKTDAASRPYADVRVIDCGVLA
XP_020728073	GSQFFITTKPAPHLDGVHVVFGLVISGFEVIEQIENLKTDAASRPYADVRVIDCGVLA
XP_021062319	GSQFFITTKPAPHLDGVHVVFGLVISGFEVIEQIENLKTDAASRPYADVRVIDCGVLA
XP_021027181	GSQFFITTKPAPHLDGVHVVFGLVISGFEVIEQIENLKTDAASRPYADVRVIDCGVLA
XP_021027180	GSQFFITTKPAPHLDGVHVVFGLVISGFEVIEQIENLKTDAASRPYADVRVIDCGVLA
XP_004835151	GSQFFITTKPAPHLDGVHVVFGLVISGFEVIEQIENLKTDAASRPYADVRVIDCGVLA
XP_012928271	GSQFFITTKPAPHLDGVHVVFGLVISGFEVIEQIENLKTDAASRPYADVRVIDCGVLA
XP_021590705	GSQFFITTKPAPHLDGVHVVFGLVISGFEVIEQIENLKTDAASRPYADVRVIDCGVLA
XP_011241241	GSQFFITTKPAPHLDGVHVVFGLVISGFEVIEQIENLKTDAASRPYADVRVIDCGVLA
NP_035048	GSQFFITTKPAPHLDGVHVVFGLVISGFEVIEQIENLKTDAASRPYADVRVIDCGVLA
XP_017168694	GSQFFITTKPAPHLDGVHVVFGLVISGFEVIEQIENLKTDAASRPYADVRVIDCGVLA
XP_005348185	GSQFFITTKPAPHLDGVHVVFGLVISGFEVIEQIENLKTDAASRPYADVRVIDCGVLA
XP_005082370	GSQFFITTKPAPHLDGVHVVFGLVISGFEVIEQIENLKTDAASRPYADVRVIDCGVLA
XP_021503066	GSQFFITTKPAPHLDGVHVVFGLVISGFEVIEQIENLKTDAASRPYADVRVIDCGVIA
XP_021503065	GSQFFITTKPAPHLDGVHVVFGLVISGFEVIEQIENLKTDAASRPYADVRVIDCGVIA
XP_021503062	GSQFFITTKPAPHLDGVHVVFGLVISGFEVIEQIENLKTDAASRPYADVRVIDCGVIA
XP_021503063	GSQFFITTKPAPHLDGVHVVFGLVISGFEVIEQIENLKTDAASRPYADVRVIDCGVIA
XP_008568057	GSQFFITTKPAPHLDGVHVVFGLVISGFEIIKQIENLKTDAASRPYADVRVIDCGVLA
XP_008568056	GSQFFITTKPAPHLDGVHVVFGLVISGFEIIKQIENLKTDAASRPYADVRVIDCGVLA
XP_006120664	GSQFFITTKPAPHLDGVHVVFGLVISGFEVIEQIENLKTDTASRPYADVRVIDCGVLV
XP_007055629	GSQFFITTKPAPHLDGVHVVFGLVISGFEVIEQIENLKTDTASRPYADVRVIDCGVLV
XP_006120661	GSQFFITTKPAPHLDGVHVVFGLVISGFEVIEQIENLKTDTASRPYADVRVIDCGVLV
XP_006120659	GSQFFITTKPAPHLDGVHVVFGLVISGFEVIEQIENLKTDTASRPYADVRVIDCGVLV
XP_007106819	GSQFFITTKPAPHLDGVHVVFGLVISGFEVIEQIENLKTDAASRPYADVRVIDCGVLA

XP_015708506	GSQFFITTKPAPHLDGVHVVFGLVISGFEVIEQIENLKTDTASRPYADVRVIDCGVLV
XP_009682494	GSQFFITTKPAPHLDGVHVVFGLVISGFEVIEQIENLKTDTASRPYADVRVIDCGVLV
XP_009682493	GSQFFITTKPAPHLDGVHVVFGLVISGFEVIEQIENLKTDTASRPYADVRVIDCGVLV
XP_009682492	GSQFFITTKPAPHLDGVHVVFGLVISGFEVIEQIENLKTDTASRPYADVRVIDCGVLV
XP_009682491	GSQFFITTKPAPHLDGVHVVFGLVISGFEVIEQIENLKTDTASRPYADVRVIDCGVLV
XP_019471734	GSQFFITTKPAPHLDGVHVVFGLVISGFEVIEQIENLKTDAASRPYADVRVIDCGVLV
XP_010710549	GSQFFITTKPAPHLDGVHVVFGLVISGFEVIEQIENLKTDAASRPYADVRVIDCGVLV
XP_010710548	GSQFFITTKPAPHLDGVHVVFGLVISGFEVIEQIENLKTDAASRPYADVRVIDCGVLV
XP_015136632	GSQFFITTKPAPHLDGVHVVFGLVISGFEVIEQIENLKTDTASRPYADVRVIDCGVLV
XP_004939132	GSQFFITTKPAPHLDGVHVVFGLVISGFEVIEQIENLKTDTASRPYADVRVIDCGVLV
XP_004939131	GSQFFITTKPAPHLDGVHVVFGLVISGFEVIEQIENLKTDTASRPYADVRVIDCGVLV
XP_013795593	GSQFFITTKPAPHLDGVHVVFGLVISGFEVIEQIENLKTDTASRPYADVRVIDCGVLV
XP_019363610	GSQFFITTKPAPHLDGVHVVFGLVISGFEVIEQIENLKTDTASRPYADVRVIDCGVVI
XP_014376468	GSQFFITTKPAPHLDGVHVVFGLVISGFEVIEQIENLKTDTASRPYADVRVIDCGVVI
XP_007659513	GSQFFITTKPAPHLDGVHVVFGLVISGFEVIEQIENLKTDSASRPYADVRVIDCGVLI
XP_021241246	GSQFFITTKPAPHLDGVHVVFGLVISGFEVIEQIENLKTDTASRPYADVRVIDCGVLV
XP_021241236	GSQFFITTKPAPHLDGVHVVFGLVISGFEVIEQIENLKTDTASRPYADVRVIDCGVLV
XP_021241235	GSQFFITTKPAPHLDGVHVVFGLVISGFEVIEQIENLKTDTASRPYADVRVIDCGVLV
XP_017731869	GSQFFITTKPAPHLDGVHVVFGLVISGFEVIEQIENLKTDAASRPYADVRVIDCGVLA
XP_012426793	GSQFFITTKPAPHLDGVHVVFGLVISGFEVIEQIENLKTDTASRPYADVRVIDCGVLV
XP_017944741	GSQFFITTKPAPHLDGVHVVFGLVISGFEVIEQIENLKTDTASRPYADVRVIDCGVLV
XP_017944740	GSQFFITTKPAPHLDGVHVVFGLVISGFEVIEQIENLKTDTASRPYADVRVIDCGVLV
XP_010393085	GSQFFITTKPAPHLDGVHVVFGLVISGFEVIEQIENLKTDTASRPYADVRVIDCGVLV
XP_017685518	GSQFFITTKPAPHLDGVHVVFGLVISGFEVIEQIENLKTDTASRPYADVRVIDCGVLV
XP_017602246	GSQFFITTKPAPHLDGVHVVFGLVISGFEVIEQIENLKTDTASRPYADVRVIDCGVLV
XP_016151723	GSQFFITTKPAPHLDGVHVVFGLVISGFEVIEQIENLKTDTASRPYADVRVIDCGVLV
XP_019138709	GSQFFITTKPAPHLDGVHVVFGLVISGFEVIEQIENLKTDTASRPYADVRVIDCGVLV
XP_017685517	GSQFFITTKPAPHLDGVHVVFGLVISGFEVIEQIENLKTDTASRPYADVRVIDCGVLV
XP_017602245	GSQFFITTKPAPHLDGVHVVFGLVISGFEVIEQIENLKTDTASRPYADVRVIDCGVLV
XP_005040539	GSQFFITTKPAPHLDGVHVVFGLVISGFEVIEQIENLKTDTASRPYADVRVIDCGVLV
XP_005040538	GSQFFITTKPAPHLDGVHVVFGLVISGFEVIEQIENLKTDTASRPYADVRVIDCGVLV
XP_005040537	GSQFFITTKPAPHLDGVHVVFGLVISGFEVIEQIENLKTDTASRPYADVRVIDCGVLV
XP_014800495	GSQFFITTKPAPHLDGVHVVFGLVISGFEVIEQIENLKTDTASRPYADVRVIDCGVLV
XP_008168983	GSQFFITTKPAPHLDGVHVVFGLVISGFEVIEQIENLKTDTASRPYADVRVIDCGVLV
XP_017944739	GSQFFITTKPAPHLDGVHVVFGLVISGFEVIEQIENLKTDTASRPYADVRVIDCGVLV
XP_014729203	GSQFFITTKPAPHLDGVHVVFGLVISGFEVIEQIENLKTDTASRPYADVRVIDCGVLV
XP_017944736	GSQFFITTKPAPHLDGVHVVFGLVISGFEVIEQIENLKTDTASRPYADVRVIDCGVLV
XP_014729201	GSQFFITTKPAPHLDGVHVVFGLVISGFEVIEQIENLKTDTASRPYADVRVIDCGVLV
XP_010393084	GSQFFITTKPAPHLDGVHVVFGLVISGFEVIEQIENLKTDTASRPYADVRVIDCGVLV
XP_017944735	GSQFFITTKPAPHLDGVHVVFGLVISGFEVIEQIENLKTDTASRPYADVRVIDCGVLV
XP_017685520	GSQFFITTKPAPHLDGVHVVFGLVISGFEVIEQIENLKTDTASRPYADVRVIDCGVLV
XP_017602244	GSQFFITTKPAPHLDGVHVVFGLVISGFEVIEQIENLKTDTASRPYADVRVIDCGVLV
XP_014729200	GSQFFITTKPAPHLDGVHVVFGLVISGFEVIEQIENLKTDTASRPYADVRVIDCGVLV
XP_010393082	GSQFFITTKPAPHLDGVHVVFGLVISGFEVIEQIENLKTDTASRPYADVRVIDCGVLV
XP_017685515	GSQFFITTKPAPHLDGVHVVFGLVISGFEVIEQIENLKTDTASRPYADVRVIDCGVLV
XP_017602242	GSQFFITTKPAPHLDGVHVVFGLVISGFEVIEQIENLKTDTASRPYADVRVIDCGVLV
XP_017602241	GSQFFITTKPAPHLDGVHVVFGLVISGFEVIEQIENLKTDTASRPYADVRVIDCGVLV
KFP58102	GSQFFITTKPAPHLDGVHVVFGLVISGFEVIEQIENLKTDTASRPYADVRVIDCGVLV
XP_010393081	GSQFFITTKPAPHLDGVHVVFGLVISGFEVIEQIENLKTDTASRPYADVRVIDCGVLV
XP_017685514	GSQFFITTKPAPHLDGVHVVFGLVISGFEVIEQIENLKTDTASRPYADVRVIDCGVLV
XP_017602240	GSQFFITTKPAPHLDGVHVVFGLVISGFEVIEQIENLKTDTASRPYADVRVIDCGVLV
XP_010559721	GSQFFITTKPAPHLDGVHVVFGLVISGFEVIEQIENLKTDTASRPYADVRVIDCGVLV
XP_014800494	GSQFFITTKPAPHLDGVHVVFGLVISGFEVIEQIENLKTDTASRPYADVRVIDCGVLV
XP_011571332	GSQFFITTKPAPHLDGVHVVFGLVISGFEVIEQIENLKTDTASRPYADVRVIDCGVLV
XP_010559712	GSQFFITTKPAPHLDGVHVVFGLVISGFEVIEQIENLKTDTASRPYADVRVIDCGVLV
XP_009331567	GSQFFITTKPAPHLDGVHVVFGLVISGFEVIEQIENLKTDTASRPYADVRVIDCGVLV
XP_008492672	GSQFFITTKPAPHLDGVHVVFGLVISGFEVIEQIENLKTDTASRPYADVRVIDCGVLV
XP_014800492	GSQFFITTKPAPHLDGVHVVFGLVISGFEVIEQIENLKTDTASRPYADVRVIDCGVLV

XP_011571324	GSQFFITTKPAPHLDGVHVVFGLVISGFEVIEQIENLKTDTASRPYADVRVIDCGVLV
XP_009697766	GSQFFITTKPAPHLDGVHVVFGLVISGFEVIEQIENLKTDTASRPYADVRVIDCGVLV
XP_009331566	GSQFFITTKPAPHLDGVHVVFGLVISGFEVIEQIENLKTDTASRPYADVRVIDCGVLV
XP_009274213	GSQFFITTKPAPHLDGVHVVFGLVISGFEVIEQIENLKTDTASRPYADVRVIDCGVLV
XP_014800491	GSQFFITTKPAPHLDGVHVVFGLVISGFEVIEQIENLKTDTASRPYADVRVIDCGVLV
XP_010301634	GSQFFITTKPAPHLDGVHVVFGLVISGFEVIEQIENLKTDTASRPYADVRVIDCGVLV
XP_008168980	GSQFFITTKPAPHLDGVHVVFGLVISGFEVIEQIENLKTDTASRPYADVRVIDCGVLV
XP_023963187	GSQFFITTKPAPHLDGVHVVFGLVISGFEVIEQIENLKTDTASRPYADVRVIDCGVLV
XP_005150066	GSQFFITTKPAPHLDGVHVVFGLVISGFEVIEQIENLKTDTASRPYADVRVIDCGVLV
XP_009473612	GSQFFITTKPAPHLDGVHVVFGLVISGFEVIEQIENLKTDTASRPYADVRVIDCGVLV
XP_021393646	GSQFFITTKPAPHLDGVHVVFGLVISGFEVIEQIENLKTDTASRPYADVRVIDCGVLV
XP_021393642	GSQFFITTKPAPHLDGVHVVFGLVISGFEVIEQIENLKTDTASRPYADVRVIDCGVLV
XP_021393643	GSQFFITTKPAPHLDGVHVVFGLVISGFEVIEQIENLKTDTASRPYADVRVIDCGVLV
XP_009878793	GSQFFITTKPAPHLDGVHVVFGLVISGFEVIEQIENLKTDTASRPYADVRVIDCGVLV
XP_021393641	GSQFFITTKPAPHLDGVHVVFGLVISGFEVIEQIENLKTDTASRPYADVRVIDCGVLV
XP_018764676	GSQFFITTKPAPHLDGVHVVFGLVISGFEVIEQIENLKTDTASRPYADVRVIDCGVLV
XP_018764674	GSQFFITTKPAPHLDGVHVVFGLVISGFEVIEQIENLKTDTASRPYADVRVIDCGVLV
XP_018764673	GSQFFITTKPAPHLDGVHVVFGLVISGFEVIEQIENLKTDTASRPYADVRVIDCGVLV
KFV02250	GSQFFITTKPAPHLDGVHVVFGLVISGFEVIEQIENLKTDTASRPYADVRVIDCGVLV
XP_014125627	GSQFFITTKPAPHLDGVHVVFGLVISGFEVIEQIENLKTDTASRPYADVRVIDCGVLL
XP_010083448	GSQFFITTKPAPHLDGVHVVFGLVISGFEVIEQIENLKTDTASRPYADVRVIDCGVLV
XP_014125626	GSQFFITTKPAPHLDGVHVVFGLVISGFEVIEQIENLKTDTASRPYADVRVIDCGVLL
XP_014112250	GSQFFITTKPAPHLDGVHVVFGLVISGFEVIEQIENLKTDTASRPYADVRVIDCGVLV
XP_014112249	GSQFFITTKPAPHLDGVHVVFGLVISGFEVIEQIENLKTDTASRPYADVRVIDCGVLV
XP_014125625	GSQFFITTKPAPHLDGVHVVFGLVISGFEVIEQIENLKTDTASRPYADVRVIDCGVLL
XP_014112248	GSQFFITTKPAPHLDGVHVVFGLVISGFEVIEQIENLKTDTASRPYADVRVIDCGVLV
XP_005428264	GSQFFITTKPAPHLDGVHVVFGLVISGFEVIEQIENLKTDTASRPYADVRVIDCGVLV
XP_015474510	GSQFFITTKPAPHLDGVHVVFGLVISGFEVIEQIENLKTDTASRPYADVRVIDCGVLV
XP_023777798	GSQFFITTKPAPHLDGVHVVFGLVISGFEVIEQIENLKTDTASRPYADVRVIDCGVLV
XP_015474508	GSQFFITTKPAPHLDGVHVVFGLVISGFEVIEQIENLKTDTASRPYADVRVIDCGVLV
XP_023777797	GSQFFITTKPAPHLDGVHVVFGLVISGFEVIEQIENLKTDTASRPYADVRVIDCGVLV
XP_023777796	GSQFFITTKPAPHLDGVHVVFGLVISGFEVIEQIENLKTDTASRPYADVRVIDCGVLV
XP_015474507	GSQFFITTKPAPHLDGVHVVFGLVISGFEVIEQIENLKTDTASRPYADVRVIDCGVLV
XP_023777795	GSQFFITTKPAPHLDGVHVVFGLVISGFEVIEQIENLKTDTASRPYADVRVIDCGVLV
KFQ42818	GSQFFITTKPAPHLDGVHVVFGLVISGFEVIEQIENLKTDTASRPYADVRVIDCGVLV
XP_019355067	GSQFFITTKPAPHLDSVHVVFGLVISGFEVIEQIENLKTDTASRPYADVRVIDCGVVI
XP_010014722	GSQFFITTKPAPHLDGVHVVFGLVISGFEVIEQIENLKTDTASRPYADVRVIDCGVLV
XP_009556564	GSQFFITTKPAPHLDGVHVVFGLVISGFEVIEQIENLKTDTASRPYADVRVIDCGVLV
XP_009993002	GSQFFITTKPAPHLDGVHVVFGLVISGFEVIEQIENLKTDTASRPYADVRVIDCGVLV
XP_009896433	GSQFFITTKPAPHLDGVHVVFGLVISGFEVIEQIENLKTDTASRPYADVRVIDCGVLV
XP_010213065	GSQFFITTKPAPHLDGVHVVFGLVISGFEVIEQIENLKTDTASRPYADVRVIDCGVLV
XP_019384731	GSQFFITTKPAPHLDGVHVVFGLVISGFEVIEQIENLKTDTASRPYADVRVIDCGVVI
XP_008110438	GSQFFITTKPAPHLDGVHVVFGLVISGFEAIEQIENLKTDTASRPYADVRVIDCGVLV
XP_008110436	GSQFFITTKPAPHLDGVHVVFGLVISGFEAIEQIENLKTDTASRPYADVRVIDCGVLV
XP_015264278	GSQFFITTKPAPHLDNVHVVFGLVISGFEVIEQIENLKTDAASRPYADVRVIKCGVLV
XP_013917336	GSQFFITTKPAPHLDSVHVVFGLVISGFEVIEQIENLKTDSASRPYADVRVIDCGVLV
XP_020670421	GSQFFITTKPAPHLDGVHVVFGLVISGFEVIEQIENLKTDTASRPYADVRVIDCGVLV
XP_020670420	GSQFFITTKPAPHLDGVHVVFGLVISGFEVIEQIENLKTDTASRPYADVRVIDCGVLV
XP_015667896	GSQFFITTKPAPHLDSVHVVFGLVISGFEVIEQIENLKTDSASRPYADVRVIDCGVLV
XP_007443413	GSQFFITTKPAPHLDGVHVVFGLVISGFEVIEQIENLKTDPASRPYADVRVIDCGVLV
XP_007443411	GSQFFITTKPAPHLDGVHVVFGLVISGFEVIEQIENLKTDPASRPYADVRVIDCGVLV
XP_017451684	GSQFFITTKPAPHLDGVHVVFGLVISGFEVIEQIENLKTDAASRPYADVRVIDCGVLA
XP_014918330	GSQFFITTKPAPHLDGVHVVFGLVISGFEVIEQIENLKTDAASRPYADVRVIDCGVLA
XP_002939352	GSQFFITTKAAPHLDGVHVVFGLVISGFEVIEQIESLKTDAASRPYADVRVIDCGLLV
XP_018124467	GSQFFITTKAAPHLDGVHVVFGLVISGFEVIEQIESLKTDAASRPYADVRVIDCGLLL
XP_017572959	GSQFFITTKPAPHLDGVHVVFGLVISGFEVIKRIEGLKTDSASRPYADVRVIDCGQLI
XP_018607018	GSQFFITTKTAPHLDGVHVVFGLVISGFEVIKQIECLKTDSASRPYADVRVIDCGQLI
XP_018607016	GSQFFITTKTAPHLDGVHVVFGLVISGFEVIKQIECLKTDSASRPYADVRVIDCGQLI

XP_007544596	GSQFFITTKMAPHLDGVHVVFGLVISGFEVIKKIEGLKTDSASRPYADVRVVDCGQLI
XP_018607022	GSQFFITTKTAPHLDGVHVVFGLVISGFEVIKQIECLKTDSASRPYADVRVIDCGQLI
XP_009443552	GSQFFM----------VHVVFGLVISGFEVIEQIENLKTDAASRPYADVRVIDCGVLA
PNJ74568	GSQFFM----------VHVVFGLVISGFEVIEQIENLKTDAASRPYADVRVIDCGVLA
PNJ74566	GSQFFM----------VHVVFGLVISGFEVIEQIENLKTDAASRPYADVRVIDCGVLA
XP_018122561	GSQFFITTKAAPHLDGVHVVFGLVISGFEVIERIESLKSDAASRPYADVRVIDCGLLV
XP_012735608	GSQFFITTKMAPHLDGVHVVFGLVISGFEVIKKIEKLKTDSASRPYADVRVVDCGQLI
XP_022065410	GSQFFITTKMAPHLDGVHVVFGLVISGFEVIKKIEGLKTDSASRPYADVRVMDCGQLI
XP_023122049	GSQFFITTKMAPHLDGVHVVFGLVISGFEVIKKIEGLKTDSASRPYADVRVMDCGQLI
XP_022065408	GSQFFITTKMAPHLDGVHVVFGLVISGFEVIKKIEGLKTDSASRPYADVRVMDCGQLI
XP_023122045	GSQFFITTKMAPHLDGVHVVFGLVISGFEVIKKIEGLKTDSASRPYADVRVMDCGQLI
XP_022065407	GSQFFITTKMAPHLDGVHVVFGLVISGFEVIKKIEGLKTDSASRPYADVRVMDCGQLI
XP_022065406	GSQFFITTKMAPHLDGVHVVFGLVISGFEVIKKIEGLKTDSASRPYADVRVMDCGQLI
XP_023122044	GSQFFITTKMAPHLDGVHVVFGLVISGFEVIKKIEGLKTDSASRPYADVRVMDCGQLI
XP_022065405	GSQFFITTKMAPHLDGVHVVFGLVISGFEVIKKIEGLKTDSASRPYADVRVMDCGQLI
XP_023122043	GSQFFITTKMAPHLDGVHVVFGLVISGFEVIKKIEGLKTDSASRPYADVRVMDCGQLI
XP_018414151	GSQFFITTKPAAHLDGVHVVFGLVISGFEVIEQIENQKSDAASRPYADVRVVDCGLVG
XP_023490562	GSQFFITTKPAPHLDGVHVVFGLVISGFEVIEQIENLKTDAASRPYADVRVIDCGVLA
XP_008304546	GSQFFITTKMAPHLDGVHVVFGLVISGFEVIKKIEGLKTDSASRPYADVRVMDCGQLI
KKF16876	GSQFFITTKMAPHLDGVHVVFGVVISGFEVIKKIEGLKTDSASRPYADVRVMDCGQLI
XP_008304545	GSQFFITTKMAPHLDGVHVVFGLVISGFEVIKKIEGLKTDSASRPYADVRVMDCGQLI
XP_015249666	GSQFFITTRMAPHLDGVHVVFGLVISGFEVIKKIEKLKTDSASRPYADVRVVDCGQLI
XP_018529332	GSQFFITTKMAPHLDGVHVVFGLVISGFEVIKKIEGLKTDSASRPYADVRVMDCGQLI
XP_018529331	GSQFFITTKMAPHLDGVHVVFGLVISGFEVIKKIEGLKTDSASRPYADVRVMDCGQLI
XP_010751380	GSQFFITTKMAPHLDGVHVVFGVVISGFEVIKKIEGLKTDSASRPYADVRVMDCGQLI
XP_010751371	GSQFFITTKMAPHLDGVHVVFGVVISGFEVIKKIEGLKTDSASRPYADVRVMDCGQLI
XP_019745550	GSQFFITTKMAPHLDGVHVVFGLVISGFEVIKKIEGLKTDSASRPYADVRVVDCGQLI
XP_010751365	GSQFFITTKMAPHLDGVHVVFGVVISGFEVIKKIEGLKTDSASRPYADVRVMDCGQLI
XP_019745545	GSQFFITTKMAPHLDGVHVVFGLVISGFEVIKKIEGLKTDSASRPYADVRVVDCGQLI
XP_023263705	GSQFFITTKMAPHLDGVHVVFGLVISGFEVIKKIEGLKTDSASRPYADVRVVDCGQLI
XP_023263706	GSQFFITTKMAPHLDGVHVVFGLVISGFEVIKKIEGLKTDSASRPYADVRVVDCGQLI
XP_001338072	GSQFFITTKTAPHLDGVHVVFGLVISGFEVIKNIEGLKTDSASRPYADVRVIDCGQLI
XP_022599954	GSQFFITTKMAPHLDGVHVVFGLVISGFEVIKKIEGLKTDSASRPYADVRVVDCGQLI
XP_019897364	GSQFFITTKTAPHLDGVHVVFGLVISGFEVVKRIEGLKTDGASRPYADVRVMDCGQLI
XP_010900666	GSQFFITTKTAPHLDGVHVVFGLVISGFEVVKRIEGLKTDGASRPYADVRVMDCGQLI
XP_010900665	GSQFFITTKTAPHLDGVHVVFGLVISGFEVVKRIEGLKTDGASRPYADVRVMDCGQLI
XP_010900664	GSQFFITTKTAPHLDGVHVVFGLVISGFEVVKRIEGLKTDGASRPYADVRVMDCGQLI
XP_019897363	GSQFFITTKTAPHLDGVHVVFGLVISGFEVVKRIEGLKTDGASRPYADVRVMDCGQLI
XP_010167755	GSQFFITTKPAPHLDGVHVVFGLVISGFEVIEQIENLKTDTASRPYADVRVIDCGVLV
XP_010900668	GSQFFITTKTAPHLDGVHVVFGLVISGFEVVKRIEGLKTDGASRPYADVRVMDCGQLI
XP_016427080	GSQFFITTKTAPHLDGVHVVFGLVISGFEVIKKIEGLKTDSASRPYADVRVIDCGQLI
XP_016318510	GSQFFITTKTAPHLDGVHVVFGLVISGFEVIKKIEGLKTDSASRPYADVRVIDCGQLI
XP_015209664	GSQFFITTKMAPHLDGVHVVFGLVISGFEVIRQIENLKTDAASRPYADVRVIDCGELI
XP_015209662	GSQFFITTKMAPHLDGVHVVFGLVISGFEVIRQIENLKTDAASRPYADVRVIDCGELI
XP_015209665	GSQFFITTKMAPHLDGVHVVFGLVISGFEVIRQIENLKTDAASRPYADVRVIDCGELI
XP_005912346	GSQFFITTKMAPHLDGVHVVFGLVISGFEVIKKIEGLKTDSASRPYADVRVIDCGQLI
XP_005722738	GSQFFITTKMAPHLDGVHVVFGLVISGFEVIKKIEGLKTDSASRPYADVRVIDCGQLI
XP_020457914	GSQFFITTKMAPHLDGVHVVFGLVISGFEVIKKIEGLKTDSASRPYADVRVVDCGQLI
XP_013132609	GSQFFITTKMAPHLDGVHVVFGLVISGFEVIKKIEGLKTDSASRPYADVRVVDCGQLI
XP_004546791	GSQFFITTKMAPHLDGVHVVFGLVISGFEVIKKIEGLKTDSASRPYADVRVIDCGQLI
XP_006795195	GSQFFITTKMAPHLDGVHVVFGLVISGFEVIKKIEGLKTDSASRPYADVRVIDCGQLI
XP_005912345	GSQFFITTKMAPHLDGVHVVFGLVISGFEVIKKIEGLKTDSASRPYADVRVIDCGQLI
XP_005722737	GSQFFITTKMAPHLDGVHVVFGLVISGFEVIKKIEGLKTDSASRPYADVRVIDCGQLI
XP_021475699	GSQFFITTKTAPHLDGVHVVFGLVISGFEVIKRIEGLKTDGASRPYADVRVMDCGQLI
XP_004546790	GSQFFITTKMAPHLDGVHVVFGLVISGFEVIKKIEGLKTDSASRPYADVRVIDCGQLI
XP_003439241	GSQFFITTKMAPHLDGVHVVFGLVISGFEVIKKIEGLKTDSASRPYADVRVVDCGQLI
XP_006795194	GSQFFITTKMAPHLDGVHVVFGLVISGFEVIKKIEGLKTDSASRPYADVRVIDCGQLI
XP_023490560	GSQFFITTKPAPHLDGVHVVFGLVISGFEVIEQIENLKTDAASRPYADVRVIDCGVLA

```
XP_015209666      GSQFFITTKMAPHLDGVHVVFGLVISGFEVIRQIENLKTDAASRPYADVRVIDCGELI
XP_005797888      GSQFFITTKMAPHLDGVHVVFGLVISGFEVIKKIEGLKTDSASRPYADVRVVDCGQLI
CBN81984          GSQFFITTKMAPHLDGVHVVFGLVISGFEVIKKIEGLKTDSASRPYADVRVMDCGQLI
XP_010559731      GSQFFI----------VHVVFGLVISGFEVIEQIENLKTDTASRPYADVRVIDCGVLV
XP_007544415      GSQFFITTKMAPHLDGVHVVFGLVISGFEVIKKIEGLKTDSASRPYADVRVVDCGQLI
XP_014870682      GSQFFITTKMAPHLDGVHVVFGLVISGFEVIKKIEGLKTDSASRPYADVRVVDCGQLI
XP_011606242      GSQFFITTKMAPHLDGVHVVFGLVISGFEVVKKIEGLKTDSASRPYADVRVVDCGQLI
CAG04245          GSQFFITTKMAPHLDGVHVVFGLVISGFEVVKKIEGLKTDAASRPYADVRVVDCGQLI
XP_016887118      GSQFFITTKMAPHLDGVHVVFGLVISGFEVIKKIEGLKTDSASRPYADVRVMDCGQLI
XP_008306161      GSQFFITTKMAPHLDGVHVVFGLVISGFEVIKKIEGLKTDSASRPYADVRVMDCGQLI
XP_008394441      GSQFFITTKMAPHLDGVHVVFGLVISGFEVIKKIEGLKTDSASRPYADVRVVDCGQLI
XP_014825423      GSQFFITTKMAPHLDGVHVVFGLVISGFEVIKKIEGLKTDSASRPYADVRVVDCGQLI
XP_012398954      GSQFFITTKPAPHLDGVHVVFGLVISGFEVIEQIENLKTDAASRPYADVRVIDCGVLV
XP_019063096      GSQFFITTKPAPHLDGVHVVFGLVISGFEVIEQIENLKTDAASRPYADVRVIDCGVLA
XP_009073803      GSQFFITTKPAPHLDGVHVVFGLVISGFEVIEQIENLKTDTASRPYADVRVIDCGVLV
XP_016400109      GSQFFITTKTAPHLDGVHVVFGLVISGFEVIKTIEGLKTDSASRPYADVRVIDCGQLI
XP_016359192      GSQFFITTKTAPHLDGVHVVFGLVISGFEVIKKIEGLKTDSASRPYADVRVIDCGQLI
XP_004081327      GSQFFITTKTAPHLDGVHVVFGLVISGFEIIKKIEGLKTDSASRPYADVRVVDCGQLI
XP_020568300      GSQFFITTKTAPHLDGVHVVFGLVISGFEIIKKIEGLKTDSASRPYADVRVVDCGQLI
XP_016041020      GSQFFITTKPAPHLDGVHVVFGLVISGFEVIEQIENLKTDAASRPYADVRVIDCGVLA
XP_017942181      GSQFFITTKPAPHLDGVHVVFGLVISGFEVIEQIENLKTDTASRPYADVRVIDCGVLV
XP_017942183      GSQFFITTKPAPHLDGVHVVFGLVISGFEVIEQIENLKTDTASRPYADVRVIDCGVLV
XP_017942182      GSQFFITTKPAPHLDGVHVVFGLVISGFEVIEQIENLKTDTASRPYADVRVIDCGVLV
XP_020501265      GSQFFITTKMAPHLDGVHVVFGVVISGFEVIKKIEGLKADSASRPYADVRVMDCGQLF
KFP01329          GSQFFITTKPAPHLDGVHVVFGLVISGFEVIEQIENLKTDTASRPYADVRVIDCGVLV
XP_015856948      GSQFFITTKPAPHLDGVHVVFGLVISGFEVIEQIENLKTDAASRPYADVRVIDCGVLA
XP_020139631      GSQFFITTKPAPHLDGVHVVFGLVISGFEVIEQIENLKTDAASRPYADVRVIDCGVLA
XP_020139628      GSQFFITTKPAPHLDGVHVVFGLVISGFEVIEQIENLKTDAASRPYADVRVIDCGVLA
XP_006982975      GSQFFITTKPAPHLDGVHVVFGLVISGFEVIEQIENLKTDAASRPYADVRVIDCGVLA
XP_015856947      GSQFFITTKPAPHLDGVHVVFGLVISGFEVIEQIENLKTDAASRPYADVRVIDCGVLA
XP_014427753      GSQFFITTKPAPHLDGVHVVFGLVISGFEVIEQIENLKTDTASRPYADVRVIDCGVLV
XP_013857648      GSQFFITTKMAPHLDGVDVVFGHVISGFEVIKKIEGLKTDSASRPYADVRVVDCGQLI
XP_023656013      GSQFFITTKMAPHLDGVHVVFGLVISGFEVVKKIESLKTDSASRPYADVRVIDCGQLI
XP_023656011      GSQFFITTKMAPHLDGVHVVFGLVISGFEVVKKIESLKTDSASRPYADVRVIDCGQLI
XP_023656014      GSQFFITTKMAPHLDGVHVVFGLVISGFEVVKKIESLKTDSASRPYADVRVIDCGQLI
XP_004448689      GSQFFITTKPAPHLDGVHVVFGLVISGFEVIEQIENLKTDAASRPYADVRVIDCGVLA
XP_004448688      GSQFFITTKPAPHLDGVHVVFGLVISGFEVIEQIENLKTDAASRPYADVRVIDCGVLA
XP_023476440      GSQFFITTKPAPHLDGVHVVFGLVISGFEVIEQIENLKTDAASRPYADVRVIDCGVLA
                  *****:          *.**** ****** :. **  *:* **********:.** :
```
