# Supplementary material for: Compression of Large Sets of Sequence Data Reveals Fine Diversification of Functional Profiles in Multigene Families of Proteins: A Study for Peptidyl-Prolyl cis/trans Isomerases (PPIase)
Source: Biomolecules. 2019 Feb 11;9(2):59. doi: 10.3390/biom9020059 (PMC6406343; doi:10.3390/biom9020059)
Supplement: Supplementary file 1 [file biomolecules-09-00059-s001.zip › Figure S8B-Names of CyP157s.nam.pdf]

CyP-157 (nuclear membrane)

(1)	NP_005376	NK-tumor recognition protein isoform a [Homo sapiens].
(2)	XP_014646452	PREDICTED: NK-tumor recognition protein isoform X5 [Ceratotherium simum simum
(3)	XP_017657797	PREDICTED: NK-tumor recognition protein isoform X5 [Nannospalax galili].
(4)	XP_012885690	PREDICTED: NK-tumor recognition protein [Dipodomys ordii].
(5)	XP_008530969	PREDICTED: NK-tumor recognition protein isoform X8 [Equus przewalskii].
(6)	XP_015423871	PREDICTED: NK-tumor recognition protein isoform X7 [Myotis davidii].
(7)	XP_015091833	PREDICTED: NK-tumor recognition protein isoform X5 [Vicugna pacos].
(8)	XP_010973060	PREDICTED: NK-tumor recognition protein isoform X1 [Camelus dromedarius].
(9)	XP_019583096	PREDICTED: NK-tumor recognition protein isoform X5 [Rhinolophus sinicus].
(10)	XP_011231111	PREDICTED: NK-tumor recognition protein isoform X3 [Ailuropoda melanoleuca].
(11)	XP_014646457	PREDICTED: NK-tumor recognition protein isoform X6 [Ceratotherium simum simum
(12)	XP_023988069	NK-tumor recognition protein isoform X8 [Physeter catodon].
(13)	XP_008530970	PREDICTED: NK-tumor recognition protein isoform X9 [Equus przewalskii].
(14)	XP_017497119	PREDICTED: NK-tumor recognition protein isoform X7 [Manis javanica].
(15)	XP_021503072	NK-tumor recognition protein isoform X11 [Meriones unguiculatus].
(16)	XP_021503071	NK-tumor recognition protein isoform X10 [Meriones unguiculatus].
(17)	XP_020041828	NK-tumor recognition protein isoform X2 [Castor canadensis].
(18)	XP_021503070	NK-tumor recognition protein isoform X9 [Meriones unguiculatus].
(19)	XP_012590780	PREDICTED: NK-tumor recognition protein isoform X2 [Condylura cristata].
(20)	XP_019274390	PREDICTED: NK-tumor recognition protein isoform X5 [Panthera pardus].
(21)	XP_019489071	PREDICTED: NK-tumor recognition protein isoform X2 [Hipposideros armiger].
(22)	XP_019489063	PREDICTED: NK-tumor recognition protein isoform X1 [Hipposideros armiger].
(23)	XP_010588206	NK-tumor recognition protein isoform X5 [Loxodonta africana].
(24)	XP_023403600	NK-tumor recognition protein isoform X4 [Loxodonta africana].
(25)	XP_009982730	PREDICTED: NK-tumor recognition protein-like, partial [Tauraco erythrolophus]
(26)	XP_019839655	PREDICTED: NK-tumor recognition protein, partial [Bos indicus].
(27)	XP_006512034	PREDICTED: NK-tumor recognition protein isoform X1 [Mus musculus].
(28)	XP_024100264	NK-tumor recognition protein isoform X5 [Pongo abelii].
(29)	XP_019666422	NK-tumor recognition protein isoform X3 [Felis catus].
(30)	XP_008530962	PREDICTED: NK-tumor recognition protein isoform X1 [Equus przewalskii].
(31)	XP_008530967	PREDICTED: NK-tumor recognition protein isoform X6 [Equus przewalskii].
(32)	XP_010944204	PREDICTED: NK-tumor recognition protein isoform X2 [Camelus bactrianus].
(33)	XP_015091830	PREDICTED: NK-tumor recognition protein isoform X3 [Vicugna pacos].
(34)	XP_014411884	PREDICTED: NK-tumor recognition protein isoform X2 [Camelus ferus].
(35)	XP_010944201	PREDICTED: NK-tumor recognition protein isoform X1 [Camelus bactrianus].
(36)	XP_009443554	PREDICTED: NK-tumor recognition protein isoform X4 [Pan troglodytes].
(37)	XP_021241242	NK-tumor recognition protein isoform X6 [Numida meleagris].
(38)	XP_020927360	NK-tumor recognition protein isoform X5 [Sus scrofa].
(39)	XP_004581770	PREDICTED: NK-tumor recognition protein [Ochotona princeps].
(40)	XP_005669469	NK-tumor recognition protein isoform X4 [Sus scrofa].

```
( 41)  XP_009443555     PREDICTED: NK-tumor recognition protein isoform X5 [Pan troglodytes].
( 42)  XP_005669468     NK-tumor recognition protein isoform X3 [Sus scrofa].
( 43)  XP_008708042     PREDICTED: LOW QUALITY PROTEIN: NK-tumor recognition protein [Ursus maritimus
( 44)  AAA37500         cyclophilin-related protein [Mus musculus domesticus].
( 45)  XP_004676331     PREDICTED: NK-tumor recognition protein isoform X1 [Condylura cristata].
( 46)  XP_006748020     PREDICTED: NK-tumor recognition protein [Leptonychotes weddellii].
( 47)  XP_006770577     PREDICTED: NK-tumor recognition protein isoform X4 [Myotis davidii].
( 48)  XP_019583095     PREDICTED: NK-tumor recognition protein isoform X4 [Rhinolophus sinicus].
( 49)  XP_015423867     PREDICTED: NK-tumor recognition protein isoform X3 [Myotis davidii].
( 50)  XP_005383594     PREDICTED: NK-tumor recognition protein isoform X1 [Chinchilla lanigera].
( 51)  XP_019583089     PREDICTED: NK-tumor recognition protein isoform X1 [Rhinolophus sinicus].
( 52)  XP_014445953     PREDICTED: NK-tumor recognition protein [Tupaia chinensis].
( 53)  XP_005891140     PREDICTED: NK-tumor recognition protein [Bos mutus].
( 54)  XP_019489104     PREDICTED: NK-tumor recognition protein isoform X4 [Hipposideros armiger].
( 55)  XP_012004520     PREDICTED: NK-tumor recognition protein isoform X2 [Ovis aries musimon].
( 56)  XP_006051863     PREDICTED: NK-tumor recognition protein isoform X4 [Bubalus bubalis].
( 57)  XP_005954297     PREDICTED: NK-tumor recognition protein [Pantholops hodgsonii].
( 58)  XP_005695639     PREDICTED: NK-tumor recognition protein isoform X2 [Capra hircus].
( 59)  XP_011954828     PREDICTED: NK-tumor recognition protein isoform X2 [Ovis aries].
( 60)  XP_003992255     NK-tumor recognition protein isoform X2 [Felis catus].
( 61)  XP_002702773     PREDICTED: NK-tumor recognition protein isoform X2 [Bos taurus].
( 62)  XP_019666421     NK-tumor recognition protein isoform X1 [Felis catus].
( 63)  XP_019489077     PREDICTED: NK-tumor recognition protein isoform X3 [Hipposideros armiger].
( 64)  XP_012004518     PREDICTED: NK-tumor recognition protein isoform X1 [Ovis aries musimon].
( 65)  XP_011954826     PREDICTED: NK-tumor recognition protein isoform X1 [Ovis aries].
( 66)  XP_006051860     PREDICTED: NK-tumor recognition protein isoform X1 [Bubalus bubalis].
( 67)  XP_005695638     PREDICTED: NK-tumor recognition protein isoform X1 [Capra hircus].
( 68)  XP_005196454     PREDICTED: NK-tumor recognition protein isoform X1 [Bos taurus].
( 69)  XP_851976        NK-tumor recognition protein isoform X2 [Canis lupus familiaris].
( 70)  XP_012520957     PREDICTED: NK-tumor recognition protein [Propithecus coquereli].
( 71)  XP_005634360     NK-tumor recognition protein isoform X1 [Canis lupus familiaris].
( 72)  XP_003132182     NK-tumor recognition protein isoform X2 [Sus scrofa].
( 73)  XP_020139644     NK-tumor recognition protein isoform X3 [Microcebus murinus].
( 74)  XP_014715584     PREDICTED: NK-tumor recognition protein isoform X3 [Equus asinus].
( 75)  XP_007193513     PREDICTED: NK-tumor recognition protein isoform X4 [Balaenoptera acutorostrat
( 76)  XP_005669463     NK-tumor recognition protein isoform X1 [Sus scrofa].
( 77)  XP_004419619     PREDICTED: NK-tumor recognition protein isoform X3 [Ceratotherium simum simum
( 78)  XP_003363180     NK-tumor recognition protein isoform X6 [Equus caballus].
( 79)  XP_014986734     PREDICTED: NK-tumor recognition protein isoform X3 [Macaca mulatta].
( 80)  XP_014715581     PREDICTED: NK-tumor recognition protein isoform X1 [Equus asinus].
( 81)  XP_014646446     PREDICTED: NK-tumor recognition protein isoform X2 [Ceratotherium simum simum
( 82)  XP_006893048     PREDICTED: LOW QUALITY PROTEIN: NK-tumor recognition protein [Elephantulus ed
```

```
( 83)  XP_012625718     NK-tumor recognition protein isoform X8 [Microcebus murinus].
( 84)  XP_010973061     PREDICTED: NK-tumor recognition protein isoform X2 [Camelus dromedarius].
( 85)  XP_010944206     PREDICTED: NK-tumor recognition protein isoform X3 [Camelus bactrianus].
( 86)  XP_007193512     PREDICTED: NK-tumor recognition protein isoform X3 [Balaenoptera acutorostrat
( 87)  XP_015091829     PREDICTED: NK-tumor recognition protein isoform X1 [Vicugna pacos].
( 88)  XP_014411882     PREDICTED: NK-tumor recognition protein isoform X1 [Camelus ferus].
( 89)  XP_006770574     PREDICTED: NK-tumor recognition protein isoform X2 [Myotis davidii].
( 90)  XP_006770573     PREDICTED: NK-tumor recognition protein isoform X1 [Myotis davidii].
( 91)  XP_003809334     PREDICTED: LOW QUALITY PROTEIN: NK-tumor recognition protein [Pan paniscus].
( 92)  ELK25111         NK-tumor recognition protein [Myotis davidii].
( 93)  XP_023087260     NK-tumor recognition protein isoform X3 [Piliocolobus tephrosceles].
( 94)  XP_005546833     PREDICTED: NK-tumor recognition protein isoform X2 [Macaca fascicularis].
( 95)  XP_008258471     PREDICTED: NK-tumor recognition protein isoform X3 [Oryctolagus cuniculus].
( 96)  XP_008258470     PREDICTED: NK-tumor recognition protein isoform X2 [Oryctolagus cuniculus].
( 97)  XP_004708608     PREDICTED: NK-tumor recognition protein [Echinops telfairi].
( 98)  XP_003309783     PREDICTED: NK-tumor recognition protein isoform X1 [Pan troglodytes].
( 99)  XP_009443557     PREDICTED: NK-tumor recognition protein isoform X6 [Pan troglodytes].
(100)  XP_011889503     PREDICTED: NK-tumor recognition protein isoform X7 [Cercocebus atys].
(101)  XP_019274386     PREDICTED: NK-tumor recognition protein isoform X3 [Panthera pardus].
(102)  XP_015392541     PREDICTED: NK-tumor recognition protein isoform X3 [Panthera tigris altaica].
(103)  XP_021573498     NK-tumor recognition protein [Carlito syrichta].
(104)  XP_017396461     PREDICTED: NK-tumor recognition protein isoform X4 [Cebus capucinus imitator]
(105)  XP_005546834     PREDICTED: NK-tumor recognition protein isoform X3 [Macaca fascicularis].
(106)  XP_014986732     PREDICTED: NK-tumor recognition protein isoform X1 [Macaca mulatta].
(107)  XP_018879079     PREDICTED: NK-tumor recognition protein isoform X3 [Gorilla gorilla gorilla].
(108)  XP_014986735     PREDICTED: NK-tumor recognition protein isoform X4 [Macaca mulatta].
(109)  XP_009443553     PREDICTED: NK-tumor recognition protein isoform X3 [Pan troglodytes].
(110)  XP_016861963     PREDICTED: NK-tumor recognition protein isoform X3 [Homo sapiens].
(111)  XP_011839703     PREDICTED: NK-tumor recognition protein [Mandrillus leucophaeus].
(112)  XP_006713234     PREDICTED: NK-tumor recognition protein isoform X2 [Homo sapiens].
(113)  XP_017396460     PREDICTED: NK-tumor recognition protein isoform X3 [Cebus capucinus imitator]
(114)  XP_018879080     PREDICTED: NK-tumor recognition protein isoform X4 [Gorilla gorilla gorilla].
(115)  XP_010372621     PREDICTED: NK-tumor recognition protein isoform X3 [Rhinopithecus roxellana].
(116)  XP_007981992     PREDICTED: NK-tumor recognition protein isoform X3 [Chlorocebus sabaeus].
(117)  XP_009199351     NK-tumor recognition protein isoform X2 [Papio anubis].
(118)  PNJ74565         NKTR isoform 5 [Pongo abelii].
(119)  XP_023087259     NK-tumor recognition protein isoform X2 [Piliocolobus tephrosceles].
(120)  XP_009237437     NK-tumor recognition protein isoform X3 [Pongo abelii].
(121)  XP_023087256     NK-tumor recognition protein isoform X1 [Piliocolobus tephrosceles].
(122)  XP_010372620     PREDICTED: NK-tumor recognition protein isoform X2 [Rhinopithecus roxellana].
(123)  XP_004414069     PREDICTED: NK-tumor recognition protein isoform X1 [Odobenus rosmarus diverge
(124)  XP_011231109     PREDICTED: NK-tumor recognition protein isoform X2 [Ailuropoda melanoleuca].
```

(125)	XP_015392540	PREDICTED: NK-tumor recognition protein isoform X2 [Panthera tigris altaica].
(126)	XP_011231110	PREDICTED: NK-tumor recognition protein isoform X1 [Ailuropoda melanoleuca].
(127)	XP_015392538	PREDICTED: NK-tumor recognition protein isoform X1 [Panthera tigris altaica].
(128)	XP_004754311	PREDICTED: NK-tumor recognition protein isoform X1 [Mustela putorius furo].
(129)	XP_011806342	PREDICTED: NK-tumor recognition protein [Colobus angolensis palliatus].
(130)	XP_005546832	PREDICTED: NK-tumor recognition protein isoform X1 [Macaca fascicularis].
(131)	XP_005546835	PREDICTED: NK-tumor recognition protein isoform X4 [Macaca fascicularis].
(132)	XP_023384831	NK-tumor recognition protein isoform X4 [Pteropus vampyrus].
(133)	XP_015447191	PREDICTED: NK-tumor recognition protein isoform X3 [Pteropus alecto].
(134)	XP_006868826	PREDICTED: NK-tumor recognition protein [Chrysochloris asiatica].
(135)	XP_006102363	NK-tumor recognition protein isoform X4 [Myotis lucifugus].
(136)	XP_005860445	PREDICTED: NK-tumor recognition protein isoform X4 [Myotis brandtii].
(137)	XP_019274385	PREDICTED: NK-tumor recognition protein isoform X2 [Panthera pardus].
(138)	XP_014323127	NK-tumor recognition protein isoform X3 [Myotis lucifugus].
(139)	XP_005860444	PREDICTED: NK-tumor recognition protein isoform X3 [Myotis brandtii].
(140)	XP_023988067	NK-tumor recognition protein isoform X4 [Physeter catodon].
(141)	XP_019274384	PREDICTED: NK-tumor recognition protein isoform X1 [Panthera pardus].
(142)	XP_017396459	PREDICTED: NK-tumor recognition protein isoform X2 [Cebus capucinus imitator]
(143)	XP_008152845	PREDICTED: NK-tumor recognition protein [Eptesicus fuscus].
(144)	NP_001336053	NK-tumor recognition protein isoform c [Homo sapiens].
(145)	XP_007106821	NK-tumor recognition protein isoform X3 [Physeter catodon].
(146)	XP_018879078	PREDICTED: NK-tumor recognition protein isoform X2 [Gorilla gorilla gorilla].
(147)	XP_017396458	PREDICTED: NK-tumor recognition protein isoform X1 [Cebus capucinus imitator]
(148)	XP_012315533	NK-tumor recognition protein isoform X7 [Aotus nancymaae].
(149)	XP_007981991	PREDICTED: NK-tumor recognition protein isoform X2 [Chlorocebus sabaeus].
(150)	XP_003256959	PREDICTED: LOW QUALITY PROTEIN: NK-tumor recognition protein [Nomascus leucog
(151)	XP_003735136	PREDICTED: NK-tumor recognition protein isoform X3 [Callithrix jacchus].
(152)	XP_019802415	PREDICTED: NK-tumor recognition protein isoform X4 [Tursiops truncatus].
(153)	XP_018879076	PREDICTED: NK-tumor recognition protein isoform X1 [Gorilla gorilla gorilla].
(154)	XP_012315532	NK-tumor recognition protein isoform X6 [Aotus nancymaae].
(155)	XP_011737181	PREDICTED: LOW QUALITY PROTEIN: NK-tumor recognition protein [Macaca nemestri
(156)	XP_011889502	PREDICTED: NK-tumor recognition protein isoform X6 [Cercocebus atys].
(157)	XP_010372619	PREDICTED: NK-tumor recognition protein isoform X1 [Rhinopithecus roxellana].
(158)	XP_007981990	PREDICTED: NK-tumor recognition protein isoform X1 [Chlorocebus sabaeus].
(159)	XP_004277919	PREDICTED: NK-tumor recognition protein [Orcinus orca].
(160)	XP_003894654	NK-tumor recognition protein isoform X1 [Papio anubis].
(161)	XP_019802412	PREDICTED: NK-tumor recognition protein isoform X1 [Tursiops truncatus].
(162)	XP_017811031	NK-tumor recognition protein isoform X3 [Papio anubis].
(163)	XP_008982345	PREDICTED: NK-tumor recognition protein isoform X8 [Callithrix jacchus].
(164)	XP_003477052	NK-tumor recognition protein isoform X2 [Cavia porcellus].
(165)	XP_013015643	NK-tumor recognition protein isoform X1 [Cavia porcellus].
(166)	XP_007499863	PREDICTED: NK-tumor recognition protein isoform X3 [Monodelphis domestica].

(167)	XP_023581750	NK-tumor recognition protein isoform X1 [Trichechus manatus latirostris].
(168)	XP_002813940	NK-tumor recognition protein isoform X2 [Pongo abelii].
(169)	XP_014390380	PREDICTED: NK-tumor recognition protein isoform X2 [Myotis brandtii].
(170)	XP_014323126	NK-tumor recognition protein isoform X2 [Myotis lucifugus].
(171)	XP_024100262	NK-tumor recognition protein isoform X1 [Pongo abelii].
(172)	XP_022409524	NK-tumor recognition protein isoform X4 [Delphinapterus leucas].
(173)	XP_022409523	NK-tumor recognition protein isoform X3 [Delphinapterus leucas].
(174)	XP_019802414	PREDICTED: NK-tumor recognition protein isoform X3 [Tursiops truncatus].
(175)	XP_021062318	NK-tumor recognition protein isoform X1 [Mus pahari].
(176)	XP_014390377	PREDICTED: NK-tumor recognition protein isoform X1 [Myotis brandtii].
(177)	XP_014323123	NK-tumor recognition protein isoform X1 [Myotis lucifugus].
(178)	XP_011889501	PREDICTED: NK-tumor recognition protein isoform X5 [Cercocebus atys].
(179)	XP_015344917	PREDICTED: NK-tumor recognition protein isoform X3 [Marmota marmota marmota].
(180)	XP_011889500	PREDICTED: NK-tumor recognition protein isoform X4 [Cercocebus atys].
(181)	XP_016018800	PREDICTED: NK-tumor recognition protein isoform X6 [Rousettus aegyptiacus].
(182)	XP_019802413	PREDICTED: NK-tumor recognition protein isoform X2 [Tursiops truncatus].
(183)	XP_023581752	NK-tumor recognition protein isoform X2 [Trichechus manatus latirostris].
(184)	XP_008847679	PREDICTED: NK-tumor recognition protein isoform X3 [Nannospalax galili].
(185)	XP_016018799	PREDICTED: NK-tumor recognition protein isoform X5 [Rousettus aegyptiacus].
(186)	XP_016018798	PREDICTED: NK-tumor recognition protein isoform X4 [Rousettus aegyptiacus].
(187)	XP_017497112	PREDICTED: NK-tumor recognition protein isoform X3 [Manis javanica].
(188)	XP_023371677	NK-tumor recognition protein isoform X3 [Otolemur garnettii].
(189)	XP_007947878	PREDICTED: NK-tumor recognition protein [Orycteropus afer afer].
(190)	XP_007445750	PREDICTED: NK-tumor recognition protein [Lipotes vexillifer].
(191)	XP_011889497	PREDICTED: NK-tumor recognition protein isoform X1 [Cercocebus atys].
(192)	XP_020854834	NK-tumor recognition protein isoform X2 [Phascolarctos cinereus].
(193)	XP_012315531	NK-tumor recognition protein isoform X5 [Aotus nancymaae].
(194)	XP_020854833	NK-tumor recognition protein isoform X1 [Phascolarctos cinereus].
(195)	XP_012315530	NK-tumor recognition protein isoform X4 [Aotus nancymaae].
(196)	XP_022409521	NK-tumor recognition protein isoform X1 [Delphinapterus leucas].
(197)	XP_023384830	NK-tumor recognition protein isoform X3 [Pteropus vampyrus].
(198)	XP_020041827	NK-tumor recognition protein isoform X1 [Castor canadensis].
(199)	XP_015344916	PREDICTED: NK-tumor recognition protein isoform X2 [Marmota marmota marmota].
(200)	XP_008847678	PREDICTED: NK-tumor recognition protein isoform X2 [Nannospalax galili].
(201)	XP_015344914	PREDICTED: NK-tumor recognition protein isoform X1 [Marmota marmota marmota].
(202)	XP_008847677	PREDICTED: NK-tumor recognition protein isoform X1 [Nannospalax galili].
(203)	XP_005337989	NK-tumor recognition protein isoform X4 [Ictidomys tridecemlineatus].
(204)	XP_021590709	NK-tumor recognition protein isoform X3 [Ictidomys tridecemlineatus].
(205)	XP_022409522	NK-tumor recognition protein isoform X2 [Delphinapterus leucas].
(206)	XP_021590708	NK-tumor recognition protein isoform X2 [Ictidomys tridecemlineatus].
(207)	XP_021027182	NK-tumor recognition protein isoform X3 [Mus caroli].
(208)	XP_006914886	PREDICTED: NK-tumor recognition protein isoform X2 [Pteropus alecto].

(209)	XP_011355685	NK-tumor recognition protein isoform X2 [Pteropus vampyrus].
(210)	XP_023384828	NK-tumor recognition protein isoform X1 [Pteropus vampyrus].
(211)	XP_006914885	PREDICTED: NK-tumor recognition protein isoform X1 [Pteropus alecto].
(212)	XP_012663507	NK-tumor recognition protein isoform X2 [Otolemur garnettii].
(213)	XP_012663506	NK-tumor recognition protein isoform X1 [Otolemur garnettii].
(214)	XP_017497113	PREDICTED: NK-tumor recognition protein isoform X4 [Manis javanica].
(215)	XP_016051563	PREDICTED: NK-tumor recognition protein isoform X1 [Miniopterus natalensis].
(216)	XP_017497111	PREDICTED: NK-tumor recognition protein isoform X2 [Manis javanica].
(217)	XP_017497110	PREDICTED: NK-tumor recognition protein isoform X1 [Manis javanica].
(218)	XP_012315529	NK-tumor recognition protein isoform X2 [Aotus nancymaae].
(219)	XP_020728074	NK-tumor recognition protein isoform X2 [Odocoileus virginianus texanus].
(220)	XP_012315528	NK-tumor recognition protein isoform X1 [Aotus nancymaae].
(221)	XP_020728073	NK-tumor recognition protein isoform X1 [Odocoileus virginianus texanus].
(222)	XP_021062319	NK-tumor recognition protein isoform X2 [Mus pahari].
(223)	XP_021027181	NK-tumor recognition protein isoform X2 [Mus caroli].
(224)	XP_021027180	NK-tumor recognition protein isoform X1 [Mus caroli].
(225)	XP_004835151	NK-tumor recognition protein isoform X2 [Heterocephalus glaber].
(226)	XP_012928271	NK-tumor recognition protein isoform X1 [Heterocephalus glaber].
(227)	XP_021590705	NK-tumor recognition protein isoform X1 [Ictidomys tridecemlineatus].
(228)	XP_011241241	PREDICTED: NK-tumor recognition protein isoform X2 [Mus musculus].
(229)	NP_035048	NK-tumor recognition protein [Mus musculus].
(230)	XP_017168694	PREDICTED: NK-tumor recognition protein isoform X4 [Mus musculus].
(231)	XP_005348185	PREDICTED: NK-tumor recognition protein isoform X1 [Microtus ochrogaster].
(232)	XP_005082370	NK-tumor recognition protein [Mesocricetus auratus].
(233)	XP_021503066	NK-tumor recognition protein isoform X5 [Meriones unguiculatus].
(234)	XP_021503065	NK-tumor recognition protein isoform X4 [Meriones unguiculatus].
(235)	XP_021503062	NK-tumor recognition protein isoform X1 [Meriones unguiculatus].
(236)	XP_021503063	NK-tumor recognition protein isoform X2 [Meriones unguiculatus].
(237)	XP_008568057	PREDICTED: NK-tumor recognition protein isoform X2 [Galeopterus variegatus].
(238)	XP_008568056	PREDICTED: NK-tumor recognition protein isoform X1 [Galeopterus variegatus].
(239)	XP_006120664	PREDICTED: NK-tumor recognition protein isoform X3 [Pelodiscus sinensis].
(240)	XP_007055629	PREDICTED: NK-tumor recognition protein [Chelonia mydas].
(241)	XP_006120661	PREDICTED: NK-tumor recognition protein isoform X1 [Pelodiscus sinensis].
(242)	XP_006120659	PREDICTED: NK-tumor recognition protein isoform X5 [Pelodiscus sinensis].
(243)	XP_007106819	NK-tumor recognition protein isoform X1 [Physeter catodon].
(244)	XP_015708506	PREDICTED: NK-tumor recognition protein isoform X1 [Coturnix japonica].
(245)	XP_009682494	PREDICTED: NK-tumor recognition protein isoform X4 [Struthio camelus australi
(246)	XP_009682493	PREDICTED: NK-tumor recognition protein isoform X3 [Struthio camelus australi
(247)	XP_009682492	PREDICTED: NK-tumor recognition protein isoform X2 [Struthio camelus australi
(248)	XP_009682491	PREDICTED: NK-tumor recognition protein isoform X1 [Struthio camelus australi
(249)	XP_019471734	PREDICTED: NK-tumor recognition protein isoform X5 [Meleagris gallopavo].
(250)	XP_010710549	PREDICTED: NK-tumor recognition protein isoform X2 [Meleagris gallopavo].

(251)	XP_010710548	PREDICTED: NK-tumor recognition protein isoform X1 [Meleagris gallopavo].
(252)	XP_015136632	PREDICTED: NK-tumor recognition protein isoform X7 [Gallus gallus].
(253)	XP_004939132	PREDICTED: NK-tumor recognition protein isoform X2 [Gallus gallus].
(254)	XP_004939131	PREDICTED: NK-tumor recognition protein isoform X1 [Gallus gallus].
(255)	XP_013795593	PREDICTED: NK-tumor recognition protein [Apteryx australis mantelli].
(256)	XP_019363610	PREDICTED: NK-tumor recognition protein isoform X1 [Gavialis gangeticus].
(257)	XP_014376468	PREDICTED: LOW QUALITY PROTEIN: NK-tumor recognition protein [Alligator sinen
(258)	XP_007659513	PREDICTED: NK-tumor recognition protein isoform X1 [Ornithorhynchus anatinus]
(259)	XP_021241246	NK-tumor recognition protein isoform X9 [Numida meleagris].
(260)	XP_021241236	NK-tumor recognition protein isoform X2 [Numida meleagris].
(261)	XP_021241235	NK-tumor recognition protein isoform X1 [Numida meleagris].
(262)	XP_017731869	PREDICTED: LOW QUALITY PROTEIN: NK-tumor recognition protein [Rhinopithecus b
(263)	XP_012426793	PREDICTED: NK-tumor recognition protein [Taeniopygia guttata].
(264)	XP_017944741	PREDICTED: NK-tumor recognition protein isoform X6 [Manacus vitellinus].
(265)	XP_017944740	PREDICTED: NK-tumor recognition protein isoform X5 [Manacus vitellinus].
(266)	XP_010393085	NK-tumor recognition protein isoform X6 [Corvus cornix cornix].
(267)	XP_017685518	PREDICTED: NK-tumor recognition protein isoform X5 [Lepidothrix coronata].
(268)	XP_017602246	PREDICTED: NK-tumor recognition protein isoform X6 [Corvus brachyrhynchos].
(269)	XP_016151723	PREDICTED: NK-tumor recognition protein isoform X4 [Ficedula albicollis].
(270)	XP_019138709	NK-tumor recognition protein isoform X5 [Corvus cornix cornix].
(271)	XP_017685517	PREDICTED: NK-tumor recognition protein isoform X4 [Lepidothrix coronata].
(272)	XP_017602245	PREDICTED: NK-tumor recognition protein isoform X5 [Corvus brachyrhynchos].
(273)	XP_005040539	PREDICTED: NK-tumor recognition protein isoform X3 [Ficedula albicollis].
(274)	XP_005040538	PREDICTED: NK-tumor recognition protein isoform X2 [Ficedula albicollis].
(275)	XP_005040537	PREDICTED: NK-tumor recognition protein isoform X1 [Ficedula albicollis].
(276)	XP_014800495	PREDICTED: NK-tumor recognition protein isoform X5 [Calidris pugnax].
(277)	XP_008168983	NK-tumor recognition protein isoform X3 [Chrysemys picta bellii].
(278)	XP_017944739	PREDICTED: NK-tumor recognition protein isoform X4 [Manacus vitellinus].
(279)	XP_014729203	PREDICTED: NK-tumor recognition protein isoform X4 [Sturnus vulgaris].
(280)	XP_017944736	PREDICTED: NK-tumor recognition protein isoform X2 [Manacus vitellinus].
(281)	XP_014729201	PREDICTED: NK-tumor recognition protein isoform X2 [Sturnus vulgaris].
(282)	XP_010393084	NK-tumor recognition protein isoform X4 [Corvus cornix cornix].
(283)	XP_017944735	PREDICTED: NK-tumor recognition protein isoform X1 [Manacus vitellinus].
(284)	XP_017685520	PREDICTED: NK-tumor recognition protein isoform X7 [Lepidothrix coronata].
(285)	XP_017602244	PREDICTED: NK-tumor recognition protein isoform X4 [Corvus brachyrhynchos].
(286)	XP_014729200	PREDICTED: NK-tumor recognition protein isoform X1 [Sturnus vulgaris].
(287)	XP_010393082	NK-tumor recognition protein isoform X2 [Corvus cornix cornix].
(288)	XP_017685515	PREDICTED: NK-tumor recognition protein isoform X2 [Lepidothrix coronata].
(289)	XP_017602242	PREDICTED: NK-tumor recognition protein isoform X3 [Corvus brachyrhynchos].
(290)	XP_017602241	PREDICTED: NK-tumor recognition protein isoform X2 [Corvus brachyrhynchos].
(291)	KFP58102	NK-tumor recognition protein [Cariama cristata].
(292)	XP_010393081	NK-tumor recognition protein isoform X1 [Corvus cornix cornix].

(293) XP_017685514 PREDICTED: NK-tumor recognition protein isoform X1 [Lepidothrix coronata].
(294) XP_017602240 PREDICTED: NK-tumor recognition protein isoform X1 [Corvus brachyrhynchos].
(295) XP_010559721 PREDICTED: NK-tumor recognition protein isoform X2 [Haliaeetus leucocephalus]
(296) XP_014800494 PREDICTED: NK-tumor recognition protein isoform X4 [Calidris pugnax].
(297) XP_011571332 PREDICTED: NK-tumor recognition protein isoform X2 [Aquila chrysaetos canaden
(298) XP_010559712 PREDICTED: NK-tumor recognition protein isoform X1 [Haliaeetus leucocephalus]
(299) XP_009331567 PREDICTED: NK-tumor recognition protein isoform X2 [Pygoscelis adeliae].
(300) XP_008492672 PREDICTED: NK-tumor recognition protein [Calypte anna].
(301) XP_014800492 PREDICTED: NK-tumor recognition protein isoform X2 [Calidris pugnax].
(302) XP_011571324 PREDICTED: NK-tumor recognition protein isoform X1 [Aquila chrysaetos canaden
(303) XP_009697766 PREDICTED: NK-tumor recognition protein [Cariama cristata].
(304) XP_009331566 PREDICTED: NK-tumor recognition protein isoform X1 [Pygoscelis adeliae].
(305) XP_009274213 PREDICTED: NK-tumor recognition protein isoform X1 [Aptenodytes forsteri].
(306) XP_014800491 PREDICTED: NK-tumor recognition protein isoform X1 [Calidris pugnax].
(307) XP_010301634 PREDICTED: NK-tumor recognition protein [Balearica regulorum gibbericeps].
(308) XP_008168980 NK-tumor recognition protein isoform X2 [Chrysemys picta bellii].
(309) XP_023963187 NK-tumor recognition protein isoform X1 [Chrysemys picta bellii].
(310) XP_005150066 PREDICTED: LOW QUALITY PROTEIN: NK-tumor recognition protein [Melopsittacus u
(311) XP_009473612 PREDICTED: LOW QUALITY PROTEIN: NK-tumor recognition protein [Nipponia nippon
(312) XP_021393646 NK-tumor recognition protein isoform X6 [Lonchura striata domestica].
(313) XP_021393642 NK-tumor recognition protein isoform X2 [Lonchura striata domestica].
(314) XP_021393643 NK-tumor recognition protein isoform X3 [Lonchura striata domestica].
(315) XP_009878793 PREDICTED: LOW QUALITY PROTEIN: NK-tumor recognition protein [Charadrius voci
(316) XP_021393641 NK-tumor recognition protein isoform X1 [Lonchura striata domestica].
(317) XP_018764676 PREDICTED: NK-tumor recognition protein isoform X4 [Serinus canaria].
(318) XP_018764674 PREDICTED: NK-tumor recognition protein isoform X2 [Serinus canaria].
(319) XP_018764673 PREDICTED: NK-tumor recognition protein isoform X1 [Serinus canaria].
(320) KFV02250 NK-tumor recognition protein [Pterocles gutturalis].
(321) XP_014125627 PREDICTED: NK-tumor recognition protein isoform X3 [Zonotrichia albicollis].
(322) XP_010083448 PREDICTED: NK-tumor recognition protein [Pterocles gutturalis].
(323) XP_014125626 PREDICTED: NK-tumor recognition protein isoform X2 [Zonotrichia albicollis].
(324) XP_014112250 PREDICTED: NK-tumor recognition protein isoform X3 [Pseudopodoces humilis].
(325) XP_014112249 PREDICTED: NK-tumor recognition protein isoform X2 [Pseudopodoces humilis].
(326) XP_014125625 PREDICTED: NK-tumor recognition protein isoform X1 [Zonotrichia albicollis].
(327) XP_014112248 PREDICTED: NK-tumor recognition protein isoform X1 [Pseudopodoces humilis].
(328) XP_005428264 PREDICTED: NK-tumor recognition protein [Geospiza fortis].
(329) XP_015474510 PREDICTED: NK-tumor recognition protein isoform X4 [Parus major].
(330) XP_023777798 NK-tumor recognition protein isoform X4 [Cyanistes caeruleus].
(331) XP_015474508 PREDICTED: NK-tumor recognition protein isoform X2 [Parus major].
(332) XP_023777797 NK-tumor recognition protein isoform X3 [Cyanistes caeruleus].
(333) XP_023777796 NK-tumor recognition protein isoform X2 [Cyanistes caeruleus].
(334) XP_015474507 PREDICTED: NK-tumor recognition protein isoform X1 [Parus major].

(335)	XP_023777795	NK-tumor recognition protein isoform X1 [Cyanistes caeruleus].
(336)	KFQ42818	NK-tumor recognition protein [Nestor notabilis].
(337)	XP_019355067	PREDICTED: NK-tumor recognition protein isoform X1 [Alligator mississippiensi
(338)	XP_010014722	PREDICTED: LOW QUALITY PROTEIN: NK-tumor recognition protein [Nestor notabili
(339)	XP_009556564	PREDICTED: NK-tumor recognition protein [Cuculus canorus].
(340)	XP_009993002	PREDICTED: NK-tumor recognition protein [Chaetura pelagica].
(341)	XP_009896433	PREDICTED: NK-tumor recognition protein [Picoides pubescens].
(342)	XP_010213065	PREDICTED: NK-tumor recognition protein [Tinamus guttatus].
(343)	XP_019384731	PREDICTED: NK-tumor recognition protein isoform X1 [Crocodylus porosus].
(344)	XP_008110438	PREDICTED: NK-tumor recognition protein isoform X2 [Anolis carolinensis].
(345)	XP_008110436	PREDICTED: NK-tumor recognition protein isoform X1 [Anolis carolinensis].
(346)	XP_015264278	PREDICTED: NK-tumor recognition protein [Gekko japonicus].
(347)	XP_013917336	PREDICTED: NK-tumor recognition protein [Thamnophis sirtalis].
(348)	XP_020670421	NK-tumor recognition protein isoform X2 [Pogona vitticeps].
(349)	XP_020670420	NK-tumor recognition protein isoform X1 [Pogona vitticeps].
(350)	XP_015667896	PREDICTED: LOW QUALITY PROTEIN: NK-tumor recognition protein [Protobothrops m
(351)	XP_007443413	PREDICTED: NK-tumor recognition protein isoform X2 [Python bivittatus].
(352)	XP_007443411	PREDICTED: NK-tumor recognition protein isoform X1 [Python bivittatus].
(353)	XP_017451684	PREDICTED: NK-tumor recognition protein isoform X1, partial [Rattus norvegicu
(354)	XP_014918330	PREDICTED: NK-tumor recognition protein [Acinonyx jubatus].
(355)	XP_002939352	PREDICTED: NK-tumor recognition protein isoform X1 [Xenopus tropicalis].
(356)	XP_018124467	PREDICTED: NK-tumor recognition protein-like isoform X1 [Xenopus laevis].
(357)	XP_017572959	PREDICTED: NK-tumor recognition protein isoform X1 [Pygocentrus nattereri].
(358)	XP_018607018	PREDICTED: NK-tumor recognition protein isoform X2 [Scleropages formosus].
(359)	XP_018607016	PREDICTED: NK-tumor recognition protein isoform X1 [Scleropages formosus].
(360)	XP_007544596	PREDICTED: NK-tumor recognition protein-like [Poecilia formosa].
(361)	XP_018607022	PREDICTED: NK-tumor recognition protein isoform X4 [Scleropages formosus].
(362)	XP_009443552	PREDICTED: NK-tumor recognition protein isoform X2 [Pan troglodytes].
(363)	PNJ74568	NKTR isoform 8 [Pongo abelii].
(364)	PNJ74566	NKTR isoform 6 [Pongo abelii].
(365)	XP_018122561	PREDICTED: NK-tumor recognition protein-like isoform X1 [Xenopus laevis].
(366)	XP_012735608	NK-tumor recognition protein isoform X1 [Fundulus heteroclitus].
(367)	XP_022065410	NK-tumor recognition protein isoform X6 [Acanthochromis polyacanthus].
(368)	XP_023122049	NK-tumor recognition protein isoform X6 [Amphiprion ocellaris].
(369)	XP_022065408	NK-tumor recognition protein isoform X4 [Acanthochromis polyacanthus].
(370)	XP_023122045	NK-tumor recognition protein isoform X3 [Amphiprion ocellaris].
(371)	XP_022065407	NK-tumor recognition protein isoform X3 [Acanthochromis polyacanthus].
(372)	XP_022065406	NK-tumor recognition protein isoform X2 [Acanthochromis polyacanthus].
(373)	XP_023122044	NK-tumor recognition protein isoform X2 [Amphiprion ocellaris].
(374)	XP_022065405	NK-tumor recognition protein isoform X1 [Acanthochromis polyacanthus].
(375)	XP_023122043	NK-tumor recognition protein isoform X1 [Amphiprion ocellaris].
(376)	XP_018414151	PREDICTED: NK-tumor recognition protein [Nanorana parkeri].

(377)	XP_023490562	NK-tumor recognition protein-like isoform X3 [Equus caballus].
(378)	XP_008304546	PREDICTED: NK-tumor recognition protein isoform X2 [Stegastes partitus].
(379)	KKF16876	NK-tumor recognition protein [Larimichthys crocea].
(380)	XP_008304545	PREDICTED: NK-tumor recognition protein isoform X1 [Stegastes partitus].
(381)	XP_015249666	PREDICTED: NK-tumor recognition protein isoform X1 [Cyprinodon variegatus].
(382)	XP_018529332	PREDICTED: NK-tumor recognition protein isoform X2 [Lates calcarifer].
(383)	XP_018529331	PREDICTED: NK-tumor recognition protein isoform X1 [Lates calcarifer].
(384)	XP_010751380	PREDICTED: NK-tumor recognition protein isoform X3 [Larimichthys crocea].
(385)	XP_010751371	PREDICTED: NK-tumor recognition protein isoform X2 [Larimichthys crocea].
(386)	XP_019745550	PREDICTED: NK-tumor recognition protein isoform X2 [Hippocampus comes].
(387)	XP_010751365	PREDICTED: NK-tumor recognition protein isoform X1 [Larimichthys crocea].
(388)	XP_019745545	PREDICTED: NK-tumor recognition protein isoform X1 [Hippocampus comes].
(389)	XP_023263705	NK-tumor recognition protein isoform X1 [Seriola lalandi dorsalis].
(390)	XP_023263706	NK-tumor recognition protein isoform X2 [Seriola lalandi dorsalis].
(391)	XP_001338072	NK-tumor recognition protein isoform X1 [Danio rerio].
(392)	XP_022599954	NK-tumor recognition protein isoform X2 [Seriola dumerili].
(393)	XP_019897364	PREDICTED: NK-tumor recognition protein isoform X6 [Esox lucius].
(394)	XP_010900666	PREDICTED: NK-tumor recognition protein isoform X4 [Esox lucius].
(395)	XP_010900665	PREDICTED: NK-tumor recognition protein isoform X3 [Esox lucius].
(396)	XP_010900664	PREDICTED: NK-tumor recognition protein isoform X2 [Esox lucius].
(397)	XP_019897363	PREDICTED: NK-tumor recognition protein isoform X1 [Esox lucius].
(398)	XP_010167755	PREDICTED: NK-tumor recognition protein-like, partial [Antrostomus carolinens
(399)	XP_010900668	PREDICTED: NK-tumor recognition protein isoform X5 [Esox lucius].
(400)	XP_016427080	PREDICTED: NK-tumor recognition protein-like isoform X1 [Sinocyclocheilus rhi
(401)	XP_016318510	PREDICTED: NK-tumor recognition protein-like isoform X1 [Sinocyclocheilus ans
(402)	XP_015209664	PREDICTED: NK-tumor recognition protein isoform X2 [Lepisosteus oculatus].
(403)	XP_015209662	PREDICTED: NK-tumor recognition protein isoform X1 [Lepisosteus oculatus].
(404)	XP_015209665	PREDICTED: NK-tumor recognition protein isoform X3 [Lepisosteus oculatus].
(405)	XP_005912346	PREDICTED: NK-tumor recognition protein isoform X2 [Haplochromis burtoni].
(406)	XP_005722738	PREDICTED: NK-tumor recognition protein isoform X2 [Pundamilia nyererei].
(407)	XP_020457914	LOW QUALITY PROTEIN: NK-tumor recognition protein [Monopterus albus].
(408)	XP_013132609	PREDICTED: NK-tumor recognition protein isoform X2 [Oreochromis niloticus].
(409)	XP_004546791	NK-tumor recognition protein isoform X2 [Maylandia zebra].
(410)	XP_006795195	PREDICTED: NK-tumor recognition protein-like isoform X2 [Neolamprologus brich
(411)	XP_005912345	PREDICTED: NK-tumor recognition protein isoform X1 [Haplochromis burtoni].
(412)	XP_005722737	PREDICTED: NK-tumor recognition protein isoform X1 [Pundamilia nyererei].
(413)	XP_021475699	NK-tumor recognition protein [Oncorhynchus mykiss].
(414)	XP_004546790	NK-tumor recognition protein isoform X1 [Maylandia zebra].
(415)	XP_003439241	PREDICTED: NK-tumor recognition protein isoform X1 [Oreochromis niloticus].
(416)	XP_006795194	PREDICTED: NK-tumor recognition protein-like isoform X1 [Neolamprologus brich
(417)	XP_023490560	NK-tumor recognition protein-like isoform X1 [Equus caballus].
(418)	XP_015209666	PREDICTED: NK-tumor recognition protein isoform X4 [Lepisosteus oculatus].

(419)	XP_005797888	NK-tumor recognition protein isoform X1 [Xiphophorus maculatus].
(420)	CBN81984	NK-tumor recognition protein [Dicentrarchus labrax].
(421)	XP_010559731	PREDICTED: NK-tumor recognition protein isoform X3 [Haliaeetus leucocephalus]
(422)	XP_007544415	PREDICTED: NK-tumor recognition protein isoform X1 [Poecilia formosa].
(423)	XP_014870682	PREDICTED: NK-tumor recognition protein isoform X1 [Poecilia latipinna].
(424)	XP_011606242	PREDICTED: NK-tumor recognition protein [Takifugu rubripes].
(425)	CAG04245	unnamed protein product, partial [Tetraodon nigroviridis].
(426)	XP_016887118	PREDICTED: NK-tumor recognition protein isoform X2 [Cynoglossus semilaevis].
(427)	XP_008306161	PREDICTED: NK-tumor recognition protein isoform X1 [Cynoglossus semilaevis].
(428)	XP_008394441	PREDICTED: NK-tumor recognition protein isoform X1 [Poecilia reticulata].
(429)	XP_014825423	PREDICTED: NK-tumor recognition protein isoform X1 [Poecilia mexicana].
(430)	XP_012398954	NK-tumor recognition protein isoform X8 [Sarcophilus harrisii].
(431)	XP_019063096	PREDICTED: NK-tumor recognition protein [Fukomys damarensis].
(432)	XP_009073803	PREDICTED: NK-tumor recognition protein-like, partial [Acanthisitta chloris].
(433)	XP_016400109	PREDICTED: NK-tumor recognition protein-like isoform X1 [Sinocyclocheilus rhi
(434)	XP_016359192	PREDICTED: NK-tumor recognition protein-like isoform X1 [Sinocyclocheilus ans
(435)	XP_004081327	NK-tumor recognition protein isoform X1 [Oryzias latipes].
(436)	XP_020568300	NK-tumor recognition protein isoform X2 [Oryzias latipes].
(437)	XP_016041020	PREDICTED: NK-tumor recognition protein [Erinaceus europaeus].
(438)	XP_017942181	PREDICTED: NK-tumor recognition protein-like isoform X1 [Manacus vitellinus].
(439)	XP_017942183	PREDICTED: NK-tumor recognition protein-like isoform X3 [Manacus vitellinus].
(440)	XP_017942182	PREDICTED: NK-tumor recognition protein-like isoform X2 [Manacus vitellinus].
(441)	XP_020501265	LOW QUALITY PROTEIN: NK-tumor recognition protein [Labrus bergylta].
(442)	KFP01329	NK-tumor recognition protein, partial [Calypte anna].
(443)	XP_015856948	PREDICTED: NK-tumor recognition protein isoform X3 [Peromyscus maniculatus ba
(444)	XP_020139631	NK-tumor recognition protein isoform X2 [Microcebus murinus].
(445)	XP_020139628	NK-tumor recognition protein isoform X1 [Microcebus murinus].
(446)	XP_006982975	PREDICTED: NK-tumor recognition protein isoform X2 [Peromyscus maniculatus ba
(447)	XP_015856947	PREDICTED: NK-tumor recognition protein isoform X1 [Peromyscus maniculatus ba
(448)	XP_014427753	PREDICTED: NK-tumor recognition protein isoform X4 [Pelodiscus sinensis].
(449)	XP_013857648	PREDICTED: NK-tumor recognition protein-like isoform X1 [Austrofundulus limna
(450)	XP_023656013	NK-tumor recognition protein isoform X2 [Paramormyrops kingsleyae].
(451)	XP_023656011	NK-tumor recognition protein isoform X1 [Paramormyrops kingsleyae].
(452)	XP_023656014	NK-tumor recognition protein isoform X3 [Paramormyrops kingsleyae].
(453)	XP_004448689	NK-tumor recognition protein isoform X2 [Dasypus novemcinctus].
(454)	XP_004448688	NK-tumor recognition protein isoform X1 [Dasypus novemcinctus].
(455)	XP_023476440	NK-tumor recognition protein isoform X2 [Equus caballus].
