# Supplementary material for: Compression of Large Sets of Sequence Data Reveals Fine Diversification of Functional Profiles in Multigene Families of Proteins: A Study for Peptidyl-Prolyl cis/trans Isomerases (PPIase)
Source: Biomolecules. 2019 Feb 11;9(2):59. doi: 10.3390/biom9020059 (PMC6406343; doi:10.3390/biom9020059)
Supplement: Supplementary file 1 [file biomolecules-09-00059-s001.zip › Figure S9A-MSA of FKBP38.aln.pdf]

CLUSTAL 2.0.9 multiple sequence alignment of FKBP38s

```
NP_036313           LRKKTLVPG-PPGSSRPVKGQVVTVHLQTSLE-NG--TRVQEEPELVFTLGDCDVIQALD
NP_001295302        LRKKTLVPG-PPGSSRPVKGQVVTVHLQTSLE-NG--TRVQEEPELVFTLGDCDVIQALD
XP_003817801        LRKKTLVPG-PPGSSRPVKGQVVTVHLQTSLE-NG--TRVQEEPELVFTLGDCDVIQALD
XP_010384957        LRKKTLVPG-PPGSSRPVKGQVVTVHLQTSLE-NG--TRVQEEPELVFTLGDCDVIQALD
XP_003817802        LRKKTLVPG-PPGSSRPVKGQVVTVHLQTSLE-NG--TRVQEEPELVFTLGDCDVIQALD
XP_011747960        LRKKTLVPG-PPGSSRPVKGQVVTVHLQTSLE-NG--TRVQEEPELVFTLGDCDVIQALD
XP_003915233        LRKKTLVPG-PPGSSRPVKGQVVTVHLQTSLE-NG--TRVQEEPELVFTLGDCDVIQALD
EHH59371            LRKKTLVPG-PPGSSRPVKGQVVTVHLQTSLE-NG--TRVQEEPELVFTLGDCDVIQALD
XP_005588521        LRKKTLVPG-PPGSSRPVKGQVVTVHLQTSLE-NG--TRVQEEPELVFTLGDCDVIQALD
XP_002828980        LRKKTLVPG-PPGSSRPVKGQVVTVHLQTSLE-NG--TRVQEEPELVFTLGDCDVIQALD
PNJ28395            LRKKTLVPG-PPGSSRPVKGQVVTVHLQTSLE-NG--TRVQEEPELVFTLGDCDVIQALD
XP_011949838        LRKKTLVPG-PPGSSRPVKGQVVTVHLQTSLE-NG--TRVQEEPELVFTLGDCDVIQALD
XP_018870801        LRKKTLVPG-PPGSSRPVKGQVVTVHLQTSLE-NG--TRVQEEPELVFTLGDCDVIQALD
XP_007993999        LRKKTLVPG-PPGSSRPVKGQVVTVHLQTSLE-NG--TRVQEEPELVFTLGDCDVIQALD
XP_014978797        LRKKTLVPG-PPGSSRPVKGQVVTVHLQTSLE-NG--TRVQEEPELVFTLGDCDVIQALD
XP_011747796        LRKKTLVPG-PPGSSRPVKGQVVTVHLQTSLE-NG--TRVQEEPELVFTLGDCDVIQALD
XP_003915232        LRKKTLVPG-PPGSSRPVKGQVVTVHLQTSLE-NG--TRVQEEPELVFTLGDCDVIQALD
XP_002828981        LRKKTLVPG-PPGSSRPVKGQVVTVHLQTSLE-NG--TRVQEEPELVFTLGDCDVIQALD
XP_010328455        LRKKTLVPG-PPGSSRPVKGQVVTVHLQTSLE-NG--TRVQEEPELVFTLGDCDVIQALD
XP_008969279        LRKKTLVPG-PPGSSRPVKGQVVTVHLQTSLE-NG--TRVQEEPELVFTLGDCDVIQALD
XP_011845848        LRKKTLVPG-PPGSSRPVKGQVVTVHLQTSLE-NG--TRVQEEPELVFTLGDCDVIQALD
XP_024207707        LRKKTLVPG-PPGSSRPVKGQVVTVHLQTSLE-NG--TRVQEEPELVFTLGDCDVIQALD
XP_011526166        LRKKTLVPG-PPGSSRPVKGQVVTVHLQTSLE-NG--TRVQEEPELVFTLGDCDVIQALD
XP_011949835        LRKKTLVPG-PPGSSRPVKGQVVTVHLQTSLE-NG--TRVQEEPELVFTLGDCDVIQALD
XP_017356796        LRKKTLVPG-PPGSSRPVKGQVVTVHLQTSLE-NG--TRVQEEPELVFTLGDCDVIQALD
XP_008969278        LRKKTLVPG-PPGSSRPVKGQVVTVHLQTSLE-NG--TRVQEEPELVFTLGDCDVIQALD
NP_001247999        LRKKTLVPG-PPGSSRPVKGQVVTVHLQTSLE-NG--TRVQEEPELVFTLGDCDVIQALD
XP_024307207        LRKKTLVPG-PPGSSRPVKGQVVTVHLQTSLE-NG--TRVQEEPELVFTLGDCDVIQALD
XP_016791013        LRKKTLVPG-PPGSSRPVKGQVVTVHLQTSLE-NG--TRVQEEPELVFTLGDCDVIQALD
XP_009192156        LRKKTLVPG-PPGSSRPVKGQVVTVHLQTSLE-NG--TRVQEEPELVFTLGDCDVIQALD
XP_024307210        LRKKTLVPG-PPGSSRPVKGQVVTVHLQTSLE-NG--TRVQEEPELVFTLGDCDVIQALD
XP_005588520        LRKKTLVPG-PPGSSRPVKGQVVTVHLQTSLE-NG--TRVQEEPELVFTLGDCDVIQALD
XP_011747874        LRKKTLVPG-PPGSSRPVKGQVVTVHLQTSLE-NG--TRVQEEPELVFTLGDCDVIQALD
XP_024092631        LRKKTLVPG-PPGSSRPVKGQVVTVHLQTSLE-NG--TRVQEEPELVFTLGDCDVIQALD
XP_004060362        LRKKTLVPG-PPGSSRPVKGQVVTVHLQTSLE-NG--TRVQEEPELVFTLGDCDVIQALD
XP_011949836        LRKKTLVPG-PPGSSRPVKGQVVTVHLQTSLE-NG--TRVQEEPELVFTLGDCDVIQALD
XP_007993998        LRKKTLVPG-PPGSSRPVKGQVVTVHLQTSLE-NG--TRVQEEPELVFTLGDCDVIQALD
XP_014978796        LRKKTLVPG-PPGSSRPVKGQVVTVHLQTSLE-NG--TRVQEEPELVFTLGDCDVIQALD
XP_009251263        LRKKTLVPG-PPGSSRPVKGQVVTVHLQTSLE-NG--TRVQEEPELVFTLGDCDVIQALD
XP_011747747        LRKKTLVPG-PPGSSRPVKGQVVTVHLQTSLE-NG--TRVQEEPELVFTLGDCDVIQALD
XP_017822960        LRKKTLVPG-PPGSSHPVKGQVVTVHLQTSLE-NG--TRVQEEPELVFTLGDCDVIQALD
XP_009192157        LRKKTLVPG-PPGSSRPVKGQVVTVHLQTSLE-NG--TRVQEEPELVFTLGDCDVIQALD
XP_010328454        LRKKTLVPG-PPGSSRPVKGQVVTVHLQTSLE-NG--TRVQEEPELVFTLGDCDVIQALD
XP_011845855        LRKKTLVPG-PPGSSRPVKGQVVTVHLQTSLE-NG--TRVQEEPELVFTLGDCDVIQALD
XP_012299012        LRKKTLVPG-PPGSSRPVQGQVVTVHLQTSLE-NG--TRVQEEPELVFTLGDCDVIQALD
XP_011949833        LRKKTLVPG-PPGSSRPVKGQVVTVHLQTSLE-NG--TRVQEEPELVFTLGDCDVIQALD
XP_017356795        LRKKTLVPG-PPGSSRPVKGQVVTVHLQTSLE-NG--TRVQEEPELVFTLGDCDVIQALD
XP_014401247        LRKKTLVPG-PPGSSRPTKGQVVTVQLQTSLE-NG--TRVQEEPELVFTLGDCDVIQALD
EPQ12447            LRKKTLVPG-PPGSSRPTKGQVVTVQLQTSLE-NG--TRVQEEPELVFTLGDCDVIQALD
XP_008154612        LRKKTLVPG-PPGSSRPAKGQVVTVQLQTSLE-NG--TRVQEEPELVFTLGDCDVIQALD
XP_016066232        LRKKTLVPG-PPGSSRPAKGQVVTVQLQTSLE-NG--TRVQEEPELVFTLGDCDVIQALD
XP_012584673        LRKKTLVPG-PPGSSRPAKGQVVTVQLQTSLE-NG--TRVQEEPELVFTLGDCDVIQALD
XP_015413209        LRKKTLVPG-PPGSSRPAKGQVVTVQLQTSLE-NG--TRVQEEPELVFTLGDCDVIQALD
XP_022262557        LRKKTLVPG-PPGSSRPAKGQVVTVQLQTSLE-NG--TRVQEEPELVFTLGDCDVIQALD
XP_023103313        LRKKTLVPG-PPGSSRPAKGQVVTVQLQTSLE-NG--TRVQEEPELVFTLGDCDVIQALD
```

```
XP_019271925        LRKKTLVPG-PPGSSRPAKGQVVTVQLQTSLE-NG--TRVQEEPELVFTLGDCDVIQALD
XP_007088417        LRKKTLVPG-PPGSSRPAKGQVVTVQLQTSLE-NG--TRVQEEPELVFTLGDCDVIQALD
XP_002761955        LRKKTLVPG-PPGSSHPVKGQVVTVHLQTSLE-NG--TRVQEEPELVFTLGDCDVIQALD
XP_014921066        LRKKTLVPG-PPGSSRPAKGQVVTVQLQTSLE-NG--TRVQEEPELVFTLGDCDVIQALD
XP_005972346        LRKKTLVPG-PPGSSRPTKGQVVTVRLQTSLE-NG--TRVQEEPELVFTLGDCDVIQALD
XP_006058025        LRKKTLVPG-PPGSSRPTKGQVVTVRLQTSLE-NG--TRVQEEPELVFTLGDCDVIQALD
XP_017905457        LRKKTLVPG-PPGSSRPTKGQVVTVRLQTSLE-NG--TRVQEEPELVFTLGDCDVIQALD
XP_019271923        LRKKTLVPG-PPGSSRPAKGQVVTVQLQTSLE-NG--TRVQEEPELVFTLGDCDVIQALD
XP_008542456        LRKKTLVPG-PPGSSRPTKGQVVTVQLQTSLE-NG--TRVQEEPELVFTLGDCDVIQALD
XP_006186302        LRKKTLVPG-PPGSSRPAKGQVVTVQLQTSLE-NG--TRVQEEPELVFTLGDCDVIQALD
XP_006199114        LRKKTLVPG-PPGSSRPAKGQVVTVQLQTSLE-NG--TRVQEEPELVFTLGDCDVIQALD
XP_010970466        LRKKTLVPG-PPGSSRPAKGQVVTVQLQTSLE-NG--TRVQEEPELVFTLGDCDVIQALD
XP_024416679        LRKKTLVPG-PPGSSRPTKGQVVTVQLQMSLE-NG--TRVQEEPELVFTLGDCDVIQALD
XP_005652984        LRKKTLVPG-PPGSSRPTKGQVVTVQLQTSLE-NG--TRVQEEPELVFTLGDCDVIQALD
XP_014687059        LRKKTLVPG-PPGSSRPTKGQVVTVQLQTSLE-NG--TRVQEEPELVFTLGDCDVIQALD
XP_015344756        LRKKTLVPG-PPGSSRPAKGQVVTVQLQMSLE-NG--TRVQEEPELVFTLGDCDVIQALD
XP_012299010        LRKKTLVPG-PPGSSRPVQGQVVTVHLQTSLE-NG--TRVQEEPELVFTLGDCDVIQALD
XP_019819388        LRKKTLVPG-PPGSSRPTKGQVVTVRLQTSLE-NG--TRVQEEPELVFTLGDCDVIQALD
ELW67147            LRKKTLVPG-PPGSSRPAKGQVVTVQLQTSLE-NG--TRVQEDPELVFTLGDCDVIQALD
XP_005873908        LRKKTLVPG-PPGSSRPTKGQVVTVQLQTSLE-NG--TRVQEEPELVFTLGDCDVIQALD
XP_016066229        LRKKTLVPG-PPGSSRPAKGQVVTVQLQTSLE-NG--TRVQEEPELVFTLGDCDVIQALD
XP_008154611        LRKKTLVPG-PPGSSRPAKGQVVTVQLQTSLE-NG--TRVQEEPELVFTLGDCDVIQALD
XP_020740155        LRKKTLVPG-PPGSSRPTKGQVVTVQLQTSLE-NG--TRVQEEPELTFTLGDCDVIQALD
XP_003982130        LRKKTLVPG-PPGSSRPAKGQVVTVQLQTSLE-NG--TRVQEEPELVFTLGDCDVIQALD
XP_019271924        LRKKTLVPG-PPGSSRPAKGQVVTVQLQTSLE-NG--TRVQEEPELVFTLGDCDVIQALD
XP_007088416        LRKKTLVPG-PPGSSRPAKGQVVTVQLQTSLE-NG--TRVQEEPELVFTLGDCDVIQALD
XP_541935           LRKKTLVPG-PPGSSRPAKGQVVTVQLQTSLE-NG--TRVQEEPELVFTLGDCDVIQALD
XP_012419293        LRKKTLVPG-PPGSSRPAKGQVVTVQLQTSLE-NG--TRVQEEPELVFTLGDCDVIQALD
XP_004688664        LRKKTLVPG-PPGSSRPAKGQVVTVQLQTSLE-NG--TRVQEEPELVFTLGDCDVIQALD
XP_015413208        LRKKTLVPG-PPGSSRPAKGQVVTVQLQTSLE-NG--TRVQEEPELVFTLGDCDVIQALD
XP_006747423        LRKKTLVPG-PPGSSRPAKGQVVTVQLQTSLE-NG--TRVQEEPELVFTLGDCDVIQALD
XP_017905455        LRKKTLVPG-PPGSSRPTKGQVVTVRLQTSLE-NG--TRVQEEPELVFTLGDCDVIQALD
XP_010993437        LRKKTLVPG-PPGSSRPAKGQVVTVQLQTSLE-NG--TRVQEEPELVFTLGDCDVIQALD
XP_006058026        LRKKTLVPG-PPGSSRPTKGQVVTVRLQTSLE-NG--TRVQEEPELVFTLGDCDVIQALD
XP_014921065        LRKKTLVPG-PPGSSRPAKGQVVTVQLQTSLE-NG--TRVQEEPELVFTLGDCDVIQALD
XP_006058024        LRKKTLVPG-PPGSSRPTKGQVVTVRLQTSLE-NG--TRVQEEPELVFTLGDCDVIQALD
XP_001503379        LRKKTLVPG-PPGSSRPTKGQVVTVQLQTSLE-NG--TRVQEEPELVFTLGDCDVIQALD
XP_005972345        LRKKTLVPG-PPGSSRPTKGQVVTVRLQTSLE-NG--TRVQEEPELVFTLGDCDVIQALD
XP_019271926        LRKKTLVPG-PPGSSRPAKGQVVTVQLQTSLE-NG--TRVQEEPELVFTLGDCDVIQALD
XP_006186301        LRKKTLVPG-PPGSSRPAKGQVVTVQLQTSLE-NG--TRVQEEPELVFTLGDCDVIQALD
XP_006199113        LRKKTLVPG-PPGSSRPAKGQVVTVQLQTSLE-NG--TRVQEEPELVFTLGDCDVIQALD
XP_010970465        LRKKTLVPG-PPGSSRPAKGQVVTVQLQTSLE-NG--TRVQEEPELVFTLGDCDVIQALD
XP_003123564        LRKKTLVPG-PPGSSRPTKGQVVTVQLQTSLE-NG--TRVQEEPELVFTLGDCDVIQALD
XP_024416678        LRKKTLVPG-PPGSSRPTKGQVVTVQLQMSLE-NG--TRVQEEPELVFTLGDCDVIQALD
NP_001192650        LRKKTLVPG-PPGSSRPTKGQVVTVRLQTSLE-NG--TRVQEEPDLVFTLGDCDVIQALD
XP_020740157        LRKKTLVPG-PPGSSRPTKGQVVTVQLQTSLE-NG--TRVQEEPELTFTLGDCDVIQALD
XP_015344757        LRKKTLVPG-PPGSSRPAKGQVVTVQLQMSLE-NG--TRVQEEPELVFTLGDCDVIQALD
XP_006147177        LRKKTLVPG-PPGSSRPAKGQVVTVQLQTSLE-NG--TRVQEDPELVFTLGDCDVIQALD
XP_020740156        LRKKTLVPG-PPGSSRPTKGQVVTVQLQTSLE-NG--TRVQEEPELTFTLGDCDVIQALD
XP_014687058        LRKKTLVPG-PPGSSRPTKGQVVTVQLQTSLE-NG--TRVQEEPELVFTLGDCDVIQALD
XP_004404706        LRKKTLVPG-PPGSSRPAKGQVVTVQLQTSLE-NG--TRVQEEPELVFTLGDCDVIQALD
XP_019819387        LRKKTLVPG-PPGSSRPTKGQVVTVRLQTSLE-NG--TRVQEEPELVFTLGDCDVIQALD
XP_020008573        LRKKTLVPG-PPGSGRPAKGQVVTVRLQMSLE-NG--TRVQEEPELAFTLGDCDVIQALD
XP_007116995        LRKKMLVPG-PPGSSRPAKGQVVTVQLQTSLE-NG--TRVQEEPELVFTLGDCDVIQALD
XP_024622992        LRKKMLVPG-PPGSSRPAKGQVVTVQLQTSLE-NG--TRVQEEPELVFTLGDCDVIQALD
XP_012391225        LRKKMLVPG-PPGSSRPAKGQVVTVQLQTSLE-NG--TRVQEEPELVFTLGDCDVIQALD
XP_020740154        LRKKTLVPG-PPGSSRPTKGQVVTVQLQTSLE-NG--TRVQEEPELTFTLGDCDVIQALD
XP_022414296        LRKKMLVPG-PPGSSRPAKGQVVTVQLQTSLE-NG--TRVQEEPELVFTLGDCDVIQALD
```

XP_021587090	LRKKTLVPG-PPGSSRPAKGQVVTVQLQMSLE-NG--TRVQEEPELVFTLGDCDVIQALD
XP_019592316	LRKKTLVPG-PPGSSRPTKGQVVTVQLQTSLE-NG--TRVQEEPELVFTLGDCDVIQALD
XP_023989754	LRKKMLVPG-PPGSSRPAKGQVVTVQLQTSLE-NG--TRVQEEPELVFTLGDCDVIQALD
XP_024622991	LRKKMLVPG-PPGSSRPAKGQVVTVQLQTSLE-NG--TRVQEEPELVFTLGDCDVIQALD
XP_004277581	LRKKMLVPG-PPGSSRPAKGQVVTVQLQTSLE-NG--TRVQEEPELVFTLGDCDVIQALD
XP_017651479	LRKKTLVPG-PPGSSRPLKGQVVTVHLQMSLE-SG--TRVQEEPELSFTLGDCDVIQALD
XP_022414295	LRKKMLVPG-PPGSSRPAKGQVVTVQLQTSLE-NG--TRVQEEPELVFTLGDCDVIQALD
XP_017651477	LRKKTLVPG-PPGSSRPLKGQVVTVHLQMSLE-SG--TRVQEEPELSFTLGDCDVIQALD
XP_007461122	LRKKMLVPG-PPGSSRPAKGQVVTVQLQTSLE-NG--TRVQEEPELVFTLGDCDVIQALD
XP_007195521	LRKKMLVPG-PPGSSRPAKGQVVTVQLQTSLE-NG--TRVQEEPELVFTLGDCDVIQALD
XP_019592317	LRKKTLVPG-PPGSSRPTKGQVVTVQLQTSLE-NG--TRVQEEPELVFTLGDCDVIQALD
XP_005001666	LRKKTLVPG-PPGSSRPTKGQVVTVQLQMSLE-NG--TRVQEEPELTFTLGDCDVIQALD
XP_008822765	LRKKTLVPG-PPGSSRPLKGQVVTVHLQMSLE-SG--TRVQEEPELSFTLGDCDVIQALD
XP_017651474	LRKKTLVPG-PPGSSRPLKGQVVTVHLQMSLE-SG--TRVQEEPELSFTLGDCDVIQALD
XP_013002970	LRKKTLVPG-PPGSSRPTKGQVVTVQLQMSLE-NG--TRVQEEPELTFTLGDCDVIQALD
XP_012924198	LRKKTLMPG-LPGSGRPTKGQVVTVQLQMSLE-NG--TRVQEEPELAFTLGDCDVIQALD
EHB02679	LRKKTLIPG-PPGSGRPTKGQVVTVQLQMSLE-NG--TRVQEEPELAFTLGDCDVIQALD
XP_012924197	LRKKTLMPG-LPGSGRPTKGQVVTVQLQMSLE-NG--TRVQEEPELAFTLGDCDVIQALD
XP_021535026	LRKKTLVPG-PPGSSRPAKGQVVTVQLQTSLE-NG--TRVQEEPELVFTLGDCDVIQALD
XP_007943554	LRKKTLVPG-PPGSSRPAKGQVVTVWLQTSLE-NG--TKVQEEPELVFTLGDCDVIQALD
XP_011220551	LRKKTLVPG-PPGSSRPAKGQVVTVQLQTSLE-NG--TRVQEEPGLVFTLGDCDVIQALD
XP_008588853	LRKKTLVPG-PPGSSRPAKGQVITVQLQMSLE-NG--TRVQEEPELVFTLGDCDVIQALD
XP_012916132	LRKKTLVPG-PPGSSRPAKGQVVTVQLQTSLE-NG--TRVQEEPELVFTLGDCDVIQALD
XP_022353384	LRKKTLVPG-PPGSSRPAKGQVVTVQLQTSLE-NG--TRVQEEPELVFTLGDCDVIQALD
XP_008048663	LRKKTLVPG-PPGSSHPAKGQVVTVHLQTSLE-NG--TRVQDELELVFTLGDCDVIQALD
XP_019652974	LRKKTLVPG-PPGSSRPAKGQVVTVQLQTSLE-NG--TRVQEEPGLVFTLGDCDVIQALD
XP_008702161	LRKKTLVPG-PPGSSHPAKGQVVTVQLQTSLE-NG--TRVQEEPELVFTLGDCDVIQALD
XP_019497623	LRKKTLVPG-PPGSSRPAKGQVVTVQLQTSLE-NG--TRVQEEPELVFTLGDCDVIQALD
XP_007943555	LRKKTLVPG-PPGSSRPAKGQVVTVWLQTSLE-NG--TKVQEEPELVFTLGDCDVIQALD
XP_002912777	LRKKTLVPG-PPGSSRPAKGQVVTVQLQTSLE-NG--TRVQEEPGLVFTLGDCDVIQALD
EFB19124	LRKKTLVPG-PPGSSRPAKGQVVTVQLQTSLE-NG--TRVQEEPGLVFTLGDCDVIQALD
XP_013362739	LRKKTLVPG-PPGSGRPTKGQVVTVQLQMSLE-NG--TRVQEEPELAFTLGDCDVIQALD
XP_006876371	LRKKTLVPG-PPGSSRPAKGQVVTVQLQTSLE-NG--TRVQEELELVFTLGDCDVIQALD
XP_021493693	LRKKTLVPG-PPGSSRPLKGQVVKVHLQMSLE-NG--TRVQEEPELAFTLGDCDVIQALD
XP_022353383	LRKKTLVPG-PPGSSRPAKGQVVTVQLQTSLE-NG--TRVQEEPELVFTLGDCDVIQALD
NP_001272500	LRKKTLVPG-PPGSSRPTKGQVVTVRLQTSLE-NG--TRVQEEPELVFTLGDCDVIQALD
XP_012916133	LRKKTLVPG-PPGSSRPAKGQVVTVQLQTSLE-NG--TRVQEEPELVFTLGDCDVIQALD
XP_011365085	LRKKTLVPG-PPGSSRPAKGQVVTVQLQTSLE-NG--TRVQEEPEVVFTLGDCDVIQALD
XP_006885938	LRKKTLVPG-PPGSSRPVKGQVVTVWLQTSLE-NG--TRVQEEPELMFTLGDCDVIQALD
XP_004423006	LRKKTLVPG-PPGSSRPTKGQVVTVQLQTSLE-NG--TRVQEEPELVFTLGDCDVIQALD
XP_012413338	LRKKTLVPG-PPGSSRPAKGQVVTVRLQTSLE-NG--TRVQEEPELVFTLGDCDVIQALD
XP_010591524	VRKKTLVPG-PPGSSRPAKGQVVTVRVQTSLE-NG--TRVQEEPELVFTLGDCDVIQALD
XP_008048662	LRKKTLVPG-PPGSSHPAKGQVVTVHLQTSLE-NG--TRVQDELELVFTLGDCDVIQALD
XP_015987916	LRKKTLVPG-PPGSSRPTKGQVVTVQLQTSLE-NG--TRVQEEPEVVFTLGDCDVIQALD
XP_004666075	LRKKTLVPG-PLGSGRPLKGQVVTVHLQMSLE-NG--TRVQEEPELVFTLGDCDVIQALD
XP_019497624	LRKKTLVPG-PPGSSRPAKGQVVTVQLQTSLE-NG--TRVQEEPELVFTLGDCDVIQALD
XP_004649071	LRKKTLVPG-PPGCNRPTKGQVVTVQLQMSLE-NG--TRVQEEPELSFTLGDCDVIQALD
APD32922	LRKKTLVPG-PPGSGRPAKGQVVTVRLQMALE-NG--TRVQEEPELAFTLGDCDVIQALD
XP_013362738	LRKKTLVPG-PPGSGRPTKGQVVTVQLQMSLE-NG--TRVQEEPELAFTLGDCDVIQALD
XP_012509480	LRKKTLVPG-PPGSSHPTRGQVVTVQLQTSLE-NG--TRVQEEPELVFTLGDCDVIQALD
NP_034353	LRMKTLVPG-PKGSSRPLKGQVVTVHLQMSLE-NG--TRVQEEPELAFTLGDCDVIQALD
XP_021074830	LRMKTLVPG-PKGSSRPLKGQVVTVHLQMSLE-NG--TRVQEEPELAFTLGDCDVIQALD
XP_006876370	LRKKTLVPG-PPGSSRPAKGQVVTVQLQTSLE-NG--TRVQEELELVFTLGDCDVIQALD
XP_021493691	LRKKTLVPG-PPGSSRPLKGQVVKVHLQMSLE-NG--TRVQEEPELAFTLGDCDVIQALD
XP_006904374	LRKKTLVPG-PPGSSRPAKGQVVTVQLQTSLE-NG--TRVQEEPEVVFTLGDCDVIQALD
XP_006989222	LRKKTLVPG-PPGSGRPLKGQVVTVHLHVSLE-NG--TRVQEEPELAFTLGDCDVIQALD
XP_004423005	LRKKTLVPG-PPGSSRPTKGQVVTVQLQTSLE-NG--TRVQEEPELVFTLGDCDVIQALD
XP_006885939	LRKKTLVPG-PPGSSRPVKGQVVTVWLQTSLE-NG--TRVQEEPELMFTLGDCDVIQALD

XP_004666073	LRKKTLVPG-PLGSGRPLKGQVVTVHLQMSLE-NG--TRVQEEPELVFTLGDCDVIQALD
ELK19193	LRKKTLVPG-PPGSSRPAKGQVVTVQLQTSLE-NG--TRVQEEPEVVFTLGDCDVIQALD
XP_003499206	LRKKTLVPG-PPGSTRPLKGQVVTVHLQMSLE-NG--TRVQEEPELAFTLGDCDVIQALD
XP_014637207	LRKKTLVPG-PPGSSRPTKGQVVTVQLQTSLE-NG--TRVQEEPELVFTLGDCDVIQALD
XP_012790031	LRKKTLIPG-PPGSERPTKGQVVTVRLQTALE-NG--TRVQDEPELQFTLGDCDVIQALD
XP_003413066	VRKKTLVPG-PPGSSRPAKGQVVTVRVQTSLE-NG--TRVQEEPELVFTLGDCDVIQALD
XP_004384434	LRKKTLVPG-PPGSSRPAKGQVVTVRLQTSLE-NG--TRVQEEPELVFTLGDCDVIQALD
XP_015987917	LRKKTLVPG-PPGSSRPTKGQVVTVQLQTSLE-NG--TRVQEEPEVVFTLGDCDVIQALD
XP_021078721	LRKKTLVPG-PPGSSRPLKGQVVTVYLQMSLE-NG--TRVQEEPELAFTLGDCDVIQALD
5MGX_E	LRKKTLVPG-PPGSSRPVKGQVVTVHLQTSLE-NG--TRVQEEPELVFTLGDCDVIQALD
XP_017168055	LRMKTLVPG-PKGSSRPLKGQVVTVHLQMSLE-NG--TRVQEEPELAFTLGDCDVIQALD
NP_001032257	LRKKTLVPG-PTGSSRPLKGQVVTVHLQMSLE-NG--TRVQEEPELAFTLGDCDVIQALD
XP_004595597	LRKKTLVPG-PPGSSRPSKGQVVTVHLHTSLE-NG--TRVQEDPELVFTLGDCDVIQALD
XP_017529992	LRKKTLVPG-LPGSSLPAQGQVVTVQLQTSLE-NG--TRVQEESELVFTLGDCDVIQALD
XP_005370624	LRKKTLVPG-PPGSSRPLKGQVVTVHLQMSLE-NG--THVQEEPELTFTLGDCDVIQALD
NP_001104536	LRMKTLVPG-PKGSSRPLKGQVVTVHLQMSLE-NG--TRVQEEPELAFTLGDCDVIQALD
XP_021074828	LRMKTLVPG-PKGSSRPLKGQVVTVHLQMSLE-NG--TRVQEEPELAFTLGDCDVIQALD
XP_012664880	LRKKTLVPG-PPGSSRPTKGQVVTVQLQTSLE-NG--TRVQDEPELMFTLGDCDVIQALD
XP_006989221	LRKKTLVPG-PPGSGRPLKGQVVTVHLHVSLE-NG--TRVQEEPELAFTLGDCDVIQALD
XP_007635977	LRKKTLVPG-PPGSTRPLKGQVVTVHLQMSLE-NG--TRVQEEPELAFTLGDCDVIQALD
XP_004616706	LRKKTLIPG-PPGSERPTKGQVVTVRLQTALE-NG--TRVQDEPELQFTLGDCDVIQALD
XP_012664879	LRKKTLVPG-PPGSSRPTKGQVVTVQLQTSLE-NG--TRVQDEPELMFTLGDCDVIQALD
XP_004705311	LRKKTLVPG-PPGSSRPAKGQVVTVRLQTSLE-NG--TRVQEEPELVFTLGDCDVIQALD
XP_017168054	LRMKTLVPG-PKGSSRPLKGQVVTVHLQMSLE-NG--TRVQEEPELAFTLGDCDVIQALD
XP_021026690	LRMKTLVPG-PKGSSRPLKGQVVTVHLQMSLE-NG--TRVQEEPELAFTLGDCDVIQALD
XP_004595596	LRKKTLVPG-PPGSSRPSKGQVVTVHLHTSLE-NG--TRVQEDPELVFTLGDCDVIQALD
XP_017529990	LRKKTLVPG-LPGSSLPAQGQVVTVQLQTSLE-NG--TRVQEESELVFTLGDCDVIQALD
XP_005370622	LRKKTLVPG-PPGSSRPLKGQVVTVHLQMSLE-NG--THVQEEPELTFTLGDCDVIQALD
XP_012883833	LRKKTLVPG-PPGAGRPTKGQVVTVRLQMSLE-DG--ARVQEEPELLFTLGDCDVIQALD
XP_021026691	LRMKTLVPG-PKGSSRPLKGQVVTVHLQMSLE-NG--TRVQEEPELAFTLGDCDVIQALD
XP_010849455	LRKKTLVPG-PPGSSRPTEGQVVTVRLQTSLE-NG--TRVQEEPELVFTLGDCDVIQALD
XP_010604777	LRKKTLVPG-PPGSGRPTKGQVVTVQLQMSLE-NG--TRVQEEPELSFTLGDCDVIQALD
XP_004705310	LRKKTLVPG-PPGSSRPAKGQVVTVRLQTSLE-NG--TRVQEEPELVFTLGDCDVIQALD
XP_021026689	LRMKTLVPG-PKGSSRPLKGQVVTVHLQMSLE-NG--TRVQEEPELAFTLGDCDVIQALD
XP_005898381	LRKKTLVPG-PPGSSRPTKGQVVTVRLQTSLE-NG--TRVQEEPELVFTLGDCDVIQALD
XP_012883832	LRKKTLVPG-PPGAGRPTKGQVVTVRLQMSLE-DG--ARVQEEPELLFTLGDCDVIQALD
XP_024622994	LRKKMLVPG-PPGSSRPAKGQVVTVQLQTSLE-NG--TR------------------ALD
XP_022414294	LRKKMLVPG-PPGSSRPAKGQVVTVQLQTSLE-NG--TR------------------ALD
XP_007116996	LRKKMLVPG-PPGSSRPAKGQVVTVQLQTSLE-NG--TR------------------ALD
XP_024622993	LRKKMLVPG-PPGSSRPAKGQVVTVQLQTSLE-NG--TR------------------ALD
XP_022414293	LRKKMLVPG-PPGSSRPAKGQVVTVQLQTSLE-NG--TR------------------ALD
XP_023055022	LRKKTLVPG-PPGSSRPVKGQVVTVHLQTSLE-NG--TRVQEEPELVFTLGDCDVIQALD
ELR54589	LRKKTLVPG-PPGSSRPTKGQVVTVRLQTSLE-NG--TRVQEEPELVFTLGDCDVIQALD
XP_010845609	LRKKTLVPG-PPGSSRPTKGQVVTVRLQ-------------------FTLGDCDVIQALD
XP_021538869	LRKKTLVPG-PPGSSRPAKGQVVTVQLQTSLE-NG--TRVQEEPELVFTLGDCDVIQALD
KFO22602	LRKKTLVPG-PPGSGRPTKGQVVTVQLQMSLE-NG--TRVQEEPELSFTLGDCDVIQALD
2MF9_A	LRKKTLVPG-PPGSSRPVKGQVVTVHLQTSLE-NG--TRVQEEPELVFTLGDCDVIQALD
PNI50452	LRKKTLVPG-PPGSSRPVKGQVVTVHLQTSLE-NG--TRVQEEPELVFTLGDCDVIQALD
PNJ28404	LRKKTLVPG-PPGSSRPVKGQVVTVHLQTSLE-NG--TRVQEEPELVFTLGDCDVIQALD
XP_007533227	LRKKTLIPG-PPGSARPVQGQVVTVRLQVSLE-SG--ARVLDEPELQVTLGDCDVIQALD
XP_020864537	LRKKILAAA-PPGAERPSKGQDVTVQLKVSLEDHG--TQVEEHPRLTFTLGDCDFLQALD
XP_012397649	LRKKIVVAA-PPGAERPTKGQDMTVQLKVSLEDDG--TQVEEEPSLTFTLGDCDFLQALD
XP_001369403	LKKKILVAA-PPGAERPTKGQDVTVHLKISLEEDG--TQVEEQPSITFTLGDCDFLQALD
2F2D_A	LRKKTLVPG-PPGSSRPVKGQVVTVHLQTSLE-NG--TRVQEEPELVFTLGDCDVIQALD
2D9F_A	LRKKTLVPG-PPGSSRPVKGQVVTVHLQTSLE-NG--TRVQEEPELVFTLGDCDVIQALD
XP_015668943	LKKKVIVSG-QGQESRPVKGQDVTVHFKATLEDG---TVVEEDPRLTFTLGDCDIIQALD
2AWG_A	LRKKTLVPG-PPGSSRPVKGQVVTVHLQTSLE-NG--TRVQEEPELVFTLGDCDVIQALD
XP_015668942	LKKKVIVSG-QGQESRPVKGQDVTVHFKATLEDG---TVVEEDPRLTFTLGDCDIIQALD

XP_025032464	LKKKVIISG-QGQESRPVKGQDVTIRLKVMLEDG---TVVEEDPSLTFTLGDCDVIQALD
XP_007443923	LKKKVIISG-QGQESRPVKGQDVTIRLKVMLEDG---TVVEEDPSLTFTLGDCDVIQALD
KFV73372	LKKKTLVPG-QGVESRPRKGQDVTVRLKATLEDG---SVVEENPSLTFTLGDCDVLQALD
OXB56359	LKKKTLVPG-QGVESRPRKGQEVTVRLRATLEDG---NVVEENPSLTFTLGDCDVLQALD
XP_009675861	LKKKTLVPG-QGVESRPRKGQDVTVRLKATLEDG---SVVEENPSLTFTLGDCDVLQALD
OXB73131	LKKKTLVPG-QGVESRPHKGQEVTVRLRATLEDG---NVVEENPSLTFTLGDCDVLQALD
XP_010723402	LKKKTLVPG-QGVESRPRKGQDVTVRLRATLEDG---SVVEENPSLTFTLGDCDVLQALD
KFV67807	LKKKTLVPG-QGVDTRPRKGQDVTIRLEARLEDG---SVVEEDPALTFTLGDCDVLQALD
XP_010304001	LKKKTLVPG-QGVDTRPRKGQDVTVRLKATLEDG---SVVEENPALTFTLGDCDVLQALD
NP_001241668	LKKKTLVPG-QGVESRPRKGQEVTVRLRATLEDG---NVVEENPSLTFTLGDCDVLQALD
KFO03599	LKKKTLVPG-QGVDTRPRKGQDVTVRLKATLEDG---SVVEENPALTFTLGDCDVLQALD
XP_021234495	LKKKTLMPG-QGVESRPRKGQEVTVRLRATLEDG---SVVEENPRLTFTLGDCDVLQALD
POI29828	LKKKTLVPG-QGVESRPRKGQEVTVRLRATLEDG---NVVEENPSLTFTLGDCDVLQALD
XP_021234496	LKKKTLMPG-QGVESRPRKGQEVTVRLRATLEDG---SVVEENPRLTFTLGDCDVLQALD
XP_023963943	LKKKTLVPG-QGMASRPRKGQDVTVRLKAMLEDG---SVVEDSPSLSFTLGDCDVMQALD
XP_014793985	LKKKTLVPG-QGVDTRPRKGQDVTIRLKATLEDG---SVVEENPALTFTLGDCDVLQALD
XP_009899401	LKKKTLVPG-QGVDTRPRKGQDVTIRLEARLEDG---SVVEEDPALTFTLGDCDVLQALD
XP_015742210	LKKKTLVPG-QGVESRPHKGQEVTVRLRATLDDG---TVVEENPSLTFTLGDCDVLQALD
XP_009641057	LKKKTLVPG-QGVDTRPHKGQDVTVRLKATLEDG---SVVEENPALTFTLGDCDVLQALD
KFP19261	LKKKTLVPG-QGVDTRPHKGQDVTVRLKATLEDG---SVVEENPALTFTLGDCDVLQALD
XP_024065593	LKKKTLVPG-QGVASRPRKGQDVTVRLKAMLEDG---SVVEDSPSLSFTLGDCDVMQALD
XP_011594878	LKKKTLVPG-QGVDTRPRKGQDVTVRLKATLEDG---SVVEENPALTFTLGDCDVLQALD
XP_014742726	LKKKTLVPG-QGVDSRPQKGQDVTIRLKATLEDG---TVVEEDPALTFTLGDCDVMQALD
XP_015742208	LKKKTLVPG-QGVESRPHKGQEVTVRLRATLDDG---TVVEENPSLTFTLGDCDVLQALD
XP_019369180	LKKKTLVPG-QGVESRPRKGQNVTVRLRVMLEDG---SVVEENPGLTFTLGDCDVLQALD
XP_010561556	LKKKTLVPG-QGVDTRPRKGQDVTVRLKATLEDG---SVVEENPALTFTLGDCDVLQALD
XP_008638175	LKKKTLVPG-QGADSRPQKGQDVTIRLKATLEDG---TVVEENPALTFTLGDCDVLQALD
XP_002195923	LKKKTLVPG-QGVDSRPQKGQDVTIRLKATLEDG---TVVEEDPALTFTLGDCDVLQALD
XP_015266773	LKKKVLVAG-QGRETRPVKNQDVTIRLKTTLEDG---TVVEENPSLTFTLGDCDVIQALD
XP_005505599	LKKKTLVPG-QGVDTRPNKGQNVTIRLKATLEDG---SVVEENPALTFTLGDCDVLQALD
XP_014115778	LKKKTLVPG-QGVDSRPQKGQDVTIRLKATLEDG---TVVEENPALTFTLGDCDVLQALD
XP_014742725	LKKKTLVPG-QGVDSRPQKGQDVTIRLKATLEDG---TVVEEDPALTFTLGDCDVMQALD
XP_018780521	LKKKTLVPG-QGVESRPQKGQDVTIRLKATLEDG---TVVEEDPALTFTLGDCDVLQALD
XP_005235043	LKKKTLVPG-QGVDTRPHKGQDVTVRLRAALEDG---TVVEENPALTFTLGDCDVLQALD
OPJ86694	LKKKTLLPG-RGVDTRPNKGQNVTIRLKATLEDG---SVVEENPALTFTLGDCDVLQALD
XP_019341692	LKKKTLVPG-RGMESRPSKGQVVTVRLRVMLEDG---SVVEEDPGLTFTLGDCDVLQALD
XP_021400798	LKKKTLVPG-QGADSRPQKGQDVTIRLKATLEDG---TVVEEDPALTFTLGDCDVLQALD
XP_006028906	LKKKTLVPG-QGAESRPRKGQDVTVRLRVMLEDG---SVVEEDPGLTFTLGDCDVLQALD
KFQ97904	LKKKTLVPG-QGVDTRPRKGQDVTVRLKATLEDG---SVVEENPALTFTLGDCDVLQALD
XP_015507158	LKKKTLVAG-QGVDSRPQKGQDVTIRLKATLEDG---TVVEENPALTFTLGDCDVLQALD
KFW12711	LKKKTLVPG-QGVDTRPRKGQDVTVRLKATLEDG---SVVEENPALTFTLGDCDVLQALD
XP_006271082	LKKKTLVPG-RGMESRPSKGQVVTVRLRVMLEDG---SVVEEDPGLTFTLGDCDVLQALD
KFO81655	LKKKTLVSG-QGVDTRPRKGQDVTIRLKAMLEDG---SVVEENPALTFTLGDCDVLQALD
XP_007072098	LKKKTLVPG-QGVESRPRKGQDVTVRLKVMLEDG---SVVEDNPSLSFTLGDCDVMQALD
XP_020669929	LKKKVLVAG-QGRETRPVRGQDVTVRLKATLEDG---SVVEEASTLTFTLGDCDIIHALD
XP_009464539	LKKKTLVPG-QGVDTRPRKGQDVTVRLKATLEDG---SVVEENPALTFTLGDCDVLQALD
XP_010158039	LKKKTLVPG-QGVDTRPRKGQDVTVRLKATLEDG---SVVEENPALTFTLGDCDVLQALD
XP_019149030	LKKKTLVPG-QGADSRPQKGQDVTIRLKATLEDG---TVVEENPALTFTLGDCDVLQALD
XP_020669930	LKKKVLVAG-QGRETRPVRGQDVTVRLKATLEDG---SVVEEASTLTFTLGDCDIIHALD
XP_019393900	LKKKTLVPG-QGVESRPRKGQNVTVRLRMMLEDG---SVVEENPGLTFTLGDCDVLQALD
EMP24085	LKKKTLVPG-QGVESRPRKGQDVTVRLKVMLEDG---SVVEDNPSLSFTLGDCDVMQALD
XP_009566597	LKKKTLVSG-QGVDTRPRKGQDVTIRLKAMLEDG---SVVEENPALTFTLGDCDVLQALD
KFW65305	LKKKTLVPG-QGVDPRPRKGQEVTVRLKATLEDG---SVVEENPALTFTLGDCDVLQALD
XP_015507157	LKKKTLVAG-QGVDSRPQKGQDVTIRLKATLEDG---TVVEENPALTFTLGDCDVLQALD
XP_013799130	LKKKTLVPG-RGVESRPHKGQDVTVRLKAMLEDS---SVVEENPSLTFTLGDCDVLQALD
XP_014433658	LRKKTLVPG-HGVESRPRKGQDVTVRLKVMLEDG---HVVEENPSLSFTVGDCDVMQALD
XP_009319696	LKKKTLVPG-QGVDPRPRKGQEVTVRLKATLEDG---SVVEENPALTFTLGDCDVLQALD
KFQ36923	LKKKTLVPG-QGVDTRPHKGQNVTIRLKATLEDGGG-SVVEENPALTFTLGDCDVLQALD

```
XP_005060257        LKKKTLVAG-QGVDSRPQKGQDVTIRLKATLEDG---TVVEEDPALTFTLGDCDVLQALD
KFU87694            LKKKTLVPG-RGVDTRPRKGQDVTIRLKATLQDG---RVVEENPALTFTLGDCDVLQALD
XP_005139681        LKKKTLVPG-RGGDTRPRKGQDVTVRLKAALQDG---RVVEENPALTFTLGDCDVVQALD
XP_009993397        LKKKTLVPG-RGVDTRPRKGQDVTIRLKATLQDG---RVVEENPALTFTLGDCDVLQALD
AAH84415            LKKKVLISG-KGAESRPRKGQDVTVQLKSMLENE---NVVEDQESLTFTLGDGDVIQALD
KQL50954            LKKKTLVPG-RGADTRPRKGQDVTVRLKAALEDG---SVVEESPALTFTLGDCDVLQALD
NP_001088346        LKKKVLISG-KGAESRPRKGQDVTVQLKSMLENE---NVVEDQESLTFTLGDGDVIQALD
XP_013913386        LKKKVLLPG-QGQETRPFRGQDVTIRFKAMLEDG---TVVEENPRLTFTLGDFDGIQALD
KFQ13009            LKKKTLVPG-QGVDTRPRKGQDVTIRLKATLEDG---SVVEENPALTFTLGDCDVLQALD
KFQ75436            VKQKTLVPG-QGVDTRPRKGQDVTVRLKATLEDG---SVVEENPALTFTLGDCDVLQALD
KFP56346            LKKKTLVPG-QGVDTRPRKGQDVTVRLKASLEDG---SVVEENPALTFTLGDCDVLQALD
KFM07869            LKKKTLVPG-QGVDTRPRKGQDVTVRLKATLEDG---SVVEENPALTFTLGDCDVLQALD
XP_018096952        LKKKVLISG-KGAESRPRKGQDVTVRLKSMLEDE---NVVEDQESLTFTLGDGDVIQALD
KFP65829            LKKKTLGPG-QGGDTRPCKGQDVTVRLKATLEDG---SVVEENPALTFTLGDCDVLQALD
XP_023803625        LKKKTLVPG-QGVDSRPQKGQDVTIRLKATLEDG---TVVEEDPALTFTLGDCDVLQALD
XP_009951725        LKKKTLVPG-QGVDTRPRKGQDVTIRLKATLEDG---SVVEENPALTFTLGDCDVLQALD
NP_001120570        LKKKILISG-KGADSRPRKGQDVTVGLKSMLEDG---NVVEEQETLTFTLGDGDVIQALD
KFP23912            LKKKTLVPG-QGVDTRPRKGQDVTIRLKATLEDG---SVVEENPALTFTLGDCDVLQALD
XP_010073720        LKKKTVVPG-QGVDTRPCKGQDVTIRLKATLEDG---SVVEENPALTFTLGDCDVLQALD
KGL86304            LQKKTLVPG-QGVDTRPRRGQDVTIRLKATLEDG---SVVEENPALTFTLGDCDVLQALD
KFV08597            LKKKTVVPG-QGVDTRPCKGQDVTIRLKATLEDG---SVVEENPALTFTLGDCDVLQALD
KFW76752            LKKKTLVPG-QGVDTRPRKGQDVTIRLKATLEDG---TVVEEDPALTFTLGDCDVLQALD
XP_009700019        LKKKTLGPG-QGGDTRPCKGQDVTVRLKATLEDG---SVVEENPALTFTLGDCDVLQALD
XP_010208130        LKKKTLVPG-QGVDTRPRKGQDVTIRLKATLEDG---SVVEENPALTFTLGDCDVLQALD
KFQ81502            LKKKTLVPG-QGVDTCPRKGQDVTIRLKAMLEDG---SVVEENPALTFTLGDCDVLQVLD
XP_017938939        LKKKTLVPG-QGVDTRPRKGQDVTIRLKATLEDG---TVVEEDPALTFTLGDCDVLQALD
KFP70760            LKKKTLVPG-QGVDTRPQKGQDVTIRLKATLDDG---TVVEEDPALTFTLGDCDVMQALD
XP_009880959        LQKKTLVPG-QGVDTRPRRGQDVTIRLKATLEDG---SVVEENPALTFTLGDCDVLQALD
XP_010292010        LKKKTLVPG-QGVDTCPRKGQDVTIRLKAMLEDG---SVVEENPALTFTLGDCDVLQVLD
XP_009076312        LKKKTLVPG-QGVDTRPQKGQDVTIRLKATLDDG---TVVEEDPALTFTLGDCDVMQALD
XP_018412986        LRKKVLIKG-QGVESRPKKGQDVTVKLTTSLKNG---SVVEEHVNYAFTLGDGDVMQSMD
XP_022525503        LRKKVLEPG-SEPDSKPQRGQIVTINLKTSLTDG---TTVEEESNISFTLGDGDVMQALD
XP_021459512        LRKKVLRAG-NGPDSRPTKGQNVVIHLKTSLADG---TLIEEQPELSFTLGDGDVIQALD
XP_021459510        LRKKVLRAG-NGPDSRPTKGQNVVIHLKTSLADG---TLIEEQPELSFTLGDGDVIQALD
XP_024277334        LRKKVLRAG-NGPDSRPTKGQNVVIHLKTSLADG---TLIEEQPELSFTLGDGDVIQALD
XP_024277330        LRKKVLRAG-NGPDSRPTKGQNVVIHLKTSLADG---TLIEEQPELSFTLGDGDVIQALD
XP_024277333        LRKKVLRAG-NGPDSRPTKGQNVVIHLKTSLADG---TLIEEQPELSFTLGDGDVIQALD
XP_009512288        --------------SRPRKGQDVTIRLKAMLEDG---SVVEENPALTFTLGDCDVLQALD
XP_020354512        LRKKVLRAG-NGPDSRPTKGQNVVIHLKTSLADG---TLIEEQPELSFTLGDGDVIQALD
KFZ50883            ------------VDTRPRKGQDVTVRLKATLEDG---RVVEENPALTFTLGDCDVLQALD
KFW81721            -------------DSRPRKGQDVTIRLKAMLEDG---SVVEENPALTFTLGDCDVLQALD
CDQ57258            LRKKVLRAG-NGPDSRPTKGQNVVIHLKTSLADG---TLIEEQPELSFTLGDGDVIQALD
XP_020354513        LRKKVLRAG-NGPDSRPTKGQNVVIHLKTSLADG---TLIEEQPELSFTLGDGDVIQALD
XP_020487811        LKKKVLEDG-EGRDSRPQKGQNVRINLKTSLTDG---KLVEEQHDLSFTLGDGDVIQALD
XP_024277331        LRKKVLRAG-NGPDSRPTKGQNVVIHLKTSLADG---TLIEEQPELSFTLGDGDVIQALD
XP_014071869        LRKKVLRAG-NGPDSRPTKGQNVVIHLKTSLADG---TLIEEQPELSFTLGDGDVIQALD
XP_011472153        LKKKVTAPG-KGRASRPQKGQNVRIHLKASLIDG---TLVEEQPNFSFTLGDCDVIQALD
XP_014071870        LRKKVLRAG-NGPDSRPTKGQNVVIHLKTSLADG---TLIEEQPELSFTLGDGDVIQALD
XP_024114895        LKKKVTASG-RGRASRPVKGQNVRIDLKTSLMDG---TVVEEQPDFSFTLGDGDVIQALD
XP_024114896        LKKKVTASG-RGRASRPVKGQNVRIDLKTSLMDG---TVVEEQPDFSFTLGDGDVIQALD
ETE64390            -----------RKESRPVKGQDVTIRFKATLEDG---TVVEEDPRLTFTLGDCDGIQALD
XP_023809890        LKKKVTAPG-KGRASRPQKGQNVRIHLKASLIDG---TLVEEQPNFSFTLGDCDVIQALD
XP_008499209        --------M-AGVDTRPRKGQEVTIRLKTSLEDG---RVGEEEPTLTFTLGDCDVLQALD
KFP06384            --------G-QGVDTRPRKGQEVTIRLKTSLEDG---RVGEEEPTLTFTLGDCDVLQALD
XP_023859426        LRKKVLRAG-NGPDSRPTKGQNVVIHLKTSLADG---TLVEEQPELSFTLGDGDVIQALD
XP_023859424        LRKKVLRAG-NGPDSRPTKGQNVVIHLKTSLADG---TLVEEQPELSFTLGDGDVIQALD
XP_023859423        LRKKVLRAG-NGPDSRPTKGQNVVIHLKTSLADG---TLVEEQPELSFTLGDGDVIQALD
XP_021454666        LKKKVLQVG-NGPDSRPTKGQNVMIHLKTSLADG---TLIEEQPELSFTLGDGDVIQALD
```

XP_017572299	LRKKVLEVG-SGPDSRPQRGQTVTINLKTTLTDG---TTVEEESNISFTLGDGDVIQALD
CDQ97962	LKKKVLQVG-NGPDSRPTKGQNVMIHLKTSLADG---TLIEEQPELSFTLGDGDVIQALD
XP_023854736	LKKKVLQAG-NGPDSRPTKGQNVVIHLKTSLADG---TLIEEQPELSFTLGDGDVIQALD
XP_018920592	LRKKVVEAG-AGPDSRPQRGQNVTIHLKTSLTNG---AVVEELPNLSFTLGDGDVLQALD
NP_001133417	LKKKVLQAG-NGPDSRPTKGQNVVIHLKTSLADG---TLIEEQPELSFTLGDGDVIQALD
XP_023854735	LKKKVLQAG-NGPDSRPTKGQNVVIHLKTSLADG---TLIEEQPELSFTLGDGDVIQALD
XP_024258320	LKKKVLQAG-HGPDSRPTKGQNVKIHLKTSLADG---TLIEEQPELSFTLGDGDVIQALD
AAI52651	LRKKVLEAG-AGPDSRPQKGQNVTIHLKTALTNG---TVVDELKDLSFTLGDGDVLQALD
XP_020348060	LKKKVLQAG-NGPDSRPTKGQNVKIHLKTSLADG---TLIEEQPELSFTLGDGDVIQALD
NP_957178	LRKKVLEAG-AGPDSRPQKGQNVTIHLKTALTNG---TVVDELKDLSFTLGDGDVLQALD
XP_005932512	LKKKVLKEG-EGRDSRPQKGQNVKIRLKTSLVDG---TLVEEKPDLAFTLGDGDVIQALD
XP_013860095	LKKKALEAG-RGRDSRPLKGQSVKINLKTHLMDG---TLIEEQHGLSFTLGDGDVIQALD
PWA20923	LKKKVLEPG-KGRASRPQKGQDVEINLKTQLMDG---TLVEEQNNLSFTLGDGDVIQALD
XP_005727139	LKKKVLKEG-EGRDSRPQKGQNVKIRLKTSLVDG---TLVEEKPDLTFTLGDGDVIQALD
XP_006790401	LKKKVLKEG-EGRDSRPQKGQNVKIRLKTSLVDG---TLVEEKPDLSFTLGDGDVIQALD
XP_019207270	LKKKVLKEG-EGRDSRPQKGQNVKIRLKTSLVDG---TVVEEKPDLAFTLGDGDVIQALD
XP_024654530	LKKKVLKEG-EGRDSRPQKGQNVKIRLKTSLVDG---TLVEEKPDFAFTLGDGDVIQALD
XP_007897075	LKKKVLVAG-RGELYQPQKGQNVTIRLKTSLEDG---TVLQEEPGLSFTLGDGDVIQALD
XP_010870009	LKKKVLQSG-NGPDTRPTKGQNVIIHLRTTLADG---TFIEEQPNFSFTLGDGDVIQGLD
XP_022615368	LKKKVLEAG-DGRDSRPQKGQNVKIHLKTYLKDG---ALVEEQSDLTFTLGDGDVIHALD
XP_010870008	LKKKVLQSG-NGPDTRPTKGQNVIIHLRTTLADG---TFIEEQPNFSFTLGDGDVIQGLD
XP_005795669	LKKKVLEPG-KGRASRPQKGQDVEINLKTLLMDG---TLVEEQNNLSFTLGDGDVIQALD
XP_020391166	LKKKVLVAG-QSQEPRPQKGQNVTVRLKMTLEDG---TEVEEDPNLCFTVGDGDVIQALD
AWP11099	LKKKVLVAG-QGRDSRPQKGQNVKTNLRTYLKDG---TLVEEQTGVTFTLGDSDVIQALD
XP_017333233	LRKKVLEAG-SGPDSKPQRGQNVTINLKTTLSDG---TTVEE-SKISFTLGDGDVIQALD
KTF74275	LRKKIVEAG-AGPDSRPQRGQIVTIDLKTTLTDG---TVVEELPNLSFTLGDGDVLQVLD
XP_017262938	LKKKALEAG-QGRDSRPLKGQNVKINLKTHLVDG---TLVEEQQDLSFTMGDGDVIQALD
XP_023263872	LKKKVLEAG-DGRDSRPQKGQNVKIHLKTYLKDR---ALVEEQSDLTFTLGDGDVIHALD
XP_020391165	LKKKVLVAG-QSQEPRPQKGQNVTVRLKMTLEDG---TEVEEDPNLCFTVGDGDVIQALD
XP_016327233	LRKKVVEAG-AGPDSRPQRGQNVTIHLKTSLTNG---TVVEELPNLSFTLGDGDVLQALD
XP_016138463	LRKKVVEAG-AGPDSRPQRGQNVTIHLKTSLTNG---TVVEELPNLSFTLGDGDVLQALD
XP_016356127	LRKKVVEAG-AGLDSRPQRGQNVTIHLKTTLTDG---TVVEELPNVSFTLGDGDVLQVLD
XP_007577281	LKKKVLEPG-KGRASRPQKGQDVEINLRTHLMDG---TLVEEQNNLSFTLGDGDVIQALD
XP_008405333	LKKKVLEPG-KGRASRPQKGQDVEINLRTHLMDG---TLVEEQNNLSFTLGDGDVIQALD
XP_012715520	LKKKVLEAG-KGRGSRPQKGQNVKINLKTHLMDG---ALVEEQHCLSFTLGDGDVLQALD
XP_012695070	LRKKVLEGG-EGLDTRPTRGQNVQIKLKTLLFDG---TIVEEDSELAFTLGDGDVIQALD
XP_020795782	LKKKVLVPG-KGRDSRPQKGQNVKINLKTTISDG---TVVVEISDLSFTLGDGDVIQALD
XP_016382508	LRKKVVEAG-AGPDSRPQRGQNVTIHLKTTLTDG---TVVEELPNVSFTLGDGDVLQVLD
XP_018522913	LKKKVLEAG-QGRDSRPQKGQNVKIHLKTYLKDG---KLVEEQSDLAFTLGDGDVIQALD
XP_008305299	LKKKVLEEG-QGRDSRPQKGQNVVINLKTCLKDG---TPVDEQTGLTFTLGDGDVIQAVD
XP_015238114	LKKKVVEAG-KGRDSRPQKGQNVEINLKTQLMDG---TLVDEQHNLSFTLGDGDVIQALD
XP_018583793	LKKKVLEAG-KGPDSKPTKGQNVKIYLKASLADG---AVVVEEPCLSFTVGDGDVIQALD
XP_016886204	LKKKVLEEG-QGRDSRPQKGQNVVINLKTCLKDG---TPVDEQTGLTFTLGDGDVIQAVD
XP_019752339	LKKKVLVAG-EGRESRPKKGQNVTICLKAFLKDG---TLVEEQPELTFTLGDGDVIQALD
XP_010728467	LKKKVLEAG-AGRDSRPQKGQNVKINLKTYLKDG---TPAAEEPNISFTLGDGDVIQALD
KKF19855	LKKKVLEAG-AGRDSRPQKGQNVKINLKTYLKDG---TPAAEEPNISFTLGDGDVIQALD
XP_015827013	LKKKVLEAG-KGRDSRPHKGQVVKIHLKTHLMDG---TLAQELCDLAFTLGDGDVIQALD
XP_022061518	LKKKVLEAG-QGRDSRPQKGQNVKINLKTYLMDG---TLVAEQPDLSFTLGDGDVLQALD
XP_019936960	LKKKVLEAG-EGRDSRPQKGQNVKINLKTSLKDG---TLVEEQSDVAFTLGDGDVFQALD
XP_003974097	LKKKVLEVG-EGRDSRPQKGQNVKIDLKTYTKDG---DLVAEEPELCFTLGNGDVIQAVD
XP_023699813	LKKKVLEAG-NGPDSKPQKGQNVKIFLKMSLSDG---TVVEEEPCLSFTLGDGDVIQALD
XP_023699812	LKKKVLEAG-NGPDSKPQKGQNVKIFLKMSLSDG---TVVEEEPCLSFTLGDGDVIQALD
XP_020478047	LKKKVVKAG-KGRDTRPQKGQNVKIHLKTYLKDG---TLVEEQSDLSFTLGDGDVIQALD
XP_015220721	LRKKVLAAG-RGPDSRPRKGQTVRVRLKASLQDG---SLVQEEPSLDFTLGDGDVIQALD
XP_023123034	LKKKVLEAG-EGQDSRPQKGQNVKINLKTYLMDG---TLVEEQPDLSFTLGDGDVFQALD
XP_020478046	LKKKVVKAG-KGRDTRPQKGQNVKIHLKTYLKDG---TLVEEQSDLSFTLGDGDVIQALD
XP_020478045	LKKKVVKAG-KGRDTRPQKGQNVKIHLKTYLKDG---TLVEEQSDLSFTLGDGDVIQALD
XP_008275730	LKIKALEAG-EGRDSRPQKGQNVKINLKTYLMDG---TLLVEQPDLSFTVGDGDVIQALD

XP_010770232	LKKKVLEEG-KGRDSRPQKGQNVKINLKTCLSDG---TLVEKQSDLSFTLGDGDVIQALD
XP_002601637	LKKKVIVKG-GDESTRPRPGQDVTIWSRGRLEDG---TTVDQHDEITFGLQEGDVIQAWD
XP_019619553	LKKKVIVKG-GDESTRPRPGQEVTIWSRGRLEDG---TTVDQHDEITFGLQEGDVIQAWD
XP_006814126	LKKKVLKPG-GGYRSRPARGDFVTVKYKTYLEND---NEVEDCESLKFILGDGDVIAAFD
XP_006629043	LRKRVLQPG-QEQAASPSWGQEVTVKLQGVLEDG---TVVEKDPRLTFVIGEGDVNQALE
PVD20167	LKKKVLNQG-REN-SRPTQGSLVTVKVQSFLENG---ICFQD-NTETFILGDGDTLSAID
XP_023841292	LRKKVLAPG-PQKAEHPAWGQEVTVKMQGVLEDR---SVVEKDRKLVFVIGEGDVNQALE
XP_008410571	LRKKILESG-DEQVPRSTWGQEVTVKMQGVLEDR---TVVEKDCKLVFVIGEGDVIQALE
XP_008410568	LRKKILESG-DEQVPRSTWGQEVTVKMQGVLEDR---TVVEKDCKLVFVIGEGDVIQALE
XP_022329628	LKRKILSKA-PKNAKRPISSDVVTIKVEGKLEDG---TKVDVFESLTFVLGDGDVIQAWD
XP_014897626	LRKKILESG-DEQVPRSTWGQEVTVKMQGVLEDR---TVVEKDCKLVFVIGEGDVIQALE
XP_014848641	LRKKILESG-DEQVPRSTWGQEVTVKMQGVLEDR---TVVEKDCKLVFVIGEGDVIQALE
XP_014848639	LRKKILESG-DEQVPRSTWGQEVTVKMQGVLEDR---TVVEKDCKLVFVIGEGDVIQALE
XP_007566466	LRKKILESG-DEQVPRSTWGQEVTVKMQGVLEDR---TVVEKDCKLVFVIGEGDVIQALE
XP_023180976	LRKKILESG-DEQVPRSTWGQEVTVKMQGVLEDR---TVVEKDCKLVFVIGEGDVIQALE
KMQ91381	LKKRVIRKG--ENGTRPNRGDICTLNITGKLE-DS--KVVEEYENLVIQLGDMEVIQGLD
XP_007566465	LRKKILESG-DEQVPRSTWGQEVTVKMQGVLEDR---TVVEKDCKLVFVIGEGDVIQALE
XP_014897625	LRKKILESG-DEQVPRSTWGQEVTVKMQGVLEDR---TVVEKDCKLVFVIGEGDVIQALE
XP_015254615	LRKKVLEPG-DIQVPHSTWGQEVTVKMQGVLEDR---TVVEKDCKLVFVIGEGDVIQALE
XP_005798327	LRKKILESG-DEQVPRSTWGQEVTVKMQGVLEDR---TVVEKDCKLVFVIGEGDVIQALE
XP_015254612	LRKKVLEPG-DIQVPHSTWGQEVTVKMQGVLEDR---TVVEKDCKLVFVIGEGDVIQALE
XP_013980328	LRKKVLAPG-PQKAEHPAWGQEVTVKMQGVLEDR---SVVEKDPKLVFVIGEGDVNQALE
XP_009061784	LTKKVLVQG-NGRESRPDRGDIVTLDIEGHLSDG---TAVDKYTAFTFPLGEGDVIQALD
XP_022106769	LMKKILREG-QGAASRPQRGEMVTLKSKGLLEDG---TEVDCHEAISFVLGEGDVVSAWD
XP_020625955	LKKRVLRQG-KGQDTRPDKGQRVTIQVAGRTEDG---TGVDWNSSLEFILGEGEVIQAFD
XP_020625956	LKKRVLRQG-KGQDTRPDKGQRVTIQVAGRTEDG---TGVDWNSSLEFILGEGEVIQAFD
XP_010732628	LRKKVLESS-APNALSPTWGQEVSVKMQCVLEDR---TVVEKDCKLVFVIGEGDVNQALE
XP_015728921	LRKRVVRDG-TG-G-APEPGQEVSVKVLGALQDG---AVVERDPRLSFVPGQGDVVQALE
XP_003440659	LRKKVLEPG-TPQGPHPAWDQEVTVKMQCVLEDR---TVVEKDRKLVFVIGEGDVNQALE
XP_014004032	LRKKVLVPG-PKQAEHPAWGQEVTVKMQGVLEDR---TVVEKDPKLVFVIGEGDVNQALE
XP_010899928	LRKKVLEPG-PKQAEHPAWGQEVTVKMQGLLEDG---TVVEKDPKLVFIIGEGDVNQALE
XP_020336380	LRKKVLVPG-PQQAEHPAWGQEVTVKMQGVLEDR---TVVEKDPKLVFVIGEGDVNQALE
XP_022330049	LKRKILSKA-PKNAKRPISSDVVTIKVEGNLEDG---TKVDVFESLTFVLGDGDVIQAWD
PIK48936	LQKKILTPG-KGHTTRPKADQIVTLRTKGWLEDG---TEIDWNDSITICLGDGDAVQAWD
XP_020336379	LRKKVLVPG-PQQAEHPAWGQEVTVKMQGVLEDR---TVVEKDPKLVFVIGEGDVNQALE
XP_022330048	LKRKILSKA-PKNAKRPISSDVVTIKVEGNLEDG---TKVDVFESLTFVLGDGDVIQAWD
XP_007907980	IQKKILREG-FGDASRPFMGQEVTMKLQGLLEDG---TIVDRDPKLTFVIGDGDVIQALE
XP_013859000	LRKKVLEPG-DAQVPLPSWGQEVTVRLQGVLEDR---TVVEKDCKLVFVIGESDVIQALE
XP_019737313	LGKKVLEPA-APGAPRPVRGQQAAVKMQAVLEDR---TVVEKDRELLFTIGEGDVSQALE
XP_021427237	LRKKVLAPG-PQKAEHPAWGQEVTVKMQGVLEDR---SVVEKDRKLVFVIGEGDVNQALE
XP_023827235	LRKKVLVPG-PQQAEHPSWGQEVTVKMQGVLEDR---TVVEKDPKLVFVIGEGDVNQALE
XP_023827230	LRKKVLVPG-PQQAEHPSWGQEVTVKMQGVLEDR---TVVEKDPKLVFVIGEGDVNQALE
XP_008309233	LRKKVLEPV-EPHVPHPTWGQEVTVKMQGVLEDH---TVVEKDWKLVFVVGEGDVTQALE
XP_024269308	LRKKVLVPG-PQQAEHPAWGQEVTVKMQGVLEDR---TVVEKDPKLVFVIGEGDVNQALE
XP_002932306	LKKKILKAG-HGKATIPLPGQEVTVHLMGILEDG---SLVEKDPKLSFVLDEGDVIQALE
XP_022785584	LQKRVLREG-KGQDTRPDKGQRVMIQSAGRTEDG---TAVDWNSNLEFILGEGEVIQAFD
XP_008309234	LRKKVLEPV-EPHVPHPTWGQEVTVKMQGVLEDH---TVVEKDWKLVFVVGEGDVTQALE
XP_013386268	LKKKVLKKG-DGLSSRPQRGQRPVISFSAHTEDG---TVVDSETQLMITLGDCDVVQAMD
XP_005928731	LRKKVLEPG-TPQGPHPAWDQEVTVKMQCVLEDR---TVVEKDCKLVFVIGEGDVNQALE
XP_002119960	LKKKTVTKG-QGRDTRPERGQEVTLDLETRSSDG---TILEEMHSVTFILGDHEVPESLD
XP_024235293	LRKKVLAPG-PQKAEHPAWGQEVTVKMQGVLEDR---SVVEKDRKLVFVIGEGDVNQALE
XP_005729996	LRKKVLEPG-TPQGPHPAWDQEVTVKMQCVLEDR---TVVEKDCKLVFVIGEGDVNQALE
XP_018619414	LRKKVLEQG-PEPSSRPTWGQEVTLRLQGVLEDR---TVVEKDSKLVFVIGEGDVNLALE
XP_012796312	IIKKTLKKG-LGGETRPSHGDSVVVNYKCWLEDG---TLVDDAENVKMVLGDGDIIHAFD
XP_004553258	LRKKVLEPG-TPQGPHPAWDQEVTVKMQCVLEDR---TVVEKDCKLVFVIGEGDVNQALE
CDQ72337	LRKKVLVPG-PQQAEHPAWGQEVTVKMQGVLEDR---TVVEKDPKLVFVIGEGDVNQALE
XP_024269303	LRKKVLVPG-PQQAEHPAWGQEVTVKMQGVLEDR---TVVEKDPKLVFVIGEGDVNQALE
XP_024269307	LRKKVLVPG-PQQAEHPAWGQEVTVKMQGVLEDR---TVVEKDPKLVFVIGEGDVNQALE

```
XP_024269300        LRKKVLVPG-PQQAEHPAWGQEVTVKMQGVLEDR---TVVEKDPKLVFVIGEGDVNQALE
XP_017893134        LKKKVIKQG--KNGTRPNRSDICILDIIGKLQ-DG--TKVEEYKNLKIQLGDVEVIQGLD
XP_021454564        LRKKVLVPG-PQQAEHPAWGQEVTVKMQGVLEDR---TVVEKDPKLVFVIGEGDVNQALE
XP_003967095        LRKKVLESS-DPSGPSPSWGQEVTVKMQCVLEDR---TVVEKDSKLVFVIGEGDVNQALE
XP_021454548        LRKKVLVPG-PQQAEHPAWGQEVTVKMQGVLEDR---TVVEKDPKLVFVIGEGDVNQALE
XP_023697743        LRKKIVQQG-LQQWTRPVWGQEVTVKLQGILEDR---TVVEKDSKLVFVTGEGDVHQALE
PIK51124            LLKKILTPG-KGRTTNPKADQIVTLRTKGWLEDG---TEVDWNDSITICLGDGDAVQAWD
XP_020311464        LRKKVLAPG-PQKAEHPAWGQEVTVKMQGVLEDR---SVVEKDRKLVFVIGEGDVNQALE
XP_017261735        LRKKVLKPG-NPQVPGPSWGQEVTVRMQGVLEDR---TVVEKDCNLVFVIGERDVIQALE
XP_011312767        LRKKTLTKG--TNNTRPQRNDICTIDIVGRLE-DGT-E-VECLPDFVVQLGDVELIQGLD
XP_022047038        LRKKVLKCG-TLQAPHPTWGQEVTVKMQCVLEDR---TVVEKDCKLVFVIGEGDVNQALE
XP_003427294        LRKKVLKAG--KENSRPNRSDVCVLKFTGRLE-DG--TIVEEEEDIKIQLGDMEVIQGLD
XP_020139363        ------GPG--GFAQRGPDGSGSGAASAPALFVP---PTPQPHFPISAVRGESDTWGAHS
XP_011661975        LRKKVLKAG-QGEAARPDRGMAMTVRYKGMLEDG---TEVEGEEKATFTQGEGEIVQAID
XP_021263191        LRKRVVRDG-QG-G-APQPGQEVSVKVLGALEDG---GLVERDPRLSFVPGQGDAVQALE
XP_005110253        LKKKVLVPG-K-EDTRPTNGDTVTIRVDGVLENG---THVD-VQDFTFILNDGDVILAFD
OCT89685            LKKKILKAG-RGRATIPLPGQEVTIHLMGILEDG---SLVEIDPKLSFVLDEGDVIQALE
XP_018106932        LKKKILKAG-RGRATIPLPGQEVTIHLMGILEDG---SLVEIDPKLSFVLDEGDVIQALE
XP_022609905        LRKKVLEPV-APHSLCPTWGQEVTVKMQCVLEDR---TVVEKDGKLVFVIGEGDVNQALE
XP_023278137        LRKKVLEPV-APHSLCPTWGQEVTVKMQCVLEDR---TVVEKDGKLVFVIGEGDVNQALE
XP_023151164        LRKKVLKCG-TLQAPHPTWGQEVTVKMQCVLEDR---TVVEKDGKLVFVIGEGDVNQALE
OON20683            ITKKILTKG-LGRESRPNHGDLVEISFKGYLEDG---TIVDDVASMVCTLGDGDVIQALD
XP_014340350        LRKKIMKEG-DTEAAKPLPGQEVTVKLQGAMEDG---TFVEKDPKLTFVIEEGDVIQALE
XP_012284026        LIKTVLKPG--KKGTRPNKSDICTLKMSGKLE-DG--TIVEEYEELIIQLGDVEVVQGLD
OAD61952            LKMKVIKSG--KDGTRPNRSDICILKIIGKLK-DG--TIVEEYEDLKIQLGDVEVIQGLD
XP_023962949        LRKLLVREG-QGEASRPQPGQEVTVKLLGVLEDG---SLVEKDPKLTFILEQGDVIQALE
XP_008121023        LRKCLVKQG-HG--SRPQAGQEMAVKLLGALEDG---SLVERDPRLTFVLGQWEAIQALE
XP_017763036        LKMKVIKSG--KDGTRPNRSDICILKIIGKLK-DG--TIVEEYEDLKIQLGDVEVIQGLD
XP_017691650        LRKRVVRAG-LG-GPQPRPGQEVSVKVLGALEDG---GLVERDPRLTFVPGHGDVVQALE
XP_012710521        LRKKVLQSG-DIHVPHATWDQEVTVKMQGVVEDR---TVVEKDCKLVFVIGEGDVIQALE
XP_015819997        LRKKVLQNG-DSQVLHPVWGQEVTVKMQGVLEDR---TVVEKDCKLVFVIGEGDVIQALE
XP_023962948        LRKLLVREG-QGEASRPQPGQEVTVKLLGVLEDG---SLVEKDPKLTFILEQGDVIQALE
XP_019921720        LKRKILRAA-AKNAKRPISSDLVTIKVEGKLEDG---TKVDVYDSLSFVLGDGDVIQAWD
XP_020904887        LKRKILREG-KGRDTRPDRGQIVMIRTAGRLSDG---TPVDWNQCLDFILGEGEVIQAWD
XP_024138547        LRKKVLKCG-DVQVPRPTWGQEVTIMMQGVLEDR---TVVEKDCKLVFVIGEGDVNQALE
AWP08482            LRKKVLEPV-APHALSPIWGQEVTMKMQCVLEDR---TVVEKDRKLVFVIGEGDVNQALE
CDQ67637            LRKKVLAPG-PQKAEHPAWGQEVTVKMQGVLEDR---SVVEKDRKLVFVIGEGDVNQALE
XP_011423840        LKRKILRAA-AKNAKRPISSDLVTIKVEGKLEDG---TKVDVYDSLSFVLGDGDVIQAWD
XP_014808190        LRKRVVQDG-QG-GPRPQPGQEVSVKVLGALEDG---GLVERDPRLTFVPGHGDVVQALE
XP_007059625        LRKLVVREG-QGEAGRPQPGQEVTVKLLGVLEDG---SLVEKDPMLTFTLEQGDVIQALE
XP_024138546        LRKKVLKCG-DVQVPRPTWGQEVTIMMQGVLEDR---TVVEKDCKLVFVIGEGDVNQALE
EMP36068            LRKLVVREG-QGEAGRPQPGQEVTVKLLGVLEDG---SLVEKDPMLTFTLEQGDVIQALE
XP_024138543        LRKKVLKCG-DVQVPRPTWGQEVTIMMQGVLEDR---TVVEKDCKLVFVIGEGDVNQALE
OXU31097            LRKKVLKAG--KENSRPNRSDVCVLKFTGRLE-DG--TIVEEEEDIKIQLGDMEVIQGLD
XP_022202921        LKKKVIREG--KPDTRPQRADICQVKFEGRLE-NGE--IIEKEDCLTINIGDGEVIQGLD
XP_006557516        LKKKVIKNG--KNGTRPNRSDICTLKIIGKLK-DN--TIVEKYEDLKIQLGDVELIQGLD
XP_016770764        LKKKVIKNG--KNGTRPNRSDICTLKIIGKLK-DN--TIVEKYEDLKIQLGDVELIQGLD
XP_006615021        LKKKVIKNG--KNGTRPNRSDICTLKIIGKLK-DN--TIVEKYEDLKIQLGDVELIQGLD
XP_008546855        LKKKTIVKG--QKGTRPNQGDLCTVDITGSLTPDGK-EVIETLKAVTVQLGDHEVIQGLE
XP_006557515        LKKKVIKNG--KNGTRPNRSDICTLKIIGKLK-DN--TIVEKYEDLKIQLGDVELIQGLD
XP_007254541        LRKKVLEAG-PERAPCPVWGQEVTLKMQGVLEDR---TMVEKDCKLVFVIGEGDVNQALE
XP_006615020        LKKKVIKNG--KNGTRPNRSDICTLKIIGKLK-DN--TIVEKYEDLKIQLGDVELIQGLD
XP_006557514        LKKKVIKNG--KNGTRPNRSDICTLKIIGKLK-DN--TIVEKYEDLKIQLGDVELIQGLD
XP_011053698        LMKKVIKKG--TKDVKPIQKDICTLKFTGVLD-DG--TVVEDQNNISIQLGDFEIVQGLD
XP_007254540        LRKKVLEAG-PERAPCPVWGQEVTLKMQGVLEDR---TMVEKDCKLVFVIGEGDVNQALE
XP_006615019        LKKKVIKNG--KNGTRPNRSDICTLKIIGKLK-DN--TIVEKYEDLKIQLGDVELIQGLD
XP_016913411        LKKKVIKNG--KNGTRPNRSDMCTLKIIGKLK-DN--TIVEKYEDLKIQLGDVELIQGLD
XP_017559733        LRKKILEPG-PEQAPCPVWGQEVMLKMQGVLEDR---TMVEKDSKLVFVIGEGDVNQALE
```

PKU40687 LRKRVVQDG-QG-GPRPQPGQEVSVKVLGALEDG---GLVERDPRLTFVPGHGDVVQALE
XP_016913410 LKKKVIKNG--KNGTRPNRSDMCTLKIIGKLK-DN--TIVEKYEDLKIQLGDVELIQGLD
EGI65888 LMKKVIKKG--TKDVKPIQKDICTLKFTGVLD-DG--TVVEDQNNISIQLGDFEIVQGLD
XP_014252350 LKKKVLKPG-EP-DSRPTKPDVCEISFEGRLEDG---TVVDKEDRIEVFLGDNDTAQGID
XP_012165944 LRKKVIKSG--KNGTRPNRSDICTLKITGKLK-DG--TTVEEYEDLKIQLGDVEVIQGLD
XP_016913409 LKKKVIKNG--KNGTRPNRSDMCTLKIIGKLK-DN--TIVEKYEDLKIQLGDVELIQGLD
XP_011053696 LMKKVIKKG--TKDVKPIQKDICTLKFTGVLD-DG--TVVEDQNNISIQLGDFEIVQGLD
XP_019934661 LRKKVLEPA-APLVPCPTWGQEVAIKMQCVLEDR---TVVEKDRKLVFVIGEGDVNQALE
XP_008304064 LRKKVLERG-TLQAPHPTWGQEVAVKMQCVLEDR---TVVEKDCKLVFVIGEGDVNQALE
XP_011053695 LMKKVIKKG--TKDVKPIQKDICTLKFTGVLD-DG--TVVEDQNNISIQLGDFEIVQGLD
CAX74525 VVIKTLRKG-LGRETRPSHGDTVVINYKGWLEDG---TLVDDVENAKIVLGDGDVIHAFD
XP_023811597 LRKKVLKSG-DVQIPHPTWGQEVTVMMQGVLEDR---TVVEKDCKLVFVIGEGDVNQALE
EFZ15418 LKKKVIQKG--KENVKPHKGDICTIKMSGVLD-DN--TVVEDYDNLTIQIGDLEVVQGLD
OPJ70235 LRKRVVRDG-QD-EPRPRPGQEVSVKVLGVLDDG---GLVERDPRLTFVPGHGDVVQALE
AAX27902 VVIKTLRKG-LGRETRPSHGDTVVINYKGWLEDG---TLVDDVENAKIVLGDGDVIHAFD
XP_011053694 LMKKVIKKG--TKDVKPIQKDICTLKFTGVLD-DG--TVVEDQNNISIQLGDFEIVQGLD
XP_011164040 LKKKVIQKG--KENVKPHKGDICTIKMSGVLD-DN--TVVEDYDNLTIQIGDLEVVQGLD
XP_011150605 LKKKVILKG--KNGTRPNRGDICTLKIIGKLK-NNQ-KIVENYEDFVIQLGDVEVVQGLD
EFN77170 LKKKVILKG--KNGTRPNRGDICTLKIIGKLK-NNQ-KIVENYEDFVIQLGDVEVVQGLD
XP_011164039 LKKKVIQKG--KENVKPHKGDICTIKMSGVLD-DN--TVVEDYDNLTIQIGDLEVVQGLD
AAH90698 LKKKVLQAG-PENALTPAWGQEVTLKMQGVLEDR---TVVEKDSKLVFIIGEGDVTQALE
XP_014606393 LRKKVIKEG--QNGTRPNTTDYCTLKIIGKLE-AG--KIVEEHENITIQLGDVEVVQGLD
XP_003700328 LRKKVIRKG--KNGTRPNTSDICTLKIIGKLQ-DG--TIVEEYEELKIQLGDVEVIQGLD
XP_683608 LKKKVLQAG-PENALTPAWGQEVTLKMQGVLEDR---TVVEKDSKLVFIIGEGDVTQALE
XP_014606392 LRKKVIKEG--QNGTRPNTTDYCTLKIIGKLE-AG--KIVEEHENITIQLGDVEVVQGLD
XP_023726255 LKKKVLKRA--ENGYRPQRSHICEINLQGSLP-DGT--IIDKHDHLSIQIGDAEVVQGLD
XP_011404052 LLKKILKPG-DGVSSRPSPLSEVTMRVLGSLPNG---TVIDKHNSLRFIVSDGDVVQAVD
XP_018337850 LMKKVIKKG--TQDVKPIQKDICTLKFTGVLD-DG--TVVEDQDNVSIQLGDFEVVQGLD
XP_023726263 LKKKVLKRA--ENGYRPQRSHICEINLQGSLP-DGT--IIDKHDHLSIQIGDAEVVQGLD
XP_012689022 LRKKVQEPG-PPEAQGPAWGQQVTLKMQGVLEDR---MVVEKDTKLVFVIGEGDVNQALE
XP_015190182 LRKKVIKEG--QSGTRPNTTDYCTLKIIGKLE-SG--KIVEEHENITIQLGDVEVVQGLD
XP_024943519 LRRKIIKLG--EKGNRPNASDICTLKIIGKLE-DG--TVVEEYEELTIQLGDIEVIQGLD
XP_015437909 LKKRVIRKG--KHGTRPTRSDICTLKIVGKLT-DG--TVVEEYENLKIQLGDVEVIQGLD
KYN13005 LMKKIIKKG--TKDVKPIQKDVCTLTFTGVLD-DG--IVVEDQDNVSIQLGDFEVVQGLD
XP_024943518 LRRKIIKLG--EKGNRPNASDICTLKIIGKLE-DG--TVVEEYEELTIQLGDIEVIQGLD
XP_012526171 LKKKVIQKG--EENVKPHNGDMCTIKMIGKLD-DG--TVVEDYNNLTIQIGDMEVIQGLD
XP_012237396 LKKKVIKSG--KNGTRPNRSDICTLKIIGKLK-DG--TTVEEYEDLKIQLGDVEVIQGLD
XP_018372055 LMKKIIKKG--TKDVKPIQKDVCTLTFTGVLD-DG--IVVEDQDNVSIQLGDFEVVQGLD
XP_015600846 LRRKIIKLG--EKGNRPNASDICTLKIIGKLE-DG--TVVEEYEELTIQLGDIEVIQGLD
XP_018372054 LMKKIIKKG--TKDVKPIQKDVCTLTFTGVLD-DG--IVVEDQDNVSIQLGDFEVVQGLD
XP_023811598 LRKKVLKSG-DVQIPHPTWGQEVTVMMQGVLEDR---TVVEKDCKLVFVIGEGDVNQALE
XP_013086934 LIKKILTAG-YGIDTRPTNGDIVTLKVEGRREDG---KLVD-NEEITFYLNDGDVILAFD
XP_012340112 LKKKVIKNG--KNGTRPNRSDICTLKIIGKLK-DN--TIVEKYEDLKIQLGDVELIQGLD
XP_012064601 LMKKVIKKG--TKDVKPIQKDICTLKFTGMLD-DG--TVVEDQDNISIQLGDFEVIQGLD
XP_021933338 LKKKILKRA--ESSYRPQRSHICEINLEGRLP-DGT--IVDKHDHLSIQIGDAEVVQGLD
XP_020469449 LRKNVLKPG-DLNALCPTWGQEVTVKMQCVLEDR---TVVEKDSKLVFVIGEGDVNQALE
XP_012340111 LKKKVIKNG--KNGTRPNRSDICTLKIIGKLK-DN--TIVEKYEDLKIQLGDVELIQGLD
XP_014772499 LTKSIVKKG-DGVLTRPLQGQKVTLKTEGLLPDG---TVIDKNESISFILGDGDVISGWD
XP_021933337 LKKKILKRA--ESSYRPQRSHICEINLEGRLP-DGT--IVDKHDHLSIQIGDAEVVQGLD
XP_002425448 LKLKTIKQG--EPDSRPQANDICEIRLLGKLN-DG--KIVENFDSLKIELGSHEVVQGVE
XP_018053112 LMKKVIKKG--TKDVKPIQKDICTLKFTGVLD-DG--TVVEDQDNISIQLGDFEVIQGLD
XP_023811595 LRKKVLKSG-DVQIPHPTWGQEVTVMMQGVLEDR---TVVEKDCKLVFVIGEGDVNQALE
XP_003692338 LKKKVIKNG--KNGTRPNRSDICTLKIIGKLK-DN--TIVEKYEDLKIQLGDVELIQGLD
KQK73886 LRKRVVRDG-QD-GLRPRPGQEVSVKVLGALEDG---GLVERDPRLTFVPGYGDVVQALE
XP_021933339 LKKKILKRA--ESSYRPQRSHICEINLEGRLP-DGT--IVDKHDHLSIQIGDAEVVQGLD
XP_018025482 LKKKVLVAG--IVETRPVRGDTVCIELTMRLS-DG--TLVEDTHTLYFTLGDSEVIPGLD
XP_019335449 LRKRVVQQG-RGESTRPCPGQEVTVKLLGVLEDG---SLVEKDPALRFTLGQGDVVQALE
XP_014772498 LTKSIVKKG-DGVLTRPLQGQKVTLKTEGLLPDG---TVIDKNESISFILGDGDVISGWD

```
XP_023811594       LRKKVLKSG-DVQIPHPTWGQEVTVMMQGVLEDR---TVVEKDCKLVFVIGEGDVNQALE
XP_001846585       LLKKVLQEG--TDE-RPESRDIAVINFTGRLE-DG--TIVEQEQGMVVQIDDVEVVQGLD
XP_014470017       LKKKVLLAG--DKGIRPNRSDTCTLKIVGRLK-DND-TIVENYDDLVIQLGDVEVIQGLD
XP_002111046       LKKATLVAG-SG--VAPKKGQLVTIKYSMHLS-NA--IVVLHDMESQFILGEGEVIPALD
XP_014772494       LTKSIVKKG-DGVLTRPLQGQKVTLKTEGLLPDG---TVIDKNESISFILGDGDVISGWD
KZS20178           LKKKTIKKG--TPNARPVTSEICTIKISGELA-DG--TFVDVKDKFSFQLGDLEVIQGID
XP_014470016       LKKKVLLAG--DKGIRPNRSDTCTLKIVGRLK-DND-TIVENYDDLVIQLGDVEVIQGLD
KFM75734           FKKKVIKKG-LGHESRPSRGDRVTVDVSYYLNE----KLFEENKDLKFIVGDLDVIQGLD
XP_018900271       LKKKVTVPG--EPDTRPQFSEICILDYEGRLD-DGT--VIEKEENHRIQLGDLEVIHGLD
XP_020392992       LKKKILKYG-NVKLERPGKGQNVKIWVKIMHENGD---VILGRQRLSFTINGTDVVKALE
XP_016376789       IRKKVLQAS-PENALTPAWGQEVTLKMQGVLEDR---TVVEKDCKLVFVIGEGDVNQALE
XP_016345079       LRKKVLQAS-PENVLTPAWGQEVTLKMQGVLEDR---TVVEKDCKLVFIIGEGDVNQALE
XP_014106477       LRKRVVRAG-PG-GPRPLPGQEVSVKVLGALEDG---GLVERDPRLIFVPGHGDVVLALE
XP_014237957       LLKKVVKAG--IKNTRPNRSDLCTIDIIGRLE-DG--TIVEEHEDLVVQLGDMETIQGID
XP_016376788       IRKKVLQAS-PENALTPAWGQEVTLKMQGVLEDR---TVVEKDCKLVFVIGEGDVNQALE
XP_016376787       IRKKVLQAS-PENALTPAWGQEVTLKMQGVLEDR---TVVEKDCKLVFVIGEGDVNQALE
CDS28193           VLIKTINKG-LGQGSRPSHGDSVVINYKCYLEDG---TLVEELDNLNIVIGDGDCTHAID
KOC65132           LKKKVLKKG--KNGTRPNRSDICTLNIIGKLK-DD--TTVENYEDLKIQLGDVEVIQGLD
XP_017790334       LKKKVLKKG--KNGTRPNRSDICTLNIIGKLK-DD--TTVENYEDLKIQLGDVEVIQGLD
XP_020790728       LRKKILEPG-CP--PCPSWGEEVTVRMQAVLEDR---TVVEKDTKLVFVIGEGDVNQALE
XP_012221110       LKKKVIKEG--EDGTRPNKRDICKLKVVCEVN-G---EIVEEKDDFMIQLGDMEVVQGLD
GAA30660           -------KG-LGRESRPNHGDLVEVSFKGYLEDG---TIVDDVASMVCTLGDGDVIQALD
XP_012221101       LKKKVIKEG--EDGTRPNKRDICKLKVVCEVN-G---EIVEEKDDFMIQLGDMEVVQGLD
XP_012221093       LKKKVIKEG--EDGTRPNKRDICKLKVVCEVN-G---EIVEEKDDFMIQLGDMEVVQGLD
XP_018649440       ----TLKKG-LGGETRPSHGDSVVVNYKCWLEDG---TLVDDVEDVKMVLGDGDIIHAFD
EFX68474           LKKKTVKKG--IPNTRPTTNEICTIKISGQLA-DG--KAVDVQEKFSFQLGDLEVIQGID
XP_023229640       LKKRVIESG-VGLESRPQRGDWIVIDATGELEDG---TVVEKYDNMTIIVGDSDIVQGLD
EAT32833           LLKKVLKEG--QGE-RPESRDIAIVNFTGRLD-DG--TVVEEEQNCVVQIDDVEVVQGLD
XP_016344167       IRKKVLQAS-PENALTPAWGQEVTLKMQGVLEDR---TVVEKDCKLVFVIGEGDVNQALE
XP_016344168       IRKKVLQAS-PENALTPAWGQEVTLKMQGVLEDR---TVVEKDCKLVFVIGEGDVNQALE
XP_016344166       IRKKVLQAS-PENALTPAWGQEVTLKMQGVLEDR---TVVEKDCKLVFVIGEGDVNQALE
XP_016376234       LRKKVLQAS-PENVLTPAWGQEVTLKMQGVLEDR---TVVEKDCKLVFIIGEGDVNQALE
XP_007906811       LKKITLKSG-NAQLGKPQPSQNVSIWIKMMHENRK---EVEDPQYISFIIGEGDVNEALE
KYN04145           LMKKVIKKG--KKDVKPIRQDICTIKFTGMLD-DG--TVVEDYDDFSIQLGDFDVIQGLD
XP_014282311       LMKKVLTPG--IPNSRPQKSDICVLNIVGCLE-DGS--IVEKHENLEINHSDNEVIQGLD
XP_015109871       LKKKTLIKG--KKNTRPQRNDICTINIVGKLE-NGK-E-VECFIDVAIQLGDVELIQGLD
CDS42647           VEKKVLKKG-LGQGSRPGHGDSVVISYKGWLEDG---MLVEEVENLSLVIGDGECIHAFD
XP_011870270       LKKRVIRKG--EKGMRPEKGDICTINFVGKLC-DG--TEVEKEDHLSIQVGDLEVIQGLD
XP_011870269       LKKRVIRKG--EKGMRPEKGDICTINFVGKLC-DG--TEVEKEDHLSIQVGDLEVIQGLD
XP_018977514       LRKKVLQAS-PENVLTPAWGQEVTLKMQGVLEDR---TVVEKDCKLVFIIGEGDVNQALE
XP_007906810       LKKITLKSG-NAQLGKPQPSQNVSIWIKMMHENRK---EVEDPQYISFIIGEGDVNEALE
CDS17605           VEKKVLKKG-LGQGSRPGHGDSVVISYKGWLEDG---MLVEEVENLSLVIGDGECIHAFD
XP_014206998       LRKKVVKPG--KDNTRPNRLDLCMLSFTVKLE-DG--TIVDEAEDLIVQVGDVEYTQGLD
XP_018393954       LMKKVIKKG--KKDVKPIRQDICTIKFTGMLD-DG--TVVEDYDDFSIQLGDFDVIQGLD
XP_015929100       FKKLVIKKG-NGHETRPEKGDRVTVRLSCFVND----KLVEEQENLQVIVGDMDVIQGLD
ELU06040           LKKKVLTKG-DGE--RPVNGQMVTIKCAGHLPDG---KAVDQHDNLRFTLGDGDVIQAFD
XP_024353326       VEKKVLKKG-LGQGSRPGHGDSVVISYKGWLEDG---MLVEEVENLSLVIGDGECIHAFD
EAT35561           LLKKVLKEG--QGE-RPESRDIAIVNFTGRLD-DG--TVVEEEQNCVVQIDDVEVVQGLD
XP_018359770       LMKKVIKKG--TKDVKPIQKDICTLTFTGVLD-DG--TVVEDQDNVSIQLGDFEVVQGLD
EAT35560           LLKKVLKEG--QGE-RPESRDIAIVNFTGRLD-DG--TVVEEEQNCVVQIDDVEVVQGLD
XP_018319727       ILKLTLVEG--KLGTRPISLQNCKIKYKCYLEDG---VLVEEQDDFEVQIGDNEVVQGLD
XP_001662371       LLKKVLKEG--QGE-RPESRDIAIVNFTGRLD-DG--TVVEEEQNCVVQIDDVEVVQGLD
XP_018319726       ILKLTLVEG--KLGTRPISLQNCKIKYKCYLEDG---VLVEEQDDFEVQIGDNEVVQGLD
XP_001662370       LLKKVLKEG--QGE-RPESRDIAIVNFTGRLD-DG--TVVEEEQNCVVQIDDVEVVQGLD
XP_012269548       LKKKVVKAG--QPGTRPNRSDICTIKLTGKLE-DG--KVVEDHEKLTVQLGDVEVIQGLD
PAA92621           LWKKTLVEG-RGRDSRPQSGDQVTLKLEASLPDG---TPVESESGLIISIGEYDHIQALD
XP_019867812       IMKKIIHEG--IPDTKPQRLQTCCINYTCTLEDG---TVIEEVDGMEIFVGDSDVIQGLD
PAA53270           LWKKTLVEG-RGRDSRPQSGDQVTLKLEASLPDG---TPVESESGLIISIGEYDHIQALD
```

PAA57938	LWKKTLVEG-RGRDSRPQSGDQVTLKLEASLPDG---TPVESESGLIISIGEYDHIQALD
XP_966821	LLKKIVKEG--QANTRPQRLQKCTISYELSLADG---TFIERKDNEEIQLGDCDVVQGLD
KXJ84517	LLKKVLKEG--QGE-RPESKDIAVINYTGRLD-DG--TIVEEERDCVVQIDDVEVVQGLD
XP_023345477	LRKKILIEG-TGD--KPDKSIRVRIHLKEFLYEDG---DCIKDEELEMNVGEFELVQALD
XP_011494478	LKKKVLKAG--KQNSRPNRSDLCSVKFIGKLE-DG--TIIENEEEITIQLGDMEVIQGLD
XP_023345475	LRKKILIEG-TGD--KPDKSIRVRIHLKEFLYEDG---DCIKDEELEMNVGEFELVQALD
XP_016940923	LIKRTIKKAPENNDRRPLRGELVTLNLIGKLD-NG--KVVEEEKNFQCHVGDFEVVQGLD
XP_023345476	LRKKILIEG-TGD--KPDKSIRVRIHLKEFLYEDG---DCIKDEELEMNVGEFELVQALD
PNF41144	LKKKVLKRA--ENGYRPQRSHICEINLQGSLP-DGT--IIDKHDHLSIQIGDAEVVQGLD
XP_019932587	LLKKVLKEG--QGE-RPESKDIAIINYTGRLD-DG--TIVEEERDCVVQIDDVEVVQGLD
XP_019932586	LLKKVLKEG--QGE-RPESKDIAIINYTGRLD-DG--TIVEEERDCVVQIDDVEVVQGLD
XP_016028426	LIKRTIKKAPQDSFRRPVRGELVTVNFTGKLD-NG--TVVENELNFQCHVGDYEVVQGLD
XP_016958803	LIKRTIKKAPEDGGRRPLRGELVTLNLIGKLD-NG--KVVEEEKNFQCHVGDFEVVQGLD
EKC18274	LKRKILRAA-AKNAKRPISSDLVTIKVEGKLEDG---TKVDVYDSLSFVLGDGDVIQAWD
PNF41145	LKKKVLKRA--ENGYRPQRSHICEINLQGSLP-DGT--IIDKHDHLSIQIGDAEVVQGLD
XP_011636531	IKKKVLKKG--KDDTRPNRGDICTINIVGTLD-DG--TVAERYNDLTIQIGDMEVVQGLD
XP_017014991	LIKRTIRKAPEDGGRRPLRGELVTLNLTGKLD-NG--QVVEEEKNFQCHVGDFEVVQGLD
XP_001974711	LIKRTIKKAPQDSVRRPIRGELVTVNFTGKLD-NG--TVVEKELNFQCHVGDYEVVQGLD
XP_024874265	LKKRVVKKG--EQNVKPQRGDICTVTIMGVLD-SG--KVVEEHVDKQIQTGDMEVVQGLD
NP_611353	LIKRTIKKAPQDSFRRPIRGELVTVNFTGKLD-NG--TVVENELNFQCHVGDYEVIQGLD
XP_016984960	LIKRTIKKAPEGGDRRPLRGELVTLNFTGKLD-NG--KVVEEEKNFQCHVGDYEVVQGLD
XP_017054378	LIKRTIKKPPVDGGRRPLRGELVTLNFTGKLD-NG--KVVDDEKNFQCHVGDYEVVQGLD
XP_024874264	LKKRVVKKG--EQNVKPQRGDICTVTIMGVLD-SG--KVVEEHVDKQIQTGDMEVVQGLD
XP_019545364	LLKKVLKEG--QGE-RPESKDIAIINYTGRLD-DG--TIVEEERDCVVQIDDVEVVQGLD
XP_019545363	LLKKVLKEG--QGE-RPESKDIAIINYTGRLD-DG--TIVEEERDCVVQIDDVEVVQGLD
XP_014670901	------------METRPERGSLVKLSYSGKLDDG---TEVDTCECKEFVLGDGDMIHGLE
XP_017079421	LIKRTIKKPPQEEARRPVRGELVTLNLTGKLD-NG--KVVEDEKNFQCHVGDYEVIQGLD
XP_013113222	LIKRVLKKS--TSNKRPQRGDKCTLSFTGKLE-NG--KIVEKQENFEIHVGDFEVIQGLD
XP_002034480	LIKRTITKAPQDSFRRPVRGELVTVNFTGALD-NG--TVVENELNFQCHVGDYEVVQGLD
XP_017031858	LIKRIIKKAPKGADQ-PSRGFLVTINFTGKLD-NG--KVVEEEKGFQCHIGDFEVVQGLD
XP_015788695	ILKKVIKEG-NKEKARPEKGCRVKINLSVFLKDSD--EKVESLDGIEIFVGDCDLNHAVD
XP_017117238	LIKRTIKKAPGDVDRRPLRGELVTLNFTGKLD-NG--KVVEEEKNFQCHVGDYEVVQGLD
XP_015364528	LLKKIIKQG--APDERPMKGEQIVINLVGRLE-END-DIFEKEENFEITLGDCEVIQGVD
XP_017836078	LIKRIIKKSPANAKR-PSRGDLVKVRFTAKLD-DG--TVVEQEDNFECHVGDLEIVQGLD
XP_316645	LLKKVLKKG--RSELRPESKDLVTVSYTGRLE-DG--TVVEEQSNAVVQIDDVEVVQGLD
XP_002091890	LIKRTIKKAPQDSLRRPIRGELVTVNFTGKLD-NG--TVVEKEINFQCHVGDYEVVQGLD
XP_009022869	IKKRVIMKG-DEKACRPRNGQQIQLKCSGKLDDG---TVVDVHEKLTLALGEDEHINAFD
XP_020815089	LIKRIIKKAPKGANQ-PSRGFLVTINFTGKLD-NG--KVVEEEKGFQCHIGDFEVVQGLD
KYO18601	LRKRVVQQG-RGESTRPCPGQEVTVKLLGVLEDG---SLVEKDPALRFTLGQGDVVQALE
XP_023307145	LIKRVIKKS--KVEKKPQRGELVTISFTGKLD-DG--TVVEKEENYQVHVGDYEVVQGLD
XP_004526322	LVKRVLKPS--QLDRRPMRGDLATITYSGKLP-NG--IVVEQKENYQVHVGDFEVVQGLD
XP_017468465	LVKRVLKQS--TTGKRPTRGDLVTVSFTGKLP-DG--TVVEQKKNFQMHVGDFEVVQGLD
XP_011213061	LVKRVIKQS--QLSTRPARGDLVTVTFKGRMP-NG--IVVEEKENYQIHVGDYEVVQGLD
ETN59059	LLKKVLRKG--LTEMRPESKDIATISYTGRLE-DG--TIVEEEHEAVVQIDDVEVVQGLD
XP_019877780	IMKKIIREG--LPDTKPQRLQKCRINYTCTLEDDP--EVILQEEGNEIFLGDCDVIQGLD
XP_001951673	LLKKIIKQG--ALDERPMKGEQIVINLVGRLE-DND-DIFEKEENFEITLGDCEVIQGVD
XP_002062976	LIKRTIKKASSTSNRRPTRGDLVTINFSGKLD-NG--SLVEEEKNFQCHVGDYEIVQGLD
XP_001986816	LLKRIIKKASSSAKR-PNRGDLVTITYTGKLE-NG--TIVEKEERFQCHVGDLEVVQGLD
XP_022181773	LLKKIIKQG--ALDERPMKGEQIVINLVGRLE-DND-DIFEKEDNFEITLGDCEVIQGVD
XP_002048800	LIKRIIKKAPANAKR-PSRGDLVTITYTGKLE-DG--TVVEKEERFQCHVGDFEVVQGID
XP_018801327	LVKRIIKQS--QLSTRPARGDLVTVTFKGRIP-NG--IVVEKKENYQIHVGDYEVVQGLD
XP_020300622	LRKRVIREG--KPSTRPERGDLCVLNITGQLE-SD--KIVDVHKNKVVQLGDFEVIMGLD
XP_011193416	LVKRVIKQS--QTSNRPARGDLVTVTFTGRLA-NG--IVVEKMENYQMHVGDFEVVQGLD
XP_017960399	LIKRIIKKAPANAKR-PNRGDLVTITYTGKLE-DG--TVVEKEERFQCHVGDFEVVQGLD
ACO13176	LKKRILSEG-TPDT-KPEPGDEVKVVISG-THLGV---EVIPEETLTFRLAEFEAPKALD
XP_011350211	LMKKIIKEG--KEWTKPVRGDICTVNITGRFE-NSQ-ITVEEHTNLEIIVGDGDIVQGLD
KRT81327	IMKKVIQAG--TPDSRPQRLQICEINYESKLESG---ILVEKCNNFIMQLGDCDVIQGLD
XP_023162735	LIKRIIKKAPANAKR-PNRGDLVTITYTGKLE-DG--TVVEKEERFQCHVGDFEVVQGLD

```
XP_019771996      IMKKVLKEG--QPDSRPSRSSRCVINYTCKLEEAHEDDFADVADNYELGLGEGDVVQGLD
XP_005176045      LVKRVLKKS--KVNKRPQRGDKVTLNFTGKLD-DG--RVVEKQENFQVHVGDFEVIQGLD
XP_014095636      LVKRVIKQS--QLIARPARGDLVTVTFNGRMP-NG--IVVEKKENYQIHVGDYEVVQGLD
XP_017867395      LIKRIIKKAPANAKR-PNRGDLVTITYTGKLE-DG--TVVEKEERFQCHVGDFEVVQGLD
XP_002004550      LIKRIIKKAPANAKR-PNRGDLVTITYTGKLE-DG--TVVEKEERFQCHVGDFEVVQGLD
XP_022921247      ILKRVIKKG--ENDLKPQKYHVCKINYVCKLENG---LEVEREENFLMQVDGCDVVQGLD
XP_002015767      LIKRIIKKAPEDAKS-PSRGELVTISFTGKLE-DG--KVVEEEKNFQCHVGDYEVAQGLD
XP_001360319      LIKRIIKKAPEDAKS-PSRGELVTISFTGKLE-DG--KVVEEEKNFQCHVGDYEVAQGLD
XP_017147297      LIKRIIKKAPEDAKS-PSRGELVTISFTGKLE-DG--KVVEEEKNFQCHVGDYEVAQGLD
XP_019879021      LMKRILIGG--QPDTKPLALQLCTINYSISLEPE---TELNRAIDQTITLGDCDILHGID
                                                                             .

NP_036313         LSVPLMDVGETAMVTADSKYCYGPQGS-----RSPY---IPPHAALCLEVTLKTAV
NP_001295302      LSVPLMDVGETAMVTADSKYCYGPQG------RSPY---IPPHAALCLEVTLKTAV
XP_003817801      LSVPLMDVGETAMVTADSKYCYGPQG------RSPY---IPPHAALCLEVTLKTAV
XP_010384957      LSVPLMDVGETAMVTADSKYCYGPQG------RSPY---IPPHAALCLEVTLKTAV
XP_003817802      LSVPLMDVGETAMVTADSKYCYGPQG------RSPY---IPPHAALCLEVTLKTAV
XP_011747960      LSVPLMDVGETAMVTADSKYCYGPQG------RSPY---IPPHAALCLEVTLKTAV
XP_003915233      LSVPLMDVGETAMVTADSKYCYGPQG------RSPY---IPPHAALCLEVTLKTAV
EHH59371          LSVPLMDVGETAMVTADSKYCYGPQG------RSPY---IPPHAALCLEVTLKTAV
XP_005588521      LSVPLMDVGETAMVTADSKYCYGPQG------RSPY---IPPHAALCLEVTLKTAV
XP_002828980      LSVPLMDVGETAMVTADSKYCYGPQG------RSPY---IPPHAALCLEVTLKTAV
PNJ28395          LSVPLMDVGETAMVTADSKYCYGPQGS-----RSPY---IPPHAALCLEVTLKTAV
XP_011949838      LSVPLMDVGETAMVTADSKYCYGPQG------RSPY---IPPHAALCLEVTLKTAV
XP_018870801      LSVPLMDVGETAMVTADSKYCYGPQG------RSPY---IPPHAALCLEVTLKTAV
XP_007993999      LSVPLMDVGETAMVTADSKYCYGPQG------RSPY---IPPHAALCLEVTLKTAV
XP_014978797      LSVPLMDVGETAMVTADSKYCYGPQG------RSPY---IPPHAALCLEVTLKTAV
XP_011747796      LSVPLMDVGETAMVTADSKYCYGPQG------RSPY---IPPHAALCLEVTLKTAV
XP_003915232      LSVPLMDVGETAMVTADSKYCYGPQG------RSPY---IPPHAALCLEVTLKTAV
XP_002828981      LSVPLMDVGETAMVTADSKYCYGPQG------RSPY---IPPHAALCLEVTLKTAV
XP_010328455      LSVPLMDVGETAMVTADSKYCYGPQG------RSPY---IPPHAALCLEVTLKTAV
XP_008969279      LSVPLMDVGETAMVTADSKYCYGPQGS-----RSPY---IPPHAALCLEVTLKTAV
XP_011845848      LSVPLMDVGETAMVTADSKYCYGPQG------RSPY---IPPHAALCLEVTLKTAV
XP_024207707      LSVPLMDVGETAMVTADSKYCYGPQGS-----RSPY---IPPHAALCLEVTLKTAV
XP_011526166      LSVPLMDVGETAMVTADSKYCYGPQGS-----RSPY---IPPHAALCLEVTLKTAV
XP_011949835      LSVPLMDVGETAMVTADSKYCYGPQG------RSPY---IPPHAALCLEVTLKTAV
XP_017356796      LSVPLMDVGETAMVTADSKYCYGPQG------RSPY---IPPHAALCLEVTLKTAV
XP_008969278      LSVPLMDVGETAMVTADSKYCYGPQGS-----RSPY---IPPHAALCLEVTLKTAV
NP_001247999      LSVPLMDVGETAMVTADSKYCYGPQGS-----RSPY---IPPHAALCLEVTLKTAV
XP_024307207      LSVPLMDVGETAMVTADSKYCYGPQGS-----RSPY---IPPHAALCLEVTLKTAV
XP_016791013      LSVPLMDVGETAMVTADSKYCYGPQGS-----RSPY---IPPHAALCLEVTLKTAV
XP_009192156      LSVPLMDVGETAMVTADSKYCYGPQGS-----RSPY---IPPHAALCLEVTLKTAV
XP_024307210      LSVPLMDVGETAMVTADSKYCYGPQGS-----RSPY---IPPHAALCLEVTLKTAV
XP_005588520      LSVPLMDVGETAMVTADSKYCYGPQGS-----RSPY---IPPHAALCLEVTLKTAV
XP_011747874      LSVPLMDVGETAMVTADSKYCYGPQGS-----RSPY---IPPHAALCLEVTLKTAV
XP_024092631      LSVPLMDVGETAMVTADSKYCYGPQGS-----RSPY---IPPHAALCLEVTLKTAV
XP_004060362      LSVPLMDVGETAMVTADSKYCYGPQGS-----RSPY---IPPHAALCLEVTLKTAV
XP_011949836      LSVPLMDVGETAMVTADSKYCYGPQGS-----RSPY---IPPHAALCLEVTLKTAV
XP_007993998      LSVPLMDVGETAMVTADSKYCYGPQGS-----RSPY---IPPHAALCLEVTLKTAV
XP_014978796      LSVPLMDVGETAMVTADSKYCYGPQGS-----RSPY---IPPHAALCLEVTLKTAV
XP_009251263      LSVPLMDVGETAMVTADSKYCYGPQGS-----RSPY---IPPHAALCLEVTLKTAV
XP_011747747      LSVPLMDVGETAMVTADSKYCYGPQGS-----RSPY---IPPHAALCLEVTLKTAV
XP_017822960      LSVPLMDVGETAMVTADSKYCYGPQG------RSPY---IPPHAALCLEVTLKTAV
XP_009192157      LSVPLMDVGETAMVTADSKYCYGPQGS-----RSPY---IPPHAALCLEVTLKTAV
XP_010328454      LSVPLMDVGETAMVTADSKYCYGPQGS-----RSPY---IPPHAALCLEVTLKTAV
XP_011845855      LSVPLMDVGETAMVTADSKYCYGPQGS-----RSPY---IPPHAALCLEVTLKTAV
XP_012299012      LSVPLMDVGETAMVTADSKYCYGPQG------RSPY---IPPHAALCLEVTLKTAV
XP_011949833      LSVPLMDVGETAMVTADSKYCYGPQGS-----RSPY---IPPHAALCLEVTLKTAV
```

```
XP_017356795      LSVPLMDVGETAMVTADSKYCYGPQGS-----RSPY---IPPHAALCLEVTLKTAV
XP_014401247      LSVPLMDVGETAMVTADSKYCYGPQG------RSPY---IPPHAALCLEVTLKTAV
EPQ12447          LSVPLMDVGETAMVTADSKYCYGPQG------RSPY---IPPHAALCLEVTLKTAV
XP_008154612      LSVPLMDVGETAMVTADSKYCYGPQG------RSPY---IPPHAALCLEVTLKTAV
XP_016066232      LSIPLMDVGETAMVTADSKYCYGPQG------RSPY---IPPHAALCLEVTLKTAV
XP_012584673      LSVPLMDVGETAMVTADSKYCYGPQG------RSPY---IPPHAALCLEVTLKTAV
XP_015413209      LSVPLMDVGETAMVTADSKYCYGPQG------RSPY---IPPHAALCLEVTLKTAV
XP_022262557      LSVPLMDVGETAMVTADSKYCYGPQG------RSPY---IPPHAALCLEVTLKTAV
XP_023103313      LSVPLMDVGETAMVTADSKYCYGPQG------RSPY---IPPHAALCLEVTLKTAV
XP_019271925      LSVPLMDVGETAMVTADSKYCYGPQG------RSPY---IPPHAALCLEVTLKTAV
XP_007088417      LSVPLMDVGETAMVTADSKYCYGPQG------RSPY---IPPHAALCLEVTLKTAV
XP_002761955      LSVPLMDVGETAMVTADSKYCYGPQGS-----RSPY---IPPHAALCLEVTLKTAV
XP_014921066      LSVPLMDVGETAMVTADSKYCYGPQG------RSPY---IPPHAALCLEVTLKTAV
XP_005972346      LSVPLMDVGETAMVTADSKYCYGPQG------RSPY---IPPHAALCLEVTLKTAV
XP_006058025      LSVPLMDVGETAMVTADSKYCYGPQG------RSPY---IPPHAALCLEVTLKTAV
XP_017905457      LSVPLMDVGETAMVTADSKYCYGPQG------RSPY---IPPHAALCLEVTLKTAV
XP_019271923      LSVPLMDVGETAMVTADSKYCYGPQG------RSPY---IPPHAALCLEVTLKTAV
XP_008542456      LSVPLMDVGETAMVTADSKYCYGPQG------RSPY---IPPHAALCLEVTLKMAV
XP_006186302      LSVPLMDVGETAMVTADSKYCYGPQG------RSPY---IPPHAALCLEVTLKTAV
XP_006199114      LSVPLMDVGETAMVTADSKYCYGPQG------RSPY---IPPHAALCLEVTLKTAV
XP_010970466      LSVPLMDVGETAMVTADSKYCYGPQG------RSPY---IPPHAALCLEVTLKTAV
XP_024416679      LSVPLMDVGETAMVTADSKYCYGPQG------RSPY---IPPHAALCLEVTLKTAV
XP_005652984      LSVPLMDVGETAMVTADSKYCYGPQG------RSPY---IPPHAALCLEVTLKAAV
XP_014687059      LSVPLMDVGETAMVTADSKYCYGPQG------RSPY---IPPHAALCLEVTLKMAV
XP_015344756      LSVPLMDVGETAMVTADSKYCYGPQG------RSPY---IPPHAALCLEVTLKTAV
XP_012299010      LSVPLMDVGETAMVTADSKYCYGPQGS-----RSPY---IPPHAALCLEVTLKTAV
XP_019819388      LSVPLMDVGETAMVTADSKYCYGPQG------RSPY---IPPHAALCLEVTLKTAV
ELW67147          LSVPLMDVGETAMVTADSKYCYGPQGS-----RSPY---IPPNAALCLEVTLKTAV
XP_005873908      LSVPLMDVGETAMVTADSKYCYGPQGS-----RSPY---IPPHAALCLEVTLKTAV
XP_016066229      LSIPLMDVGETAMVTADSKYCYGPQGS-----RSPY---IPPHAALCLEVTLKTAV
XP_008154611      LSVPLMDVGETAMVTADSKYCYGPQGS-----RSPY---IPPHAALCLEVTLKTAV
XP_020740155      LSVPLMDVGETAMVTADSKYCYGPQG------RSPY---IPPHAALCLEVTLKTAV
XP_003982130      LSVPLMDVGETAMVTADSKYCYGPQGS-----RSPY---IPPHAALCLEVTLKTAV
XP_019271924      LSVPLMDVGETAMVTADSKYCYGPQGS-----RSPY---IPPHAALCLEVTLKTAV
XP_007088416      LSVPLMDVGETAMVTADSKYCYGPQGS-----RSPY---IPPHAALCLEVTLKTAV
XP_541935         LSVPLMDVGETAMVTADSKYCYGPQGS-----RSPY---IPPHAALCLEVTLKTAV
XP_012419293      LSVPLMDVGETAMVTADSKYCYGPQGS-----RSPY---IPPHAALCLEVTLKTAV
XP_004688664      LSVPLMDVGETAMVTADSKYCYGPQGS-----RSPY---IPPHAALCLEVTLKTAV
XP_015413208      LSVPLMDVGETAMVTADSKYCYGPQGS-----RSPY---IPPHAALCLEVTLKTAV
XP_006747423      LSVPLMDVGETAMVTADSKYCYGPQGS-----RSPY---IPPHAALCLEVTLKTAV
XP_017905455      LSVPLMDVGETAMVTADSKYCYGPQGS-----RSPY---IPPHAALCLEVTLKTAV
XP_010993437      LSVPLMDVGETAMVTADSKYCYGPQGS-----RSPY---IPPHAALCLEVTLKTAV
XP_006058026      LSVPLMDVGETAMVTADSKYCYGPQGS-----RSPY---IPPHAALCLEVTLKTAV
XP_014921065      LSVPLMDVGETAMVTADSKYCYGPQGS-----RSPY---IPPHAALCLEVTLKTAV
XP_006058024      LSVPLMDVGETAMVTADSKYCYGPQGS-----RSPY---IPPHAALCLEVTLKTAV
XP_001503379      LSVPLMDVGETAMVTADSKYCYGPQGS-----RSPY---IPPHAALCLEVTLKMAV
XP_005972345      LSVPLMDVGETAMVTADSKYCYGPQGS-----RSPY---IPPHAALCLEVTLKTAV
XP_019271926      LSVPLMDVGETAMVTADSKYCYGPQGS-----RSPY---IPPHAALCLEVTLKTAV
XP_006186301      LSVPLMDVGETAMVTADSKYCYGPQGS-----RSPY---IPPHAALCLEVTLKTAV
XP_006199113      LSVPLMDVGETAMVTADSKYCYGPQGS-----RSPY---IPPHAALCLEVTLKTAV
XP_010970465      LSVPLMDVGETAMVTADSKYCYGPQGS-----RSPY---IPPHAALCLEVTLKTAV
XP_003123564      LSVPLMDVGETAMVTADSKYCYGPQGS-----RSPY---IPPHAALCLEVTLKAAV
XP_024416678      LSVPLMDVGETAMVTADSKYCYGPQGS-----RSPY---IPPHAALCLEVTLKTAV
NP_001192650      LSVPLMDVGETAMVTADSKYCYGPQGS-----RSPY---IPPHAALCLEVTLKTAV
XP_020740157      LSVPLMDVGETAMVTADSKYCYGPQGS-----RSPY---IPPHAALCLEVTLKTAV
XP_015344757      LSVPLMDVGETAMVTADSKYCYGPQGS-----RSPY---IPPHAALCLEVTLKTAV
XP_006147177      LSVPLMDVGETAMVTADSKYCYGPQGS-----RSPY---IPPNAALCLEVTLKTAV
XP_020740156      LSVPLMDVGETAMVTADSKYCYGPQGS-----RSPY---IPPHAALCLEVTLKTAV
```

XP_014687058	LSVPLMDVGETAMVTADSKYCYGPQGS-----RSPY---IPPHAALCLEVTLKMAV
XP_004404706	LSVPLMDVGETAMVTADSKYCYGPQGS-----RSPY---IPPHAALCLEVTLKTAV
XP_019819387	LSVPLMDVGETAMVTADSKYCYGPQGS-----RSPY---IPPHAALCLEVTLKTAV
XP_020008573	LSVPLMDVGETAMVTADSKYCYGPQGS-----RSPY---IPPHAALCLEVTLKTAV
XP_007116995	LSVPLMDVGETAMVTADSKYCYGPQG------RSPY---IPPHAALCLEVTLKTAV
XP_024622992	LSVPLMDVGETAMVTADSKYCYGPQG------RSPY---IPPHAALCLEVTLKTAV
XP_012391225	LSVPLMDVGETAMVTADSKYCYGPQG------RSPY---IPPHAALCLEVTLKTAV
XP_020740154	LSVPLMDVGETAMVTADSKYCYGPQGS-----RSPY---IPPHAALCLEVTLKTAV
XP_022414296	LSVPLMDVGETAMVTADSKYCYGPQG------RSPY---IPPHAALCLEVTLKTAV
XP_021587090	LSVPLMDVGETAMVTADSKYCYGPQGS-----RSPY---IPPHAALCLEVTLKTAV
XP_019592316	LSVPLMDVGETAMVTADSKYCYGPQG------RSPY---IPPHAALCLEVTLKMAV
XP_023989754	LSVPLMDVGETAMVTADSKYCYGPQGS-----RSPY---IPPHAALCLEVTLKTAV
XP_024622991	LSVPLMDVGETAMVTADSKYCYGPQGS-----RSPY---IPPHAALCLEVTLKTAV
XP_004277581	LSVPLMDVGETAMVTADSKYCYGPQGS-----RSPY---IPPHAALCLEVTLKTAV
XP_017651479	LSVPLMDVGETAMVTADSKYCYGPQG------RSPY---IPPHAALCLEVTLKTAE
XP_022414295	LSVPLMDVGETAMVTADSKYCYGPQGS-----RSPY---IPPHAALCLEVTLKTAV
XP_017651477	LSVPLMDVGETAMVTADSKYCYGPQG------RSPY---IPPHAALCLEVTLKTAE
XP_007461122	LSVPLMDVGETAMVTADSKYCYGPQGS-----RSPY---IPPHAALCLEVTLKTAV
XP_007195521	LSVPLMDVGETAMVTADSKYCYGPQGS-----RSPY---IPPHAALCLEVTLKAAV
XP_019592317	LSVPLMDVGETAMVTADSKYCYGPQGS-----RSPY---IPPHAALCLEVTLKMAV
XP_005001666	LSVPLMDVGETAMVTADSKYCYGPQG------RSPY---IPPHAALCLEVTLKTAV
XP_008822765	LSVPLMDVGETAMVTADSKYCYGPQGS-----RSPY---IPPHAALCLEVTLKTAE
XP_017651474	LSVPLMDVGETAMVTADSKYCYGPQGS-----RSPY---IPPHAALCLEVTLKTAE
XP_013002970	LSVPLMDVGETAMVTADSKYCYGPQGS-----RSPY---IPPHAALCLEVTLKTAV
XP_012924198	LSAPLMDVGETAMVTADSKYCYGPQG------RSPY---IPPHAALCLEVTLKTAV
EHB02679	LSAPLMDVGETAMVTADSKYCYGPQGS-----RSPY---IPPHAALCLEVTLKTAV
XP_012924197	LSAPLMDVGETAMVTADSKYCYGPQGS-----RSPY---IPPHAALCLEVTLKTAV
XP_021535026	LSVPLMDVGETAMVTADSKYCYGPQGS-----RSPY---IPPHAALCLEVTLKTAV
XP_007943554	LSVPLMDVGETAMVTADSKYCYGPQG------RSPY---IPPNAALCLEVTLKTAV
XP_011220551	LSVPLMDVGETAMVTADSKYCYGPQG------RSPY---IPPHAALCLEVTLKTAV
XP_008588853	LSVPLMDVGETAMVTADSKYCYGPQGS-----RSPY---IPPHAALCLEVTLKTAV
XP_012916132	LSVPLMDVGETAMVTADSKYCYGPQG------RSPY---IPPHAALCLEVTLKTAV
XP_022353384	LSVPLMDVGETAMVTADSKYCYGPQG------RSPY---IPPHAALCLEVTLKTAV
XP_008048663	LSVPLMDVGETAMVTADSKYCYGPQG------RSPY---IPPHAALCLEVTLKMAV
XP_019652974	LSVPLMDVGETAMVTADSKYCYGPQGS-----RSPY---IPPHAALCLEVTLKTAV
XP_008702161	LSVPLMDVGETAMVTADSKYCYGPQGS-----RSPY---IPPHAALCLEVTLKTAV
XP_019497623	LSVPLMDVGETAMVTADSKYCYGPQG------RSPY---IPPHAALCLEVTLKTAV
XP_007943555	LSVPLMDVGETAMVTADSKYCYGPQGS-----RSPY---IPPNAALCLEVTLKTAV
XP_002912777	LSVPLMDVGETAMVTADSKYCYGPQGS-----RSPY---IPPHAALCLEVTLKTAV
EFB19124	LSVPLMDVGETAMVTADSKYCYGPQGS-----RSPY---IPPHAALCLEVTLKTAV
XP_013362739	LSVPLMDVGETAMVTADSKYCYGPQG------RSPY---IPPHAALCLEVTLKTAV
XP_006876371	LSVPLMDVGETAMVTADSKYCYGPQG------RSPY---IPPHAALCLEVTLKTAV
XP_021493693	LSVPLMDVGETAMVTADSKYCYGPQG------RSPY---IPPHAALCLEVTLKTAE
XP_022353383	LSVPLMDVGETAMVTADSKYCYGPQGS-----RSPY---IPPHAALCLEVTLKTAV
NP_001272500	LSVPLMDVGETAMVTADSKYCYGPQG------RSPY---IPPHATLCLEVTLKTAV
XP_012916133	LSVPLMDVGETAMVTADSKYCYGPQGS-----RSPY---IPPHAALCLEVTLKTAV
XP_011365085	LSVPLMEVGETAMVTADSKYCYGPQG------RSPY---IPPHAALCLEVTLKTAV
XP_006885938	LSVALMDVGETAMVTADSKYCYGPQG------RSPY---IPPNAALCLEVTLKTAV
XP_004423006	LSVPLMDVGETAMVTADSKYCYGPQG------RSPY---IPPHAALCLEVILKTAV
XP_012413338	LSVPLMDVGETAMVTADSKYCYGPQG------RSPY---IPPHAALCLEVTLKTAV
XP_010591524	LSVPLMDVGETAMVTADSKYCYGPQG------RSPY---IPPHAALCLEVTLKTAV
XP_008048662	LSVPLMDVGETAMVTADSKYCYGPQGS-----RSPY---IPPHAALCLEVTLKMAV
XP_015987916	LSVPLMEVGETAMVTADSKYCYGSQG------RSPY---IPPHAALCLEVTLKTAV
XP_004666075	LSVPLMDVGETAMVTADSKYCYGPQGS-------PY---IPPHAALCLEVTLKTAV
XP_019497624	LSVPLMDVGETAMVTADSKYCYGPQGS-----RSPY---IPPHAALCLEVTLKTAV
XP_004649071	LSVPLMDVGETAMVTADSKYCYGPQGS-----RSPY---IPPHAALCLEVTLKTAV
APD32922	LSVPLMDVGETAMVTADSKYCYGPQGS-----RSPY---IPPHAALCLEVTLKTAV
XP_013362738	LSVPLMDVGETAMVTADSKYCYGPQGS-----RSPY---IPPHAALCLEVTLKTAV

```
XP_012509480      LSVPLMDVGETAMVTADSKYCYGPQGS-----RSPY---IPPHAALCLEVTLKTAV
NP_034353         LSVPLMDVGETAMVTADSKYCYGPQG------RSPY---IPPHAALCLEVTLKTAE
XP_021074830      LSVPLMDVGETAMVTADSKYCYGPQG------RSPY---IPPHAALCLEVTLKTAE
XP_006876370      LSVPLMDVGETAMVTADSKYCYGPQGS-----RSPY---IPPHAALCLEVTLKTAV
XP_021493691      LSVPLMDVGETAMVTADSKYCYGPQGS-----RSPY---IPPHAALCLEVTLKTAE
XP_006904374      LSVPLMEVGETAMVTADSKYCYGPQGS-----RSPY---IPPHAALCLEVTLKTAV
XP_006989222      LSVPLMDVGETAMVTADSKYCYGPQG------RSPY---IPPHAALCLEVTLKTAE
XP_004423005      LSVPLMDVGETAMVTADSKYCYGPQGS-----RSPY---IPPHAALCLEVILKTAV
XP_006885939      LSVALMDVGETAMVTADSKYCYGPQGS-----RSPY---IPPNAALCLEVTLKTAV
XP_004666073      LSVPLMDVGETAMVTADSKYCYGPQGS-----RSPY---IPPHAALCLEVTLKTAV
ELK19193          LSVPLMEVGETAMVTADSKYCYGPQGS-----RSPY---IPPHAALCLEVTLKTAV
XP_003499206      LSVPLMDVGETAMVTADSKYCYGSQG------RSPY---IPPHAALCLEVTLKTAE
XP_014637207      LSVPLMDVGETAMVTADSKYCYGPQGS-----RSPY---IPPHAALCLEVILKTAV
XP_012790031      LSVPLMDVGETAMVTADSKYCYGPQG------RSPY---IPPHAALCLEVTLKAAV
XP_003413066      LSVPLMDVGETAMVTADSKYCYGPQGS-----RSPY---IPPHAALCLEVTLKTAV
XP_004384434      LSVPLMDVGETAMVTADSKYCYGPQGS-----RSPY---IPPHAALCLEVTLKTAV
XP_015987917      LSVPLMEVGETAMVTADSKYCYGSQGS-----RSPY---IPPHAALCLEVTLKTAV
XP_021078721      LSVPLMDVGETAMVTADSKYCYGPQGS-----RSPY---IPPHAALCLEVTLKTAE
5MGX_E            LSVPLMDVGETAMVTADSKYCYGPQG------RSPY---IPPHAALCLEVTLKTAV
XP_017168055      LSVPLMDVGETAMVTADSKYCYGPQG------RSPY---IPPHAALCLEVTLKTAE
NP_001032257      LSVPLMHVGETAMVTADSKYCYGPQGS-----RSPY---IPPHAALCLEVTLKTAE
XP_004595597      LSVPLMDVGETAMVTADSKYCYGPQG------RSPY---IPPHAALCLEVTLKTAV
XP_017529992      LSVPLMEVGETAMVTADSKYCYGPQG------RSPY---IPPHAALCLEVTLKTAV
XP_005370624      LSVPLMDVGETAMVTADSKYCYGPQG------RSPY---IPPHAALCLEVTLKMAE
NP_001104536      LSVPLMDVGETAMVTADSKYCYGPQGS-----RSPY---IPPHAALCLEVTLKTAE
XP_021074828      LSVPLMDVGETAMVTADSKYCYGPQGS-----RSPY---IPPHAALCLEVTLKTAE
XP_012664880      LSVPLMDVGETAMVTADSKYCYGPQG------WSPY---IPPHAALCLEVSLKTAV
XP_006989221      LSVPLMDVGETAMVTADSKYCYGPQGS-----RSPY---IPPHAALCLEVTLKTAE
XP_007635977      LSVPLMDVGETAMVTADSKYCYGSQGS-----RSPY---IPPHAALCLEVTLKTAE
XP_004616706      LSVPLMDVGETAMVTADSKYCYGPQGS-----RSPY---IPPHAALCLEVTLKAAV
XP_012664879      LSVPLMDVGETAMVTADSKYCYGPQGC-----RSPY---IPPHAALCLEVSLKTAV
XP_004705311      LSVPLMEVGETAMVTADSKYCYGPQG------RSPY---IPPHAALCLEVTLQTAE
XP_017168054      LSVPLMDVGETAMVTADSKYCYGPQGS-----RSPY---IPPHAALCLEVTLKTAE
XP_021026690      LSVPLMDVGETAMVTADSKYCYGPQG------RSPY---IPPHAALCLEVTLKTAE
XP_004595596      LSVPLMDVGETAMVTADSKYCYGPQGS-----RSPY---IPPHAALCLEVTLKTAV
XP_017529990      LSVPLMEVGETAMVTADSKYCYGPQGS-----RSPY---IPPHAALCLEVTLKTAV
XP_005370622      LSVPLMDVGETAMVTADSKYCYGPQGS-----RSPY---IPPHAALCLEVTLKMAE
XP_012883833      LSVPLMDVGETAMVTADSKYCYGPQG------RSPY---IPPHAALCLEVTLKSAV
XP_021026691      LSVPLMDVGETAMVTADSKYCYGPQGS-----RSPY---IPPHAALCLEVTLKTAE
XP_010849455      FSVPLMDVGETAMVTADSKYCYGPQGS-----RSPY---IPPHAALCLEVTLKTAV
XP_010604777      LSVPLMDVGETAMVTADAKYCYGPQGX-----XXXY---IPPHAALCLEVTLKTAV
XP_004705310      LSVPLMEVGETAMVTADSKYCYGPQGS-----RSPY---IPPHAALCLEVTLQTAE
XP_021026689      LSVPLMDVGETAMVTADSKYCYGPQGS-----RSPY---IPPHAALCLEVTLKTAE
XP_005898381      LSVPLMDVGETAMVTADSKYCYGPQGS-----RSPY---IPPHAALCLEVTLKTAV
XP_012883832      LSVPLMDVGETAMVTADSKYCYGPQGS-----RSPY---IPPHAALCLEVTLKSAV
XP_024622994      LSVPLMDVGETAMVTADSKYCYGPQG------RSPY---IPPHAALCLEVTLKTAV
XP_022414294      LSVPLMDVGETAMVTADSKYCYGPQG------RSPY---IPPHAALCLEVTLKTAV
XP_007116996      LSVPLMDVGETAMVTADSKYCYGPQGS-----RSPY---IPPHAALCLEVTLKTAV
XP_024622993      LSVPLMDVGETAMVTADSKYCYGPQGS-----RSPY---IPPHAALCLEVTLKTAV
XP_022414293      LSVPLMDVGETAMVTADSKYCYGPQGS-----RSPY---IPPHAALCLEVTLKTAV
XP_023055022      LSVPLMDVGETAMVTADSKYCYGPQGS-----RSPY---IPPHAALCLEVTLKTAV
ELR54589          LSVPLMDVGETAMVTADSKYCYGPQGS-----RSPY---IPPHAALCLEVTLKTAV
XP_010845609      LSVPLMDVGETAMVTADSKYCYGPQGS-----RSPY---IPPHAALCLEVTLKTAV
XP_021538869      LSVPLMDVGETAMVTADSKYCYGPQGS-----RSPY---IPPHAALCLEVTLKTAV
KFO22602          LSVPLMDVGETAMVTADAKYCYGPQG-------SGD---AP---GLVAVVTLKTAV
2MF9_A            LSVPLMDVGETAMVTADSKYCYGPQGS-----RSPY---IPPHAALCLEVTLKTAV
PNI50452          LSVPLMDVGETAMVTADSKYCYGPQGS-----RSPY---IPPHAALCLEVTLKTA-
PNJ28404          LSVPLMDVGETAMVTADSKYCYGPQGS-----RSPY---IPPHAALCLEVTLKTA-
```

```
XP_007533227      LSIPLMDVGESAMVTADSKYCYGPQGS-----RSPC---VPPDAALCLEVTLLAAA
XP_020864537      LSVQLMNVGETALIIADAKYCYGPHG------RSPT---IPPNSTLRLEVALQTAA
XP_012397649      LSVQLMNVGETALIIADAKYCYGSCG------RSPA---IPPNSTLRLEVALQSAV
XP_001369403      LSVQLMNVGETALIIADAKYCFGSHG------RSPT---IPPNSTLRLEVALQTAV
2F2D_A            LSVPLMDVGETAMVTADSKYCYGPQG------RSPY---IPPHAALCLEVTLKTAV
2D9F_A            LSVPLMDVGETAMVTADSKYCYGPQGS-----RSPY---IPPHAALCLEVTLKTAV
XP_015668943      LCVQLMDMGETALITSDGKYCFGPQG------RSPD---IPPNAFLTLEVELLAAQ
2AWG_A            LSVPLMDVGETAMVTADSKYCYGPQG------RSPY---IPPHAALCLEVTLKTAV
XP_015668942      LCVQLMDMGETALITSDGKYCFGPQGS-----RSPD---IPPNAFLTLEVELLAAQ
XP_025032464      LCVQLMDMGETALITSDGKYCFGPQG------RSPD---IPPDASLTLEVELLAAQ
XP_007443923      LCVQLMDMGETALITSDGKYCFGPQG------RSPD---IPPDASLTLEVELLAAQ
KFV73372          LCVQLLEMGETALIMSDAKYCYGPQG------RSPD---IPPNAALTLEVELLEAR
OXB56359          LCVQLMEMGETALIMSDAKYCYGAQG------RSPD---VPPNAALTLEVELLEAR
XP_009675861      LCVQLLEMGETALIMSDAKYCYGPQG------RSPD---IPPNAALTLEVELLEAR
OXB73131          LCVQLMEMGETALIMSDAKYCYGAQG------RSPD---VPPNAALTLEVELLEAR
XP_010723402      LCVQLMEMGETALIMSDAKYCYGVQG------RSPD---IPPNAALTLEVELLEAQ
KFV67807          LCVQLLEMGETALIQSDAKYCYGLQG------RSPD---IPPNAALTLEVELLAAR
XP_010304001      LCVQLLEMGETALIVSDAKYCYGSQG------RSPD---IPPDAALTLEVELLAAQ
NP_001241668      LCVQLMEMGETALIMSDAKYCYGAQG------RSPD---IPPNAALTLEVELLEAR
KFO03599          LCVQLLEMGETALIVSDAKYCYGSQG------RSPD---IPPDAALTLEVELLAAQ
XP_021234495      LCVQLMEMGETALIMSDAKYCYGAQG------RSPD---IPPNAALTLEVELLEAQ
POI29828          LCVQLMEKGETALIMSDAKYCYGAQG------RSPD---IPPNAALTLEVELLEAQ
XP_021234496      LCVQLMEMGETALIMSDAKYCYGAQG------RSPD---IPPNAALTLEVELLEAQ
XP_023963943      LCVQLMEMGETASILSDAKYCYGPQG------RSPD---IPPHAALTLEVQLLAVQ
XP_014793985      LCVQLLEMGETALIVSDAKYCYGAQG------RSPD---IPPNAALTLEVELLKAQ
XP_009899401      LCVQLLEMGETALIQSDAKYCYGLQG------RSPD---IPPNAALTLEVELLAAR
XP_015742210      LCVQLMEMRETALITSDAKYCYGAQG------RSPD---IPPNAALTLEVELLEAR
XP_009641057      LCVQLLEMGETALIVSDAKYCYGAQG------RSPD---IPPNAALTLEVELLSAR
KFP19261          LCVQLLEMGETALIVSDAKYCYGAQG------RSPD---IPPNAALTLEVELLSAR
XP_024065593      LCVQLMEMGETASIMSDAKYCYGPQG------RSPD---IPPHAALTLEVQLLAVQ
XP_011594878      LCVQLLEMGETALIVADAKYCYGAQG------RSPD---IPPNAALTLEVELLAAQ
XP_014742726      LCVQLLEMGETALIVSDAKYCYGAQG------RSPD---IPPNASLTLEVELLAAR
XP_015742208      LCVQLMEMRETALITSDAKYCYGAQG------RSPD---IPPNAALTLEVELLEAR
XP_019369180      LCTQLLETGETASIMSDAKYCYGPQG------RSPD---IPPNAALALEVELLAVQ
XP_010561556      LCVQLLEMGETALIVADAKYCYGAQG------RSPD---IPPNAALTLEVELLAAQ
XP_008638175      LCVQLLEMGETALIVSEAKYCYGAQG------RSPD---IPPNASLTLEVELLAAR
XP_002195923      LCVQLLEMGETALIMSEAKYCYGAQG------RSPD---IPPNAALTLEVELLAAR
XP_015266773      LCVQLMEMGETALIQSDPKYCHGSQG------RSSD---IPPDAALTLEVELLAAR
XP_005505599      LCVQLLEMGETALIVSDAKYCYGAQG------RSPD---IPPNATLTLEVELLAAQ
XP_014115778      LCVQLLEMGETALVVSEAKYCYGAQG------RSPD---IPPNASLTLEVELLAAR
XP_014742725      LCVQLLEMGETALIVSDAKYCYGAQG------RSPD---IPPNASLTLEVELLAAR
XP_018780521      LCVQLLEMGETALIVSDAKYCYGAQG------RSPD---VPPNASLSLEVELLAAR
XP_005235043      LCVQLLEMGETALIVSDAKYCYGAQG------RSPD---IPPNAALTLEVELLAAR
OPJ86694          LCVQLLEIGETALIVSDAKYCYGAQG------RSPD---IPPNATLTLEVELLAAQ
XP_019341692      LCTQLLEMGETALITSDAKYCYGPQG------RSPG---IPPNTALTLEVELLAVQ
XP_021400798      LCVQLLEMGETALIVSEAKYCYGAQG------RSPD---IPPNASLRLEVELLAAR
XP_006028906      LCTQLLEMGETASITSDAKYCYGPQG------RSPG---IPPNAALTLEVELLAVQ
KFQ97904          LCVQLLEMGETALIVSDAKYCYGAQG------RSPD---VPPNAALTLEVELLAAR
XP_015507158      LCVQLLEMGETALIVSEAKYCYGAQG------RSPD---IPPNASLTLEVELLAAR
KFW12711          LCVQLLEMGETALIVSDAKYCYGVQG------RSPD---VPPNASLTLEVELLEAR
XP_006271082      LCTQLLEMGETALITSDAKYCYGPQG------RSPG---IPPNTALTLEVELLAVQ
KFO81655          LCVQLLEMGETALIVSDAKYCYGTQG------RSPD---IPPNAALTLEVELLAAQ
XP_007072098      LCAQLLEMGETALIMSDAKYCFGPQG------RSPD---IPPHAALTLEVQLLAVQ
XP_020669929      LCVQLMEMGETAMILSDAKYCYGPQG------RSPD---VPPDASLAFEVELLSAR
XP_009464539      LCVQLLEMGETALIVSDAKYCYGAQG------RSPD---VPPNAALTLEVELLAAR
XP_010158039      LCVQLLEMGETALIVSDAKYCYGVQG------RSPD---VPPNASLTLEVELLEAR
XP_019149030      LCVQLLEMGETALIVSEAKYCYGAQG------RSPD---IPPNASLTLEVELLAAR
XP_020669930      LCVQLMEMGETAMILSDAKYCYGPQG------RSPD---VPPDASLAFEVELLSAR
```

```
XP_019393900       LCTQLLETGEMASIVSDAKYCYGPQG------RSPD---IPPNAALTLEVELLAVQ
EMP24085           LCAQLLEMGETALIMSDAKYCFGPQG------RSPD---IPPHAALTLEVQLLAVQ
XP_009566597       LCVQLLEMGETALIVSDAKYCYGTQG------RSPD---IPPNAALTLEVELLAAQ
KFW65305           LCVQLLEMGETALIMSDAKYCYGAQG------RRPD---VPPNAALALEVELLGAR
XP_015507157       LCVQLLEMGETALIVSEAKYCYGAQG------RSPD---IPPNASLTLEVELLAAR
XP_013799130       LCVQLLEMGETALIVSDAKYCYGSQG------RSPD---IPPNAALTLEVELLEAR
XP_014433658       LCAQLLEMGETALIVSDAKYCYGPQG------RTPD---IPPHATLTLEVELLAAQ
XP_009319696       LCVQLLEMGETALIMSDAKYCYGAQG------RRPD---VPPNAALALEVELLGAR
KFQ36923           LCVQLLEMGETALIVSDAKYCYGAQG------RSPD---VPPNAALTLEVELLAAQ
XP_005060257       LCVQLLEMGETALIVSDAKYCYGAQG------RSPD---IPPNASLTLEVELLAAQ
KFU87694           LCVQLLEMGETALIMSDAKYCYGAQG------RSPD---VPPNAALTLEVELLAAQ
XP_005139681       LCVQLLEMGETALIVSDAKYCYGAQG------RSPD---IPPNAALTLEVELLEAR
XP_009993397       LCVQLLEMGETALIMSDAKYCYGAQG------RSPD---VPPNAALTLEVELLAAQ
AAH84415           LCVQLMEVGETSLIASDAKYCYGKEG------RSPD---IPPNSNLNLEVTLLDVQ
KQL50954           LCVQLLEVGETALIVSDAKYCYGAQG------RSPD---IPPNAALTLEVELLEAR
NP_001088346       LCVQLMEVGETSLIASDAKYCYGKEG------RSPD---IPPNSNLNLEVTLLDVQ
XP_013913386       LCVQLMDMGETALITSDGNYCFGPLG------RIPD---IPPCASLTLEVELLTAQ
KFQ13009           LCVQLLEMGETALIVSDAKYCYGVQG------RSPD---VPPNAAITLEVELLVAR
KFQ75436           LCVQLLEMGETALIVSDAKYCYGTQG------RSPD---VPPNAALTLEVELLAAR
KFP56346           LCVQLLEMGETALIVSDAKYCYGSQG------RSPD---VPPNAALTLEVELLAAR
KFM07869           LCVQLLEMGETALIMSDAKYCYGAQG------RSPD---VPPNAALTLEVELLGAR
XP_018096952       LCVQLMEEGETSLIASDAKYCYGKEG------RSPD---IPPNSNLNLEVTLLVVQ
KFP65829           LCVQLLEMRETALIVSDAKYCYGAQG------RSPD---IPPNAALTLEVELLAAR
XP_023803625       LCVQLLEMGETALIVSEAKYCYGAQG------RSPD---IPPNASLTLEVELLAAR
XP_009951725       LCVQLLEMGETALIVSDAKYCYGVQG------RSPD---VPPNAAITLEVELLVAR
NP_001120570       LCVQLMEAEETSLIASDAKYCYGKEG------RSPD---IPPNSNLNLEVALLDVQ
KFP23912           LCVQLLEMGETALIVSDAKYCYGVQG------RSPD---VPPNAALTLEVELLAAR
XP_010073720       LCVQLLELGETALIVSDAKYCYGAQG------RSPD---IPPNAALTLEVELLAAR
KGL86304           LCVQLLEMGETALIVSDAKYCYGAQG------RSPD---VPPNAALTLEVELLAAR
KFV08597           LCVQLLELGETALIVSDAKYCYGAQG------RSPD---IPPNAALTLEVELLAAR
KFW76752           LCVQLLEMGETALIVSDAKYCYGAQG------RSPD---IPPNASLTLEVELLAAR
XP_009700019       LCVQLLEMRETALIVSDAKYCYGAQG------RSPD---IPPNAALTLEVELLAAR
XP_010208130       LCVQLLEMGETALIVSDAKYCYGVQG------RSPD---VPPNAALTLEVELLAAR
KFQ81502           LCVQLLEMGETALIVSDAKYCYGAQG------RSPD---IPPNAALTLEVELLAAQ
XP_017938939       LCVQLLEMGETALIVSDAKYCYGAQG------RSPD---IPPNASLTLEVELLAAR
KFP70760           LCVQLLEMKETALIVSDAKYCYGAQG------RSPD---IPPNTSLTLEVELLAAR
XP_009880959       LCVQLLEMGETALIVSDAKYCYGAQG------RSPD---VPPNAALTLEVELLAAR
XP_010292010       LCVQLLEMGETALIVSDAKYCYGAQG------RSPD---IPPNAALTLEVELLAAQ
XP_009076312       LCVQLLEMKETALIVSDAKYCYGAQG------RSPD---IPPNTSLTLEVELLAAR
XP_018412986       LCVQLMELGETALIEADAKYCYGKQG------RDPD---IPPNADLLLEVSLLEVK
XP_022525503       LTVQLMEIGEKALVEAGSKYAYGALGS-----STPA---VPPHADLILEIQLLSAA
XP_021459512       LTVQLMEMGETALVQADAKYAYGALGS-----LAPE---VLPSADLALEVQLLDAT
XP_021459510       LTVQLMEMGETALVQADAKYAYGALGS-----LAPE---VLPSADLALEVQLLDAT
XP_024277334       LTVQLMEMGETALVQADAKYAYGALGS-----LAPE---VLPSADLALEVQLLDAT
XP_024277330       LTVQLMEMGETALVQADAKYAYGALGS-----LAPE---VLPSADLALEVQLLDAT
XP_024277333       LTVQLMEMGETALVQADAKYAYGALGS-----LAPE---VLPSADLALEVQLLDAT
XP_009512288       LCVQLLEMGETALIVSDAKYCYGAQG------RSPD---IPPNASLTLEVELLAAQ
XP_020354512       LTVQLMEMGETALVQADAKYAYGALGS-----LAPE---VLPSADLALEVQLLDAT
KFZ50883           LCVQLLEMGETALIVSDAKYCYGVQG------RSPD---IPPNAALTLEVELLVAK
KFW81721           LCVQLLEMGETALIVSDAKYCYGAQG------RSPD---IPPNASLTLEVELLAAQ
CDQ57258           LTVQLMEMGETALVQADAKYAYGALGS-----LAPE---VLPSADLALEVQLLDAT
XP_020354513       LTVQLMEMGETALVQADAKYAYGALGS-----LAPE---VLPSADLALEVQLLDAT
XP_020487811       LSVQLMEKGEKALIQTDAKYAYGARGS-----YEPE---IPPNAPLCLEVELKEAT
XP_024277331       LTVQLMEMGETALVQADAKYAYGALGS-----LAPE---VLPSADLALEVQLLDAT
XP_014071869       LTVQLMEMGEKALVQADAKYAYGALGS-----LAPE---VLPSADLALEVQLLDAT
XP_011472153       LTVQLMEMGEKALIQSDPKYAYGDRGS-----LEPR---VPPNAQLSLEVELLEAT
XP_014071870       LTVQLMEMGEKALVQADAKYAYGALGS-----LAPE---VLPSADLALEVQLLDAT
XP_024114895       LTVQLMEMGEKAEIQADAKYAYGDRGS-----LEPA---VPPNATLSLEVELLEAT
```

XP_024114896	LTVQLMEMGEKAEIQADAKYAYGDRGS-----LEPA---VPPNATLSLEVELLEAT
ETE64390	LCVQLMDMGETALITSDGKYCFGPQG------RYMR---KWTEAGLLARVELLTAQ
XP_023809890	LTVQLMEMGEKALIQSDPKYAYGDRGS-----LEPR---VPPNAQLSLEVELLEAT
XP_008499209	LCVQLLEMGETALIVSDPKYCYGAQG------RSPD---IPPNATLTLEVELLGAR
KFP06384	LCVQLLEMGETALIVSDPKYCYGAQG------RSPD---IPPNATLTLEVELLGAR
XP_023859426	LIVQLMEMGEKALVQADAKYAYGALGS-----LAPE---VLPNADLALEVQLLDAT
XP_023859424	LIVQLMEMGEKALVQADAKYAYGALGS-----LAPE---VLPNADLALEVQLLDAT
XP_023859423	LIVQLMEMGEKALVQADAKYAYGALGS-----LAPE---VLPNADLALEVQLLDAT
XP_021454666	LTVQLMEMREKALVQANAKYAYGALGS-----LAPD---VPPNAALALEVQLLDAT
XP_017572299	LTVQLMEMGEKALVEAGAKYAYGALGS-----STPA---VPPDADLIMEVQLLSAA
CDQ97962	LTVQLMEMREKALVQANAKYAYGALGS-----LAPD---VPPNAALALEVQLLDAT
XP_023854736	LTVQLMEMREKALVQANAKYAYGSLGS-----LVPE---VPPNAELALEVQLLDAT
XP_018920592	LTVQMMEMGEKALIEAGAKYAYGALGS-----STPA---VPPDADLMLEVQLLSAD
NP_001133417	LTVQLMEMREKALVQANAKYAYGALGS-----LAPE---VPPNAELALEVQLLDAT
XP_023854735	LTVQLMEMREKALVQANAKYAYGSLGS-----LVPE---VPPNAELALEVQLLDAT
XP_024258320	LTVQLMEMREKALVQANAKYAYGALGS-----LAPD---VPPNAELALEVQLLDAT
AAI52651	LTVQLMEMGEKALIEAAAKYAYGALGS-----SAPA---VPPDADLLLEVQLLSAD
XP_020348060	LTVQLMEMREKALVQANAKYAYGALGS-----LVPD---VPPNAELALEVQLLDAT
NP_957178	LTVQLMEMGEKALIEAAAKYAYGALGS-----SAPA---VPPDADLLLEVQLLSAD
XP_005932512	LTVQLMAMGEEALIQTDAKYAYGARGS-----LEPA---VPANAELSLEVKLLEAT
XP_013860095	LTVQLMEIEEKALIHTDAKYAYGVRGS-----LDPE---IPPNASLSLEVELLEAT
PWA20923	LTVQLMEMGEKALIHTDAKYAYGARGS-----LEPE---VPPNAQLSLEVELVKAT
XP_005727139	LTVQLMALGEEALIQTDAKYAYGARGS-----LEPA---VPANAELSLEVKLLEAT
XP_006790401	LTVQLMAMGEEALIQTDAKYAYGARGS-----LEPA---VPANAELSLEVKLLEAT
XP_019207270	LTVQLMAMGEEALIQTDAKYAYGARGS-----LEPA---VPANAELSLEVKLLEAT
XP_024654530	LTVQLMALGEEALIQTDAKYAYGARGS-----LEPA---VPANAELSLEVKLLEAT
XP_007897075	LCVQLMELDETAQIISDSKYAYGTRGS-----TQPA---IPPSARLHMEVQLVDVE
XP_010870009	LSVQLMEMGEKALVQADAKYAYGTLES-----LSPP---VPPSADLSLEVQLLDAT
XP_022615368	LTVQLMEMGEKALIQTDAKYAYGTRGS-----LEPE---VPPNTELSLEVDLLEAT
XP_010870008	LSVQLMEMGEKALVQADAKYAYGTLES-----LSPP---VPPSADLSLEVQLLDAT
XP_005795669	LTVQLMEMGEKALIHTDAKYAYGARGS-----LEPE---VPPNAQLSLEVELVKAT
XP_020391166	LCVQLMDQNEVALIVSDAKYAYGSLGS-----SEPS---VPSNATVHLEVQLLSVG
AWP11099	LTVQLMEMGEKALIQTDAKYAYGTRGS-----LDPE---VPPDAELSLEVELLGAT
XP_017333233	LTVQLMEMGEKALIEASAKYAYGALGSN----STPV---VPPNADLILEVHLLSAV
KTF74275	LTVQLMEMGEKALIEAGAKYTYGALGS-----STPA---VPPDADLILEVQLLSAE
XP_017262938	LTVQLMEMGEKALVQTDAKYAYGARGS-----LDPE---VPPNAALSLEVELLEAT
XP_023263872	LTVQLMEMGEKALIQTDAKYAYGTRGS-----LEPE---VPPNAELSLEVDLLEAT
XP_020391165	LCVQLMDQNEVALIVSDAKYAYGSLGS-----SEPS---VPSNATVHLEVQLLSVG
XP_016327233	LTVQLMEMGEKALIEAGAKYAYGALGS-----STPA---APPDSDLILEVQLLSAD
XP_016138463	LTVQLMEMGEKALIEAGAKYAYGALGS-----STPA---APPDSDLILEVQLLSAD
XP_016356127	LTVQLMEMSEKALIEAGAKYAYGALGS-----STPA---VPPDADLILEVQLLSAE
XP_007577281	LTVQLMEMGEKALIHTDAKYAYGARGS-----LEPE---IPPNAQLSLEVELVGAT
XP_008405333	LTVQLMEMGEKALIHTDAKYAYGVRGS-----LEPD---IPPNAQLSLEVELVGAT
XP_012715520	LTVQLMEMGEKALIHTDAKYAYGARGS-----LDPE---VPPNAQLSLEVELLEAT
XP_012695070	LAVQLMEMGEKALVEADAKYAYGSVGS-----ACPP---VVPNSNLTMEVQLLTAL
XP_020795782	LTVQLMELGERALVLTDAKYAYGSQGS-----FDPK---VPPNAELSLEVELLEVT
XP_016382508	LTVQLMEMSEKALIEAGAKYAYGALGS-----STPA---VPPDADLILEVQLLSAE
XP_018522913	LTVQLMEKGEKALIQTDAKYAYGTRGS-----LEPE---VPPSAELSLEVELLEAT
XP_008305299	LTVQLMEMGEKALIHTDAIYAYSSFGS-----QDPA---IPPDAELSLEVELLEAK
XP_015238114	LTVQLMEMGEKALIHTDAKYAYGARGS-----LEPE---VPPNAQLSLEVELLKAA
XP_018583793	LTVLLMEMDERALVQTDAKYAYGALGS-----TTPA---VPPDASLILELKLLEAV
XP_016886204	LTVQLMEMGEKALIHTDAIYAYSSFGS-----QDPA---IPPDAELSLEVELLEAK
XP_019752339	LAVLLMEMGEKALVQTAAIYAYGTWGS-----CDPD---VPPNAELSLEVELKKVC
XP_010728467	LTVQLMEMGEKALVQTDAKYAYGARGS-----LEPE---VPPNAELSLEVELLEAT
KKF19855	LTVQLMEMGEKALVQTDAKYAYGARGS-----LEPE---VPPNAELSLEVELLEAT
XP_015827013	LTVQLMEIGEKALIQTDAKYAYGVRGS-----LDPD---IPPNAELSLEVELLEAT
XP_022061518	LTVQLMEMREKALIQTDAKYAYGARGS-----LEPE---VSPNAELSLEVELLEAT
XP_019936960	LTVQLMEMGEKALIHTDAKYAYGTRGS-----LEPK---VPPNAELCLEVELLEAT

```
XP_003974097      LTVQLMEMGEKASIQTDAKYAYGELGS-----SEPQ---VPPNTDLHLEIKLLEAT
XP_023699813      LTVLLMEMNEKAIVHTDAKYAYGGLGS-----TAPA---VPPTSPLILELQLLEAT
XP_023699812      LTVLLMEMNEKAIVHTDAKYAYGGLGS-----TAPA---VPPTSPLILELQLLEAT
XP_020478047      LTVQLMEMEEKVLIQTDAKYAFGARGS-----LEPV---VPPNAPLSLEVELLEAT
XP_015220721      LAVQLMELEEKALIYSDAKYAYGTLGNE----SDPA---APPAAALSLEVQLLEAS
XP_023123034      LTVQLMEMREKALIQTDAKYAYGARGS-----LEPE---VSPNAELSLEVELLEAT
XP_020478046      LTVQLMEMEEKVLIQTDAKYAFGARGS-----LEPV---VPPNAPLSLEVELLEAT
XP_020478045      LTVQLMEMEEKVLIQTDAKYAFGARGS-----LEPV---VPPNAPLSLEVELLEAT
XP_008275730      LTVQLMEMKEKALIQTDAKYAYGARGS-----LEPE---VPPNAELSLEVELLEAT
XP_010770232      LTVMLMEMGEKALVQANAKYAYGERGS-----LEPE---VPANEELSLEVELLEAS
XP_002601637      LCVSLMGMGETCEVIVDALYAYGSTGRE------PD---IPENATITYELQLLNAQ
XP_019619553      LCVSLMGMGETCEVIVDALYAYGALGRE------PD---IPENATITYELQLLNAE
XP_006814126      LAVAVMEMGEVCTLLSDSRFTYGDLG------REPD---IPGGAKLRFELELLAVD
XP_006629043      LCAPSMQLDEIALLITDSQYAYGQRG------REPD---IPPGAALLYQLHLLHIR
PVD20167          LTVALMNFGEKAEVTSSSRFAYGKHG------REPD---IPADADVHFTLELLDVQ
XP_023841292      ECAIAMQQDEITLLLADSQYTYGLLG------REPD---IPAWAPLLYQLQLLNIR
XP_008410571      ECVMSMQKGEITLLLADSQYAYGLLG------REPD---IPAWAPLLYQLQLLDIR
XP_008410568      ECVMSMQKGEITLLLADSQYAYGLLG------REPD---IPAWAPLLYQLQLLDIR
XP_022329628      LAVALMEEGQVIELQTDARFAYGEKGRK------PD---IPPNSSISYIIELVKKE
XP_014897626      ECVMSMQKGEITLLLADSQYAYGLLG------REPD---IPAWAPLLYQLQLLDIR
XP_014848641      ECVMSMQKGEITLLLADSQYAYGLLG------REPD---IPAWAPLLYQLQLLDIR
XP_014848639      ECVMSMQKGEITLLLADSQYAYGLLG------REPD---IPAWAPLLYQLQLLDIR
XP_007566466      ECVMSMQKGEITLLLADSQYAYGLLG------REPD---IPAWAPLLYQLQLLDIR
XP_023180976      ECVMSMQKGEITLLVADSQYAYGLLG------REPD---IPAWAPLLYQLQLLDIR
KMQ91381          LAIALMDVGEVAEIVVDPRFAYGKHGK---------RP-IPPDATIIYTVELKTSE
XP_007566465      ECVMSMQKGEITLLLADSQYAYGLLG------REPD---IPAWAPLLYQLQLLDIR
XP_014897625      ECVMSMQKGEITLLLADSQYAYGLLG------REPD---IPAWAPLLYQLQLLDIR
XP_015254615      ECVMSMQKGEITLLLADSQYAYGLLG------REPE---IPAWAPLLYQLQLLDIR
XP_005798327      ECVMSMQKGEITLLVADSQYAYGLLG------REPD---IPAWAPLLYQLQLLDIR
XP_015254612      ECVMSMQKGEITLLLADSQYAYGLLG------REPE---IPAWAPLLYQLQLLDIR
XP_013980328      ECAIAMQQDEITLLLADSQYTYGLLG------REPD---IPAWAPLLYQLQLLNIR
XP_009061784      LGVSLMEIGETCELFAASKFAYGERGRE------PD---IPLNSDITYTLELLKYE
XP_022106769      LCLSLMDLGEVSIIKTSPRFAYGETGRA------PD---VPPNAEVTYELELLNVQ
XP_020625955      LALALTELDEVCELVTDARYAYGKTGKPD---ANPP---IPPDAKLTYEIQLLAVR
XP_020625956      LALALTELDEVCELVTDARYAYGKTGKPD---ANPP---IPPDAKLTYEIQLLAVR
XP_010732628      ECVIAMQQGEITLLLADSQYAYGLLG------REPD---IPAWAPLLYQLQLLDIR
XP_015728921      LGVPTMRTGEVCFFLAASPYGYGPAG------REPD---VPPEAPLLFEVTLLDAR
XP_003440659      ECVMSMQKGEITLLLTDSQYAYGLLG------REPD---IPAWAPLLYQLQLLDFR
XP_014004032      ECAIAMQQDEIALLLADSQYTYGLLG------RETD---IPAWAPLLYQLQLLDIR
XP_010899928      ECVLTMQKDEIVLLLADSQYTYGLLG------REPD---IPAWAPLLYQLQLLEIR
XP_020336380      ECAIAMQQGEIALLLADSQYTYGLLG------RETD---IPAWAPLLYQLQLLDIR
XP_022330049      LAVALMEEGQVIELQTDARFAYGEKGRK------PD---IPPNSSISYIIELVKKE
PIK48936          LTSILMEVGEKAVIIAAPRFAYGEEGRE------PD---VPPNSLVRYELELLEVK
XP_020336379      ECAIAMQQGEIALLLADSQYTYGLLG------RETD---IPAWAPLLYQLQLLDIR
XP_022330048      LAVALMEEGQVIELQTDARFAYGEKGRK------PD---IPPNSSISYIIELVKKE
XP_007907980      LCAYSMELNEIALVISDGKYAYGHLG------RAPD---IRADATVVYEVQLLKVR
XP_013859000      ECVMSMQKGEIALLLADSQYAYQLLG------REPD---IPAWAPLLYQLQLLDVR
XP_019737313      ECVVSMRRGEIALLLADSRYAYGPAG------REPD---IPAGAPLLYQLQLLDVS
XP_021427237      ECAMAMQQDEITLLLADSQYTYGLLG------REPD---IPAWAPLLYQLQLLNIR
XP_023827235      ECAIAMQQDEIALLLADSQYTYGLLG------RETD---IPAWAPLLYQLQLLDIR
XP_023827230      ECAIAMQQDEIALLLADSQYTYGLLG------RETD---IPAWAPLLYQLQLLDIR
XP_008309233      ECVLSMQQGEITLLLADSQYAYGLLG------REPD---VPAWSPLLYQLQLLDVR
XP_024269308      ECAIAMQQDEIALLLADSQYTYGLLG------RETD---IPAWAPLLYQLQLLDIR
XP_002932306      LGVRTMQLGEVAFLLTNSLYAFGLLG------RDPD---IPSDASLLYKVTLLQVR
XP_022785584      IGISLMELDEVCELVTDARYAYGKAGKPD---AKPP---IPPDAKITYEIQLLSVR
XP_008309234      ECVLSMQQGEITLLLADSQYAYGLLG------REPD---VPAWSPLLYQLQLLDVR
XP_013386268      LALSLMERGEVAEIVTDARFAYGSMG------RDPD---IPPNAQLTYEVELVRIE
XP_005928731      ECVMSMQKGEITLLLTDSQYAYGLLG------REPD---IPAWAPLLYQLQLLDFR
```

XP_002119960	MTISLMELGEKSEITSDAKYCYG--DEG----KPPN---IEPGMDLCFTVILLNID
XP_024235293	ECAIAMQQDEITLLLADSQYTYGLLG------REPD---IPAWAPLLYQLQLLNIR
XP_005729996	ECVMSMQKGEITLLLTDSQYAYGLLG------REPD---IPAWAPLLYQLQLLDFR
XP_018619414	HCAASMCLWEIALLLADSQYTYGHQG------RDPD---IPAWSPLLYELQLLDIR
XP_012796312	LSVPLAEHKEIFELTTDSRFAYGSRG------RDSD---IPSGAKLTYRIEVLKVD
XP_004553258	ECVMSMQKGEITLLLTDSQYAYGLLG------REPD---IPAWAPLLYQLQLLDFR
CDQ72337	ECAISMQQDEIALLLADSQYTYGLLG------RETD---IPAWAPLLYQLQLLDIR
XP_024269303	ECAIAMQQDEIALLLADSQYTYGLLG------RETD---IPAWAPLLYQLQLLDIR
XP_024269307	ECAIAMQQDEIALLLADSQYTYGLLG------RETD---IPAWAPLLYQLQLLDIR
XP_024269300	ECAIAMQQDEIALLLADSQYTYGLLG------RETD---IPAWAPLLYQLQLLDIR
XP_017893134	LAIALMDVEEIAEIVVDPRFAYGSLGD---------EPNIPASSTITYTVELKSSE
XP_021454564	ECAISMQQDEIALLLADSQYTYGLLG------RETD---IPAWAPLLYQLQLLDIR
XP_003967095	ECVMSMQMGEVSLLLADSQYAYGLLG------REPD---VPAWAPLLYQLQLLDFR
XP_021454548	ECAISMQQDEIALLLADSQYTYGLLG------RETD---IPAWAPLLYQLQLLDIR
XP_023697743	RCAVSMHPEEISLLLTNSQYAYGRQG------RDPD---VPAWASLLYEVQLLGFR
PIK51124	LAAILMEVGEKAEIIAIPRFAYGEQGRE------PD---VPPNSIVRYELELLEVK
XP_020311464	ECAIAMQQDEITLLLADSQYTYGLLG------REPD---IPAWAPLLYQLQLLNIR
XP_017261735	ECVMSMQKGEIALLLADSQYAYELLG------REPD---IPAWAPLLYQLQLLDVR
XP_011312767	LAVALMDVGEVAQIEISPRFAYGDLGK---------KPDIPPGATLHYNITLKAVE
XP_022047038	ECVMSMQKGEITLLLADSQYAYGLLG------REPD---IPAWAPLLYQLQLLDIR
XP_003427294	LAVALMDLEEEAEVETASRFAYGSLGRKA----TASLPEIPPDAKITYNIVLKSIE
XP_020139363	FVLHMLR-GVPRERDSANPHSWP--GP------------VPPHGYSENKKTV----
XP_011661975	LCVCLMELGEVAEIHTNARFAYGEYGKA------PK---ILPNTDMIYEVELLETN
XP_021263191	LGVPTMRGGEICFFLAASPYGYGRAG------REPD---VPPEAPLVFEVTLLEVR
XP_005110253	LAVALMDVGEKCELFTDARYAYGPLGRD------PD---IPKDASITYTIELINVQ
OCT89685	LGVRTMQLGEVAFLLTNSLYAFGLLG------RDPD---IPSDASLLYKVTLLCVR
XP_018106932	LGVRTMQLGEVAFLLTNSLYAFGLLG------RDPD---IPSDASLLYKVTLLCVR
XP_022609905	ECVISMQKGEITLLLADSQYAYGLLG------REPD---IPAWAPLLYQLQLLDIR
XP_023278137	ECVISMQKGEITLLLADSQYAYGLLG------REPD---IPAWAPLLYQLQLLDIR
XP_023151164	ECVMSMQKGEITLLLADSQYAYGLLG------REPD---IPAWTPLLYQLQLLDIR
OON20683	LSLPLAERDERFELITDARFAYGSLG------RDPD---IPGDATLRYEVHLLSSS
XP_014340350	LCTLSMHPGEVSILITNAQYAYGHQG------RDPD---VPADAPLLYEVQLLCVR
XP_012284026	LAIALMDVGEVATIEVHSRFAYGDLGK---------EPNIPSNATIIYNVELKSVG
OAD61952	LAIALMNVNEVAEIEVDPRFAYGSLGK---------EPNIPPNTTILYTVELKSSK
XP_023962949	LGVQSMQLGEVSFLLTSFLYAYGHLG------RDPD---IPSDASLLYEVTLLQIR
XP_008121023	LAVPSMQVGEVALFLVSPTCAYGSLG------REPD---IPPNATLLYEVTLLQVQ
XP_017763036	LAIALMNVNEVAEIEVDPRFAYGSLGK---------EPNIPPNTTILYTVELKSSK
XP_017691650	LGVPTMQPGEISFFLAACPYAYGRPG------REPD---VPPEAPLLFEVTLLEVR
XP_012710521	ECVMSMQKGEIALLLTDSQYAYGLLG------REPD---IPAWAPLLYQLQLLDIR
XP_015819997	ECVMSMKKGEIALLLADSQYGYGLLG------REPD---IPAWAPVLYQLQLLDTR
XP_023962948	LGVQSMQLGEVSFLLTSFLYAYGHLG------RDPD---IPSDASLLYEVTLLQIR
XP_019921720	LAVALMEEGQVIELQTDARFAYGEKGRK------PD---IPPNSSIAYIIELVKKD
XP_020904887	VGVALIELSEVCELVTDARYAYGEQGRSG---DNP----VPPNAKITYEIELLAVR
XP_024138547	ECVLSMQMGEITLLLADSQYAYGLLG------REPD---IPAWAPILYQLQLQDIR
AWP08482	ECVMSMQKGEITLLLADSRYAYGLLG------REPD---IPAWAPLLYQLQLLGIR
CDQ67637	ECAMAMQQDEITLLLADSQYTYGLLG------REPD---IPAWAPLLYQLQLLNIR
XP_011423840	LAVALMEEGQVIELQTDARFAYGEKGRK------PD---IPPNSSIAYIIELVKKD
XP_014808190	LGVPTMQPGEVSFFLAAFPYGYGRPG------REPD---VPPEAPLLFEVTLLEVR
XP_007059625	LGVQSMQLGEVSFLLTSFQYAYGHLG------RDPD---IPSDASLLYEVTLLQIR
XP_024138546	ECVLSMQMGEITLLLADSQYAYGLLG------REPD---IPAWAPILYQLQLQDIR
EMP36068	LGVQSMQLGEVSFLLTSFQYAYGHLG------RDPD---IPSDASLLYEVTLLQIR
XP_024138543	ECVLSMQMGEITLLLADSQYAYGLLG------REPD---IPAWAPILYQLQLQDIR
OXU31097	LAVALMDLEEEAEVETASRFAYGSLGRKA----TASFPEIRPDAKITYNIVLKSIE
XP_022202921	FAIPLMDVDEEAELVVGPRFAFGARGRQ---------PDVPPGATLHYTVTLLSAD
XP_006557516	LAIALMDVNEIAEIEVDPRFAYGSLGK---------EPNIPSNATILYTVELKSSE
XP_016770764	LAIALMDVNEIAEIEVDPRFAYGSLGK---------EPNIPSNATILYTVELKSSE
XP_006615021	LAIALMDVNEIAEIEVDPRFAYGSLGK---------EPNIPSNATILYTVELKSSE
XP_008546855	LALALMDVGEVAEIEIAARFGYGDVGR---------QPEIPAGAKLFYNVELKSVE

```
XP_006557515       LAIALMDVNEIAEIEVDPRFAYGSLGK---------EPNIPSNATILYTVELKSSE
XP_007254541       ECAISMKKEEIALLLADSQYTYGLLG------RDPD---IPPWTPILYQLQLVEIR
XP_006615020       LAIALMDVNEIAEIEVDPRFAYGSLGK---------EPNIPSNATILYTVELKSSE
XP_006557514       LAIALMDVNEIAEIEVDPRFAYGSLGK---------EPNIPSNATILYTVELKSSE
XP_011053698       LTIVLMELGEIAEIKIDPRFAYGTRGE----------GSIPPNATITYTVELKTIE
XP_007254540       ECAISMKKEEIALLLADSQYTYGLLG------RDPD---IPPWTPILYQLQLVEIR
XP_006615019       LAIALMDVNEIAEIEVDPRFAYGSLGK---------EPNIPSNATILYTVELKSSE
XP_016913411       LAIALMDVNEIAEIEVDPRFAYGSLGK---------EPNIPSNATILYTVELKSSE
XP_017559733       ECAISMKKGEIALLLADSQYTYGLLG------REPD---IPAWTPILYQLQLIEIR
PKU40687           LGVPTMQPGEVSFFLAAFPYGYGHPG------REPD---VPPEAPLLFEVTLLEVR
XP_016913410       LAIALMDVNEIAEIEVDPRFAYGSLGK---------EPNIPSNATILYTVELKSSE
EGI65888           LTIVLMELGEIAEIKIDPRFAYGTRGE----------GSIPPNATITYTVELKTIE
XP_014252350       FALALMDKGEEAEVVIASRFAYGSLG------RKPD---IPPDAVLTYRLTLHNVR
XP_012165944       LAIALMDVNEVAEIEIDPRFAYGSLGK---------EPNIPPNATILYTVELKSSE
XP_016913409       LAIALMDVNEIAEIEVDPRFAYGSLGK---------EPNIPSNATILYTVELKSSE
XP_011053696       LTIVLMELGEIAEIKIDPRFAYGTRGE----------GSIPPNATITYTVELKTIE
XP_019934661       ECVMSMQRGEITLLLADSLYAYGLLG------REPD---IPAWTPLLYQLQLLDIR
XP_008304064       ECVMSMQKGEITLLLADSQYAYGLLG------REPD---IPAWAPLLYQLQLLDIR
XP_011053695       LTIVLMELGEIAEIKIDPRFAYGTRGE----------GSIPPNATITYTVELKTIE
CAX74525           LSIPLAEHKETFELITDARFAYGSRG------RDPD---IPSGAKLTYHIEILKVD
XP_023811597       ECVLSMQMGEITLLLADSQYAYGLLG------REPD---IPAWAPTLYQLQLLDIR
EFZ15418           LTIILMELDEITQILVHPRFAYGSQGK----------ESIPPNANITYTVELKMIE
OPJ70235           LGVPTMQPGEVAFFLAAFPYGYGHPG------REPD---VPPEAPLLFEVTLLEVR
AAX27902           LSIPLAEHKETFELITDARFAYGSRG------RDPD---IPSGAKLTYHIEILKVD
XP_011053694       LTIVLMELGEIAEIKIDPRFAYGTRGE----------GSIPPNATITYTVELKTIE
XP_011164040       LTIILMELDEITQILVHPRFAYGSQGK----------ESIPPNANITYTVELKMIE
XP_011150605       LAIAMMDVNEIAVIEVDPRFAYGILGQ---------RPDIPPDATIEYTVELKTVD
EFN77170           LAIAMMDVNEIAVIEVDPRFAYGILGQ---------RPDIPPDATIEYTVELKTVD
XP_011164039       LTIILMELDEITQILVHPRFAYGSQGK----------ESIPPNANITYTVELKMIE
AAH90698           ECAITMKKGEIALLLADSQYTYGLLG------REPD---IPAWAPLLYQLQLLDFR
XP_014606393       LAIALMNVGETAVVEVASRFAYGTLGV---------EPDIPPNATIWYTVELKSVE
XP_003700328       LAIALMDTDEVAEIEVNPRFAYGKLGK---------EPNIPSDATILYTVELKSVE
XP_683608          ECAITMKKGEIALLLADSQYTYGLLG------REPD---IPAWAPLLYQLQLLDFR
XP_014606392       LAIALMNVGETAVVEVASRFAYGTLGV---------EPDIPPNATIWYTVELKSVE
XP_023726255       LALPLMDVGEVALLEVGPRFAYGSQGRE---------PDIPCDVTVMYTVELLSSE
XP_011404052       IVASLMQRGEICCVKANPKYAYGSQG------RSPN---VPPESPVMYELELLEVL
XP_018337850       LTIVLMELGEIAEIKIDPRFAYGTRGE----------ESIPPNATITYIVELKAIE
XP_023726263       LALPLMDVGEVALLEVGPRFAYGSQGRE---------PDIPCDVTVMYTVELLSSE
XP_012689022       ECAISMRKEEIALLLADSQYTYGLLG------REPD---IQAWAPILYQLQLLDFR
XP_015190182       LAIALMNVGEIAVVEVASRFAYGTLGV---------EPDIPPNATIWYTVELKSFH
XP_024943519       LAIALMNIGEIAEVEVGPRFGYGDIGN---------KPNIPGGAKIFYTVELKAIE
XP_015437909       LAIALMDTGEIAEIEINPRFAYGHLGK---------PPSIPPAAIILYTVELISTE
KYN13005           LTIVLMELGEIAEIKIDPRFAYGTRGE----------GSIPPNATITYTVELKAIE
XP_024943518       LAIALMNIGEIAEVEVGPRFGYGDIGN---------KPNIPGGAKIFYTVELKAIE
XP_012526171       LTIILMELNEIAEILIHPRFAYGPHGN----------KSIPPDSTITYTVHLKAIE
XP_012237396       LAIALMDVNEVAEIEIDPRFAYGSLGK---------EPNIPPNATILYTVELKSSE
XP_018372055       LTIVLMELGEIAEIKIDPRFAYGTRGE----------GSIPPNATITYTVELKAIE
XP_015600846       LAIALMNIGEIAEVEVGPRFGYGDIGN---------KPNIPGGAKIFYTVELKAIE
XP_018372054       LTIVLMELGEIAEIKIDPRFAYGTRGE----------GSIPPNATITYTVELKAIE
XP_023811598       ECVLSMQMGEITLLLADSQYAYGLLG------REPD---IPAWAPTLYQLQLLDIR
XP_013086934       LAAALMEQGEKCELRTDAKYAYGSLGRE------PD---IPKDSKLIYILELLHVQ
XP_012340112       LAIALMDVNEIAEIEVDPRFAYGSLGK---------EPNIPSNATILYTVELKSSE
XP_012064601       LTIVLMELGEIAEIKINPRFAYGTRGE----------GSVPPNATITYIVELKAIE
XP_021933338       LALPLMDVGEVALLEIGPRFAYGALGRE---------PDVPRDATVVYTVELLSAD
XP_020469449       ECVMSMQEGELTLLLTHSQYAYGHLG------REPD---IPAWAPLLYHLQLLDIR
XP_012340111       LAIALMDVNEIAEIEVDPRFAYGSLGK---------EPNIPSNATILYTVELKSSE
XP_014772499       IAVALMELNEVAELKCDAKYAYGSQGRF------PD---VPSNTAIVYTLELLDKE
XP_021933337       LALPLMDVGEVALLEIGPRFAYGALGRE---------PDVPRDATVVYTVELLSAD
```

XP_002425448	LAVPLMNVGEEAIVVVSPRFGYGSVGNL---------PKIPPNATITYEVTLVNVL
XP_018053112	LTIVLMELGEIAEIKINPRFAYGTRGE----------GSVPPNATITYIVELKAIE
XP_023811595	ECVLSMQMGEITLLLADSQYAYGLLG------REPD---IPAWAPTLYQLQLLDIR
XP_003692338	LAIALMDVNEIAEIEVDPRFAYGSLGK---------EPNIPSNATILYTVELKSSE
KQK73886	LGVPTMQPGEVAFFLAAFPYGYGRPG------REPD---VPPEAPLLFEVTLLEVR
XP_021933339	LALPLMDVGEVALLEIGPRFAYGALGRE---------PDVPRDATVVYTVELLSAD
XP_018025482	LVVPLMDRGEESEVYIHSRFAYGSKGDG---------ERVPADASLLCTVKLLDYK
XP_019335449	LGVPSMQLGEVSFFLASFQYAYGRLG------REPD---IPADALLLYEVTLLLAR
XP_014772498	IAVALMELNEVAELKCDAKYAYGSQGRF------PD---VPSNTAIVYTLELLDKE
XP_023811594	ECVLSMQMGEITLLLADSQYAYGLLG------REPD---IPAWAPTLYQLQLLDIR
XP_001846585	MTLKLMNVGERAEVIVDARFAYGDQGYKNEDHPE---RSVPPNAKITYTVELVSSK
XP_014470017	LAVALMDVHEVAEIEIDPRFAYSKLGK---------KPDIPPDAVMSYTVELKDFE
XP_002111046	LVVALMNVGETVCVFANSRYGYAQYGS-----SKHN---VPQDADLEFEVTLIDVS
XP_014772494	IAVALMELNEVAELKCDAKYAYGSQGRF------PD---VPSNTAIVYTLELLDKE
KZS20178	LIVALMDCGEISQVEVAPRFAYGSLGNG---------KDIPPDATILYTIELLEIS
XP_014470016	LAVALMDVHEVAEIEIDPRFAYSKLGK---------KPDIPPDAVMSYTVELKDFE
KFM75734	LVICLMEKGEKARVVIPSKLAYGEIG------RTPD---VPPNSDIKCDLELKEVE
XP_018900271	FVIALMDVGEVAEVFVGPRFGFGKIGRQEP-----DKPEVPPNANLHYTMKLIKVE
XP_020392992	LCVPLMYIHETALILTDSKFCSGRASS-----EASE---VKPGSRLLLEVHLLSAS
XP_016376789	ECTISMKQGEIALLLADSQYTYGHLG------REPD---IPAWAPLLYQLQLLDFR
XP_016345079	ECAISMTQGEIALLLADSQYTYGHLG------REPD---IPAWDPLLYQLQLLDFR
XP_014106477	LGVPTMQPGEVSFFLAAFPYAYGRPG------REPD---VPPEAPLLFEVTLLEVR
XP_014237957	LALALMNTEEEAEVKVGARFGYGKTGR------TSPLPVIPPNTDLVYNVTLKSVT
XP_016376788	ECTISMKQGEIALLLADSQYTYGHLG------REPD---IPAWAPLLYQLQLLDFR
XP_016376787	ECTISMKQGEIALLLADSQYTYGHLG------REPD---IPAWAPLLYQLQLLDFR
CDS28193	LALPFVELMEDFELVTDARFAYGSKG------NPPN---IPPNAKLTYRISLIACN
KOC65132	LAIALMDVGEVAEIEVNPRFAYGSLGK---------EPNIPPNACILYTVELKSSQ
XP_017790334	LAIALMDVGEVAEIEVNPRFAYGSLGK---------EPNIPPNACILYTVELKSSQ
XP_020790728	ECVLSMQTGEIALLLADSQYAYGLLG------RQPD---IPAWAPLLYQLQLCDVR
XP_012221110	LTISLMDKDEIAEVYVDPRFGYGDQPE----------EKIPPNSVLKYIVELKEII
GAA30660	LSLPLAERDERFELVTDARFAYGSLG------RDPD---IPGDATLRYEVHLLSSS
XP_012221101	LTISLMDKDEIAEVYVDPRFGYGDQPE----------EKIPPNSVLKYIVELKEII
XP_012221093	LTISLMDKDEIAEVYVDPRFGYGDQPE----------EKIPPNSVLKYIVELKEII
XP_018649440	LSIPLAEHKEIFEMTTDSRFAYGSRG------RDPG---IPSGAKLTYRIEVLKVD
EFX68474	LIVALMDCHEISQVEVGPRFAYGSLGNG---------KDIPPEATILYTIELLEVS
XP_023229640	LTVTLMEKGELAEVIIPARLAYGEQG------KLPE---IPPNSVINYKIRLVDVR
EAT32833	MALKLMNVGEQAEVIVSSRFAYGEQGLKNEDHPE---RTVPPNEKITYTVELVSTK
XP_016344167	ECTISMKQGEIALLLADSQYTYGHLG------REPD---IPAWAPLLYQLQLLDFR
XP_016344168	ECTISMKQGEIALLLADSQYTYGHLG------REPD---IPAWAPLLYQLQLLDFR
XP_016344166	ECTISMKQGEIALLLADSQYTYGHLG------REPD---IPAWAPLLYQLQLLDFR
XP_016376234	ECAISMMQGEIALLLADSQYTYGHLG------REPD---IPAWAPLLYQLQLLDFR
XP_007906811	ICVPLMEVEETSLILADVKFCYLSKEC-----QNGS---LPPGTKLLLEVKLLSVT
KYN04145	LTISLMELDEIAEIKIDPRFAYGTRGE----------GAVPPNATITYIVELKAIE
XP_014282311	FALALMDKGEVAEIKITPRFGYGSLGKR---------PEIPPGANLIYTVTLLDVK
XP_015109871	LAIALMDVGEAAEIVIAPRFAYGDIGK---------EPDIPPGATLYYNVNLKAVK
CDS42647	LALPLAELKEVFELKTDARFAYGDMG------KMPD---IPGKAKLTYHITLNECG
XP_011870270	LAIVLMELGEIAEIVINERFAYGMSGT----------STIPSLATVTYVVELKAIE
XP_011870269	LAIVLMELGEIAEIVINERFAYGMSGT----------STIPSLATVTYVVELKAIE
XP_018977514	ECVISMTKGEIALLLADSQYTYGHLG------REPD---IPAWAPLLYQLELLDFR
XP_007906810	ICVPLMEVEETSLILADVKFCYLSKEC-----QNGS---LPPGTKLLLEVKLLSVT
CDS17605	LALPLAELKEVFELKTDARFAYGDMG------KMPD---IPGKAKLTYHITLNECG
XP_014206998	LALALMETEEEAIIEAEARFGYGSLGRPA----VGPFPEIPSNAKLIYNVTLKRVG
XP_018393954	LTISLMELDEIAEIKIDPRFAYGTRGE----------GAVPPNATITYIVELKAIE
XP_015929100	LVFCLMEKDEVAKVKIPPKLAYGKLG------RSPE---IPPESELEYEVHLKDVQ
ELU06040	LCVALADEHETFELFTAAQYAYGAFGKP-----DSG---IPPNSDIIYEIAVLKVE
XP_024353326	LALPLAELKEVFELKTDARFAYGDMG------KMPD---IPGKAKLTYHITLNECG
EAT35561	MALKLMNVGEQAEVIVSSRFAYGEQGLKNEDHPE---RTVPPNEKITYTVELVSTK
XP_018359770	LTIPLMELGEIAEIRIDPRFSYGTHGE----------GSVPPNATITYTVELKAIK

EAT35560	MALKLMNVGEQAEVIVSSRFAYGEQGLKNEDHPE---RTVPPNEKITYTVELVSTK
XP_018319727	VVLALMNVGETSKIKIEPRLAYGSRGLE---------SKIPPNATILYEVTLLSAD
XP_001662371	MALKLMNVGEQAEVIVSSRFAYGEQGLKNEDHPE---RTVPPNEKITYTVELVSTK
XP_018319726	VVLALMNVGETSKIKIEPRLAYGSRGLE---------SKIPPNATILYEVTLLSAD
XP_001662370	MALKLMNVGEQAEVIVSSRFAYGEQGLKNEDHPE---RTVPPNEKITYTVELVSTK
XP_012269548	LVIALMDVGEVAEVEVESRFAYGDLGR---------EPDIPGGAKLFYTLELLSVE
PAA92621	IAVLLMELDEQCSLVTSARYAYGYSAEV----KTSTGVVIPPGSVLHCTVTLVSVD
XP_019867812	FCIGIMNVGEQSMVKVAPLFAYGDKGLS---------EXIPGKATLIFNIELKALK
PAA53270	IAVLLMELDEQCSLVTSARYAYGYSAEV----KTSTGVVIPPGSVLHCTVTLVSVD
PAA57938	IAVLLMELDEQCSLVTSARYAYGYSAEV----KTSTGVVIPPGSVLHCTVTLVSVD
XP_966821	VAIGLMNVGEKCSLKIEPRLAFGGVGLP---------PKIPPNATVVYDIELVGVE
KXJ84517	MALKLMNVGEQAEVIVAARFAYGELGLKDEDHPE---RTVPPNEKITYTVDLVSTK
XP_023345477	LILPLMRVGETCRIESESAFCYGEQG------DGDR---VPGNHKLYLEVELISAR
XP_011494478	LGITLMDLEEEAELEICPRFAYGALGR------NLPLPEIPPNSKLTYNVILKDIK
XP_023345475	LILPLMRVGETCRIESESAFCYGEQG------DGDR---VPGNHKLYLEVELISAR
XP_016940923	MVVPMMQIGEVAQVSVDPRFGYGSVGLK---KDGESEYLVPPDSHLTYEIELLDIK
XP_023345476	LILPLMRVGETCRIESESAFCYGEQG------DGDR---VPGNHKLYLEVELISAR
PNF41144	LALPLMDVGEVALLEVGPRFAYGSQGRE---------PDIPCDVTVMYTVELLSSE
XP_019932587	MALKLMNVGEQAEVIVAARFAYGELGLKDEDHPE---RTVPPNEKITYTVELVSTK
XP_019932586	MALKLMNVGEQAEVIVAARFAYGELGLKDEDHPE---RTVPPNEKITYTVELVSTK
XP_016028426	MVLPMLQVGEVSQVSVDSRFGYGSIGLK---KEGESEYLVPPDANLTYEIELLDIK
XP_016958803	MVVPMMQIGEVAQVSVDPRFGYGSLGLK---KDGESEYLVPPDSHLTYEIELLDIK
EKC18274	LAVALMEEGQVIELQTDARFAYGEKGRK------PD---IPPNSSIAYIIELVKKD
PNF41145	LALPLMDVGEVALLEVGPRFAYGSQGRE---------PDIPCDVTVMYTVELLSSE
XP_011636531	LTIVLMDLGEIAEIIIHPRFAYGACGN----------DPIPPNATITYTVELKAIE
XP_017014991	MVVPMLQVGEVAQVSVDPRFGYGSLGLK---KDGESDYLVPPDSHLTYEIELLDIK
XP_001974711	MVLPMLQVGEVAQVSVDPRFGYGSLGLK---KDGESAYLVPPDSHLTYEIELLDIK
XP_024874265	LTIVLMELGEIAVVVVDPRFAYGTRGT----------ASIPSNATITYTVELKEIK
NP_611353	MVLPMLQVGEVSQVSVDSRFGYGSLGLK---KEGESEYLVPPDAHLTYEIELLDIK
XP_016984960	MVVPMLQVGEVAQVSVDPRFGYGSIGLK---KDGESDYLVPPDSHLTYEIELLDVK
XP_017054378	MVVPMLQVGEVAQVSVDPRFGYGSLGLK---KEGESEYLVPPDSHLTYEIELLDIK
XP_024874264	LTIVLMELGEIAVVVVDPRFAYGTRGT----------ASIPSNATITYTVELKEIK
XP_019545364	IALKLMNVGEQAEVIVAARFAYGELGLKDEDHPE---RTVPPNEKITYTVELVSTK
XP_019545363	IALKLMNVGEQAEVIVAARFAYGELGLKDEDHPE---RTVPPNEKITYTVELVSTK
XP_014670901	MAVTLMELEETCELKINARFAYGERGRL------PD---IPSNATVTYQLSLLGIY
XP_017079421	MVVPMLRIGEVAQVSVDPRFGYGSLGLK---KDGETDYLVPPDSHLTYEIELLDIK
XP_013113222	MTIPLMDIGEVAEVQVDARFAYGSLGLK---NDTDPELSIPPESKLTYEVHLKDAI
XP_002034480	MVLPMLQVGEVSQVSVDSRFGYGSIGLK---KEGESEYLVPPDANLTYEIELLDIK
XP_017031858	MVLPMMLVGEVSQVSVDPRFGYGSIGLK---REGESEFVVPPDSNLTYEIELLDSK
XP_015788695	LIVPLMNVGEIAEVIVTPRFAYGEKG------REPD---IPPNASLKLIIELIAID
XP_017117238	MVVPMMQVGEVAQVSVDPRFGYGSMGLK---KDGESEYLVPPDSHLTYEIELLDIK
XP_015364528	LALSLMNVGEIAELKIASRFGYGEKGLS---------PKVLGGARLVYTVELVTVK
XP_017836078	MVLPMMHVGEVSEISVDARFGYGAIGLK---KDNSTDVLIPPEAHLSYEIELLEVK
XP_316645	MALKLMNEGEVAEVIVNPRFAYGELGVKDPTEQDPVIRTVPPNATITYTVELVSMR
XP_002091890	MVLPMLQVGEVAQVSVDPRFGYGSLGLK---KDGESTYLVPPDSHLTYEIELLDTK
XP_009022869	LCVPLMHPGEVCELVTEARFAYGEKGRK------PD---IPGNARITYELELLDVQ
XP_020815089	MVLPMMLVGEVSQVSVDPRFGYGSIGLK---REGDSEFVVPPDSNLTYEIELLDSK
KYO18601	LGVPSMQLGEVSFFLASFQYAYGRLG------REPD---IPADALLLYEPEAG---
XP_023307145	MVVPLMNIGEVAEVKVDPRFAYGSLGLK---NDKEPALSIPPEATITYEVHLIDSK
XP_004526322	MVIPLMNVGEVAEVTIDARFAYGSIGMK---NNENECASIPPDTTLTYEVHLVESK
XP_017468465	MAIPLMSVGEVAEIRIDTRFAYGSLGFK---NENNESASVPADANLIYEVHLLDSK
XP_011213061	MVIPLMNVGEVAEVTIDARFAYGSLGLK---NDENENASVPADSTLIYEVHLQDCK
ETN59059	MALKLMNEGEQAEVVVNPRFAYGEIGLNNETDPK---RSVPPNATITYTVELLSTK
XP_019877780	VAVALMNLNERCLLKIAPRLAYGQKGLP---------GKVPENATVVYDIELTAVE
XP_001951673	LALSLMNVGEIAELKIASRFGYGEKGLN---------PKVLGGARLVYTVELVTVK
XP_002062976	MVLPMMQVGEVAQVSVDPRFAYGSTGLKIN-KDDDTEVVVPPDSHLTYEIELLDAK
XP_001986816	MVLPMMHVGEVAEVTVDQRFGYGTAGLK---TDNPPEIVIPPNAQLIYELELVDVK
XP_022181773	LALSLMNVGEIAELKIASRFGYGEKGLS---------PKVLGGARLVYIVELVSVK

```
XP_002048800      MVLPMMHVGEVSQVNVDQRFGYGTIGLK---TENPPEIIVPPNANLVYQVELVDVK
XP_018801327      MVIPLMNVGEIAEVTIDARFAYGSLGLK---NDENENANVPADSTLIYEVHLQDCK
XP_020300622      LALALMDVGEIALVYVHPRFAYGEQGT----------EDIPPNSIITYIIELKKIT
XP_011193416      MVIPLMNIGEVAEVSVDSRFAYGSLGLK---NEKDDSANVPADSTLTYEVHLLDCK
XP_017960399      MVLPMMHVGEVAQVSIDQRFGYGTIGLK---TENPPETVIPPNANLIYELELVDVQ
ACO13176          YILPIMNVNEVVEVITESDFMYGDQGL-----DSPP---VPPKATLQLKLHLIDSV
XP_011350211      LVIVLMNIGEVADVIVEPRFAYGLSGT----------KDIPAGTPLVYTVELLTAR
KRT81327          IVIGLMNIGDIWELEIEPRLAYGTKGLP---------PLIPADAKVLYRV------
XP_023162735      MVLPMMHVGEVAQVSIDQRFGYGTIGLK---TENPPETVIPPNTNLIYELELVDVK
XP_019771996      VALGLMNSSERCLLKIGPRLAFGDRGLP---------PKIPAGATVLFDVELVSHQ
XP_005176045      MVVPLMNVGEVAEVKVDPRFAYGSLGLK---NDADPELNIPPDSKLTYEVHLLDTK
XP_014095636      MVIPLMNIGEVAEVTIDARFAYGSLGLK---NDENESASVPADSTLIYEVHLLNCK
XP_017867395      MVLPMMHVGEVAQVSIDQRFGYGTIGLK---TENPPETVIPPNANLIYELELVDVK
XP_002004550      MVLPMMHVGEVAQVSIDQRFGYGTIGLK---TENPPETVIPPNANLIYELELVDVK
XP_022921247      LSIAMMNVGEICELKIQPRLAFGAKGLP---------PLIPPNTTVIYEVALLSIV
XP_002015767      MILPMLKVGEVAEVVVDPRFGYGTLGLK---KD-DGDFIVPPNALLTYEIELLDSK
XP_001360319      MILPMLKVGEVAEVVVDPRFGYGTLGLK---KD-DGDFIVPPNALLTYEIELLDSK
XP_017147297      MILPMLKVGEVAEVVVDPRFGYGTLGLK---KDNDGDFIVPPNALLTYEIELLDSK
XP_019879021      IAITSMNLHERSLFIIDPGLAYGPEGVN---------EIVPKNATIICVIELTRLM
```
