# Supplementary material for: Compression of Large Sets of Sequence Data Reveals Fine Diversification of Functional Profiles in Multigene Families of Proteins: A Study for Peptidyl-Prolyl cis/trans Isomerases (PPIase)
Source: Biomolecules. 2019 Feb 11;9(2):59. doi: 10.3390/biom9020059 (PMC6406343; doi:10.3390/biom9020059)
Supplement: Supplementary file 1 [file biomolecules-09-00059-s001.zip › Figure S9B-Names of FKBP38s-nam.pdf]

FKBP38s

(1)	NP_036313	peptidyl-prolyl cis-trans isomerase FKBP8 isoform 1 [Homo sapiens].
(2)	NP_001295302	peptidyl-prolyl cis-trans isomerase FKBP8 isoform 2 [Homo sapiens].
(3)	XP_003817801	peptidyl-prolyl cis-trans isomerase FKBP8 isoform X4 [Pan paniscus].
(4)	XP_010384957	PREDICTED: peptidyl-prolyl cis-trans isomerase FKBP8 isoform X2 [Rhinopithecu
(5)	XP_003817802	peptidyl-prolyl cis-trans isomerase FKBP8 isoform X2 [Pan paniscus].
(6)	XP_011747960	peptidyl-prolyl cis-trans isomerase FKBP8 isoform X4 [Macaca nemestrina].
(7)	XP_003915233	peptidyl-prolyl cis-trans isomerase FKBP8 isoform X4 [Papio anubis].
(8)	EHH59371	hypothetical protein EGM_09459 [Macaca fascicularis].
(9)	XP_005588521	PREDICTED: peptidyl-prolyl cis-trans isomerase FKBP8 isoform X4 [Macaca fasci
(10)	XP_002828980	peptidyl-prolyl cis-trans isomerase FKBP8 isoform X4 [Pongo abelii].
(11)	PNJ28395	FKBP8 isoform 1, partial [Pongo abelii].
(12)	XP_011949838	PREDICTED: peptidyl-prolyl cis-trans isomerase FKBP8 isoform X4 [Cercocebus a
(13)	XP_018870801	PREDICTED: peptidyl-prolyl cis-trans isomerase FKBP8 isoform X2 [Gorilla gori
(14)	XP_007993999	PREDICTED: peptidyl-prolyl cis-trans isomerase FKBP8 isoform X2 [Chlorocebus
(15)	XP_014978797	PREDICTED: peptidyl-prolyl cis-trans isomerase FKBP8 isoform X2 [Macaca mulat
(16)	XP_011747796	peptidyl-prolyl cis-trans isomerase FKBP8 isoform X2 [Macaca nemestrina].
(17)	XP_003915232	peptidyl-prolyl cis-trans isomerase FKBP8 isoform X2 [Papio anubis].
(18)	XP_002828981	peptidyl-prolyl cis-trans isomerase FKBP8 isoform X2 [Pongo abelii].
(19)	XP_010328455	PREDICTED: peptidyl-prolyl cis-trans isomerase FKBP8 isoform X2 [Saimiri boli
(20)	XP_008969279	peptidyl-prolyl cis-trans isomerase FKBP8 isoform X3 [Pan paniscus].
(21)	XP_011845848	PREDICTED: peptidyl-prolyl cis-trans isomerase FKBP8 isoform X1 [Mandrillus l
(22)	XP_024207707	peptidyl-prolyl cis-trans isomerase FKBP8 isoform X6 [Pan troglodytes].
(23)	XP_011526166	peptidyl-prolyl cis-trans isomerase FKBP8 isoform X7 [Homo sapiens].
(24)	XP_011949835	PREDICTED: peptidyl-prolyl cis-trans isomerase FKBP8 isoform X2 [Cercocebus a
(25)	XP_017356796	PREDICTED: peptidyl-prolyl cis-trans isomerase FKBP8 isoform X2 [Cebus capuci
(26)	XP_008969278	peptidyl-prolyl cis-trans isomerase FKBP8 isoform X1 [Pan paniscus].
(27)	NP_001247999	peptidyl-prolyl cis-trans isomerase FKBP8 [Macaca mulatta].
(28)	XP_024307207	peptidyl-prolyl cis-trans isomerase FKBP8 isoform X1 [Homo sapiens].
(29)	XP_016791013	peptidyl-prolyl cis-trans isomerase FKBP8 isoform X5 [Pan troglodytes].
(30)	XP_009192156	peptidyl-prolyl cis-trans isomerase FKBP8 isoform X3 [Papio anubis].
(31)	XP_024307210	peptidyl-prolyl cis-trans isomerase FKBP8 isoform X5 [Homo sapiens].
(32)	XP_005588520	PREDICTED: peptidyl-prolyl cis-trans isomerase FKBP8 isoform X3 [Macaca fasci
(33)	XP_011747874	peptidyl-prolyl cis-trans isomerase FKBP8 isoform X3 [Macaca nemestrina].
(34)	XP_024092631	peptidyl-prolyl cis-trans isomerase FKBP8 isoform X3 [Pongo abelii].
(35)	XP_004060362	PREDICTED: peptidyl-prolyl cis-trans isomerase FKBP8 isoform X1 [Gorilla gori
(36)	XP_011949836	PREDICTED: peptidyl-prolyl cis-trans isomerase FKBP8 isoform X3 [Cercocebus a
(37)	XP_007993998	PREDICTED: peptidyl-prolyl cis-trans isomerase FKBP8 isoform X1 [Chlorocebus
(38)	XP_014978796	PREDICTED: peptidyl-prolyl cis-trans isomerase FKBP8 isoform X1 [Macaca mulat
(39)	XP_009251263	peptidyl-prolyl cis-trans isomerase FKBP8 isoform X1 [Pongo abelii].
(40)	XP_011747747	peptidyl-prolyl cis-trans isomerase FKBP8 isoform X1 [Macaca nemestrina].

```
( 41)  XP_017822960     PREDICTED: peptidyl-prolyl cis-trans isomerase FKBP8 isoform X2 [Callithrix j
( 42)  XP_009192157     peptidyl-prolyl cis-trans isomerase FKBP8 isoform X1 [Papio anubis].
( 43)  XP_010328454     PREDICTED: peptidyl-prolyl cis-trans isomerase FKBP8 isoform X1 [Saimiri boli
( 44)  XP_011845855     PREDICTED: peptidyl-prolyl cis-trans isomerase FKBP8 isoform X2 [Mandrillus l
( 45)  XP_012299012     peptidyl-prolyl cis-trans isomerase FKBP8 isoform X2 [Aotus nancymaae].
( 46)  XP_011949833     PREDICTED: peptidyl-prolyl cis-trans isomerase FKBP8 isoform X1 [Cercocebus a
( 47)  XP_017356795     PREDICTED: peptidyl-prolyl cis-trans isomerase FKBP8 isoform X1 [Cebus capuci
( 48)  XP_014401247     PREDICTED: peptidyl-prolyl cis-trans isomerase FKBP8 isoform X1 [Myotis brand
( 49)  EPQ12447         Peptidyl-prolyl cis-trans isomerase FKBP8 [Myotis brandtii].
( 50)  XP_008154612     PREDICTED: peptidyl-prolyl cis-trans isomerase FKBP8 isoform X2 [Eptesicus fu
( 51)  XP_016066232     PREDICTED: peptidyl-prolyl cis-trans isomerase FKBP8 isoform X2 [Miniopterus 
( 52)  XP_012584673     PREDICTED: peptidyl-prolyl cis-trans isomerase FKBP8 isoform X1 [Condylura cr
( 53)  XP_015413209     PREDICTED: peptidyl-prolyl cis-trans isomerase FKBP8 isoform X2 [Myotis david
( 54)  XP_022262557     peptidyl-prolyl cis-trans isomerase FKBP8 isoform X2 [Canis lupus familiaris]
( 55)  XP_023103313     peptidyl-prolyl cis-trans isomerase FKBP8 isoform X2 [Felis catus].
( 56)  XP_019271925     PREDICTED: peptidyl-prolyl cis-trans isomerase FKBP8 isoform X3 [Panthera par
( 57)  XP_007088417     PREDICTED: peptidyl-prolyl cis-trans isomerase FKBP8 isoform X2 [Panthera tig
( 58)  XP_002761955     PREDICTED: peptidyl-prolyl cis-trans isomerase FKBP8 isoform X1 [Callithrix j
( 59)  XP_014921066     PREDICTED: peptidyl-prolyl cis-trans isomerase FKBP8 isoform X2 [Acinonyx jub
( 60)  XP_005972346     PREDICTED: peptidyl-prolyl cis-trans isomerase FKBP8 isoform X2 [Pantholops h
( 61)  XP_006058025     PREDICTED: peptidyl-prolyl cis-trans isomerase FKBP8 isoform X2 [Bubalus buba
( 62)  XP_017905457     PREDICTED: peptidyl-prolyl cis-trans isomerase FKBP8 isoform X2 [Capra hircus
( 63)  XP_019271923     PREDICTED: peptidyl-prolyl cis-trans isomerase FKBP8 isoform X1 [Panthera par
( 64)  XP_008542456     PREDICTED: peptidyl-prolyl cis-trans isomerase FKBP8 isoform X2 [Equus przewa
( 65)  XP_006186302     PREDICTED: peptidyl-prolyl cis-trans isomerase FKBP8 isoform X2 [Camelus feru
( 66)  XP_006199114     PREDICTED: peptidyl-prolyl cis-trans isomerase FKBP8 isoform X2 [Vicugna paco
( 67)  XP_010970466     PREDICTED: peptidyl-prolyl cis-trans isomerase FKBP8 isoform X2 [Camelus bact
( 68)  XP_024416679     peptidyl-prolyl cis-trans isomerase FKBP8 isoform X2 [Desmodus rotundus].
( 69)  XP_005652984     peptidyl-prolyl cis-trans isomerase FKBP8 isoform X2 [Sus scrofa].
( 70)  XP_014687059     PREDICTED: peptidyl-prolyl cis-trans isomerase FKBP8 isoform X2 [Equus asinus
( 71)  XP_015344756     PREDICTED: peptidyl-prolyl cis-trans isomerase FKBP8 isoform X1 [Marmota marm
( 72)  XP_012299010     peptidyl-prolyl cis-trans isomerase FKBP8 isoform X1 [Aotus nancymaae].
( 73)  XP_019819388     PREDICTED: peptidyl-prolyl cis-trans isomerase FKBP8 isoform X2 [Bos indicus]
( 74)  ELW67147         Peptidyl-prolyl cis-trans isomerase FKBP8 [Tupaia chinensis].
( 75)  XP_005873908     PREDICTED: peptidyl-prolyl cis-trans isomerase FKBP8 isoform X2 [Myotis brand
( 76)  XP_016066229     PREDICTED: peptidyl-prolyl cis-trans isomerase FKBP8 isoform X1 [Miniopterus 
( 77)  XP_008154611     PREDICTED: peptidyl-prolyl cis-trans isomerase FKBP8 isoform X1 [Eptesicus fu
( 78)  XP_020740155     peptidyl-prolyl cis-trans isomerase FKBP8 isoform X2 [Odocoileus virginianus
( 79)  XP_003982130     peptidyl-prolyl cis-trans isomerase FKBP8 isoform X1 [Felis catus].
( 80)  XP_019271924     PREDICTED: peptidyl-prolyl cis-trans isomerase FKBP8 isoform X2 [Panthera par
( 81)  XP_007088416     PREDICTED: peptidyl-prolyl cis-trans isomerase FKBP8 isoform X1 [Panthera tig
( 82)  XP_541935        peptidyl-prolyl cis-trans isomerase FKBP8 isoform X1 [Canis lupus familiaris]
```

```
( 83)  XP_012419293     PREDICTED: peptidyl-prolyl cis-trans isomerase FKBP8 isoform X2 [Odobenus ros
( 84)  XP_004688664     PREDICTED: peptidyl-prolyl cis-trans isomerase FKBP8 isoform X2 [Condylura cr
( 85)  XP_015413208     PREDICTED: peptidyl-prolyl cis-trans isomerase FKBP8 isoform X1 [Myotis david
( 86)  XP_006747423     PREDICTED: peptidyl-prolyl cis-trans isomerase FKBP8 [Leptonychotes weddellii
( 87)  XP_017905455     PREDICTED: peptidyl-prolyl cis-trans isomerase FKBP8 isoform X1 [Capra hircus
( 88)  XP_010993437     PREDICTED: LOW QUALITY PROTEIN: peptidyl-prolyl cis-trans isomerase FKBP8 [Ca
( 89)  XP_006058026     PREDICTED: peptidyl-prolyl cis-trans isomerase FKBP8 isoform X3 [Bubalus buba
( 90)  XP_014921065     PREDICTED: peptidyl-prolyl cis-trans isomerase FKBP8 isoform X1 [Acinonyx jub
( 91)  XP_006058024     PREDICTED: peptidyl-prolyl cis-trans isomerase FKBP8 isoform X1 [Bubalus buba
( 92)  XP_001503379     peptidyl-prolyl cis-trans isomerase FKBP8 isoform X1 [Equus caballus].
( 93)  XP_005972345     PREDICTED: peptidyl-prolyl cis-trans isomerase FKBP8 isoform X1 [Pantholops h
( 94)  XP_019271926     PREDICTED: peptidyl-prolyl cis-trans isomerase FKBP8 isoform X4 [Panthera par
( 95)  XP_006186301     PREDICTED: peptidyl-prolyl cis-trans isomerase FKBP8 isoform X1 [Camelus feru
( 96)  XP_006199113     PREDICTED: peptidyl-prolyl cis-trans isomerase FKBP8 isoform X1 [Vicugna paco
( 97)  XP_010970465     PREDICTED: peptidyl-prolyl cis-trans isomerase FKBP8 isoform X1 [Camelus bact
( 98)  XP_003123564     peptidyl-prolyl cis-trans isomerase FKBP8 isoform X1 [Sus scrofa].
( 99)  XP_024416678     peptidyl-prolyl cis-trans isomerase FKBP8 isoform X1 [Desmodus rotundus].
(100)  NP_001192650     peptidyl-prolyl cis-trans isomerase FKBP8 [Bos taurus].
(101)  XP_020740157     peptidyl-prolyl cis-trans isomerase FKBP8 isoform X4 [Odocoileus virginianus
(102)  XP_015344757     PREDICTED: peptidyl-prolyl cis-trans isomerase FKBP8 isoform X2 [Marmota marm
(103)  XP_006147177     PREDICTED: peptidyl-prolyl cis-trans isomerase FKBP8 [Tupaia chinensis].
(104)  XP_020740156     peptidyl-prolyl cis-trans isomerase FKBP8 isoform X3 [Odocoileus virginianus
(105)  XP_014687058     PREDICTED: peptidyl-prolyl cis-trans isomerase FKBP8 isoform X1 [Equus asinus
(106)  XP_004404706     PREDICTED: peptidyl-prolyl cis-trans isomerase FKBP8 isoform X1 [Odobenus ros
(107)  XP_019819387     PREDICTED: peptidyl-prolyl cis-trans isomerase FKBP8 isoform X1 [Bos indicus]
(108)  XP_020008573     LOW QUALITY PROTEIN: peptidyl-prolyl cis-trans isomerase FKBP8 [Castor canade
(109)  XP_007116995     peptidyl-prolyl cis-trans isomerase FKBP8 isoform X2 [Physeter catodon].
(110)  XP_024622992     peptidyl-prolyl cis-trans isomerase FKBP8 isoform X2 [Neophocaena asiaeorient
(111)  XP_012391225     PREDICTED: peptidyl-prolyl cis-trans isomerase FKBP8 isoform X1 [Orcinus orca
(112)  XP_020740154     peptidyl-prolyl cis-trans isomerase FKBP8 isoform X1 [Odocoileus virginianus
(113)  XP_022414296     peptidyl-prolyl cis-trans isomerase FKBP8 isoform X4 [Delphinapterus leucas].
(114)  XP_021587090     peptidyl-prolyl cis-trans isomerase FKBP8 [Ictidomys tridecemlineatus].
(115)  XP_019592316     PREDICTED: peptidyl-prolyl cis-trans isomerase FKBP8 isoform X1 [Rhinolophus
(116)  XP_023989754     peptidyl-prolyl cis-trans isomerase FKBP8 isoform X1 [Physeter catodon].
(117)  XP_024622991     peptidyl-prolyl cis-trans isomerase FKBP8 isoform X1 [Neophocaena asiaeorient
(118)  XP_004277581     PREDICTED: peptidyl-prolyl cis-trans isomerase FKBP8 isoform X2 [Orcinus orca
(119)  XP_017651479     PREDICTED: peptidyl-prolyl cis-trans isomerase FKBP8 isoform X4 [Nannospalax
(120)  XP_022414295     peptidyl-prolyl cis-trans isomerase FKBP8 isoform X3 [Delphinapterus leucas].
(121)  XP_017651477     PREDICTED: peptidyl-prolyl cis-trans isomerase FKBP8 isoform X2 [Nannospalax
(122)  XP_007461122     PREDICTED: peptidyl-prolyl cis-trans isomerase FKBP8 [Lipotes vexillifer].
(123)  XP_007195521     PREDICTED: peptidyl-prolyl cis-trans isomerase FKBP8 [Balaenoptera acutorostr
(124)  XP_019592317     PREDICTED: peptidyl-prolyl cis-trans isomerase FKBP8 isoform X2 [Rhinolophus
```

(125) XP_005001666 peptidyl-prolyl cis-trans isomerase FKBP8 isoform X2 [Cavia porcellus].
(126) XP_008822765 PREDICTED: peptidyl-prolyl cis-trans isomerase FKBP8 isoform X3 [Nannospalax
(127) XP_017651474 PREDICTED: peptidyl-prolyl cis-trans isomerase FKBP8 isoform X1 [Nannospalax
(128) XP_013002970 peptidyl-prolyl cis-trans isomerase FKBP8 isoform X1 [Cavia porcellus].
(129) XP_012924198 peptidyl-prolyl cis-trans isomerase FKBP8 isoform X2 [Heterocephalus glaber].
(130) EHB02679 FK506-binding protein 8 [Heterocephalus glaber].
(131) XP_012924197 peptidyl-prolyl cis-trans isomerase FKBP8 isoform X1 [Heterocephalus glaber].
(132) XP_021535026 peptidyl-prolyl cis-trans isomerase FKBP8 [Neomonachus schauinslandi].
(133) XP_007943554 PREDICTED: peptidyl-prolyl cis-trans isomerase FKBP8 isoform X1 [Orycteropus
(134) XP_011220551 PREDICTED: peptidyl-prolyl cis-trans isomerase FKBP8 isoform X1 [Ailuropoda m
(135) XP_008588853 PREDICTED: peptidyl-prolyl cis-trans isomerase FKBP8 [Galeopterus variegatus]
(136) XP_012916132 PREDICTED: peptidyl-prolyl cis-trans isomerase FKBP8 isoform X1 [Mustela puto
(137) XP_022353384 peptidyl-prolyl cis-trans isomerase FKBP8 isoform X2 [Enhydra lutris kenyoni]
(138) XP_008048663 peptidyl-prolyl cis-trans isomerase FKBP8 isoform X2 [Carlito syrichta].
(139) XP_019652974 PREDICTED: peptidyl-prolyl cis-trans isomerase FKBP8 isoform X2 [Ailuropoda m
(140) XP_008702161 PREDICTED: peptidyl-prolyl cis-trans isomerase FKBP8 [Ursus maritimus].
(141) XP_019497623 PREDICTED: peptidyl-prolyl cis-trans isomerase FKBP8 isoform X1 [Hipposideros
(142) XP_007943555 PREDICTED: peptidyl-prolyl cis-trans isomerase FKBP8 isoform X2 [Orycteropus
(143) XP_002912777 PREDICTED: peptidyl-prolyl cis-trans isomerase FKBP8 isoform X3 [Ailuropoda m
(144) EFB19124 hypothetical protein PANDA_000557, partial [Ailuropoda melanoleuca].
(145) XP_013362739 PREDICTED: peptidyl-prolyl cis-trans isomerase FKBP8 isoform X2 [Chinchilla l
(146) XP_006876371 PREDICTED: peptidyl-prolyl cis-trans isomerase FKBP8 isoform X2 [Chrysochlori
(147) XP_021493693 peptidyl-prolyl cis-trans isomerase FKBP8 isoform X2 [Meriones unguiculatus].
(148) XP_022353383 peptidyl-prolyl cis-trans isomerase FKBP8 isoform X1 [Enhydra lutris kenyoni]
(149) NP_001272500 peptidyl-prolyl cis-trans isomerase FKBP8 [Capra hircus].
(150) XP_012916133 PREDICTED: peptidyl-prolyl cis-trans isomerase FKBP8 isoform X2 [Mustela puto
(151) XP_011365085 peptidyl-prolyl cis-trans isomerase FKBP8 isoform X2 [Pteropus vampyrus].
(152) XP_006885938 PREDICTED: peptidyl-prolyl cis-trans isomerase FKBP8 isoform X1 [Elephantulus
(153) XP_004423006 PREDICTED: peptidyl-prolyl cis-trans isomerase FKBP8 isoform X2 [Ceratotheriu
(154) XP_012413338 peptidyl-prolyl cis-trans isomerase FKBP8 isoform X2 [Trichechus manatus lati
(155) XP_010591524 peptidyl-prolyl cis-trans isomerase FKBP8 isoform X2 [Loxodonta africana].
(156) XP_008048662 peptidyl-prolyl cis-trans isomerase FKBP8 isoform X1 [Carlito syrichta].
(157) XP_015987916 PREDICTED: peptidyl-prolyl cis-trans isomerase FKBP8 isoform X1 [Rousettus ae
(158) XP_004666075 PREDICTED: peptidyl-prolyl cis-trans isomerase FKBP8 isoform X2 [Jaculus jacu
(159) XP_019497624 PREDICTED: peptidyl-prolyl cis-trans isomerase FKBP8 isoform X2 [Hipposideros
(160) XP_004649071 LOW QUALITY PROTEIN: peptidyl-prolyl cis-trans isomerase FKBP8 [Octodon degus
(161) APD32922 Fkbp8 [Castor fiber].
(162) XP_013362738 PREDICTED: peptidyl-prolyl cis-trans isomerase FKBP8 isoform X1 [Chinchilla l
(163) XP_012509480 PREDICTED: peptidyl-prolyl cis-trans isomerase FKBP8 [Propithecus coquereli].
(164) NP_034353 peptidyl-prolyl cis-trans isomerase FKBP8 isoform b [Mus musculus].
(165) XP_021074830 peptidyl-prolyl cis-trans isomerase FKBP8 isoform X2 [Mus pahari].
(166) XP_006876370 PREDICTED: peptidyl-prolyl cis-trans isomerase FKBP8 isoform X1 [Chrysochlori

```
(167)  XP_021493691     peptidyl-prolyl cis-trans isomerase FKBP8 isoform X1 [Meriones unguiculatus].
(168)  XP_006904374     peptidyl-prolyl cis-trans isomerase FKBP8 isoform X1 [Pteropus alecto].
(169)  XP_006989222     PREDICTED: peptidyl-prolyl cis-trans isomerase FKBP8 isoform X2 [Peromyscus m
(170)  XP_004423005     PREDICTED: peptidyl-prolyl cis-trans isomerase FKBP8 isoform X3 [Ceratotheriu
(171)  XP_006885939     PREDICTED: peptidyl-prolyl cis-trans isomerase FKBP8 isoform X2 [Elephantulus
(172)  XP_004666073     PREDICTED: peptidyl-prolyl cis-trans isomerase FKBP8 isoform X1 [Jaculus jacu
(173)  ELK19193         FK506-binding protein 8 [Pteropus alecto].
(174)  XP_003499206     PREDICTED: peptidyl-prolyl cis-trans isomerase FKBP8 isoform X2 [Cricetulus g
(175)  XP_014637207     PREDICTED: peptidyl-prolyl cis-trans isomerase FKBP8 isoform X1 [Ceratotheriu
(176)  XP_012790031     PREDICTED: peptidyl-prolyl cis-trans isomerase FKBP8 isoform X1 [Sorex araneu
(177)  XP_003413066     peptidyl-prolyl cis-trans isomerase FKBP8 isoform X1 [Loxodonta africana].
(178)  XP_004384434     peptidyl-prolyl cis-trans isomerase FKBP8 isoform X1 [Trichechus manatus lati
(179)  XP_015987917     PREDICTED: peptidyl-prolyl cis-trans isomerase FKBP8 isoform X2 [Rousettus ae
(180)  XP_021078721     peptidyl-prolyl cis-trans isomerase FKBP8 [Mesocricetus auratus].
(181)  5MGX_E           Chain E, Peptidyl-prolyl Cis-trans Isomerase Fkbp8.
(182)  XP_017168055     PREDICTED: peptidyl-prolyl cis-trans isomerase FKBP8 isoform X2 [Mus musculus
(183)  NP_001032257     peptidyl-prolyl cis-trans isomerase FKBP8 [Rattus norvegicus].
(184)  XP_004595597     PREDICTED: peptidyl-prolyl cis-trans isomerase FKBP8 isoform X2 [Ochotona pri
(185)  XP_017529992     PREDICTED: peptidyl-prolyl cis-trans isomerase FKBP8 isoform X2 [Manis javani
(186)  XP_005370624     PREDICTED: peptidyl-prolyl cis-trans isomerase FKBP8 isoform X2 [Microtus och
(187)  NP_001104536     peptidyl-prolyl cis-trans isomerase FKBP8 isoform a [Mus musculus].
(188)  XP_021074828     peptidyl-prolyl cis-trans isomerase FKBP8 isoform X1 [Mus pahari].
(189)  XP_012664880     peptidyl-prolyl cis-trans isomerase FKBP8 isoform X2 [Otolemur garnettii].
(190)  XP_006989221     PREDICTED: peptidyl-prolyl cis-trans isomerase FKBP8 isoform X1 [Peromyscus m
(191)  XP_007635977     PREDICTED: peptidyl-prolyl cis-trans isomerase FKBP8 isoform X1 [Cricetulus g
(192)  XP_004616706     PREDICTED: peptidyl-prolyl cis-trans isomerase FKBP8 isoform X2 [Sorex araneu
(193)  XP_012664879     peptidyl-prolyl cis-trans isomerase FKBP8 isoform X1 [Otolemur garnettii].
(194)  XP_004705311     PREDICTED: peptidyl-prolyl cis-trans isomerase FKBP8 isoform X2 [Echinops tel
(195)  XP_017168054     PREDICTED: peptidyl-prolyl cis-trans isomerase FKBP8 isoform X1 [Mus musculus
(196)  XP_021026690     peptidyl-prolyl cis-trans isomerase FKBP8 isoform X2 [Mus caroli].
(197)  XP_004595596     PREDICTED: peptidyl-prolyl cis-trans isomerase FKBP8 isoform X1 [Ochotona pri
(198)  XP_017529990     PREDICTED: peptidyl-prolyl cis-trans isomerase FKBP8 isoform X1 [Manis javani
(199)  XP_005370622     PREDICTED: peptidyl-prolyl cis-trans isomerase FKBP8 isoform X1 [Microtus och
(200)  XP_012883833     PREDICTED: peptidyl-prolyl cis-trans isomerase FKBP8 isoform X2 [Dipodomys or
(201)  XP_021026691     peptidyl-prolyl cis-trans isomerase FKBP8 isoform X3 [Mus caroli].
(202)  XP_010849455     PREDICTED: LOW QUALITY PROTEIN: peptidyl-prolyl cis-trans isomerase FKBP8-lik
(203)  XP_010604777     PREDICTED: peptidyl-prolyl cis-trans isomerase FKBP8 [Fukomys damarensis].
(204)  XP_004705310     PREDICTED: peptidyl-prolyl cis-trans isomerase FKBP8 isoform X1 [Echinops tel
(205)  XP_021026689     peptidyl-prolyl cis-trans isomerase FKBP8 isoform X1 [Mus caroli].
(206)  XP_005898381     PREDICTED: peptidyl-prolyl cis-trans isomerase FKBP8 [Bos mutus].
(207)  XP_012883832     PREDICTED: peptidyl-prolyl cis-trans isomerase FKBP8 isoform X1 [Dipodomys or
(208)  XP_024622994     peptidyl-prolyl cis-trans isomerase FKBP8 isoform X4 [Neophocaena asiaeorient
```

(209)	XP_022414294	peptidyl-prolyl cis-trans isomerase FKBP8 isoform X2 [Delphinapterus leucas].
(210)	XP_007116996	peptidyl-prolyl cis-trans isomerase FKBP8 isoform X3 [Physeter catodon].
(211)	XP_024622993	peptidyl-prolyl cis-trans isomerase FKBP8 isoform X3 [Neophocaena asiaeorient
(212)	XP_022414293	peptidyl-prolyl cis-trans isomerase FKBP8 isoform X1 [Delphinapterus leucas].
(213)	XP_023055022	peptidyl-prolyl cis-trans isomerase FKBP8, partial [Piliocolobus tephrosceles
(214)	ELR54589	Peptidyl-prolyl cis-trans isomerase FKBP8 [Bos mutus].
(215)	XP_010845609	PREDICTED: peptidyl-prolyl cis-trans isomerase FKBP8 [Bison bison bison].
(216)	XP_021538869	peptidyl-prolyl cis-trans isomerase FKBP8 [Neomonachus schauinslandi].
(217)	KFO22602	Peptidyl-prolyl cis-trans isomerase FKBP8 [Fukomys damarensis].
(218)	2MF9_A	Chain A, Peptidyl-prolyl cis-trans isomerase FKBP8.
(219)	PNI50452	FKBP8 isoform 11, partial [Pan troglodytes].
(220)	PNJ28404	FKBP8 isoform 11, partial [Pongo abelii].
(221)	XP_007533227	PREDICTED: peptidyl-prolyl cis-trans isomerase FKBP8 [Erinaceus europaeus].
(222)	XP_020864537	peptidyl-prolyl cis-trans isomerase FKBP8 [Phascolarctos cinereus].
(223)	XP_012397649	peptidyl-prolyl cis-trans isomerase FKBP8 [Sarcophilus harrisii].
(224)	XP_001369403	PREDICTED: peptidyl-prolyl cis-trans isomerase FKBP8 [Monodelphis domestica].
(225)	2F2D_A	Chain A, kDa FK-506 binding protein homolog, FKBP38.
(226)	2D9F_A	Chain A, FK506-binding protein 8 variant.
(227)	XP_015668943	PREDICTED: peptidyl-prolyl cis-trans isomerase FKBP8 isoform X2 [Protobothrop
(228)	2AWG_A	Chain A, Structure Of The Ppiase Domain Of The Human Fk506-Binding Protein 8.
(229)	XP_015668942	PREDICTED: peptidyl-prolyl cis-trans isomerase FKBP8 isoform X1 [Protobothrop
(230)	XP_025032464	peptidyl-prolyl cis-trans isomerase FKBP8 isoform X2 [Python bivittatus].
(231)	XP_007443923	peptidyl-prolyl cis-trans isomerase FKBP8 isoform X1 [Python bivittatus].
(232)	KFV73372	Peptidyl-prolyl cis-trans isomerase FKBP8, partial [Struthio camelus australi
(233)	OXB56359	hypothetical protein ASZ78_001767 [Callipepla squamata].
(234)	XP_009675861	PREDICTED: peptidyl-prolyl cis-trans isomerase FKBP8 [Struthio camelus austra
(235)	OXB73131	hypothetical protein H355_012056 [Colinus virginianus].
(236)	XP_010723402	PREDICTED: peptidyl-prolyl cis-trans isomerase FKBP8, partial [Meleagris gall
(237)	KFV67807	Peptidyl-prolyl cis-trans isomerase FKBP8, partial [Picoides pubescens].
(238)	XP_010304001	PREDICTED: peptidyl-prolyl cis-trans isomerase FKBP8, partial [Balearica regu
(239)	NP_001241668	peptidyl-prolyl cis-trans isomerase FKBP8 [Gallus gallus].
(240)	KFO03599	Peptidyl-prolyl cis-trans isomerase FKBP8, partial [Balearica regulorum gibbe
(241)	XP_021234495	peptidyl-prolyl cis-trans isomerase FKBP8 isoform X1 [Numida meleagris].
(242)	POI29828	hypothetical protein CIB84_006422 [Bambusicola thoracicus].
(243)	XP_021234496	peptidyl-prolyl cis-trans isomerase FKBP8 isoform X2 [Numida meleagris].
(244)	XP_023963943	peptidyl-prolyl cis-trans isomerase FKBP8 [Chrysemys picta bellii].
(245)	XP_014793985	PREDICTED: peptidyl-prolyl cis-trans isomerase FKBP8 [Calidris pugnax].
(246)	XP_009899401	PREDICTED: peptidyl-prolyl cis-trans isomerase FKBP8 [Picoides pubescens].
(247)	XP_015742210	PREDICTED: peptidyl-prolyl cis-trans isomerase FKBP8 isoform X2 [Coturnix jap
(248)	XP_009641057	PREDICTED: peptidyl-prolyl cis-trans isomerase FKBP8, partial [Egretta garzet
(249)	KFP19261	Peptidyl-prolyl cis-trans isomerase FKBP8, partial [Egretta garzetta].
(250)	XP_024065593	peptidyl-prolyl cis-trans isomerase FKBP8-like [Terrapene mexicana triunguis]

(251)	XP_011594878	PREDICTED: peptidyl-prolyl cis-trans isomerase FKBP8 [Aquila chrysaetos canad
(252)	XP_014742726	PREDICTED: peptidyl-prolyl cis-trans isomerase FKBP8 isoform X2 [Sturnus vulg
(253)	XP_015742208	PREDICTED: peptidyl-prolyl cis-trans isomerase FKBP8 isoform X1 [Coturnix jap
(254)	XP_019369180	PREDICTED: peptidyl-prolyl cis-trans isomerase FKBP8 [Gavialis gangeticus].
(255)	XP_010561556	PREDICTED: peptidyl-prolyl cis-trans isomerase FKBP8 [Haliaeetus leucocephalu
(256)	XP_008638175	PREDICTED: peptidyl-prolyl cis-trans isomerase FKBP8, partial [Corvus brachyr
(257)	XP_002195923	PREDICTED: peptidyl-prolyl cis-trans isomerase FKBP8 [Taeniopygia guttata].
(258)	XP_015266773	PREDICTED: peptidyl-prolyl cis-trans isomerase FKBP8 [Gekko japonicus].
(259)	XP_005505599	peptidyl-prolyl cis-trans isomerase FKBP8, partial [Columba livia].
(260)	XP_014115778	PREDICTED: peptidyl-prolyl cis-trans isomerase FKBP8 [Pseudopodoces humilis].
(261)	XP_014742725	PREDICTED: peptidyl-prolyl cis-trans isomerase FKBP8 isoform X1 [Sturnus vulg
(262)	XP_018780521	PREDICTED: LOW QUALITY PROTEIN: peptidyl-prolyl cis-trans isomerase FKBP8 [Se
(263)	XP_005235043	PREDICTED: peptidyl-prolyl cis-trans isomerase FKBP8 isoform X2 [Falco peregr
(264)	OPJ86694	peptidyl-prolyl cis-trans isomerase FKBP8 [Patagioenas fasciata monilis].
(265)	XP_019341692	PREDICTED: peptidyl-prolyl cis-trans isomerase FKBP8 isoform X2 [Alligator mi
(266)	XP_021400798	peptidyl-prolyl cis-trans isomerase FKBP8 [Lonchura striata domestica].
(267)	XP_006028906	peptidyl-prolyl cis-trans isomerase FKBP8 [Alligator sinensis].
(268)	KFQ97904	Peptidyl-prolyl cis-trans isomerase FKBP8, partial [Nipponia nippon].
(269)	XP_015507158	PREDICTED: peptidyl-prolyl cis-trans isomerase FKBP8 isoform X2 [Parus major]
(270)	KFW12711	Peptidyl-prolyl cis-trans isomerase FKBP8, partial [Eurypyga helias].
(271)	XP_006271082	PREDICTED: peptidyl-prolyl cis-trans isomerase FKBP8 isoform X1 [Alligator mi
(272)	KFO81655	Peptidyl-prolyl cis-trans isomerase FKBP8, partial [Cuculus canorus].
(273)	XP_007072098	PREDICTED: peptidyl-prolyl cis-trans isomerase FKBP8, partial [Chelonia mydas
(274)	XP_020669929	peptidyl-prolyl cis-trans isomerase FKBP8 isoform X1 [Pogona vitticeps].
(275)	XP_009464539	PREDICTED: peptidyl-prolyl cis-trans isomerase FKBP8 [Nipponia nippon].
(276)	XP_010158039	PREDICTED: peptidyl-prolyl cis-trans isomerase FKBP8, partial [Eurypyga helia
(277)	XP_019149030	peptidyl-prolyl cis-trans isomerase FKBP8, partial [Corvus cornix cornix].
(278)	XP_020669930	peptidyl-prolyl cis-trans isomerase FKBP8 isoform X2 [Pogona vitticeps].
(279)	XP_019393900	PREDICTED: peptidyl-prolyl cis-trans isomerase FKBP8 [Crocodylus porosus].
(280)	EMP24085	Peptidyl-prolyl cis-trans isomerase FKBP8 [Chelonia mydas].
(281)	XP_009566597	PREDICTED: peptidyl-prolyl cis-trans isomerase FKBP8 [Cuculus canorus].
(282)	KFW65305	Peptidyl-prolyl cis-trans isomerase FKBP8, partial [Pygoscelis adeliae].
(283)	XP_015507157	PREDICTED: peptidyl-prolyl cis-trans isomerase FKBP8 isoform X1 [Parus major]
(284)	XP_013799130	PREDICTED: peptidyl-prolyl cis-trans isomerase FKBP8 [Apteryx australis mante
(285)	XP_014433658	peptidyl-prolyl cis-trans isomerase FKBP8 [Pelodiscus sinensis].
(286)	XP_009319696	PREDICTED: peptidyl-prolyl cis-trans isomerase FKBP8, partial [Pygoscelis ade
(287)	KFQ36923	Peptidyl-prolyl cis-trans isomerase FKBP8, partial [Mesitornis unicolor].
(288)	XP_005060257	PREDICTED: peptidyl-prolyl cis-trans isomerase FKBP8 [Ficedula albicollis].
(289)	KFU87694	Peptidyl-prolyl cis-trans isomerase FKBP8, partial [Chaetura pelagica].
(290)	XP_005139681	PREDICTED: peptidyl-prolyl cis-trans isomerase FKBP8 [Melopsittacus undulatus
(291)	XP_009993397	PREDICTED: peptidyl-prolyl cis-trans isomerase FKBP8 [Chaetura pelagica].
(292)	AAH84415	LOC495188 protein, partial [Xenopus laevis].

(293) KQL50954 peptidyl-prolyl cis-trans isomerase FKBP8 [Amazona aestiva].
(294) NP_001088346 FK506 binding protein 8 L homeolog [Xenopus laevis].
(295) XP_013913386 PREDICTED: peptidyl-prolyl cis-trans isomerase FKBP8 [Thamnophis sirtalis].
(296) KFQ13009 Peptidyl-prolyl cis-trans isomerase FKBP8, partial [Leptosomus discolor].
(297) KFQ75436 Peptidyl-prolyl cis-trans isomerase FKBP8, partial [Phoenicopterus ruber rube
(298) KFP56346 Peptidyl-prolyl cis-trans isomerase FKBP8, partial [Cathartes aura].
(299) KFM07869 Peptidyl-prolyl cis-trans isomerase FKBP8, partial [Aptenodytes forsteri].
(300) XP_018096952 PREDICTED: peptidyl-prolyl cis-trans isomerase FKBP8-like [Xenopus laevis].
(301) KFP65829 Peptidyl-prolyl cis-trans isomerase FKBP8, partial [Cariama cristata].
(302) XP_023803625 peptidyl-prolyl cis-trans isomerase FKBP8, partial [Cyanistes caeruleus].
(303) XP_009951725 PREDICTED: peptidyl-prolyl cis-trans isomerase FKBP8, partial [Leptosomus dis
(304) NP_001120570 peptidyl-prolyl cis-trans isomerase FKBP8 [Xenopus tropicalis].
(305) KFP23912 Peptidyl-prolyl cis-trans isomerase FKBP8, partial [Colius striatus].
(306) XP_010073720 PREDICTED: peptidyl-prolyl cis-trans isomerase FKBP8, partial [Pterocles gutt
(307) KGL86304 Peptidyl-prolyl cis-trans isomerase FKBP8, partial [Charadrius vociferus].
(308) KFV08597 Peptidyl-prolyl cis-trans isomerase FKBP8, partial [Pterocles gutturalis].
(309) KFW76752 Peptidyl-prolyl cis-trans isomerase FKBP8, partial [Manacus vitellinus].
(310) XP_009700019 PREDICTED: peptidyl-prolyl cis-trans isomerase FKBP8, partial [Cariama crista
(311) XP_010208130 PREDICTED: peptidyl-prolyl cis-trans isomerase FKBP8, partial [Colius striatu
(312) KFQ81502 Peptidyl-prolyl cis-trans isomerase FKBP8, partial [Phaethon lepturus].
(313) XP_017938939 PREDICTED: LOW QUALITY PROTEIN: peptidyl-prolyl cis-trans isomerase FKBP8, pa
(314) KFP70760 Peptidyl-prolyl cis-trans isomerase FKBP8, partial [Acanthisitta chloris].
(315) XP_009880959 PREDICTED: peptidyl-prolyl cis-trans isomerase FKBP8 [Charadrius vociferus].
(316) XP_010292010 PREDICTED: peptidyl-prolyl cis-trans isomerase FKBP8, partial [Phaethon leptu
(317) XP_009076312 PREDICTED: peptidyl-prolyl cis-trans isomerase FKBP8, partial [Acanthisitta c
(318) XP_018412986 PREDICTED: peptidyl-prolyl cis-trans isomerase FKBP8 [Nanorana parkeri].
(319) XP_022525503 peptidyl-prolyl cis-trans isomerase FKBP8 [Astyanax mexicanus].
(320) XP_021459512 peptidyl-prolyl cis-trans isomerase FKBP8-like isoform X2 [Oncorhynchus mykis
(321) XP_021459510 peptidyl-prolyl cis-trans isomerase FKBP8-like isoform X1 [Oncorhynchus mykis
(322) XP_024277334 peptidyl-prolyl cis-trans isomerase FKBP8-like isoform X4 [Oncorhynchus tshaw
(323) XP_024277330 peptidyl-prolyl cis-trans isomerase FKBP8-like isoform X1 [Oncorhynchus tshaw
(324) XP_024277333 peptidyl-prolyl cis-trans isomerase FKBP8-like isoform X3 [Oncorhynchus tshaw
(325) XP_009512288 PREDICTED: peptidyl-prolyl cis-trans isomerase FKBP8, partial [Phalacrocorax
(326) XP_020354512 peptidyl-prolyl cis-trans isomerase FKBP8-like isoform X1 [Oncorhynchus kisut
(327) KFZ50883 Peptidyl-prolyl cis-trans isomerase FKBP8, partial [Podiceps cristatus].
(328) KFW81721 Peptidyl-prolyl cis-trans isomerase FKBP8, partial [Phalacrocorax carbo].
(329) CDQ57258 unnamed protein product [Oncorhynchus mykiss].
(330) XP_020354513 peptidyl-prolyl cis-trans isomerase FKBP8-like isoform X2 [Oncorhynchus kisut
(331) XP_020487811 peptidyl-prolyl cis-trans isomerase FKBP8 [Labrus bergylta].
(332) XP_024277331 peptidyl-prolyl cis-trans isomerase FKBP8-like isoform X2 [Oncorhynchus tshaw
(333) XP_014071869 PREDICTED: peptidyl-prolyl cis-trans isomerase FKBP8-like isoform X1 [Salmo s
(334) XP_011472153 peptidyl-prolyl cis-trans isomerase FKBP8 isoform X2 [Oryzias latipes].

(335)	XP_014071870	PREDICTED: peptidyl-prolyl cis-trans isomerase FKBP8-like isoform X2 [Salmo s
(336)	XP_024114895	peptidyl-prolyl cis-trans isomerase FKBP8 isoform X1 [Oryzias melastigma].
(337)	XP_024114896	peptidyl-prolyl cis-trans isomerase FKBP8 isoform X2 [Oryzias melastigma].
(338)	ETE64390	Peptidyl-prolyl cis-trans isomerase FKBP8, partial [Ophiophagus hannah].
(339)	XP_023809890	peptidyl-prolyl cis-trans isomerase FKBP8 isoform X1 [Oryzias latipes].
(340)	XP_008499209	PREDICTED: peptidyl-prolyl cis-trans isomerase FKBP8 [Calypte anna].
(341)	KFP06384	Peptidyl-prolyl cis-trans isomerase FKBP8, partial [Calypte anna].
(342)	XP_023859426	peptidyl-prolyl cis-trans isomerase FKBP8 isoform X3 [Salvelinus alpinus].
(343)	XP_023859424	peptidyl-prolyl cis-trans isomerase FKBP8 isoform X2 [Salvelinus alpinus].
(344)	XP_023859423	peptidyl-prolyl cis-trans isomerase FKBP8 isoform X1 [Salvelinus alpinus].
(345)	XP_021454666	peptidyl-prolyl cis-trans isomerase FKBP8-like [Oncorhynchus mykiss].
(346)	XP_017572299	PREDICTED: peptidyl-prolyl cis-trans isomerase FKBP8 [Pygocentrus nattereri].
(347)	CDQ97962	unnamed protein product [Oncorhynchus mykiss].
(348)	XP_023854736	peptidyl-prolyl cis-trans isomerase FKBP8 isoform X2 [Salvelinus alpinus].
(349)	XP_018920592	PREDICTED: peptidyl-prolyl cis-trans isomerase FKBP8 [Cyprinus carpio].
(350)	NP_001133417	peptidyl-prolyl cis-trans isomerase FKBP8 [Salmo salar].
(351)	XP_023854735	peptidyl-prolyl cis-trans isomerase FKBP8 isoform X1 [Salvelinus alpinus].
(352)	XP_024258320	peptidyl-prolyl cis-trans isomerase FKBP8-like [Oncorhynchus tshawytscha].
(353)	AAI52651	FK506 binding protein 8 [Danio rerio].
(354)	XP_020348060	peptidyl-prolyl cis-trans isomerase FKBP8-like [Oncorhynchus kisutch].
(355)	NP_957178	peptidyl-prolyl cis-trans isomerase FKBP8 [Danio rerio].
(356)	XP_005932512	PREDICTED: peptidyl-prolyl cis-trans isomerase FKBP8 [Haplochromis burtoni].
(357)	XP_013860095	PREDICTED: peptidyl-prolyl cis-trans isomerase FKBP8 [Austrofundulus limnaeus
(358)	PWA20923	hypothetical protein CCH79_00007271, partial [Gambusia affinis].
(359)	XP_005727139	PREDICTED: peptidyl-prolyl cis-trans isomerase FKBP8 [Pundamilia nyererei].
(360)	XP_006790401	PREDICTED: peptidyl-prolyl cis-trans isomerase FKBP8-like [Neolamprologus bri
(361)	XP_019207270	PREDICTED: peptidyl-prolyl cis-trans isomerase FKBP8 [Oreochromis niloticus].
(362)	XP_024654530	peptidyl-prolyl cis-trans isomerase FKBP8 [Maylandia zebra].
(363)	XP_007897075	PREDICTED: peptidyl-prolyl cis-trans isomerase FKBP8 [Callorhinchus milii].
(364)	XP_010870009	PREDICTED: peptidyl-prolyl cis-trans isomerase FKBP8 isoform X2 [Esox lucius]
(365)	XP_022615368	peptidyl-prolyl cis-trans isomerase FKBP8 [Seriola dumerili].
(366)	XP_010870008	PREDICTED: peptidyl-prolyl cis-trans isomerase FKBP8 isoform X1 [Esox lucius]
(367)	XP_005795669	peptidyl-prolyl cis-trans isomerase FKBP8 [Xiphophorus maculatus].
(368)	XP_020391166	peptidyl-prolyl cis-trans isomerase FKBP8 isoform X2 [Rhincodon typus].
(369)	AWP11099	putative peptidyl-prolyl cis-trans isomerase FKBP8 [Scophthalmus maximus].
(370)	XP_017333233	PREDICTED: peptidyl-prolyl cis-trans isomerase FKBP8 [Ictalurus punctatus].
(371)	KTF74275	hypothetical protein cypCar_00030593, partial [Cyprinus carpio].
(372)	XP_017262938	peptidyl-prolyl cis-trans isomerase FKBP8 [Kryptolebias marmoratus].
(373)	XP_023263872	peptidyl-prolyl cis-trans isomerase FKBP8 [Seriola lalandi dorsalis].
(374)	XP_020391165	peptidyl-prolyl cis-trans isomerase FKBP8 isoform X1 [Rhincodon typus].
(375)	XP_016327233	PREDICTED: peptidyl-prolyl cis-trans isomerase FKBP8-like [Sinocyclocheilus a
(376)	XP_016138463	PREDICTED: peptidyl-prolyl cis-trans isomerase FKBP8 [Sinocyclocheilus graham

```
(377)  XP_016356127     PREDICTED: peptidyl-prolyl cis-trans isomerase FKBP8-like [Sinocyclocheilus a
(378)  XP_007577281     PREDICTED: peptidyl-prolyl cis-trans isomerase FKBP8 [Poecilia formosa].
(379)  XP_008405333     PREDICTED: peptidyl-prolyl cis-trans isomerase FKBP8 [Poecilia reticulata].
(380)  XP_012715520     peptidyl-prolyl cis-trans isomerase FKBP8 [Fundulus heteroclitus].
(381)  XP_012695070     PREDICTED: peptidyl-prolyl cis-trans isomerase FKBP8 [Clupea harengus].
(382)  XP_020795782     peptidyl-prolyl cis-trans isomerase FKBP8 [Boleophthalmus pectinirostris].
(383)  XP_016382508     PREDICTED: peptidyl-prolyl cis-trans isomerase FKBP8-like [Sinocyclocheilus r
(384)  XP_018522913     PREDICTED: peptidyl-prolyl cis-trans isomerase FKBP8 [Lates calcarifer].
(385)  XP_008305299     peptidyl-prolyl cis-trans isomerase FKBP8 isoform X2 [Cynoglossus semilaevis]
(386)  XP_015238114     PREDICTED: peptidyl-prolyl cis-trans isomerase FKBP8 [Cyprinodon variegatus].
(387)  XP_018583793     PREDICTED: peptidyl-prolyl cis-trans isomerase FKBP8 [Scleropages formosus].
(388)  XP_016886204     peptidyl-prolyl cis-trans isomerase FKBP8 isoform X1 [Cynoglossus semilaevis]
(389)  XP_019752339     PREDICTED: peptidyl-prolyl cis-trans isomerase FKBP8 [Hippocampus comes].
(390)  XP_010728467     PREDICTED: peptidyl-prolyl cis-trans isomerase FKBP8 [Larimichthys crocea].
(391)  KKF19855         Peptidyl-prolyl cis-trans isomerase FKBP8 [Larimichthys crocea].
(392)  XP_015827013     PREDICTED: peptidyl-prolyl cis-trans isomerase FKBP8 [Nothobranchius furzeri]
(393)  XP_022061518     peptidyl-prolyl cis-trans isomerase FKBP8 [Acanthochromis polyacanthus].
(394)  XP_019936960     PREDICTED: peptidyl-prolyl cis-trans isomerase FKBP8 [Paralichthys olivaceus]
(395)  XP_003974097     PREDICTED: peptidyl-prolyl cis-trans isomerase FKBP8 [Takifugu rubripes].
(396)  XP_023699813     peptidyl-prolyl cis-trans isomerase FKBP8 isoform X2 [Paramormyrops kingsleya
(397)  XP_023699812     peptidyl-prolyl cis-trans isomerase FKBP8 isoform X1 [Paramormyrops kingsleya
(398)  XP_020478047     peptidyl-prolyl cis-trans isomerase FKBP8 isoform X3 [Monopterus albus].
(399)  XP_015220721     PREDICTED: peptidyl-prolyl cis-trans isomerase FKBP8 [Lepisosteus oculatus].
(400)  XP_023123034     peptidyl-prolyl cis-trans isomerase FKBP8 [Amphiprion ocellaris].
(401)  XP_020478046     peptidyl-prolyl cis-trans isomerase FKBP8 isoform X2 [Monopterus albus].
(402)  XP_020478045     peptidyl-prolyl cis-trans isomerase FKBP8 isoform X1 [Monopterus albus].
(403)  XP_008275730     PREDICTED: peptidyl-prolyl cis-trans isomerase FKBP8 [Stegastes partitus].
(404)  XP_010770232     PREDICTED: peptidyl-prolyl cis-trans isomerase FKBP8 [Notothenia coriiceps].
(405)  XP_002601637     hypothetical protein BRAFLDRAFT_85785 [Branchiostoma floridae].
(406)  XP_019619553     PREDICTED: LOW QUALITY PROTEIN: peptidyl-prolyl cis-trans isomerase FKBP8-lik
(407)  XP_006814126     PREDICTED: peptidyl-prolyl cis-trans isomerase FKBP8-like [Saccoglossus kowal
(408)  XP_006629043     PREDICTED: peptidyl-prolyl cis-trans isomerase FKBP8-like [Lepisosteus oculat
(409)  PVD20167         hypothetical protein C0Q70_20661 [Pomacea canaliculata].
(410)  XP_023841292     peptidyl-prolyl cis-trans isomerase FKBP8-like [Salvelinus alpinus].
(411)  XP_008410571     PREDICTED: peptidyl-prolyl cis-trans isomerase FKBP8-like isoform X2 [Poecili
(412)  XP_008410568     PREDICTED: peptidyl-prolyl cis-trans isomerase FKBP8-like isoform X1 [Poecili
(413)  XP_022329628     peptidyl-prolyl cis-trans isomerase FKBP8-like [Crassostrea virginica].
(414)  XP_014897626     PREDICTED: peptidyl-prolyl cis-trans isomerase FKBP8-like isoform X2 [Poecili
(415)  XP_014848641     PREDICTED: peptidyl-prolyl cis-trans isomerase FKBP8-like isoform X2 [Poecili
(416)  XP_014848639     PREDICTED: peptidyl-prolyl cis-trans isomerase FKBP8-like isoform X1 [Poecili
(417)  XP_007566466     PREDICTED: peptidyl-prolyl cis-trans isomerase FKBP8-like isoform X2 [Poecili
(418)  XP_023180976     peptidyl-prolyl cis-trans isomerase FKBP8-like isoform X2 [Xiphophorus macula
```

(419)	KMQ91381	peptidyl-prolyl cis-trans isomerase fkbp8-like protein [Lasius niger].
(420)	XP_007566465	PREDICTED: peptidyl-prolyl cis-trans isomerase FKBP8-like isoform X1 [Poecili
(421)	XP_014897625	PREDICTED: peptidyl-prolyl cis-trans isomerase FKBP8-like isoform X1 [Poecili
(422)	XP_015254615	PREDICTED: peptidyl-prolyl cis-trans isomerase FKBP8-like isoform X2 [Cyprino
(423)	XP_005798327	peptidyl-prolyl cis-trans isomerase FKBP8-like isoform X1 [Xiphophorus macula
(424)	XP_015254612	PREDICTED: peptidyl-prolyl cis-trans isomerase FKBP8-like isoform X1 [Cyprino
(425)	XP_013980328	PREDICTED: peptidyl-prolyl cis-trans isomerase FKBP8-like [Salmo salar].
(426)	XP_009061784	hypothetical protein LOTGIDRAFT_127555 [Lottia gigantea].
(427)	XP_022106769	peptidyl-prolyl cis-trans isomerase FKBP8-like [Acanthaster planci].
(428)	XP_020625955	peptidyl-prolyl cis-trans isomerase FKBP8-like isoform X1 [Orbicella faveolat
(429)	XP_020625956	peptidyl-prolyl cis-trans isomerase FKBP8-like isoform X2 [Orbicella faveolat
(430)	XP_010732628	PREDICTED: peptidyl-prolyl cis-trans isomerase FKBP8 [Larimichthys crocea].
(431)	XP_015728921	PREDICTED: peptidyl-prolyl cis-trans isomerase FKBP8-like [Coturnix japonica]
(432)	XP_003440659	PREDICTED: peptidyl-prolyl cis-trans isomerase FKBP8 [Oreochromis niloticus].
(433)	XP_014004032	PREDICTED: peptidyl-prolyl cis-trans isomerase FKBP8-like [Salmo salar].
(434)	XP_010899928	PREDICTED: peptidyl-prolyl cis-trans isomerase FKBP8 [Esox lucius].
(435)	XP_020336380	peptidyl-prolyl cis-trans isomerase FKBP8-like isoform X2 [Oncorhynchus kisut
(436)	XP_022330049	peptidyl-prolyl cis-trans isomerase FKBP8-like isoform X2 [Crassostrea virgin
(437)	PIK48936	putative peptidyl-prolyl cis-trans isomerase FKBP8-like [Apostichopus japonic
(438)	XP_020336379	peptidyl-prolyl cis-trans isomerase FKBP8-like isoform X1 [Oncorhynchus kisut
(439)	XP_022330048	peptidyl-prolyl cis-trans isomerase FKBP8-like isoform X1 [Crassostrea virgin
(440)	XP_007907980	PREDICTED: peptidyl-prolyl cis-trans isomerase FKBP8-like [Callorhinchus mili
(441)	XP_013859000	PREDICTED: peptidyl-prolyl cis-trans isomerase FKBP8-like [Austrofundulus lim
(442)	XP_019737313	PREDICTED: peptidyl-prolyl cis-trans isomerase FKBP8-like [Hippocampus comes]
(443)	XP_021427237	peptidyl-prolyl cis-trans isomerase FKBP8-like [Oncorhynchus mykiss].
(444)	XP_023827235	peptidyl-prolyl cis-trans isomerase FKBP8 isoform X2 [Salvelinus alpinus].
(445)	XP_023827230	peptidyl-prolyl cis-trans isomerase FKBP8 isoform X1 [Salvelinus alpinus].
(446)	XP_008309233	peptidyl-prolyl cis-trans isomerase FKBP8 isoform X1 [Cynoglossus semilaevis]
(447)	XP_024269308	peptidyl-prolyl cis-trans isomerase FKBP8-like isoform X4 [Oncorhynchus tshaw
(448)	XP_002932306	PREDICTED: peptidyl-prolyl cis-trans isomerase FKBP8-like [Xenopus tropicalis
(449)	XP_022785584	peptidyl-prolyl cis-trans isomerase FKBP8-like [Stylophora pistillata].
(450)	XP_008309234	peptidyl-prolyl cis-trans isomerase FKBP8 isoform X2 [Cynoglossus semilaevis]
(451)	XP_013386268	peptidyl-prolyl cis-trans isomerase FKBP8 [Lingula anatina].
(452)	XP_005928731	PREDICTED: peptidyl-prolyl cis-trans isomerase FKBP8-like [Haplochromis burto
(453)	XP_002119960	PREDICTED: peptidyl-prolyl cis-trans isomerase FKBP8 [Ciona intestinalis].
(454)	XP_024235293	peptidyl-prolyl cis-trans isomerase FKBP8-like [Oncorhynchus tshawytscha].
(455)	XP_005729996	PREDICTED: peptidyl-prolyl cis-trans isomerase FKBP8-like [Pundamilia nyerere
(456)	XP_018619414	PREDICTED: peptidyl-prolyl cis-trans isomerase FKBP8-like [Scleropages formos
(457)	XP_012796312	Peptidyl-prolyl cis-trans isomerase FKBP8 [Schistosoma haematobium].
(458)	XP_004553258	peptidyl-prolyl cis-trans isomerase FKBP8 [Maylandia zebra].
(459)	CDQ72337	unnamed protein product [Oncorhynchus mykiss].
(460)	XP_024269303	peptidyl-prolyl cis-trans isomerase FKBP8-like isoform X2 [Oncorhynchus tshaw

(461)	XP_024269307	peptidyl-prolyl cis-trans isomerase FKBP8-like isoform X3 [Oncorhynchus tshaw
(462)	XP_024269300	peptidyl-prolyl cis-trans isomerase FKBP8-like isoform X1 [Oncorhynchus tshaw
(463)	XP_017893134	PREDICTED: peptidyl-prolyl cis-trans isomerase FKBP8 [Ceratina calcarata].
(464)	XP_021454564	peptidyl-prolyl cis-trans isomerase FKBP8-like isoform X2 [Oncorhynchus mykis
(465)	XP_003967095	PREDICTED: peptidyl-prolyl cis-trans isomerase FKBP8-like [Takifugu rubripes]
(466)	XP_021454548	peptidyl-prolyl cis-trans isomerase FKBP8-like isoform X1 [Oncorhynchus mykis
(467)	XP_023697743	peptidyl-prolyl cis-trans isomerase FKBP8-like [Paramormyrops kingsleyae].
(468)	PIK51124	putative peptidyl-prolyl cis-trans isomerase FKBP8 [Apostichopus japonicus].
(469)	XP_020311464	peptidyl-prolyl cis-trans isomerase FKBP8-like [Oncorhynchus kisutch].
(470)	XP_017261735	peptidyl-prolyl cis-trans isomerase FKBP8-like [Kryptolebias marmoratus].
(471)	XP_011312767	PREDICTED: peptidyl-prolyl cis-trans isomerase FKBP8 [Fopius arisanus].
(472)	XP_022047038	peptidyl-prolyl cis-trans isomerase FKBP8-like [Acanthochromis polyacanthus].
(473)	XP_003427294	PREDICTED: peptidyl-prolyl cis-trans isomerase FKBP8 [Nasonia vitripennis].
(474)	XP_020139363	peptidyl-prolyl cis-trans isomerase FKBP8 [Microcebus murinus].
(475)	XP_011661975	PREDICTED: peptidyl-prolyl cis-trans isomerase FKBP8 [Strongylocentrotus purp
(476)	XP_021263191	peptidyl-prolyl cis-trans isomerase FKBP8-like isoform X2 [Numida meleagris].
(477)	XP_005110253	PREDICTED: peptidyl-prolyl cis-trans isomerase FKBP8-like [Aplysia californic
(478)	OCT89685	hypothetical protein XELAEV_18018304mg [Xenopus laevis].
(479)	XP_018106932	PREDICTED: peptidyl-prolyl cis-trans isomerase FKBP8-like [Xenopus laevis].
(480)	XP_022609905	peptidyl-prolyl cis-trans isomerase FKBP8-like [Seriola dumerili].
(481)	XP_023278137	peptidyl-prolyl cis-trans isomerase FKBP8-like [Seriola lalandi dorsalis].
(482)	XP_023151164	peptidyl-prolyl cis-trans isomerase FKBP8-like [Amphiprion ocellaris].
(483)	OON20683	peptidyl-prolyl cis-trans isomerase, FKBP-type [Opisthorchis viverrini].
(484)	XP_014340350	PREDICTED: peptidyl-prolyl cis-trans isomerase FKBP8-like [Latimeria chalumna
(485)	XP_012284026	peptidyl-prolyl cis-trans isomerase FKBP8 [Orussus abietinus].
(486)	OAD61952	Peptidyl-prolyl cis-trans isomerase FKBP8 [Eufriesea mexicana].
(487)	XP_023962949	peptidyl-prolyl cis-trans isomerase FKBP8-like isoform X2 [Chrysemys picta be
(488)	XP_008121023	PREDICTED: peptidyl-prolyl cis-trans isomerase FKBP8 [Anolis carolinensis].
(489)	XP_017763036	PREDICTED: peptidyl-prolyl cis-trans isomerase FKBP8-like [Eufriesea mexicana
(490)	XP_017691650	PREDICTED: peptidyl-prolyl cis-trans isomerase FKBP8-like [Lepidothrix corona
(491)	XP_012710521	peptidyl-prolyl cis-trans isomerase FKBP8 [Fundulus heteroclitus].
(492)	XP_015819997	PREDICTED: peptidyl-prolyl cis-trans isomerase FKBP8-like [Nothobranchius fur
(493)	XP_023962948	peptidyl-prolyl cis-trans isomerase FKBP8-like isoform X1 [Chrysemys picta be
(494)	XP_019921720	PREDICTED: peptidyl-prolyl cis-trans isomerase FKBP8 isoform X2 [Crassostrea
(495)	XP_020904887	peptidyl-prolyl cis-trans isomerase FKBP8-like [Exaiptasia pallida].
(496)	XP_024138547	peptidyl-prolyl cis-trans isomerase FKBP8-like isoform X3 [Oryzias melastigma
(497)	AWP08482	putative peptidyl-prolyl cis-trans isomerase FKBP8-like [Scophthalmus maximus
(498)	CDQ67637	unnamed protein product [Oncorhynchus mykiss].
(499)	XP_011423840	PREDICTED: peptidyl-prolyl cis-trans isomerase FKBP8 isoform X1 [Crassostrea
(500)	XP_014808190	PREDICTED: peptidyl-prolyl cis-trans isomerase FKBP8-like [Calidris pugnax].
(501)	XP_007059625	PREDICTED: peptidyl-prolyl cis-trans isomerase FKBP8-like [Chelonia mydas].
(502)	XP_024138546	peptidyl-prolyl cis-trans isomerase FKBP8-like isoform X2 [Oryzias melastigma

(503) EMP36068 Peptidyl-prolyl cis-trans isomerase FKBP8 [Chelonia mydas].
(504) XP_024138543 peptidyl-prolyl cis-trans isomerase FKBP8-like isoform X1 [Oryzias melastigma
(505) OXU31097 hypothetical protein TSAR_016393 [Trichomalopsis sarcophagae].
(506) XP_022202921 peptidyl-prolyl cis-trans isomerase FKBP8 [Nilaparvata lugens].
(507) XP_006557516 PREDICTED: peptidyl-prolyl cis-trans isomerase FKBP8 isoform X4 [Apis mellife
(508) XP_016770764 PREDICTED: peptidyl-prolyl cis-trans isomerase FKBP8 isoform X3 [Apis mellife
(509) XP_006615021 PREDICTED: peptidyl-prolyl cis-trans isomerase FKBP8-like isoform X3 [Apis do
(510) XP_008546855 PREDICTED: peptidyl-prolyl cis-trans isomerase FKBP8 [Microplitis demolitor].
(511) XP_006557515 PREDICTED: peptidyl-prolyl cis-trans isomerase FKBP8 isoform X2 [Apis mellife
(512) XP_007254541 peptidyl-prolyl cis-trans isomerase FKBP8-like isoform X2 [Astyanax mexicanus
(513) XP_006615020 PREDICTED: peptidyl-prolyl cis-trans isomerase FKBP8-like isoform X2 [Apis do
(514) XP_006557514 PREDICTED: peptidyl-prolyl cis-trans isomerase FKBP8 isoform X1 [Apis mellife
(515) XP_011053698 PREDICTED: peptidyl-prolyl cis-trans isomerase FKBP8 isoform X4 [Acromyrmex e
(516) XP_007254540 peptidyl-prolyl cis-trans isomerase FKBP8-like isoform X1 [Astyanax mexicanus
(517) XP_006615019 PREDICTED: peptidyl-prolyl cis-trans isomerase FKBP8-like isoform X1 [Apis do
(518) XP_016913411 PREDICTED: peptidyl-prolyl cis-trans isomerase FKBP8-like isoform X3 [Apis ce
(519) XP_017559733 PREDICTED: peptidyl-prolyl cis-trans isomerase FKBP8-like [Pygocentrus natter
(520) PKU40687 peptidyl-prolyl cis-trans isomerase fkbp8-like [Limosa lapponica baueri].
(521) XP_016913410 PREDICTED: peptidyl-prolyl cis-trans isomerase FKBP8-like isoform X2 [Apis ce
(522) EGI65888 FK506-binding protein 8 [Acromyrmex echinatior].
(523) XP_014252350 peptidyl-prolyl cis-trans isomerase FKBP8 [Cimex lectularius].
(524) XP_012165944 LOW QUALITY PROTEIN: peptidyl-prolyl cis-trans isomerase FKBP8 [Bombus terres
(525) XP_016913409 PREDICTED: peptidyl-prolyl cis-trans isomerase FKBP8-like isoform X1 [Apis ce
(526) XP_011053696 PREDICTED: peptidyl-prolyl cis-trans isomerase FKBP8 isoform X3 [Acromyrmex e
(527) XP_019934661 PREDICTED: peptidyl-prolyl cis-trans isomerase FKBP8-like [Paralichthys oliva
(528) XP_008304064 PREDICTED: peptidyl-prolyl cis-trans isomerase FKBP8-like, partial [Stegastes
(529) XP_011053695 PREDICTED: peptidyl-prolyl cis-trans isomerase FKBP8 isoform X2 [Acromyrmex e
(530) CAX74525 hypotherical protein [Schistosoma japonicum].
(531) XP_023811597 peptidyl-prolyl cis-trans isomerase FKBP8-like isoform X3 [Oryzias latipes].
(532) EFZ15418 hypothetical protein SINV_13234, partial [Solenopsis invicta].
(533) OPJ70235 hypothetical protein AV530_019426 [Patagioenas fasciata monilis].
(534) AAX27902 SJCHGC02834 protein, partial [Schistosoma japonicum].
(535) XP_011053694 PREDICTED: peptidyl-prolyl cis-trans isomerase FKBP8 isoform X1 [Acromyrmex e
(536) XP_011164040 PREDICTED: peptidyl-prolyl cis-trans isomerase FKBP8 isoform X2 [Solenopsis i
(537) XP_011150605 peptidyl-prolyl cis-trans isomerase FKBP8 [Harpegnathos saltator].
(538) EFN77170 FK506-binding protein 8 [Harpegnathos saltator].
(539) XP_011164039 PREDICTED: peptidyl-prolyl cis-trans isomerase FKBP8 isoform X1 [Solenopsis i
(540) AAH90698 Si:ch211-13k12.1 protein, partial [Danio rerio].
(541) XP_014606393 PREDICTED: peptidyl-prolyl cis-trans isomerase FKBP8 isoform X2 [Polistes can
(542) XP_003700328 PREDICTED: peptidyl-prolyl cis-trans isomerase FKBP8 [Megachile rotundata].
(543) XP_683608 peptidyl-prolyl cis-trans isomerase FKBP8 isoform X1 [Danio rerio].
(544) XP_014606392 PREDICTED: peptidyl-prolyl cis-trans isomerase FKBP8 isoform X1 [Polistes can

(545) XP_023726255 peptidyl-prolyl cis-trans isomerase FKBP8 isoform X1 [Cryptotermes secundus].
(546) XP_011404052 PREDICTED: peptidyl-prolyl cis-trans isomerase FKBP8-like [Amphimedon queensl
(547) XP_018337850 PREDICTED: peptidyl-prolyl cis-trans isomerase FKBP8 [Trachymyrmex septentrio
(548) XP_023726263 peptidyl-prolyl cis-trans isomerase FKBP8 isoform X2 [Cryptotermes secundus].
(549) XP_012689022 PREDICTED: peptidyl-prolyl cis-trans isomerase FKBP8-like [Clupea harengus].
(550) XP_015190182 PREDICTED: peptidyl-prolyl cis-trans isomerase FKBP8 [Polistes dominula].
(551) XP_024943519 peptidyl-prolyl cis-trans isomerase FKBP8 isoform X3 [Cephus cinctus].
(552) XP_015437909 PREDICTED: peptidyl-prolyl cis-trans isomerase FKBP8 [Dufourea novaeangliae].
(553) KYN13005 Peptidyl-prolyl cis-trans isomerase FKBP8 [Trachymyrmex cornetzi].
(554) XP_024943518 peptidyl-prolyl cis-trans isomerase FKBP8 isoform X2 [Cephus cinctus].
(555) XP_012526171 PREDICTED: peptidyl-prolyl cis-trans isomerase FKBP8 [Monomorium pharaonis].
(556) XP_012237396 peptidyl-prolyl cis-trans isomerase FKBP8 [Bombus impatiens].
(557) XP_018372055 PREDICTED: peptidyl-prolyl cis-trans isomerase FKBP8-like isoform X2 [Trachym
(558) XP_015600846 peptidyl-prolyl cis-trans isomerase FKBP8 isoform X1 [Cephus cinctus].
(559) XP_018372054 PREDICTED: peptidyl-prolyl cis-trans isomerase FKBP8-like isoform X1 [Trachym
(560) XP_023811598 peptidyl-prolyl cis-trans isomerase FKBP8-like isoform X4 [Oryzias latipes].
(561) XP_013086934 PREDICTED: peptidyl-prolyl cis-trans isomerase FKBP8-like [Biomphalaria glabr
(562) XP_012340112 PREDICTED: peptidyl-prolyl cis-trans isomerase FKBP8 isoform X3 [Apis florea]
(563) XP_012064601 PREDICTED: peptidyl-prolyl cis-trans isomerase FKBP8 [Atta cephalotes].
(564) XP_021933338 peptidyl-prolyl cis-trans isomerase FKBP8-like isoform X2 [Zootermopsis nevad
(565) XP_020469449 peptidyl-prolyl cis-trans isomerase FKBP8-like [Monopterus albus].
(566) XP_012340111 PREDICTED: peptidyl-prolyl cis-trans isomerase FKBP8 isoform X2 [Apis florea]
(567) XP_014772499 PREDICTED: FK506-binding protein-like isoform X3 [Octopus bimaculoides].
(568) XP_021933337 peptidyl-prolyl cis-trans isomerase FKBP8-like isoform X1 [Zootermopsis nevad
(569) XP_002425448 fk506 binding protein, putative [Pediculus humanus corporis].
(570) XP_018053112 PREDICTED: peptidyl-prolyl cis-trans isomerase FKBP8 [Atta colombica].
(571) XP_023811595 peptidyl-prolyl cis-trans isomerase FKBP8-like isoform X2 [Oryzias latipes].
(572) XP_003692338 PREDICTED: peptidyl-prolyl cis-trans isomerase FKBP8 isoform X1 [Apis florea]
(573) KQK73886 peptidyl-prolyl cis-trans isomerase FKBP8-like protein [Amazona aestiva].
(574) XP_021933339 peptidyl-prolyl cis-trans isomerase FKBP8-like isoform X3 [Zootermopsis nevad
(575) XP_018025482 PREDICTED: peptidyl-prolyl cis-trans isomerase FKBP8-like [Hyalella azteca].
(576) XP_019335449 PREDICTED: peptidyl-prolyl cis-trans isomerase FKBP8-like, partial [Alligator
(577) XP_014772498 PREDICTED: peptidyl-prolyl cis-trans isomerase FKBP8-like isoform X2 [Octopus
(578) XP_023811594 peptidyl-prolyl cis-trans isomerase FKBP8-like isoform X1 [Oryzias latipes].
(579) XP_001846585 FK506 binding protein [Culex quinquefasciatus].
(580) XP_014470017 PREDICTED: peptidyl-prolyl cis-trans isomerase FKBP8 isoform X2 [Dinoponera q
(581) XP_002111046 hypothetical protein TRIADDRAFT_63767 [Trichoplax adhaerens].
(582) XP_014772494 PREDICTED: peptidyl-prolyl cis-trans isomerase FKBP8-like isoform X1 [Octopus
(583) KZS20178 Tetratricopeptide repeat protein 9C [Daphnia magna].
(584) XP_014470016 PREDICTED: peptidyl-prolyl cis-trans isomerase FKBP8 isoform X1 [Dinoponera q
(585) KFM75734 Peptidyl-prolyl cis-trans isomerase FKBP8, partial [Stegodyphus mimosarum].
(586) XP_018900271 PREDICTED: peptidyl-prolyl cis-trans isomerase FKBP8 [Bemisia tabaci].

(587) XP_020392992 peptidyl-prolyl cis-trans isomerase FKBP8-like [Rhincodon typus].
(588) XP_016376789 PREDICTED: peptidyl-prolyl cis-trans isomerase FKBP8-like isoform X3 [Sinocyc
(589) XP_016345079 PREDICTED: peptidyl-prolyl cis-trans isomerase FKBP8-like [Sinocyclocheilus a
(590) XP_014106477 PREDICTED: peptidyl-prolyl cis-trans isomerase FKBP8-like [Pseudopodoces humi
(591) XP_014237957 peptidyl-prolyl cis-trans isomerase FKBP8 [Trichogramma pretiosum].
(592) XP_016376788 PREDICTED: peptidyl-prolyl cis-trans isomerase FKBP8-like isoform X2 [Sinocyc
(593) XP_016376787 PREDICTED: peptidyl-prolyl cis-trans isomerase FKBP8-like isoform X1 [Sinocyc
(594) CDS28193 FK506 binding protein 8 38 kDa [Hymenolepis microstoma].
(595) KOC65132 Peptidyl-prolyl cis-trans isomerase FKBP8 [Habropoda laboriosa].
(596) XP_017790334 PREDICTED: peptidyl-prolyl cis-trans isomerase FKBP8 [Habropoda laboriosa].
(597) XP_020790728 peptidyl-prolyl cis-trans isomerase FKBP8-like [Boleophthalmus pectinirostris
(598) XP_012221110 PREDICTED: peptidyl-prolyl cis-trans isomerase FKBP8 isoform X3 [Linepithema
(599) GAA30660 peptidyl-prolyl cis-trans isomerase FKBP8, partial [Clonorchis sinensis].
(600) XP_012221101 PREDICTED: peptidyl-prolyl cis-trans isomerase FKBP8 isoform X2 [Linepithema
(601) XP_012221093 PREDICTED: peptidyl-prolyl cis-trans isomerase FKBP8 isoform X1 [Linepithema
(602) XP_018649440 putative fk506 binding protein [Schistosoma mansoni].
(603) EFX68474 hypothetical protein DAPPUDRAFT_63074 [Daphnia pulex].
(604) XP_023229640 peptidyl-prolyl cis-trans isomerase FKBP8-like [Centruroides sculpturatus].
(605) EAT32833 AAEL014933-PA, partial [Aedes aegypti].
(606) XP_016344167 PREDICTED: peptidyl-prolyl cis-trans isomerase FKBP8-like isoform X2 [Sinocyc
(607) XP_016344168 PREDICTED: peptidyl-prolyl cis-trans isomerase FKBP8-like isoform X3 [Sinocyc
(608) XP_016344166 PREDICTED: peptidyl-prolyl cis-trans isomerase FKBP8-like isoform X1 [Sinocyc
(609) XP_016376234 PREDICTED: peptidyl-prolyl cis-trans isomerase FKBP8-like [Sinocyclocheilus r
(610) XP_007906811 PREDICTED: peptidyl-prolyl cis-trans isomerase FKBP8-like isoform X2 [Callorh
(611) KYN04145 Peptidyl-prolyl cis-trans isomerase FKBP8 [Cyphomyrmex costatus].
(612) XP_014282311 peptidyl-prolyl cis-trans isomerase FKBP8 [Halyomorpha halys].
(613) XP_015109871 PREDICTED: peptidyl-prolyl cis-trans isomerase FKBP8-like [Diachasma alloeum]
(614) CDS42647 FK506 binding protein 8 38 kDa [Echinococcus multilocularis].
(615) XP_011870270 PREDICTED: peptidyl-prolyl cis-trans isomerase FKBP8 isoform X2 [Vollenhovia
(616) XP_011870269 PREDICTED: peptidyl-prolyl cis-trans isomerase FKBP8 isoform X1 [Vollenhovia
(617) XP_018977514 PREDICTED: peptidyl-prolyl cis-trans isomerase FKBP8-like [Cyprinus carpio].
(618) XP_007906810 PREDICTED: peptidyl-prolyl cis-trans isomerase FKBP8-like isoform X1 [Callorh
(619) CDS17605 FK506 binding protein 8 38 kDa [Echinococcus granulosus].
(620) XP_014206998 peptidyl-prolyl cis-trans isomerase FKBP8 [Copidosoma floridanum].
(621) XP_018393954 PREDICTED: peptidyl-prolyl cis-trans isomerase FKBP8 [Cyphomyrmex costatus].
(622) XP_015929100 peptidyl-prolyl cis-trans isomerase FKBP8 [Parasteatoda tepidariorum].
(623) ELU06040 hypothetical protein CAPTEDRAFT_168280 [Capitella teleta].
(624) XP_024353326 Peptidyl-prolyl cis-trans isomerase FKBP8 [Echinococcus granulosus].
(625) EAT35561 AAEL012272-PA [Aedes aegypti].
(626) XP_018359770 PREDICTED: peptidyl-prolyl cis-trans isomerase FKBP8-like [Trachymyrmex corne
(627) EAT35560 AAEL012272-PB [Aedes aegypti].
(628) XP_018319727 PREDICTED: peptidyl-prolyl cis-trans isomerase FKBP8 isoform X2 [Agrilus plan

(629)	XP_001662371	peptidyl-prolyl cis-trans isomerase FKBP8 isoform X2 [Aedes aegypti].
(630)	XP_018319726	PREDICTED: peptidyl-prolyl cis-trans isomerase FKBP8 isoform X1 [Agrilus plan
(631)	XP_001662370	peptidyl-prolyl cis-trans isomerase FKBP8 isoform X1 [Aedes aegypti].
(632)	XP_012269548	peptidyl-prolyl cis-trans isomerase FKBP8 isoform X1 [Athalia rosae].
(633)	PAA92621	hypothetical protein BOX15_Mlig010424g1 [Macrostomum lignano].
(634)	XP_019867812	PREDICTED: LOW QUALITY PROTEIN: peptidyl-prolyl cis-trans isomerase FKBP8-lik
(635)	PAA53270	hypothetical protein BOX15_Mlig007838g1 [Macrostomum lignano].
(636)	PAA57938	hypothetical protein BOX15_Mlig010979g3 [Macrostomum lignano].
(637)	XP_966821	PREDICTED: peptidyl-prolyl cis-trans isomerase FKBP8 [Tribolium castaneum].
(638)	KXJ84517	hypothetical protein RP20_CCG023547 [Aedes albopictus].
(639)	XP_023345477	peptidyl-prolyl cis-trans isomerase FKBP8-like isoform X3 [Eurytemora affinis
(640)	XP_011494478	PREDICTED: peptidyl-prolyl cis-trans isomerase FKBP8 [Ceratosolen solmsi marc
(641)	XP_023345475	peptidyl-prolyl cis-trans isomerase FKBP8-like isoform X1 [Eurytemora affinis
(642)	XP_016940923	PREDICTED: peptidyl-prolyl cis-trans isomerase FKBP8 [Drosophila suzukii].
(643)	XP_023345476	peptidyl-prolyl cis-trans isomerase FKBP8-like isoform X2 [Eurytemora affinis
(644)	PNF41144	hypothetical protein B7P43_G04230 [Cryptotermes secundus].
(645)	XP_019932587	PREDICTED: peptidyl-prolyl cis-trans isomerase FKBP8-like isoform X2 [Aedes a
(646)	XP_019932586	PREDICTED: peptidyl-prolyl cis-trans isomerase FKBP8-like isoform X1 [Aedes a
(647)	XP_016028426	uncharacterized protein Dsimw501_GD25378, isoform B [Drosophila simulans].
(648)	XP_016958803	PREDICTED: peptidyl-prolyl cis-trans isomerase FKBP8 [Drosophila biarmipes].
(649)	EKC18274	hypothetical protein CGI_10013873 [Crassostrea gigas].
(650)	PNF41145	hypothetical protein B7P43_G04230, partial [Cryptotermes secundus].
(651)	XP_011636531	peptidyl-prolyl cis-trans isomerase FKBP8 [Pogonomyrmex barbatus].
(652)	XP_017014991	PREDICTED: peptidyl-prolyl cis-trans isomerase FKBP8 [Drosophila takahashii].
(653)	XP_001974711	uncharacterized protein Dere_GG20961 [Drosophila erecta].
(654)	XP_024874265	peptidyl-prolyl cis-trans isomerase FKBP8-like isoform X2 [Temnothorax curvis
(655)	NP_611353	uncharacterized protein Dmel_CG5482, isoform A [Drosophila melanogaster].
(656)	XP_016984960	PREDICTED: peptidyl-prolyl cis-trans isomerase FKBP8 [Drosophila rhopaloa].
(657)	XP_017054378	PREDICTED: peptidyl-prolyl cis-trans isomerase FKBP8 [Drosophila ficusphila].
(658)	XP_024874264	peptidyl-prolyl cis-trans isomerase FKBP8-like isoform X1 [Temnothorax curvis
(659)	XP_019545364	PREDICTED: peptidyl-prolyl cis-trans isomerase FKBP8 isoform X2 [Aedes albopi
(660)	XP_019545363	PREDICTED: peptidyl-prolyl cis-trans isomerase FKBP8 isoform X1 [Aedes albopi
(661)	XP_014670901	PREDICTED: peptidyl-prolyl cis-trans isomerase FKBP8-like isoform X1 [Priapul
(662)	XP_017079421	PREDICTED: peptidyl-prolyl cis-trans isomerase FKBP8 [Drosophila eugracilis].
(663)	XP_013113222	PREDICTED: peptidyl-prolyl cis-trans isomerase FKBP8 [Stomoxys calcitrans].
(664)	XP_002034480	GM19892 [Drosophila sechellia].
(665)	XP_017031858	PREDICTED: peptidyl-prolyl cis-trans isomerase FKBP8 [Drosophila kikkawai].
(666)	XP_015788695	peptidyl-prolyl cis-trans isomerase FKBP8 [Tetranychus urticae].
(667)	XP_017117238	PREDICTED: peptidyl-prolyl cis-trans isomerase FKBP8 [Drosophila elegans].
(668)	XP_015364528	PREDICTED: peptidyl-prolyl cis-trans isomerase FKBP8 [Diuraphis noxia].
(669)	XP_017836078	PREDICTED: peptidyl-prolyl cis-trans isomerase FKBP8 [Drosophila busckii].
(670)	XP_316645	AGAP006615-PA [Anopheles gambiae str. PEST].

(671) XP_002091890 uncharacterized protein Dyak_GE13900 [Drosophila yakuba].
(672) XP_009022869 hypothetical protein HELRODRAFT_188959 [Helobdella robusta].
(673) XP_020815089 peptidyl-prolyl cis-trans isomerase FKBP8 [Drosophila serrata].
(674) KYO18601 peptidyl-prolyl cis-trans isomerase FKBP8-like [Alligator mississippiensis].
(675) XP_023307145 peptidyl-prolyl cis-trans isomerase FKBP8 [Lucilia cuprina].
(676) XP_004526322 peptidyl-prolyl cis-trans isomerase FKBP8 [Ceratitis capitata].
(677) XP_017468465 PREDICTED: peptidyl-prolyl cis-trans isomerase FKBP8 [Rhagoletis zephyria].
(678) XP_011213061 PREDICTED: peptidyl-prolyl cis-trans isomerase FKBP8 [Bactrocera dorsalis].
(679) ETN59059 fk506 binding protein [Anopheles darlingi].
(680) XP_019877780 PREDICTED: peptidyl-prolyl cis-trans isomerase FKBP8-like [Aethina tumida].
(681) XP_001951673 PREDICTED: peptidyl-prolyl cis-trans isomerase FKBP8 [Acyrthosiphon pisum].
(682) XP_002062976 peptidyl-prolyl cis-trans isomerase FKBP8 [Drosophila willistoni].
(683) XP_001986816 GH21577 [Drosophila grimshawi].
(684) XP_022181773 peptidyl-prolyl cis-trans isomerase FKBP8 [Myzus persicae].
(685) XP_002048800 uncharacterized protein Dvir_GJ21121 [Drosophila virilis].
(686) XP_018801327 PREDICTED: peptidyl-prolyl cis-trans isomerase FKBP8 [Bactrocera latifrons].
(687) XP_020300622 peptidyl-prolyl cis-trans isomerase FKBP8 [Pseudomyrmex gracilis].
(688) XP_011193416 PREDICTED: peptidyl-prolyl cis-trans isomerase FKBP8 [Zeugodacus cucurbitae].
(689) XP_017960399 PREDICTED: peptidyl-prolyl cis-trans isomerase FKBP8 [Drosophila navojoa].
(690) ACO13176 FK506-binding protein 8 [Lepeophtheirus salmonis].
(691) XP_011350211 PREDICTED: peptidyl-prolyl cis-trans isomerase FKBP8 [Ooceraea biroi].
(692) KRT81327 hypothetical protein AMK59_5847, partial [Oryctes borbonicus].
(693) XP_023162735 peptidyl-prolyl cis-trans isomerase FKBP8 [Drosophila hydei].
(694) XP_019771996 PREDICTED: peptidyl-prolyl cis-trans isomerase FKBP8 [Dendroctonus ponderosae
(695) XP_005176045 PREDICTED: peptidyl-prolyl cis-trans isomerase FKBP8 [Musca domestica].
(696) XP_014095636 PREDICTED: peptidyl-prolyl cis-trans isomerase FKBP8 [Bactrocera oleae].
(697) XP_017867395 PREDICTED: peptidyl-prolyl cis-trans isomerase FKBP8 [Drosophila arizonae].
(698) XP_002004550 uncharacterized protein Dmoj_GI19549 [Drosophila mojavensis].
(699) XP_022921247 peptidyl-prolyl cis-trans isomerase FKBP8-like [Onthophagus taurus].
(700) XP_002015767 GL11238 [Drosophila persimilis].
(701) XP_001360319 uncharacterized protein Dpse_GA18915 [Drosophila pseudoobscura pseudoobscura]
(702) XP_017147297 PREDICTED: peptidyl-prolyl cis-trans isomerase FKBP8 [Drosophila miranda].
(703) XP_019879021 PREDICTED: peptidyl-prolyl cis-trans isomerase FKBP8-like [Aethina tumida].
