# Supplementary material for: Compression of Large Sets of Sequence Data Reveals Fine Diversification of Functional Profiles in Multigene Families of Proteins: A Study for Peptidyl-Prolyl cis/trans Isomerases (PPIase)
Source: Biomolecules. 2019 Feb 11;9(2):59. doi: 10.3390/biom9020059 (PMC6406343; doi:10.3390/biom9020059)
Supplement: Supplementary file 1 [file biomolecules-09-00059-s001.zip › Vertical MSA of cyclophilins .pdf]

Arrangement of cyclophilins in the heat-map

<table>
<tr><th></th><th>PPIA</th><th>PPIB</th><th>PPIC</th><th>PPIF</th><th>PPIL3A</th><th>PPIL1</th><th>PPID</th><th>PPWD1</th><th>PPIL2</th><th>RANBP</th><th>PPIL4</th><th>PPIG</th><th>NKTR</th><th>PPIE</th><th>PPIL4</th><th>CWC27</th><th>PPIL6</th></tr>
<tr><td>1</td><td>V</td><td>V</td><td>V</td><td>G</td><td>-</td><td>-</td><td>S</td><td>E!</td><td>-</td><td>T!</td><td>V</td><td>Q</td><td>D*</td><td>S</td><td>-</td><td>I|</td><td>K</td></tr>
<tr><td>2</td><td>N!</td><td>T!</td><td>T</td><td>N!</td><td>-</td><td>-</td><td>N!</td><td>G!</td><td>-</td><td>N*</td><td>N*</td><td>R*</td><td>R*</td><td>N*</td><td>-</td><td>Q!</td><td>H*</td></tr>
<tr><td>3</td><td>P!</td><td>V</td><td>A</td><td>P!</td><td>-</td><td>-</td><td>P!</td><td>P!</td><td>-</td><td>P*</td><td>P*</td><td>P|</td><td>P*</td><td>P*</td><td>-</td><td>E*</td><td>D</td></tr>
<tr><td>4</td><td>T</td><td>K!</td><td>K</td><td>L</td><td>-</td><td>-</td><td>R!</td><td>K!</td><td>V!</td><td>V!</td><td>V!</td><td>R!</td><td>Q*</td><td>Q*</td><td>-</td><td>P*</td><td>F|</td></tr>
<tr><td>5</td><td>V!</td><td>V*</td><td>V*</td><td>V!</td><td>-</td><td>-</td><td>V</td><td>R|</td><td>K|</td><td>V*</td><td>V*</td><td>C|</td><td>C*</td><td>V*</td><td>-</td><td>P|</td><td>V*</td></tr>
<tr><td>6</td><td>F!</td><td>Y</td><td>F*</td><td>Y</td><td>-</td><td>-</td><td>F</td><td>V!</td><td>K</td><td>F!</td><td>F*</td><td>F!</td><td>H</td><td>Y*</td><td>-</td><td>T!</td><td>F!</td></tr>
<tr><td>7</td><td>F|</td><td>F*</td><td>F*</td><td>L</td><td>-</td><td>P</td><td>F</td><td>S!</td><td>K!</td><td>F|</td><td>F|</td><td>F|</td><td>F*</td><td>M!</td><td>-</td><td>N</td><td>L!</td></tr>
<tr><td>8</td><td>D!</td><td>D*</td><td>D*</td><td>D!</td><td>M</td><td>P</td><td>D!</td><td>D!</td><td>G|</td><td>D</td><td>D|</td><td>D*</td><td>D!</td><td>D*</td><td>-</td><td>G|</td><td>D!</td></tr>
<tr><td>9</td><td>I!</td><td>L</td><td>V!</td><td>V</td><td>S</td><td>N</td><td>V</td><td>S!</td><td>Y*</td><td>V!</td><td>V</td><td>I!</td><td>I!</td><td>I*</td><td>M|</td><td>K*</td><td>I!</td></tr>
<tr><td>10</td><td>A</td><td>R</td><td>R</td><td>D</td><td>V*</td><td>V!</td><td>D</td><td>A*</td><td>V</td><td>C</td><td>S</td><td>A</td><td>E*</td><td>K!</td><td>A</td><td>V*</td><td>C</td></tr>
<tr><td>11</td><td>V</td><td>I!</td><td>I|</td><td>A!</td><td>T*</td><td>Y</td><td>I</td><td>I!</td><td>R!</td><td>A|</td><td>I</td><td>I!</td><td>I!</td><td>I|</td><td>V|</td><td>L!</td><td>I!</td></tr>
<tr><td>12</td><td>D</td><td>G*</td><td>G|</td><td>N</td><td>L|</td><td>L</td><td>G</td><td>I!</td><td>L</td><td>D|</td><td>G!</td><td>N!</td><td>N*</td><td>G*</td><td>L</td><td>L!</td><td>D</td></tr>
<tr><td>13</td><td>G</td><td>D</td><td>D!</td><td>G</td><td>H|</td><td>E</td><td>G</td><td>H*</td><td>H</td><td>G</td><td>G</td><td>N</td><td>R*</td><td>N!</td><td>L</td><td>K!</td><td>S</td></tr>
<tr><td>14</td><td>E</td><td>E!</td><td>K</td><td>K</td><td>T*</td><td>T*</td><td>E</td><td>T*</td><td>T*</td><td>E|</td><td>Q</td><td>Q</td><td>E*</td><td>K!</td><td>E!</td><td>T*</td><td>S</td></tr>
<tr><td>15</td><td>P!</td><td>D</td><td>D!</td><td>P</td><td>D!</td><td>S</td><td>R</td><td>S!</td><td>N!</td><td>P|</td><td>E</td><td>P!</td><td>P*</td><td>P</td><td>T|</td><td>T!</td><td>P</td></tr>
<tr><td>16</td><td>L!</td><td>V</td><td>V|</td><td>L</td><td>V</td><td>M!</td><td>V</td><td>M|</td><td>K</td><td>L|</td><td>V</td><td>A</td><td>V*</td><td>A!</td><td>T!</td><td>A!</td><td>I</td></tr>
<tr><td>17</td><td>G!</td><td>G*</td><td>G*</td><td>G!</td><td>G*</td><td>G*</td><td>G*</td><td>G*</td><td>G*</td><td>G*</td><td>G*</td><td>G*</td><td>G*</td><td>G*</td><td>L</td><td>G*</td><td>G*</td></tr>
<tr><td>18</td><td>R</td><td>R*</td><td>R*</td><td>R!</td><td>-</td><td>I</td><td>R*</td><td>D*</td><td>D</td><td>R</td><td>R|</td><td>R*</td><td>R*</td><td>R*</td><td>G|</td><td>D|</td><td>R!</td></tr>
<tr><td>19</td><td>V</td><td>V!</td><td>I*</td><td>V</td><td>D!</td><td>I</td><td>I|</td><td>I*</td><td>L!</td><td>I|</td><td>M</td><td>V!</td><td>I*</td><td>I!</td><td>D!</td><td>I*</td><td>L|</td></tr>
<tr><td>20</td><td>S!</td><td>I!</td><td>V|</td><td>V</td><td>I</td><td>V</td><td>V!</td><td>H|</td><td>N|</td><td>T|</td><td>K</td><td>V!</td><td>M!</td><td>Q</td><td>V</td><td>D*</td><td>I</td></tr>
<tr><td>21</td><td>F!</td><td>F</td><td>I*</td><td>L</td><td>K*</td><td>L</td><td>L</td><td>T</td><td>L</td><td>M!</td><td>I</td><td>F!</td><td>F*</td><td>M</td><td>V</td><td>I!</td><td>F!</td></tr>
<tr><td>22</td><td>E!</td><td>G*</td><td>G*</td><td>E</td><td>I</td><td>E*</td><td>E*</td><td>K|</td><td>E*</td><td>E*</td><td>E*</td><td>E!</td><td>Q*</td><td>L!</td><td>I</td><td>E*</td><td>E|</td></tr>
<tr><td>23</td><td>L*</td><td>L|</td><td>L*</td><td>L*</td><td>E*</td><td>L|</td><td>L*</td><td>L*</td><td>L*</td><td>L*</td><td>L*</td><td>L*</td><td>L*</td><td>L*</td><td>D|</td><td>L*</td><td>L*</td></tr>
<tr><td>24</td><td>F*</td><td>F|</td><td>F*</td><td>K</td><td>V</td><td>Y*</td><td>F!</td><td>F*</td><td>H</td><td>F*</td><td>F|</td><td>F*</td><td>F*</td><td>R*</td><td>L|</td><td>W*</td><td>Y!</td></tr>
<tr><td>25</td><td>A|</td><td>G*</td><td>G*</td><td>A*</td><td>F|</td><td>W</td><td>A</td><td>P|</td><td>C</td><td>S!</td><td>A!</td><td>S|</td><td>S*</td><td>S!</td><td>Y</td><td>S!</td><td>C</td></tr>
<tr><td>26</td><td>D|</td><td>K|</td><td>K!</td><td>D|</td><td>C*</td><td>K</td><td>D*</td><td>V!</td><td>D</td><td>N!</td><td>D*</td><td>D*</td><td>D*</td><td>D*</td><td>T</td><td>K|</td><td>D|</td></tr>
<tr><td>27</td><td>K!</td><td>T|</td><td>V*</td><td>V*</td><td>E!</td><td>H*</td><td>I</td><td>E*</td><td>L</td><td>I*</td><td>V</td><td>V</td><td>I!</td><td>V!</td><td>E</td><td>E*</td><td>V</td></tr>
<tr><td>28</td><td>V!</td><td>V*</td><td>V*</td><td>V*</td><td>R</td><td>A|</td><td>V!</td><td>C*</td><td>T</td><td>V*</td><td>V</td><td>C*</td><td>C*</td><td>V*</td><td>E</td><td>A!</td><td>C|</td></tr>
<tr><td>29</td><td>P*</td><td>P*</td><td>P*</td><td>P*</td><td>T</td><td>P*</td><td>P*</td><td>P*</td><td>P*</td><td>P*</td><td>P*</td><td>P*</td><td>P*</td><td>P*</td><td>R!</td><td>P*</td><td>P*</td></tr>
<tr><td>30</td><td>K*</td><td>K*</td><td>K!</td><td>K*</td><td>P|</td><td>K</td><td>K|</td><td>K*</td><td>K</td><td>R</td><td>K!</td><td>K!</td><td>K*</td><td>M!</td><td>P|</td><td>K|</td><td>K!</td></tr>
<tr><td>31</td><td>T|</td><td>T*</td><td>T*</td><td>T*</td><td>K!</td><td>T*</td><td>T*</td><td>T*</td><td>T!</td><td>T*</td><td>T|</td><td>T*</td><td>T*</td><td>T*</td><td>R</td><td>A*</td><td>T*</td></tr>
<tr><td>32</td><td>A|</td><td>V!</td><td>V*</td><td>A*</td><td>T</td><td>C*</td><td>A*</td><td>V*</td><td>C</td><td>A*</td><td>A!</td><td>C|</td><td>C!</td><td>A!</td><td>A</td><td>C*</td><td>C!</td></tr>
<tr><td>33</td><td>E*</td><td>D</td><td>E</td><td>E*</td><td>C!</td><td>K</td><td>E*</td><td>E*</td><td>E</td><td>E*</td><td>E*</td><td>E!</td><td>K*</td><td>E*</td><td>C</td><td>R*</td><td>K</td></tr>
<tr><td>34</td><td>N*</td><td>N*</td><td>N*</td><td>N*</td><td>E|</td><td>N*</td><td>N*</td><td>N*</td><td>N*</td><td>N*</td><td>N*</td><td>N*</td><td>N*</td><td>N*</td><td>L</td><td>N*</td><td>N*</td></tr>
<tr><td>35</td><td>F*</td><td>F*</td><td>F*</td><td>F*</td><td>M*</td><td>F*</td><td>F*</td><td>F*</td><td>F*</td><td>F*</td><td>F*</td><td>F*</td><td>F*</td><td>F*</td><td>N*</td><td>F*</td><td>F*</td></tr>
<tr><td>36</td><td>R</td><td>V</td><td>V!</td><td>R*</td><td>E</td><td>A</td><td>R*</td><td>C|</td><td>I</td><td>R*</td><td>R*</td><td>R!</td><td>L*</td><td>R*</td><td>F*</td><td>I!</td><td>Q</td></tr>
<tr><td>37</td><td>A|</td><td>A</td><td>A</td><td>A*</td><td>S!</td><td>E</td><td>A|</td><td>V*</td><td>R</td><td>A*</td><td>Q|</td><td>C!</td><td>C*</td><td>C!</td><td>L*</td><td>Q*</td><td>V</td></tr>
</table>

<table>
<tr><th></th><th></th><th></th><th></th><th></th><th></th><th></th><th></th><th></th><th></th><th></th><th></th><th></th><th></th><th></th><th></th><th></th><th></th></tr>
<tr><td>38</td><td>L*</td><td>L*</td><td>L*</td><td>L*</td><td>R</td><td>L*</td><td>L*</td><td>H*</td><td>L!</td><td>L*</td><td>F!</td><td>L*</td><td>L*</td><td>L*</td><td>K*</td><td>L*</td><td>L*</td></tr>
<tr><td>39</td><td>S!</td><td>A*</td><td>A*</td><td>C*</td><td>C*</td><td>A</td><td>C*</td><td>S*</td><td>C</td><td>C*</td><td>C*</td><td>C*</td><td>C*</td><td>C*</td><td>L*</td><td>C*</td><td>C*</td></tr>
<tr><td>40</td><td>T*</td><td>T!</td><td>T*</td><td>T*</td><td>V*</td><td>R</td><td>T*</td><td>R|</td><td>K</td><td>T*</td><td>T*</td><td>T!</td><td>S!</td><td>T*</td><td>C*</td><td>L</td><td>T</td></tr>
<tr><td>41</td><td>G*</td><td>G!</td><td>G*</td><td>G*</td><td>P*</td><td>R|</td><td>G*</td><td>N*</td><td>K</td><td>G*</td><td>G*</td><td>G*</td><td>G*</td><td>H*</td><td>K*</td><td>E*</td><td>G*</td></tr>
<tr><td>42</td><td>E*</td><td>E!</td><td>E*</td><td>E*</td><td>Q*</td><td>G|</td><td>E*</td><td>G*</td><td>H</td><td>E*</td><td>E*</td><td>E*</td><td>E*</td><td>E*</td><td>I</td><td>A</td><td>K</td></tr>
<tr><td>43</td><td>K|</td><td>K!</td><td>K!</td><td>K!</td><td>A|</td><td>-</td><td>K*</td><td>-</td><td>-</td><td>K|</td><td>F</td><td>K|</td><td>K|</td><td>K*</td><td>K*</td><td>-</td><td>A</td></tr>
<tr><td>44</td><td>G*</td><td>G|</td><td>G*</td><td>G*</td><td>G*</td><td>-</td><td>G*</td><td>-</td><td>-</td><td>G*</td><td>R!</td><td>G*</td><td>G*</td><td>G*</td><td>-</td><td>-</td><td>G</td></tr>
<tr><td>45</td><td>-</td><td>-</td><td>-</td><td>-</td><td>-</td><td>-</td><td>I</td><td>-</td><td>-</td><td>-</td><td>K!</td><td>T</td><td>L</td><td>-</td><td>-</td><td>-</td><td>F</td></tr>
<tr><td>46</td><td>-</td><td>-</td><td>-</td><td>-</td><td>-</td><td>-</td><td>G*</td><td>-</td><td>-</td><td>-</td><td>-</td><td>G*</td><td>G*</td><td>-</td><td>-</td><td>-</td><td>S</td></tr>
<tr><td>47</td><td>-</td><td>-</td><td>-</td><td>-</td><td>-</td><td>-</td><td>H</td><td>-</td><td>-</td><td>-</td><td>-</td><td>K*</td><td>K*</td><td>-</td><td>-</td><td>-</td><td>Q</td></tr>
<tr><td>48</td><td>-</td><td>-</td><td>-</td><td>-</td><td>-</td><td>-</td><td>T</td><td>-</td><td>-</td><td>-</td><td>-</td><td>S</td><td>T!</td><td>-</td><td>-</td><td>-</td><td>-</td></tr>
<tr><td>49</td><td>-</td><td>-</td><td>-</td><td>-</td><td>-</td><td>-</td><td>T</td><td>-</td><td>-</td><td>-</td><td>D</td><td>T*</td><td>T*</td><td>-</td><td>-</td><td>-</td><td>R</td></tr>
<tr><td>50</td><td>-</td><td>-</td><td>-</td><td>-</td><td>-</td><td>-</td><td>G*</td><td>-</td><td>-</td><td>-</td><td>G|</td><td>Q</td><td>G|</td><td>-</td><td>-</td><td>-</td><td>G!</td></tr>
<tr><td>51</td><td>-</td><td>-</td><td>-</td><td>-</td><td>-</td><td>-</td><td>K!</td><td>-</td><td>-</td><td>-</td><td>V</td><td>K*</td><td>K*</td><td>-</td><td>-</td><td>-</td><td>I</td></tr>
<tr><td>52</td><td>-</td><td>-</td><td>-</td><td>-</td><td>V|</td><td>-</td><td>P*</td><td>-</td><td>-</td><td>-</td><td>P*</td><td>P!</td><td>K*</td><td>-</td><td>-</td><td>-</td><td>R</td></tr>
<tr><td>53</td><td>F*</td><td>F</td><td>Y|</td><td>F*</td><td>Q</td><td>-</td><td>L*</td><td>-</td><td>-</td><td>F*</td><td>I</td><td>L*</td><td>L*</td><td>F!</td><td>-</td><td>-</td><td>L*</td></tr>
<tr><td>54</td><td>G|</td><td>G*</td><td>G*</td><td>G*</td><td>W!</td><td>Y*</td><td>H|</td><td>Y*</td><td>Y*</td><td>G*</td><td>G*</td><td>H!</td><td>C*</td><td>G*</td><td>Y|</td><td>Y*</td><td>H</td></tr>
<tr><td>55</td><td>Y*</td><td>Y!</td><td>Y*</td><td>Y*</td><td>R*</td><td>Y*</td><td>F!</td><td>Y*</td><td>Y*</td><td>F!</td><td>Y!</td><td>Y*</td><td>Y*</td><td>F!</td><td>Y*</td><td>Y*</td><td>Y*</td></tr>
<tr><td>56</td><td>K*</td><td>K!</td><td>K|</td><td>K*</td><td>D</td><td>N</td><td>K*</td><td>N*</td><td>D</td><td>K!</td><td>K*</td><td>K*</td><td>K*</td><td>K!</td><td>N*</td><td>D</td><td>K</td></tr>
<tr><td>57</td><td>G|</td><td>N</td><td>G*</td><td>G*</td><td>L!</td><td>G</td><td>G*</td><td>G|</td><td>G</td><td>N!</td><td>G!</td><td>S</td><td>G*</td><td>G*</td><td>Y</td><td>N</td><td>N</td></tr>
<tr><td>58</td><td>S*</td><td>S*</td><td>S|</td><td>S*</td><td>G!</td><td>T</td><td>C</td><td>H*</td><td>T</td><td>S*</td><td>S</td><td>C</td><td>S!</td><td>S*</td><td>C</td><td>T*</td><td>S|</td></tr>
<tr><td>59</td><td>C!</td><td>K!</td><td>K</td><td>T!</td><td>S</td><td>K</td><td>P</td><td>T</td><td>I</td><td>I</td><td>T</td><td>L</td><td>T*</td><td>S!</td><td>L!</td><td>I!</td><td>I</td></tr>
<tr><td>60</td><td>F*</td><td>F*</td><td>F*</td><td>F*</td><td>L|</td><td>F*</td><td>F*</td><td>F*</td><td>F*</td><td>F*</td><td>F*</td><td>F*</td><td>F*</td><td>F*</td><td>I</td><td>F*</td><td>F|</td></tr>
<tr><td>61</td><td>H*</td><td>H*</td><td>H*</td><td>H*</td><td>Q|</td><td>H*</td><td>H*</td><td>H*</td><td>H*</td><td>H*</td><td>H*</td><td>H*</td><td>H*</td><td>H*</td><td>H!</td><td>H*</td><td>H*</td></tr>
<tr><td>62</td><td>R*</td><td>R*</td><td>R*</td><td>R*</td><td>P*</td><td>R*</td><td>R*</td><td>R*</td><td>R*</td><td>R*</td><td>R*</td><td>R*</td><td>R*</td><td>R*</td><td>N</td><td>R*</td><td>R*</td></tr>
<tr><td>63</td><td>I!</td><td>V*</td><td>V*</td><td>V*</td><td>P*</td><td>I!</td><td>I!</td><td>I!</td><td>S</td><td>V!</td><td>V|</td><td>V|</td><td>V*</td><td>I|</td><td>V!</td><td>V!</td><td>I</td></tr>
<tr><td>64</td><td>I*</td><td>I*</td><td>I*</td><td>I*</td><td>P|</td><td>I*</td><td>I*</td><td>I*</td><td>I*</td><td>I!</td><td>I*</td><td>V*</td><td>V*</td><td>I*</td><td>Q!</td><td>V|</td><td>V*</td></tr>
<tr><td>65</td><td>P*</td><td>K|</td><td>K*</td><td>P*</td><td>P|</td><td>K</td><td>K|</td><td>K*</td><td>R!</td><td>P!</td><td>K*</td><td>K*</td><td>K*</td><td>P*</td><td>R</td><td>P!</td><td>Q</td></tr>
<tr><td>66</td><td>G|</td><td>D!</td><td>D*</td><td>S</td><td>G*</td><td>D</td><td>K</td><td>G|</td><td>N!</td><td>D!</td><td>D*</td><td>D!</td><td>N*</td><td>Q!</td><td>D</td><td>G!</td><td>N!</td></tr>
<tr><td>67</td><td>F*</td><td>F*</td><td>F*</td><td>F*</td><td>F*</td><td>F*</td><td>F*</td><td>F*</td><td>F*</td><td>F*</td><td>F*</td><td>F*</td><td>F*</td><td>F*</td><td>F|</td><td>F*</td><td>G|</td></tr>
<tr><td>68</td><td>M|</td><td>M*</td><td>M*</td><td>M*</td><td>K!</td><td>M!</td><td>M*</td><td>M*</td><td>V</td><td>V</td><td>M*</td><td>M*</td><td>M*</td><td>M*</td><td>I!</td><td>I*</td><td>W*</td></tr>
<tr><td>69</td><td>C*</td><td>I*</td><td>I*</td><td>C*</td><td>Q!</td><td>I</td><td>I!</td><td>I*</td><td>I!</td><td>C*</td><td>I!</td><td>V!</td><td>I*</td><td>C*</td><td>I</td><td>V!</td><td>I</td></tr>
<tr><td>70</td><td>Q*</td><td>Q*</td><td>Q*</td><td>Q*</td><td>V*</td><td>Q*</td><td>Q*</td><td>Q*</td><td>Q*</td><td>Q*</td><td>Q*</td><td>Q*</td><td>Q*</td><td>Q*</td><td>Q*</td><td>Q*</td><td>Q*</td></tr>
<tr><td>71</td><td>G*</td><td>G*</td><td>G*</td><td>A!</td><td>F*</td><td>G!</td><td>G*</td><td>T*</td><td>G*</td><td>G*</td><td>G!</td><td>G*</td><td>G*</td><td>G!</td><td>T!</td><td>G*</td><td>G*</td></tr>
<tr><td>72</td><td>G*</td><td>G*</td><td>G*</td><td>G*</td><td>C!</td><td>G*</td><td>G*</td><td>G*</td><td>G*</td><td>G*</td><td>G*</td><td>G*</td><td>G*</td><td>G*</td><td>G*</td><td>G*</td><td>G*</td></tr>
<tr><td>73</td><td>D*</td><td>D*</td><td>D*</td><td>D*</td><td>L*</td><td>D*</td><td>D*</td><td>D*</td><td>D*</td><td>D*</td><td>D*</td><td>D*</td><td>D*</td><td>D*</td><td>D*</td><td>D*</td><td>D*</td></tr>
<tr><td>74</td><td>F|</td><td>F*</td><td>I!</td><td>F*</td><td>S*</td><td>P*</td><td>F!</td><td>P*</td><td>P*</td><td>I*</td><td>F|</td><td>F*</td><td>F*</td><td>F*</td><td>P*</td><td>P*</td><td>I*</td></tr>
<tr><td>75</td><td>T*</td><td>T*</td><td>T*</td><td>T*</td><td>L*</td><td>T*</td><td>S!</td><td>T|</td><td>T|</td><td>T*</td><td>V</td><td>S|</td><td>S!</td><td>T*</td><td>T</td><td>T!</td><td>V</td></tr>
<tr><td>76</td><td>R</td><td>R!</td><td>T</td><td>N*</td><td>P</td><td>G*</td><td>N</td><td>G*</td><td>G|</td><td>K</td><td>N</td><td>E!</td><td>E*</td><td>N*</td><td>G!</td><td>G*</td><td>Y</td></tr>
<tr><td>77</td><td>H|</td><td>G*</td><td>G|</td><td>H*</td><td>R</td><td>-</td><td>Q</td><td>-</td><td>-</td><td>H</td><td>G!</td><td>G*</td><td>G*</td><td>H*</td><td>-</td><td>-</td><td>G</td></tr>
</table>

<table>
<tr><td></td><td></td><td></td><td></td><td></td><td></td><td></td><td></td><td></td><td></td><td></td><td></td><td></td><td></td><td></td><td></td><td></td><td></td></tr>
<tr><td>78</td><td>N*</td><td>D*</td><td>D*</td><td>N*</td><td>T|</td><td>T*</td><td>N!</td><td>T|</td><td>T|</td><td>D|</td><td>D*</td><td>N*</td><td>N*</td><td>N*</td><td>T|</td><td>T!</td><td>K</td></tr>
<tr><td>79</td><td>G*</td><td>G*</td><td>G*</td><td>G*</td><td>G*</td><td>G*</td><td>G*</td><td>G*</td><td>G*</td><td>G*</td><td>G*</td><td>G*</td><td>G*</td><td>G*</td><td>G*</td><td>G*</td><td>G*</td></tr>
<tr><td>80</td><td>T*</td><td>T*</td><td>T!</td><td>T*</td><td>R!</td><td>R|</td><td>T*</td><td>M!</td><td>T</td><td>T*</td><td>T!</td><td>R</td><td>K!</td><td>T*</td><td>R</td><td>S</td><td>D</td></tr>
<tr><td>81</td><td>G*</td><td>G*</td><td>G*</td><td>G*</td><td>G|</td><td>G*</td><td>G*</td><td>G*</td><td>G*</td><td>G*</td><td>G*</td><td>G*</td><td>G*</td><td>G*</td><td>G*</td><td>G*</td><td>N</td></tr>
<tr><td>82</td><td>G|</td><td>G*</td><td>G*</td><td>G*</td><td>G*</td><td>G*</td><td>G*</td><td>G*</td><td>G*</td><td>G*</td><td>V</td><td>G*</td><td>G*</td><td>G*</td><td>G*</td><td>G*</td><td>G*</td></tr>
<tr><td>83</td><td>K!</td><td>K!</td><td>V</td><td>K|</td><td>N</td><td>A</td><td>E*</td><td>E*</td><td>E</td><td>Q</td><td>A</td><td>E*</td><td>E*</td><td>K*</td><td>E!</td><td>E!</td><td>E*</td></tr>
<tr><td>84</td><td>S|</td><td>S*</td><td>S*</td><td>S*</td><td>S*</td><td>S*</td><td>S*</td><td>S*</td><td>S*</td><td>S*</td><td>S*</td><td>S*</td><td>S*</td><td>S*</td><td>S*</td><td>S*</td><td>S*</td></tr>
<tr><td>85</td><td>I|</td><td>I*</td><td>I*</td><td>I*</td><td>I*</td><td>I*</td><td>I*</td><td>I*</td><td>Y</td><td>I*</td><td>I*</td><td>I*</td><td>I*</td><td>I*</td><td>I</td><td>I!</td><td>I*</td></tr>
<tr><td>86</td><td>Y*</td><td>Y*</td><td>Y*</td><td>Y*</td><td>W*</td><td>Y|</td><td>Y*</td><td>W*</td><td>W*</td><td>Y*</td><td>Y*</td><td>Y*</td><td>Y*</td><td>Y*</td><td>F!</td><td>Y*</td><td>Y|</td></tr>
<tr><td>87</td><td>G|</td><td>G*</td><td>G*</td><td>G*</td><td>G!</td><td>G*</td><td>G*</td><td>G*</td><td>G!</td><td>G*</td><td>R</td><td>G*</td><td>G*</td><td>G*</td><td>G</td><td>G*</td><td>G*</td></tr>
<tr><td>88</td><td>-</td><td>-</td><td>-</td><td>-</td><td>-</td><td>-</td><td>-</td><td>-</td><td>-</td><td>-</td><td>-</td><td>-</td><td>-</td><td>-</td><td>Q</td><td>-</td><td>-</td></tr>
<tr><td>89</td><td>-</td><td>-</td><td>-</td><td>-</td><td>-</td><td>-</td><td>-</td><td>-</td><td>-</td><td>-</td><td>-</td><td>-</td><td>-</td><td>-</td><td>L</td><td>-</td><td>-</td></tr>
<tr><td>90</td><td>-</td><td>-</td><td>-</td><td>-</td><td>-</td><td>-</td><td>-</td><td>-</td><td>-</td><td>-</td><td>-</td><td>-</td><td>-</td><td>-</td><td>Y!</td><td>-</td><td>-</td></tr>
<tr><td>91</td><td>-</td><td>-</td><td>-</td><td>-</td><td>-</td><td>-</td><td>-</td><td>-</td><td>-</td><td>-</td><td>-</td><td>-</td><td>-</td><td>-</td><td>G*</td><td>-</td><td>-</td></tr>
<tr><td>92</td><td>-</td><td>-</td><td>-</td><td>-</td><td>-</td><td>-</td><td>-</td><td>-</td><td>-</td><td>-</td><td>-</td><td>-</td><td>-</td><td>-</td><td>D</td><td>-</td><td>-</td></tr>
<tr><td>93</td><td>-</td><td>-</td><td>-</td><td>-</td><td>-</td><td>-</td><td>-</td><td>-</td><td>-</td><td>-</td><td>-</td><td>-</td><td>-</td><td>-</td><td>Q!</td><td>-</td><td>-</td></tr>
<tr><td>94</td><td>-</td><td>-</td><td>-</td><td>-</td><td>-</td><td>-</td><td>-</td><td>-</td><td>-</td><td>-</td><td>-</td><td>-</td><td>-</td><td>-</td><td>A|</td><td>-</td><td>-</td></tr>
<tr><td>95</td><td>E|</td><td>E</td><td>E</td><td>S</td><td>K</td><td>K</td><td>E</td><td>G*</td><td>K!</td><td>D</td><td>G</td><td>G*</td><td>G*</td><td>K</td><td>S</td><td>A</td><td>P!</td></tr>
<tr><td>96</td><td>K*</td><td>R!</td><td>T</td><td>R!</td><td>K|</td><td>Q</td><td>K|</td><td>E*</td><td>P</td><td>K</td><td>P</td><td>F</td><td>Y*</td><td>K*</td><td>F!</td><td>P*</td><td>T!</td></tr>
<tr><td>97</td><td>F*</td><td>F*</td><td>F*</td><td>F*</td><td>F*</td><td>F*</td><td>F*</td><td>F*</td><td>F*</td><td>F*</td><td>F*</td><td>F*</td><td>F*</td><td>F*</td><td>F!</td><td>F*</td><td>F*</td></tr>
<tr><td>98</td><td>E</td><td>P</td><td>P!</td><td>P|</td><td>E</td><td>E</td><td>E!</td><td>E*</td><td>K</td><td>E*</td><td>A</td><td>E</td><td>K!</td><td>D!</td><td>E!</td><td>K|</td><td>E*</td></tr>
<tr><td>99</td><td>D*</td><td>D*</td><td>D*</td><td>D*</td><td>D*</td><td>D*</td><td>D*</td><td>D*</td><td>D*</td><td>D*</td><td>D*</td><td>D*</td><td>D*</td><td>D*</td><td>A</td><td>D*</td><td>D*</td></tr>
<tr><td>100</td><td>E*</td><td>E*</td><td>E*</td><td>E*</td><td>E*</td><td>E*</td><td>E*</td><td>E*</td><td>E*</td><td>E*</td><td>E*</td><td>E*</td><td>E*</td><td>E*</td><td>E*</td><td>E*</td><td>E*</td></tr>
<tr><td>101</td><td>-</td><td>-</td><td>-</td><td>-</td><td>Y</td><td>L!</td><td>-</td><td>F*</td><td>F!</td><td>-</td><td>-</td><td>-</td><td>-</td><td>-</td><td>K</td><td>F*</td><td>-</td></tr>
<tr><td>102</td><td>N*</td><td>N*</td><td>N*</td><td>N*</td><td>S</td><td>H</td><td>N!</td><td>H|</td><td>R</td><td>N|</td><td>N!</td><td>S!</td><td>N*</td><td>N*</td><td>V</td><td>H*</td><td>N</td></tr>
<tr><td>103</td><td>F|</td><td>F*</td><td>F*</td><td>F*</td><td>E!</td><td>P</td><td>F*</td><td>S</td><td>P</td><td>F*</td><td>F*</td><td>F*</td><td>F*</td><td>F*</td><td>P|</td><td>S!</td><td>F|</td></tr>
<tr><td>104</td><td>I!</td><td>K*</td><td>K|</td><td>T</td><td>Y</td><td>D</td><td>H</td><td>T!</td><td>N</td><td>D</td><td>K</td><td>A</td><td>I!</td><td>I</td><td>R</td><td>R*</td><td>S</td></tr>
<tr><td>105</td><td>L|</td><td>L*</td><td>L*</td><td>L*</td><td>L|</td><td>L*</td><td>Y</td><td>L*</td><td>L!</td><td>V|</td><td>L</td><td>V</td><td>L*</td><td>L*</td><td>I!</td><td>L*</td><td>V</td></tr>
<tr><td>106</td><td>K*</td><td>K!</td><td>K*</td><td>K*</td><td>K*</td><td>K!</td><td>K|</td><td>R*</td><td>S</td><td>K!</td><td>R!</td><td>K*</td><td>K*</td><td>K*</td><td>K!</td><td>R*</td><td>P</td></tr>
<tr><td>107</td><td>H*</td><td>H*</td><td>H*</td><td>H*</td><td>H*</td><td>F!</td><td>H*</td><td>H*</td><td>H*</td><td>H*</td><td>H*</td><td>H*</td><td>H*</td><td>H*</td><td>H*</td><td>F|</td><td>H*</td></tr>
<tr><td>108</td><td>T*</td><td>Y!</td><td>Y!</td><td>V</td><td>N</td><td>T|</td><td>D!</td><td>D*</td><td>T</td><td>T*</td><td>S</td><td>N</td><td>D*</td><td>T*</td><td>K</td><td>N</td><td>N</td></tr>
<tr><td>109</td><td>G*</td><td>G|</td><td>G*</td><td>G*</td><td>V</td><td>G*</td><td>R</td><td>R*</td><td>G</td><td>G|</td><td>A</td><td>K!</td><td>R*</td><td>G!</td><td>K!</td><td>R*</td><td>K</td></tr>
<tr><td>110</td><td>P|</td><td>P</td><td>I!</td><td>P*</td><td>R*</td><td>A*</td><td>E</td><td>P*</td><td>R*</td><td>P*</td><td>P|</td><td>E</td><td>A*</td><td>P|</td><td>K</td><td>R*</td><td>R*</td></tr>
<tr><td>111</td><td>G|</td><td>G|</td><td>G*</td><td>G*</td><td>G*</td><td>G*</td><td>G!</td><td>Y*</td><td>G*</td><td>G*</td><td>G*</td><td>F|</td><td>F*</td><td>G*</td><td>G*</td><td>G*</td><td>G*</td></tr>
<tr><td>112</td><td>I*</td><td>W!</td><td>W*</td><td>V!</td><td>V</td><td>I!</td><td>L!</td><td>T*</td><td>I</td><td>L|</td><td>L!</td><td>L*</td><td>L*</td><td>L</td><td>T</td><td>L*</td><td>V</td></tr>
<tr><td>113</td><td>L*</td><td>V!</td><td>V*</td><td>L*</td><td>V!</td><td>L!</td><td>L*</td><td>L|</td><td>L!</td><td>L*</td><td>L*</td><td>L*</td><td>L*</td><td>L|</td><td>V!</td><td>V!</td><td>L*</td></tr>
<tr><td>114</td><td>S*</td><td>S*</td><td>S*</td><td>S*</td><td>S|</td><td>A!</td><td>S*</td><td>S*</td><td>S*</td><td>S*</td><td>S*</td><td>S*</td><td>S*</td><td>S*</td><td>S|</td><td>A|</td><td>G*</td></tr>
<tr><td>115</td><td>M*</td><td>M*</td><td>M*</td><td>M*</td><td>M*</td><td>M*</td><td>M*</td><td>M*</td><td>M*</td><td>M*</td><td>M*</td><td>M*</td><td>M*</td><td>M*</td><td>M|</td><td>M*</td><td>M*</td></tr>
<tr><td>116</td><td>A*</td><td>A*</td><td>A*</td><td>A*</td><td>A*</td><td>A*</td><td>A*</td><td>A*</td><td>A*</td><td>A*</td><td>A*</td><td>A*</td><td>A*</td><td>A*</td><td>V!</td><td>A*</td><td>A!</td></tr>
<tr><td>117</td><td>N*</td><td>N*</td><td>N*</td><td>N*</td><td>N*</td><td>N*</td><td>N*</td><td>N*</td><td>N*</td><td>N*</td><td>N*</td><td>N*</td><td>N*</td><td>N*</td><td>N!</td><td>N*</td><td>N*</td></tr>
</table>

118	A*	A|	A!	A*	N!	A	A!	A|	S	Q	S|	R*	R*	S*	N	A!	K!
119	G*	G*	G*	G*	G*	G*	G*	G*	G*	G*	G*	G*	G*	G*	G|	G*	G*
120	P*	K!	P*	P*	P*	P|	R	S	P	Q!	P	K|	K*	P!	S	S	R
121	N*	D*	D*	N*	N|	D!	N	N!	N!	N!	S	D!	H!	N*	D	H!	H*
122	T*	T*	T*	T*	T!	T|	T*	T*	S!	T*	T|	T*	T*	T|	Q	D*	S!
123	N*	N*	N*	N*	N*	N*	N*	N*	N*	N*	N*	N*	N*	N*	H	N*	N*
124	G|	G*	G*	G*	G!	G*	G*	G*	R	N|	G*	G*	G*	G*	G|	G|	G!
125	S*	S*	S*	S*	S*	S*	S*	S*	S*	S*	C*	S*	S*	S*	S*	S*	S*
126	Q*	Q*	Q*	Q*	Q*	Q*	Q*	Q*	Q*	Q*	Q*	Q*	Q*	Q*	Q*	Q*	Q*
127	F*	F*	F*	F*	F*	F*	F*	F*	F*	F*	F*	F*	F*	F*	F*	F*	F*
128	F*	F*	F*	F*	F*	F*	F*	F*	F*	V|	F*	F*	F*	F*	L	F*	Y*
129	I*	I*	I*	I*	I!	V	I|	I!	I|	I*	I!	I*	I*	L	I!	F*	I*
130	C*	T*	T	C*	T|	T*	T*	T*	T!	T*	T*	T*	T*	T!	T*	T*	T*
131	T|	T*	L	T*	Y*	L*	T*	V*	F!	L*	C*	T*	T*	C	T	L|	L|
132	A	V!	T!	I	G	A!	V!	V|	R!	K*	S	K|	K*	D!	G	G!	Q*
133	K*	K	K|	K*	K	P*	P!	P*	S	K*	K!	P!	P!	K!	E	R	A
134	-	-	-	-	-	-	-	-	-	-	-	-	-	-	N	-	-
135	T|	T*	P!	T*	Q|	T!	T*	T*	C	A*	C|	T	A*	T*	L!	A!	T
136	E|	A	T	D	P|	Q	P!	P*	A	E*	D	P*	P*	D|	D	D!	P!
137	W|	W*	W*	W*	H!	W	H*	W*	Y	H!	W!	H*	H*	W*	Y!	E*	Y!
138	L*	L*	L*	L*	L*	L*	L*	L*	L*	L*	L*	L*	L*	L*	L*	L*	L!
139	D*	D*	D*	D*	D|	D*	D*	D*	D!	D*	D*	D*	D*	D*	D|	N!	D*
140	G*	G*	G*	G|	M	G!	G|	N*	K	F|	G	G|	G*	G|	G	N|	R
141	K*	K!	K!	K!	K|	K*	K*	K*	K*	K*	K*	H	V*	K*	V	K*	K!
142	H|	H*	H*	H*	Y*	H*	H*	H*	H*	H*	H*	H*	H*	H*	H*	H|	F!
143	V*	V*	V*	V*	T*	T!	V*	T!	T|	V*	V*	V*	V*	V*	T!	T*	V*
144	V|	V*	V*	V*	V	I*	V*	V*	I!	V!	V*	V*	V*	V*	V|	I|	A*
145	F*	F*	F*	F*	F*	F*	F*	F*	F*	F*	F*	F*	F*	F*	F*	F*	F*
146	G*	G*	G*	G*	G*	G*	G*	G*	G*	G*	G*	G*	G*	G*	G*	G*	G*
147	K	K!	K!	H	K!	R*	Q	R*	R	F|	K	Q	L*	E	E!	K*	Q!
148	V*	V!	V!	V*	V!	V!	V*	V!	V!	V*	I!	V*	V*	V!	V!	V!	L
149	K|	L|	I|	K|	I*	C	I	T!	V|	K!	I	I|	I*	T	T	T!	I
150	-	-	-	-	-	-	-	-	-	-	-	-	-	-	-	G*	-
151	E|	E!	D!	E|	D|	Q	K!	K*	G!	D|	D|	S*	S*	E!	E*	D!	E|
152	G|	G*	G*	G*	G*	G*	G*	G*	G|	G*	G|	G*	G*	G*	G*	T*	G|
153	M!	M!	M!	M|	L	I	I	M*	F	M*	L|	Q!	F*	L	M	V	T
154	N!	E!	T!	D|	E!	G	G!	E*	D	D|	L*	E|	E*	D*	D!	Y|	E
155	I	V|	V|	V*	T	M	V!	V|	V	T	V	V|	V*	V*	I	N*	V!
156	V|	V*	V*	V|	L*	V	A	V|	L*	V*	M	V!	I*	L!	I	M!	L*
157	E!	R	H	K|	D|	N	R!	Q*	T	K!	R*	R	E!	R!	K	L|	K

<table>
<tr><td>158</td><td>A!</td><td>K|</td><td>S|</td><td>K|</td><td>E</td><td>R|</td><td>I</td><td>R!</td><td>A</td><td>K!</td><td>K*</td><td>E</td><td>Q!</td><td>Q</td><td>K</td><td>R!</td><td>Q</td></tr>
<tr><td>159</td><td>M!</td><td>V</td><td>I</td><td>I!</td><td>L!</td><td>V</td><td>L</td><td>I*</td><td>M</td><td>I!</td><td>I|</td><td>I!</td><td>I*</td><td>I!</td><td>I</td><td>L*</td><td>L!</td></tr>
<tr><td>160</td><td>E*</td><td>E*</td><td>E*</td><td>E*</td><td>E*</td><td>G|</td><td>E*</td><td>S|</td><td>E*</td><td>E!</td><td>E*</td><td>E*</td><td>E*</td><td>E*</td><td>N*</td><td>S</td><td>E|</td></tr>
<tr><td>161</td><td>R</td><td>S</td><td>L</td><td>S!</td><td>K*</td><td>M</td><td>N</td><td>N!</td><td>N</td><td>S!</td><td>N|</td><td>N</td><td>N!</td><td>A</td><td>E!</td><td>E|</td><td>L</td></tr>
<tr><td>162</td><td>F!</td><td>T!</td><td>Q!</td><td>F!</td><td>L</td><td>V|</td><td>V</td><td>V!</td><td>V</td><td>F!</td><td>V!</td><td>Q!</td><td>L*</td><td>Q</td><td>T</td><td>V</td><td>V</td></tr>
<tr><td>163</td><td>G*</td><td>K</td><td>A</td><td>G*</td><td>P!</td><td>E</td><td>E</td><td>K*</td><td>E</td><td>G*</td><td>P</td><td>K|</td><td>K*</td><td>G|</td><td>F</td><td>D</td><td>P</td></tr>
<tr><td>164</td><td>S|</td><td>T|</td><td>T|</td><td>S*</td><td>V!</td><td>T|</td><td>V</td><td>V!</td><td>S</td><td>S|</td><td>T!</td><td>T|</td><td>T*</td><td>S</td><td>V</td><td>I</td><td>T|</td></tr>
<tr><td>165</td><td>-</td><td>D</td><td>D</td><td>-</td><td>N</td><td>N</td><td>-</td><td>N|</td><td>D!</td><td>-</td><td>G!</td><td>D*</td><td>D*</td><td>-</td><td>D*</td><td>D!</td><td>-</td></tr>
<tr><td>166</td><td>R!</td><td>S</td><td>G</td><td>K</td><td>E</td><td>S</td><td>K</td><td>P*</td><td>P</td><td>P!</td><td>P!</td><td>A</td><td>A</td><td>K|</td><td>K</td><td>D</td><td>Q</td></tr>
<tr><td>167</td><td>N!</td><td>R</td><td>H</td><td>S!</td><td>K!</td><td>Q</td><td>G</td><td>K*</td><td>K</td><td>K</td><td>N!</td><td>A</td><td>A*</td><td>D</td><td>D</td><td>D</td><td>N|</td></tr>
<tr><td>168</td><td>-</td><td>-</td><td>-</td><td>-</td><td>T</td><td>-</td><td>-</td><td>T*</td><td>T</td><td>-</td><td>-</td><td>-</td><td>-</td><td>-</td><td>-</td><td>-</td><td>-</td></tr>
<tr><td>169</td><td>G|</td><td>D*</td><td>D*</td><td>G*</td><td>Y</td><td>D*</td><td>E</td><td>D*</td><td>D!</td><td>G*</td><td>N|</td><td>S*</td><td>S*</td><td>G|</td><td>F</td><td>E!</td><td>E|</td></tr>
<tr><td>170</td><td>K|</td><td>K!</td><td>R!</td><td>R!</td><td>R!</td><td>R|</td><td>K</td><td>K*</td><td>R!</td><td>S</td><td>K</td><td>K!</td><td>R*</td><td>K</td><td>V</td><td>R*</td><td>R|</td></tr>
<tr><td>171</td><td>T|</td><td>P*</td><td>P*</td><td>T!</td><td>P*</td><td>P*</td><td>P*</td><td>P*</td><td>P*</td><td>V!</td><td>P*</td><td>P*</td><td>P*</td><td>P!</td><td>P*</td><td>P*</td><td>P|</td></tr>
<tr><td>172</td><td>S|</td><td>L</td><td>L</td><td>S|</td><td>L!</td><td>V</td><td>A</td><td>Y*</td><td>K</td><td>C</td><td>K|</td><td>F</td><td>Y*</td><td>K</td><td>Y!</td><td>H</td><td>I</td></tr>
<tr><td>173</td><td>K*</td><td>K</td><td>T</td><td>K|</td><td>N</td><td>D</td><td>K</td><td>E|</td><td>E</td><td>R</td><td>L</td><td>A!</td><td>A*</td><td>Q!</td><td>Q!</td><td>N</td><td>H</td></tr>
<tr><td>174</td><td>K|</td><td>D!</td><td>N!</td><td>K!</td><td>D</td><td>D</td><td>L</td><td>D*</td><td>E</td><td>R!</td><td>P*</td><td>E!</td><td>D*</td><td>K|</td><td>D|</td><td>P|</td><td>M</td></tr>
<tr><td>175</td><td>I|</td><td>V|</td><td>C|</td><td>I|</td><td>V</td><td>V!</td><td>C</td><td>V!</td><td>I!</td><td>I!</td><td>V*</td><td>V|</td><td>V*</td><td>V!</td><td>I|</td><td>H!</td><td>C!</td></tr>
<tr><td>176</td><td>T|</td><td>I</td><td>S</td><td>V</td><td>H</td><td>K</td><td>V</td><td>S!</td><td>R</td><td>T</td><td>V*</td><td>R!</td><td>R*</td><td>I</td><td>R*</td><td>K!</td><td>R</td></tr>
<tr><td>177</td><td>I|</td><td>I|</td><td>I|</td><td>I|</td><td>I!</td><td>I|</td><td>I</td><td>I*</td><td>I</td><td>I*</td><td>I!</td><td>I!</td><td>V*</td><td>I!</td><td>I*</td><td>I*</td><td>I!</td></tr>
<tr><td>178</td><td>A</td><td>A</td><td>I</td><td>T!</td><td>K</td><td>I</td><td>A</td><td>I*</td><td>D</td><td>T!</td><td>S</td><td>L</td><td>I!</td><td>A</td><td>N</td><td>K</td><td>T</td></tr>
<tr><td>179</td><td>D|</td><td>D!</td><td>N!</td><td>D|</td><td>D</td><td>K</td><td>E</td><td>N|</td><td>A</td><td>E</td><td>Q</td><td>S</td><td>D*</td><td>D</td><td>H*</td><td>S</td><td>D</td></tr>
<tr><td>180</td><td>C|</td><td>C!</td><td>S!</td><td>C*</td><td>I</td><td>A</td><td>-</td><td>I|</td><td>T</td><td>C*</td><td>C</td><td>C*</td><td>C*</td><td>C|</td><td>T*</td><td>C</td><td>S</td></tr>
<tr><td>181</td><td>G|</td><td>G|</td><td>G|</td><td>G*</td><td>T|</td><td>Y</td><td>-</td><td>T|</td><td>T</td><td>G*</td><td>G</td><td>G*</td><td>G*</td><td>G*</td><td>V!</td><td>E|</td><td>G|</td></tr>
<tr><td>182</td><td>Q!</td><td>K</td><td>K</td><td>Q!</td><td>I|</td><td>P</td><td>-</td><td>V!</td><td>V!</td><td>Q*</td><td>E*</td><td>E!</td><td>V!</td><td>E*</td><td>I!</td><td>V|</td><td>D</td></tr>
<tr><td>183</td><td>L</td><td>I</td><td>I</td><td>L|</td><td>H*</td><td>S</td><td>-</td><td>K*</td><td>F!</td><td>I!</td><td>M*</td><td>L*</td><td>L|</td><td>Y</td><td>L*</td><td>L*</td><td>P</td></tr>
<tr><td>184</td><td>E</td><td>E</td><td>D</td><td>S</td><td>A*</td><td>G</td><td>-</td><td>-</td><td>V!</td><td>-</td><td>-</td><td>-</td><td>A</td><td>V</td><td>D!</td><td>F</td><td>Y</td></tr>
</table>
