# Supplementary material for: Compression of Large Sets of Sequence Data Reveals Fine Diversification of Functional Profiles in Multigene Families of Proteins: A Study for Peptidyl-Prolyl cis/trans Isomerases (PPIase)
Source: Biomolecules. 2019 Feb 11;9(2):59. doi: 10.3390/biom9020059 (PMC6406343; doi:10.3390/biom9020059)
Supplement: Supplementary file 1 [file biomolecules-09-00059-s001.zip › vertical MSA of FKBPs.pdf]

Arrangement of human FKBPs

FKBPs residing in the endoplasmic reticulum

	Fk1a	Fk1b	Fk3	Fk4a	Fk4b	Fk5a	Fk5b	Fk6	Fk8	Fk2	Fk7	Fk11	Fk14	Fk9a	Fk9b	Fk9c	Fk9d	Fk10a	Fk10b	Fk10c	Fk10d
1	G	G!	K|	G|	I|	G*	I*	G	L|	Q|	K!	Q	K!	Q	Q|	S	S!	V|	Q	Q	E
2	V!	V!	Y!	V!	I	V	I!	V	R	I*	I!	V!	I	I	I	I!	I	I!	V	L	I!
3	Q	E	T	L	R|	L*	R*	L	K|	G*	E*	E|	E|	E|	H	E!	T	E!	S	E!	R
4	V	I	K*	K!	R	K!	R*	K*	K!	V!	V|	T!	V*	R	T*	N!	S!	R*	T!	T	T
5	E	E	S	V	I	I!	T|	D	T	K*	L!	L!	L!	R	Y*	K	H!	Y|	L	L	L
6	T	T	V	I!	Q	V|	K|	V	L!	K	H	V!	Q	F!	F	V	Y	H	L	E	S
7	I	I	L|	K	T	K!	R	I	V	R!	R	E	K|	V	K!	V!	K	I!	R	L	R
8	S	S	K*	R	R	R	K*	R	P	V	P*	P|	P*	P*	P*	P*	P!	P*	P!	P*	P*
9	P	P!	K|	E!	G|	V*	G*	E	G!	D	E!	P!	F|	D	P	E!	P|	R	P	P	S
10	G|	G!	G*	G*	E	G	E|	G|	P	H	N	E!	I	E	S	N	D*	A	H!	G	E
11	D!	D	D|	T	--	N	--	A	P	--	--	P	--	--	--	--	--	--	--	--	T
12	G!	G!	K|	G|	G	G	G*	G!	G	C*	C*	C|	C*	C|	C*	C*	C	C*	C	C*	C*
13	R	R!	T!	T	Y	E!	Y*	--	S	P	S	A	H	P	P	E	S	P	P	V	N|
14	T	T|	N!	E	A	E	S!	D	S	I	K	E	R*	R*	R*	R*	V*	R*	R	R*	E
15	F	F|	F|	M	K	T*	N!	L	R	K!	T	P	K!	T	T!	I	L	E|	M	R!	T|
16	P|	P|	P*	P*	P*	P*	P*	V	P*	S!	S|	A	T!	V|	I	S!	S	V!	V	A	T
17	K	K!	K|	M	N	M*	N*	A	V	R	K!	A	K*	R	Q!	Q	K!	Q!	Q	G!	K
18	R	K!	K*	I	E	I*	E!	P	K	K!	K!	F	G	S	V*	S	K!	M	D	A	L
19	G|	G!	G*	G*	G!	G*	G*	D	G!	G*	G*	G|	G*	G|	S*	G*	G*	G|	G	G*	G
20	Q	Q!	D|	D	A!	D|	A|	A	Q	D*	D*	D|	D*	D|	D*	D*	D|	D*	D	D*	D*
21	T	T!	V	R	I	K*	T!	S	V	V	L!	T!	L	F!	F!	F*	Y*	F!	F	F!	F
22	C	C!	V*	V*	V|	V	V*	V	V!	L!	L!	L!	M!	V|	V|	L	L*	V!	V!	M	V!
23	V	V!	H	F	E	Y*	E	L	T	H	N*	H	L*	R|	R*	R*	K!	R*	R*	R*	R
24	V	V|	C*	V|	V	V*	I!	V	V	M!	A|	I!	V*	Y|	Y*	Y*	Y!	Y*	Y*	Y*	Y*
25	H|	H|	W!	H*	A	H*	H|	K	H	H*	H|	H!	H*	H|	H*	H*	H*	H*	H*	H*	H!
26	Y|	Y|	Y*	Y*	L	Y	L|	Y|	L	Y*	Y|	Y!	Y!	Y|	Y*	Y*	Y*	Y!	Y!	Y*	Y*
27	T	T	T*	T	E	K*	E|	S	Q	T!	D|	T!	E!	V	N*	N*	N*	N*	N*	N*	N*
28	G*	G*	G*	G*	G|	G!	G*	G!	T	G*	G|	G*	G*	G|	G*	G*	A*	G!	G!	G*	C*
29	M	M|	T	W	Y	K*	R	Y!	S	K	Y	S	Y!	T	T*	T!	S*	T*	T*	S!	S|
30	L*	L*	L*	L*	Y	L	C!	L	L|	L*	L|	L*	L|	F|	F!	L*	L*	F*	L*	L!	L*
31	E	Q|	Q	L	K	S!	G!	E!	E	E	A!	V	E|	P!	L*	L*	L|	E	L	M	L

```
32 D   N*  D*  D|  D   N*  --  H   N   --  K   --  K   --  --  --  --  --  --  --  --
33 G*  G*  G*  G*  --  G|  G   M   G!  D|  D!  D|  D!  D!  D*  D*  D*  D*  D*  D!  D*
34 K   K*  --  T!  --  K*  --  D   T   G*  G|  G*  G*  G|  G*  G*  G!  G*  G*  G*  G*
35 K!  K*  T|  K   K   K*  R   R   R   T!  S!  R   S!  Q   T*  T*  T*  K!  T!  T   T*
36 --  --  V*  --  L   --  M   P|  V!  E!  K   I   L   K   L*  L   L*  K   S   L   Q
37 F|  F*  F*  F*  F*  F*  F*  F*  Q   F*  F|  I!  F*  F|  F*  F*  L*  F|  F|  F*  L*
38 D*  D*  D*  D*  D   D|  D*  D   E   D*  Y|  D*  H|  D!  D*  D*  D   D*  D*  D*  F!
39 S*  S*  T|  S*  Q   S*  C!  S!  E   S*  C|  T!  S*  S!  S*  S*  S!  S|  T|  S!  T|
40 S*  S*  N*  S*  --  S   --  N   --  S*  S*  S*  T!  S|  S*  S*  T!  S*  S*  S*  S*
41 R!  R*  I!  L   --  H|  --  Y   --  L   R*  L*  H   Y*  H*  Y*  W*  Y!  Y!  Y*  H
42 D!  D*  Q   D   --  D*  --  F   --  P   T!  T   K   D*  N!  S*  N*  D!  S!  S   D
43 R*  R*  T   R|  --  R   --  R   --  Q!  Q   R*  H   R*  R*  R*  L   R|  K|  R*  Y!
44 N   N*  S   K   --  N!  --  K   --  N   N   --  N!  D   M|  N*  G   N   G   N*  G
45 --  --  A   --  --  --  --  --  --  --  E   --  N!  --  --  --  --  --  --  --  --
46 --  --  K!  --  --  --  --  --  --  --  --  --  --  --  --  --  --  --  --  --  --
47 --  --  K*  --  --  --  --  --  --  --  --  --  --  --  --  --  --  --  --  --  --
48 --  --  K*  --  --  --  --  --  --  --  --  --  --  --  --  --  --  --  --  --  --
49 --  --  K*  --  --  --  --  --  --  --  --  --  --  --  --  --  --  --  --  --  --
50 --  --  N   --  --  --  --  --  --  --  --  --  --  --  --  --  --  --  --  --  --
51 --  --  A   --  --  --  --  --  --  --  G*  --  G   --  --  --  --  --  --  --  --
52 K   K*  K!  D   R|  E|  R!  T   P   Q   H*  D|  Q!  S|  K!  R!  K!  T   G   H   A
53 P*  P*  P*  K   E   P*  D|  P   E   P|  P*  P*  P|  T*  T*  T*  T*  L   T   T*  P
54 F*  F*  L*  F*  L   F   V|  R   L   F!  K!  L*  I   F!  Y*  F!  Y*  V   Y   Y*  Q!
55 K   K!  S!  S   R   V*  A   L   V   V   W*  V!  W*  N*  D*  D*  N*  A   D   N|  E
56 F   F*  F*  F*  F*  F   F*  M   F   F*  F*  I   F*  V*  T*  T*  I*  I   T   T*  A
57 M   R!  K*  D   E   S!  T   K   T   S   V*  E|  T*  F|  Y*  Y*  V|  V   Y   Y*  T!
58 L   I*  V*  L*  I   L*  V|  L!  L   L|  L*  L*  L*  V*  V*  I!  L|  V!  V!  I!  L*
59 G*  G*  G*  G|  G*  G*  G*  G!  G|  G*  G*  G*  G*  G*  G*  G*  G*  G*  G*  G*  G*
60 K   K   V   K   E!  K*  E|  E   --  T   V*  Q   I*  K|  I*  Q   S*  V   S   Q   A
61 Q   Q!  G*  G!  G|  G|  G!  D   D!  G*  G*  K   L   G!  G*  G*  G*  G*  G*  G*  N
62 --  --  --  --  E   --  E*  --  --  --  --  --  --  --  --  --  --  --  --  --  --
63 --  --  --  --  N   --  D|  --  --  --  --  --  --  --  --  --  --  --  --  --  --
64 --  --  --  --  L   --  H   --  C   --  --  --  --  --  --  --  --  --  --  --  --
65 E   E*  K!  E   D   Q*  D!  I   D!  Q|  Q!  Q   E*  Q*  W*  Y*  Q!  R|  W|  Y*  K|
66 V*  V*  V*  V!  L   V|  I!  T   V!  V*  V*  V*  A   L*  L*  V*  V|  L|  L|  I   V|
```

```
 67 I*  I*  I*  I   P   I*  P*  L!  I   I*  I*  I*  L!  I*  I*  I*  V*  I*  I*  I*  I*
 68 R   K*  R*  K*  Y   K*  I!  W   Q|  K!  K*  P!  K|  T   P!  P!  L*  T!  K!  P   E
 69 G*  G*  G*  A!  G*  A*  G*  G*  A!  G*  G*  G*  G*  G*  G*  G*  G*  G*  G*  G*  G*
 70 W*  F!  W*  W|  L   W*  I!  M   L|  W*  L*  L*  W*  M*  M*  M|  M   M   M   M*  L*
 71 E!  E*  D*  D*  E|  D|  D|  E!  D!  D*  D*  E*  D*  D*  D*  D*  D*  D*  D*  D*  D
 72 E!  E*  E*  I   R   I!  K!  L   L   Q|  I!  Q*  Q   Q   K   E|  M*  R!  Q!  Q!  T
 73 G*  G*  A!  A!  A|  G*  A*  G!  S   G*  A!  S!  G*  A*  G*  G*  G!  G*  G*  G!  G*
 74 V!  A   L|  I!  I   V   L!  L   V   L*  M|  L*  L*  L*  L*  L*  L   L!  L!  L*  L*
 75 A   A!  L|  A|  Q   A!  E!  L   P   L!  T   L   K   V!  L*  L!  R*  M   L   Q!  Q
 76 Q   Q*  T*  T|  R   T*  K*  S   L!  G   D   D!  G   G*  G*  G*  E*  G*  G*  G*  G
 77 M!  M*  M*  M*  M!  M!  M*  M*  M!  M*  M*  M   M*  M*  M*  V|  M   M*  M*  A   M*
 78 S!  S*  S|  K|  E   K!  Q|  R   D   C*  C*  C*  C*  C*  C*  C*  C   C*  C*  C*  C*
 79 V   L|  K!  V   K   K*  R!  R   V   E   P!  V!  V   V*  V|  I   V*  V*  P*  M   V!
 80 G*  G*  G*  G*  G   G*  E   G!  G   G|  G*  G*  G*  N*  G*  G*  G*  N|  G|  G*  G*
 81 Q   Q*  E*  E*  E*  E   E*  E*  E*  E*  E*  E!  E*  E*  E*  E*  E*  E*  E*  E*  E*
 82 R   R*  K!  V   H   I*  Q   L   T   K!  K*  K*  K!  R*  K!  K!  K*  R|  R|  R   R!
 83 A|  A*  A*  C!  S!  C   C|  A!  A   R*  R*  R!  R*  R!  R*  R*  R*  R*  R*  R*  R*
 84 K   K*  R   H   I   H!  I   R   M   K!  K*  R!  K|  F!  I!  R!  T!  R   K   R*  Q
 85 L|  L!  L|  I   V   L!  L|  F|  V   L*  V!  A   L*  V|  I*  I*  V*  L   I   I!  L
 86 T   T*  E!  T   Y   L*  Y!  L   T   V   V   I   I   K!  T*  V   I   I   I   T   I
 87 I   C*  I*  C|  L   C|  L*  F   A   I*  I|  I*  I!  I*  I   V!  I!  V|  I|  I   V!
 88 S   T*  E|  K   K   K*  G   K   D   P*  P*  P*  P*  P*  P*  P*  P   P*  P*  P*  P*
 89 P!  P*  P!  P!  P   P|  P*  P!  S   S   P|  S   P*  P*  P*  P*  P*  P*  P*  P*  P*
 90 D|  D!  E*  E   S   E*  R   N   K   E   S!  H!  A!  K   F!  H*  H*  H*  F*  H*  H*
 91 Y   V   W*  Y*  Y!  Y*  Y*  Y!  Y!  L|  F!  L*  L*  L*  L*  L*  L*  L*  L*  L!  L!
 92 A!  A*  A*  A*  A   A*  G*  A!  C   G|  A*  A*  G!  A*  A!  G!  G*  G!  A!  A*  A!
 93 Y*  Y*  Y*  Y*  F!  Y*  F*  Y!  Y*  Y*  Y!  Y*  Y*  Y*  Y*  Y*  Y*  Y*  Y*  Y*  H*
 94 G*  G*  G*  G*  G!  G   G*  G!  G*  G*  G*  G*  G*  G*  G*  G*  G|  G*  G*  G*  G*
 95 A   A!  K!  S   S   S!  E   T   P   E   K   K*  K*  N   E*  E*  E*  S|  E|  E*  E*
 96 T   T|  K*  A   V   A*  A   L   Q   R   E   R!  E*  E   D   E*  A*  I   K   N   S
 97 G*  G*  G*  G*  G|  G!  G*  G*  G*  G*  G*  G*  G*  G*  G*  G*  G*  G*  G*  G*  G*
 98 --  --  Q   --  K   --  K|  --  S   --  Y!  --  --  --  --  --  --  --  --  --  --
 99 H   H*  P|  S|  E   S   P!  C|  R   A!  A   F   --  V!  D*  R!  V   L   Y   T   A*
100 P|  P*  D!  P!  K   L!  K   P!  S   P!  E   P*  K|  S   G*  G*  D*  A   G   G*  R
101 G!  G*  A!  P*  F   P*  F   P*  P!  P!  G!  P*  G*  G   K!  N   G*  G!  T!  D   G|
```

```
102 I   V*  K|  K   Q   K*  G   L   Y   K!  K*  S   K*  V!  D!  --  E   L   V   K   --
103 I*  I*  I*  I*  I!  I*  I*  I*  I!  I*  I!  V!  I*  I*  I*  I*  V|  I!  I!  I*  V*
104 P*  P*  P*  P*  P*  P!  E   P|  P|  P*  P*  P*  P*  P*  P*  P*  P*  P*  P*  P*  P*
105 P!  P*  P*  P!  P!  S|  P   P   P   G   P*  A!  P*  P|  G!  G*  G*  P|  P|  G!  G
106 H!  N*  N|  N   N   N!  N!  N   H   G   D!  D*  E!  N!  Q*  S*  S*  D*  Q*  S*  S*
107 A!  A*  A!  A!  A!  A!  A!  T   A!  A!  A*  A|  S*  S!  A*  A*  A*  A!  A!  A*  A*
108 T!  T*  K!  T*  E   T|  E!  T   A   T!  T*  V   T*  V!  S*  V*  V*  T!  S!  V*  V*
109 L*  L*  L*  L*  L   L   L|  V   L!  L*  L!  V!  L*  L*  L*  L*  L*  L*  L*  L*  L*
110 V   I!  T   V   K   F*  I   L   C   V   I!  Q   I!  H*  V*  V*  V*  Y!  V!  I!  L
111 F*  F*  F*  F*  Y|  F*  Y*  F!  L   F*  F*  Y!  F*  F*  F*  F*  F*  F|  F|  F*  F*
112 D!  D*  E*  E|  E   E   E*  E   E   E   E*  D!  N   D*  D*  D*  D|  D*  H*  N!  E!
113 V*  V*  V|  V   L   I|  V*  I   V!  V!  I!  V|  I|  V*  V*  I!  I*  V!  V!  V!  V
114 E*  E*  E*  E|  H   E*  T!  E   T   E!  E*  E*  D!  L*  A   H|  E*  V   L   H|  E*
115 L*  L*  L*  L!  L*  L!  L*  L   L*  L*  L|  L!  L!  L|  L*  V!  L|  L!  L!  V!  L*
116 L!  L*  V|  F   K   L   K|  L   K   L   Y!  I   L!  M   L*  I!  L*  L|  I|  I|  V
117 K   N   D   E   S   D*  S!  D!  T   K   A!  A   E*  D*  D*  D*  E*  D*  D*  D*  S*
118 L   L|  I!  F!  F!  F   F*  F!  A   I|  V*  L*  I*  I   L*  F*  L   V   V   F*  R*
119 E   E*  D   K   E!  K   E!  L   V   E   T   I   R*  W*  H*  H*  V!  W*  H*  H*  E*
```
